# Patient‐ and xenograft‐derived organoids recapitulate pediatric brain tumor features and patient treatments

**DOI:** 10.15252/emmm.202318199

**Published:** 2023-11-30

**Authors:** Chiara Lago, Aniello Federico, Gloria Leva, Norman L Mack, Benjamin Schwalm, Claudio Ballabio, Matteo Gianesello, Luana Abballe, Isabella Giovannoni, Sofia Reddel, Sabrina Rossi, Nicolas Leone, Andrea Carai, Angela Mastronuzzi, Alessandra Bisio, Alessia Soldano, Concetta Quintarelli, Franco Locatelli, Marcel Kool, Evelina Miele, Luca Tiberi

**Affiliations:** ^1^ Armenise‐Harvard Laboratory of Brain Disorders and Cancer, CIBIO Trento Italy; ^2^ Hopp Children's Cancer Center (KiTZ) Heidelberg Germany; ^3^ Division of Paediatric Neurooncology German Cancer Research Center (DKFZ) and German Cancer Consortium (DKTK) Heidelberg Germany; ^4^ Department of Onco‐Hematology, Cell and Gene Therapy, Bambino Gesù Children's Hospital Scientific Institute for Research, Hospitalization and Healthcare (IRCCS) Rome Italy; ^5^ Pathology Unit Bambino Gesù Children's Hospital, IRCCS Rome Italy; ^6^ Neurosurgery Unit, Department of Neurosciences Bambino Gesù Children's Hospital, IRCCS Rome Italy; ^7^ Laboratory of Radiobiology, CIBIO Trento Italy; ^8^ Trento Institute for Fundamental Physics and Application, TIFPA Trento Italy; ^9^ Department of Neuroscience Scuola Internazionale Superiore di Studi Avanzati (SISSA) Trieste Italy; ^10^ Catholic University of the Sacred Heart Rome Italy; ^11^ Princess Máxima Center for Pediatric Oncology Utrecht The Netherlands; ^12^ University Medical Center Utrecht, Utrecht University Utrecht The Netherlands

**Keywords:** brain tumors, organoids, patient‐derived, pediatric cancer, translational applications, Cancer, Methods & Resources

## Abstract

Brain tumors are the leading cause of cancer‐related death in children. Experimental *in vitro* models that faithfully capture the hallmarks and tumor heterogeneity of pediatric brain cancers are limited and hard to establish. We present a protocol that enables efficient generation, expansion, and biobanking of pediatric brain cancer organoids. Utilizing our protocol, we have established patient‐derived organoids (PDOs) from ependymomas, medulloblastomas, low‐grade glial tumors, and patient‐derived xenograft organoids (PDXOs) from medulloblastoma xenografts. PDOs and PDXOs recapitulate histological features, DNA methylation profiles, and intratumor heterogeneity of the tumors from which they were derived. We also showed that PDOs can be xenografted. Most interestingly, when subjected to the same routinely applied therapeutic regimens, PDOs respond similarly to the patients. Taken together, our study highlights the potential of PDOs and PDXOs for research and translational applications for personalized medicine.

## Graphical abstract

Patient‐derived organoids (PDOs) and patient‐derived xenograft organoids (PDXOs) have been established through direct *in vitro* culture of primary tumors and patient‐derived xenograft (PDX)‐derived tumors with the goal to better model pediatric tumors of the central nervous system.

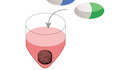

**The paper explained**

**Problem**

CNS tumors are the most common pediatric solid tumors and the main cause of childhood cancer‐related deaths. In recent years, research efforts have improved our understanding of the underlying molecular landscape, contributing to clinical advances, and improving patient life expectancy. However, developing new models that can fully resemble the heterogeneity of these tumors to test personalized treatment strategies remains a challenge.

**Results**

Patient‐derived organoids (PDOs) and patient‐derived xenograft organoids (PDXOs) were established through direct *in vitro* culture of primary ependymomas, medulloblastomas, and low‐grade glial tumors, and from medulloblastoma xenografts, respectively. PDOs and PDXOs were shown to be bona fide replicates of their corresponding primary tumors through genome and mutational status analysis and by DNA methylation profiling. They also maintained the tumoral heterogeneity and the cellular morphology and architecture of the primary tumors, as shown by scRNA sequencing and immunohistological analysis. PDOs and PDXOs could be largely amplified and biobanked, maintaining the features of their parental tumors, even after many passages in culture. The translational potential of this model was shown by treating PDOs and PDXOs with the same therapeutic regimens as the corresponding patients, as the organoids exhibited similar responses to a specific clinical treatment.

**Impact**

These PDOs and PDXOs, derived from human tumors for which there are currently very limited *in vivo* and *in vitro* models, constitute an important proof of concept of their translational application as a reliable tool for wide drug screening, and more generally for personalized medicine.

## Introduction

Central nervous system (CNS) tumors are the most common solid tumor in childhood and the leading cause of cancer death in this population (Siegel *et al*, 2021). Despite the considerable insights in the knowledge on their biology, achieved by extensive genomic and epigenomic analyses (Pollack *et al*, 2019), therapeutic advances are strongly needed to improve the outcome and the quality of life for affected children and those who survive the disease. Indeed, the treatment often implies a high price in terms of late sequelae, especially for children with prognostic favorable tumors and for craniospinal radiation treatment in young children. Pediatric neurooncologists are therefore making huge efforts to apply risk‐adapted treatment protocols and to improve cure rates using new therapeutic regimens (Pollack *et al*, 2019). A key need for identifying effective therapies for pediatric brain tumors lies on the availability of preclinical models faithful to the disease, reflecting the uniqueness of the brain biology, its microenvironment, and the complexity of the cellular heterogeneity and that can be used to predict human response. Molecular intertumoral and intratumoral heterogeneity are among the main factors contributing to the failure of numerous clinical studies. In the last decade, this continuous search for novel model systems has reached an important milestone through the creation of so‐called organoids, artificial mini‐organs that can be grown *in vitro* and reflect the molecular, physiological, and pathological characteristics of human organs (Clevers, 2016). Organoids have become a viable solution to improve the efficiency of preclinical research and limit the use of animal testing in drug discovery (Takahashi, 2019). Normal tissue‐like organoids can indeed be established from induced pluripotent stem cells (iPSCs) or embryonic stem cells (ESCs) (using different protocols to differentiate them into all kinds of different lineages), and these can be engineered to model specific tumor types (Ballabio *et al*, 2020). On the other hand, organoids can also be derived directly from normal and neoplastic tissues (Clevers, 2016).

We have previously established a human organoid‐based model for pediatric medulloblastoma (Ballabio *et al*, 2020) opening new horizons of knowledge on pediatric brain cancer development directly in a human system. This new system is based on the production of organoids from human iPSCs and although it summarizes several characteristics of the original tumors, it does not fully mirror the intratumoral heterogeneity. Therefore, we moved toward other solutions to recapitulate this important feature of pediatric brain cancers. Indeed, maintaining heterogeneity, genetic and phenotypic features, and 3D structure of the parental tumors, usually lost in the 2D culture (Foo *et al*, 2022) would improve the model and its possible applications. Here, we report the successful generation and characterization of patient‐derived organoids (PDOs) from pediatric brain tumor biopsies and patient‐derived xenografts, a powerful new platform to use at the forefront of personalized medicine.

## Results

### *In vitro* culture and xenografting of PDOs

**Figure 1:**
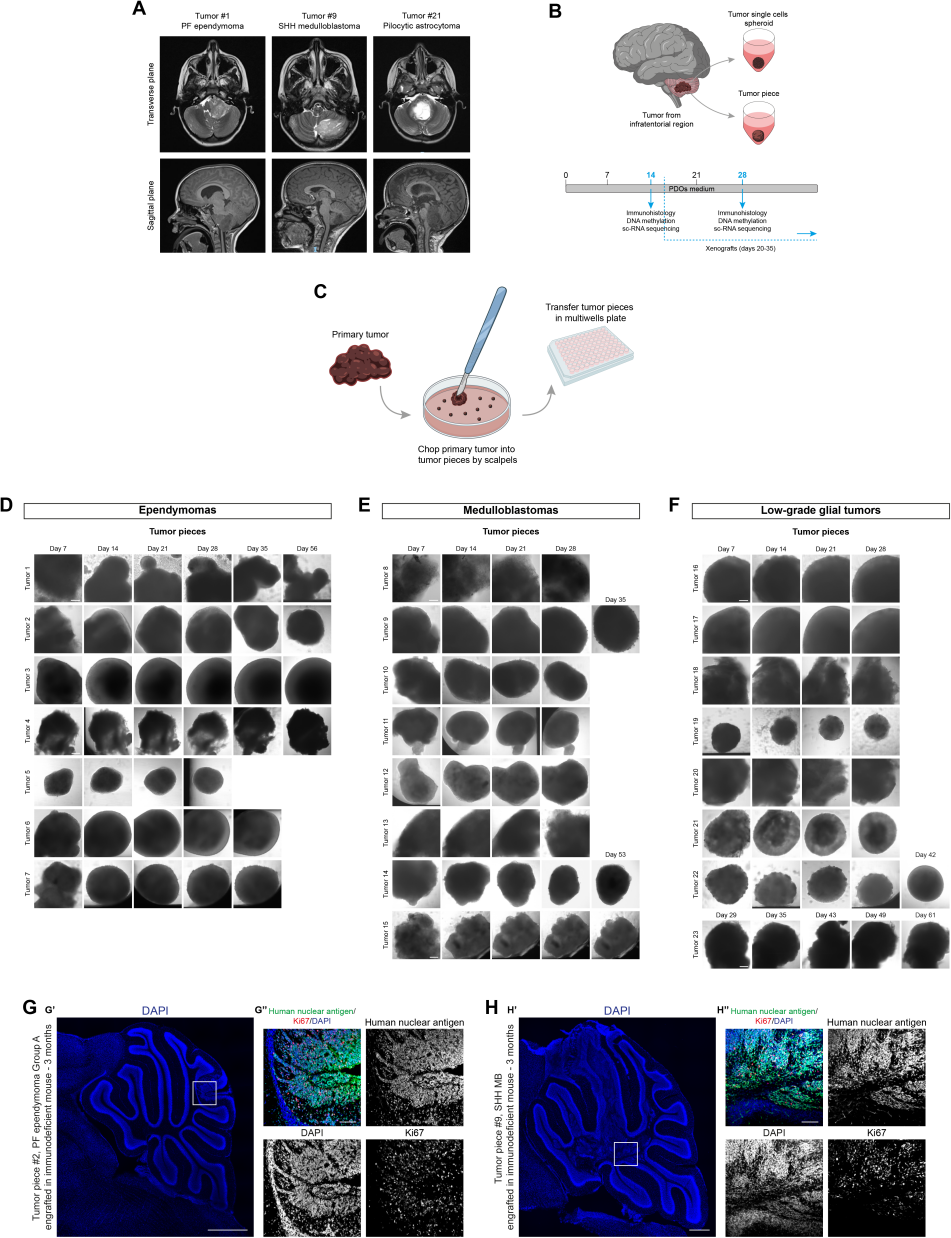
*In vitro* culture of patient‐derived organoids (PDOs) and maintenance of tumorigenic potential *in vivo* AAxial T2‐weighted (upper panels) and sagittal T1‐weighted MPRAGE (lower panels) MRI images relative to the indicated cases.BSchematic representation of primary tumor samples management workflow.CSchematic representation of primary tumor samples management for generation of PDOs as tumor piece.D–FBrightfield images of EPN‐ (D), MB‐ (E), and LGG‐ (F) derived PDOs as tumor pieces at different timepoints.G, HConfocal images of DAPI staining and immunofluorescence of human nuclear antigen and Ki67 of sagittal brain sections of immunodeficient mice engrafted with EPN‐ (G) and MB‐derived PDOs (H). Data information: The white square marks the region shown at higher magnification in (G″, H″). Scale bar 200 μm (D–F), 500 μm (G′–H′), 100 μm (G″–H″). Source data are available online for this figure.

**Figure EV1:**
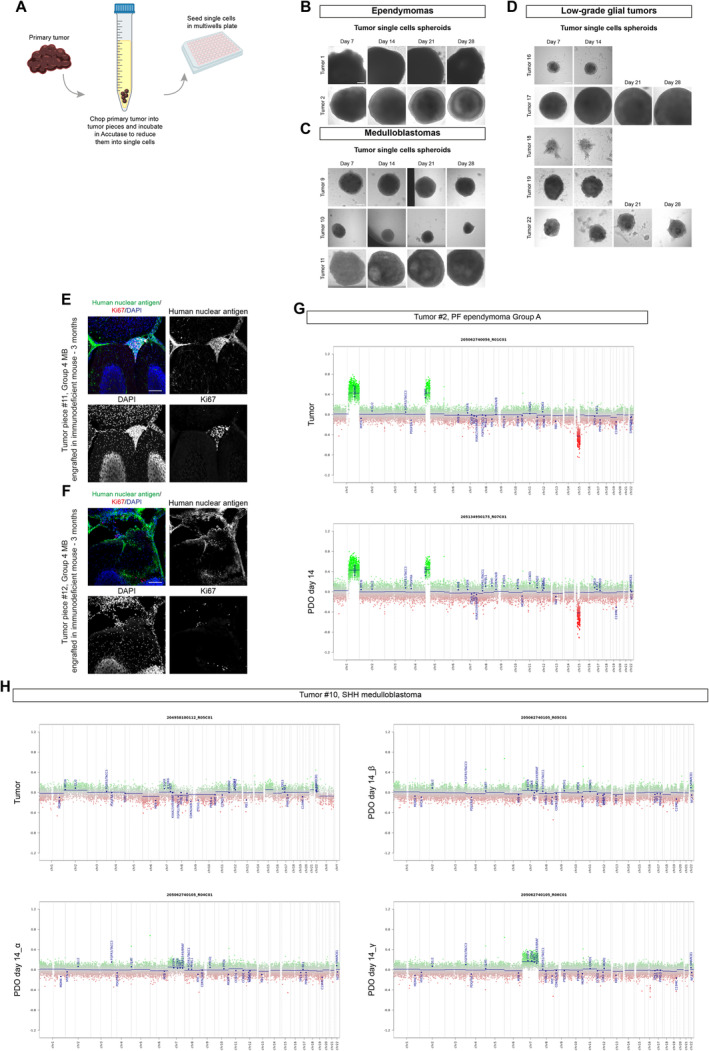
*In vitro* culture of patient‐derived organoids (PDOs) ASchematic representation of primary tumor samples management for generation of PDOs as tumor single cells spheroid.B–DBrightfield images of tumor single cells spheroids EPN‐ (B), MB‐ (C), and LGG‐ (D) derived PDOs at different timepoints.E, FConfocal images of DAPI staining and immunofluorescence of human nuclear antigen and Ki67 of sagittal brain sections of immunodeficient mice engrafted with MB‐derived PDOs.G, HCopy number variation profiles comparison between primary parental tumor and 3 different MB‐PDOs (H) and EPN‐PDOs (G). Data information: X axis: chromosomes; Y axis: Log_2_ copy number ratio. Scale bar 200 μm (B–D), 100 μm (E, F). DNA methylation (CNV) experiments (G, H) were performed once per primary tumor/matching PDOs.

We obtained fresh surgically resected tumoral tissues of a variety of pediatric brain tumors from patients referred to the Bambino Gesù Children's Hospital, Rome. Tumors were subdivided into three main groups: ependymomas (EPN), medulloblastomas (MB), and low‐grade glial (LGG) tumors (Table 1). Most of the tumors were located in the infratentorial compartment and displayed clinical characteristics typical of the three main tumor subtypes. In the case of EPN and MB, magnetic resonance images (MRI) showed solid tissues with signal features in accordance with high cellularity, high nucleus/cytoplasmic ratio, multinodular contrast enhancement. We also observed an increase in relative cerebral blood volume (rCBV) values in the perfusion study, reduction of the N‐acetyl aspartate (NAA) peak with an increase in the values of choline and peak in the lactate‐macromolecule region, under the study of spectroscopy. For LGG, images usually showed pathological tissues characterized by a hyper‐intense signal in T2, hypo‐intense in T1, by high diffusion values in ADC (apparent diffusion coefficient) maps, as signs of relatively low cellularity (Fig 1A). To find the best conditions for the generation of PDOs, we processed the tumor biopsies in two different ways (Fig 1B): resected tissues were either enzymatically dissociated to single cells for further reaggregation into spheroids (single cells spheroids, Fig EV1A) or cut into 0.5–2 mm diameter pieces using scalpels (tumor pieces, Fig 1C). Debris and red blood cells were removed (see Materials and Methods), and the processed samples were cultured in 96‐multiwell plates in a culture medium (PDOs medium) that we have previously developed for mouse Group 3 MB tumor spheroids (Ballabio *et al*, 2020). The size and the shape of tumor pieces seemed more consistent across all the different types of tumors compared to the single‐cell‐derived spheroids (Figs 1D–F and EV1B–D). Indeed using “tumor pieces” after dissection may avoid the clonal selection of specific cell populations in culture and the unnecessary stress to which the samples might undergo (Golebiewska *et al*, 2020; Jacob *et al*, 2020). Depending on the primary tumor type and the time frame by which it was received for further processing, tumor pieces formed round organoids (PDOs) within 1 week (Fig 1D–F). To assess whether PDOs still preserve the primary tumor properties *in vivo*, we engrafted PDOs into immunodeficient mice (Ballabio *et al*, 2020). A total of 2–3 PDOs were engrafted into 2–5 animals for 11 (1 EPN, 5 MB, and 5 LGG) of the 23 pediatric brain tumor samples received (Table 2). Four of 11 batches of injected PDOs exhibited successful engraftment and tumor development when examined 2–3 months after injection: 5/5 mice engrafted with tumor #2 (PF ependymoma, Group A), 2/2 mice engrafted with tumor #9 (SHH MB), 1/3 mice engrafted with tumor #11 (G4 MB), and 1/3 mice engrafted with tumor #12 (G4 MB) (Table 2). We confirmed the engraftment of PDOs‐derived cells by immunohistology of human nuclear antigen (Figs 1G and H, and EV1E and F). Of note, none of the mice engrafted with LGG‐derived PDOs showed signs of engraftments.

**Table 1:** List of primary pediatric brain tumor samples with information about location (IR: infratentorial, SR: supratentorial, MID: midbrain), patients (gender M: male, F: female; age mo: months, yo: years old), methods of processing (spheroid, piece), molecular alterations and days/passages at which PDOs were used for different analyses.

	Tumor	Location	Gender	Age	Spheroid	Piece	Molecular alterations	Analysis endpoint/Passages
EPENDYMOMAS								
1	PF ependymoma, Group A	IR	F	5 yo	✓	✓	1q gain; 6q loss	Day 61
2	PF ependymoma, Group A (relapse)	IR	F	4 yo	✓	✓	1q gain, 5p gain (*TERT*); 15q loss	Day 63
3	PF ependymoma, Group A	IR	M	3 yo		✓	/	p9 (Day 349)
4	PF ependymoma, Group A	IR	F	1 yo		✓	/	p2 (Day 228)
5	Sopratentorial ependymoma ZFTA‐RELA fusion	SR	M	6 mo		✓	/	Day 133
6	PF ependymoma, Group A (relapse)	IR	M	1 yo		✓	/	Day 99
7	PF ependymoma, Group A (relapse)	IR	M	4 yo		✓	/	Day 137
MEDULLOBLASTOMAS								
8	Group 4 medulloblastoma (relapse)	IR	M	11 yo		✓	/	Day 28
9	SHH medulloblastoma	IR	M	9 yo	✓	✓	Amplification: *MYCN*, *CCND2*; deletion: 9q (*PTCH1*), 10q (*PTEN* and *MGMT*) and 17p (*TP53*); mutation of *TP53*	Day 28
10	SHH medulloblastoma	IR	F	3 yo	✓	✓	Activating mutation of *SMO* in heterozygosity	Day 33
11	Group 4 medulloblastoma	IR	M	15 yo	✓	✓	Isochromosome 17	Day 28
12	Group 4 medulloblastoma	IR	M	7 yo		✓	/	Day 35
13	Group 4 medulloblastoma	IR	M	4 yo		✓	/	Day 28
14	Group 3 medulloblastoma	IR	F	15 yo		✓	*MYC* amplification	p21 (~2 years) and still ongoing
15	Group 3 medulloblastoma	IR	F	18 mo		✓	Chromosome 4, 8, 10, 11, 13, 15q, 16, 19, 20, 21 loss; 5, 18 gain	Day 61
LOW‐GRADE GLIAL TUMORS								
16	Low‐grade glioma with FGFR1‐TACC1 fusion	IR	M	4 yo	✓	✓	*FGFR1* (exon 17) – *TACC1* (exon 7) fusion	Day 28
17	Dysembryoplastic neuroepithelial tumor (relapse)	SR	F	9 yo	✓	✓	/	Day 28
18	Ganglioglioma (relapse)	MID	F	8 yo	✓	✓	*BRAF* mutation c.1799 T > A (p.V600E) *CDKN2A/B* deletion	Day 28
19	Pilocytic astrocytoma (relapse)	IR	F	8 yo	✓	✓	*KIAA1549* (exon 16) – *BRAF* (exon 9) fusion	Day 28
20	Pilocytic astrocytoma (relapse)	MID	M	7 yo		✓	*KIAA1549* (exon 16) – *BRAF* (exon 9) fusion	Day 28
21	Pilocytic astrocytoma (relapse)	IR	F	5 yo		✓	*KIAA1549* (exon 16) – *BRAF* (exon 9) fusion	Day 28
22	Pilocytic astrocytoma	IR	F	8 yo	✓	✓	*KIAA1549* (exon 16) – *BRAF* (exon 9) fusion	Day 28
23	Polymorphous low‐grade neuroepithelial tumor PLNTY	SR	F	8 yo		✓	Chromosome 3, 5, 7, 9, 12, 13, 15, 19, 20, 21 gain	Day 61

**Table 2:** Summary of PDOs engraftment in immunodeficient mice. Number of used PDOs and number of engrafted mice are reported, together with the number of mice displaying abnormal clusters and/or tumors.

	Tumor	# PDOs engrafted	# mice engrafted	Tumor/Neoplastic lesion
EPENDYMOMAS				
2	PF ependymoma, Group A (relapse)	2	5	5/5
MEDULLOBLASTOMAS				
9	SHH medulloblastoma	2	2	2/2
10	SHH medulloblastoma	2	3	0/3
11	Group 4 medulloblastoma	2	3	1/3
12	Group 4 medulloblastoma	2	3	1/3
13	Group 4 medulloblastoma	3	3	0/3
LOW‐GRADE GLIAL TUMORS				
18	Ganglioglioma (relapse)	2	2	0/2
19	Pilocytic astrocytoma (relapse)	3	2	0/2
20	Pilocytic astrocytoma (relapse)	2	3	0/3
21	Pilocytic astrocytoma (relapse)	2	2	0/2
22	Pilocytic astrocytoma	2	3	0/3

Therefore, we showed the possibility of putting in culture primary pediatric brain tumors establishing PDOs. Our findings suggest that the successful engraftment of PDOs may depend on the primary tumor type from which they are established, consistent with previous findings on the direct engraftment of pediatric brain tumors (Brabetz *et al*, 2018; Smith *et al*, 2020).

### DNA methylation and mutational profiling analysis of the PDOs

**Figure 2:**
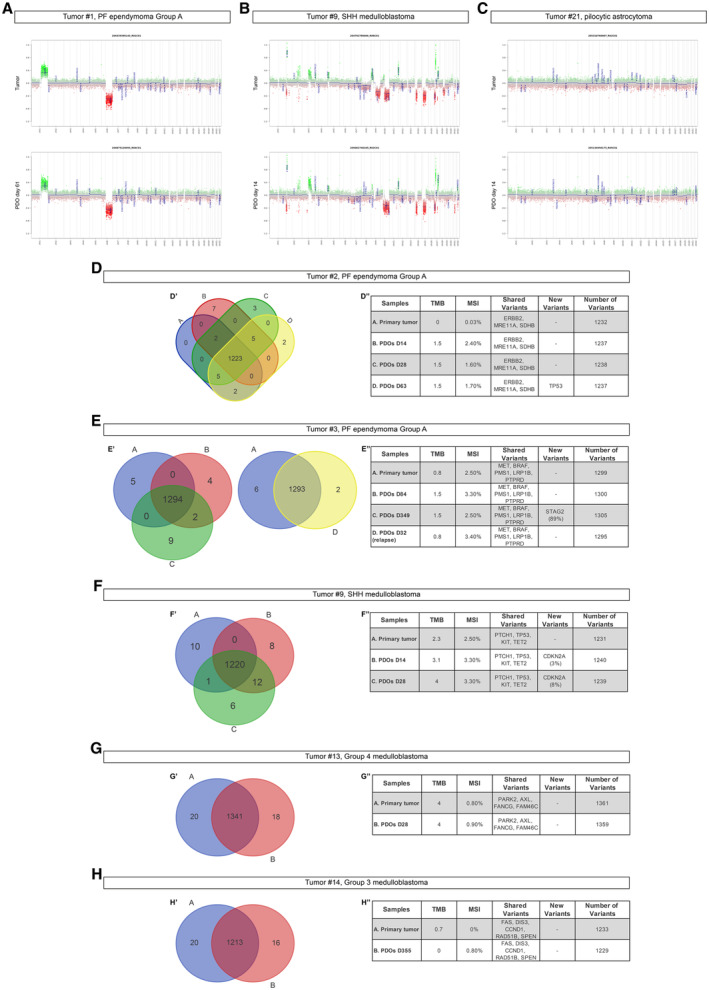
Maintenance of genomic and genetic profiles in PDOs A–CCopy number variation profiles comparison between primary parental tumor and EPN‐ (A), MB‐ (B), and LGG‐ (C) derived PDOs.D–HVenn diagram of primary tumor/PDOs at different timepoints (D'–H') and relevant shared or new variants (D''–H'') for EPN‐ (D, E) and MB‐ (F–H) derived PDOs. Data information: X axis: chromosomes; Y axis: Log2 copy number ratio. TMB, tumor mutational burden; MSI, microsatellite instability. DNA methylation (CNV) (A‐C) and TrueSight Oncology (D‐H) experiments were performed once per primary tumor/matching PDOs. Source data are available online for this figure.

To verify whether PDOs are *bona fide* replicates of their corresponding parental tumors, we analyzed the global DNA methylation and Copy Number Variation (CNV) profiles of all the organoids generated. Indeed, this recently developed approach (Capper *et al*, 2018) has been widely adopted and used for clinical‐decision making. The methylation data files from our organoids were run through the Heidelberg brain tumor classifier version v11b4 (www.molecularneuropathology.org) and they matched their original tumors (Table 3). CNV profiles showed the same genetic alterations (e.g., after 14 days in culture for tumor #9, SHH MB and tumor #21, pilocytic astrocytoma; after 61 days for tumor #1, PF EPN Group A, Figs 2A–C and EV1G and H). Interestingly, for a SHH MB‐PDO we also analyzed three different batches of organoids that showed similar CNV profiles (Fig EV1H).

**Table 3:** DNA methylation scores and methylation classes of human primary tumors and PDOs.

Tumor code	Tumor	Condition	DNA methylation score	Methylation class (v11b4)
1	PF ependymoma Group A	Tumor	0.99	Ependymoma, posterior fossa group A
		PDO day 61	0.95	Ependymoma, posterior fossa group A
2	PF ependymoma Group A (relapse)	Tumor	0.96	Ependymoma, posterior fossa group A
		PDO day 14_α	0.93	Ependymoma, posterior fossa group A
		PDO day 14_β	0.96	Ependymoma, posterior fossa group A
8	Group 4 medulloblastoma (relapse)	Tumor	0.99	Medulloblastoma, subclass group 4
		PDO day 22	0.2	Medulloblastoma, subclass group 4
9	SHH medulloblastoma	Tumor	0.96	Medulloblastoma, subclass SHH A
		PDO day 14	0.92	Medulloblastoma, subclass SHH A
10	SHH medulloblastoma	Tumor	0.99	Medulloblastoma, subclass SHH B
		PDO day 14_α	0.43	Medulloblastoma, subclass SHH B
		PDO day 14_β	0.90	Medulloblastoma, subclass SHH B
		PDO day 14_ γ	0.99	Medulloblastoma, subclass SHH B
11	Group 4 medulloblastoma	Tumor	0.97	Medulloblastoma, subclass group 4
		PDO day 14	0.88	Medulloblastoma, subclass group 4
12	Group 4 medulloblastoma	Tumor	0.99	Medulloblastoma, subclass group 4
		PDO day 14	0.99	Medulloblastoma, subclass group 4
13	Group 4 medulloblastoma	Tumor	0.99	Medulloblastoma, subclass group 4
		PDO day 14	0.99	Medulloblastoma, subclass group 4
16	Low‐grade glioma with FGFR1‐TACC1 fusion	Tumor	0.37	Low‐grade glioma, rosette‐forming glioneuronal tumor
		PDO day 14	0.30	Low‐grade glioma, subclass posterior fossa pilocytic astrocytoma
17	Dysembryoplastic neuroepithelial tumor (relapse)	Tumor	0.95	Low‐grade glioma, dysembryoplastic neuroepithelial tumor
		PDO day 14	0.96	Low‐grade glioma, dysembryoplastic neuroepithelial tumor
18	Ganglioglioma (relapse)	Tumor	0.84	(anaplastic) pleomorphic xanthoastrocytoma
		PDO day 14	0.75	(anaplastic) pleomorphic xanthoastrocytoma
19	Pilocytic astrocytoma (relapse)	Tumor	0.87	Low‐grade glioma, subclass posterior fossa pilocytic astrocytoma
		PDO day 14	Insufficient material	Insufficient material
20	Pilocytic astrocytoma (relapse)	Tumor	0.70	Low‐grade glioma, subclass posterior fossa pilocytic astrocytoma
		PDO day 14	0.53	Low‐grade glioma, subclass posterior fossa pilocytic astrocytoma
21	Pilocytic astrocytoma (relapse)	Tumor	0.95	Low‐grade glioma, subclass posterior fossa pilocytic astrocytoma
		PDO day 14	0.66	Low‐grade glioma, subclass posterior fossa pilocytic astrocytoma
22	Pilocytic astrocytoma	Tumor	0.99	Low‐grade glioma, subclass posterior fossa pilocytic astrocytoma
		PDO day 14	0.18	Low‐grade glioma, subclass posterior fossa pilocytic astrocytoma

**Figure EV2:**
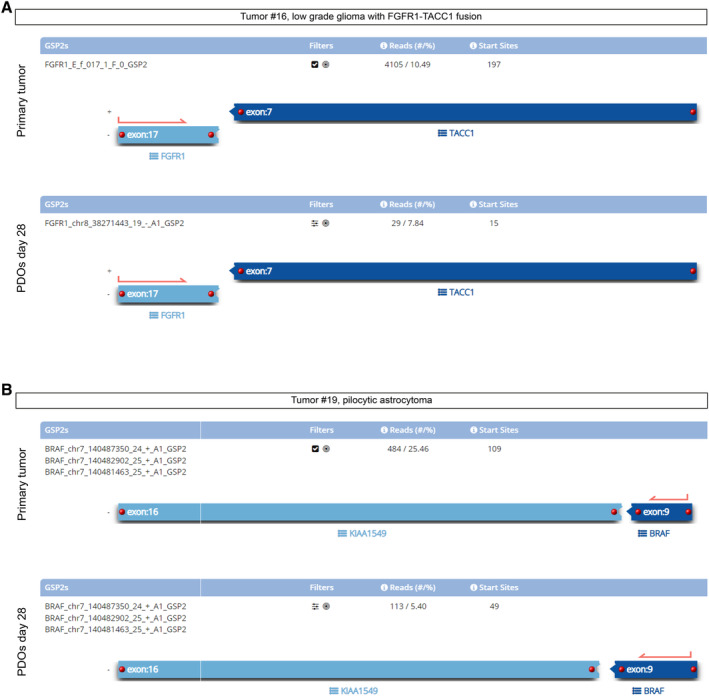
Data from RNA‐based assay showed maintenance of genomic features of fusion‐positive tumors and their corresponding PDOs Tumor #16, LGG with *FGFR1‐TACC1* fusion compared to derived PDOs at day 28: the assay detects the same fusion.Tumor #19, *KIAA1549‐BRAF* fusion was detected in pilocytic astrocytoma and in derived PDOs at day 28. Both PDOs shared the same breakpoints compared to primary parental tumors. Data information: Experiments were performed once per primary tumor/matching PDOs.

To further support the conservation of mutational profiles between PDOs and parental tumors, we analyzed samples through a comprehensive genomic profiling (TruSight Oncology 500 Library Preparation Kit, Illumina, San Diego, CA). PDOs showed a comparable number of detectable gene variants, sharing the same pathogenetic variants, a quite similar tumor mutational burden (TMB) and microsatellite instability (MSI) with respect to parental tumors (Fig 2D and E tumor #2 and #3 PF EPN Group A; Fig 2F, tumor #9 SHH MB; Fig 2G, tumor #13 G4 MB; Fig 2H, tumor #14 G3 MB). The appearance of new pathogenetic variants was a rare event occurring only after long time in culture (Fig 2D″, PDOs days 63 for tumor #2; Fig 2E″ PDOs day 349 ‐ p9 for tumor #3). Interestingly for tumor #14 (Fig 2H″), new variants were not observed even after seven passages (355 days) in culture. LGG‐derived PDOs also shared the same gene fusions and mutations, compared to parental tumors (Fig EV2A and B, Table 4).

**Table 4:** Mutation and fusions maintenance in primary tumors and PDOs.

Tumor code	Tumor	Condition	Gene alterations	Breakpoints in HG19 coordinates
18	Ganglioglioma (relapse)	Tumor	*BRAF*: c.1799 T > A; p.V600E	
		PDO day 28	*BRAF*: c.1799 T > A; p.V600E	
16	Low‐grade glioma with FGFR1‐TACC1 fusion	Tumor	*FGFR1 → TACC1*	chr8:38271436, chr8:38693680
		PDO day 28	*FGFR1 → TACC1*	chr8:38271436, chr8:38693680
19	Pilocytic astrocytoma (relapse)	Tumor	*KIAA1549 → BRAF*	chr7:138545885, chr7:140487384
		PDO day 28	*KIAA1549 → BRAF*	chr7:138545885, chr7:140487384

### Immunohistochemical analysis of the PDOs

**Figure 3:**
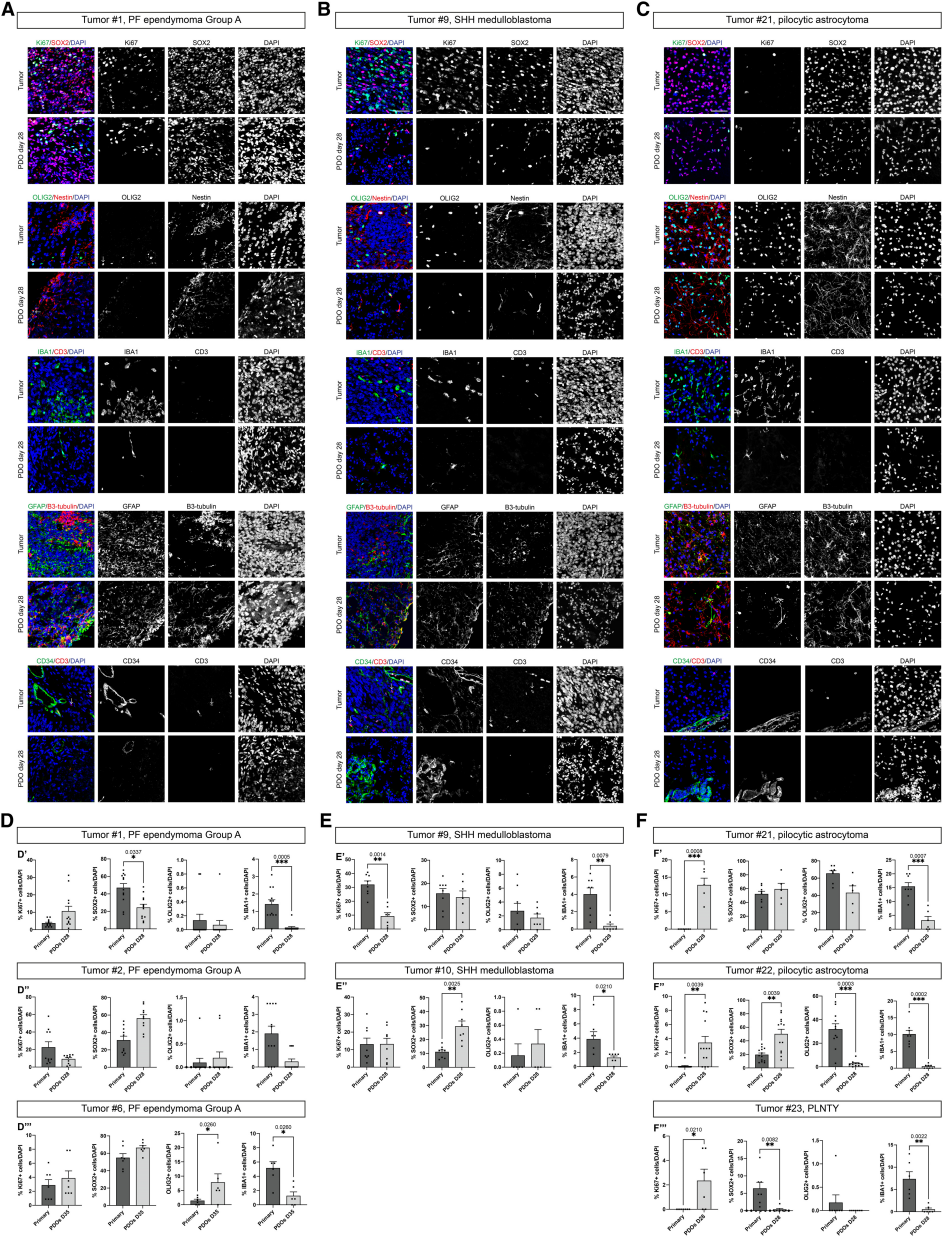
Maintenance of cellular heterogeneity in PDOs A–CConfocal images of immunofluorescence of Ki67, SOX2, OLIG2, Nestin, IBA1, CD3, GFAP, B3‐tubulin, CD34 of EPN‐ (A), MB‐ (B) and LGG‐ (C) derived PDOs.D–FQuantification in EPN‐ (D), MB‐ (E), and LGG‐ (F) derived PDOs of Ki67^+^, SOX2^+^, OLIG2^+^, and IBA1^+^. Cells are shown as percentage of specific marker^+^ cells/DAPI. Data information: Data are presented as mean ± s.e.m.; each dot represents a ROI/image. For each marker, *n* = 5–12 ROI/image of primary tumor was considered. For each marker, *n* = 2–3 PDOs (biological replicates) were considered; for each PDO, *n* = 3–4 ROI/image was used. Quantification experiments were performed once per primary tumor/matching PDOs. Kolmogorov–Smirnov test for data with non‐normal distribution; ***P ≤ 0.001, **P ≤ 0.01, *P ≤ 0.05 (D–F). Exact *P* values are reported in figure. The white arrows highlight specific cells. Scale bar 50 μm (A–C). Source data are available online for this figure.

**Figure EV3:**
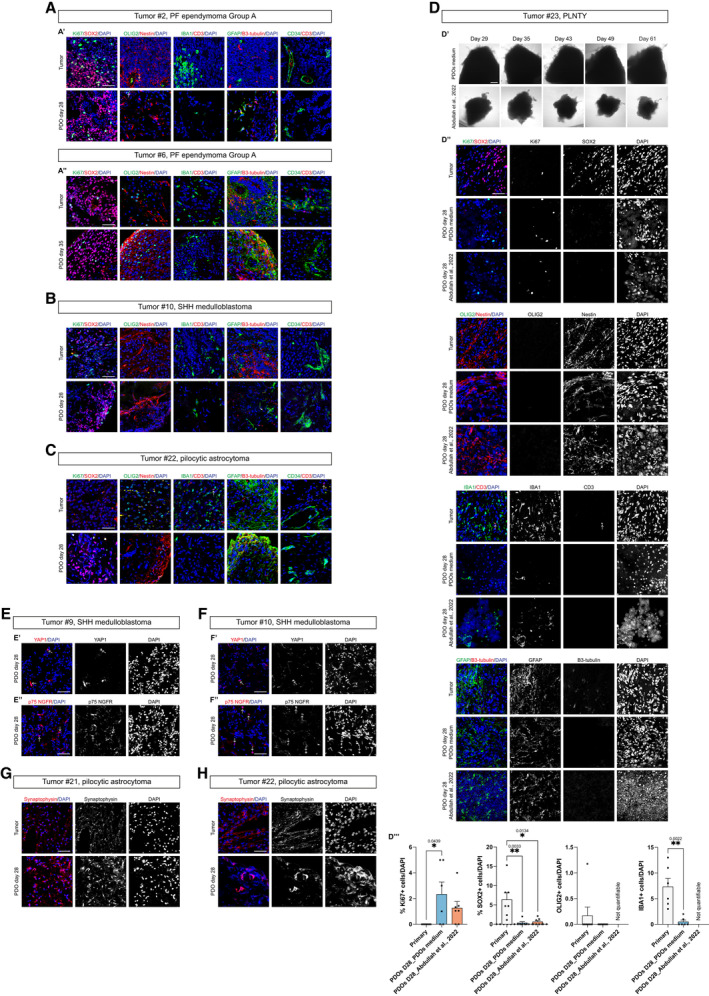
Maintenance of cellular heterogeneity in PDOs and comparison with already published medium A–CConfocal images of immunofluorescence of Ki67, SOX2, OLIG2, Nestin, IBA1, CD3, GFAP, B3‐tubulin, CD34 of EPN‐ (A′–A″), MB‐ (B) and LGG‐ (C) derived PDOs.DBrightfield images of LGG‐derived PDOs as tumor pieces at different timepoints in PDOs medium and cultured according to (Abdullah *et al*, 2022) (D′), confocal images of immunofluorescence of Ki67, SOX2, OLIG2, Nestin, IBA1, CD3, GFAP, B3‐tubulin (D″) and quantification in PDOs of Ki67^+^, SOX2^+^, OLIG2^+^ and IBA1^+^ cells (D″′).E, FConfocal images of immunofluorescence of YAP1 (E′, F′) and p75 NGFR (E″, F″) of MB‐derived PDOs.G, HConfocal images of immunofluorescence of synaptophysin of LGG‐derived PDOs. Quantifications are shown as percentage of specific marker^+^ cells/DAPI (D″′). Data information: Data are presented as mean ± s.e.m.; each dot represents a ROI/image. For each marker, *n* = 5–7 ROI/image of primary tumor was considered. For each marker, *n* = 2–3 PDOs (biological replicates) were considered; for each PDO, *n* = 3–4 ROI/image was used. Quantification experiments were performed once per primary tumor/matching PDOs. Kruskal–Wallis test with Dunn's *post hoc* correction; **P ≤ 0.01, *P ≤ 0.05. Adjusted and exact *P* values are reported in figure. The white arrows highlight specific cells. Scale bar 50 μm (A–C, D″, E–H), 200 μm (D′).

To further characterize cellular identities, we examined through immunohistochemical analyses a wide panel of neurodevelopmental markers (Figs 3A–C and EV3A–D), including proliferation (Ki67) and neural progenitor markers (SOX2, OLIG2, and Nestin), glial and neuronal differentiation markers (GFAP and B3‐tubulin), immune system microenvironment components (IBA1 and CD3), and endothelial marker (CD34). Considering PDOs for two or three tumors of each tumor subgroup (EPN, MB, and LGG), after 28 and 35 days of culture we observed no significant differences in Nestin, GFAP, and B3‐tubulin ratio between PDOs and parental tumors (Figs 3A–C and EV3A–D). We observed a slight significant difference for SOX2 between primary parental tumor and PDOs in four tumors (tumor #1, tumor #10, tumor #22, and tumor #23. Figs 3D′, E″, F″, and F″′). We found an increase, although not significant, in Ki67^+^ cells content of PDOs from tumor #1 and #6 (PF ependymoma Group A, Fig 3D′ and D″′), while this increase was statistically significant in PDOs from tumor #21, tumor #22 (pilocytic astrocytomas) and tumor #23 (polymorphous low‐grade neuroepithelial tumor of the young—PLNTY) compared to the parental tumor, most likely due to the culture conditions (Fig 3F). We detected a slight decrease of Ki67^+^ cells in PDOs from tumor #2 (PF ependymoma Group A) and tumor #9 (SHH MB) (Fig 3D″ and E′). In line with the parental tumors, OLIG2 was present and absent in the PDOs derived from the pilocytic astrocytoma and from the ependymoma, respectively (Fig 3D′, D″, and F′). However, for PDOs from one pilocytic astrocytoma (tumor #22) we detected a significant decrease in OLIG2^+^ cells (Fig 3F″) and for one from PF EPN (tumor #6) we observed an increase in this cell population (Fig 3D″′). IBA1^+^ microglia cells (Fig 3D–F) as well as CD34^+^ endothelial cells (Figs 3A–C and EV3A–C) showed a significant decrease in PDOs compared to the parental tumors in all three different subgroups. This change can most likely be attributed to the culture conditions that are not specifically meant to preserve these types of cellular populations. We also examined more tumor‐related markers in PDOs at day 28, such as YAP1 and p75 NGFR for SHH MB and synaptophysin for pilocytic astrocytoma (Fig EV3E–H). In detail, we observed the presence of YAP1^+^ (Fig EV3E′ and F′) and p75 NGFR^+^ (Fig EV3E″ and F″) cells in SHH MB‐derived PDOs (tumor #9 and #10). We also observed no significant difference in the presence of synaptophysin ratio between pilocytic astrocytoma‐derived PDOs and their parental tumors (tumor #21 and tumor #22, Fig EV3G and H).

**Figure EV4:**
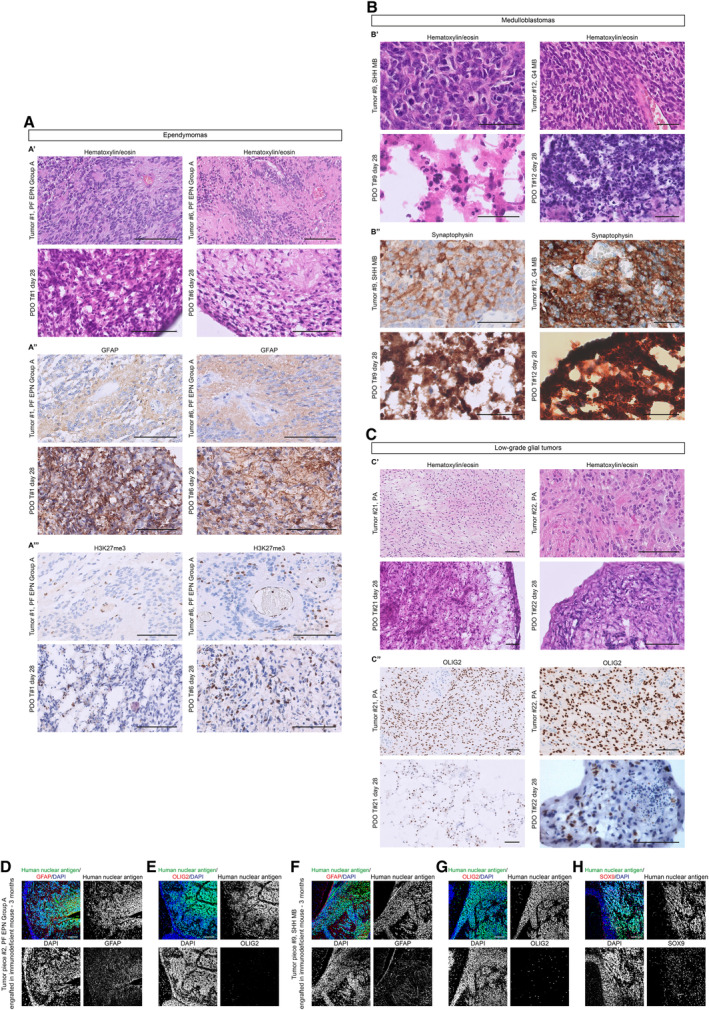
Maintenance of morphological features and cellular heterogeneity in PDOs and PDOs‐derived tumors A–CMorphological features (A′, B′, C′) and immunohistochemical expression of lineage markers GFAP (A″), H3K27me3 (A″′), synaptophysin (B″), and OLIG2 (C″) of 2 EPN, 2 MB and 2 LGG paired parental tumors/PDOs samples.D, FConfocal images of immunofluorescence of human nuclear antigen and GFAP of sagittal brain sections of immunodeficient mice engrafted with EPN‐ (D) and MB‐ (F) derived PDOs.E, GConfocal images of immunofluorescence of human nuclear antigen and OLIG2 of sagittal brain sections of immunodeficient mice engrafted with EPN‐ (E) and MB‐ (G) derived PDOs.HConfocal images of immunofluorescence of human nuclear antigen and SOX9 of sagittal brain sections of immunodeficient mice engrafted with MB‐derived PDOs. Data information: Scale bar 100 μm (A, C, D–H), 50 μm (B).

PDOs were also analyzed for their cellular morphology and architecture through hematoxylin and eosin (H&E) staining and for the immunohistochemical expression of markers currently utilized in the diagnostic setting of the brain tumors (Fig EV4A–C). As for tumor #1, the degree of cellularity of the PDOs was comparable to the parental tumor, and, although clear‐cut well‐formed rosettes and pseudorosettes were not definitively present, the cells tended to arrange in structures vaguely reminiscent of true rosettes (Fig EV4A′). Furthermore, GFAP expression was preserved (Fig EV4A″) as well as the loss of expression of H3K27me3 (Fig EV4A″′), which, in the context of a PF ependymoma, is currently used in the pathology practice as a reliable surrogate marker for the molecular subgroup A. In the PDOs of tumor #9 (SHH MB) and PDOs of tumor #12 (G4 MB), coherently with the parental anaplastic medulloblastoma, mitoses, and apoptosis were easily identified, and the expression of synaptophysin was preserved (Fig EV4B). As for tumor #21 and #22, the cells of the PDOs maintained the typical piloid morphology as well as the expression of OLIG2 (Fig EV4C). In general, PDOs maintained the cellular populations present in the parental tumors, even if changes in tumor microenvironment cells were observed. We performed similar immunohistochemical analyses on the tumors generated upon PDOs engraftment into immunodeficient mice. Tumors were positive for the proliferation marker Ki67 (Figs 1G and H, and EV1E and F) and maintained cellular populations characteristic of the parental tumors. In detail, tumor #2 (PF ependymoma Group A) still presented GFAP^+^ glial cells and was negative for OLIG2 progenitor cells marker (Fig EV4D and E); tumor #9 (SHH MB) was negative for GFAP (the GFAP^+^ cells were interpreted as resident mouse astrocytes since those are human nuclear antigen‐), positive for OLIG2 and presented also SOX9 positive glial precursors cells (Vong *et al*, 2015; Sun *et al*, 2017) (Fig EV4F–H). Lastly, we can conclude that our PDOs maintain several features of the original tumors, even if from the histological analysis PDOs incompletely reproduce the original tumor.

To verify that our *in vitro* culture conditions were the best for the establishment and long‐term maintenance of our PDOs, we tested a different published culture condition (Abdullah *et al*, 2022). In particular, we tested the method used for adult LGG PDOs (Abdullah *et al*, 2022) to generate and maintain pediatric LGG‐derived PDOs (tumor #23) (Fig EV3D). We observed that PDOs could be maintained *in vitro* culture in both conditions (Fig EV3D′). When comparing PDOs grown in the two conditions through immunohistochemical analysis (Fig EV3D″), we did not observe a significant difference in Ki67^+^ cells and SOX2^+^ cells (Fig EV3D″′), but we observed a significant difference in the nuclear morphology of PDOs. Indeed, PDOs grown in Long Term Glioma medium and hypoxic conditions displayed very few defined DAPI^+^ nuclei, surrounded by degraded or dead materials (Fig EV3D″). This might also be the reason why we could not quantify at a statistical level the difference in OLIG2^+^ and IBA1^+^ cells in PDOs grown in this condition compared to ours (Fig EV3D″′). These results suggest that the PDOs derived from pediatric LGG tumors can grow in an optimal manner in the culture conditions we have established.

### Maintenance of cell‐type heterogeneity and molecular signature in Group 3 MB‐ and EPN‐derived PDOs

To compare the transcriptomic profiles and cell type compositions between human tumors and PDOs, we profiled patient tumors (tumor #2, PF ependymoma Group A; tumor #15, Group 3 MB) along with two matching PDOs collected at different time points (Table 5).

**Table 5:** Detailed information about single‐cell RNA sequencing analysis.

Dataset	Sample_ID	Sample_type	PDO_timepoint	Tumor type	Estimated Number of Cells	Mean Reads per Cell	Median Genes per Cell	Number of cells (after QC/filtering)	Mean Reads per Cell (after QC/filtering)	Median Genes per Cell (after QC/filtering)
PDO2	Primary tumor #2	Primary tumor	–	PFA EPN	16,946	12,634	778	10,845	14,737.8	4,279.7
PDO2	PDO Day 14	PDO	Day 14	PFA EPN	4,777	51,608	4,345	3,437	7,152.2	2,685.8
PDO2	PDO Day 28	PDO	Day 28	PFA EPN	11,130	20,760	2,486	9,071	4,335.6	1,634.6
PDO15	Primary tumor #15	Primary tumor	–	G3 MB	2,638	63,542	684	1,204	4,679.7	1,823.3
PDO15	PDO Day 28	PDO	Day 28	G3 MB	2,098	78,854	506	688	6,225	2,287.7
PDO15	PDO Day 61	PDO	Day 61	G3 MB	1,450	117,784	2,082	941	7,166.2	2,668.7

**Figure 4:**
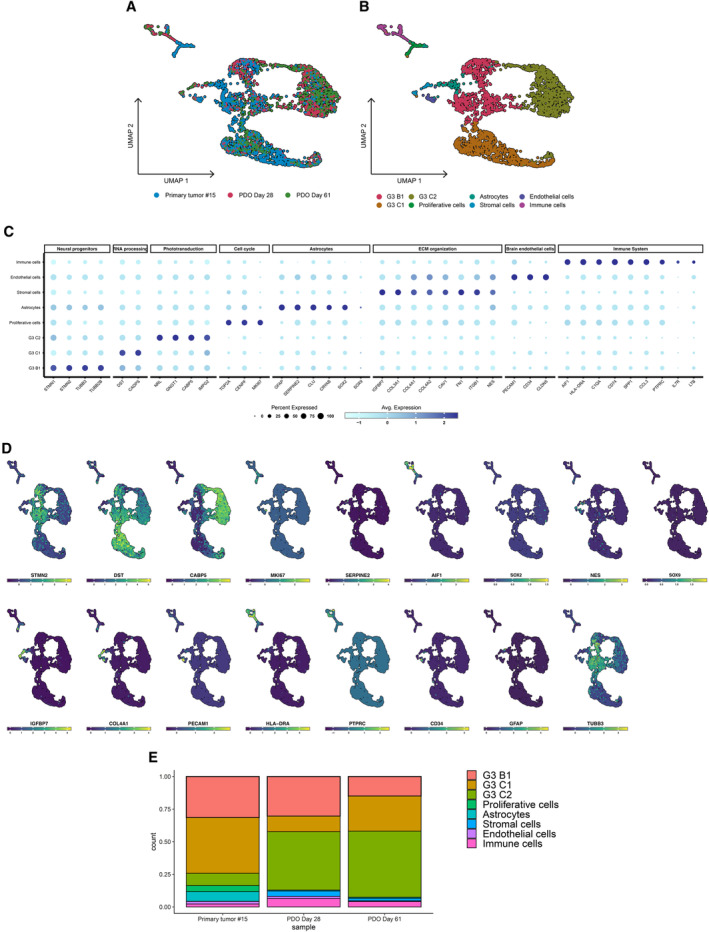
scRNA‐seq data analysis of primary tumor #15 and matching PDOs samples describes G3 MB‐specific intratumoral heterogeneity, recapitulated in the PDOs model UMAP dimensionality reduction plot showing the cluster distribution of cells obtained from tumor and PDOs samples.UMAP plot showing the different independent clusters obtained by integrating the malignant cells from “Primary tumor #15,” “PDO Day 28,” and “PDO Day 61” datasets.Expression dotplot representing the key markers identified for each cluster and belonging to cellular and/or functional categories.FeaturePlot showing the expression levels of key markers in each cell.Stacked barplot representing the relative proportion (expressed in %) of the tumor and PDO cells across the different subclusters. Data information: scRNA‐seq experiment was performed once per primary tumor/matching PDOs. Source data are available online for this figure.

**Figure EV5:**
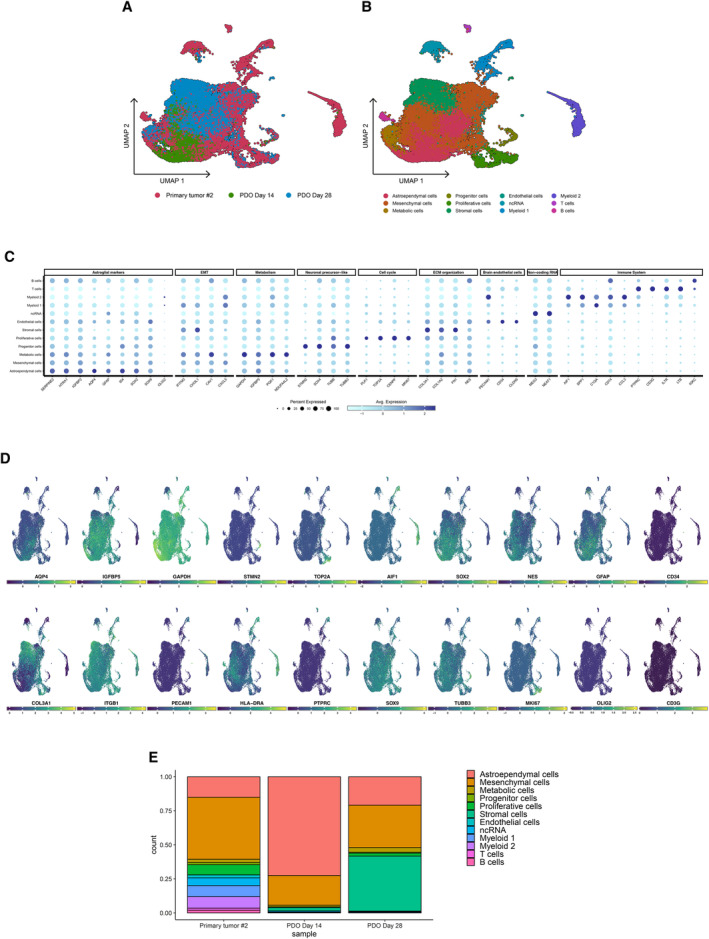
scRNA‐seq data analysis of primary tumor #2 and matching PDOs samples describes EPN PFA‐specific intratumoral heterogeneity, recapitulated in the PDOs model UMAP dimensionality reduction plot showing the cluster distribution of cells obtained from tumor and PDO samples.UMAP plot showing the different independent clusters obtained by integrating the malignant cells from “Primary tumor #2,” “PDO Day 14,” and “PDO Day 28” datasets.Expression dotplot representing the key markers identified for each cluster and belonging to cellular and/or functional categories.FeaturePlot showing the expression levels of key markers in each cell.Stacked barplot representing the relative proportion (expressed in %) of the tumor and PDO cells across the different subclusters. Data information: scRNA‐seq experiment was performed once per primary tumor/matching PDOs.

We profiled 32,853 and 6,186 cells in total in PDO2 and PDO15 sets, respectively; we selected 23,353 and 2,833 cells from the same datasets following quality control and filtering steps for downstream analysis. Next, we aimed at identifying the clusters including malignant and non‐malignant cell types; for this reason, we integrated the cells coming from the three conditions in merged datasets. Unsupervised clustering of this set indicated the formation of independent subclusters which heterogeneously included cells from both human tumor and PDO samples (Figs 4A and EV5A). The identified clusters presented robust and unique gene signatures (Figs 4B and EV5B); for each cell cluster, we observed a set of differentially expressed genes that define different cell types or functional categories commonly enriched in solid malignancies and also specifically in medulloblastoma (Riemondy *et al*, 2022) and ependymoma (Gillen *et al*, 2020; Gojo *et al*, 2020) tumors (Datasets EV1 and EV2).

In PDO15 dataset, tumor cells highly recapitulated the cell populations described by Riemondy *et al* (2022) in Group 3 medulloblastoma samples: B1 (enriched with neural progenitor markers), C1 (characterized by high levels of DST and CADPS, involved in the RNA processing) and C2 (showing high levels of factors involved in phototransduction mechanisms, such as GNGT1, CABP5, and IMPG2) (Fig 4C and D). Additional cell types included astrocytes (SERPINE2, CLU, CRYAB), proliferative cells (Ki67, TOP2A, CENPF), stromal cells, characterized by the expression of markers involved in the organization of the extracellular matrix (ECM) (COL3A1, COL1A2, ITGB1, FN1, Nestin), endothelial cells (PECAM1, CD34, CLDN5), immune cells (Fig 4C and D).

In tumor #2 and relative PDOs, we found a cluster particularly enriched with astroglial/ependymal markers (AQP4, GFAP, ID4, SERPINE2, HTRA1, IGFBP2) linked with ependymoma and brain tumorigenic progression (Chen *et al*, 2018; Yang *et al*, 2018; Khan, 2019), and another with a strong link with mesenchymal markers, similar as previously observed (Gillen *et al*, 2020) (CHI3L1, CAV1, IGFBP5) as predominant. Other clusters of cells with tumor‐associated functions included: undifferentiated/progenitor cells with “neuronal stem‐like” gene signature (STMN2/4, SOX2, TUBB/TUBB3, PAX3/6‐positive); cells with prominent metabolic functions (GAPDH, VEGFA, IGFBP5, NDUFA4L2); cells with an active proliferative phenotype (PLK1, Ki67, TOP2A, CENPF) (Fig EV5C and D). Additional cell types detected in this dataset included stromal, endothelial, and immune cells, from both myeloid (IBA1, SPP1, C1QA) and lymphoid (CD45, CD3, IL7R) compartments. Lastly, a group of cells, emerged as specifically expressing non‐coding RNAs with a tumor‐associated role, such as MEG3, NEAT1, and FTX (Zhang *et al*, 2017; Qin *et al*, 2020; Katsushima *et al*, 2021) (Fig EV5C and D).

Taken together, our transcriptomic data suggested a high cellular and functional intratumoral heterogeneity in G3 MB and PF EPN Group A tumors, respectively, in line with previous studies (Gillen *et al*, 2020; Gojo *et al*, 2020; Riemondy *et al*, 2022). Differentially expressed genes were found upregulated in PDOs compared to their original tumors encoded for ECM components (COL1A1, COL3A1, COL4A1, LUM, FN1). To define the cellular composition of the PDOs samples compared to the original tumor, we observed the distribution of all the identified clusters across the three conditions (primary tumor; PDOs early stage; PDOs late stage). All the identified clusters, in both datasets, include cells coming from both human tumor and PDOs samples, suggesting the capacity of the PDOs models to recapitulate the transcriptomic and cellular landscape of the matching human tumor (Figs 4E and EV5E, Table 6). In PDO15 datasets, the clusters representing Ki67^+^ proliferative, CD34^+^ endothelial, and IBA1^+^/CD3^+^ immune cells have been observed as impaired in PDO samples in comparison with the patient tumor (Fig EV5E, Table 6), confirming the loss of these cell types observed in PDOs of tumor #2 by immunofluorescence (Figs 3D″ and EV3A′).

**Table 6:** Proportion (%) of cells forming each cluster, divided by condition (tumor or PDOs), for PDO2 and PDO15 datasets.

	Primary tumor #2	PDO Day 14	PDO Day 28
Astroependymal cells	15.1	72.5	20.9
Mesenchymal cells	45.5	21.8	31.2
Metabolic cells	2.5	1.2	3.4
Progenitor cells	1.6	0.5	0.6
Proliferative cells	7.4	0.1	2.2
Stromal cells	0.1	2.4	40.3
Endothelial cells	2.1	0.0	0.3
ncRNA	5.7	0.8	0.4
Myeloid 1	8.1	0.3	0.6
Myeloid 2	8.4	0.3	0.0
T cells	1.5	0.0	0.1
B cells	2.0	0.1	0.0

	Primary tumor #15	PDO Day 28	PDO Day 61
G3 B1	31.5	30.4	15.0
G3 C1	42.7	11.9	26.9
G3 C2	9.5	44.8	50.7
Proliferative cells	4.7	0.0	0.1
Astrocytes	7.4	0.7	0.7
Stromal cells	0.1	4.1	1.9
Endothelial cells	2.0	1.6	0.5
Immune cells	2.2	6.5	4.1

Overall, our findings indicate that the cellular and functional heterogeneity described in the primary tumors was observed in the matching PDOs samples.

### Culture of patient‐derived xenograft organoids (PDXOs) from PDX tumor

**Figure 5:**
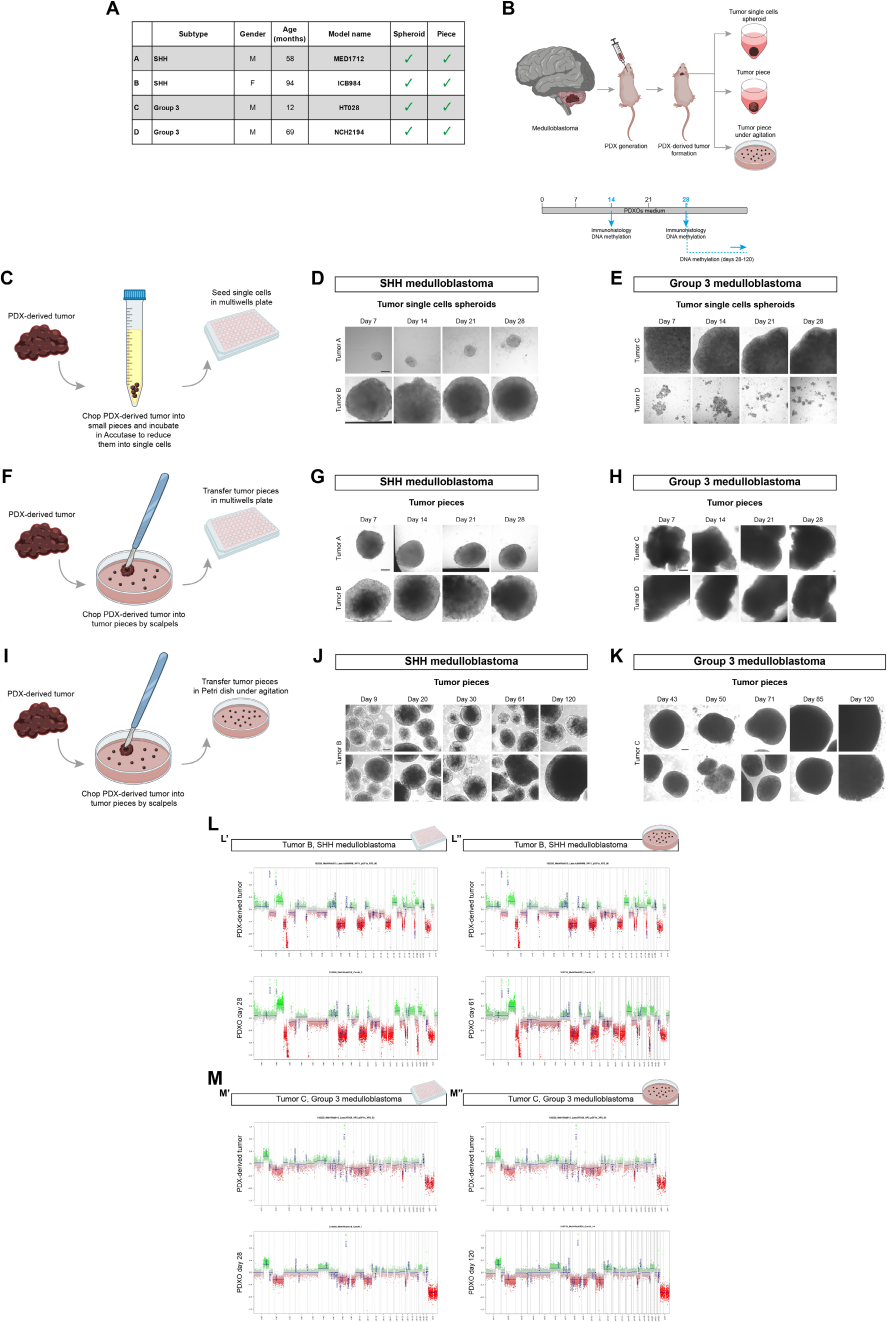
*In vitro* culture of patient‐derived xenograft organoids (PDXOs) and maintenance of genomic aberrations AList of MB PDX‐derived tumor samples with information about MB subtypes, patients (gender M: male, F: female; age in months), model name, and methods of processing (spheroid, piece).BSchematic representation of PDX‐derived tumor samples management workflow.CSchematic representation of PDX‐derived tumor samples management for generation of PDXOs as tumor single cells spheroid.D, EBrightfield images of tumor single cells spheroid PDXOs from SHH and G3 MB‐derived PDXs at different timepoints.FSchematic representation of PDX‐derived tumor samples management for generation of PDXOs as tumor piece.G, HBrightfield images of tumor piece PDXOs from SHH and G3 MB‐derived PDXs at different timepoints.ISchematic representation of PDX‐derived tumor samples management for generation of PDXOs as tumor piece in suspension.J, KBrightfield images of tumor piece PDXOs in suspension from SHH and G3 MB‐derived PDXs at different timepoints.L, MCopy number variation profiles comparison between PDX‐derived tumor and PDXOs from SHH (L) and G3 MB‐derived PDXs (M). CNVs profiles of PDXOs as tumor piece kept in multiwell are shown in (L′–M′), kept in suspension are shown in (L″–M″). Data information: X axis: chromosomes; Y axis: Log2 copy number ratio. Scale bar 200 μm in (D, E, G, H, J, K). DNA methylation experiments (CNV) (L, M) were performed once per primary tumor/matching PDXOs. Source data are available online for this figure.

Once established this strategy with samples deriving from primary tumors, we sought to verify whether it could be applied to tumors deriving from patient‐derived xenografts (PDXs), to create patient‐derived xenograft organoids (PDXOs). Indeed, large PDX‐derived tumor biobanks are already available for *in vivo* testing (Brabetz *et al*, 2018; Smith *et al*, 2020). However, these *in vivo* tests have their limitations regarding the number of drugs that can be tested. Organoids grown from PDX models could be used for *in vitro* drug screening to prioritize the drug combinations to test *in vivo* on the corresponding PDX models. We applied the same approaches to four samples from MB PDXs obtained from the Hopp Children's Cancer Center in Heidelberg (Fig 5A). Samples were either enzymatically dissociated to single cells for further reaggregation into spheroids (single cells spheroids) or cut into 0.5–2 mm diameter pieces using scalpels (tumor pieces, Fig 5B). As for PDOs formation, the enzymatic dissociation, and further single cells reaggregation did not work for all four samples (Fig 5C–E). When PDX‐derived tumors were cut into 0.5–2 mm diameter pieces, they grew in the *in vitro* culture and were more consistent across all the different types of tumors compared to the single‐cell‐derived spheroids (Fig 5F–H). The best PDXOs were the ones established through the culture of 0.5–2 mm cut tumor pieces in suspension under agitation (Fig 5I–K). PDXOs displayed significant growth and could be kept in culture up to 4 months. PDXOs grown in pieces in 96‐multiwell plates (Fig 5L′ and M′) and in suspension (Fig 5L″ and M″) were evaluated through DNA methylation analysis (classifier version v11b6) and displayed similar CNV profiles (Fig 5L and M) and DNA methylation profiles to the parental PDX‐derived tumors (Table 7). PDXOs were also checked for the maintenance of a proliferative state during time through Ki67 immunohistology and we validated that they were derived from the human samples (used for the PDX generation) thanks to immunohistology for human nuclear antigen (Appendix Fig S1A–D).

**Table 7:** DNA methylation scores and methylation classes of PDX tumors and PDXOs.

Tumor code	Model name	Subtype	Condition	DNA methylation score	Methylation class (v11b6)	Subtype score	Subtype
B	ICB984	SHH MB	PDX tumor	0.87	Medulloblastoma, subclass SHH CHL AD	0.80	MB SHH 3
			PDXOs day 28 – multiwell	0.75	Medulloblastoma, subclass SHH CHL AD	0.60	MB SHH 3
			PDXOs day 28 – suspension	0.61	Medulloblastoma, subclass SHH CHL AD	0.44	MB SHH 3
			PDXOs day 61 – suspension	0.59	Medulloblastoma, subclass SHH CHL AD	0.15	MB SHH 4
C	HT028	Group 3 MB	PDX tumor	1	Medulloblastoma, subclass group 3	1	MB G34 II
			PDXOs day 28 – multiwell	1	Medulloblastoma, subclass group 3	1	MB G34 II
			PDXOs day 28 – suspension	1	Medulloblastoma, subclass group 3	1	MB G34 II
			PDXOs day 50 – suspension	1	Medulloblastoma, subclass group 3	1	MB G34 II
			PDXOs day 120 – suspension	0.99	Medulloblastoma, subclass group 3	0.98	MB G34 II
D	NCH2194	Group 3 MB	PDX tumor	1	Medulloblastoma, subclass group 3	0.99	MB G34 III
			PDXOs day 14 – multiwell	1	Medulloblastoma, subclass group 3	1	MB G34 III
			PDXOs day 28 – multiwell	1	Medulloblastoma, subclass group 3	1	MB G34 III

In summary, we derived PDXOs from PDX‐derived tumors and showed that they retain the genomic signature of the parental tumors even after 4 months of *in vitro* culture.

Also in this case, we verified that our *in vitro* culture conditions were the best for the establishment and long‐term maintenance of our types of PDXOs by culturing them using already established protocol (Jacob *et al*, 2020). In particular, we tested the culture conditions for the maintenance of glioblastoma organoids (GBOs) (Jacob *et al*, 2020) for the *in vitro* culture of G3 MB‐ and SHH‐PDXOs, since their more aggressive traits make them closer to glioblastoma tumor. We tried both to use GBOs medium from the very first establishment of SHH‐PDXOs from the PDX‐parental tumors (Appendix Fig S1E) and also to switch the culture conditions of already established G3 MB‐PDXOs (Appendix Fig S1F and G), maintaining a corresponding counterpart growing in our PDOs/PDXOs medium. However, PDXOs could not be maintained and did not grow in GBOs medium, as they display signs of stress (e.g., loss of cells, disaggregation) when cultured in this medium (Appendix Fig S1E–G). These results suggest that PDXOs derived from these types of pediatric brain cancers could grow in an optimal manner in our culture conditions compared to already established ones.

### Amplification and biobanking of PDOs and PDXOs

To propagate PDOs and PDXOs with the aim of preserving them over time and using them for further translational applications, we developed a protocol for their amplification, freezing, and recovery after cryopreservation. PDXOs cultured in suspension were split through mild enzymatic disaggregation into small cell clusters (not at single cell level) and let to recover under agitation (Appendix Fig S2A). PDXOs from different MB subgroups were found to grow again after the splitting and could be kept in culture for several passages (Appendix Fig S2B and C). PDXOs could also be split more times and at different timepoints, leading to the same result (Appendix Fig S3A and B). They maintained the CNV and DNA methylation profiles of the parental tumors even after the amplification and after 2–4 months in culture (Appendix Fig S2D, Table 8). PDXOs could also be frozen (Appendix Fig S2E) and they recovered after the cryopreservation (Appendix Fig S2F). These recovered PDXOs exhibited continuous growth and similar CNV and DNA methylation profiles to their corresponding parental PDX tumors (Appendix Fig S3G, Table 8). Since this mild enzymatic disaggregation worked for PDXOs, we tried it with EPN‐PDOs (tumor #3) (Appendix Fig S2G and H) and G3 MB‐PDOs (tumor #14) (Appendix Fig S3C and D). Interestingly, disaggregated small cell clusters were very slow in the recovery after the amplification but their areas increased over time (Appendix Figs S2H and S3D). We also tried a different approach as already done (Jacob *et al*, 2020), cutting one PDO into 2/3 pieces and letting them recover under suspension on an orbital shaker (Appendix Figs S2I and S3E). However, G3 MB‐PDOs did not seem to recover better than the enzymatically disaggregated ones, and the growth remained slow (Appendix Fig S3F). Instead, for EPN‐PDOs we observed that after cutting them for the first time after 65 days of culture, they exhibited faster recovery and growth and needed to be split more often (Appendix Fig S2J). These PDOs exhibited similar CNV and DNA methylation profiles to their corresponding parental tumors after 84 days in culture (Appendix Fig S3H, Table 8). Furthermore, they were stable even after many passages *in vitro* displaying a comparable number of detectable gene variants, pathogenetic variants, TMB, and MSI compared to the parental tumors (tumor #3, PDOs at day 349 and tumor #14, PDOs at day 355, Fig 2E and H). PDOs could also be frozen, and they recovered after the cryopreservation (Appendix Fig S2K and L). Taken together, these results show that PDXOs and PDOs can be amplified and cryopreserved for further applications, even if the methods to be used are different for the two types of organoids and might be dependent on the kind of tumor from which they are derived.

**Table 8:** DNA methylation scores and methylation classes of amplified and frozen PDOs/PDXOs with relative parental tumors.

Tumor code	Model name	Subtype	Condition	DNA methylation score	Methylation class (v11b6)	Subtype score	Subtype
B	ICB984	SHH MB	PDX tumor	0.87	Medulloblastoma, subclass SHH CHL AD	0.80	MB SHH 3
			PDXOs (split day 15) day 28	0.69	Medulloblastoma, subclass SHH CHL AD	0.16	MB SHH 4
			PDXOs (split day 15) day 61	0.67	Medulloblastoma, subclass SHH CHL AD	0.12	MB SHH 4
C	HT028	Group 3 MB	PDX tumor	1	Medulloblastoma, subclass group 3	1	MB G34 II
			PDXOs (split day 35) day 50	1	Medulloblastoma, subclass group 3	1	MB G34 II
			PDXOs (split day 35) day 120	0.99	Medulloblastoma, subclass group 3	0.98	MB G34 II
			PDXOs (split D50 + frozen), day 50 + 14 after thawing	1	Medulloblastoma, subclass group 3	1	MB G34 II
			PDXOs (split D50 + frozen), day 50 + 28 after thawing	1	Medulloblastoma, subclass group 3	1	MB G34 II

Tumor code	Tumor		Condition	DNA methylation score	Methylation class (v11b4)		
3	PF ependymoma Group A		Tumor	0.99	Ependymoma, posterior fossa group A		
			PDOs (no split) day 84	0.98	Ependymoma, posterior fossa group A		
			PDOs (split day 14) day 84	0.98	Ependymoma, posterior fossa group A		

### Confirmed action of drug treatment using PDOs

We next applied PDOs model for testing drug responses *in vitro*. To verify that PDOs were responsive to the standard of care treatment, we treated EPN‐PDOs (tumor #2) and MB‐PDOs (tumor #10) with different combinations and concentrations of drugs used in clinical protocols (Appendix Fig S4, Table 9). For the EPN‐PDOs, we used temozolomide (TMZ), which seems to have poor effects in patients, and vincristine, etoposide, cyclophosphamide (VEC) combination that is one of the main therapeutic strategies (Appendix Fig S4A) (Massimino *et al*, 2013, 2016, 2022; Adolph *et al*, 2021). We tested the drugs at different concentrations, comparable to the drug levels that can be observed in brain extracellular fluid, in brain tumor tissue or already used *in vitro* (Van Den Berg *et al*, 1982; Zucchetti *et al*, 1991; Ribrag *et al*, 1993; Ghazal‐Aswad Hilary Calvert & Newell, 1996; Jacobs *et al*, 2005; Portnow *et al*, 2009; Wang *et al*, 2010; Andres *et al*, 2014; Ackland *et al*, 2016; Wada *et al*, 2016; Campagne *et al*, 2019; Patil *et al*, 2019; Herbener *et al*, 2020) (Table 9). The therapeutic response was evaluated by quantifying the percentage of cells expressing Ki67 (proliferation marker, Appendix Fig S4B and F) and cleaved caspase‐3 (apoptosis marker, Appendix Fig S4C and G). Compared to the control treatments, chemotherapy in EPN‐PDOs induced a statistically significant decrease in the Ki67 population with 1 mM temozolomide (high concentration without clinical relevance), but not with a lower drug concentration (100 μM). Interestingly, in EPN‐PDOs both the concentrations of the VEC combination (vincristine 5 ng/ml, etoposide 1 μg/ml, cyclophosphamide 500 ng/ml, and vincristine 50 ng/ml, etoposide 10 μg/ml, cyclophosphamide 5 μg/ml) decreased the number of Ki67^+^ cells (Appendix Fig S4D). On the contrary, the cleaved caspase‐3 content did not drastically change through the different temozolomide treatment conditions (Appendix Fig S4D). For SHH MB‐PDOs we used the commonly used drugs in frontline therapy for SHH MB, vincristine + methotrexate (Rutkowski *et al*, 2005; Gandola *et al*, 2009) (VM) at concentrations comparable to the drug levels that can be observed in brain extracellular fluid, in brain tumor tissue or already used *in vitro* (Van Den Berg *et al*, 1982; Zucchetti *et al*, 1991; Wang *et al*, 2010; Patil *et al*, 2019) (Appendix Fig S4E). In treated PDOs, we did not observe a significant decrease in Ki67^+^ cells compared to the non‐treated control when exposed to both low and high concentrations of vincristine, methotrexate combination (vincristine 5 ng/ml, methotrexate 1 μg/ml, and vincristine 50 ng/ml, methotrexate 10 μg/ml) (Appendix Fig S4F and H). Instead, PDOs showed a significant increase in the cleaved caspase‐3^+^ cells, but just when treated with the highest concentration of drugs (Appendix Fig S4G and H). Taken together, these results demonstrate that PDOs derived from different tumors respond in a heterogenous way to various drug treatments, similar to what has been already reported in the clinical settings (Massimino *et al*, 2013, 2016, 2022; Adolph *et al*, 2021), therefore they can be used as a reliable tool for rapid and functional testing of treatment responses *in vitro*.

**Table 9:** Drug concentrations and radiation doses in patients and PDOs.

Drug	Drug concentrations found in patients after treatment	PDOs concentrations range	References
Temozolomide	Mean peak TMZ concentration in brain: 0.6 ± 0.3 μg/ml Maximum concentrations of TMZ in the brain interstitium or CSF: 1–10 μM	100 μM – 1 mM	Massimino *et al* (2022), Adolph *et al* (2021), Jacobs *et al* (2005), Andres *et al* (2014)
Vincristine	Blood mean: 8.8 ng/ml Peak serum drug concentrations: 0.19–0.89 μM	5 ng/ml – 50 ng/ml	Massimino *et al* (2016), Massimino *et al* (2013), Massimino *et al* (2022), Ghazal‐Aswad Hilary Calvert and Newell (1996), Wada *et al* (2016), Ribrag *et al* (1993), Rutkowski *et al* (2005)
Etoposide	Tumors: 1.05 and 3.28 μg/g Plasma: 1.02–10.76 μg/ml	1 μg/ml – 10 μg/ml	Massimino *et al* (2016), Massimino *et al* (2013), Massimino *et al* (2022), Zucchetti *et al* (1991), Gandola *et al* (2009), Rutkowski *et al* (2005)
Cyclophosphamide	Plasma levels: 60–100 μM	500 ng/ml – 5 μg/ml	Massimino *et al* (2016), Massimino *et al* (2013), Massimino *et al* (2022), Campagne *et al* (2019), Gandola *et al* (2009), Rutkowski *et al* (2005), Collins and Pollack (2020)
Methotrexate	Plasma levels: 1.321 to 1.407 μM	1 μg/ml – 10 μg/ml	Massimino *et al* (2013), Gandola *et al* (2009), Rutkowski *et al* (2005), Leblond *et al* (2022), Collins and Pollack (2020)
Carboplatin	0.89 μmol/l 3.68 μg/ml 41–46 g/l	37 μg/ml (100 μM)	Massimino *et al* (2013), Ackland *et al* (2016), Portnow *et al* (2009), Wang *et al* (2010), Rutkowski *et al* (2005), Leblond *et al* (2022), Collins and Pollack (2020)
Cisplatin	Concentration in the brain: 0.33–2.90 μg/g Peak plasma concentration: 126 ng/ml – 166 ng/ml	500 ng/ml	Massimino *et al* (2022), Patil *et al* (2019), Herbener *et al* (2020), Collins and Pollack (2020)
Thiotepa	Plasma: 2.94–10.02 μg/ml	19 μg/ml (100 μM)	Van Den Berg *et al* (1982), Gandola *et al* (2009)

Radiation	Patient‐derived organoids radiation dose (Jacob *et al*, 2020)	PDOs radiation dose	References
Patients: 39–60 Gy	GBOs^7^: 5–10 Gy	5–10 Gy	Jacob *et al* (2020), Riemondy *et al* (2022), Gandola *et al* (2009)

### Translational application of therapeutic regimens to PDOs

To further verify the potential translational applications of PDOs, we mimicked the standard of care treatment for ST‐EPN and G3 MB using the corresponding derived PDOs. In particular, ST EPN‐PDOs (tumor #5, with *ZFTA‐RELA* fusion, Table 1) and G3 MB‐PDOs (tumor #14) derived from the surgically resected tumors were treated with the same protocol the correspondent patients were treated, adapted for an *in vitro* application (Table 9).

**Figure 6:**
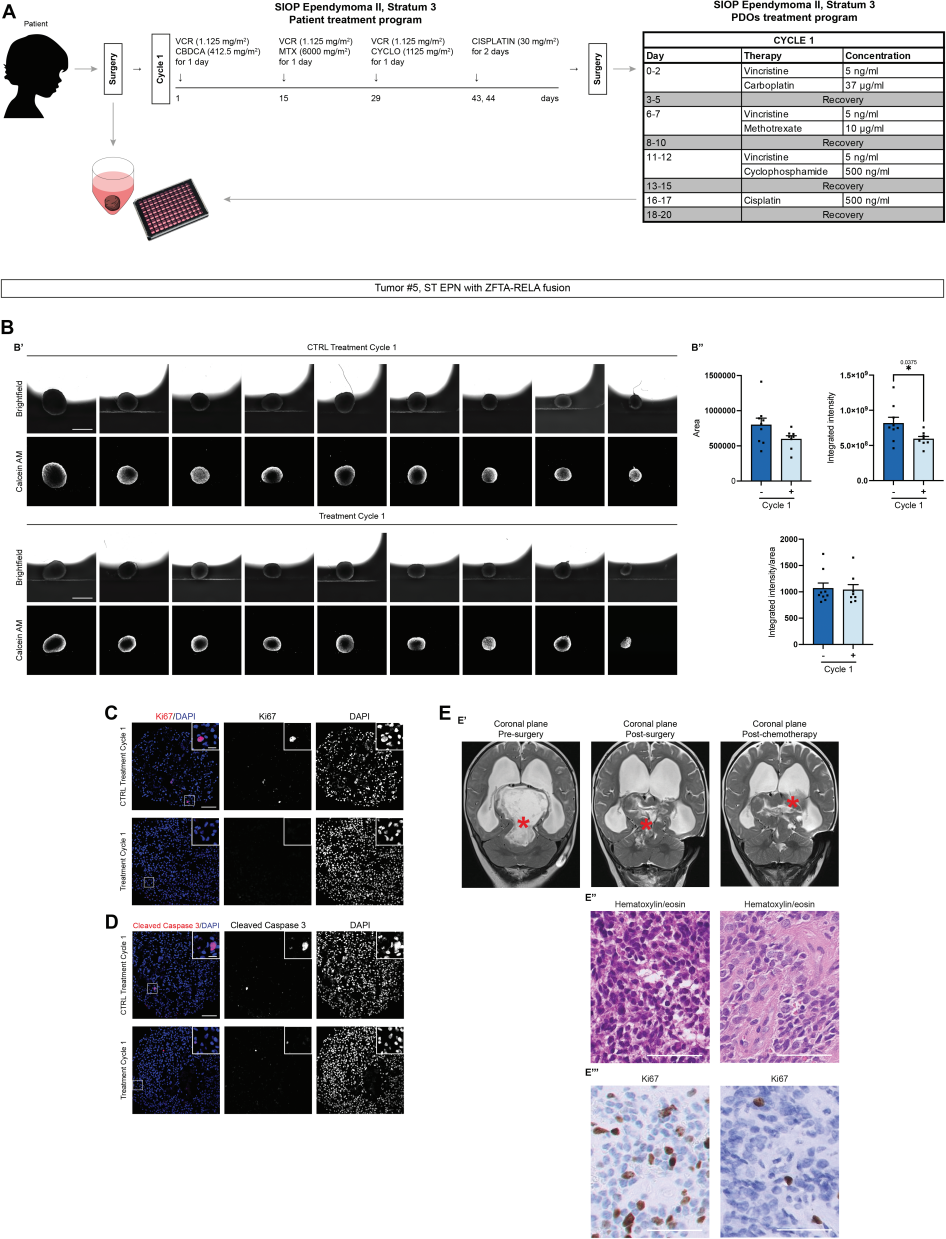
ST EPN‐derived PDOs respond to SIOP Ependymoma II, Stratum 3 protocol as the correspondent patient Summary of PDOs generation and treatment according to SIOP Ependymoma II, Stratum 3 protocol (Massimino *et al*, 2016; Leblond *et al*, 2022) for both patient and *in vitro* adaptation.PDOs live cells analysis with brightfield and fluorescence images (B′) and quantification of area, integrated intensity, and integrated intensity/area after the treatment (B″).Confocal images of immunofluorescence of Ki67 of treated ST EPN‐PDOs.Confocal images of immunofluorescence of cleaved caspase‐3 of treated ST EPN‐PDOs.T2‐weighted MRI coronal images of the patient pre‐surgery, post‐surgery and post‐chemotherapy. Morphological features (E′, E″) and immunohistochemical expression of Ki67 (E″′) of post‐initial surgery and post‐chemotherapy residual tumor. The red asterisk marks the tumor mass. Data information: Data are presented as mean ± s.e.m.; each dot represents a PDOs. For each treatment condition, *n* = 8–9 PDOs (biological replicates) were considered. Unpaired t‐test with Welch's correction or Kolmogorov–Smirnov test; *P ≤ 0.05. Exact *P* values are reported in figure. Scale bar 1 mm in (B′); 100 μm and 20 μm (higher magnification) in (C, D); 50 μm (E″, E″′). CBDCA, carboplatin; CYCLO, cyclophosphamide; MTX, methotrexate; VCR, vincristine. SIOP Ependymoma II, Stratum 3 protocol treatment experiment was performed once in ST EPN PDOs. Source data are available online for this figure.

**Figure 7:**
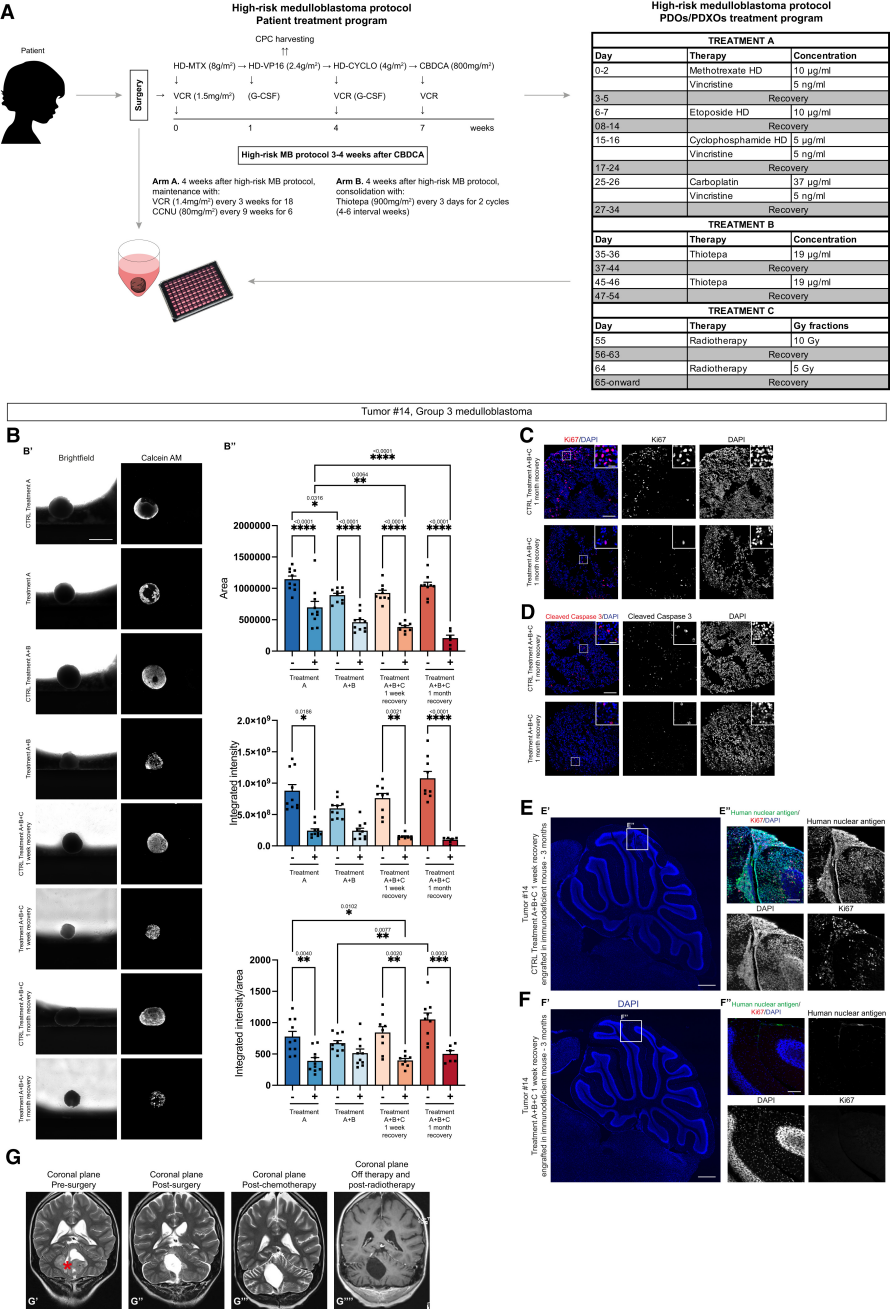
G3 MB‐derived PDOs respond to high‐risk medulloblastoma protocol as the correspondent patient ASummary of PDOs generation and treatment according to high‐risk medulloblastoma protocol (Gandola *et al*, 2009; Massimino *et al*, 2013) for both patient and *in vitro* adaptation.BPDOs live cells analysis with brightfield and fluorescence images (B′) and quantification of area, integrated intensity, and integrated intensity/area after the treatment (B″).CConfocal images of immunofluorescence of Ki67 of treated G3 MB‐PDOs.DConfocal images of immunofluorescence of cleaved caspase‐3 of treated G3 MB‐PDOs.E, FConfocal images of DAPI staining and immunofluorescence of human nuclear antigen and Ki67 of sagittal brain sections of immunodeficient mice engrafted with treatment control G3 MB‐PDOs (E) and treated G3 MB‐PDOs (F). The white square marks the region shown at higher magnification in (E″, F″).GT2‐weighted MRI coronal images of the patient pre‐surgery (G′), post‐surgery (G″), post‐chemotherapy (G″′) and post‐radiotherapy (G″″). The red asterisk marks the tumor mass. Data information: Data are presented as mean ± s.e.m.; each dot represents a PDOs. For each treatment condition, *n* = 6–10 PDOs (biological replicates) were considered. Ordinary one‐way ANOVA or Kruskal–Wallis test with Dunn's *post hoc* test; ****P ≤ 0.0001, ***P ≤ 0.001, **P ≤ 0.01, *P ≤ 0.05. Adjusted *P* values are reported in figure. Scale bar 1 mm in (B′); 100 μm and 20 μm (higher magnification) in (C, D); 500 μm (E′–F′), 100 μm (E″–F″). CBDCA, carboplatin; CCNU, lomustine; CPC, circulating progenitor cells; CYCLO, cyclophosphamide; G‐CSF, granulocyte colony‐stimulating factor; HD, high dose; MTX, methotrexate; VCR, vincristine; VP16, etoposide. High‐risk medulloblastoma protocol treatment experiment was performed once in G3 MB PDOs. Source data are available online for this figure.

ST EPN‐PDOs (tumor #5) were treated according to the adapted SIOP Ependymoma II, Stratum 3 protocol (Massimino *et al*, 2016; Leblond *et al*, 2022) (Fig 6A). This protocol was chosen because the patient was <1 year old and for this kind of patients chemotherapy is usually chosen instead of radiotherapy (Leblond *et al*, 2022). The analysis of PDOs area, fluorescence intensity of living cells (visualized through the Calcein staining) and the ratio of the two values (Fig 6B) showed no great significant difference between control and treated PDOs (Fig 6B″). In control and treated PDOs, few Ki67^+^ cells were detectable (Fig 6C), and the level of cleaved caspase‐3 was low in both conditions (Fig 6D). Interestingly, after the surgery, the patient still showed residual disease (Fig 6E′ and E″), with the presence of a few Ki67^+^ cells (Fig 6E″′). The residual disease increased after chemotherapy (Fig 6E′) and was surgically removed. On histopathological analysis, we observed a decrease in Ki67^+^ cells (Fig 6E″′), suggesting a cytostatic more than a cytotoxic effect of the treatment applied to the patient, in line also with results observed in treated PDOs. G3 MB‐PDOs (tumor #14) were treated according to the adapted high‐risk medulloblastoma protocol (Gandola *et al*, 2009; Massimino *et al*, 2013) (Fig 7A). The protocol was chosen because the patient was 15 years old and the tumor was characterized by *MYC* amplification, making it strongly aggressive (Table 1). The analysis of PDOs area, fluorescence intensity of living cells (visualized through the Calcein staining) and the ratio of the two values (Fig 7B) showed a constant and significant decrease in the area of treated PDOs compared to the control ones (Fig 7B″), as well as a significant difference in the fluorescence intensity of living cells especially in PDOs after the two doses of radiotherapy (Fig 7B″). These results were also confirmed by the decrease in Ki67^+^ cells in treated PDOs 1 month after the last dose of radiotherapy (Fig 7C), while the level of cleaved caspase‐3 was not significantly affected (Fig 7D). Most interestingly, when orthotopically injected in immunodeficient mice, control PDOs could still successfully engraft in the cerebellum and displayed tumor development when examined through human nuclear antigen immunostaining 2–3 months after engraftment (*n* = 2/3 mice) (Fig 7E). Furthermore, tumors were found positive for the proliferation marker Ki67 (Fig 7E″). On the other hand, mice engrafted with treated PDOs did not show any sign of engraftment nor tumor development (Fig 7F). The patient treated with the same protocol (Fig 7G) displayed no residual disease detectable by MRI imaging (Fig 7G″) after surgery that was still undetectable after the chemotherapy cycles (Fig 7G″′), after radiotherapy (Fig 7G″″) and at the last follow‐up, 12 months after the end of therapies, presumably also in line with PDOs sensitivity to the treatments administered.

We also treated G3 MB‐ and SHH MB‐derived PDXOs according to the high‐risk medulloblastoma protocol. The analysis of G3 MB‐PDXOs area, fluorescence intensity of living cells (visualized through the Calcein staining) and the ratio of the two values (Appendix Fig S5A) showed no significant difference between control and treated PDXOs (Appendix Fig S5A″). On the other hand, the same analysis on SHH MB‐derived PDXOs (Appendix Fig S5B) showed a significant decrease in the area and integrated intensity of living cells compared to the control ones (Appendix Fig S5B″). These data suggest a specific response of PDXOs to this protocol, probably depending on the patient from which they were derived.

Taken together, these results show the potential of PDOs model for translational applications, since in both ependymoma and medulloblastoma protocols they responded similarly to the correspondent patients.

## Discussion

Brain tumors are still fatal in children and faithful models are essential for cancer research to facilitate the study of tumor biology and to assess new anticancer therapies. In this study, we describe a PDO platform that enables generation, amplification, and biobanking of pediatric brain cancer organoids. We showed that PDOs and PDXOs could be better generated by mechanical cutting into small pieces rather than by enzymatic dissociation to single cells and further reaggregation into spheroids, as it has been already reported (Golebiewska *et al*, 2020). Depending on the type of primary tumor (i.e., EPN, MB, or LGG tumors), tumor pieces usually formed more round organoids (PDOs) within 1 week. Indeed, a difference has been observed in the generation of PDOs depending on the type of original parental tumor. In general, EPN and MB tumors survived to the *in vitro* culture, could be kept in culture for longer periods of time and could be more easily amplified for many passages. On the contrary, LGG tumors displayed more difficulties in adapting to the *in vitro* culture and could be kept in culture for shorter periods of time, apart for some specific cases (e.g., tumor #23, which could be kept in culture up to 2 months). Indeed, this could be due to the intrinsic nature of LGG tumors that in general present lower aggressiveness, growth, and capacity of infiltrating (Collins & Pollack, 2020). On the other hand, PF EPN, ST EPN with *ZFTA‐RELA* fusion, Group 3 MB with *MYC* amplification and SHH MB with *MYCN* amplification and *TP53* mutations are known to be highly aggressive and consequently leading to poor prognoses, and their mutational background could influence their maintenance *in vitro*. Furthermore, none of the mice engrafted with PDOs deriving from LGG tumors showed signs of engraftments, suggesting that the intrinsic differences characterizing different types of tumors seem to be maintained in PDOs after the *in vitro* culture. By contrast, in our hands EPN‐PDOs and MB‐PDOs successfully engrafted in mice, indicating that our PDOs can be used *in vivo* as well.

A comprehensive analysis demonstrated that when grown in PDOs medium, PDOs maintain tumor histological characteristics, DNA methylation and mutational profile, tumor heterogeneity, and biomarker expression. However, transcriptomic analysis will be necessary to confirm the adequacy and robustness of the models also at the molecular level. Organoids and corresponding primary tumors remained highly similar at the genomic level, even after several months of *in vitro* culture and after freezing and thawing passages. Nevertheless, some tumors could not be kept in culture for more than 100 days, most likely because they did not sufficiently grow. Our PDOs and PDXOs could not be established and maintained using different culture conditions (Jacob *et al*, 2020; Abdullah *et al*, 2022). This could be mostly due to the fact that other media (Jacob *et al*, 2020; Abdullah *et al*, 2022) do not contain growth factors such as FGF2 and EGF, that instead are present in our PDOs medium. Indeed, GBOs (Jacob *et al*, 2020) usually harbor EGFR variant III, making unnecessary the presence of such factors in the medium. This is not applicable to our tumors, which do not carry mutations in such gene and for which the presence of additional growth factors seems to be mandatory. Concerning LGG tumors, the culture conditions used for generation of adult LGG tumor‐derived organoids (Abdullah *et al*, 2022) did not work well for the generation of our pediatric LGG PDOs. This is in line with the notion that pediatric and adult LGG are clinically and molecularly different diseases and that pediatric LGG are generally less aggressive than the adult ones (Collins & Pollack, 2020). Lastly, scRNA‐seq analysis demonstrated that the high cellular and functional intratumoral heterogeneity in G3 MB and PF EPN Group A and the functional subpopulations described in the primary tumor were observed in the matching PDOs. We observed changes in the cellular proportions possibly reflecting adaptive mechanisms of the tumor cells in culture.

Pediatric brain tumors are frequently treated with chemotherapy and radiotherapy, depending on type of tumor, age of the patient, histopathological and molecular stratification, infiltration level, and site of onset (Louis *et al*, 2021). From a first drug testing, we found that both EPN‐ and MB‐derived PDOs were sensitive to chemotherapy‐based treatments. When treated with more specific and *ad hoc* treatment protocols established to fully mimic patients' therapeutic regimens (Rutkowski *et al*, 2005; Gandola *et al*, 2009; Massimino *et al*, 2013, 2016; Leblond *et al*, 2022), PDOs from a case of ST‐EPN with *ZFTA‐RELA* fusion responded as the patient when treated according to the SIOP Ependymoma II, Stratum 3 (Leblond *et al*, 2022). The same happened for G3 MB PDOs that were subjected to the high‐risk medulloblastoma protocol (Gandola *et al*, 2009; Massimino *et al*, 2013). Interestingly, when injected in immunodeficient mice, the treated PDOs could not engraft, suggesting a response mirroring the patient's one, currently in follow‐up with non‐evident disease. Instead, when applied to two different MB‐derived PDXOs, the treatment analysis suggested a specific response of PDXOs to this protocol, probably depending on the patient from which they were derived. These results suggest the potential of this model even at the translational level, because of the close similarity in the response to the treatments applied to the patients from which PDOs were derived.

In summary, we present a new PDO platform for the study of pediatric brain cancers. These PDOs and PDXOs biobanks can be applied not only for personalized medicine but also for wider drug screenings with the aim of finding more specific druggable targets and uncovering new therapeutic strategies.

## Materials and Methods

### Human subjects

The study included 23 individuals (12 females and 11 males, age range 6 months–15 years) cured at Bambino Gesù Children's Hospital, Rome with histological diagnosis of posterior fossa ependymomas (*n* = 6), supratentorial ependymoma (*n* = 1), medulloblastoma (*n* = 8), and low‐grade gliomas (pilocytic astrocytoma, dysembryoplastic neuroepithelial tumor—DNET, ganglioglioma, *n* = 8) (detailed in Table 1). All samples and clinical records were collected according to protocols approved by the institutional review board (Protocols no. 1863_OPBG_2019 and 2729_OPBG_2022) with written consent obtained from the patients' parents and were pseudonymized. Experiments conformed to the principles set out in the WMA Declaration of Helsinki and the Department of Health and Human Services Belmont Report. An expert neuropathologist (S.R.) reviewed the histopathological specimens and confirmed the diagnoses. The diagnoses of the low‐grade gliomas were also supported by the identification of the MAPKinase pathway alterations, for example, *BRAF‐KIAA1549* fusion. Tumors were molecularly characterized for their DNA methylation profiling. Fresh tissues were used to generate PDOs.

### Animal models

Nude mice (The Jackson laboratory, ref. NU/J (002019)) were housed in a certified specific pathogen‐free (SPF) animal facility in accordance with European Guidelines. Mice were provided ad libitum food access throughout their lifetime. All experimental procedures were approved by the Ministry of Health as conforming to the relevant regulatory standards. For orthotopic engraftment of PDOs, P4‐P5 immunodeficient mice were used; male or female mice were randomly assigned. Animals were daily monitored for any evident signs due to physical and/or neurological morbidity (ataxia, weight loss) by veterinary and biological services staff members. Animals were sacrificed when showing evident signs of neurological morbidity or at 3 months post engraftment.

### Collection and processing of fresh tumor samples from patients and PDXs and PDOs/PDXOs generation

Fresh surgically resected tumor samples from Bambino Gesù Children's Hospital, Rome or PDXs‐derived tumors from the Hopp Children's Cancer Center and German Cancer Research Center, Heidelberg were placed in Neurobasal medium (Gibco, 21103049) and shipped at room temperature within 24 h the surgical resection to University of Trento. For reliable PDOs and PDXOs generation, the tissue must be processed as soon as possible but if you cannot work on the tissue within few hours after the surgical resection, this time span has been found to be the best one in terms of time and quality of the sample. The tissue was transferred to a sterile Petri dish in fresh Neurobasal medium (Gibco, 21103049) and either enzymatically dissociated into single cells for further reaggregation with StemPro™ Accutase™ (Gibco, A1110501), 5 min at 37°C or chopped in small pieces (0.5–2 mm diameter) using scalpels under a sterile biological hood. Tumor pieces presenting high levels of blood were incubated in customized Red Blood Cells (RBC) lysis buffer for 5 min at room temperature, to remove the contaminating red blood cells. RBC lysis buffer was then removed, and tumor pieces were washed twice with 1× PBS. Single cells (for further reaggregation, 30,000–50,000 cells/well) and tumor pieces were transferred to a low‐cell‐adhesion 96‐well culture plate with V‐bottomed wells (Greiner Bio‐one, GR651970) in 150 μl of patient‐derived organoids medium (PDOs medium) containing 1:1 Neurobasal (Gibco, 21103049):DMEM/F12 (Gibco, 11320074), 50× B27 supplement (Gibco, 17504044), 100× GlutaMax (Gibco, 35050038), 100× N2 supplement (Gibco, 17502001), 20 ng/ml FGF2 (Peprotech, 100‐18B), 20 ng/ml EGF (Peprotech, 100‐47), penicillin (100 U/ml)/streptomycin (100 μg/ml) (Gibco, 15140122), 0.25 μg/ml Amphotericin (only for PDOs, Gibco, 15290018) or Heparin 2.5 μg/ml (only for PDXOs, Sigma Aldrich, H3149‐10KU) and placed in a 37°C, 5% CO_2_ incubator. PDX‐derived tumor pieces were managed with an additional condition: they were also cultured in 6‐cm/10‐cm plates (Sarstedt, 82.1194.500, 82.1472.001) in suspension in PDOs medium on an orbital shaker (70 rpm) placed in a 37°C, 5% CO_2_ incubator. Twice per week a complete change medium was performed. During the first days of culture, it was normal to observe the presence of cellular debris and leftovers of red blood cells, which disappeared within the first week. Tumor pieces formed rounded‐like organoids within the first week, depending also on tumor type and quality. All PDOs/PDXOs cultures were regularly tested and confirmed free of Mycoplasma. Primary tumor, PDX‐derived tumors, and PDOs/PDXOs were collected at different timepoints for histology, DNA methylation, and single‐cell RNA sequencing analyses (Table 10).

**Table 10:** List of performed analyses for each primary tumor/matching PDOs sample.

	Tumor	4% PFA for histological analysis	Genomic DNA for DNA methylation analysis, TSO500, RNA	scRNA sequencing	Nude mice engraftment	Drug testing
EPENDYMOMAS						
1	PF ependymoma, Group A	✓	✓			
2	PF ependymoma, Group A (relapse)	✓	✓	✓	✓	✓
3	PF ependymoma, Group A	✓	✓			
4	PF ependymoma, Group A	✓				
5	Sopratentorial ependymoma ZFTA‐RELA fusion					✓
6	PF ependymoma, Group A (relapse)	✓				
7	PF ependymoma, Group A (relapse)	✓	✓			
MEDULLOBLASTOMAS						
8	Group 4 medulloblastoma (relapse)	✓	✓			
9	SHH medulloblastoma	✓	✓		✓	
10	SHH medulloblastoma	✓	✓		✓	✓
11	Group 4 medulloblastoma	✓	✓		✓	
12	Group 4 medulloblastoma	✓	✓		✓	
13	Group 4 medulloblastoma	✓	✓		✓	
14	Group 3 medulloblastoma					✓
15	Group 3 medulloblastoma		✓	✓		
LOW‐GRADE GLIAL TUMORS						
16	Low‐grade glioma with FGFR1‐TACC1 fusion	✓	✓			
17	Dysembryoplastic neuroepithelial tumor (relapse)	✓	✓			
18	Ganglioglioma (relapse)	✓	✓		✓	
19	Pilocytic astrocytoma (relapse)	✓	✓		✓	
20	Pilocytic astrocytoma (relapse)	✓	✓		✓	
21	Pilocytic astrocytoma (relapse)	✓	✓		✓	
22	Pilocytic astrocytoma	✓	✓		✓	
23	Polymorphous low‐grade neuroepithelial tumor PLNTY	✓	✓			

For experiments with already published media, we followed the published composition (Jacob *et al*, 2020; Abdullah *et al*, 2022). In details, the GBOs medium (Jacob *et al*, 2020) contained 1:1 Neurobasal (Gibco, 21103049):DMEM/F12 (Gibco, 11320074), 100× MEM‐NEAA (Gibco, 11140035), 100× GlutaMax (Gibco, 35050038), 100× N2 supplement (Gibco, 17502001), 50× B27 supplement minus vitamin A (Gibco, 12587010), penicillin (100 U/ml)/streptomycin (100 μg/ml) (Gibco, 15140122) and 2.5 μg/ml insulin (Santa Cruz Biotechnology, sc‐29062). PDOs were placed in 6‐cm/10‐cm plates (Sarstedt, 82.1194.500, 82.1472.001) on an orbital shaker (70 rpm) in a humidified incubator at 37°C, 5% CO_2_.

The Long Term Glioma Medium (Abdullah *et al*, 2022) contained 1:1 Neurobasal (Gibco, 21103049):DMEM/F12 (Gibco, 11320074), 100× GlutaMax (Gibco, 35050038), 100× MEM‐NEAA (Gibco, 11140035), penicillin (100 U/ml)/streptomycin (100 μg/ml) (Gibco, 15140122), 50× B27 supplement minus vitamin A (Gibco, 12587010), 100× N2 supplement (Gibco, 17502001), 55 μM 2‐Mercaptoethanol (Gibco, 21985023) and 2.5 μg/ml insulin (Santa Cruz Biotechnology, sc‐29062). PDOs were placed in 24‐well ultra‐low adherence plates (VWR Avantor, 734‐2779) in a humidified incubator at 37°C, 5% CO_2_ and 5% oxygen.

### Amplification, cryopreservation, and recovery of PDOs/PDXOs

Depending on the tumor type, PDOs/PDXOs cultured for prolonged periods of time were routinely split in two ways. They were either mildly disaggregated into small cell clusters by incubation in StemPro™ Accutase™ (Gibco, A1110501) for 5 min at 37°C or cut into smaller pieces (0.5–1 mm diameter) using tweezers and scalpels. Then, they were maintained in 6‐cm/10‐cm plates in suspension in PDOs medium on an orbital shaker (70 rpm) placed in a 37°C, 5% CO_2_ incubator. Twice per week, a complete change of medium was performed.

For cryopreservation, PDOs cut into 0.5–1 mm diameter pieces or PDXOs treated with StemPro™ Accutase™ (Gibco, A1110501) for 5 min at 37°C were resuspended in freezing medium consisting of PDOs medium supplemented with 10% DMSO and placed in cryovials. PDOs/PDXOs were slowly cooled using a freezing container (Thermofisher Scientific) in a −80°C freezer. Frozen PDOs/PDXOs were placed in a liquid nitrogen tank for long‐term storage.

For recovery, cryovials were quickly thawed for 1–1.5 min in a 37°C water bath, and PDOs/PDXOs were gently moved to a 15‐ml tube. Five milliliters of PDOs medium was added to dilute the DMSO still present. PDOs/PDXOs were then centrifuged for 3 min at 200 *g* at room temperature, the medium was removed, and they were subsequently cultured in 6‐cm/10‐cm plates in suspension in PDOs medium on an orbital shaker (70 rpm) placed in a 37°C, 5% CO_2_ incubator. Twice per week, a complete change medium was performed. All PDOs/PDXOs cryopreserved cultures were confirmed free of Mycoplasma.

### Animals and orthotopic engraftment of PDOs

Orthotopic engraftment of PDOs was performed as already described for human cerebellar organoids injection into immunodeficient mice (Ballabio *et al*, 2020). PDOs between day 20–35 were used and dissociated into PDOs medium. P4–P5 immunodeficient mice were anesthetized on ice for 2 min, placed on a stage in a stereotactic apparatus and medially injected at lambda: −3.6 D/V: −1.6 with 4–5 μl of dissociated PDOs and a 30‐gauge Hamilton Syringe (Hamilton, 1701 RN).

Animals were regularly checked for the presence of any evident signs due to physical and/or neurological morbidity (ataxia, weight loss). Animals were sacrificed at 3 months or at human endpoint as they displayed signs of morbidity after the administration of a lethal dose of anesthesia by intraperitoneal injection.

### Histological and immunohistochemical analysis

PDOs and PDXOs were fixed in 4% paraformaldehyde in PBS at 4°C overnight, cryoprotected in 30% sucrose in distilled H_2_O at 4°C overnight and embedded in Frozen Section Compound (Leica, 3801480). Frozen PDOs were kept at −20°C until processing. PDOs/PDXOs cryosections at 20 μm were prepared with a cryostat (Thermo Scientific HM525 NX) on glass slides (Thermofisher Scientific, J1800AMNZ). Slides were stored at −20°C until immunohistology.

Immunodeficient mice brains were dissected and post‐fixed in 4% paraformaldehyde in PBS overnight, cryoprotected in 30% sucrose in distilled H_2_O at 4°C for 2 days, and embedded in Frozen Section Compound (Leica, 3801480). Frozen brains were kept at −20°C until processing. Mice brains cryosection at 60 μm was prepared with a cryostat (Thermo Scientific HM525 NX) on glass slides (Thermofisher Scientific, J1800AMNZ). Slides were stored at −20°C until immunohistology.

Immunohistochemistry was carried out on frozen sections using an automated immunostainer (Dako Omnis). Primary antibodies used for immunohistochemistry are listed below:

**Table:** 

Primary antibody	Dilution	Reference
OLIG2	1:20	Quartett
Polyclonal anti‐GFAP	Prediluted	Dako
Synaptophysin (clone DAK‐SYNAP)	Prediluted	Dako
H3.3K27me3 (clone C36B11)	1:200	Cell Signaling technology

*List of primary antibodies for immunohistochemistry*.

All the slides of primary tumors used for H&E and the immunostainings were retrieved from the archive of the Pathology Unit of Bambino Gesù Children's Hospital. The morphology and immunophenotype of the paired parental tumor/PDOs samples were evaluated and compared.

For immunofluorescence staining of primary tumors, paraffin sections were rehydrated, and antigen retrieval was performed by incubating slices for 15 min in retrieval solution (10 mM sodium citrate, 0.5% Tween‐20 v/v, pH 6.0) at 98°C. Primary antibodies were incubated overnight at 4°C in antibody solution (PBS supplemented with 0.3% Triton™ X100, Sigma‐Aldrich, T8787; 3% goat serum, Gibco, 16210064) and secondary antibodies for 1 h at room temperature. Nuclei were counterstained with DAPI 10 mM (Abcam, ab228549).

For immunofluorescence staining of PDOs/PDXOs and mice brains, cryosections were treated with a permeabilization solution (PBS supplemented with 3% BSA, Seqens/H2B, 033IDB1000‐70; 0.3% Triton™ X100, Sigma‐Aldrich, T8787; 5% goat serum, Gibco, 16210064) for 1 h at room temperature. Primary antibodies were incubated overnight at 4°C in antibody solution (PBS supplemented with 3% BSA, Seqens/H2B, 033IDB1000‐70; 0.1% Triton™ X100, Sigma‐Aldrich, T8787; 1% goat serum, Gibco, 16210064) and secondary antibodies for 1 h at room temperature. Nuclei were counterstained with DAPI 10 mM (Abcam, ab228549).

Sections and coverslips (Thermofisher Scientific, 15747592) were mounted with permanent mounting medium (Histo‐Line laboratories, PMT030).

The used antibodies are listed below:

**Table:** 

Primary antibody	Dilution	Reference
Mouse monoclonal anti‐Human Nuclear Antigen (235–1)	1:200	Abcam, ab191181
Mouse monoclonal anti‐Nestin (10C2)	1:500	Abcam, ab22035
Mouse monoclonal anti‐NGFR p75 (B‐1)	1:200	Santa Cruz Biotechnology, sc‐271708
Mouse monoclonal anti‐SOX2 (20G5)	1:200	Abcam, ab171380
Mouse monoclonal anti‐Tubulin β 3 (TUBB3)	1:1,000	Biolegend, 801201
Mouse monoclonal recombinant anti‐YAP1 (63.7)	1:200	Santa Cruz Biotechnology, sc‐101199
Rabbit monoclonal recombinant anti‐CD34 (EP373Y)	1:500	Abcam, ab81289
Rabbit monoclonal recombinant anti‐Iba1 (EPR16588)	1:500	Abcam, ab178846
Rabbit monoclonal recombinant anti‐Synaptophysin (YE269)	1:500	Abcam, ab32127
Rabbit polyclonal anti‐Cleaved Caspase‐3 (Asp175)	1:200	Cell Signaling Technology, 9661
Rabbit polyclonal anti‐GFAP	1:200	Sigma Aldrich, G9269
Rabbit polyclonal anti‐Ki67	1: 500	Abcam, ab15580
Rabbit polyclonal anti‐Olig2	1:200	Sigma Aldrich, AB9610
Rabbit polyclonal anti‐SOX9	1:2,000	Sigma Aldrich, AB5535
Rat monoclonal anti‐CD3 epsilon (CD3‐12)	1:200	GeneTex, GTX11089

*List of primary antibodies for immunofluorescence staining*.

**Table:** 

Secondary antibody	Dilution	Reference
Alexa Fluor 488 goat anti‐rabbit IgG	1:500	Thermofisher Scientific, A11008
Alexa Fluor 546 goat anti‐rabbit IgG	1:500	Thermofisher Scientific, A11035
Alexa Fluor 647 goat anti‐rabbit IgG	1:500	Thermofisher Scientific, A21245
Alexa Fluor 488 goat anti‐mouse IgG	1:500	Thermofisher Scientific, A11001
Alexa Fluor 546 goat anti‐mouse IgG	1:500	Thermofisher Scientific, A11030
Alexa Fluor 647 goat anti‐mouse IgG	1:500	Thermofisher Scientific, A21235
Alexa Fluor 647 goat anti‐rat IgG	1:500	Thermofisher Scientific, A21247

*List of secondary antibodies for immunofluorescence staining*.

### Genomic DNA extraction

PDOs/PDXOs were lysed in lysis buffer (20 mM EDTA, 10 mM Tris, 200 mM NaCl, 0.2% Triton X‐100, 100 μg/ml Proteinase K, pH 8.0) for 1 h at 37°C under agitation. Genomic DNA was extracted with phenol‐chloroform and precipitated with isopropanol.

### DNA methylation profiling

DNA methylation profiling was performed according to protocol approved by Bambino Gesù Children's Hospital Ethical Committee (Protocol no. 1556_OPBG_2018, 15^th^ January 2019), after obtaining written consent from the patients' parents. DNA was extracted from fresh frozen or formalin‐fixed paraffin‐embedded (FFPE) tissues using MagPurix DNA Extraction Kit (Resnova, Rome, Italy) for automatic extraction of genomic DNA. The sample was analyzed using Illumina Infinium Human Methylation EPIC BeadChip (EPIC) arrays (Illumina, San Diego, USA) according to the manufacturer's instructions, on Illumina iScan Platform (Illumina, San Diego, USA) as previously reported (Ballabio *et al*, 2020). Genomic DNA samples of PDX models (extracted from tumor fresh frozen material) and PDXOs have been processed using the EPIC arrays, following the manufacturer's instructions, at the DKFZ Genomics and Proteomics Core Facility (Heidelberg, Germany). Generated methylation data were compared to brain tumor classifier v11b4‐v11b6 (Capper *et al*, 2018), while the most recent classifier version v12.5 was consulted for tumor subtype annotation. High‐density DNA methylation arrays allowed for determining copy number alterations that were generated as described (Capper *et al*, 2018).

### DNA/RNA analysis

Formalin‐fixed and paraffin‐embedded (FFPE) tissue specimens of biopsies were collected from selected patients after obtaining written consent from the patients' parents. DNA and RNA were extracted from formalin‐fixed paraffin‐embedded tumor tissue using Maxwell CSC instrument (Promega, Madison, USA), respectively, with the Maxwell RSC DNA FFPE kit and Maxwell RSC RNA FFPE kit (Promega, Madison, USA). The nucleic acid concentrations were measured on a Qubit 2.0 Fluorometer (Thermofisher Scientific, Waltham, USA) using the Qubit dsDNA and RNA High Sensitivity kit.

Libraries from primary tumors and PDOs were constructed through the TruSight Oncology 500 (TSO500) Library Preparation Kit (Illumina, San Diego, USA) following the manufacturer's protocol. The TSO500 assay is a comprehensive genomic profiling tool performed in NGS and targeting 523 cancer‐relevant genes. The assay detects indels, small nucleotide variants (SNVs), splice variants and copy‐number/structural variations in several genes and also provides the tumor mutation burden (TMB) and microsatellite status (MSI).

Final DNA libraries were pooled and denatured following the manufacturer's protocol, then diluted to the appropriate loading concentration and loaded using NEXTSEQ 550 platform (Illumina, San Diego, USA) in paired‐end mode (2 × 101‐bp reads) and sequenced to a mean coverage depth of > 500×.

NGS data were analyzed with Illumina TruSight Oncology 500 Local App v2.1 and for interpretation and reporting, variant report files can be uploaded into the Pierian Clinical Genomics Workspace cloud (Pierian DX software CGW_V6.21.1).

To detect mutational status of *BRAF* codon 600, tumor DNA, and a control DNA were simultaneously processed as recommended in the EasyPGX^®^ ready *BRAF* kit protocol (Diatech Pharmacogenetics, Jesi, Italy). This assay is based on one‐step Real Time PCR for the detection of the main mutations of codon 600 of the gene *BRAF* using four oligo mixes; each mix allows the co‐amplification of the mutated alleles plus an endogenous control gene. Data were analyzed by EasyPGX^®^ Analysis Software (Diatech Pharmacogenetics, Jesi, Italy).

RNA assay was performed using Archer fusion plex custom Kit (Invitae, San Francisco, CA) following the manufacturer's protocols. RNA libraries were pooled in equimolar amounts of 4 nM (12 libraries/pool) and loaded at 10 pM with MiSeq Reagent Kits v3 600 cycles on MiSEQ platform (Illumina, San Diego, California). NGS data were analyzed using Archer Data Analysis Software v6.2.3.

### Tissue dissociation, single‐cell library preparation, and sequencing

For the preparation of samples intended for single‐cell RNA sequencing, multiple tumor pieces and PDOs were collected in a 1.5‐ml tube and dissociated with Trypsin (Gibco, 25200056) for 5–10 min at 37°C, under agitation. Samples were mechanically but gently dissociated, and trypsin was inactivated with the same volume of 0.1% BSA (Seqens/H2B, ref. 033IDB1000‐70) in PBS. Samples were centrifuged for 5 min at 300 *g* at room temperature, resuspended in 0.1% BSA in PBS and then strained through a 30 μm filter (BD Bioscience, 340626) into a 15‐ml tube. Filter was generously washed with 0.1% BSA in PBS to recover all cells. Cells were then centrifuged for 5 min at 300 *g* at room temperature, resuspended in freezing medium consisting of PDOs medium supplemented with 10% Fetal Bovine Serum (Gibco, 10270106) and 10% DMSO and placed in cryovials (10X Genomics.com, 2021). Samples were analyzed and counted for viability by trypan blue staining. PDOs‐derived single cells were slowly cooled using a freezing container (Thermofisher Scientific) in a −80°C freezer and placed in liquid nitrogen tank for long‐term storage.

Transcriptome single‐cell datasets of dataset of tumor #2 and matching PDOs (PDO Day 14 and PDO Day 28) and tumor #15 and matching PDOs (PDO Day 28, PDO Day 61) tumor have been generated using the Chromium Next GEM Single Cell 3´v3.1 (10X Genomics). Dual‐indexed libraries have been then sequenced on a Novaseq‐6000 (Illumina) as paired‐end runs and with SP reagent kit (Illumina). Sequencing was performed using the following parameters: Read 1: 28 cycles; i7 Index: 10 cycles; i5 Index: 10 cycles; Read 2: 90 cycles.

### Single‐cell RNA sequencing data processing

Chromium single‐cell sequencing raw (FASTQ format) data have been pre‐processed for the quantification of gene expression running Cell Ranger 6.1.2 (10X Genomics) and using the human genome reference GRCh38 for sequence alignment. The estimated number of cells, as well as the average number of reads per cell and of genes per cell across samples, have been reported in Table 5. Next, we further processed the gene expression matrices using Seurat v.3.2.3 package (Stuart *et al*, 2019). For downstream analysis, we filtered out the cells based on the following criteria: expressing less than 300 genes; with high mitochondrial content (≥ 20%); doublets (cells classified using DoubletFinder function); nCount < 1,000 and > 4,000; nFeatures < 500 and > 8,000. We also excluded the genes detected in less than five cells.

For each dataset, cells from the three different samples (tumor and 2 PDOs) have been next integrated based on the reciprocal PCA (“RPCA”) approach, performed with FindIntegrationAnchors (settings: 5,000 genes selected for data integration) and IntegrateData function in Seurat. Unsupervised cluster analysis was performed by exploiting the shared nearest neighbor (SNN) modularity clustering algorithm implemented in Seurat function FindClusters. Differentially expressed genes across the cell clusters were annotated using the Wilcox test in Seurat function FindAllMarkers. Assignment of cell type annotation has been performed both considering the top cluster markers and by leveraging publicly available reference single‐cell medulloblastoma (Riemondy *et al*, 2022) and ependymoma (Gillen *et al*, 2020; Gojo *et al*, 2020) transcriptomic datasets using the SingleR method.

Data plots have been generated using Seurat and Scpubr (v 1.1.2) packages.

### Drug treatments of PDOs

For drug treatment experiments, PDOs were cultured for 5 days in PDOs medium added with different combinations and concentrations of drugs, as listed: temozolomide 100 μM or temozolomide 1 mM (MedChemExpress, HY‐17364); vincristine 5 ng/ml (Selleckchem, S9555) + etoposide 1 μg/ml (Selleckchem, S1225) + cyclophosphamide 500 ng/ml (Selleckchem, S2057) or vincristine 50 ng/ml + etoposide 10 μg/ml + cyclophosphamide 5 μg/ml; vincristine 5 ng/ml + methotrexate 1 μg/ml (Selleckchem, S1210) or vincristine 50 ng/ml + methotrexate 10 μg/ml.

PDOs were kept in Ibidi uncoated 96‐well black μ‐plates (Ibidi, 89621) placed in a 37°C, 5% CO_2_ incubator. A complete change medium was performed every 48 h for all combinations of drugs but temozolomide, for which a complete change medium was performed every 24 h. PDOs were eventually collected, fixed in 4% paraformaldehyde overnight at 4°C, and stained as previously described.

For adaptation of SIOP Ependymoma II, Stratum 3 (Leblond *et al*, 2022) (EPN drugs protocol), PDOs were treated according to table in Fig 6. The combinations of used drugs with the relative concentrations are the following: vincristine 5 ng/ml + carboplatin 37 μg/ml (Selleckchem, S1215); vincristine 5 ng/ml + methotrexate 10 μg/ml; vincristine 5 ng/ml + cyclophosphamide 500 ng/ml; cisplatin 500 ng/ml (Selleckchem, S1166). PDOs were subjected to 2 days of treatment with each combination of drugs and to 3 days of recovery between the administrations of each combination.

For adaptation of high‐risk medulloblastoma protocol (Gandola *et al*, 2009; Massimino *et al*, 2013) (MB drugs protocol), PDOs were treated according to table in Fig 7. Treatment A consisted of the following combinations of drugs: methotrexate 10 μg/ml + vincristine 5 ng/ml; etoposide 10 μg/ml; cyclophosphamide 5 μg/ml + vincristine 5 ng/ml; carboplatin 37 μg/ml + vincristine 5 ng/ml. PDOs were subjected to 2 days of treatment with each combination of drugs and to 3 days of recovery between the first two combinations of drugs and to 1‐week recovery between the others. Treatment B consisted of two doses of thiotepa 19 μg/ml (Selleckchem, S1775). PDOs were subjected to 2 days of treatment with each dose of thiotepa and to 1 week of recovery between the two. Treatment C consisted of 2 doses of radiotherapy: 10 Gy and 5 Gy (delivering a dose rate of 1.6 Gy/min). Radiotherapy was performed using the Xstrahl RS225 X‐ray research irradiator (West Midlands, UK).

PDOs were live‐stained with 2 μM Calcein, AM, cell‐permeant dye (Invitrogen, C1430) for 3 h at 37°C and imaged at different timepoints using the ImageXpress Micro Confocal High Content Imaging System (Molecular Devices). For EPN drugs protocol, PDOs were imaged at the end of the protocol (day 20 of treatment). For MB drugs protocol, PDOs were imaged after Treatment A, Treatment A + B, Treatment A + B + C – 1 week of recovery, and Treatment A + B + C – 1 month of recovery. In detail, PDOs were first whole imaged with a 2X Plan Apo objective in widefield mode and centered using the QuickID procedure. For each object (i.e., PDO), the XY centroids position was determined by automated segmentation and used for the following imaging. For this, a z‐series of 52 z‐steps with a 10 μm step size was acquired with a 4X Plan Apo Objective in confocal mode for the fluorescent channel of Calcein AM and the brightfield one. PDOs images were analyzed with the MetaXpress® High‐Content Image Acquisition and Analysis (Molecular Devices). First, for each PDOs and each timepoint, the maximum intensity projection of the acquired z‐series for the fluorescent channel of Calcein AM was obtained. Then, from the maximum intensity projection of each PDOs at every timepoint, the fluorescence integrated intensity (i.e., the sum of the pixel intensity over all of the pixels in an object) and the total area were calculated. PDOs were eventually collected, fixed in 4% paraformaldehyde overnight at 4°C, and stained as previously described.

### Quantification and statistical analysis

Studies were not blinded. PDOs/PDXOs were randomly used for the different experimental procedures. PDOs and PDXOs not properly formed according to “Collection and processing of fresh tumor samples from patients and PDXs and PDOs/PDXOs generation” section were excluded from the following analyses. All statistical tests and sample sizes are included in the Figure Legends and text. Statistical tests were performed with Prism. *P* values are represented as follows: ****P ≤ 0.0001, ***P ≤ 0.001, **P ≤ 0.01, *P ≤ 0.05, and not statistically significant when *P* > 0.05.

For all quantifications of immunohistology, samples being compared were processed in parallel and images were acquired using the same settings and laser power. Cells positive for a determined marker were manually quantified using the cell counter function in ImageJ.

For cellular population analysis, the same number of images for a determined sample were used for the quantification. A specific area of a region of interest (ROI) was defined and used across all images, avoiding edges or bad regions of the images. A total number of 600–1,500 DAPI^+^ cells were counted inside the ROI, equally splitting them across considered images. These DAPI^+^ cells were then checked for the positivity for the marker of interest. Data are presented as mean ± s.e.m. of the percentage of specific marker^+^ cells/DAPI; each dot represents a ROI/image. The Shapiro–Wilk test was used to validate the assumption of normality. Statistical significance was determined using either the Kolmogorov–Smirnov test for data with non‐normal distribution or the Kruskal–Wallis test with Dunn's *post hoc* test for data with non‐normal distribution.

For PDOs/PDXOs growth analysis over time, images of PDOs/PDXOs were taken every 1–2 weeks. The areas of each PDOs/PDXOs were quantified using ImageJ by outlining each organoid and measuring the area within the outlined region. Data are presented as mean ± s.e.m. of the PDOs/PDXOs areas in μm^2^ for a given time point; each dot represents a PDO's area.

For drug treatment analysis, all Ki67^+^ and cleaved caspase‐3^+^ cells for each treatment condition were counted and normalized to the PDOs area. PDOs areas were quantified using ImageJ by outlining each organoid and measuring the area within the outlined region. Data are presented as mean ± s.e.m. of Ki67^+^ cells/mm^2^ PDO area or cleaved caspase‐3^+^ cells/mm^2^ PDO area; each dot represents a PDO's section. The Shapiro–Wilk test was used to validate the assumption of normality. Statistical significance was determined using the Kruskal–Wallis test with Dunn's *post hoc* test for data with non‐normal distribution.

For EPN drugs protocol and MB drugs protocol analysis, 2–15 PDOs/PDXOs were considered for the imaging and following analysis. Data are presented as mean ± s.e.m. of PDOs/PDXOs area, Calcein integrated intensity and integrated intensity/area; each dot represents a PDOs/PDXOs. The Shapiro–Wilk test was used to validate the assumption of normality. Statistical significance was determined using either unpaired *t*‐test with Welch's correction or ordinary one‐way ANOVA for data with normal distribution and either Kolmogorov–Smirnov test or Kruskal–Wallis test with Dunn's *post hoc* test for data with non‐normal distribution.

### Microscopy and image processing

Live imaging on PDOs/PDXOs was performed either with Ibidi uncoated 96‐well black μ‐plates (Ibidi, 89621) by Nikon TI2 equipped with spinning disk X‐light V2 (10X, 20X objectives) with NIS Element software (version 5.21.03) or with 6‐cm plates by Leica DM IL LED (5X, 10X objectives) with LAS X Life Science software. PDOs/PDXOs and brains sections immunohistology were acquired by either confocal imaging by Leica TCS Sp8 (10X, 20X objectives) and Leica Application Suite X software (version 3.5.7.23225) or by Nikon TI2 equipped with spinning disc X‐light V2 (10X objective) with NIS Element software (version 5.21.03). Images were analyzed and processed using ImageJ software.

## Author contributions

**Luca Tiberi:** Conceptualization; supervision; funding acquisition; investigation; writing – original draft; project administration; writing – review and editing. **Chiara Lago:** Conceptualization; data curation; formal analysis; supervision; validation; methodology; writing – original draft; project administration; writing – review and editing. **Aniello Federico:** Data curation; formal analysis; methodology; writing – original draft; writing – review and editing. **Gloria Leva:** Data curation; formal analysis; methodology; writing – review and editing. **Norman L Mack:** Resources; methodology. **Benjamin Schwalm:** Resources; methodology. **Claudio Ballabio:** Conceptualization; methodology. **Matteo Gianesello:** Investigation; methodology. **Luana Abballe:** Resources; data curation; methodology. **Isabella Giovannoni:** Resources; data curation; methodology. **Sofia Reddel:** Resources; data curation; methodology. **Sabrina Rossi:** Resources; data curation; methodology. **Nicolas Leone:** Methodology. **Andrea Carai:** Resources; investigation; methodology. **Angela Mastronuzzi:** Resources; data curation; methodology. **Alessandra Bisio:** Methodology. **Alessia Soldano:** Conceptualization; writing – review and editing. **Concetta Quintarelli:** Resources; data curation; methodology. **Franco Locatelli:** Supervision; funding acquisition; writing – original draft. **Marcel Kool:** Conceptualization; supervision; funding acquisition; writing – original draft; project administration; writing – review and editing. **Evelina Miele:** Conceptualization; resources; data curation; supervision; funding acquisition; investigation; writing – original draft; project administration; writing – review and editing.

## Disclosure and competing interests statement

The authors declare that they have no conflict of interest.

## Supporting information



Appendix

Expanded View Figures PDF

Dataset EV1

Dataset EV2

PDF+

Source Data for Figure 1

Source Data for Figure 2

Source Data for Figure 3

Source Data for Figure 4

Source Data for Figure 5

Source Data for Figure 6

Source Data for Figure 7

## Data Availability

This study includes data deposited in external repositories: Figure 2A–C: Gene Expression Omnibus GSE247381 (https://www.ncbi.nlm.nih.gov/geo/query/acc.cgi?acc=GSE247381), GSE247231 (https://www.ncbi.nlm.nih.gov/geo/query/acc.cgi?acc=GSE247231). Figure 4A and B: Gene Expression Omnibus GSE247381 (https://www.ncbi.nlm.nih.gov/geo/query/acc.cgi?acc=GSE247381), GSE247380 (https://www.ncbi.nlm.nih.gov/geo/query/acc.cgi?acc=GSE247380). Figure 5L and M: Gene Expression Omnibus GSE247381 (https://www.ncbi.nlm.nih.gov/geo/query/acc.cgi?acc=GSE247381), GSE247231 (https://www.ncbi.nlm.nih.gov/geo/query/acc.cgi?acc=GSE247231).
