# Supplementary material for: Patient‐ and xenograft‐derived organoids recapitulate pediatric brain tumor features and patient treatments
Source: EMBO Mol Med. 2023 Nov 30;15(12):e18199. doi: 10.15252/emmm.202318199 (PMC10701620; doi:10.15252/emmm.202318199)
Supplement: Supplementary file 2 — Expanded View Figures PDF [file EMMM-15-e18199-s002.pdf]

Expanded View Figures

Figure EV1. *In vitro* culture of patient-derived organoids (PDOs).

A Schematic representation of primary tumor samples management for generation of PDOs as tumor single cells spheroid.
B–D Brightfield images of tumor single cells spheroids EPN- (B), MB- (C), and LGG- (D) derived PDOs at different timepoints.
E, F Confocal images of DAPI staining and immunofluorescence of human nuclear antigen and Ki67 of sagittal brain sections of immunodeficient mice engrafted with MB-derived PDOs.
G, H Copy number variation profiles comparison between primary parental tumor and 3 different MB-PDOs (H) and EPN-PDOs (G).

Data information: X axis: chromosomes; Y axis: Log_2 copy number ratio. Scale bar 200 μm (B–D), 100 μm (E, F). DNA methylation (CNV) experiments (G, H) were performed once per primary tumor/matching PDOs.

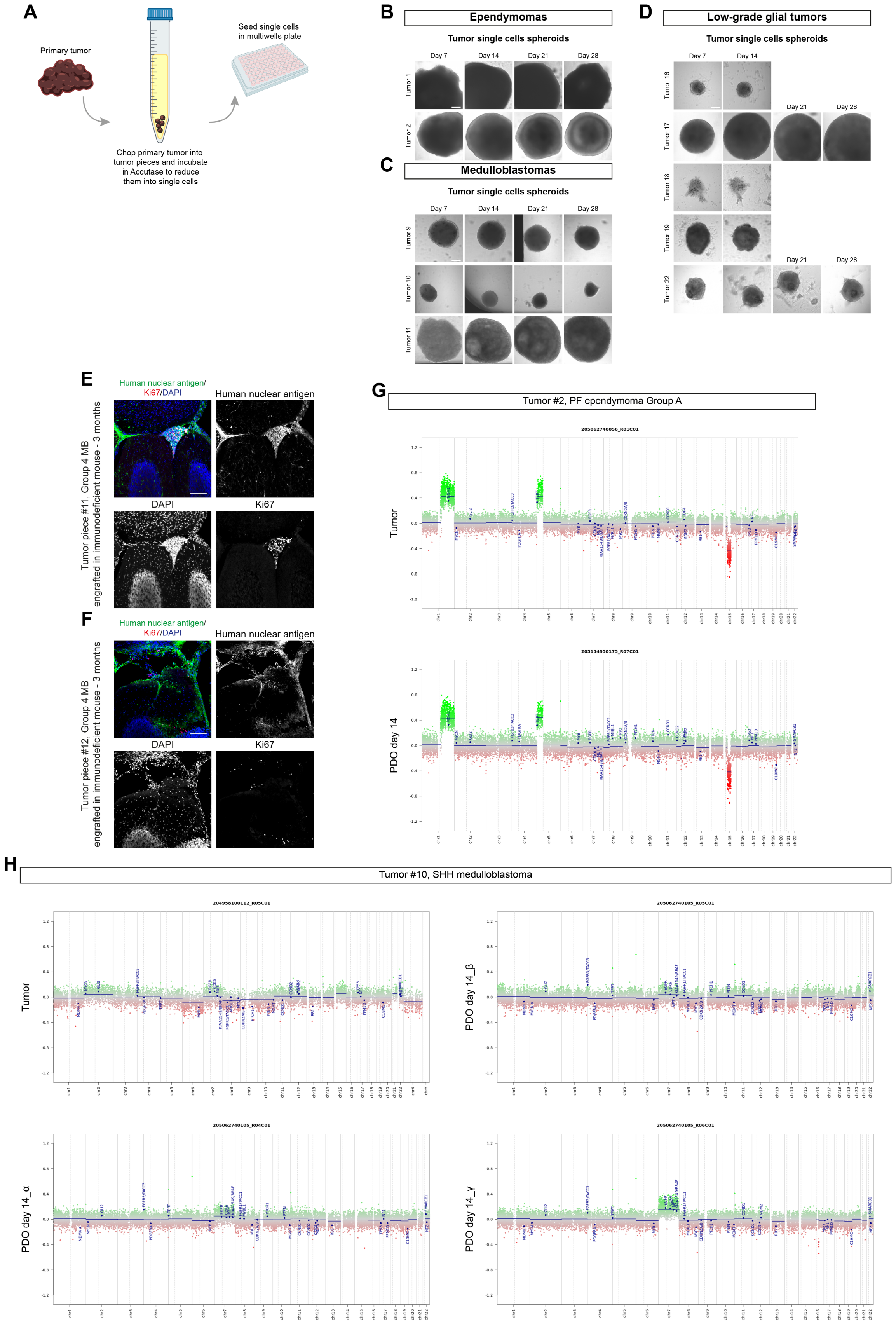

Figure EV1.

© 2023 The Authors

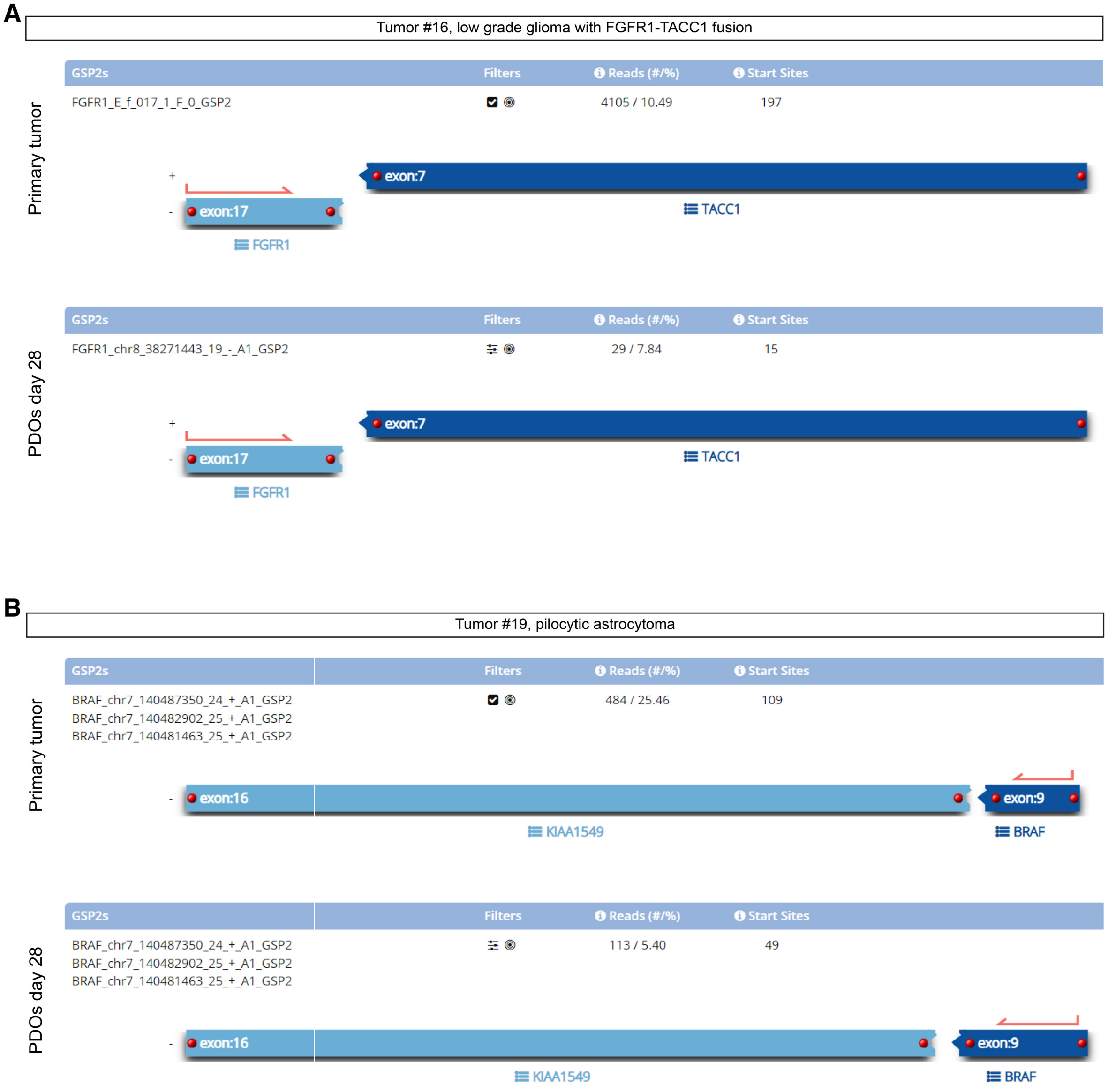

Figure EV2. Data from RNA-based assay showed maintenance of genomic features of fusion-positive tumors and their corresponding PDOs.

A Tumor #16, LGG with *FGFR1-TACC1* fusion compared to derived PDOs at day 28: the assay detects the same fusion.

B Tumor #19, *KIAA1549-BRAF* fusion was detected in pilocytic astrocytoma and in derived PDOs at day 28. Both PDOs shared the same breakpoints compared to primary parental tumors.

Data information: Experiments were performed once per primary tumor/matching PDOs.

Figure EV3. Maintenance of cellular heterogeneity in PDOs and comparison with already published medium.

A–C Confocal images of immunofluorescence of Ki67, SOX2, OLIG2, Nestin, IBA1, CD3, GFAP, B3-tubulin, CD34 of EPN- (A′–A″), MB- (B) and LGG- (C) derived PDOs.

D Brightfield images of LGG-derived PDOs as tumor pieces at different timepoints in PDOs medium and cultured according to (Abdullah *et al*, 2022) (D′), confocal images of immunofluorescence of Ki67, SOX2, OLIG2, Nestin, IBA1, CD3, GFAP, B3-tubulin (D″) and quantification in PDOs of Ki67$^+$, SOX2$^+$, OLIG2$^+$ and IBA1$^+$ cells (D‴).

E, F Confocal images of immunofluorescence of YAP1 (E′, F′) and p75 NGFR (E″, F″) of MB-derived PDOs.

G, H Confocal images of immunofluorescence of synaptophysin of LGG-derived PDOs. Quantifications are shown as percentage of specific marker$^+$ cells/DAPI (D‴).

Data information: Data are presented as mean ± s.e.m.; each dot represents a ROI/image. For each marker, n = 5–7 ROI/image of primary tumor was considered. For each marker, n = 2–3 PDOs (biological replicates) were considered; for each PDO, n = 3–4 ROI/image was used. Quantification experiments were performed once per primary tumor/matching PDOs. Kruskal–Wallis test with Dunn's *post hoc* correction; $^{**}P \leq 0.01$, $^{*}P \leq 0.05$. Adjusted and exact P values are reported in figure. The white arrows highlight specific cells. Scale bar 50 μm (A–C, D″, E–H), 200 μm (D′).

 © 2023 The Authors

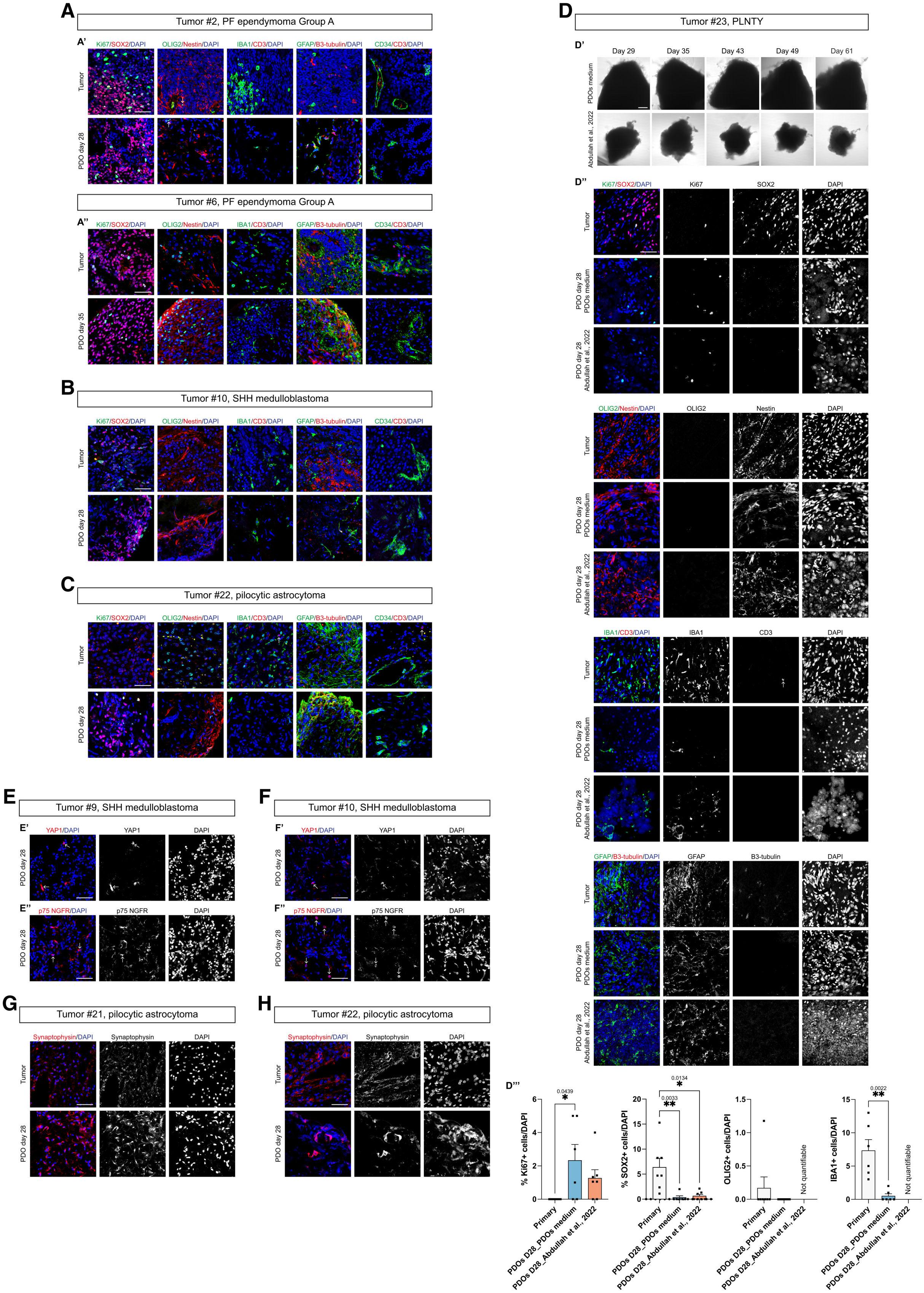
A
Tumor #2, PF ependymoma Group A
Tumor #6, PF ependymoma Group A
B
Tumor #10, SHH medulloblastoma
C
Tumor #22, pilocytic astrocytoma
D
Tumor #23, PLNTY
Day 29
Day 35
Day 43
Day 49
Day 61
PDOs medium
Abdullah et al., 2022
Ki67/SOX2/DAPI
OLIG2/Nestin/DAPI
IBA1/CD3/DAPI
GFAP/B3-tubulin/DAPI
CD34/CD3/DAPI
Tumor
PDO day 28
PDO day 35
E
Tumor #9, SHH medulloblastoma
F
Tumor #10, SHH medulloblastoma
YAP1/DAPI
p75 NGFR/DAPI
G
Tumor #21, pilocytic astrocytoma
H
Tumor #22, pilocytic astrocytoma
Synaptophysin/DAPI
% Ki67+ cells/DAPI
% SOX2+ cells/DAPI
OLIG2+ cells/DAPI
IBA1+ cells/DAPI
Primary
PDOs D28_PDOs medium
PDOs D28_Abdullah et al., 2022
Not quantifiable
0.0439
0.0033
0.0134
0.0022

Figure EV3.

Figure EV4. Maintenance of morphological features and cellular heterogeneity in PDOs and PDOs-derived tumors.

A–C Morphological features (A′, B′, C′) and immunohistochemical expression of lineage markers GFAP (A″), H3K27me3 (A‴), synaptophysin (B″), and OLIG2 (C″) of 2 EPN, 2 MB and 2 LGG paired parental tumors/PDOs samples.

D, F Confocal images of immunofluorescence of human nuclear antigen and GFAP of sagittal brain sections of immunodeficient mice engrafted with EPN- (D) and MB- (F) derived PDOs.

E, G Confocal images of immunofluorescence of human nuclear antigen and OLIG2 of sagittal brain sections of immunodeficient mice engrafted with EPN- (E) and MB- (G) derived PDOs.

H Confocal images of immunofluorescence of human nuclear antigen and SOX9 of sagittal brain sections of immunodeficient mice engrafted with MB-derived PDOs.

Data information: Scale bar 100 μm (A, C, D–H), 50 μm (B).

 © 2023 The Authors

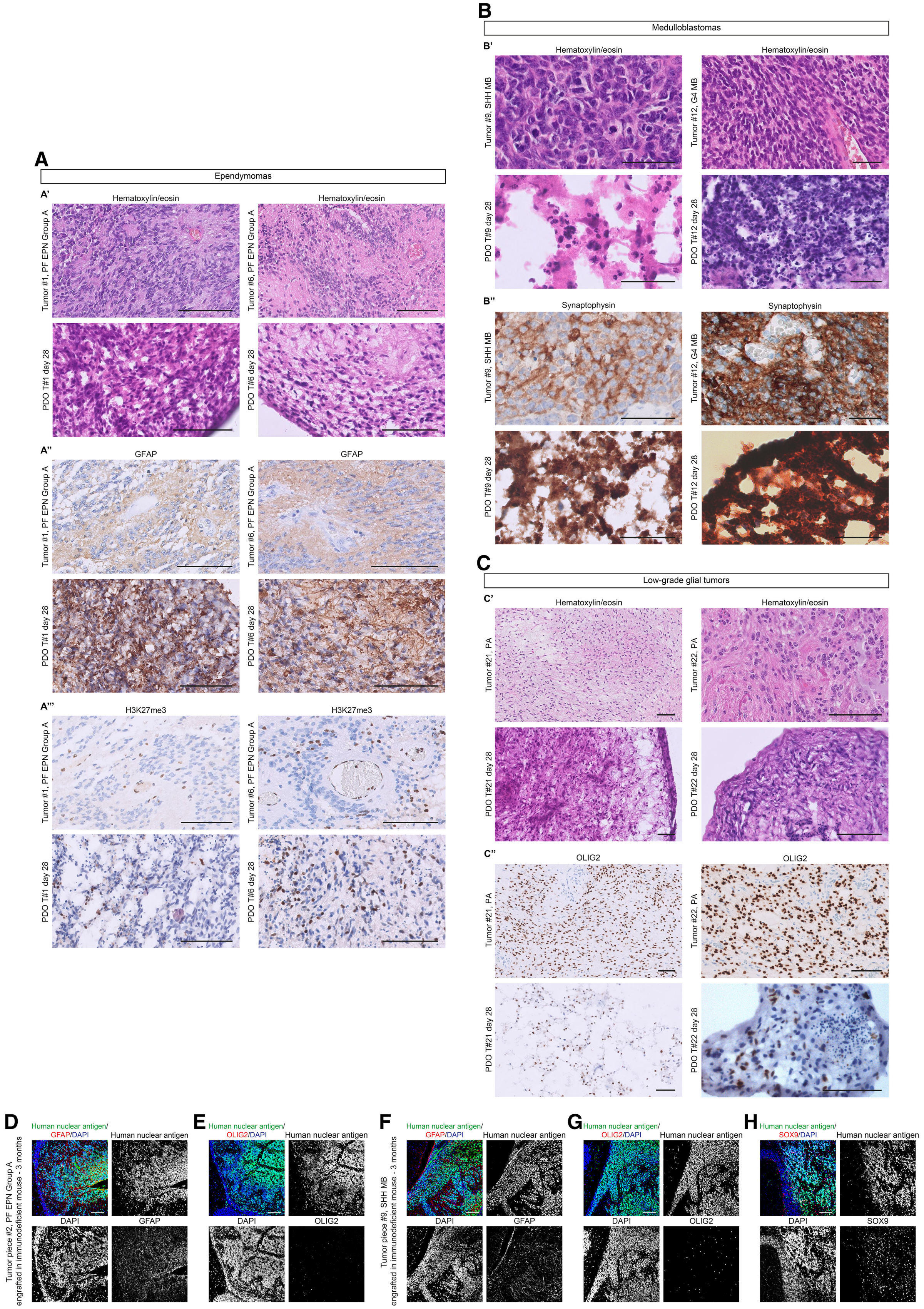
A
Ependymomas
A'
Hematoxylin/eosin
Hematoxylin/eosin
Tumor #1, PF EPN Group A
Tumor #6, PF EPN Group A
PDO T#1 day 28
PDO T#6 day 28
A''
GFAP
GFAP
A'''
H3K27me3
H3K27me3
B
Medulloblastomas
B'
Hematoxylin/eosin
Hematoxylin/eosin
Tumor #9, SHH MB
Tumor #12, G4 MB
PDO T#9 day 28
PDO T#12 day 28
B''
Synaptophysin
Synaptophysin
C
Low-grade glial tumors
C'
Hematoxylin/eosin
Hematoxylin/eosin
Tumor #21, PA
Tumor #22, PA
PDO T#21 day 28
PDO T#22 day 28
C''
OLIG2
OLIG2
D
Human nuclear antigen/
GFAP/DAPI
Human nuclear antigen
DAPI
GFAP
Tumor piece #2, PF EPN Group A
engrafted in immunodeficient mouse - 3 months
E
Human nuclear antigen/
OLIG2/DAPI
Human nuclear antigen
DAPI
OLIG2
F
Human nuclear antigen/
GFAP/DAPI
Human nuclear antigen
DAPI
GFAP
Tumor piece #9, SHH MB
engrafted in immunodeficient mouse - 3 months
G
Human nuclear antigen/
OLIG2/DAPI
Human nuclear antigen
DAPI
OLIG2
H
Human nuclear antigen/
SOX9/DAPI
Human nuclear antigen
DAPI
SOX9

Figure EV4.

Figure EV5. scRNA-seq data analysis of primary tumor #2 and matching PDOs samples describes EPN PFA-specific intratumoral heterogeneity, recapitulated in the PDOs model.

A UMAP dimensionality reduction plot showing the cluster distribution of cells obtained from tumor and PDO samples.
B UMAP plot showing the different independent clusters obtained by integrating the malignant cells from “Primary tumor #2,” “PDO Day 14,” and “PDO Day 28” datasets.
C Expression dotplot representing the key markers identified for each cluster and belonging to cellular and/or functional categories.
D FeaturePlot showing the expression levels of key markers in each cell.
E Stacked barplot representing the relative proportion (expressed in %) of the tumor and PDO cells across the different subclusters.

Data information: scRNA-seq experiment was performed once per primary tumor/matching PDOs.

 © 2023 The Authors

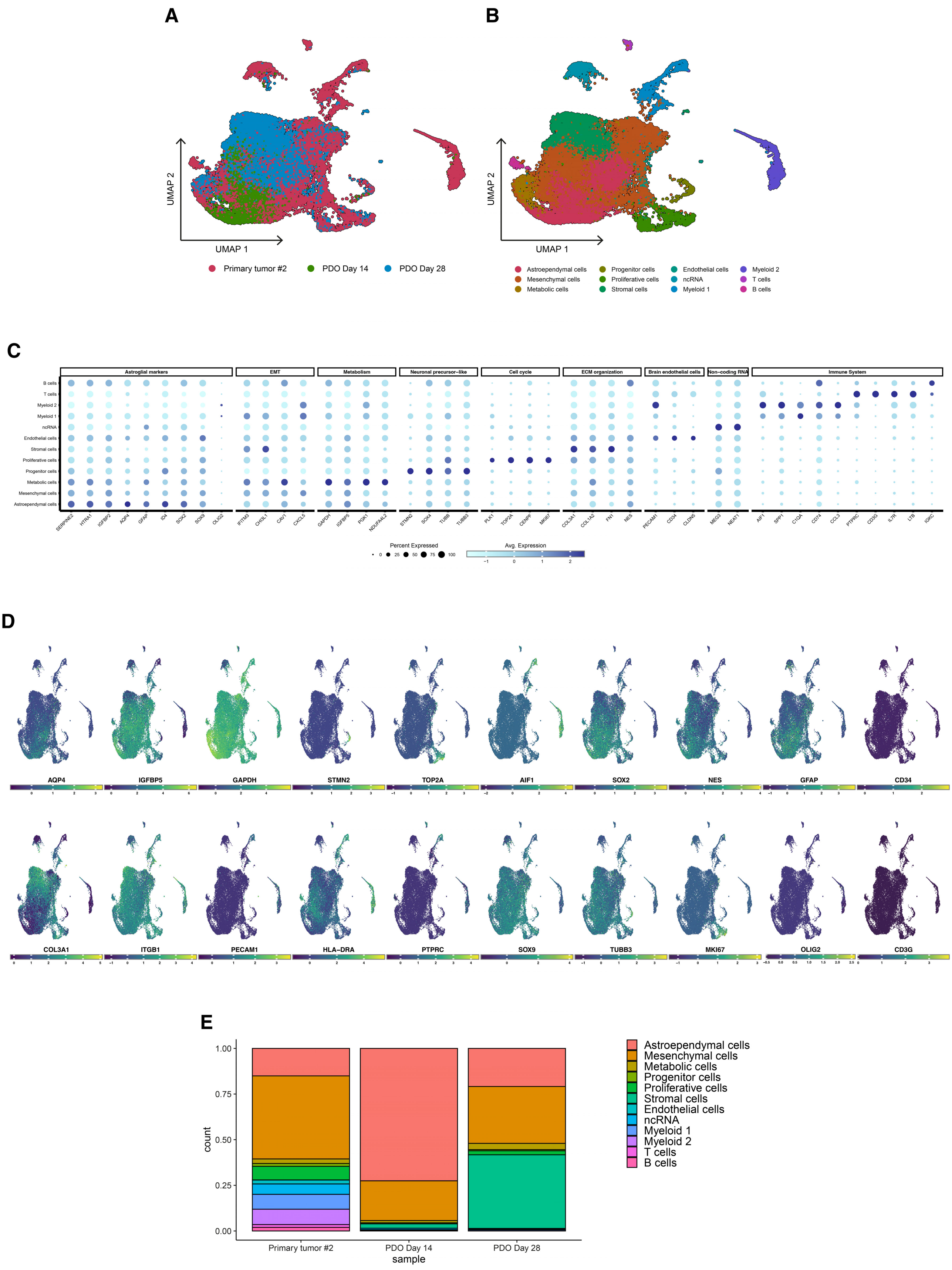
A
B
UMAP 1
UMAP 2
Primary tumor #2
PDO Day 14
PDO Day 28
Astroependymal cells
Mesenchymal cells
Metabolic cells
Progenitor cells
Proliferative cells
Stromal cells
Endothelial cells
ncRNA
Myeloid 1
Myeloid 2
T cells
B cells
C
Astroglial markers
EMT
Metabolism
Neuronal precursor-like
Cell cycle
ECM organization
Brain endothelial cells
Non-coding RNA
Immune System
Percent Expressed
Avg. Expression
D
AQP4
IGFBP5
GAPDH
STMN2
TOP2A
AIF1
SOX2
NES
GFAP
CD34
COL3A1
ITGB1
PECAM1
HLA-DRA
PTPRC
SOX9
TUBB3
MKI67
OLIG2
CD3G
E
count
sample
Primary tumor #2
PDO Day 14
PDO Day 28
1.00
0.75
0.50
0.25
0.00

Figure EV5.
