# Supplementary figures and images for: Patient‐ and xenograft‐derived organoids recapitulate pediatric brain tumor features and patient treatments (part 2 of 4)
Source: EMBO Mol Med. 2023 Nov 30;15(12):e18199. doi: 10.15252/emmm.202318199 (PMC10701620; doi:10.15252/emmm.202318199)

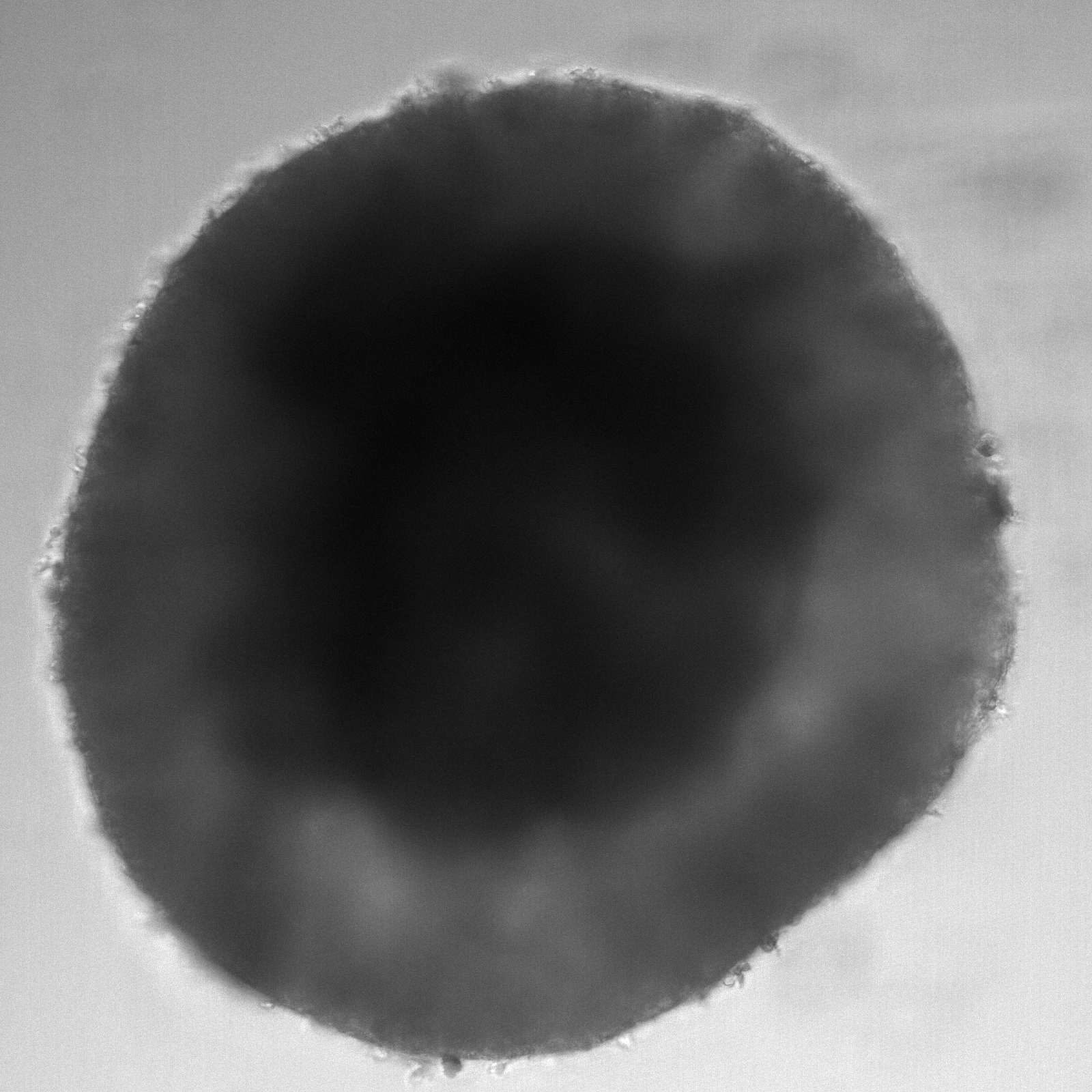

Supplement: Supplementary file 6 — Source Data for Figure 1 [file EMMM-15-e18199-s012.zip › Figure_1F,G,H/1F/Tumor_#21_D21.tif]

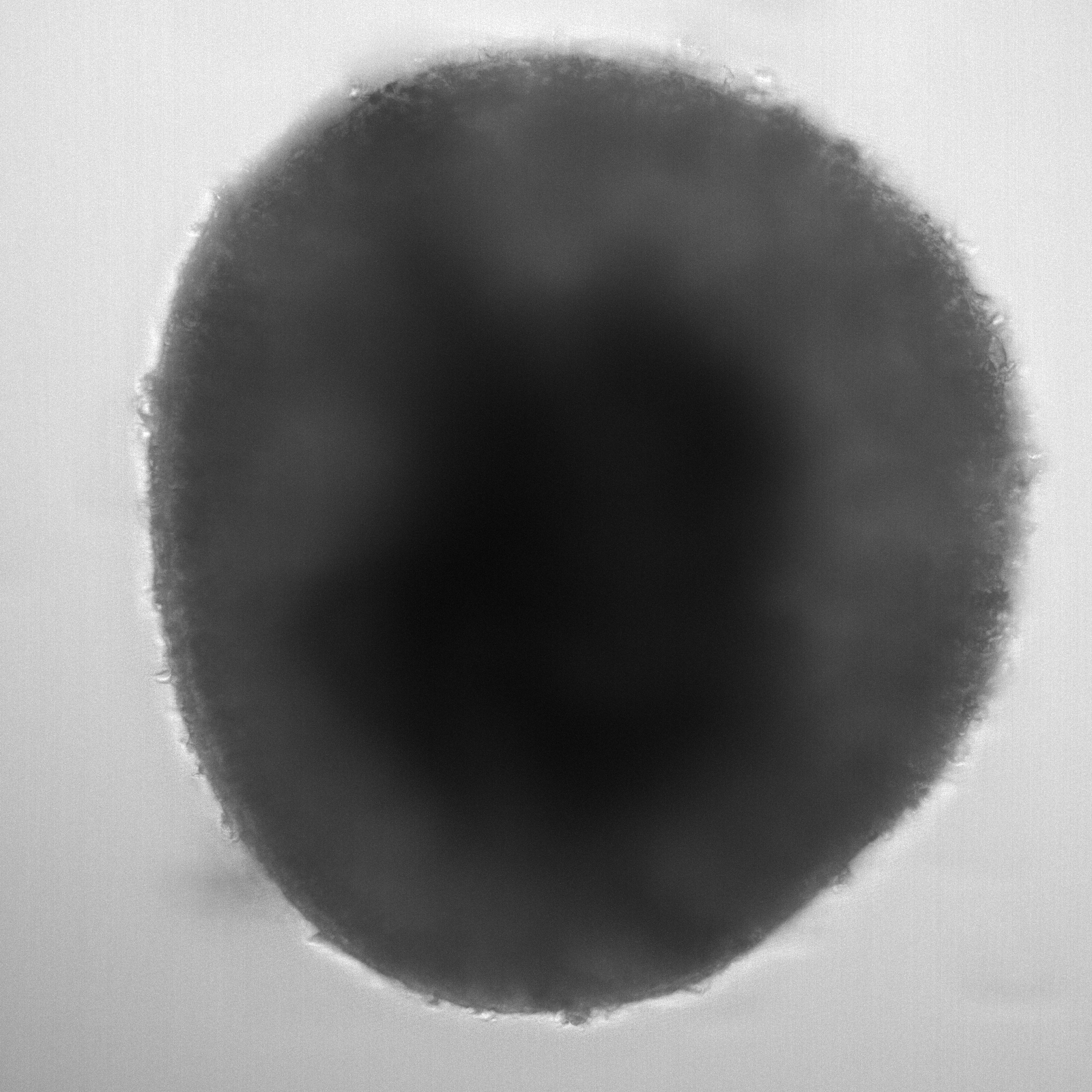

Supplement: Supplementary file 6 — Source Data for Figure 1 [file EMMM-15-e18199-s012.zip › Figure_1F,G,H/1F/Tumor_#21_D28.tif]

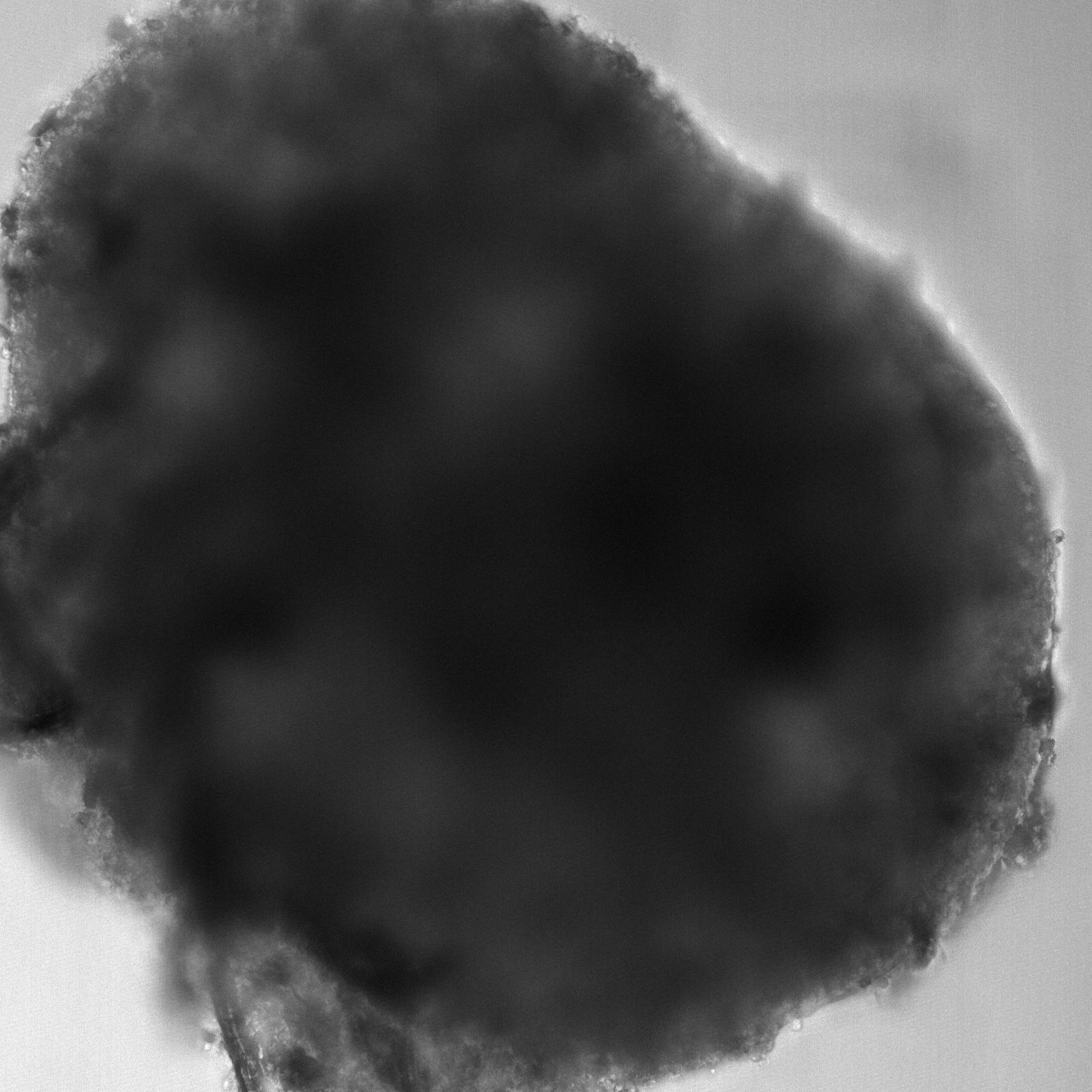

Supplement: Supplementary file 6 — Source Data for Figure 1 [file EMMM-15-e18199-s012.zip › Figure_1F,G,H/1F/Tumor_#21_D7.tif]

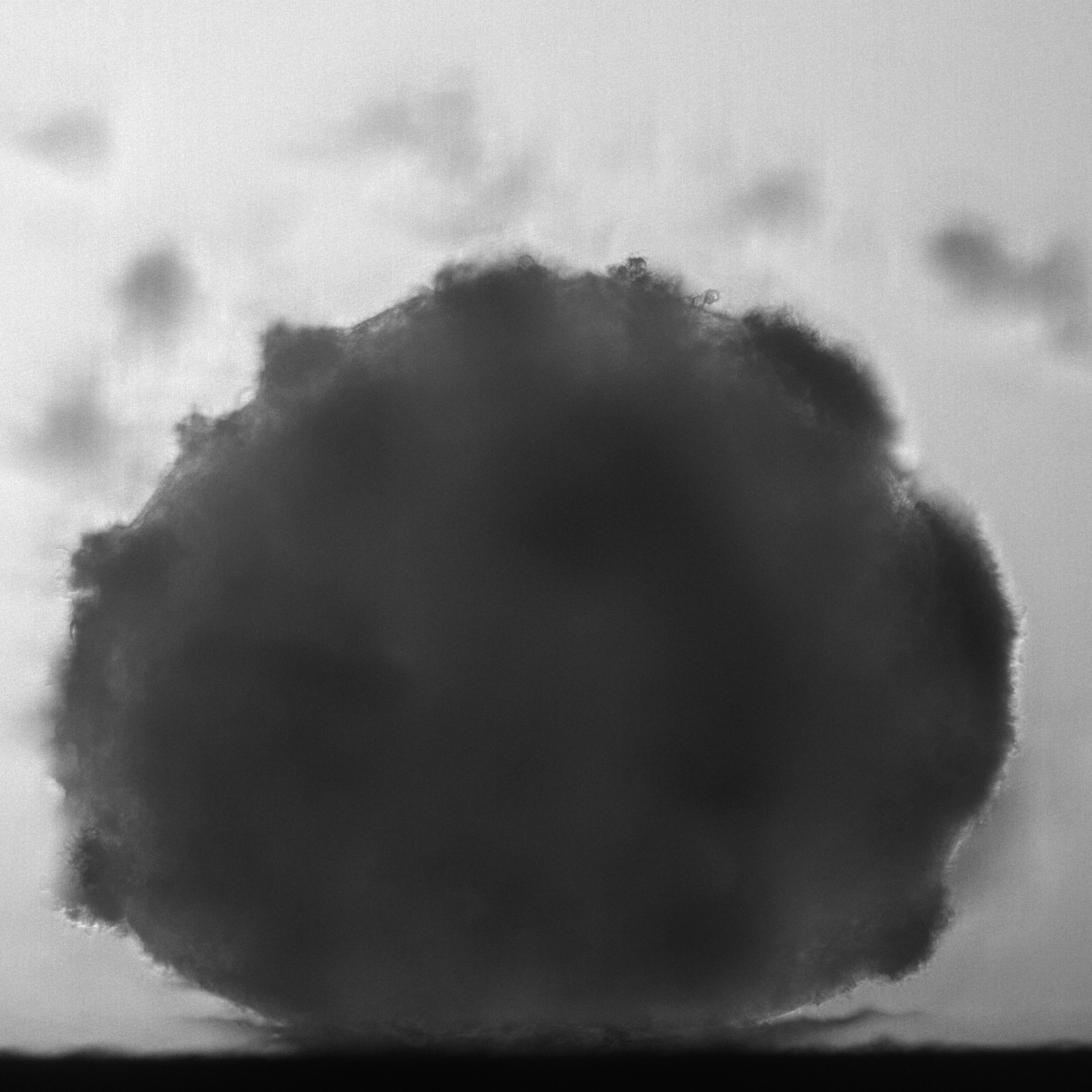

Supplement: Supplementary file 6 — Source Data for Figure 1 [file EMMM-15-e18199-s012.zip › Figure_1F,G,H/1F/Tumor_#22_D14.tif]

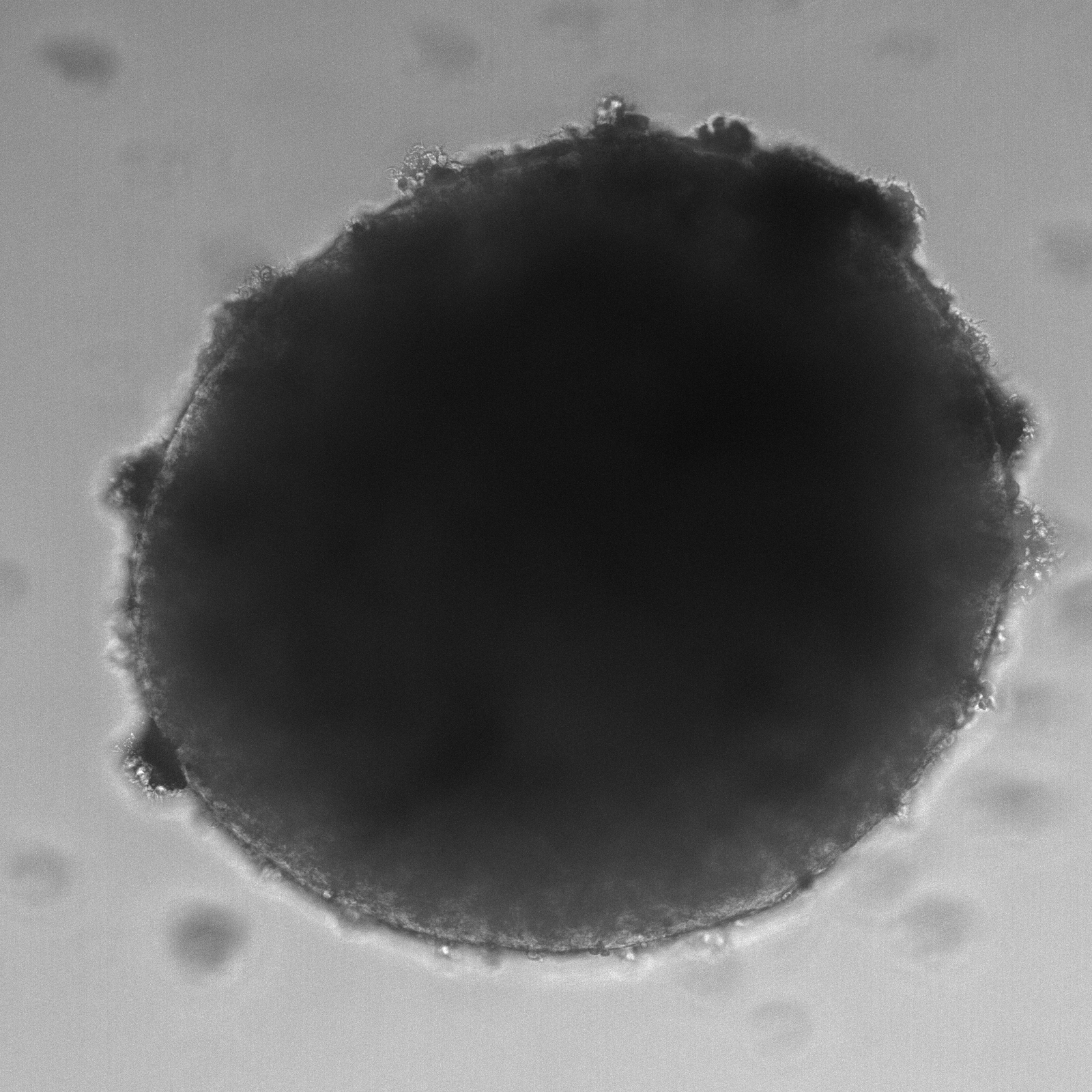

Supplement: Supplementary file 6 — Source Data for Figure 1 [file EMMM-15-e18199-s012.zip › Figure_1F,G,H/1F/Tumor_#22_D21.tif]

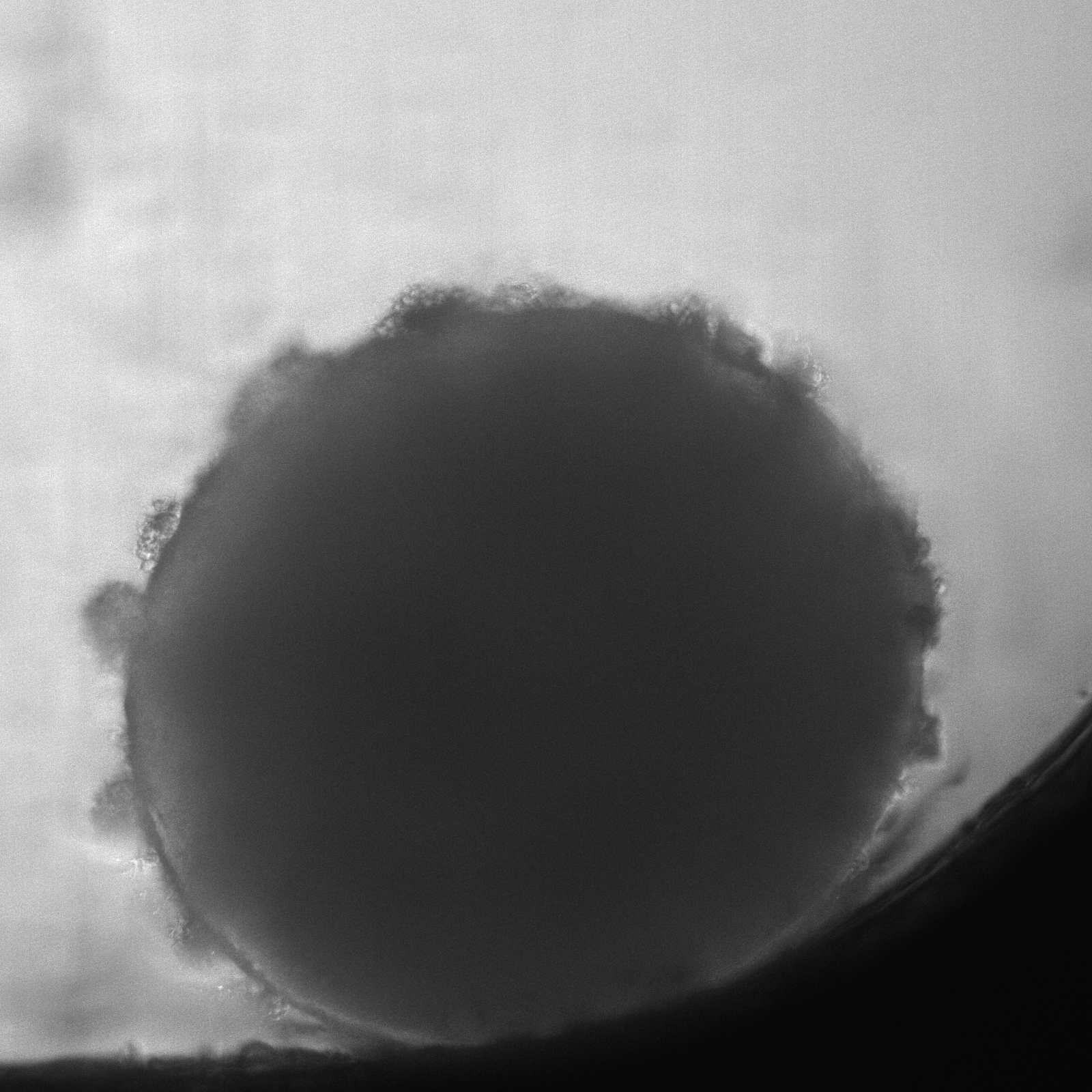

Supplement: Supplementary file 6 — Source Data for Figure 1 [file EMMM-15-e18199-s012.zip › Figure_1F,G,H/1F/Tumor_#22_D28.tif]

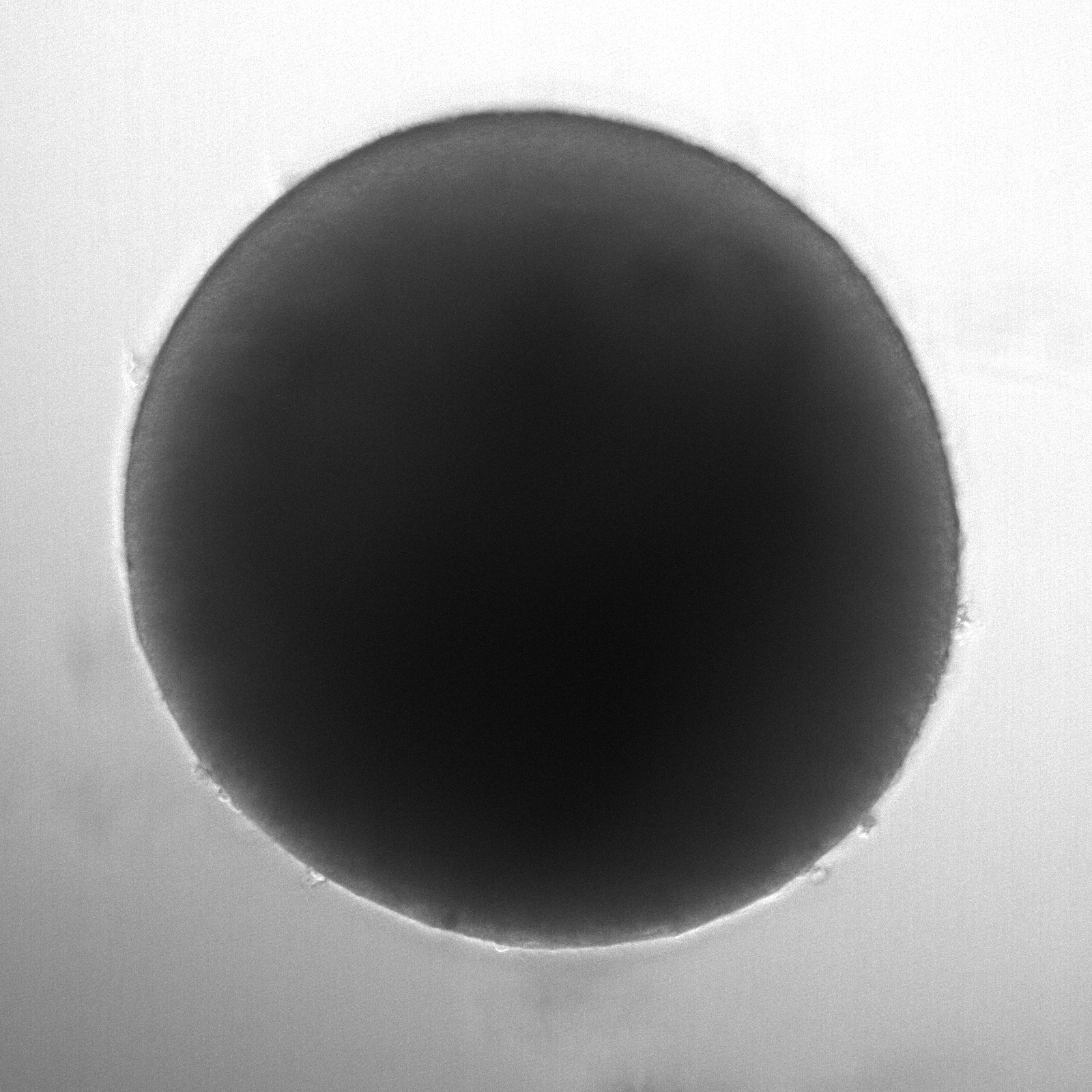

Supplement: Supplementary file 6 — Source Data for Figure 1 [file EMMM-15-e18199-s012.zip › Figure_1F,G,H/1F/Tumor_#22_D42.tif]

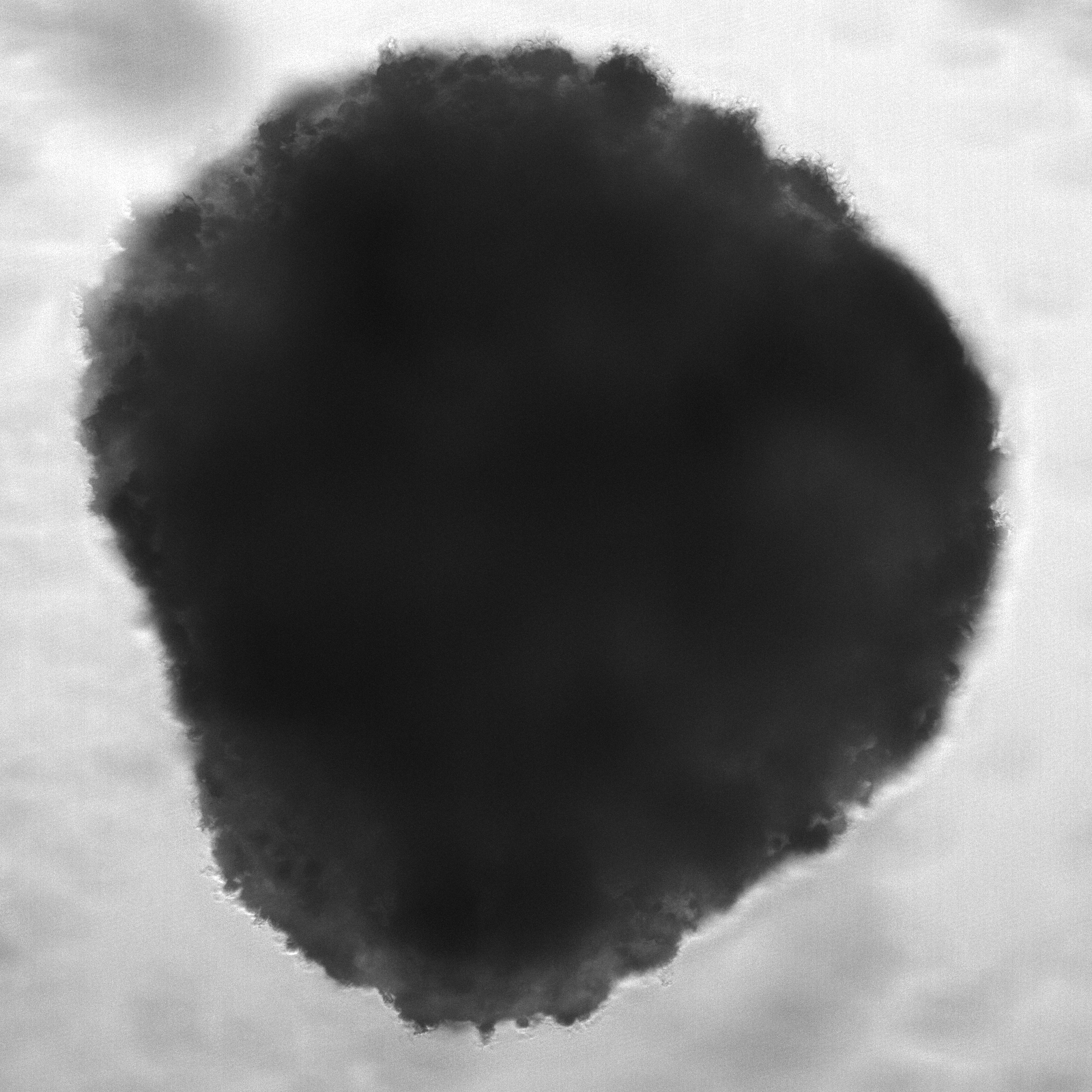

Supplement: Supplementary file 6 — Source Data for Figure 1 [file EMMM-15-e18199-s012.zip › Figure_1F,G,H/1F/Tumor_#22_D7.tif]

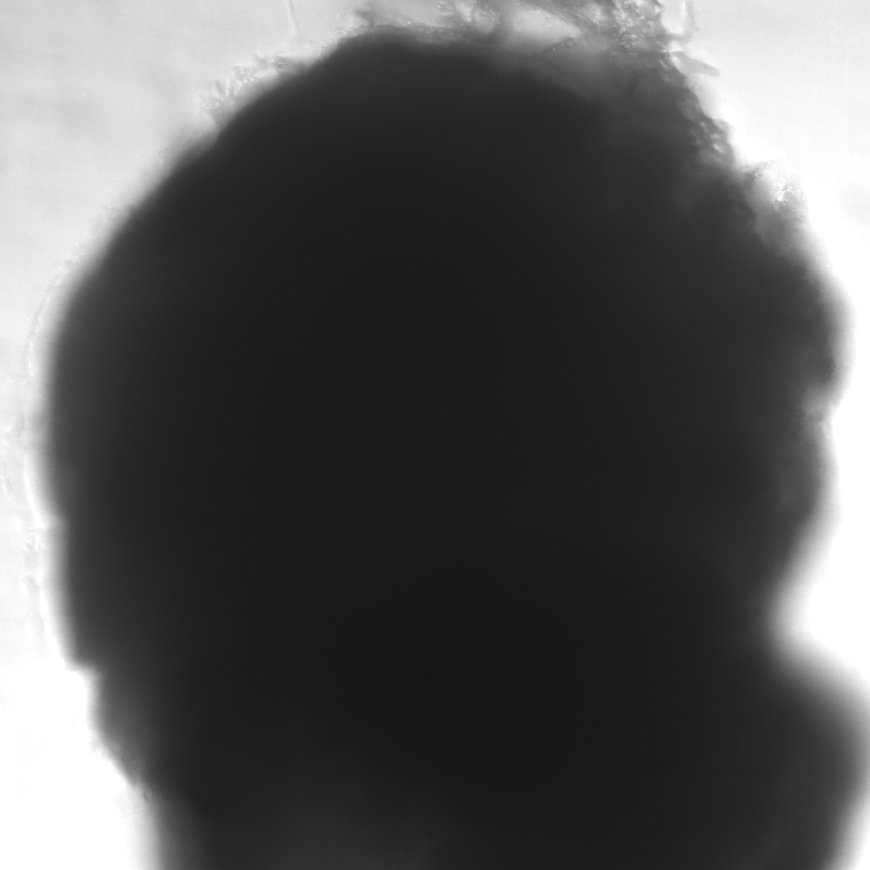

Supplement: Supplementary file 6 — Source Data for Figure 1 [file EMMM-15-e18199-s012.zip › Figure_1F,G,H/1F/Tumor_#23_D29.tif]

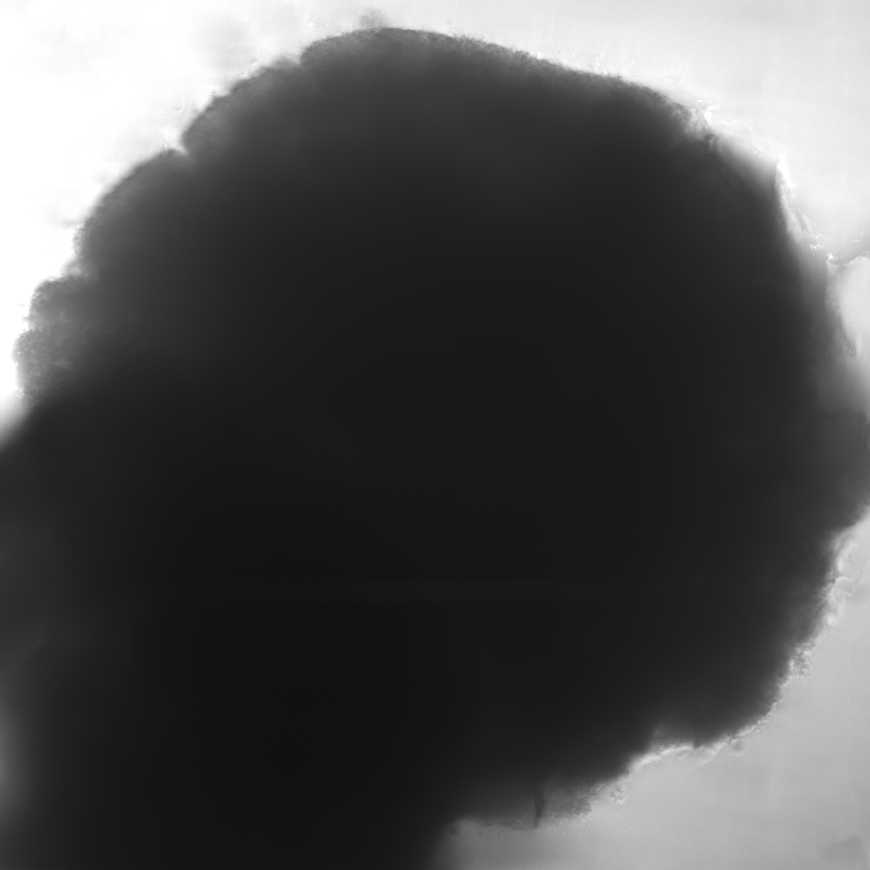

Supplement: Supplementary file 6 — Source Data for Figure 1 [file EMMM-15-e18199-s012.zip › Figure_1F,G,H/1F/Tumor_#23_D35.tif]

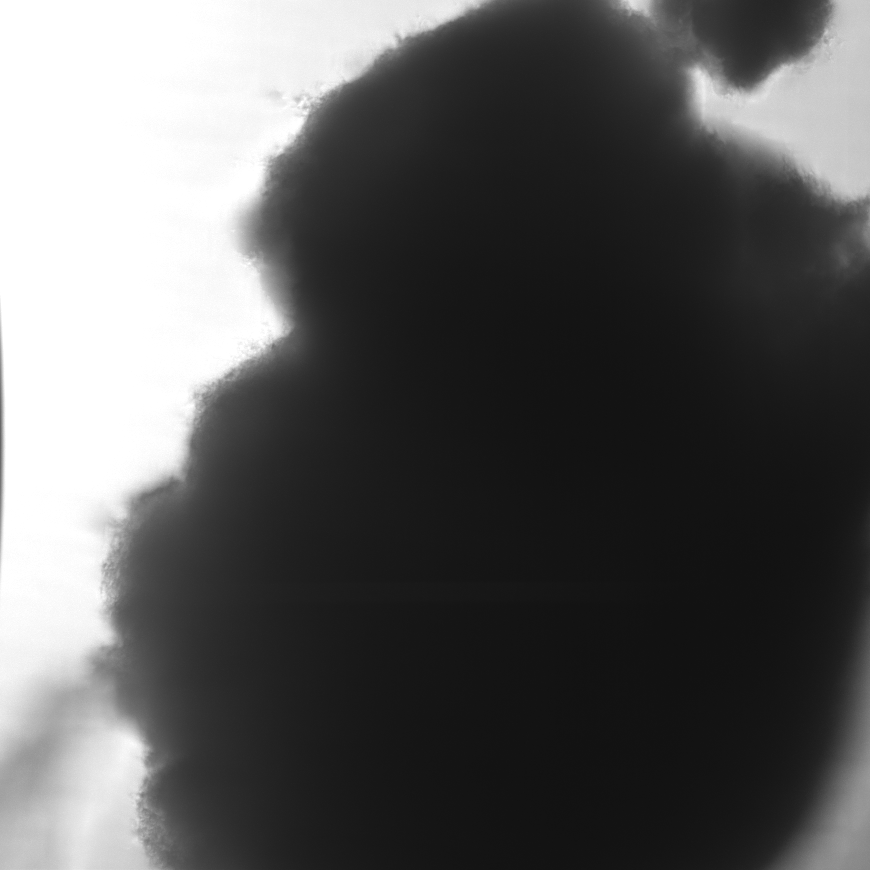

Supplement: Supplementary file 6 — Source Data for Figure 1 [file EMMM-15-e18199-s012.zip › Figure_1F,G,H/1F/Tumor_#23_D43.tif]

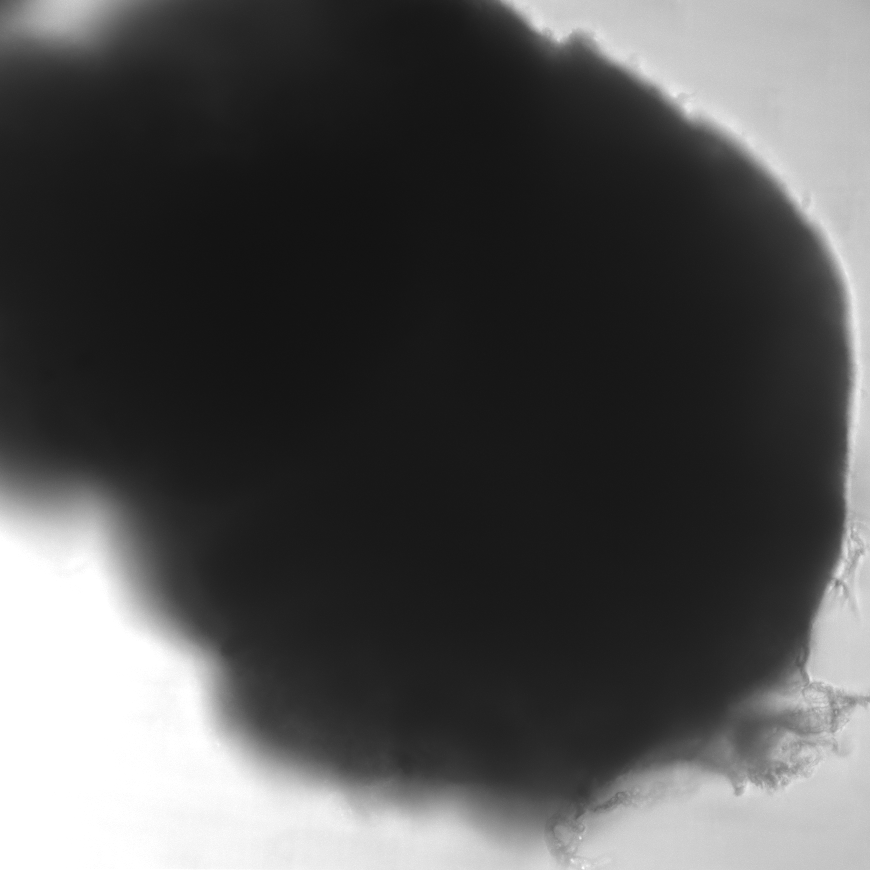

Supplement: Supplementary file 6 — Source Data for Figure 1 [file EMMM-15-e18199-s012.zip › Figure_1F,G,H/1F/Tumor_#23_D49.tif]

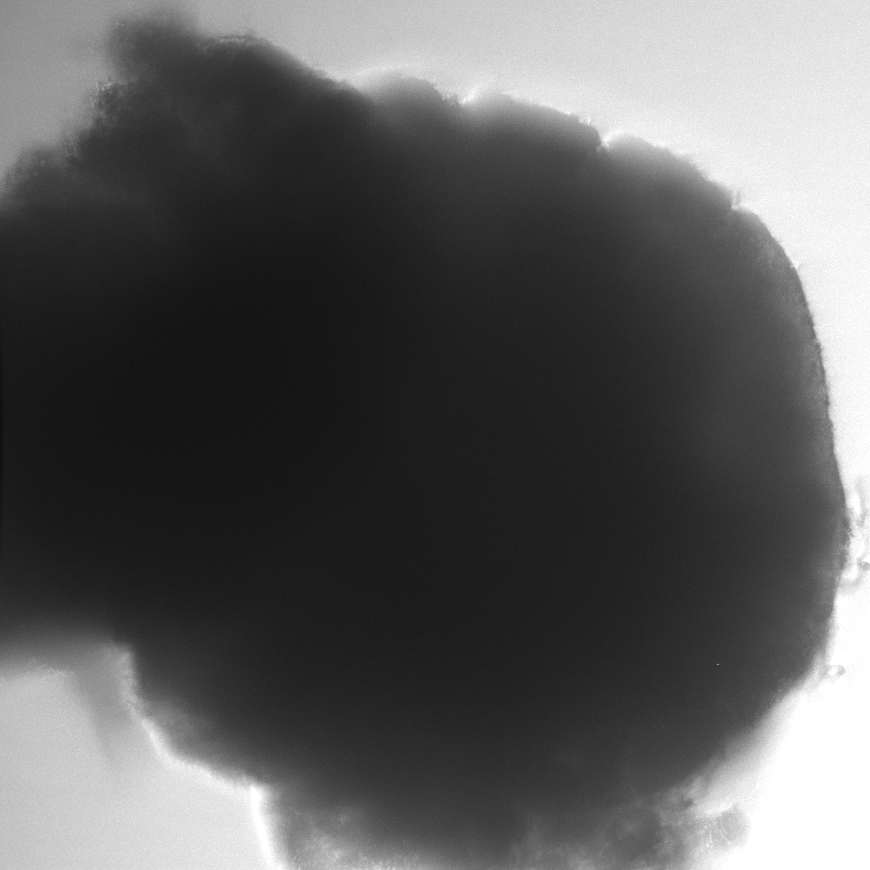

Supplement: Supplementary file 6 — Source Data for Figure 1 [file EMMM-15-e18199-s012.zip › Figure_1F,G,H/1F/Tumor_#23_D61.tif]

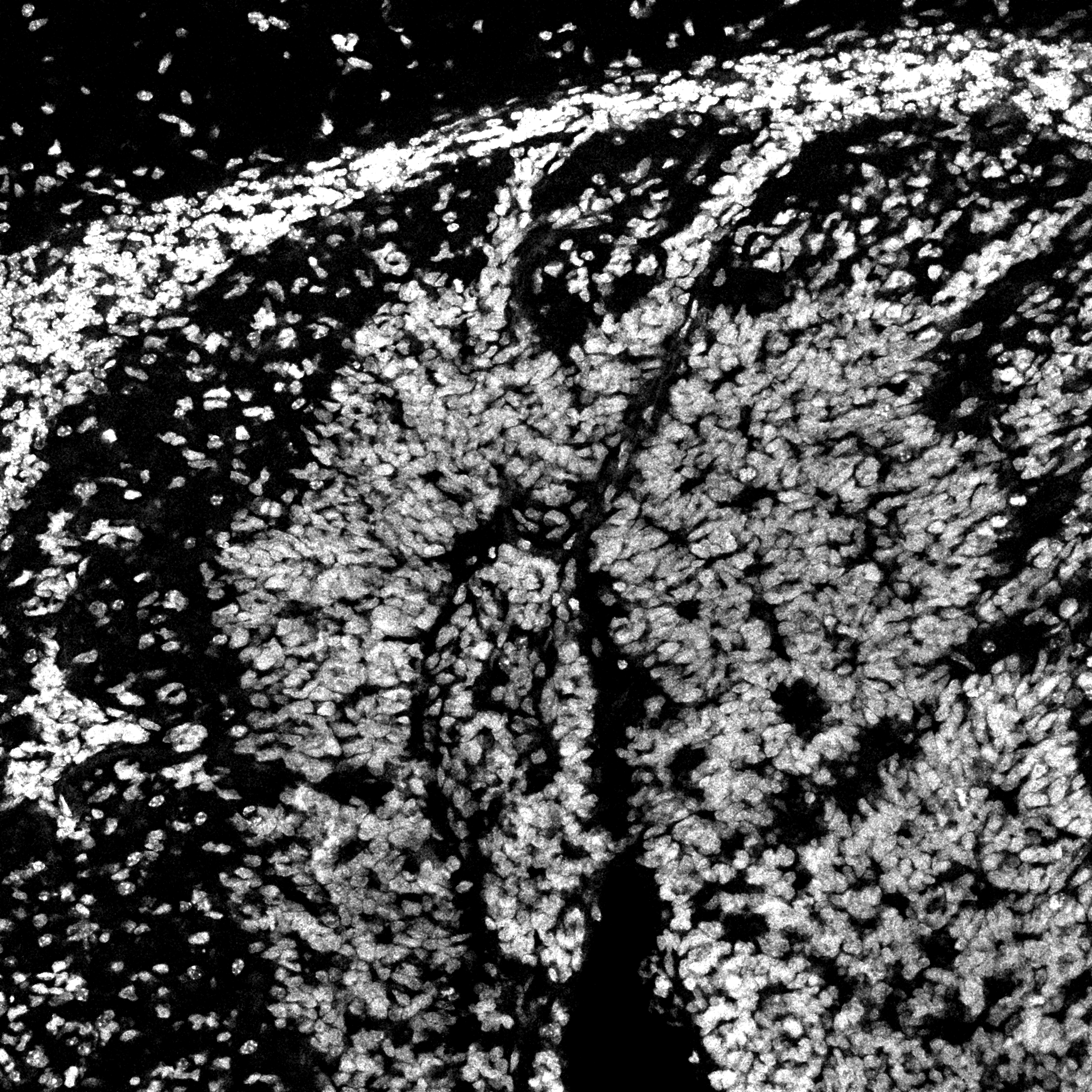

Supplement: Supplementary file 6 — Source Data for Figure 1 [file EMMM-15-e18199-s012.zip › Figure_1F,G,H/1G/G''_Ki67,_HumAnt_DAPI.tif]

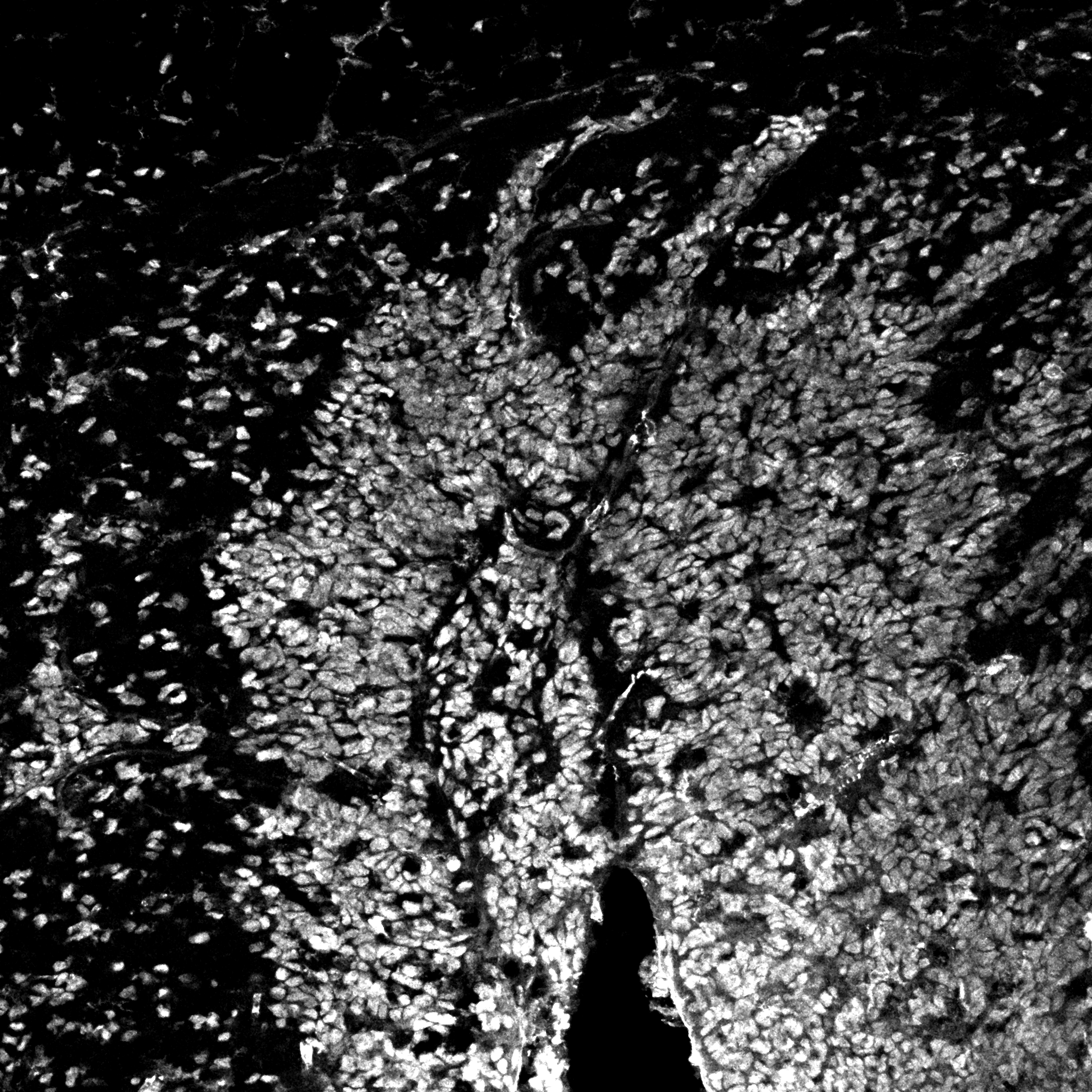

Supplement: Supplementary file 6 — Source Data for Figure 1 [file EMMM-15-e18199-s012.zip › Figure_1F,G,H/1G/G''_Ki67,_HumAnt_HumAnt.tif]

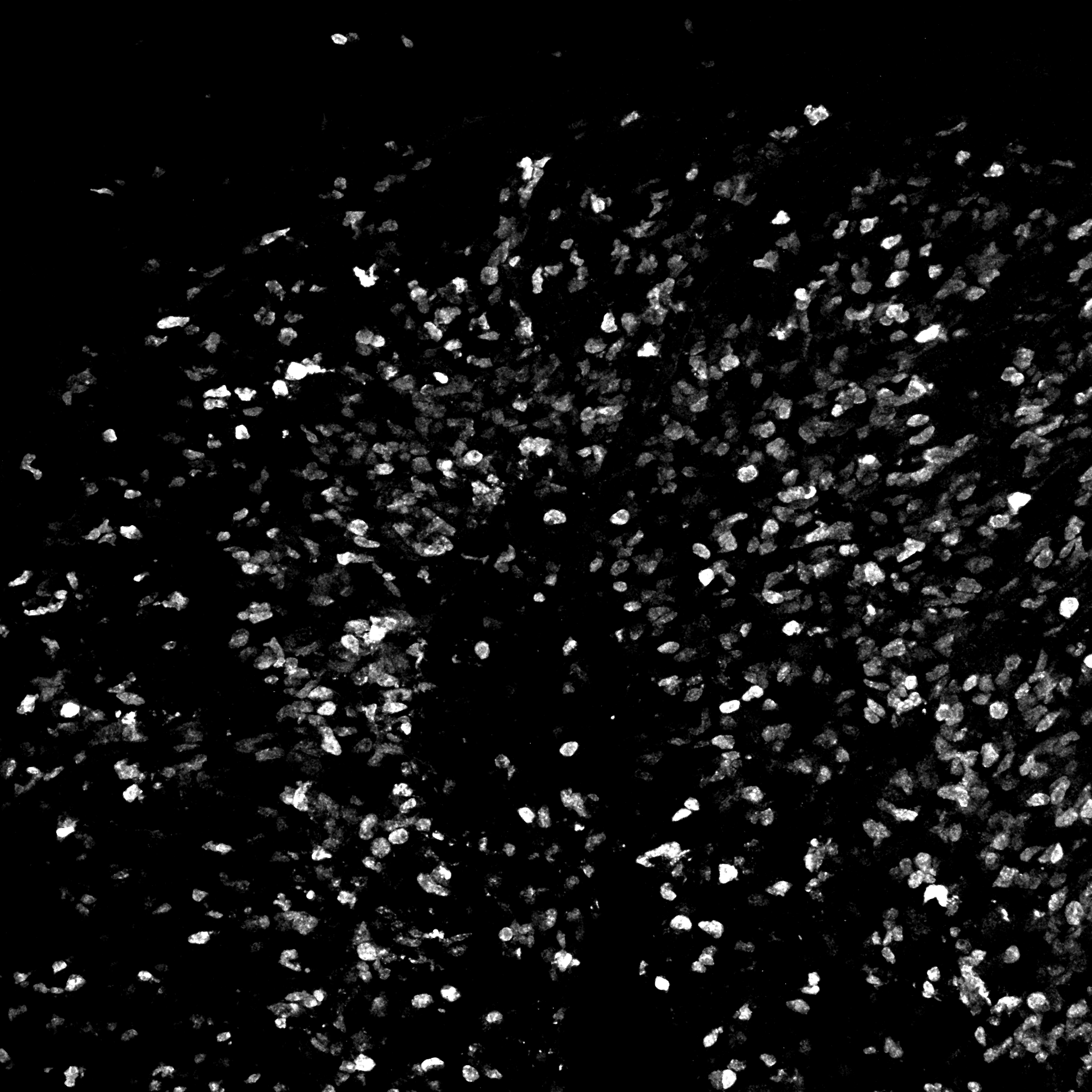

Supplement: Supplementary file 6 — Source Data for Figure 1 [file EMMM-15-e18199-s012.zip › Figure_1F,G,H/1G/G''_Ki67,_HumAnt_Ki67.tif]

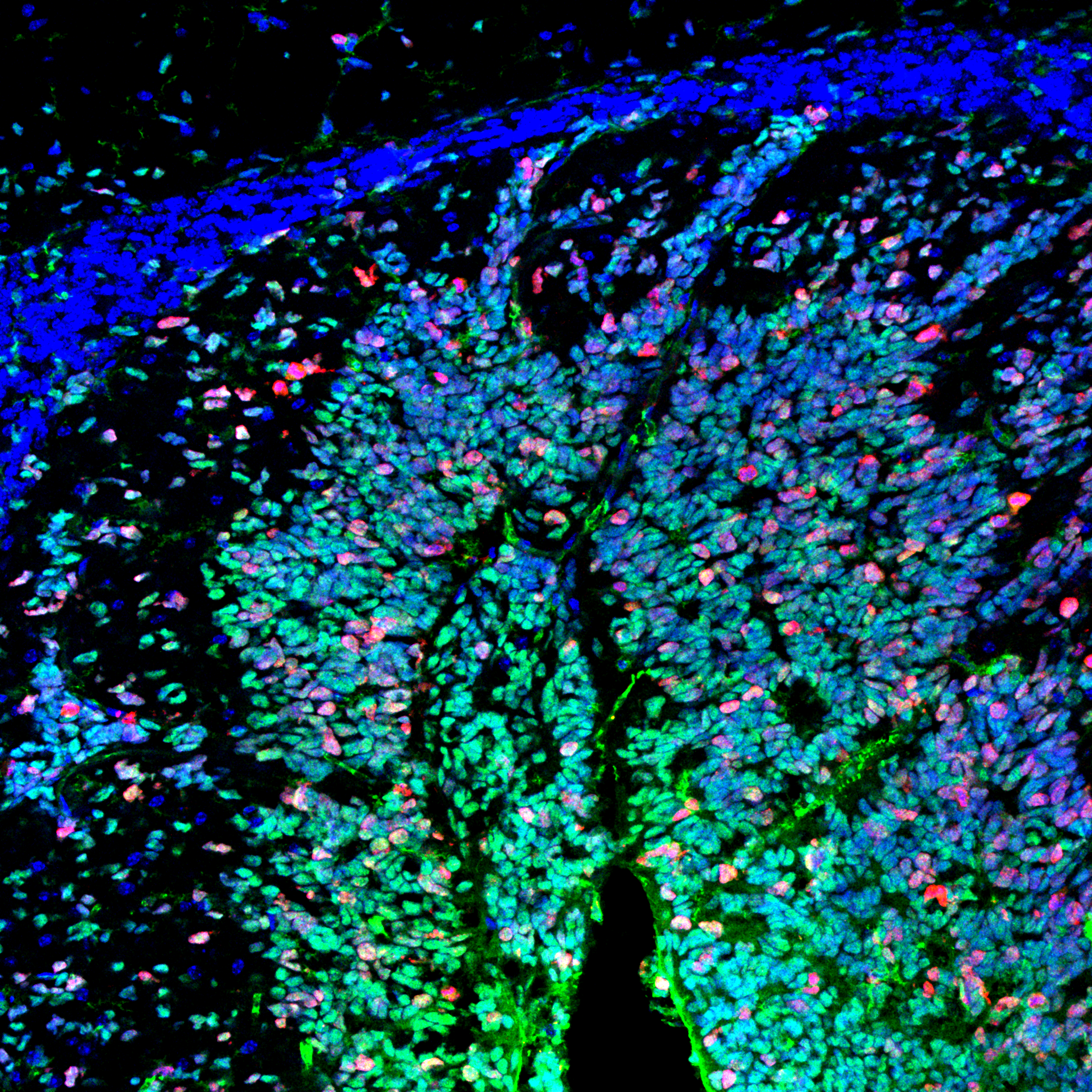

Supplement: Supplementary file 6 — Source Data for Figure 1 [file EMMM-15-e18199-s012.zip › Figure_1F,G,H/1G/G''_Ki67,_HumAnt_merge.tif]

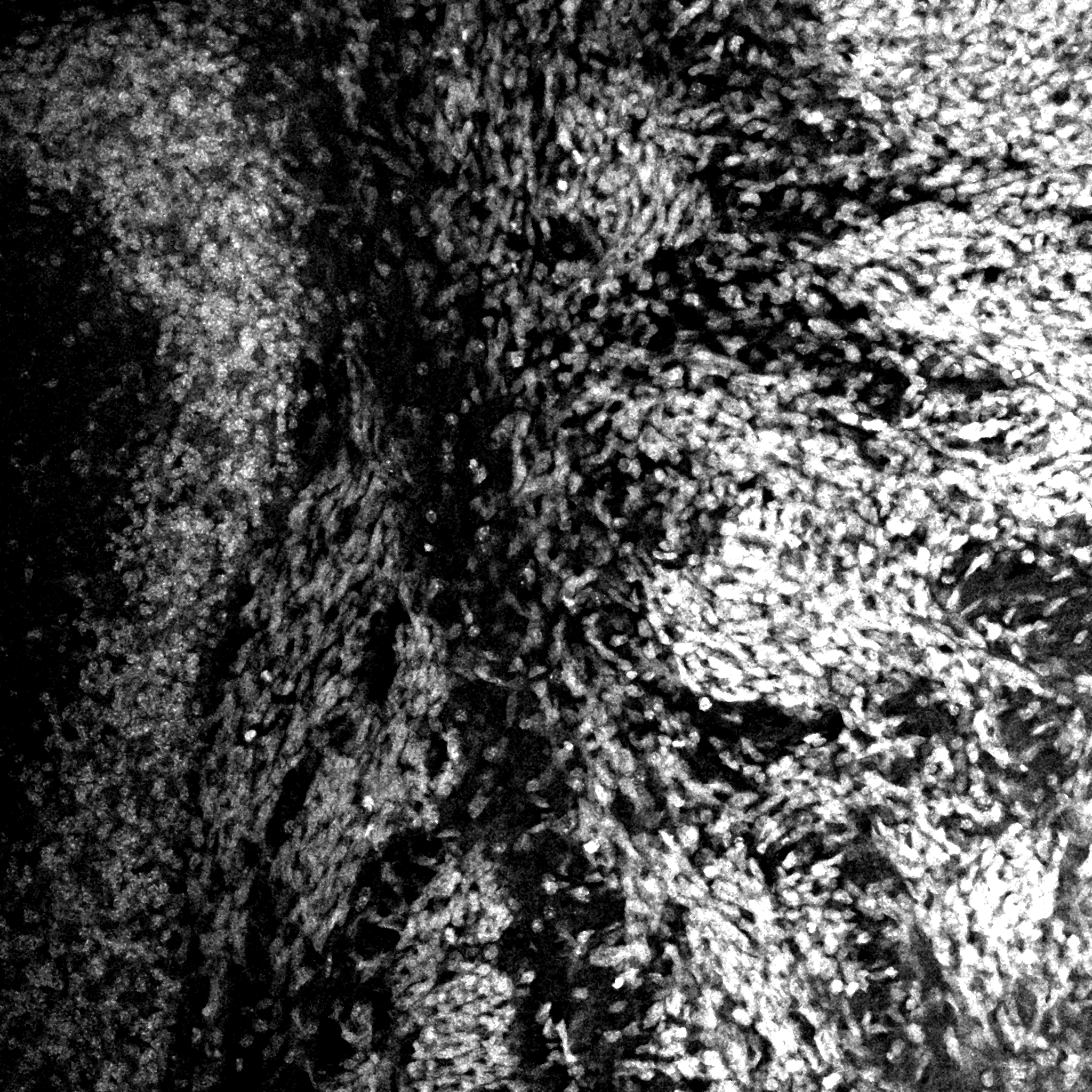

Supplement: Supplementary file 6 — Source Data for Figure 1 [file EMMM-15-e18199-s012.zip › Figure_1F,G,H/1H/H''_Ki67,_HumAnt_DAPI.tif]

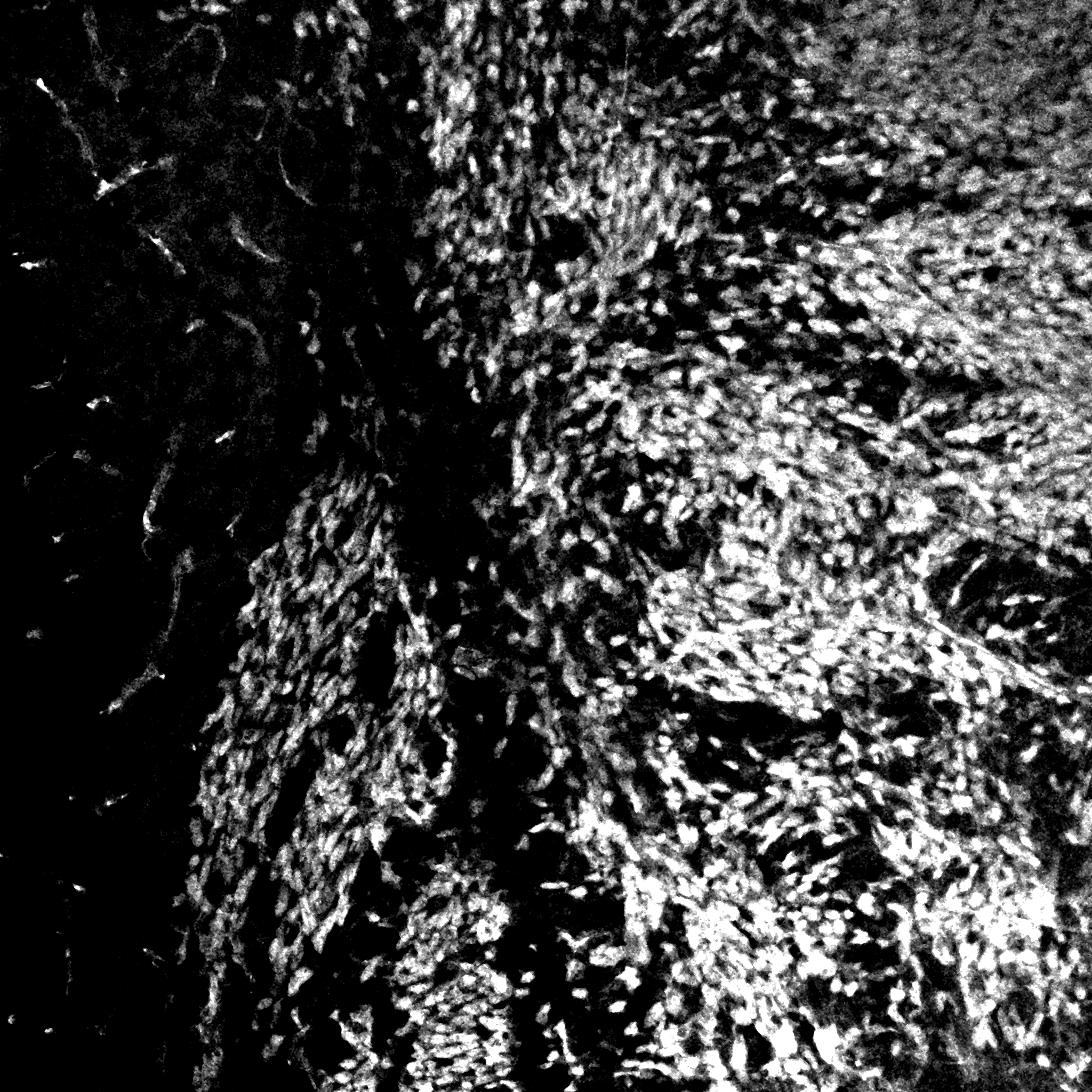

Supplement: Supplementary file 6 — Source Data for Figure 1 [file EMMM-15-e18199-s012.zip › Figure_1F,G,H/1H/H''_Ki67,_HumAnt_HumAnt.tif]

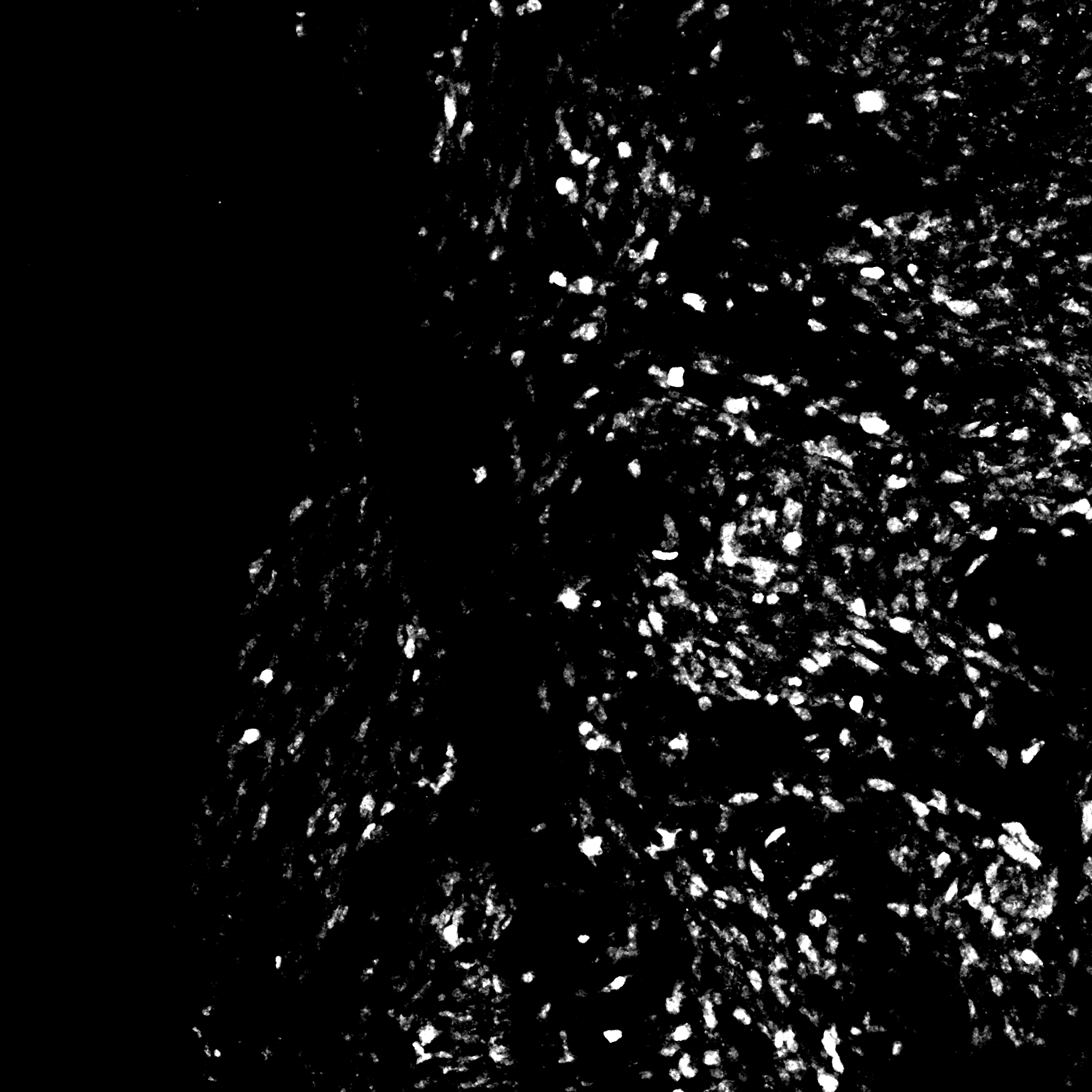

Supplement: Supplementary file 6 — Source Data for Figure 1 [file EMMM-15-e18199-s012.zip › Figure_1F,G,H/1H/H''_Ki67,_HumAnt_Ki67.tif]

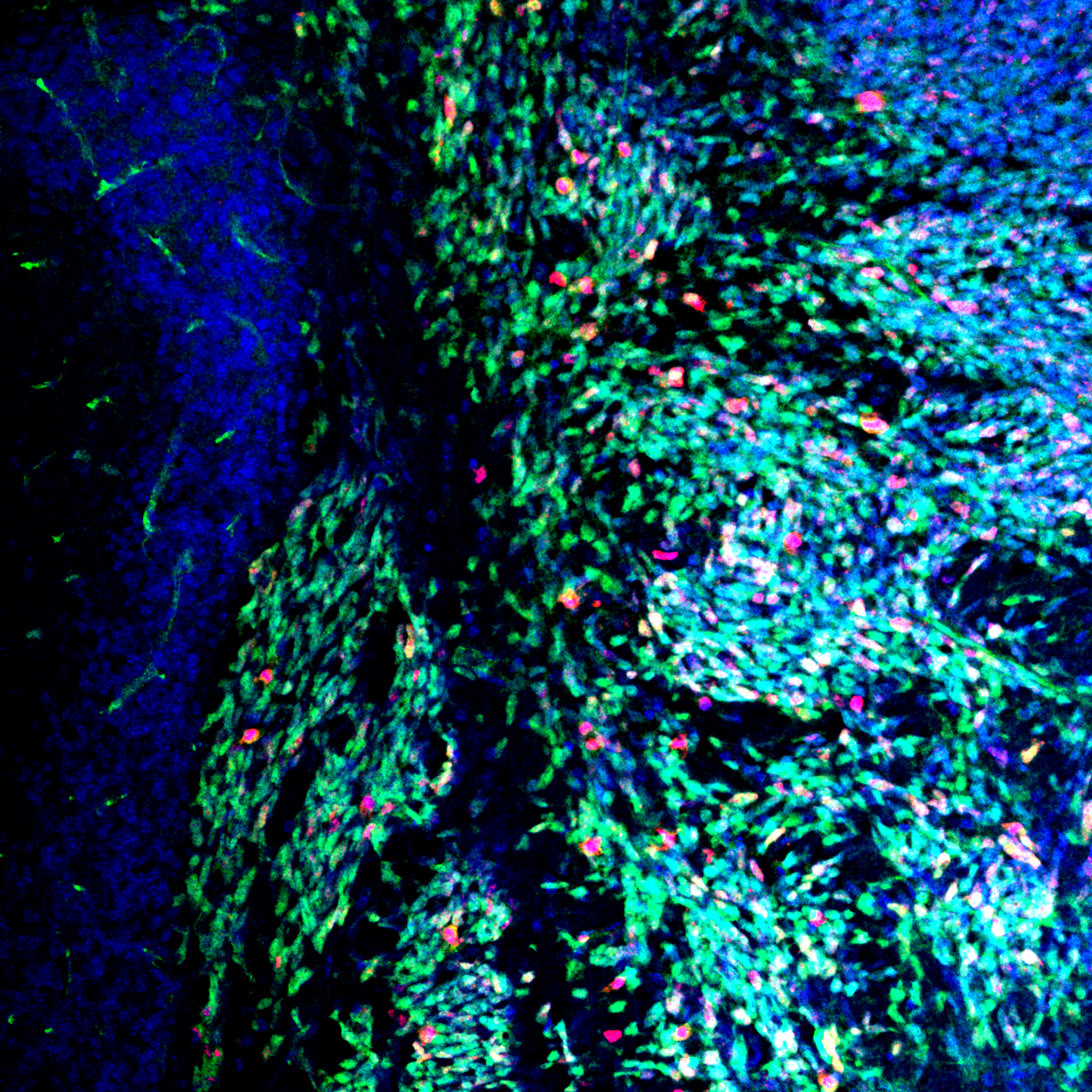

Supplement: Supplementary file 6 — Source Data for Figure 1 [file EMMM-15-e18199-s012.zip › Figure_1F,G,H/1H/H''_Ki67,_HumAnt_merge.tif]

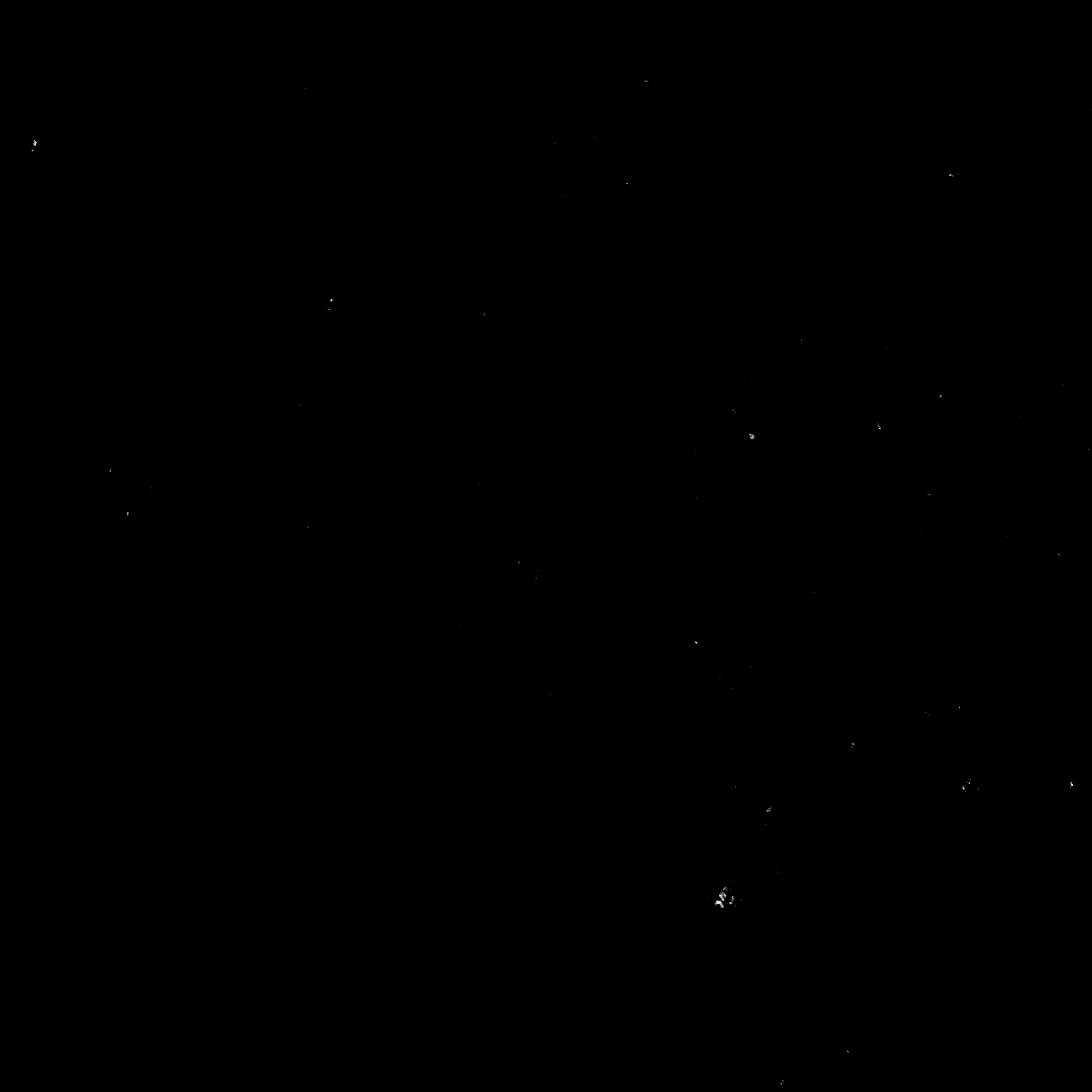

Supplement: Supplementary file 8 — Source Data for Figure 3 [file EMMM-15-e18199-s011.zip › Figure_3/3A/A'_PDO_T#1_D28_CD34,_CD3_CD3.tif]

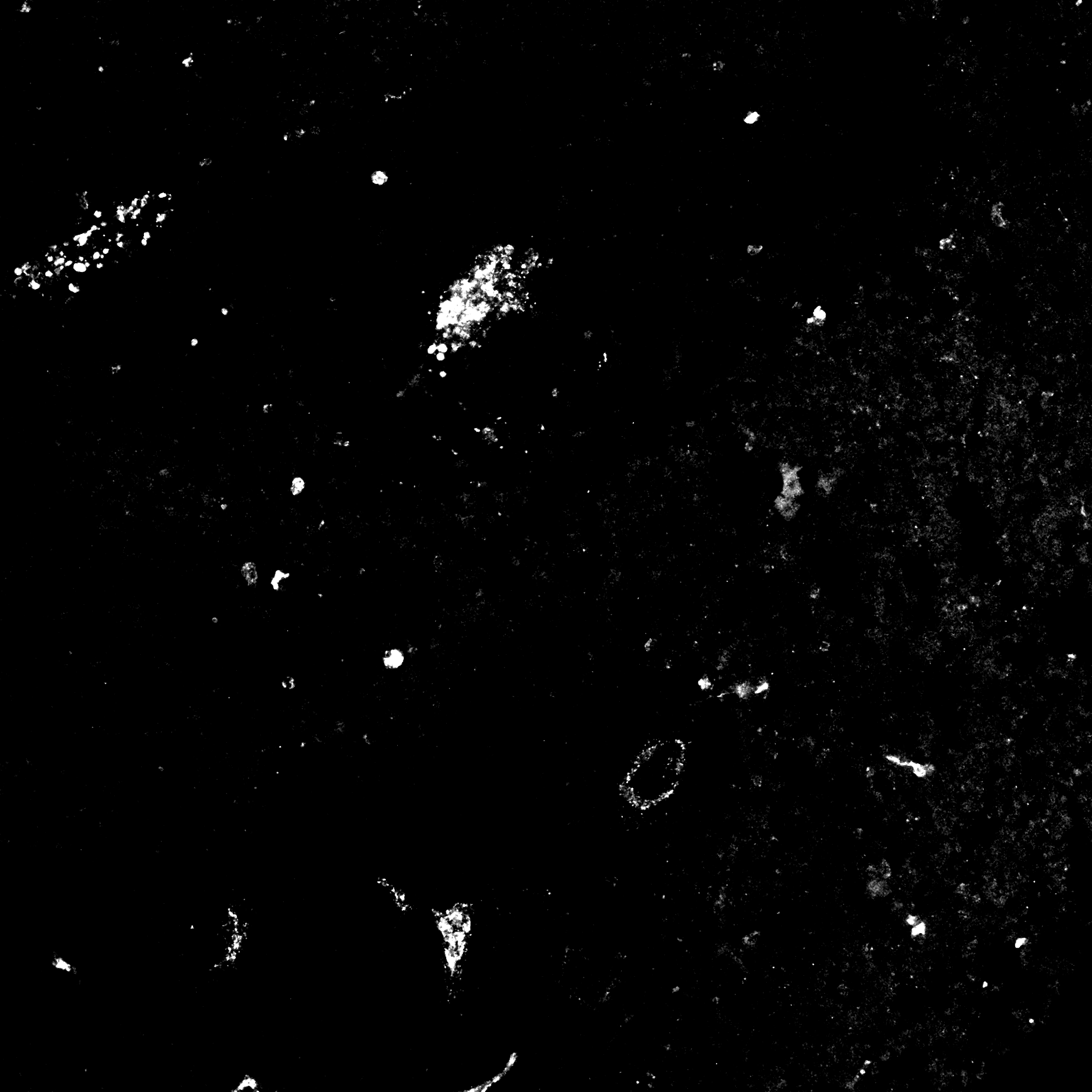

Supplement: Supplementary file 8 — Source Data for Figure 3 [file EMMM-15-e18199-s011.zip › Figure_3/3A/A'_PDO_T#1_D28_CD34,_CD3_CD34.tif]

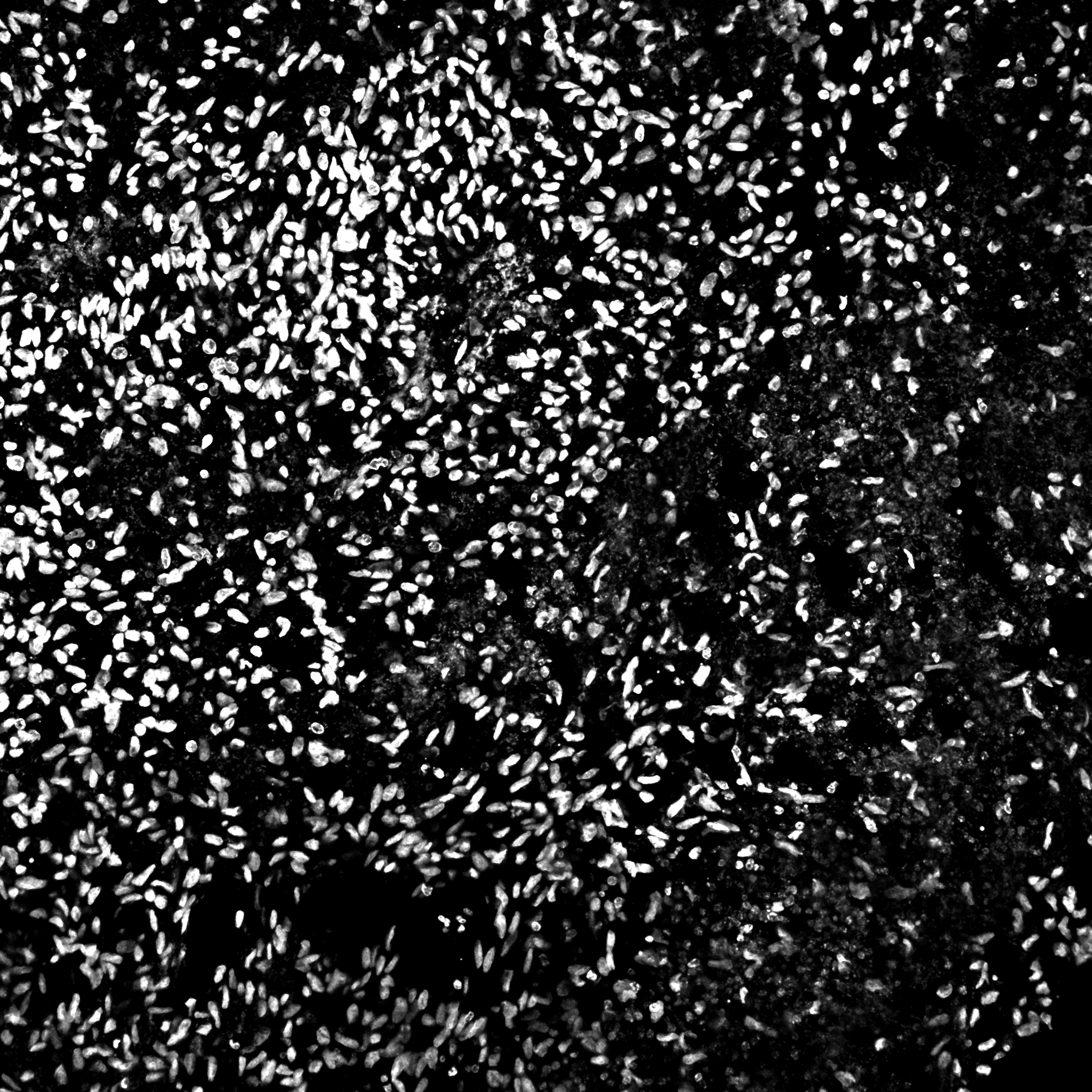

Supplement: Supplementary file 8 — Source Data for Figure 3 [file EMMM-15-e18199-s011.zip › Figure_3/3A/A'_PDO_T#1_D28_CD34,_CD3_DAPI.tif]

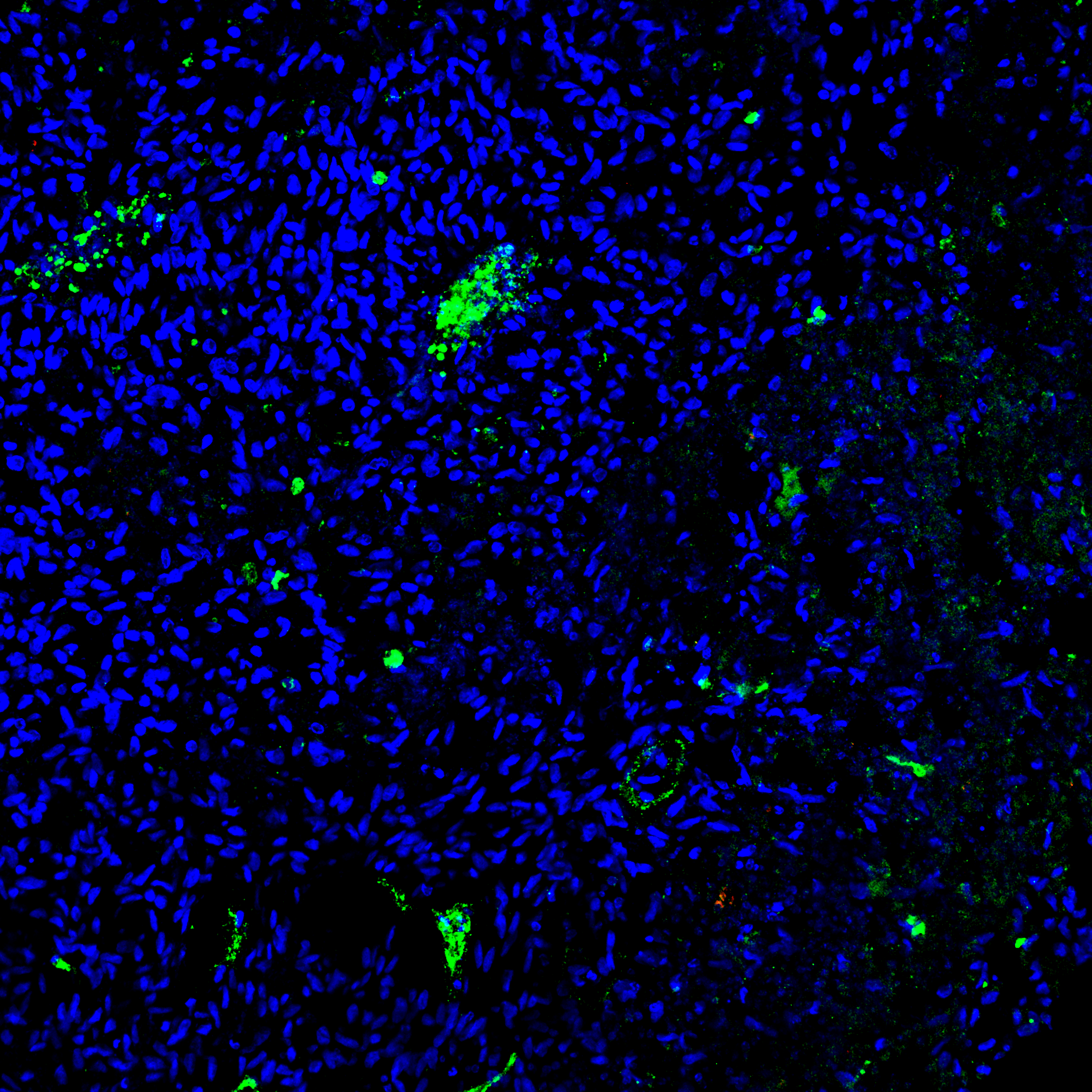

Supplement: Supplementary file 8 — Source Data for Figure 3 [file EMMM-15-e18199-s011.zip › Figure_3/3A/A'_PDO_T#1_D28_CD34,_CD3_merge.tif]

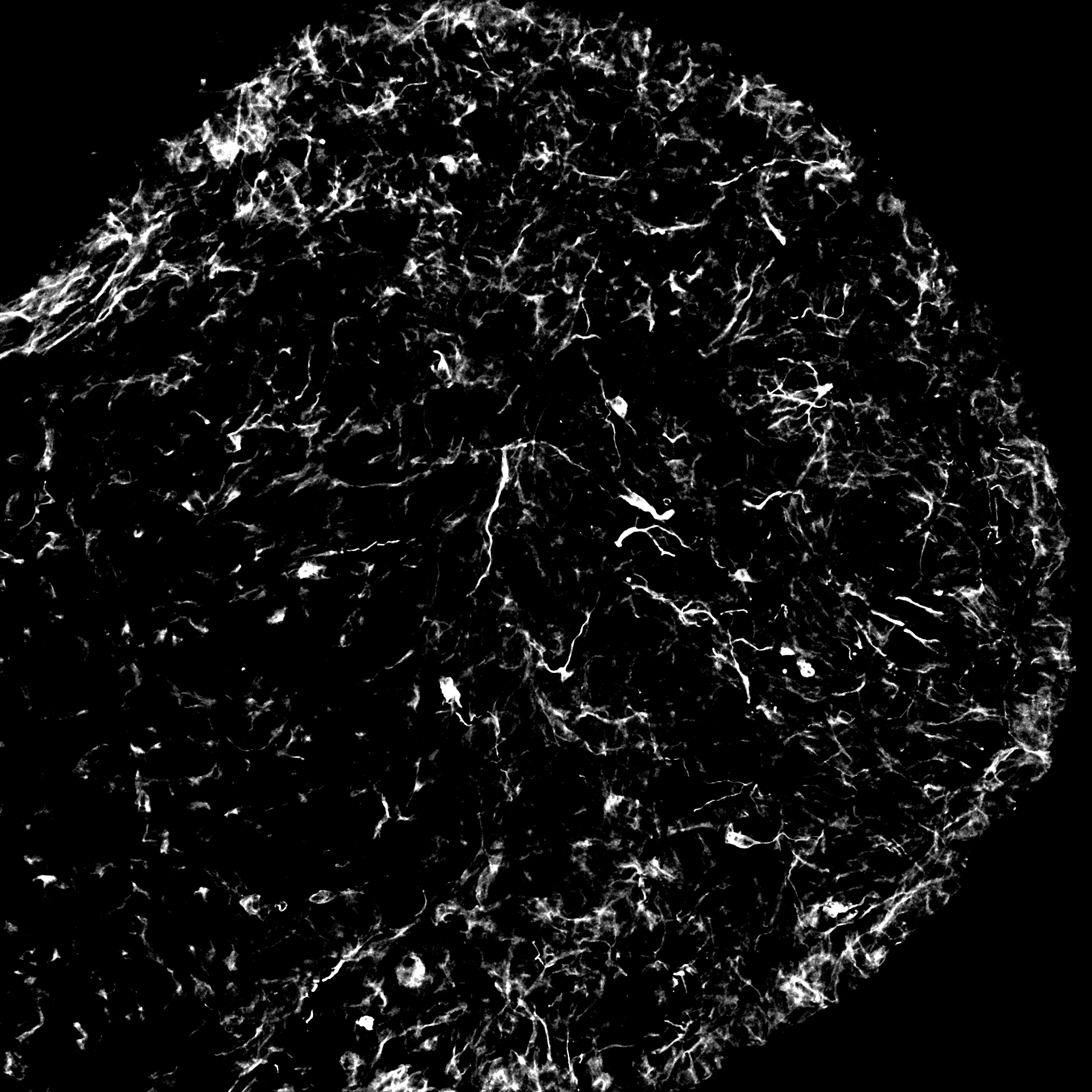

Supplement: Supplementary file 8 — Source Data for Figure 3 [file EMMM-15-e18199-s011.zip › Figure_3/3A/A'_PDO_T#1_D28_GFAP,_B3tubulin_B3tubulin.tif]

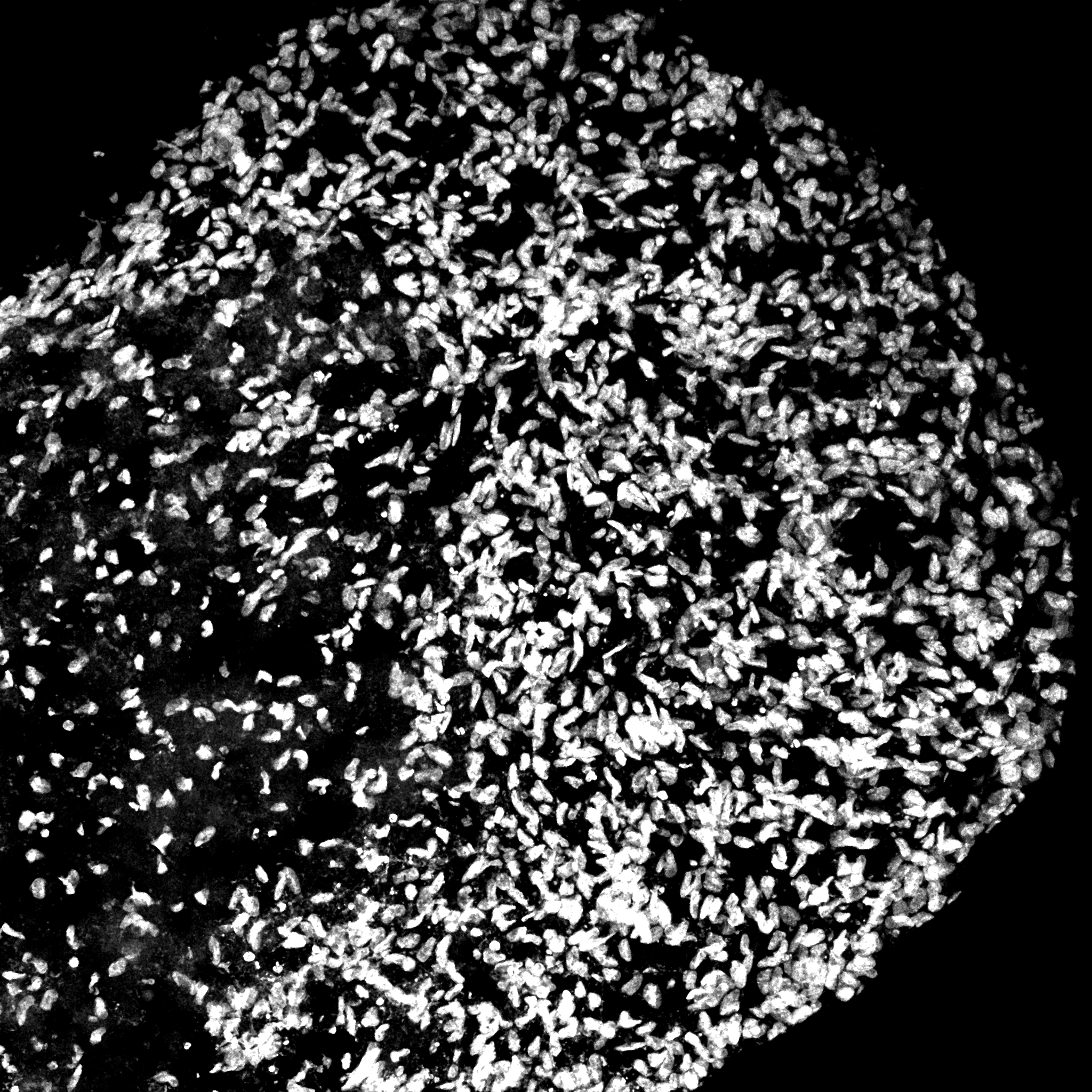

Supplement: Supplementary file 8 — Source Data for Figure 3 [file EMMM-15-e18199-s011.zip › Figure_3/3A/A'_PDO_T#1_D28_GFAP,_B3tubulin_DAPI.tif]

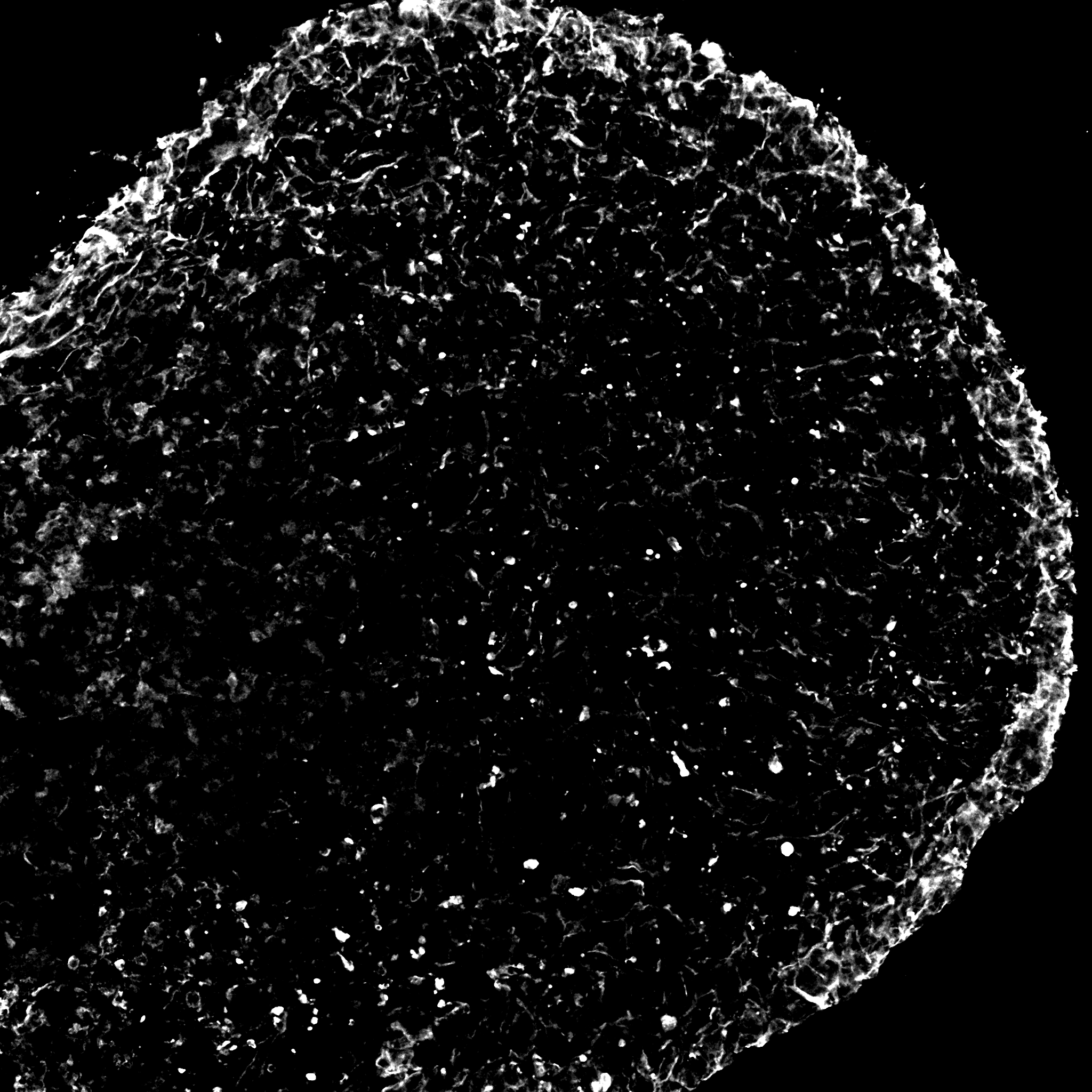

Supplement: Supplementary file 8 — Source Data for Figure 3 [file EMMM-15-e18199-s011.zip › Figure_3/3A/A'_PDO_T#1_D28_GFAP,_B3tubulin_GFAP.tif]

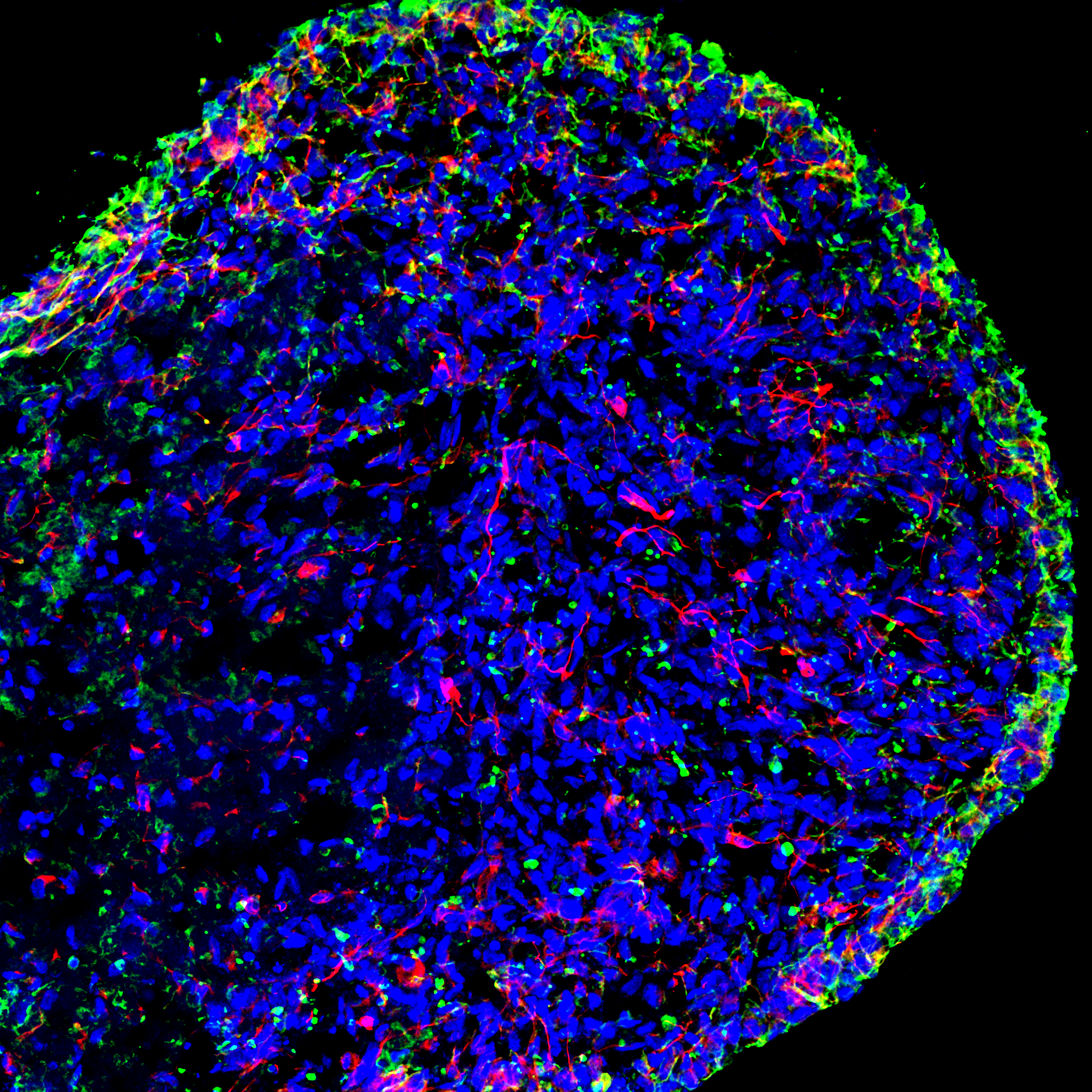

Supplement: Supplementary file 8 — Source Data for Figure 3 [file EMMM-15-e18199-s011.zip › Figure_3/3A/A'_PDO_T#1_D28_GFAP,_B3tubulin_merge.tif]

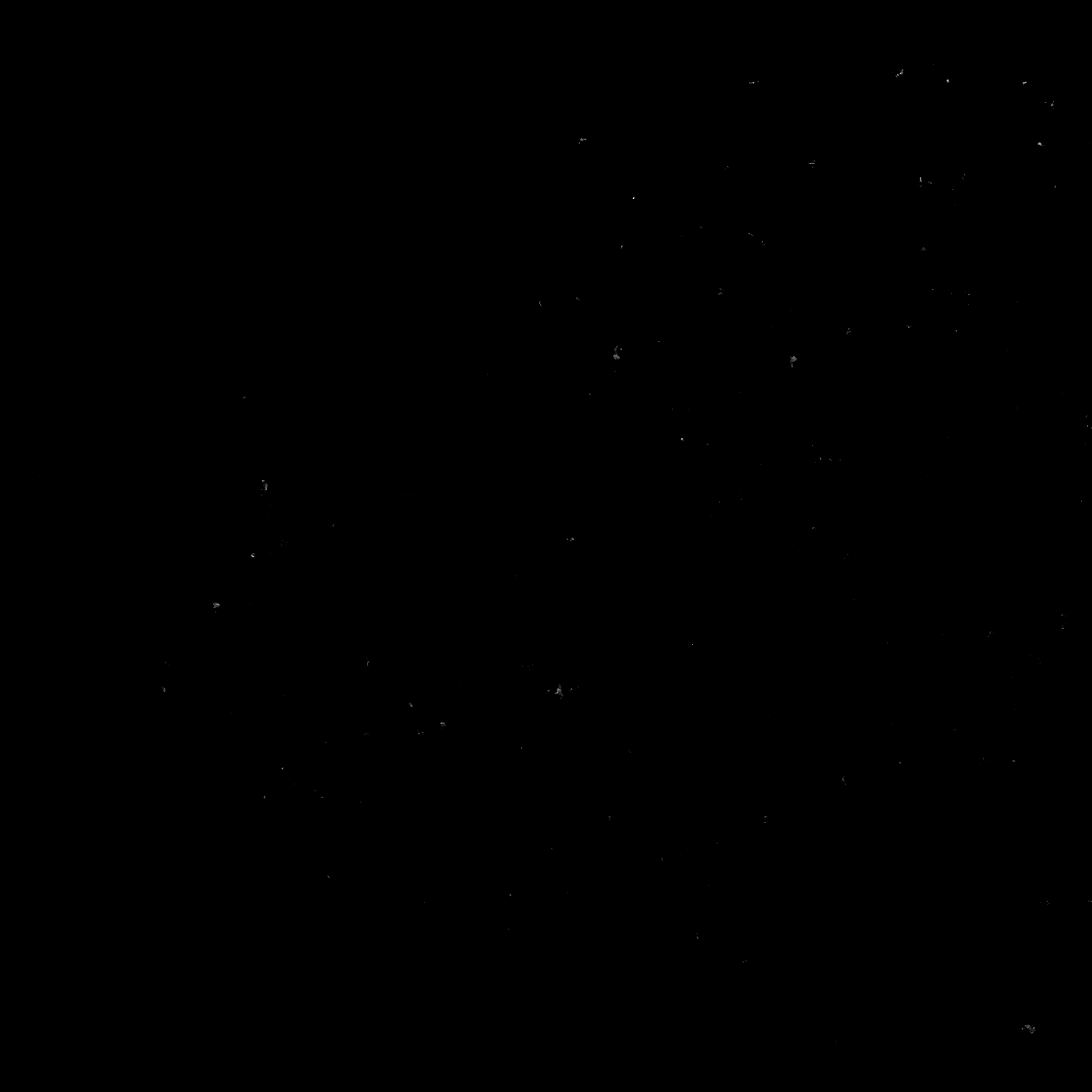

Supplement: Supplementary file 8 — Source Data for Figure 3 [file EMMM-15-e18199-s011.zip › Figure_3/3A/A'_PDO_T#1_D28_IBA1,_CD3_CD3.tif]

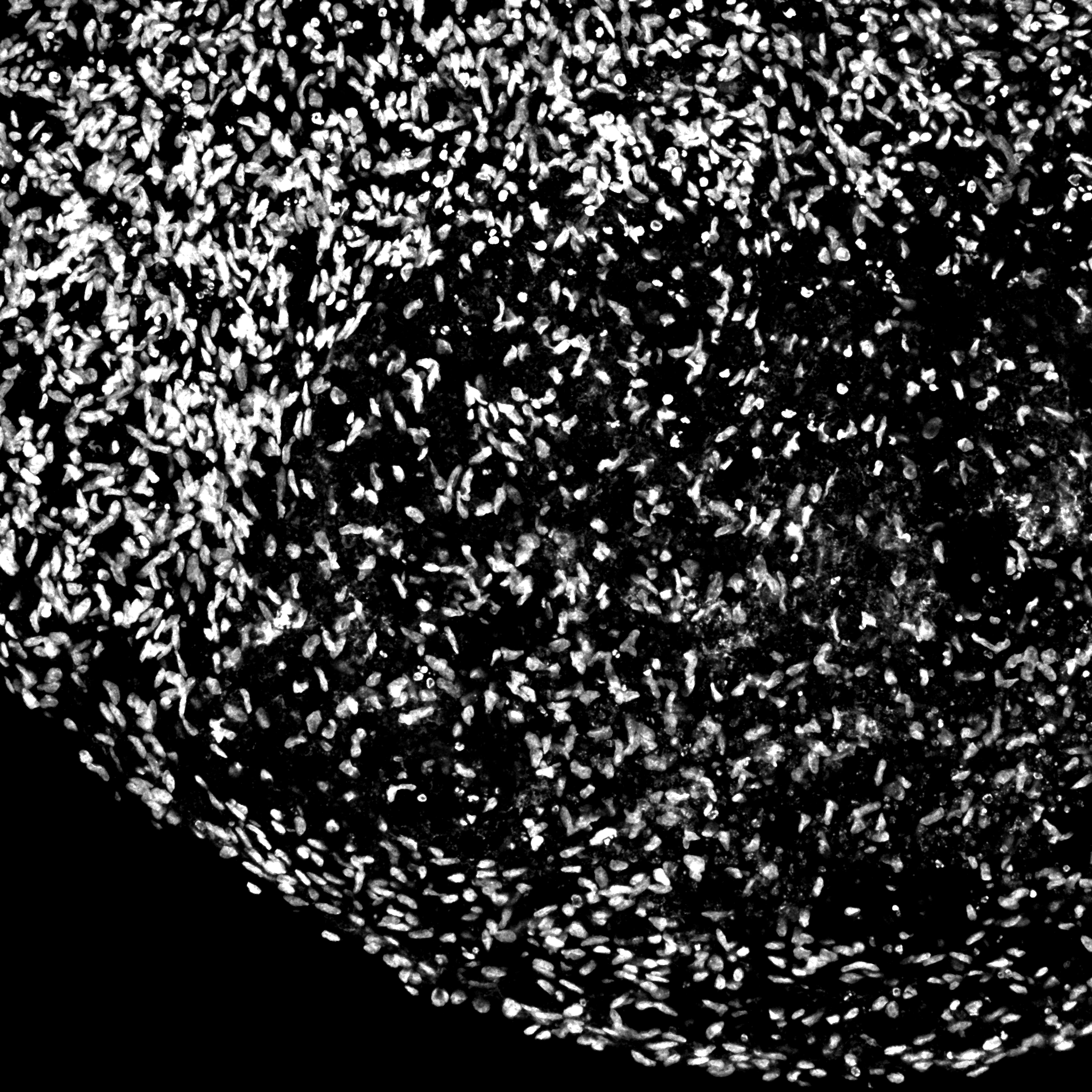

Supplement: Supplementary file 8 — Source Data for Figure 3 [file EMMM-15-e18199-s011.zip › Figure_3/3A/A'_PDO_T#1_D28_IBA1,_CD3_DAPI.tif]

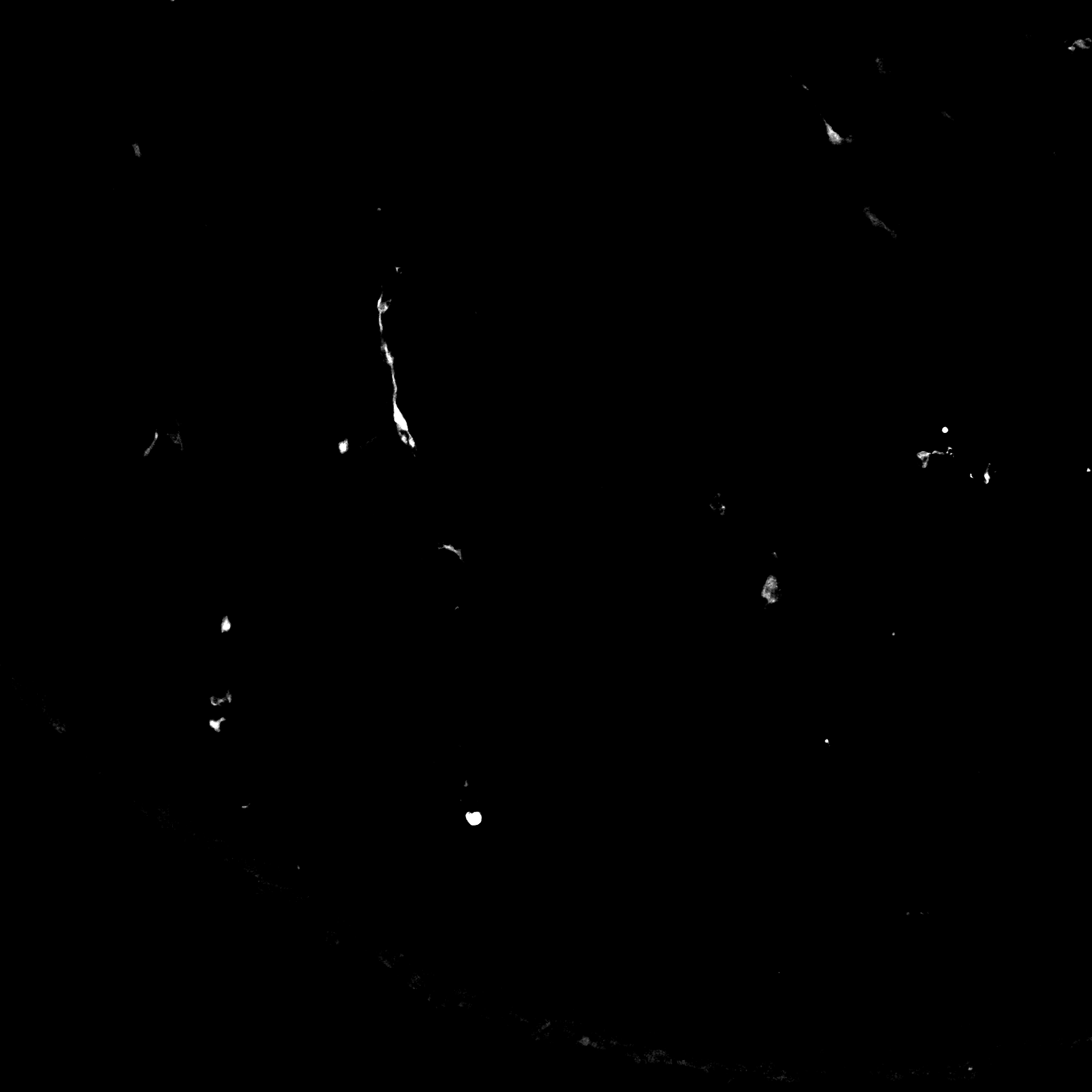

Supplement: Supplementary file 8 — Source Data for Figure 3 [file EMMM-15-e18199-s011.zip › Figure_3/3A/A'_PDO_T#1_D28_IBA1,_CD3_IBA1.tif]

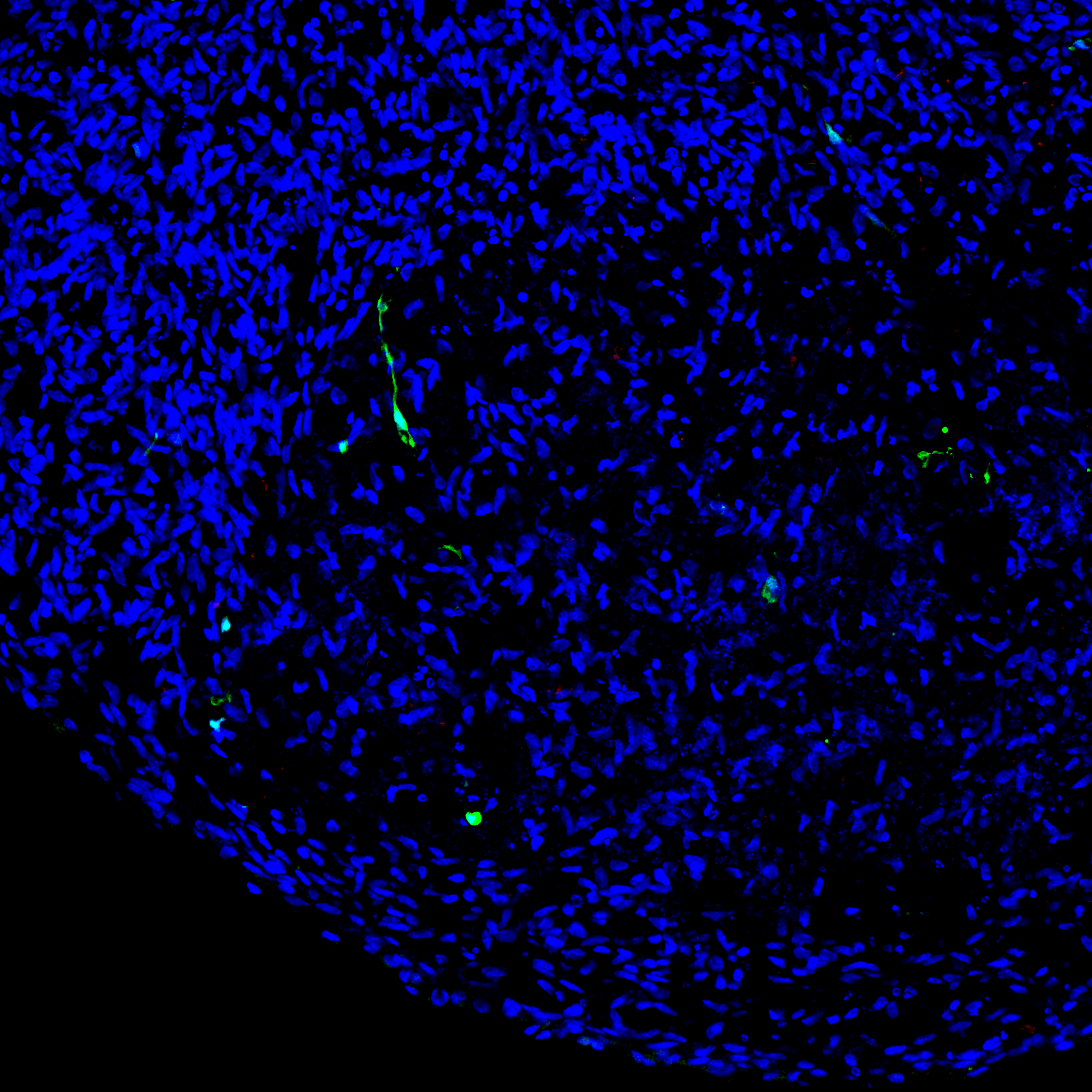

Supplement: Supplementary file 8 — Source Data for Figure 3 [file EMMM-15-e18199-s011.zip › Figure_3/3A/A'_PDO_T#1_D28_IBA1,_CD3_merge.tif]

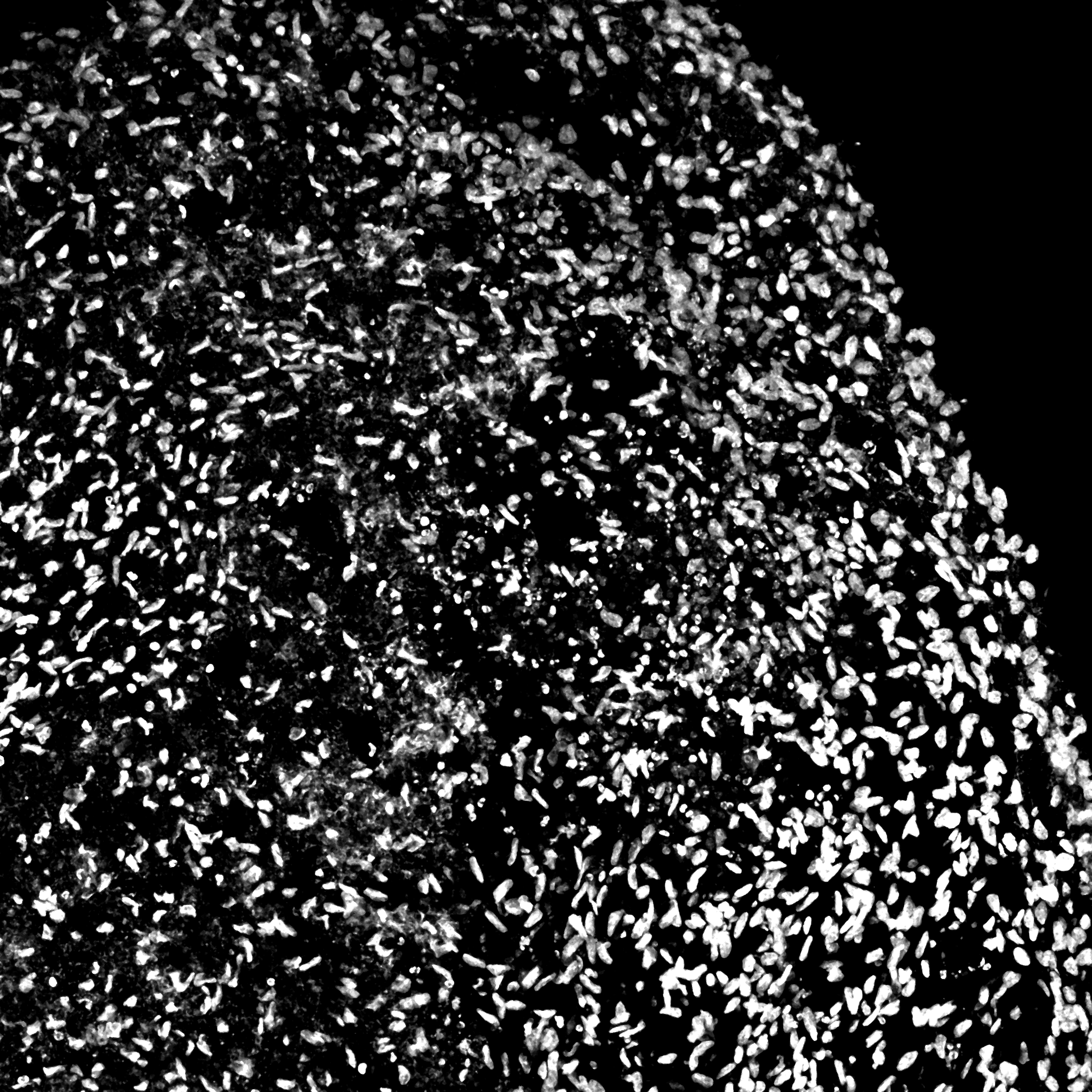

Supplement: Supplementary file 8 — Source Data for Figure 3 [file EMMM-15-e18199-s011.zip › Figure_3/3A/A'_PDO_T#1_D28_Ki67,_SOX2_DAPI.tif]

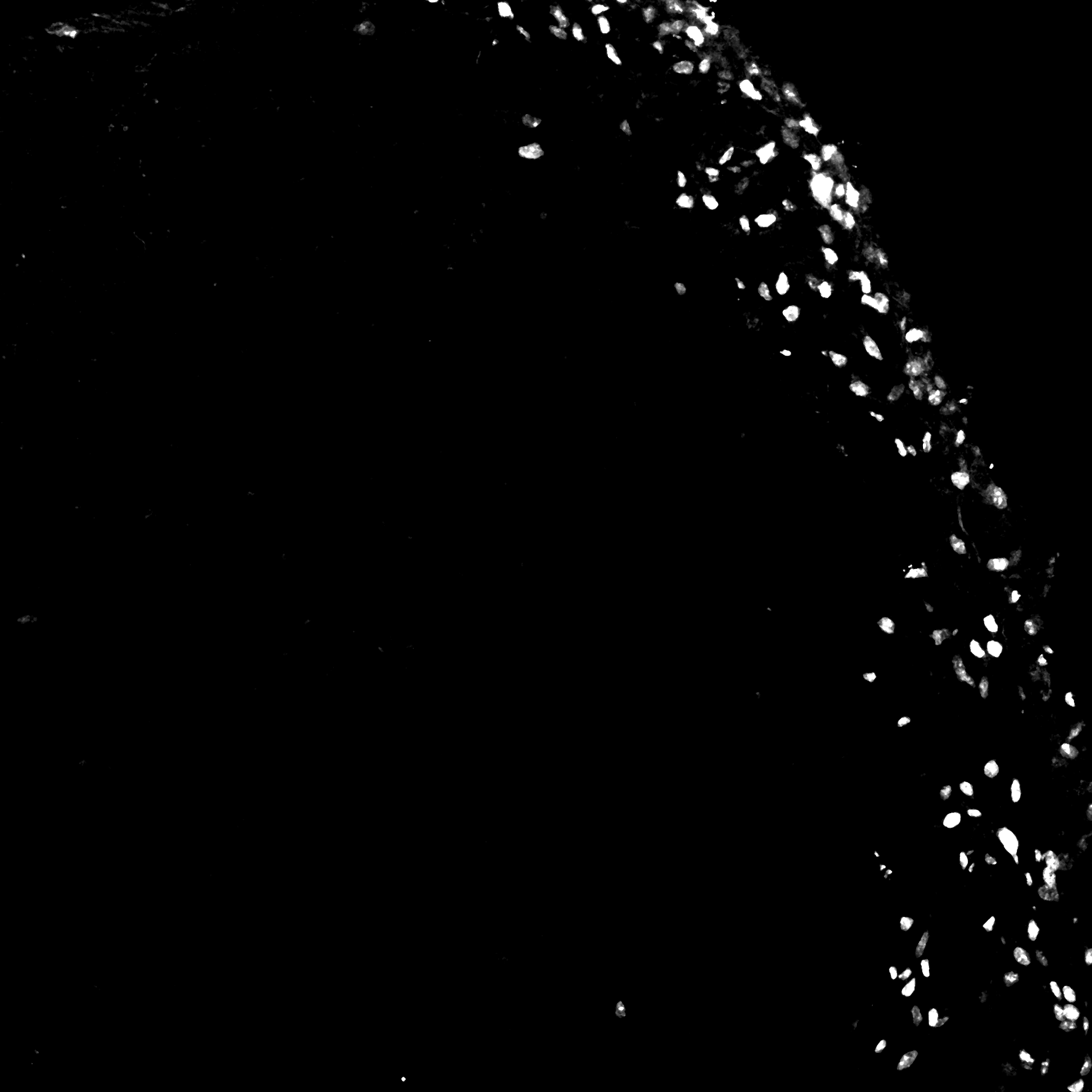

Supplement: Supplementary file 8 — Source Data for Figure 3 [file EMMM-15-e18199-s011.zip › Figure_3/3A/A'_PDO_T#1_D28_Ki67,_SOX2_Ki67.tif]

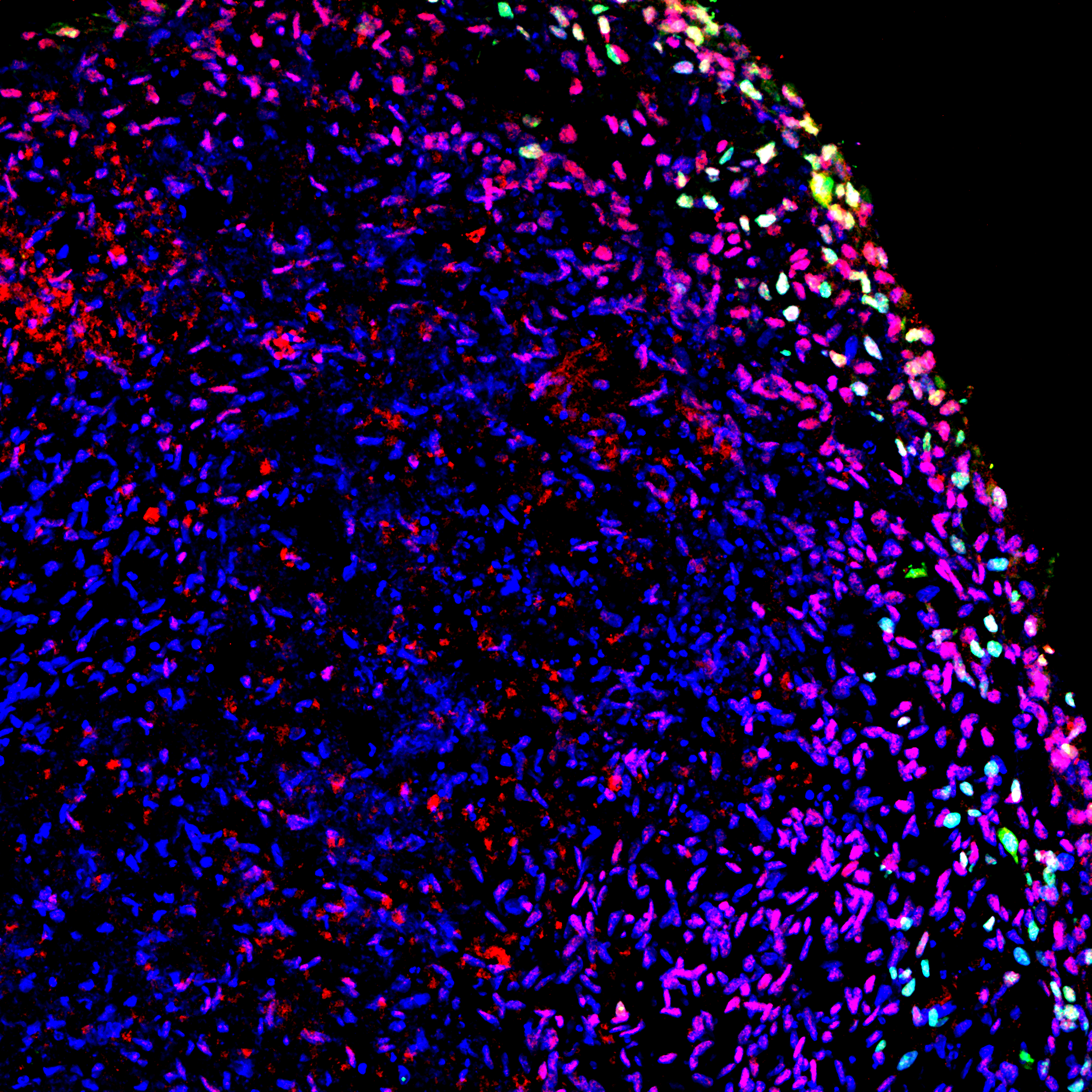

Supplement: Supplementary file 8 — Source Data for Figure 3 [file EMMM-15-e18199-s011.zip › Figure_3/3A/A'_PDO_T#1_D28_Ki67,_SOX2_merge.tif]

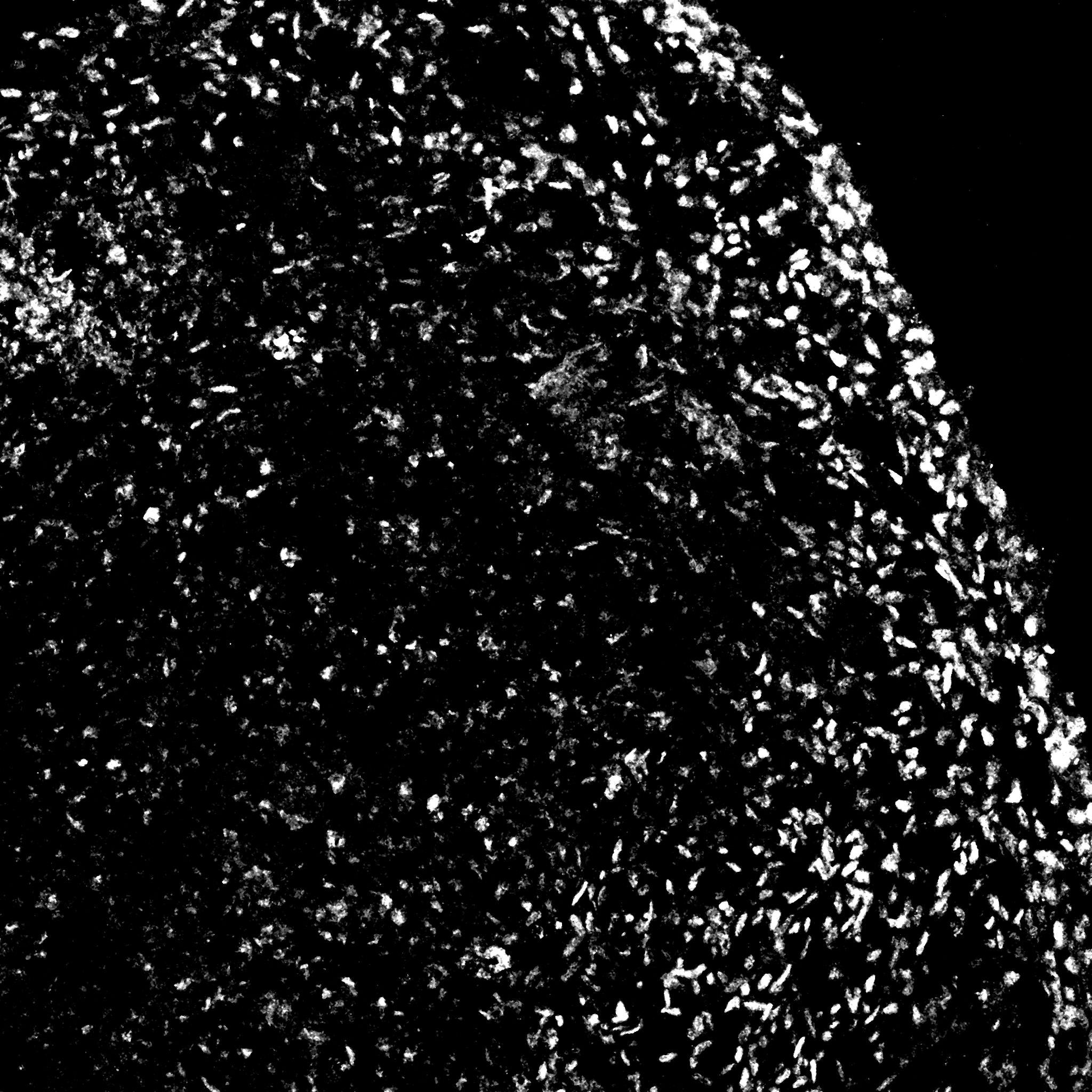

Supplement: Supplementary file 8 — Source Data for Figure 3 [file EMMM-15-e18199-s011.zip › Figure_3/3A/A'_PDO_T#1_D28_Ki67,_SOX2_SOX2.tif]

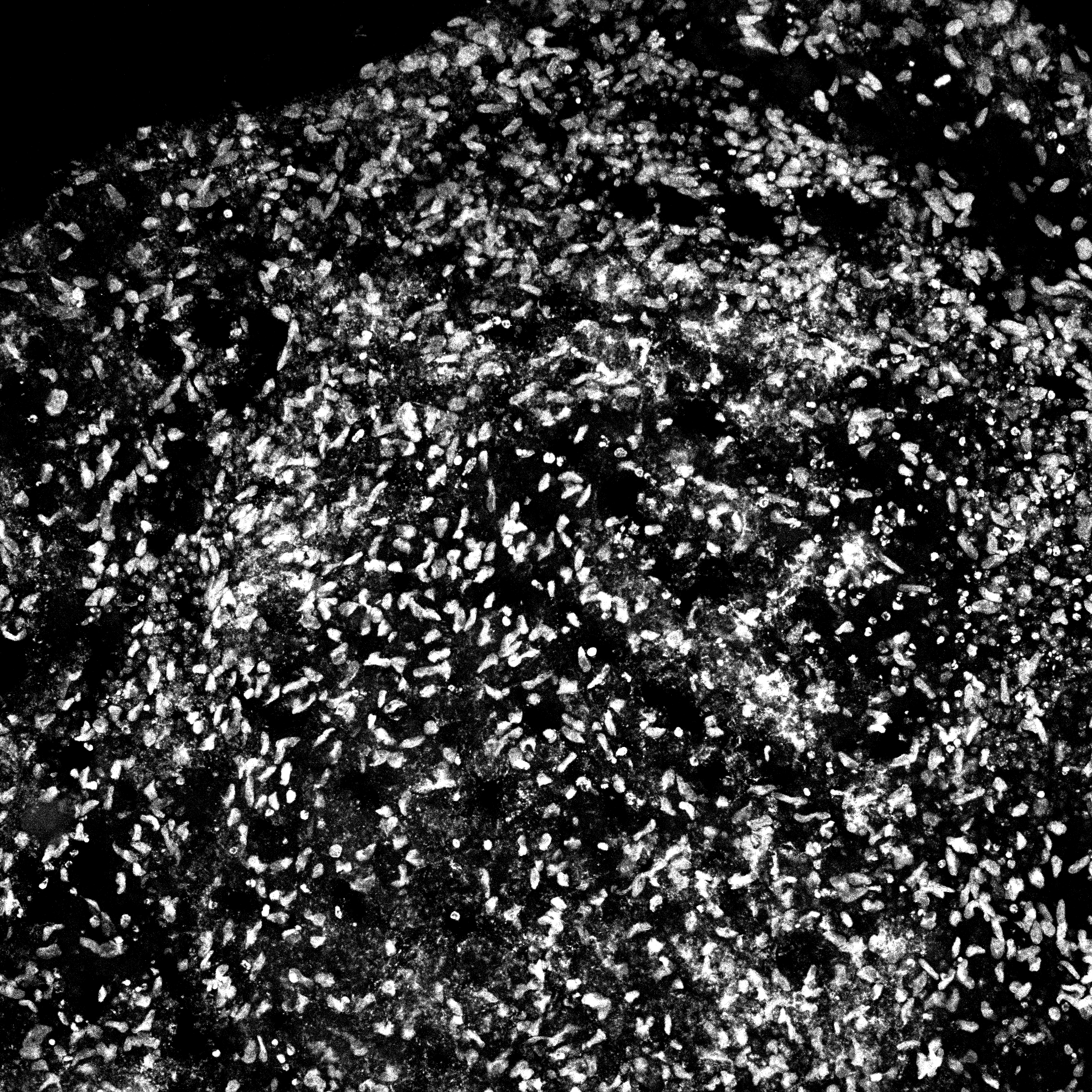

Supplement: Supplementary file 8 — Source Data for Figure 3 [file EMMM-15-e18199-s011.zip › Figure_3/3A/A'_PDO_T#1_D28_OLIG2,_Nestin_DAPI.tif]

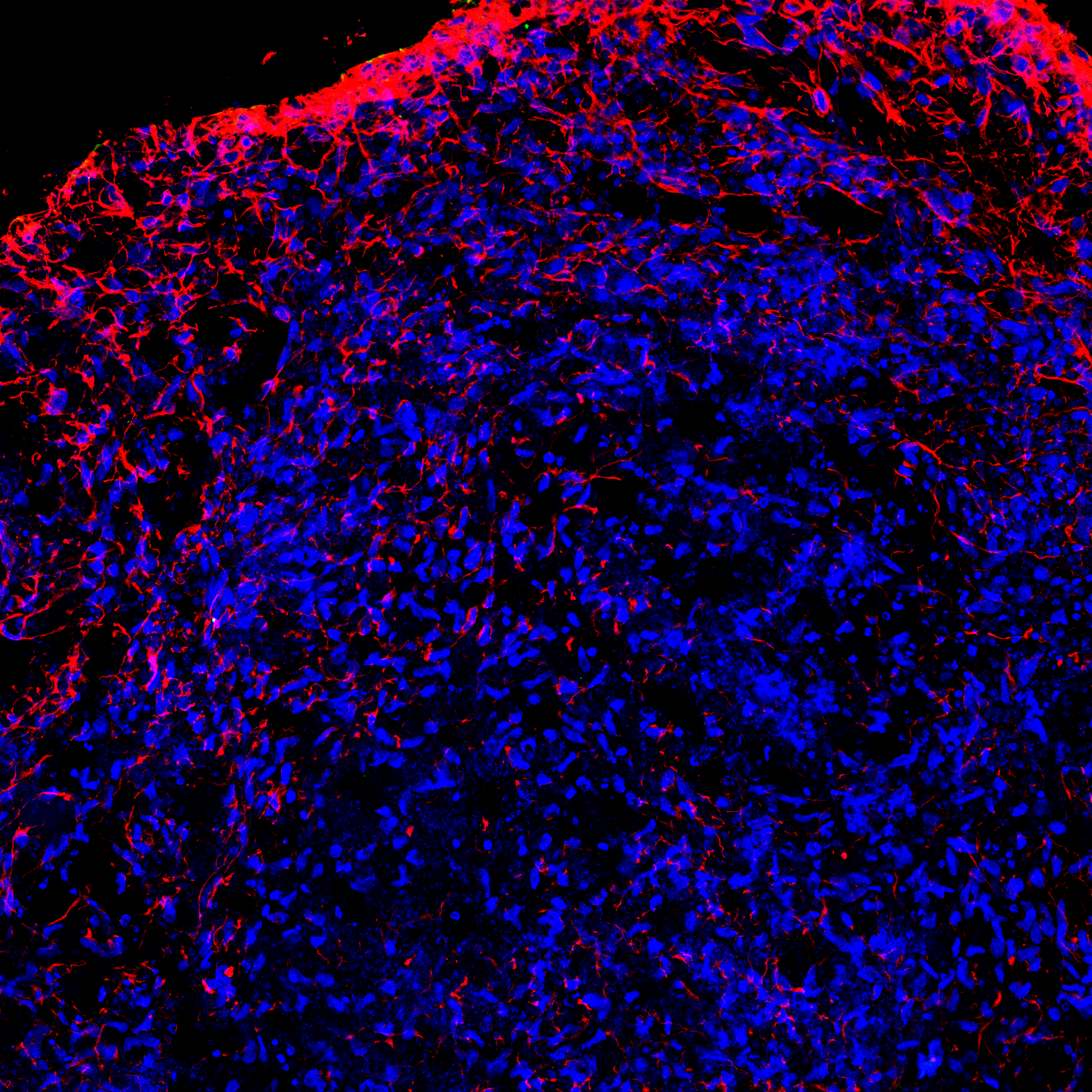

Supplement: Supplementary file 8 — Source Data for Figure 3 [file EMMM-15-e18199-s011.zip › Figure_3/3A/A'_PDO_T#1_D28_OLIG2,_Nestin_merge.tif]

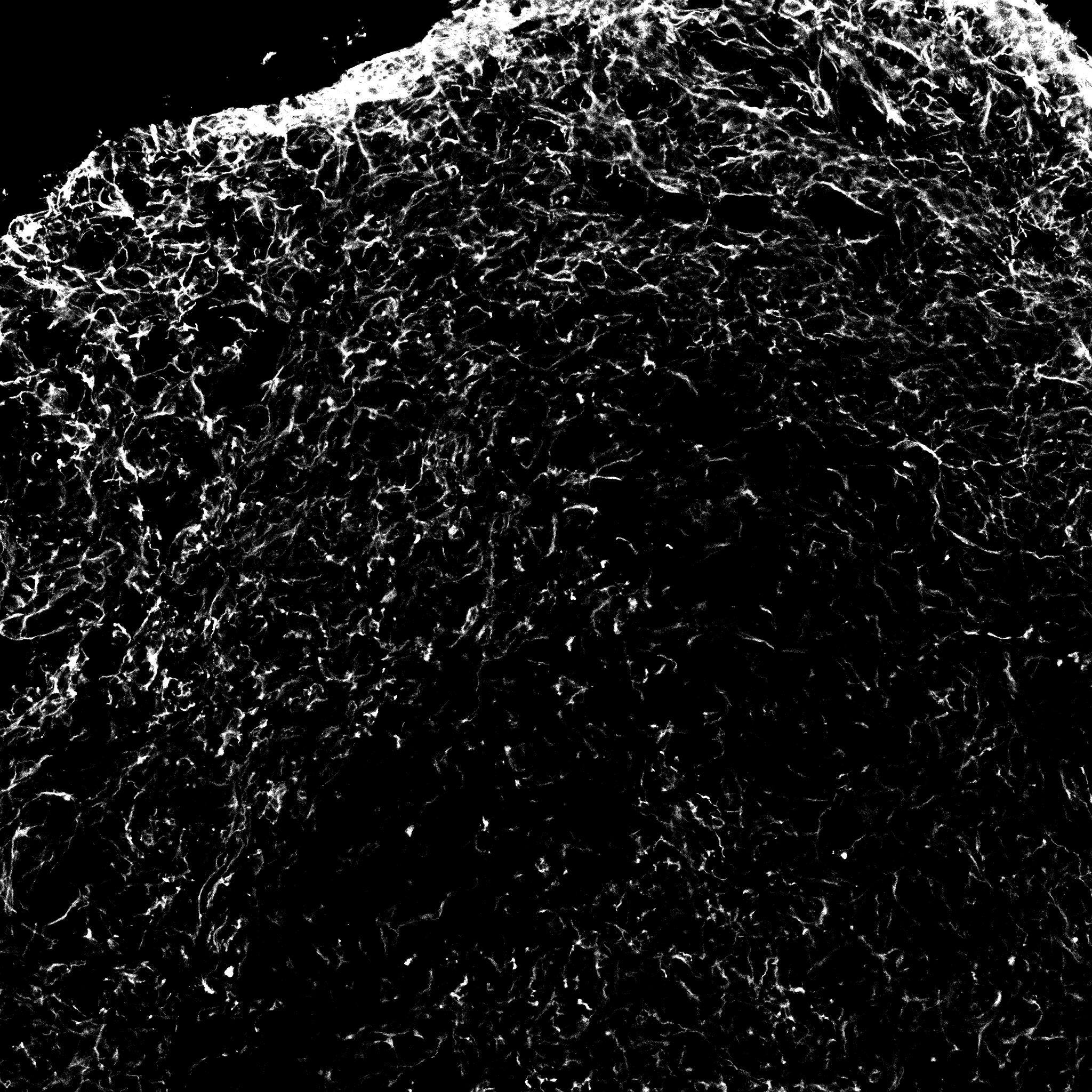

Supplement: Supplementary file 8 — Source Data for Figure 3 [file EMMM-15-e18199-s011.zip › Figure_3/3A/A'_PDO_T#1_D28_OLIG2,_Nestin_Nestin.tif]

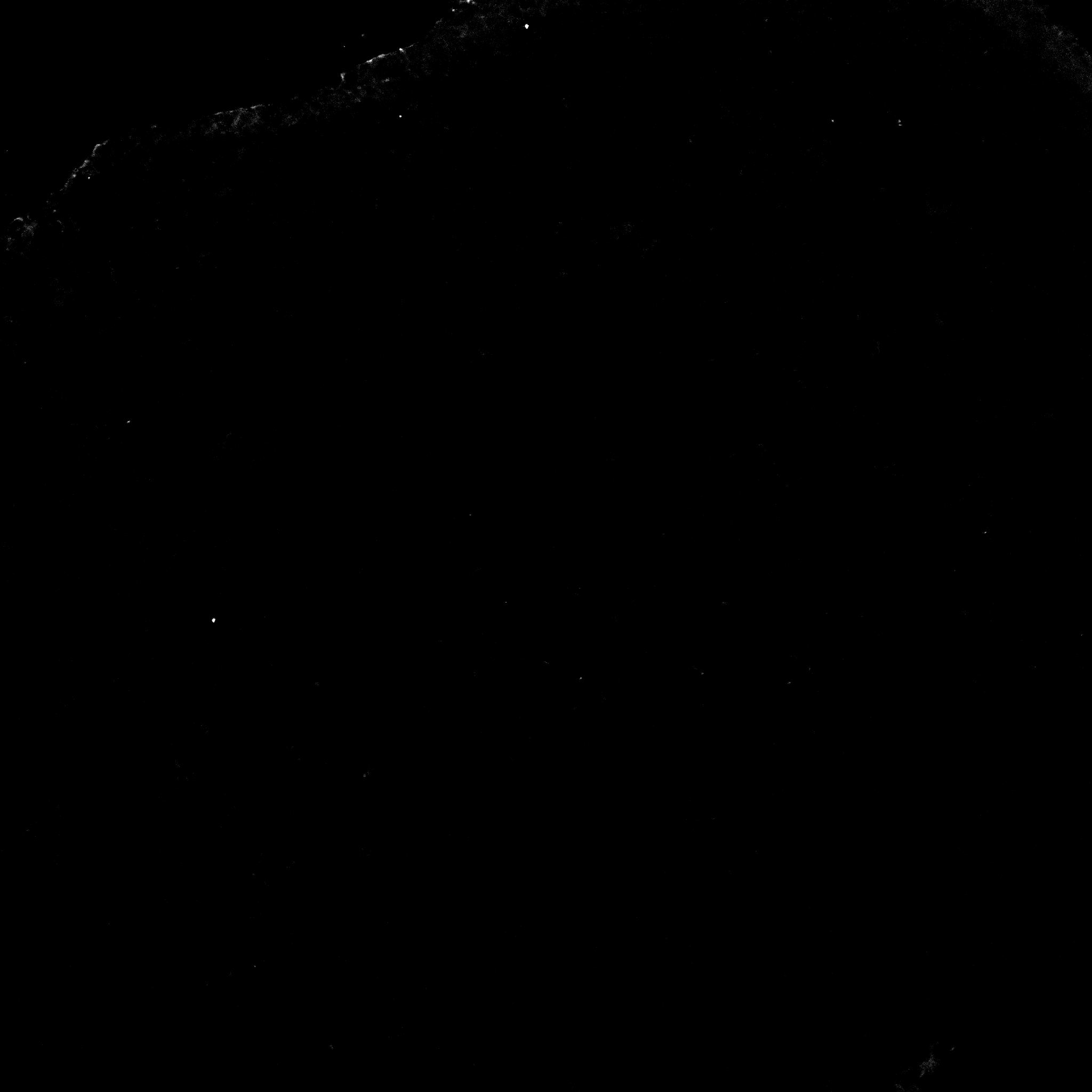

Supplement: Supplementary file 8 — Source Data for Figure 3 [file EMMM-15-e18199-s011.zip › Figure_3/3A/A'_PDO_T#1_D28_OLIG2,_Nestin_OLIG2.tif]

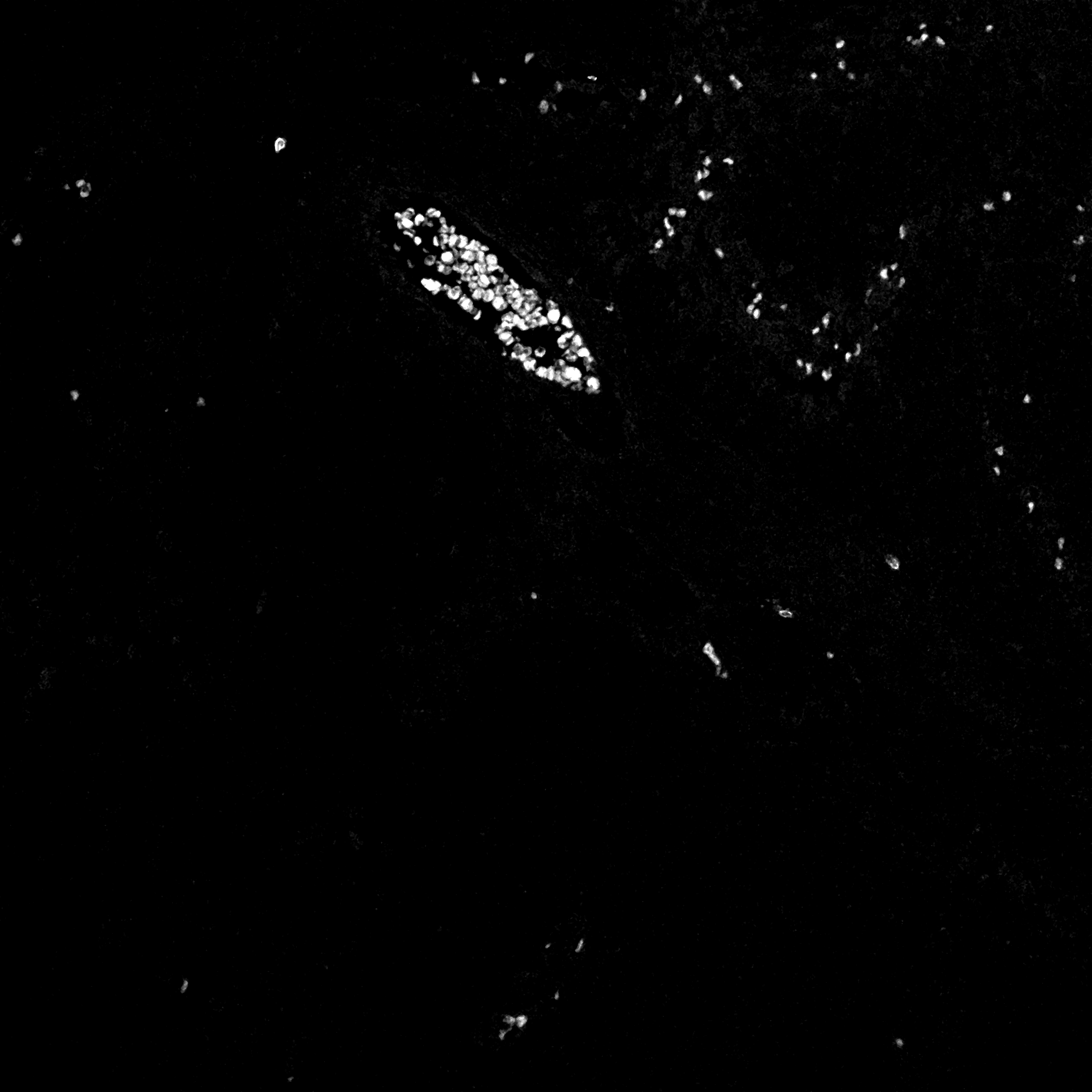

Supplement: Supplementary file 8 — Source Data for Figure 3 [file EMMM-15-e18199-s011.zip › Figure_3/3A/A'_Primary_T#1_CD34,_CD3_CD3.tif]

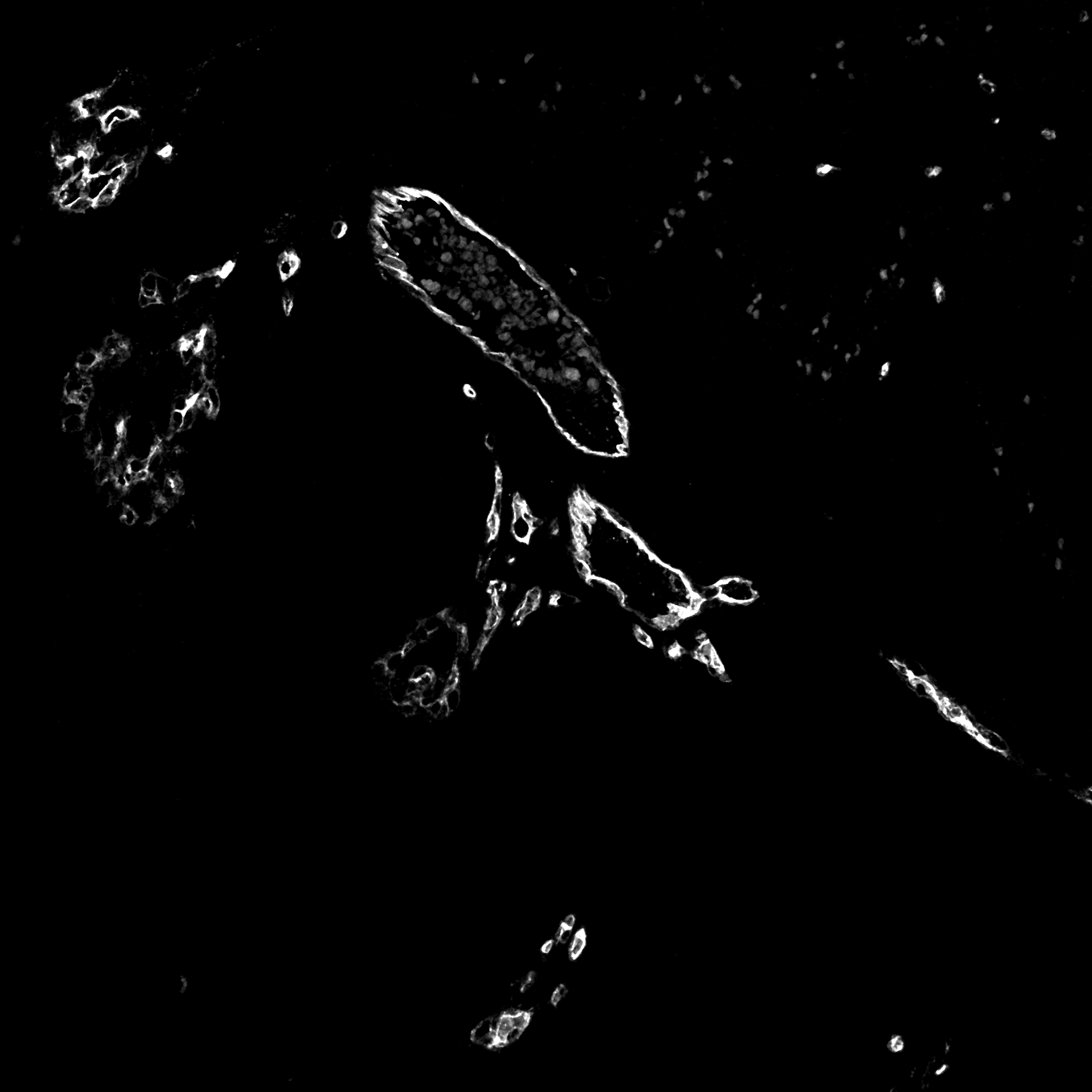

Supplement: Supplementary file 8 — Source Data for Figure 3 [file EMMM-15-e18199-s011.zip › Figure_3/3A/A'_Primary_T#1_CD34,_CD3_CD34.tif]

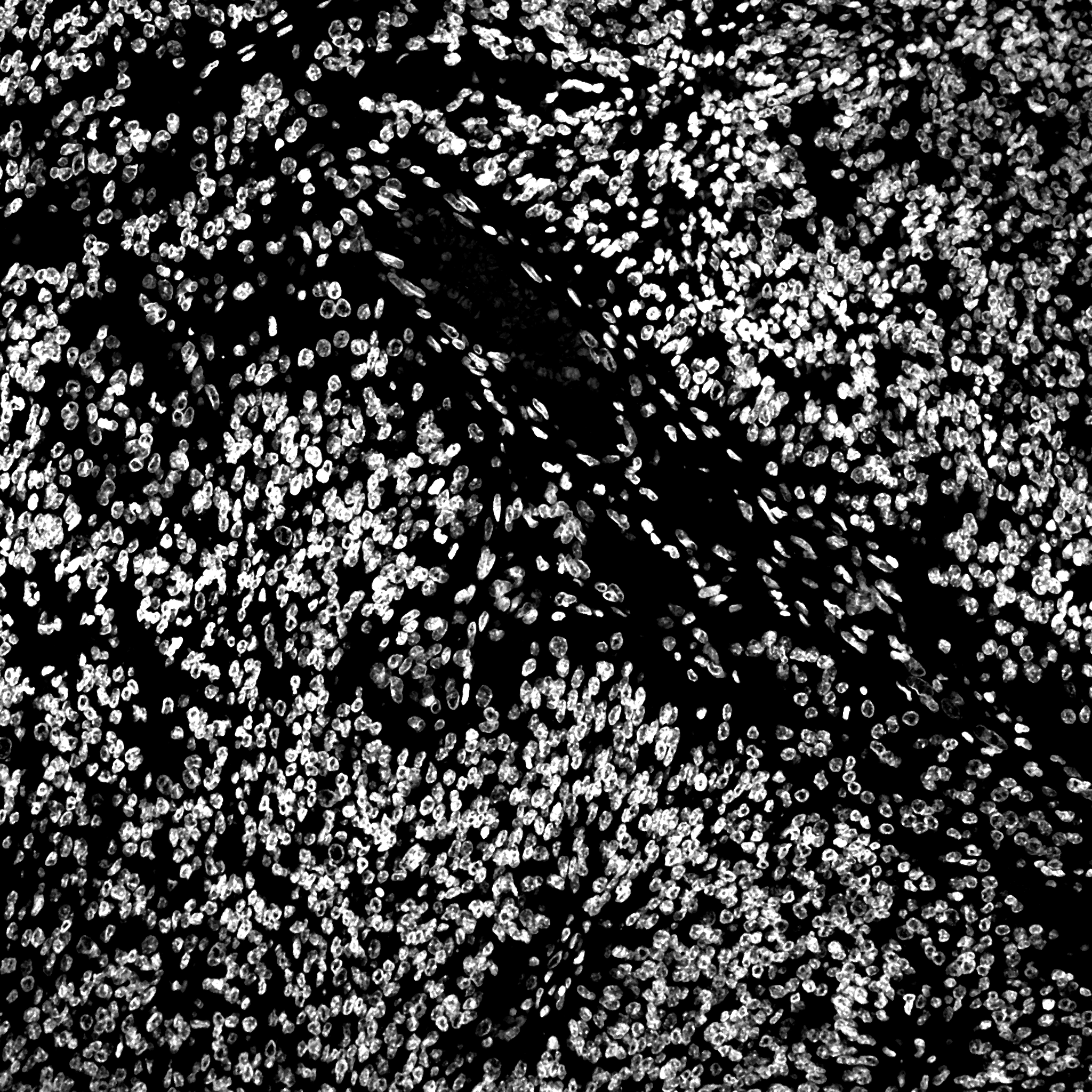

Supplement: Supplementary file 8 — Source Data for Figure 3 [file EMMM-15-e18199-s011.zip › Figure_3/3A/A'_Primary_T#1_CD34,_CD3_DAPI.tif]

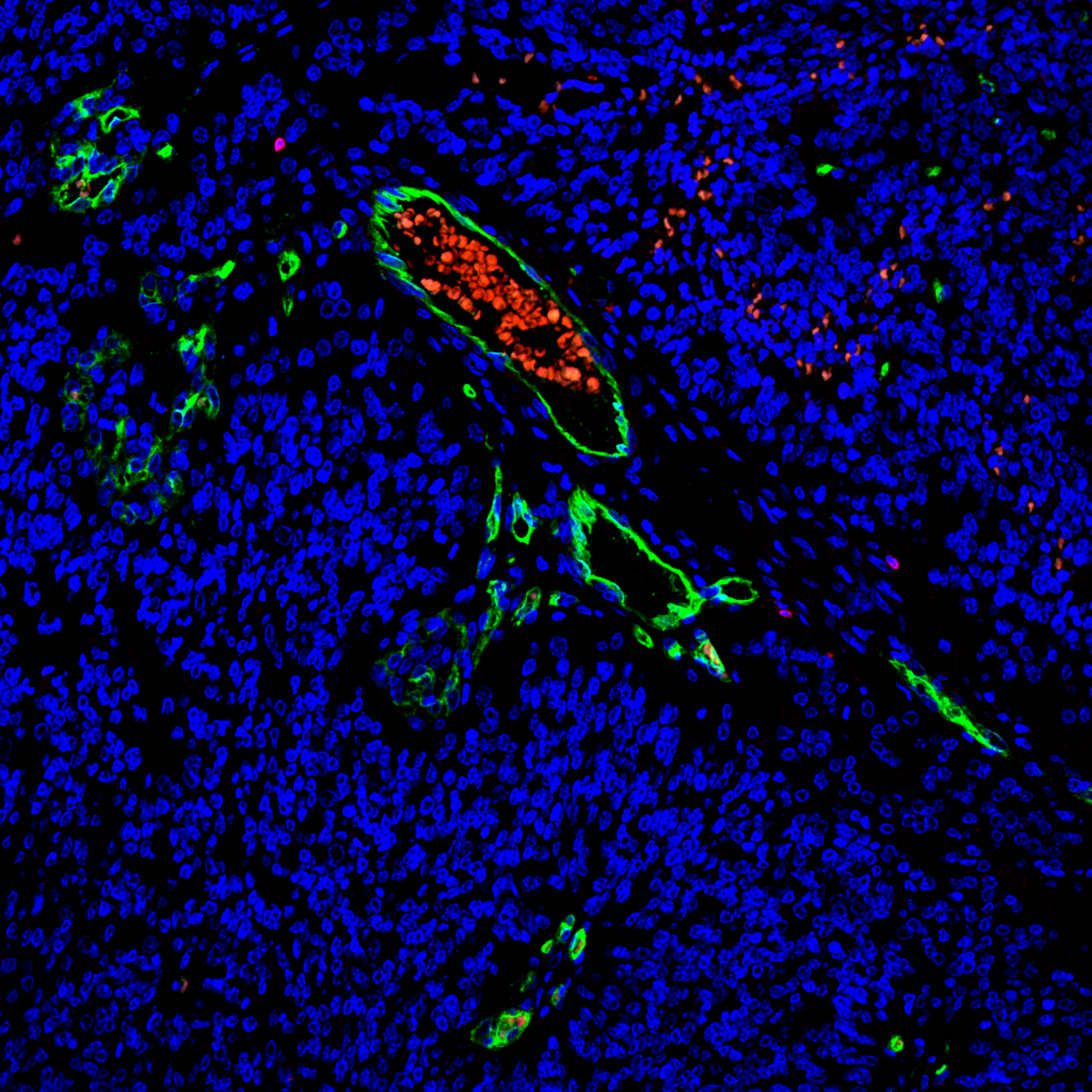

Supplement: Supplementary file 8 — Source Data for Figure 3 [file EMMM-15-e18199-s011.zip › Figure_3/3A/A'_Primary_T#1_CD34,_CD3_merge.tif]

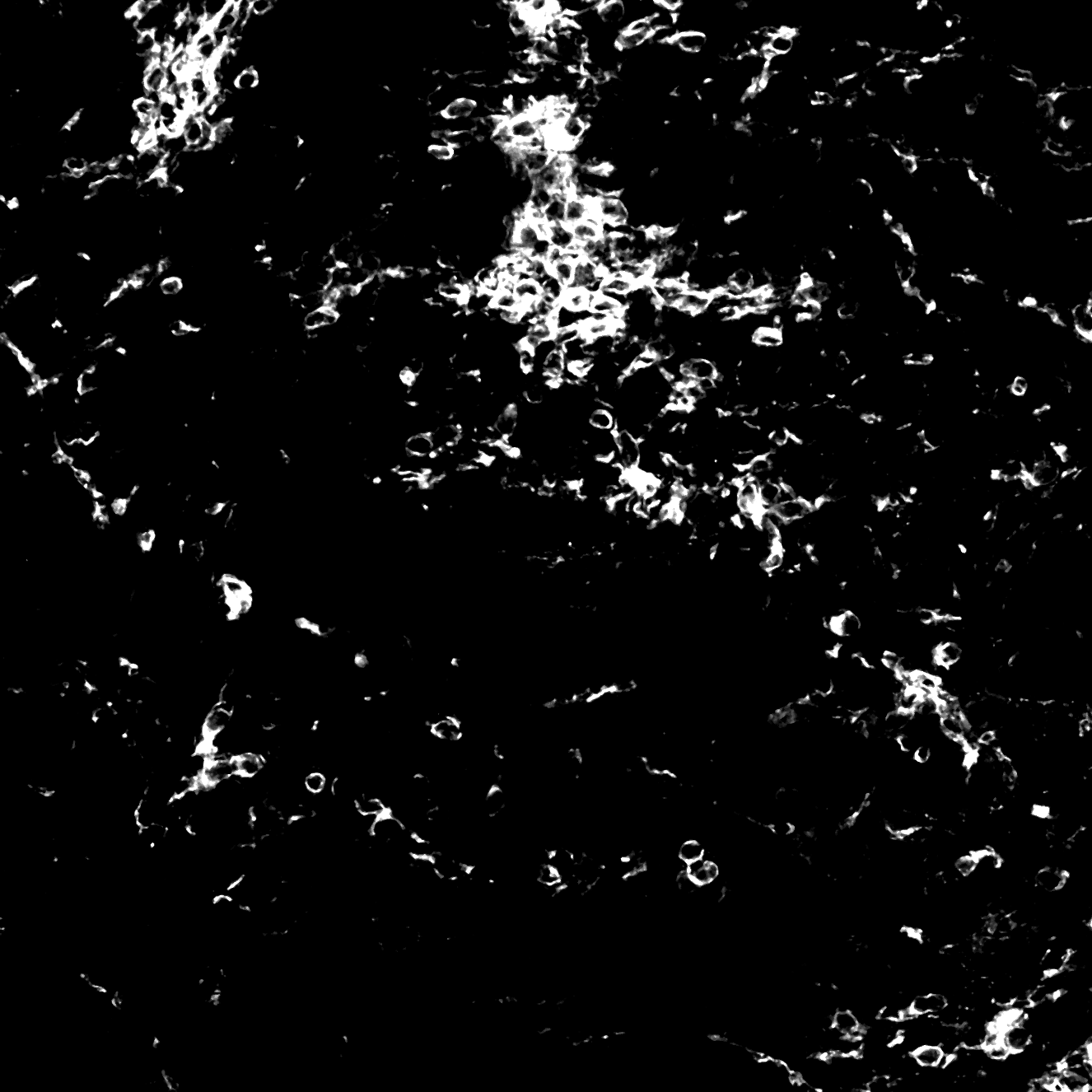

Supplement: Supplementary file 8 — Source Data for Figure 3 [file EMMM-15-e18199-s011.zip › Figure_3/3A/A'_Primary_T#1_GFAP,_B3tubulin_B3tubulin.tif]

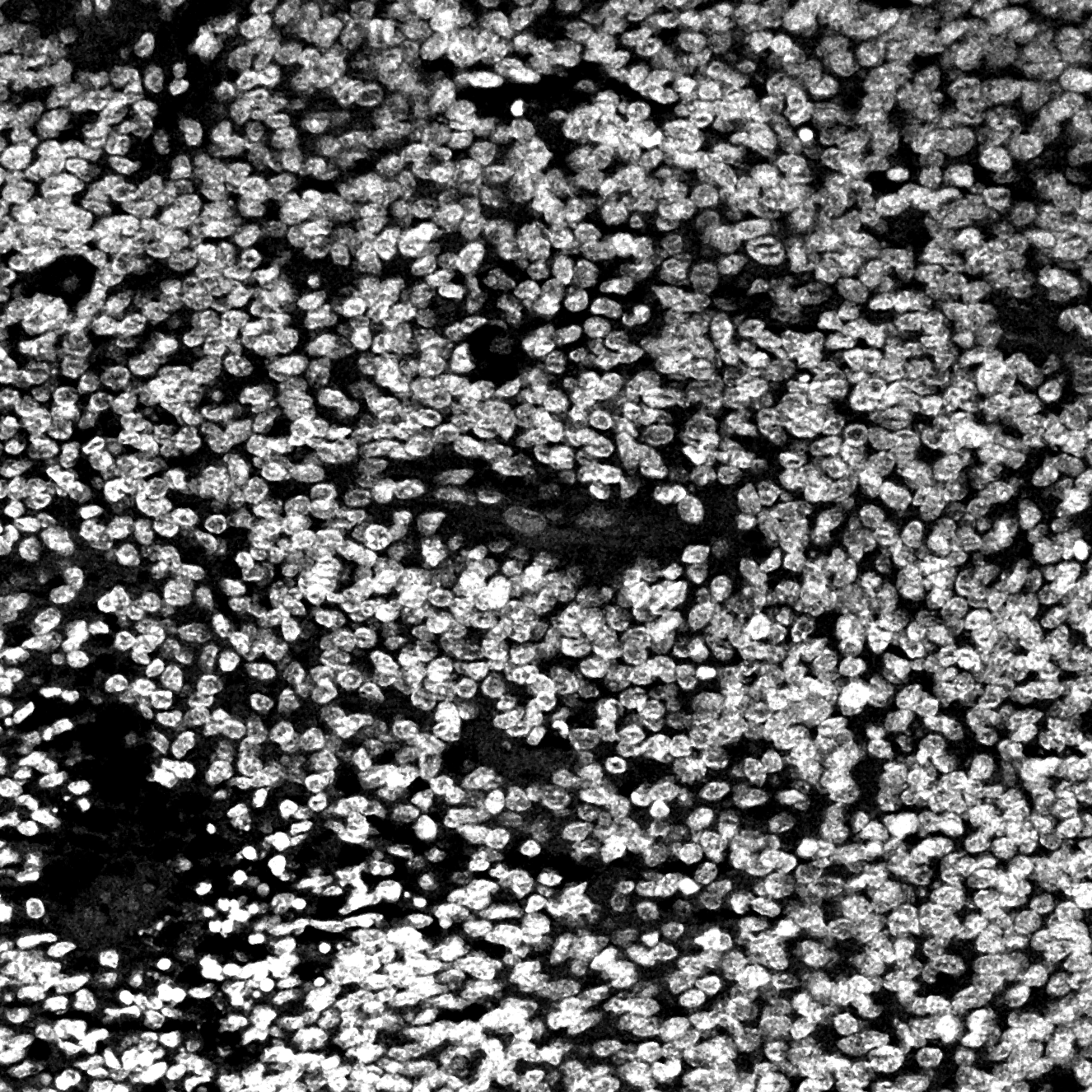

Supplement: Supplementary file 8 — Source Data for Figure 3 [file EMMM-15-e18199-s011.zip › Figure_3/3A/A'_Primary_T#1_GFAP,_B3tubulin_DAPI.tif]

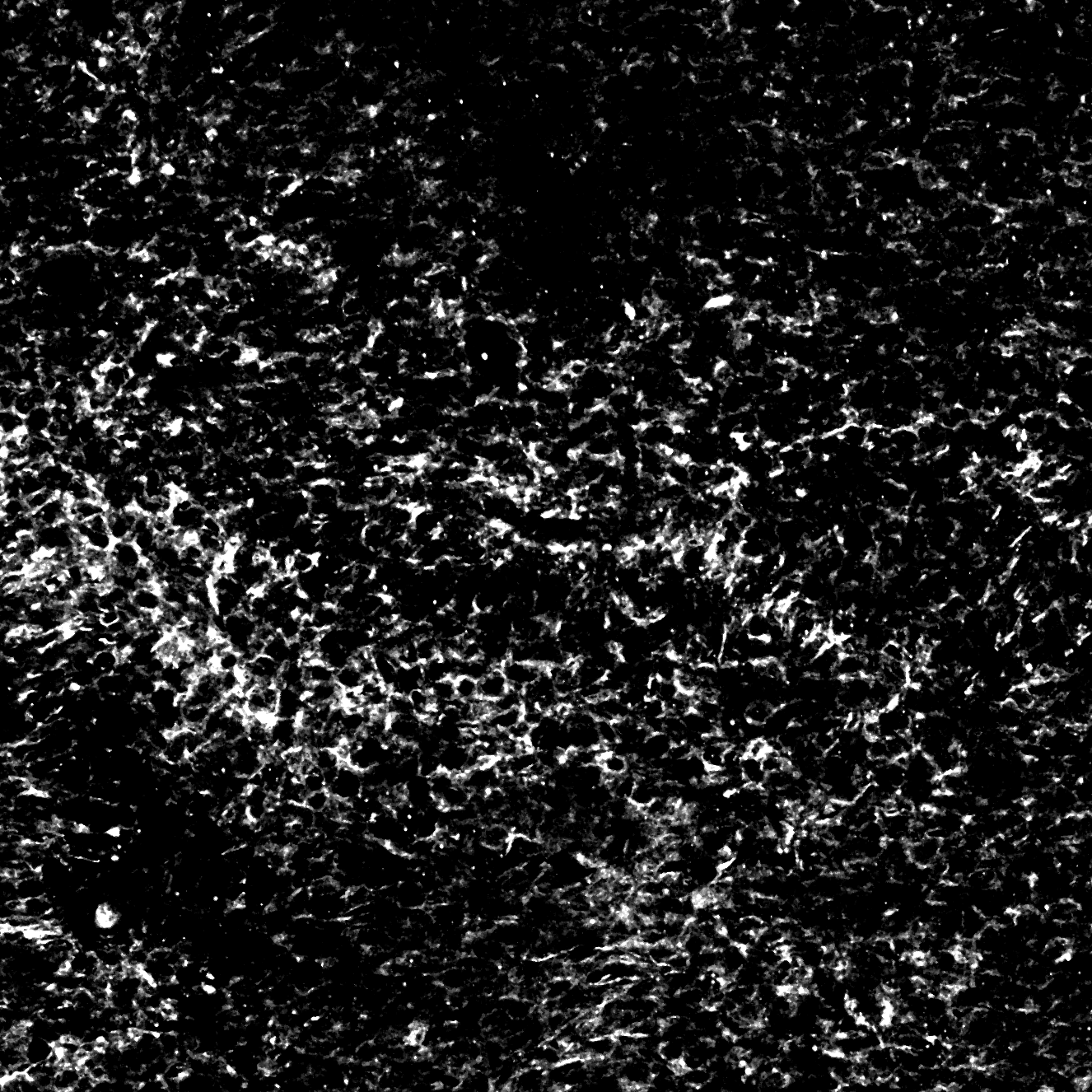

Supplement: Supplementary file 8 — Source Data for Figure 3 [file EMMM-15-e18199-s011.zip › Figure_3/3A/A'_Primary_T#1_GFAP,_B3tubulin_GFAP.tif]

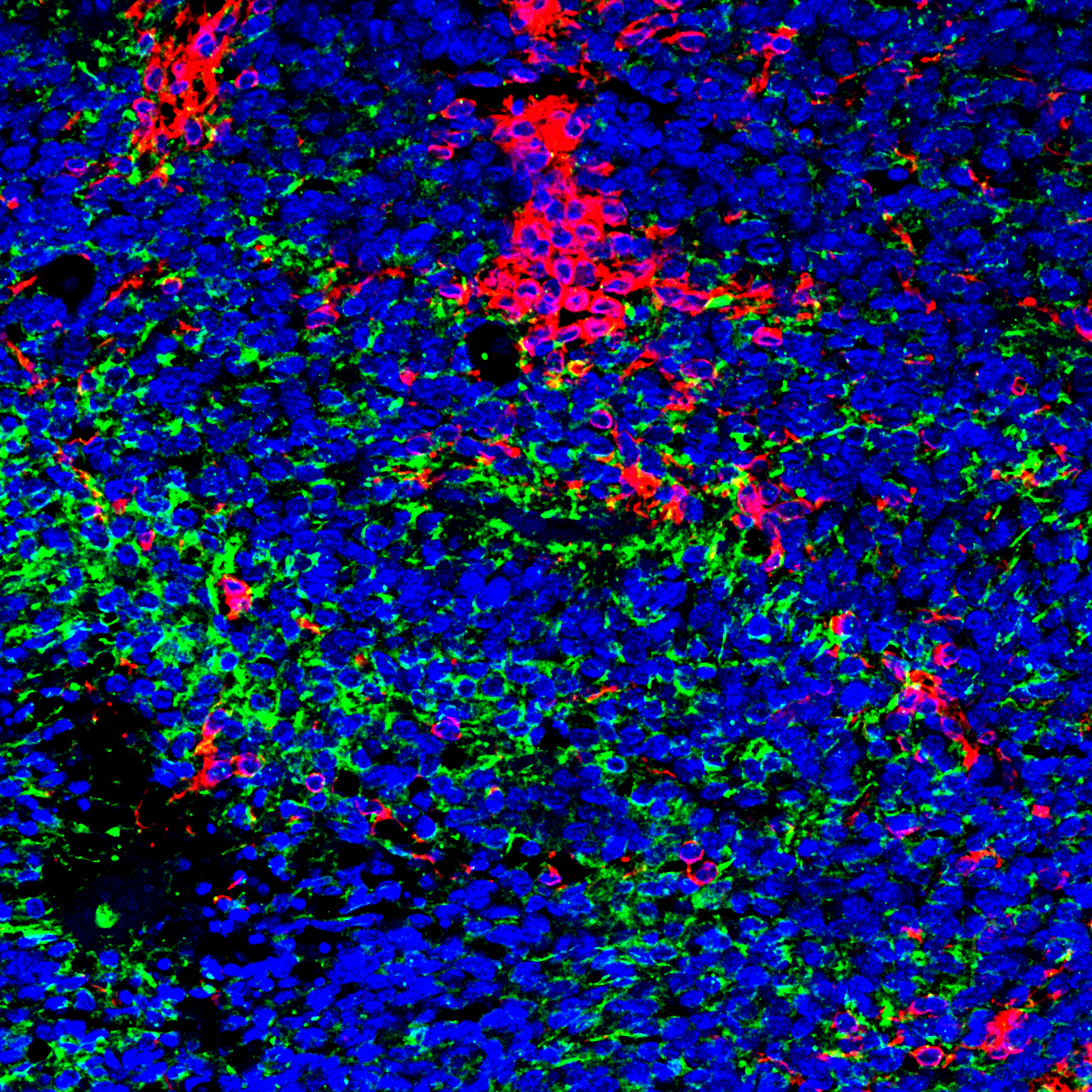

Supplement: Supplementary file 8 — Source Data for Figure 3 [file EMMM-15-e18199-s011.zip › Figure_3/3A/A'_Primary_T#1_GFAP,_B3tubulin_merge.tif]

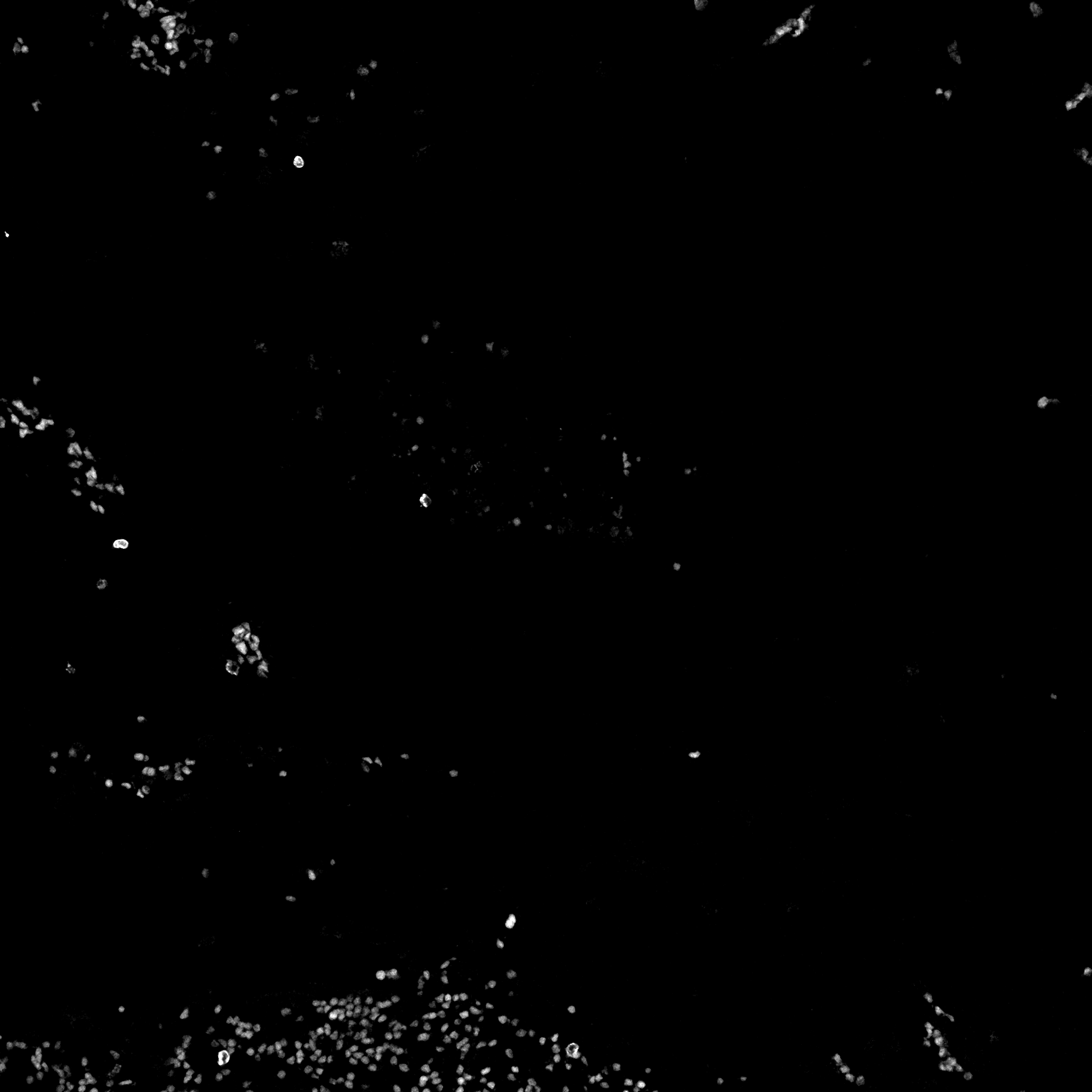

Supplement: Supplementary file 8 — Source Data for Figure 3 [file EMMM-15-e18199-s011.zip › Figure_3/3A/A'_Primary_T#1_IBA1,_CD3_CD3.tif]

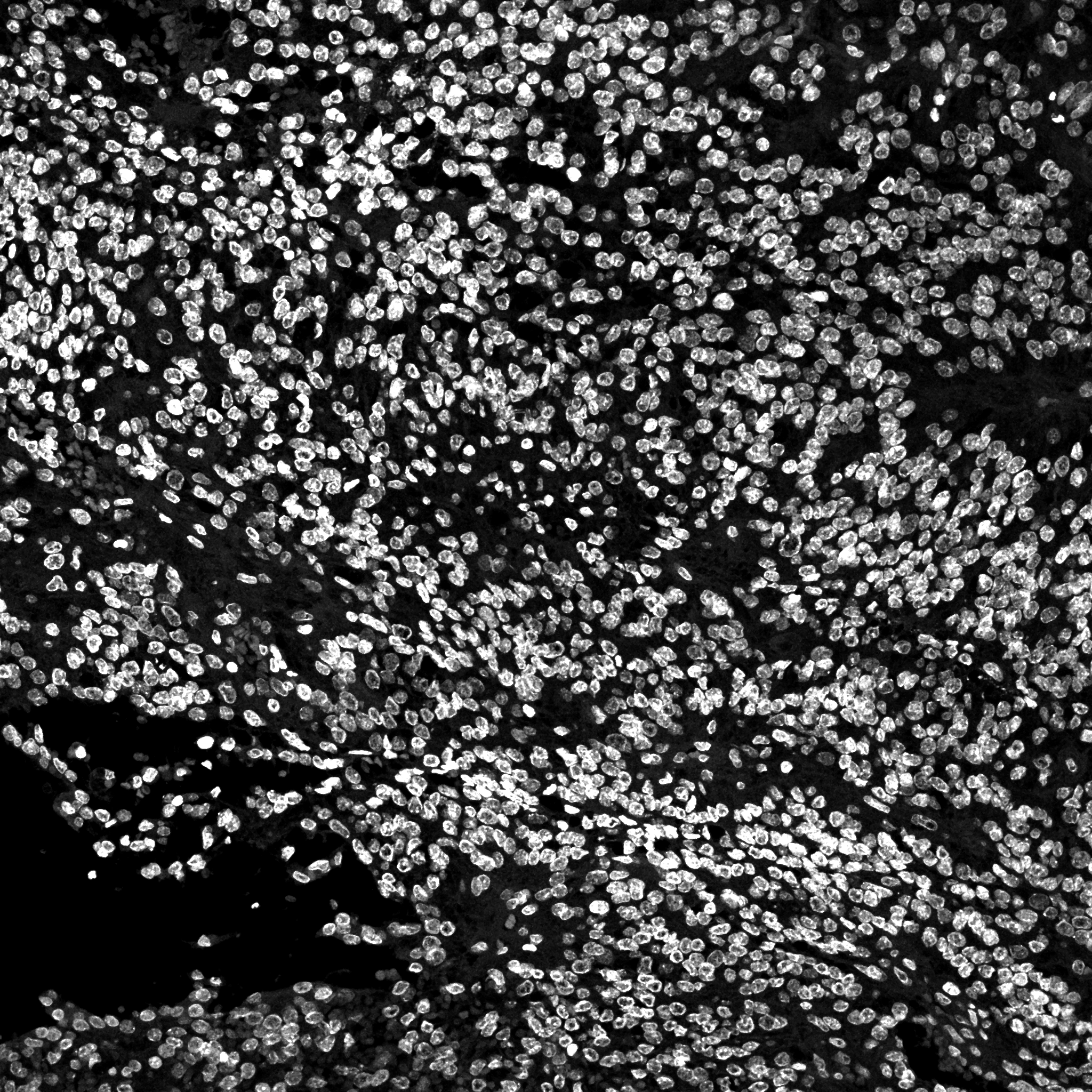

Supplement: Supplementary file 8 — Source Data for Figure 3 [file EMMM-15-e18199-s011.zip › Figure_3/3A/A'_Primary_T#1_IBA1,_CD3_DAPI.tif]

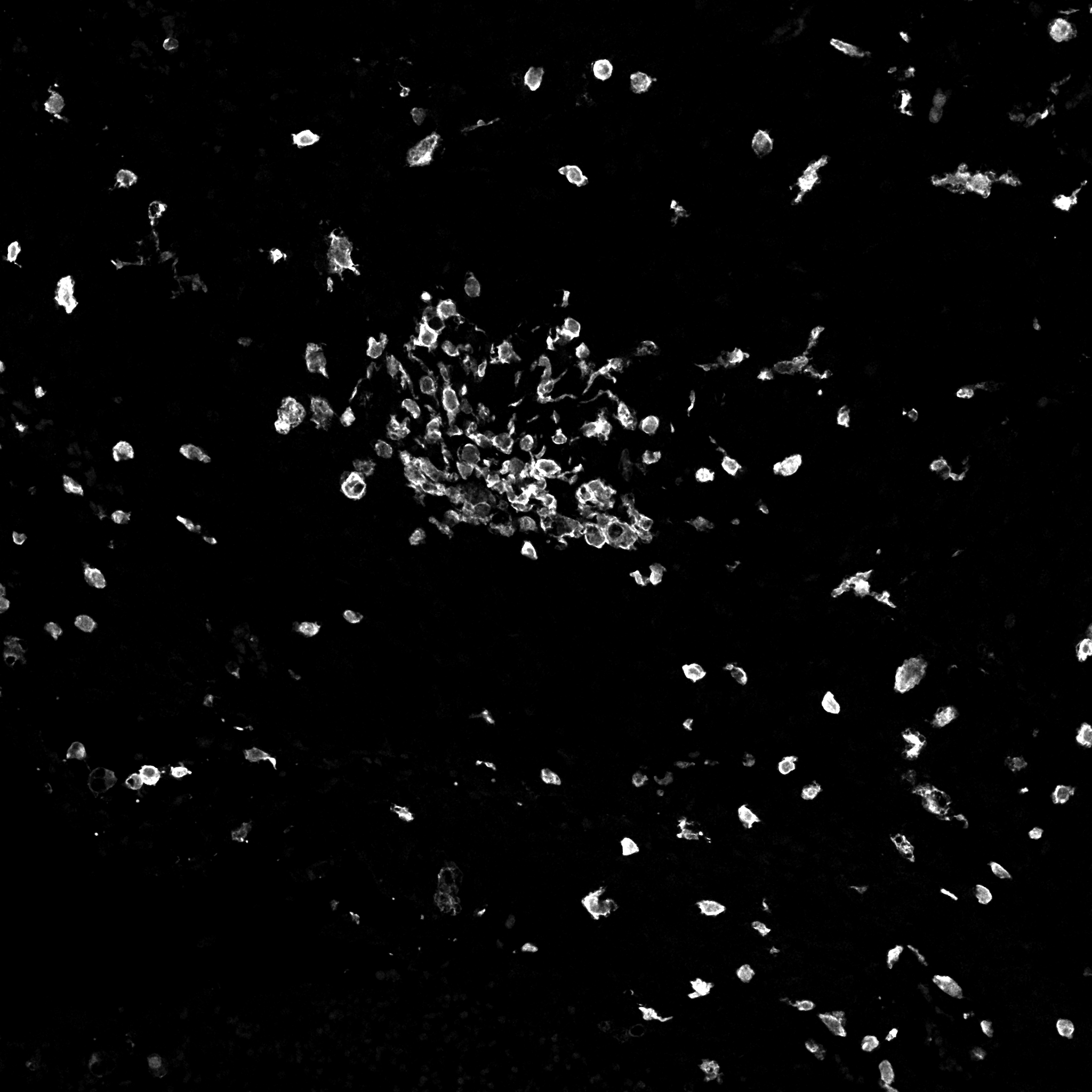

Supplement: Supplementary file 8 — Source Data for Figure 3 [file EMMM-15-e18199-s011.zip › Figure_3/3A/A'_Primary_T#1_IBA1,_CD3_IBA1.tif]

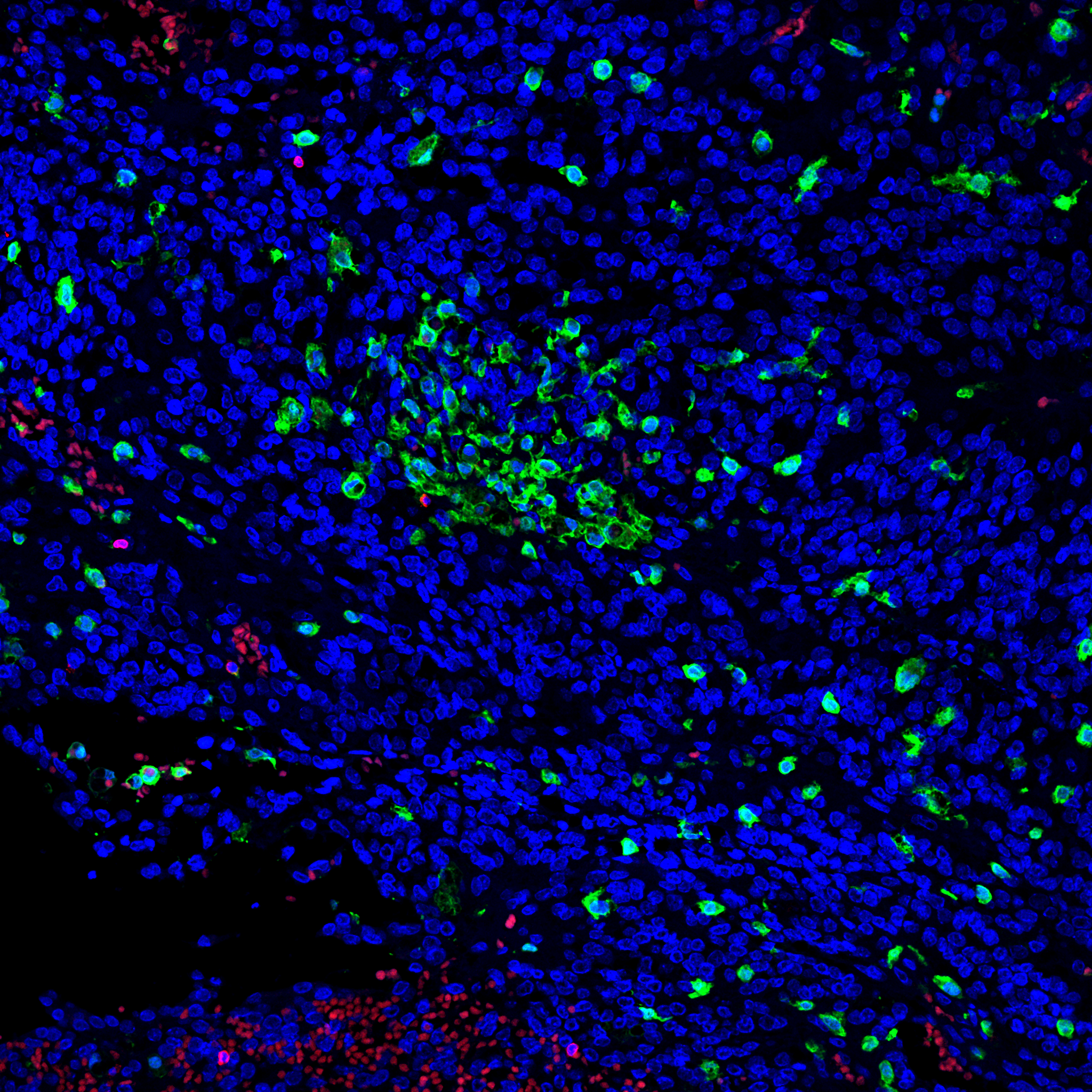

Supplement: Supplementary file 8 — Source Data for Figure 3 [file EMMM-15-e18199-s011.zip › Figure_3/3A/A'_Primary_T#1_IBA1,_CD3_merge.tif]

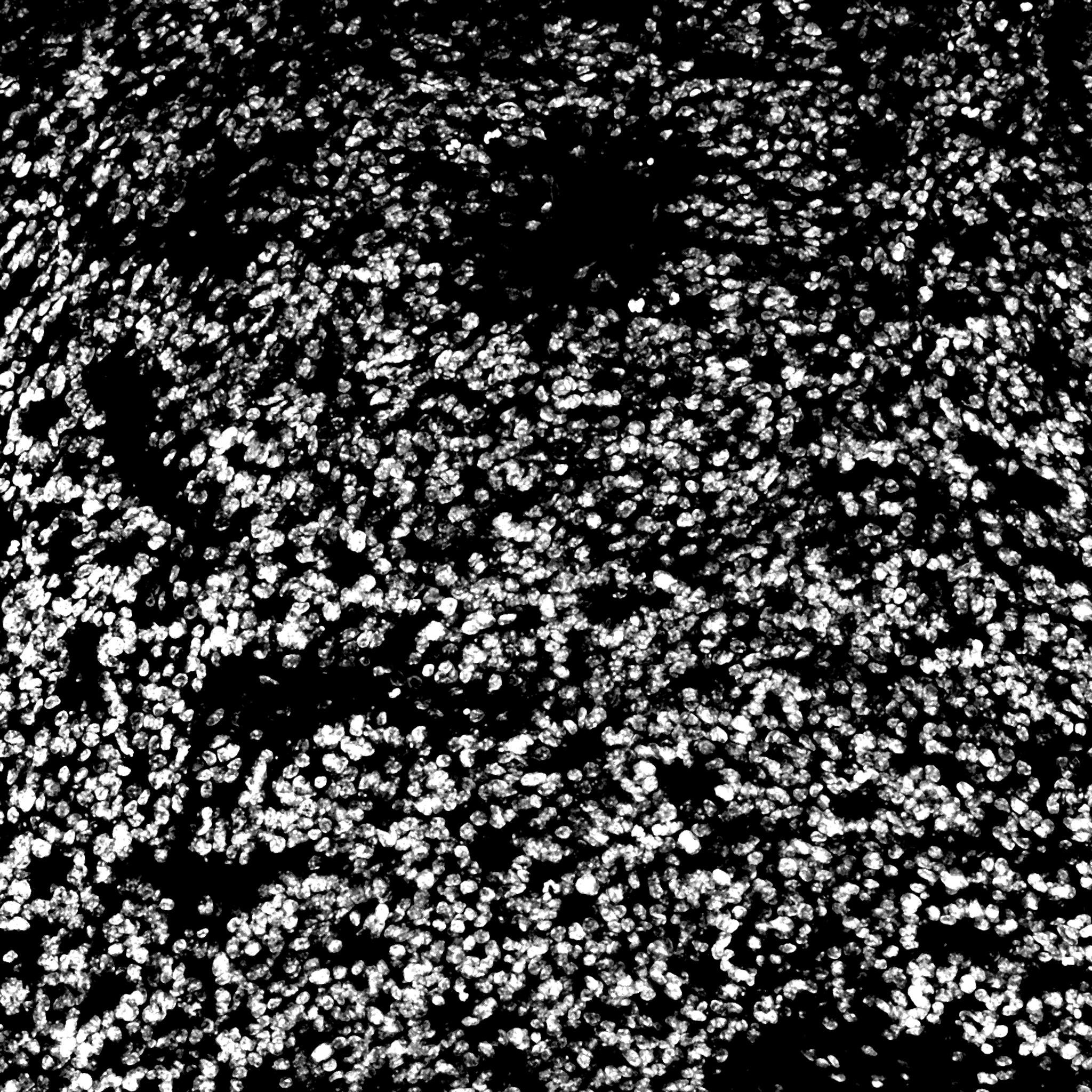

Supplement: Supplementary file 8 — Source Data for Figure 3 [file EMMM-15-e18199-s011.zip › Figure_3/3A/A'_Primary_T#1_Ki67,_SOX2_DAPI.tif]

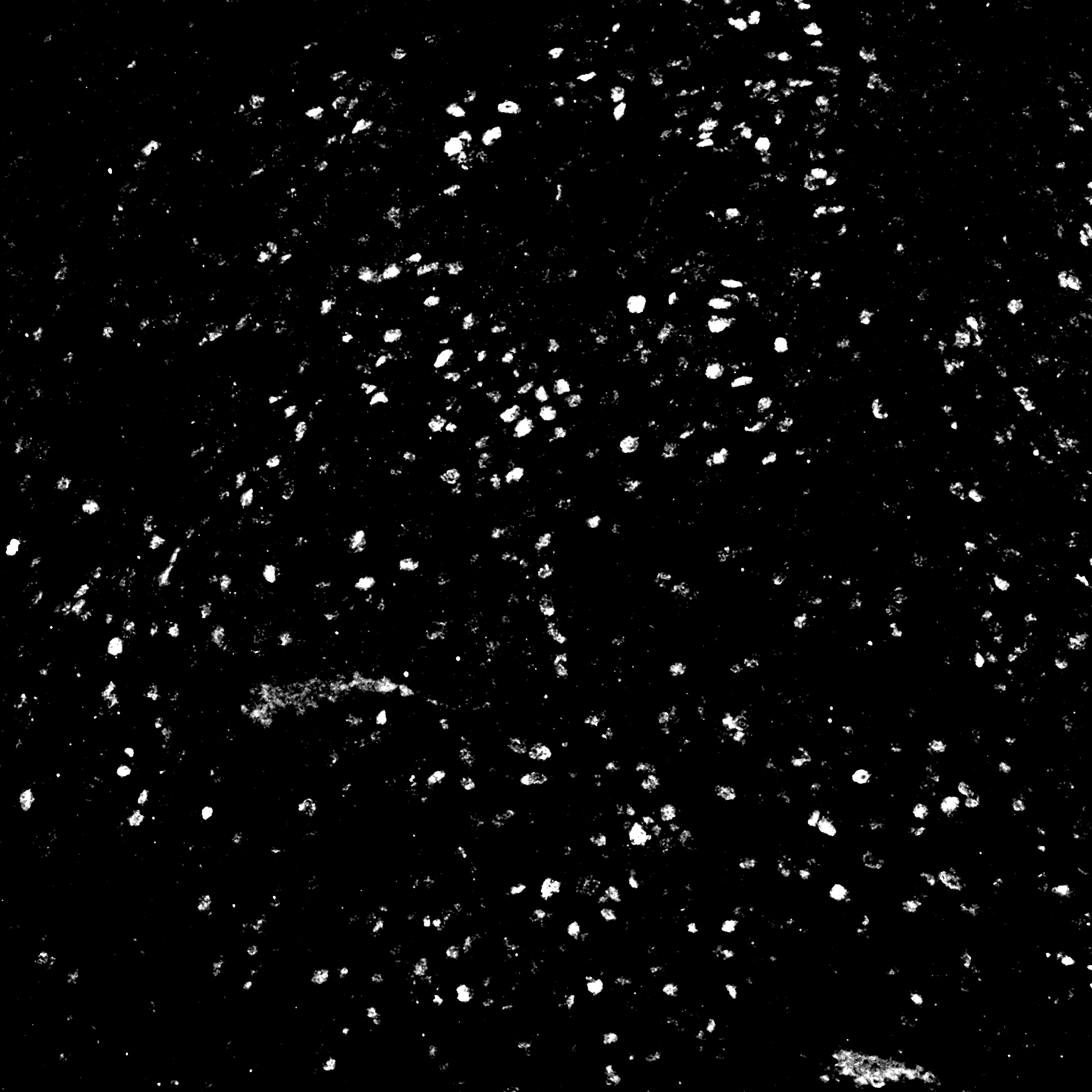

Supplement: Supplementary file 8 — Source Data for Figure 3 [file EMMM-15-e18199-s011.zip › Figure_3/3A/A'_Primary_T#1_Ki67,_SOX2_Ki67.tif]

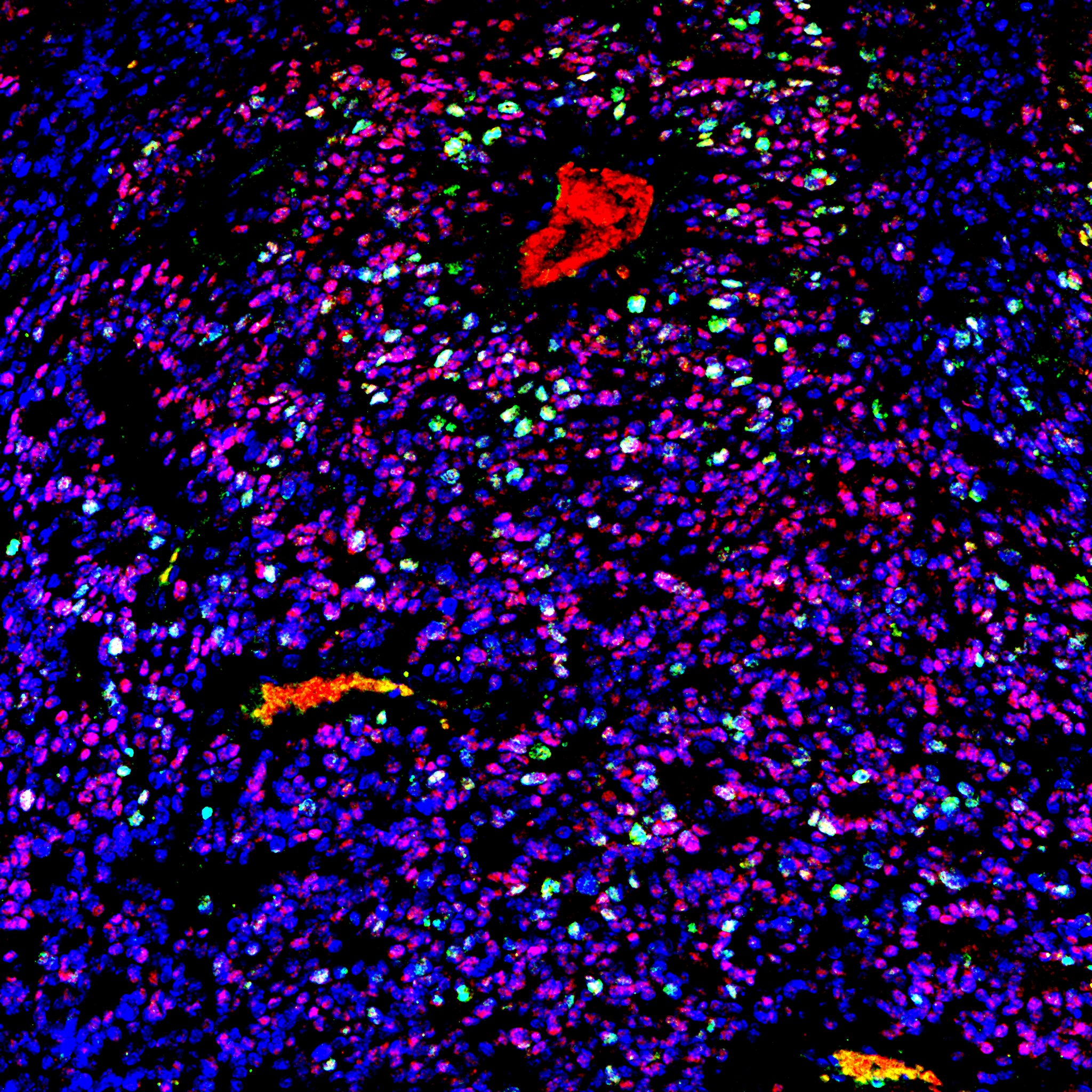

Supplement: Supplementary file 8 — Source Data for Figure 3 [file EMMM-15-e18199-s011.zip › Figure_3/3A/A'_Primary_T#1_Ki67,_SOX2_merge.tif]

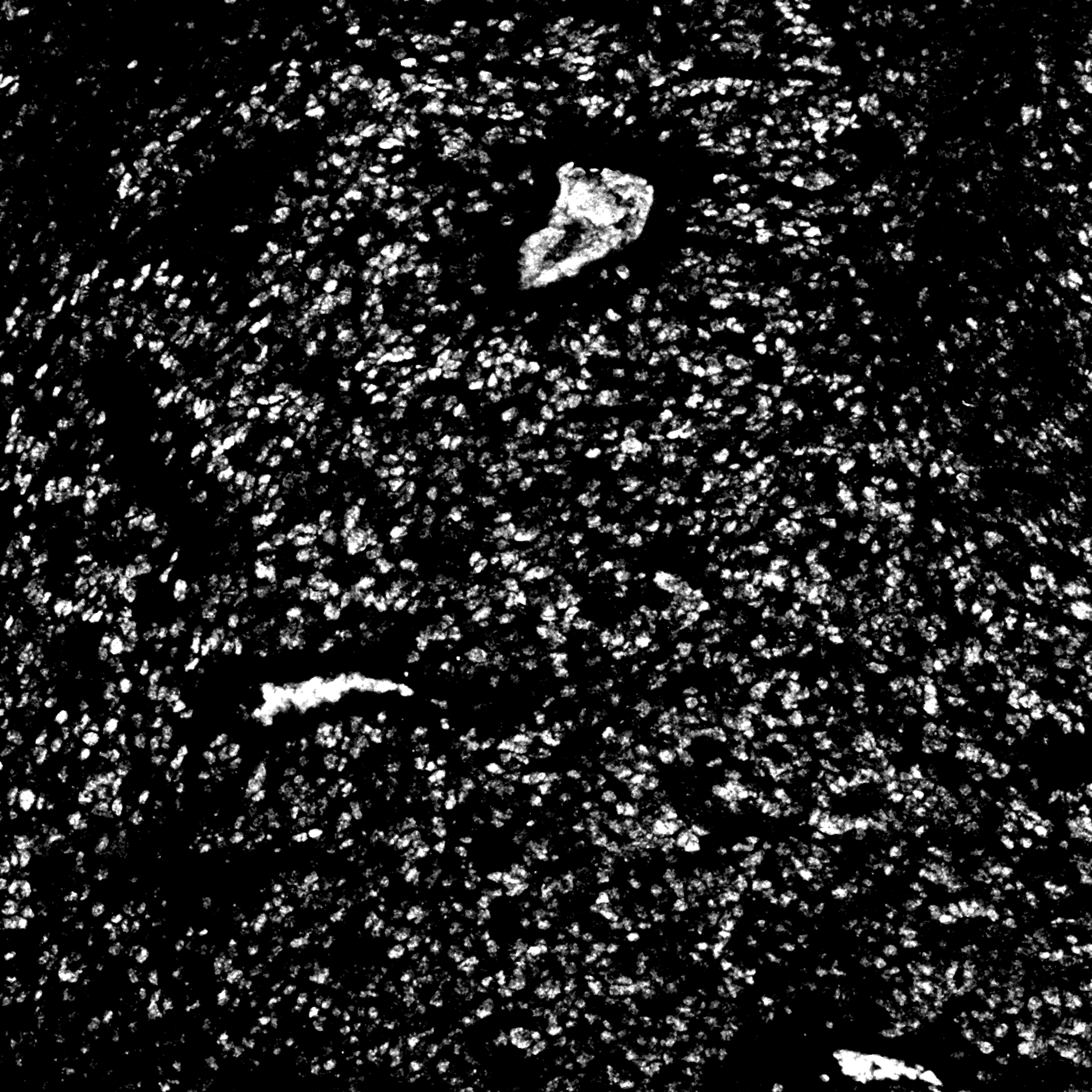

Supplement: Supplementary file 8 — Source Data for Figure 3 [file EMMM-15-e18199-s011.zip › Figure_3/3A/A'_Primary_T#1_Ki67,_SOX2_SOX2.tif]

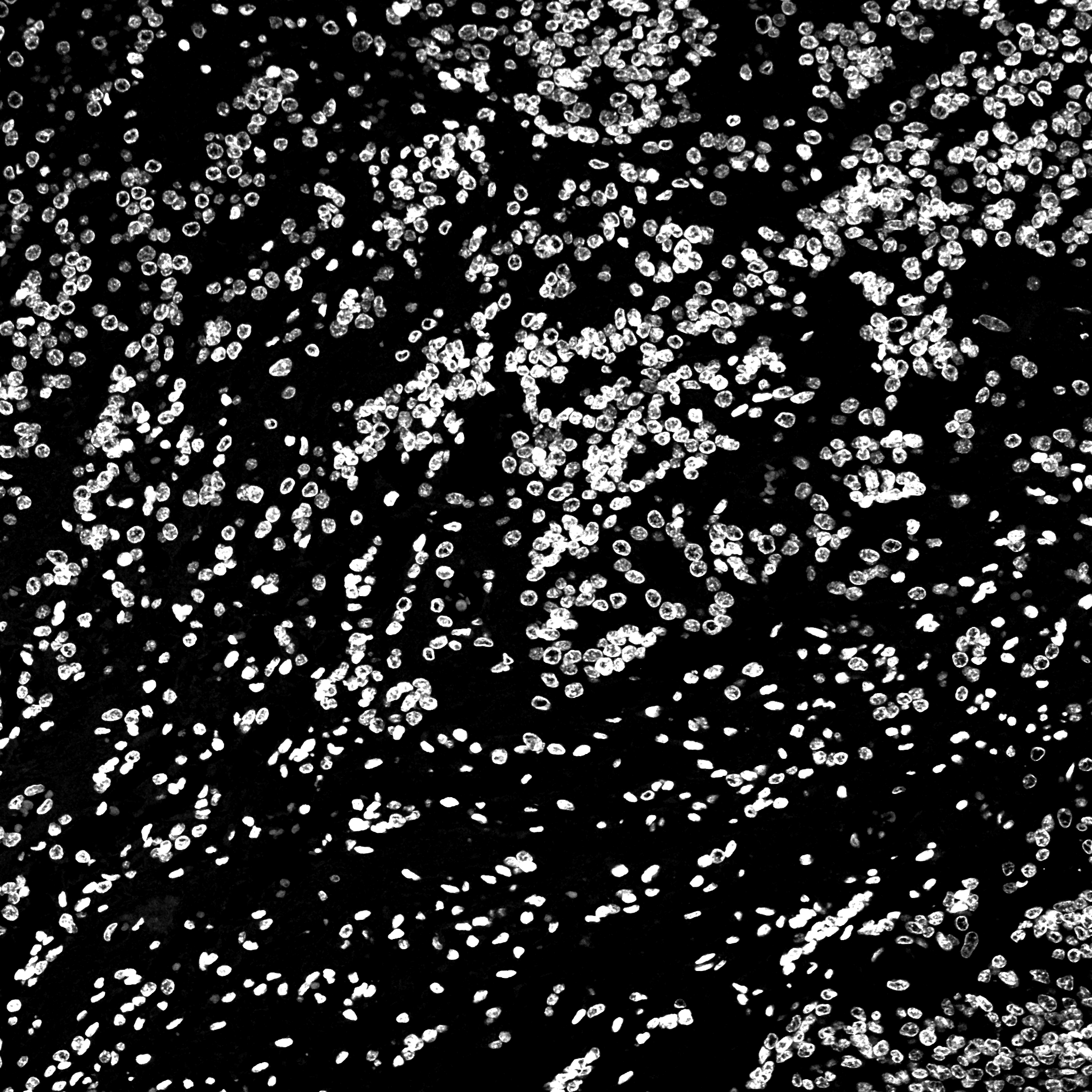

Supplement: Supplementary file 8 — Source Data for Figure 3 [file EMMM-15-e18199-s011.zip › Figure_3/3A/A'_Primary_T#1_OLIG2,_Nestin_DAPI.tif]

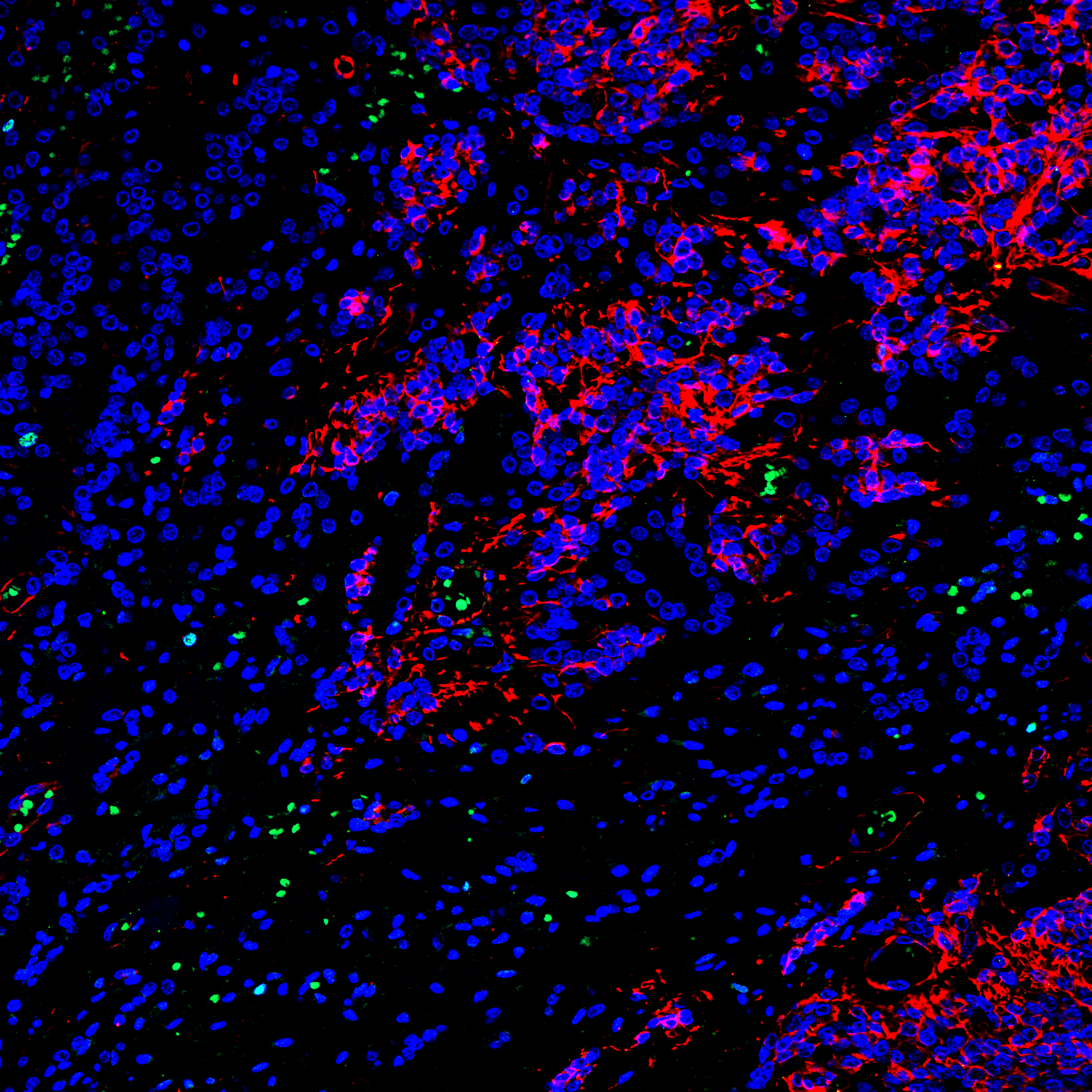

Supplement: Supplementary file 8 — Source Data for Figure 3 [file EMMM-15-e18199-s011.zip › Figure_3/3A/A'_Primary_T#1_OLIG2,_Nestin_merge.tif]

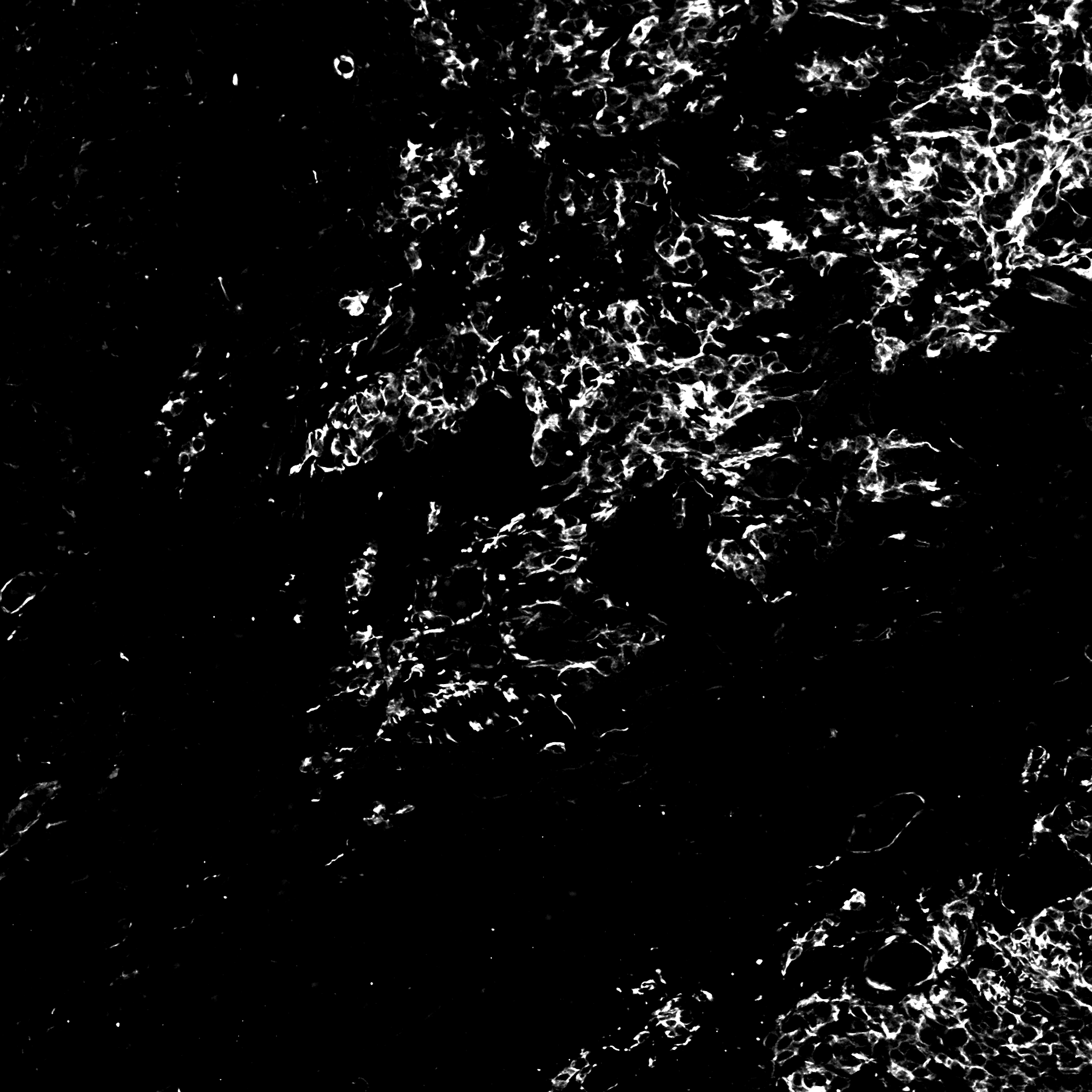

Supplement: Supplementary file 8 — Source Data for Figure 3 [file EMMM-15-e18199-s011.zip › Figure_3/3A/A'_Primary_T#1_OLIG2,_Nestin_Nestin.tif]

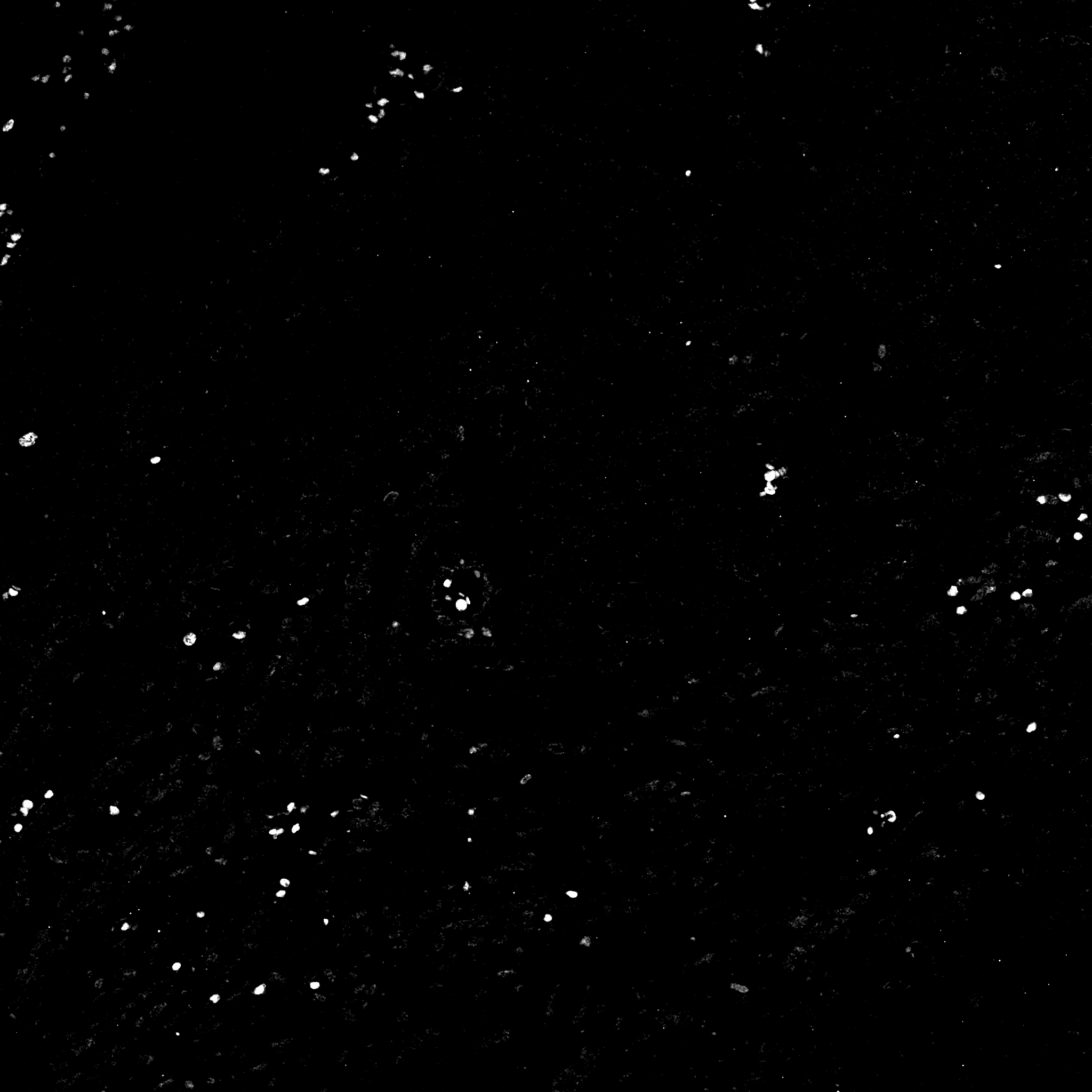

Supplement: Supplementary file 8 — Source Data for Figure 3 [file EMMM-15-e18199-s011.zip › Figure_3/3A/A'_Primary_T#1_OLIG2,_Nestin_OLIG2.tif]

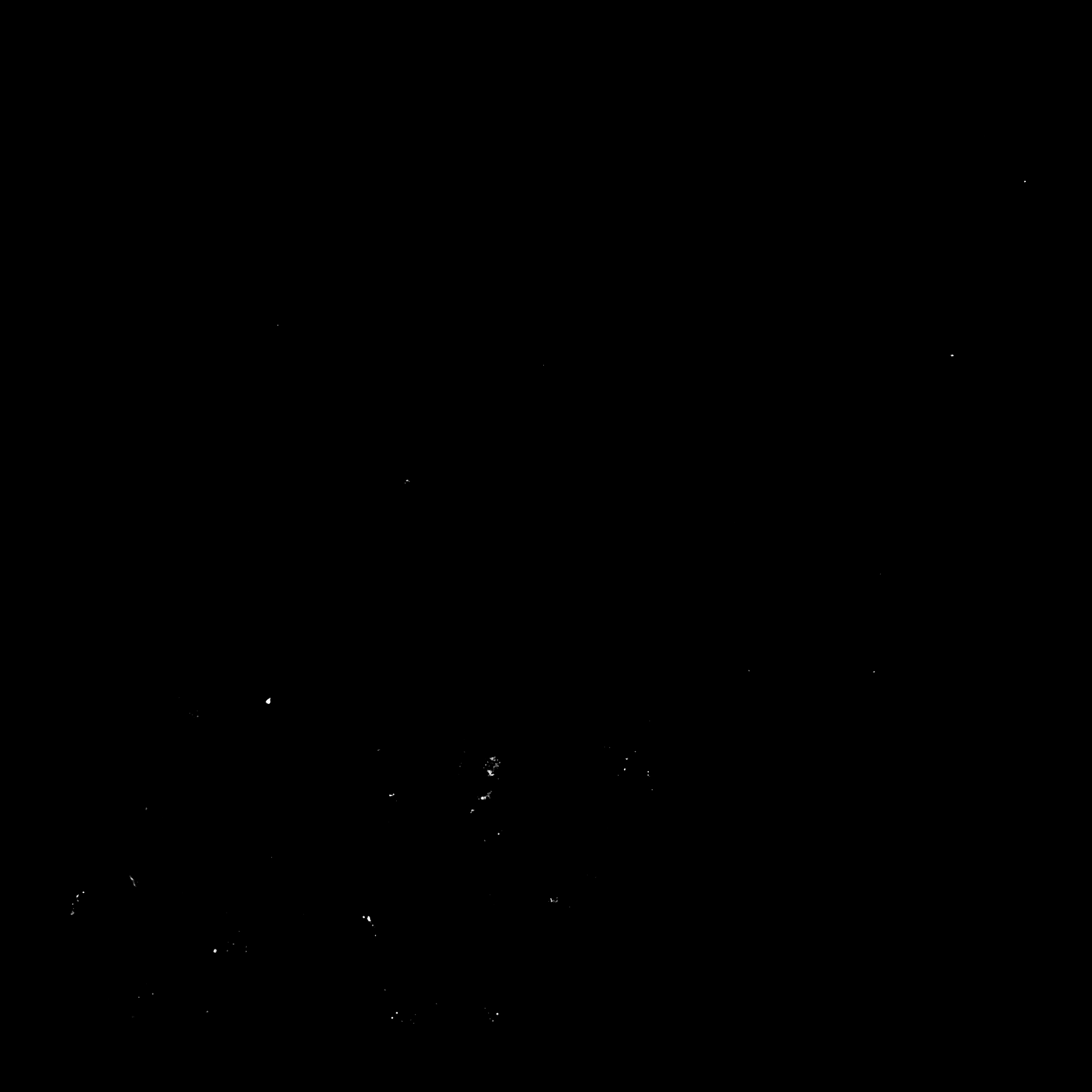

Supplement: Supplementary file 8 — Source Data for Figure 3 [file EMMM-15-e18199-s011.zip › Figure_3/3C/C'_PDO_T#9_D28_CD34,_CD3_CD3.tif]

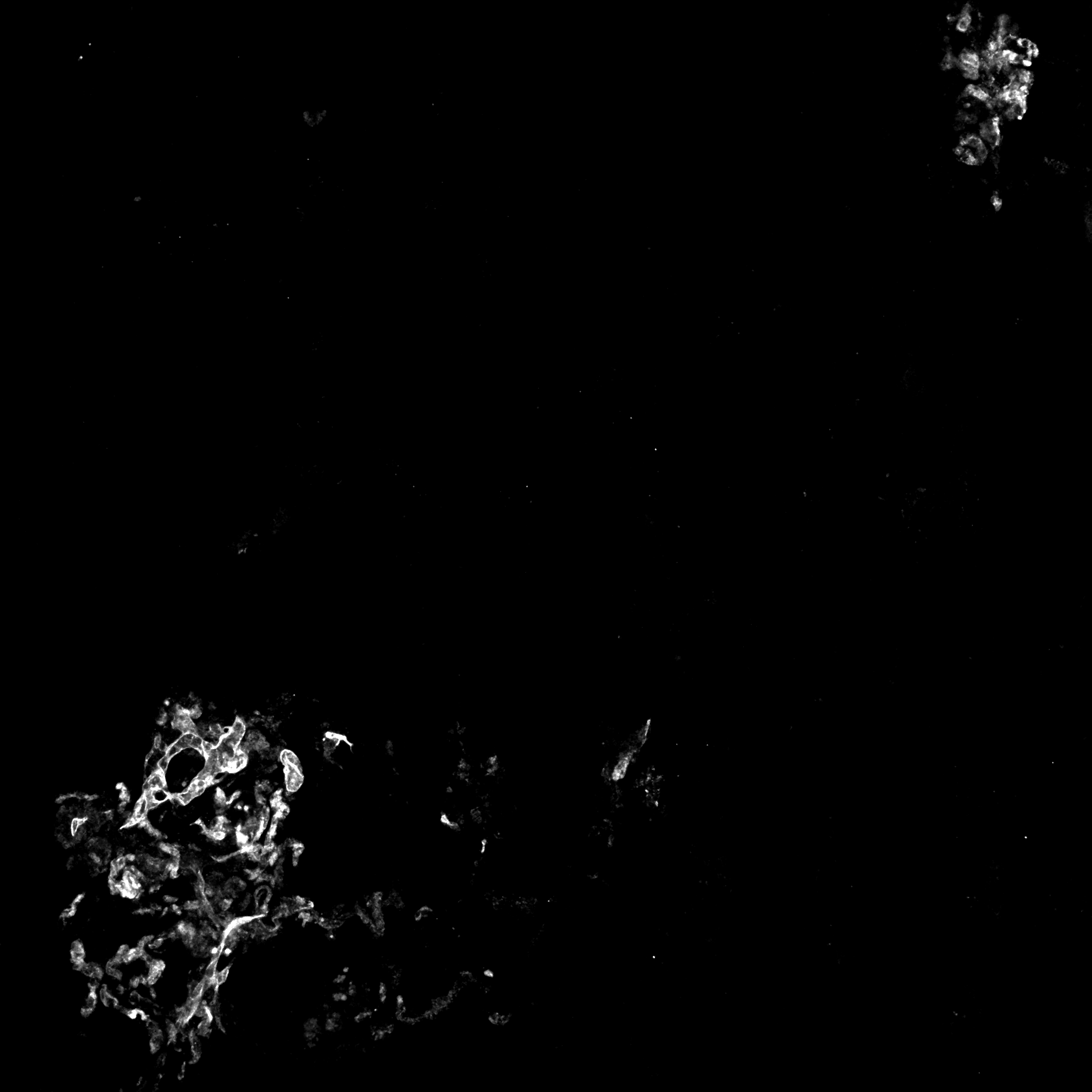

Supplement: Supplementary file 8 — Source Data for Figure 3 [file EMMM-15-e18199-s011.zip › Figure_3/3C/C'_PDO_T#9_D28_CD34,_CD3_CD34.tif]

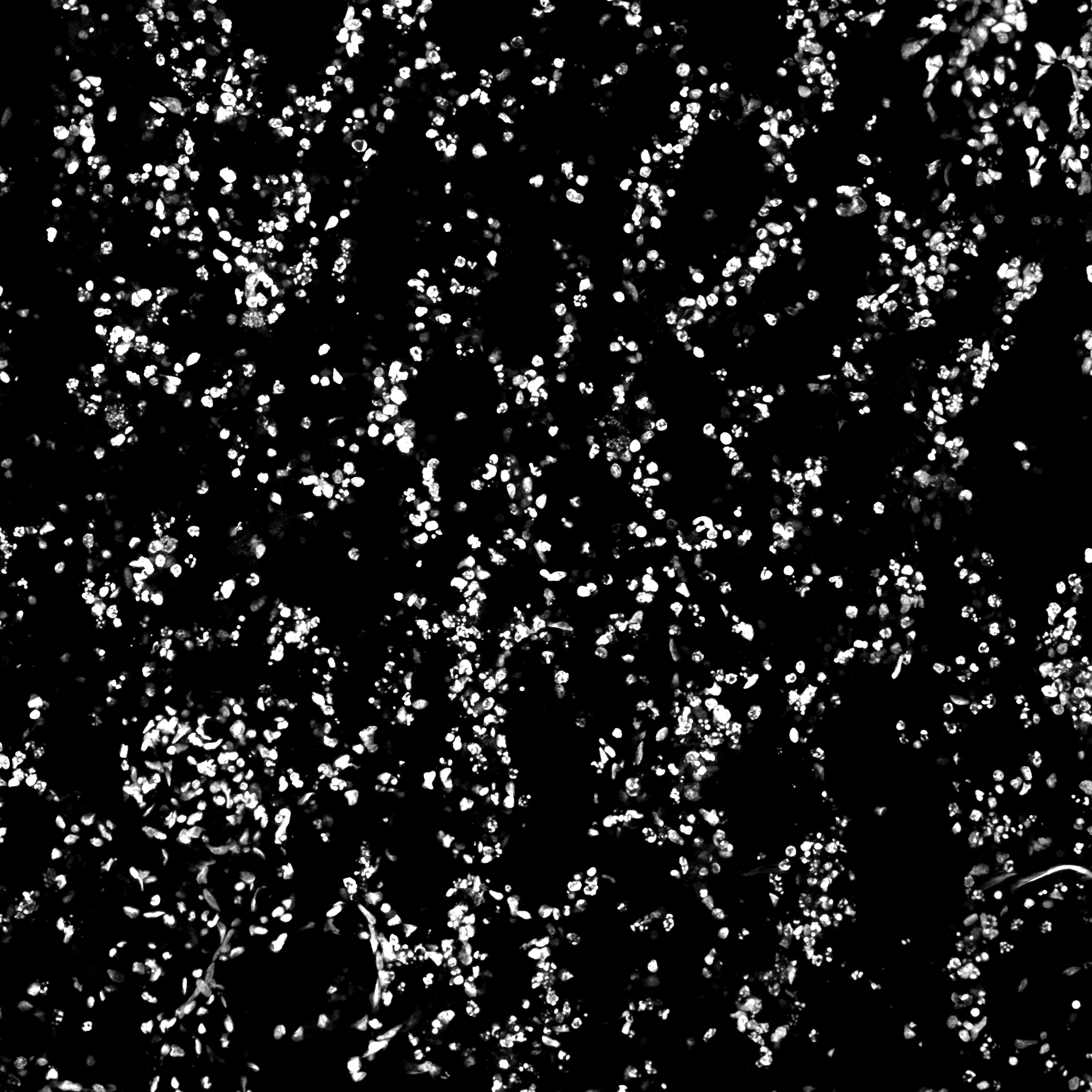

Supplement: Supplementary file 8 — Source Data for Figure 3 [file EMMM-15-e18199-s011.zip › Figure_3/3C/C'_PDO_T#9_D28_CD34,_CD3_DAPI.tif]

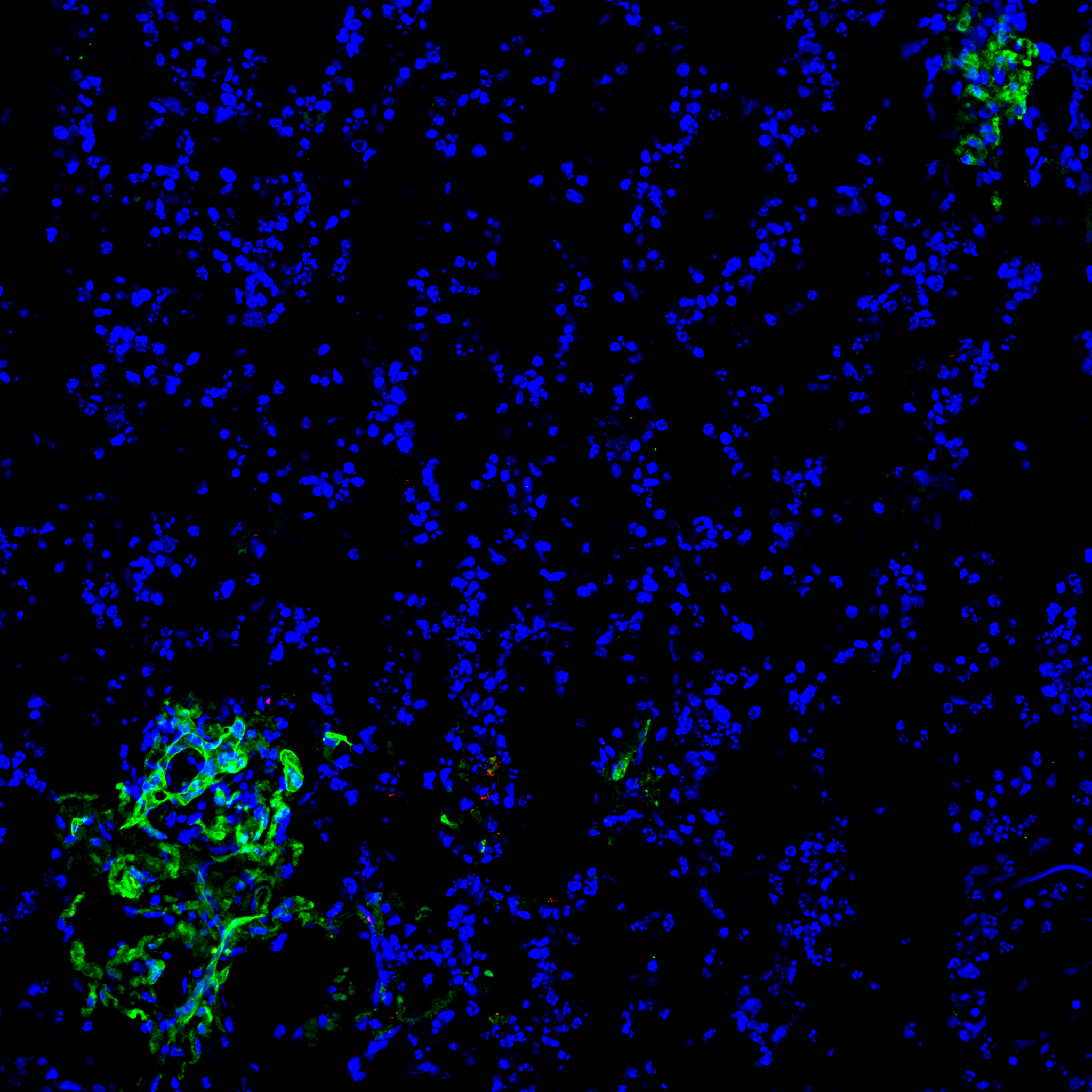

Supplement: Supplementary file 8 — Source Data for Figure 3 [file EMMM-15-e18199-s011.zip › Figure_3/3C/C'_PDO_T#9_D28_CD34,_CD3_merge.tif]

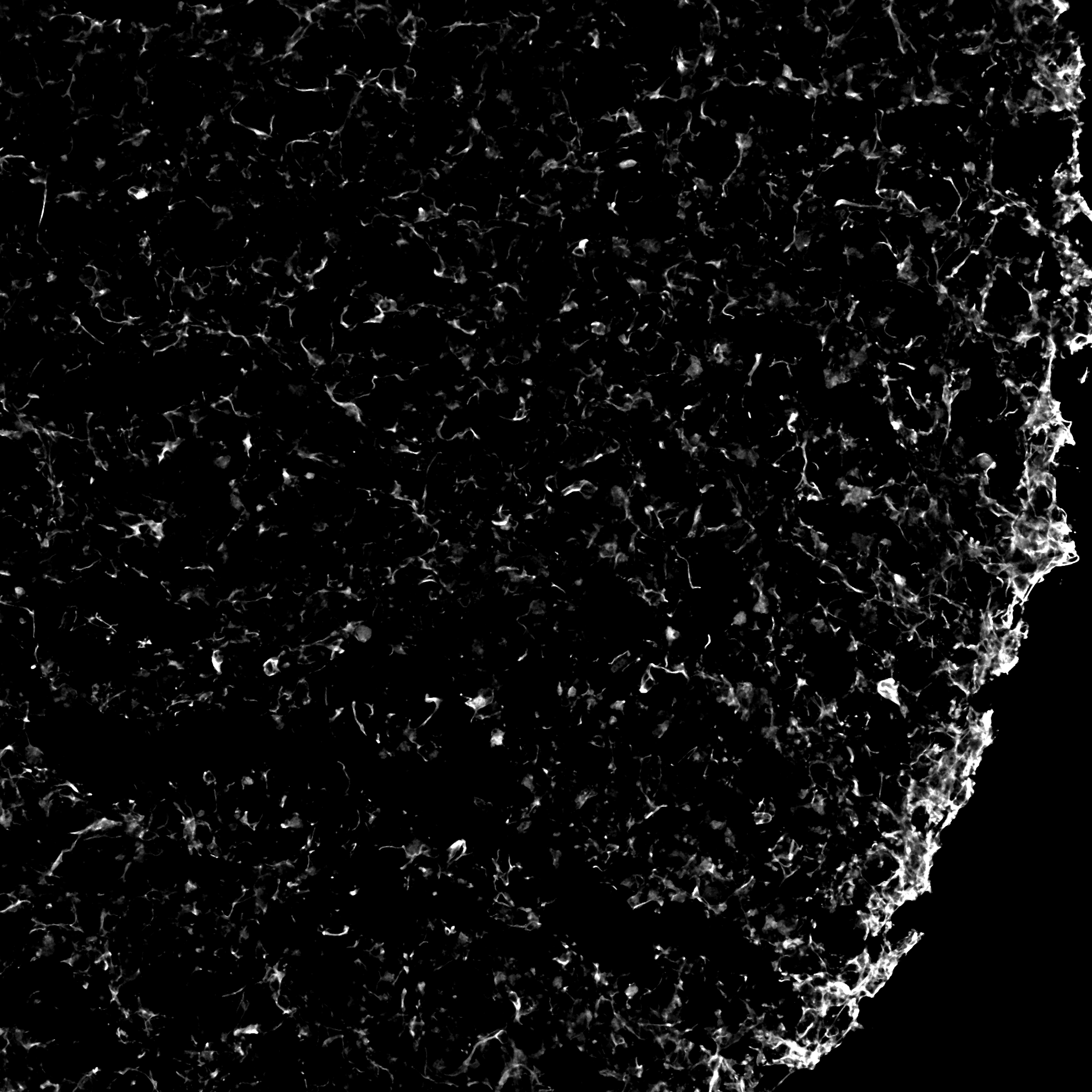

Supplement: Supplementary file 8 — Source Data for Figure 3 [file EMMM-15-e18199-s011.zip › Figure_3/3C/C'_PDO_T#9_D28_GFAP,_B3tubulin_B3tubulin.tif]

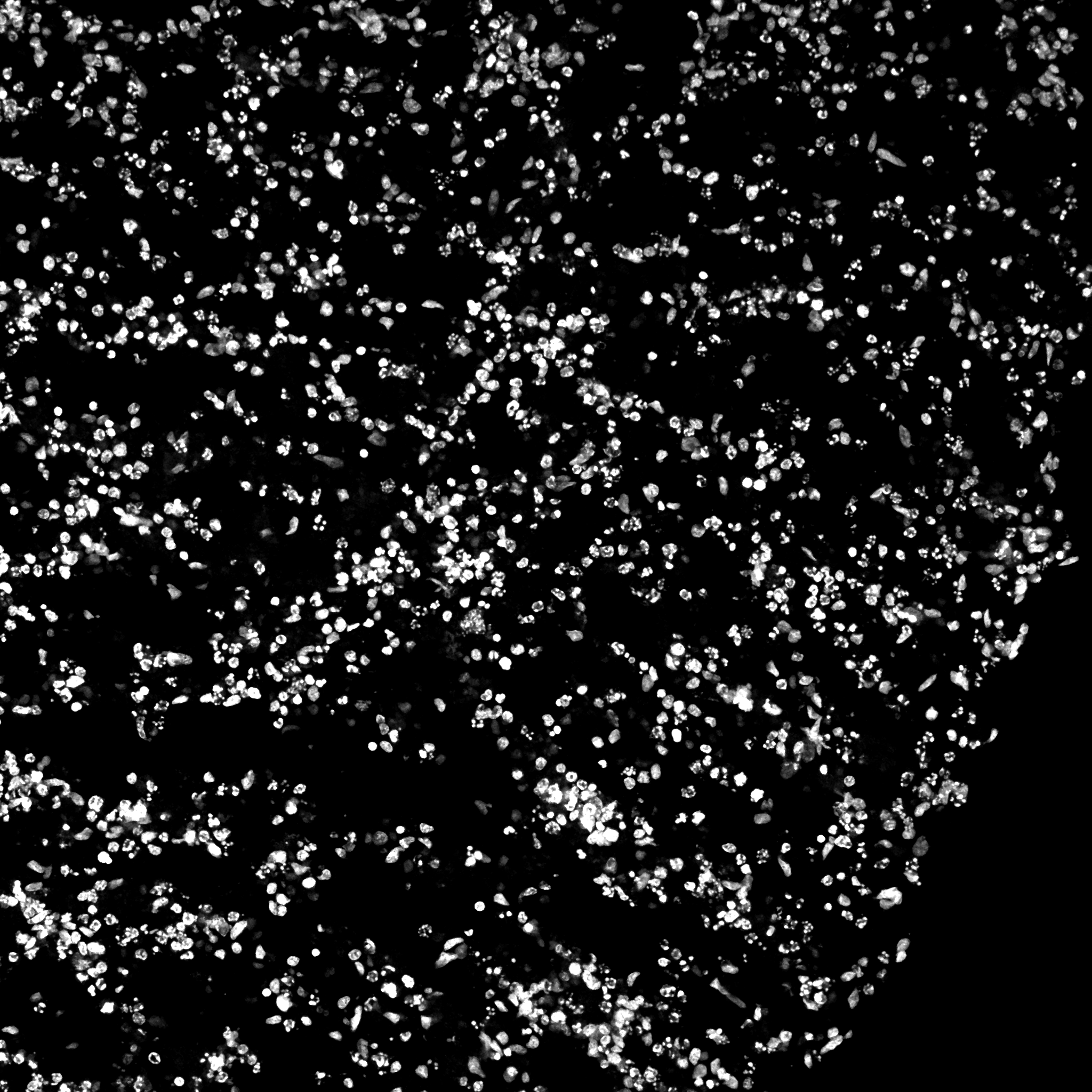

Supplement: Supplementary file 8 — Source Data for Figure 3 [file EMMM-15-e18199-s011.zip › Figure_3/3C/C'_PDO_T#9_D28_GFAP,_B3tubulin_DAPI.tif]

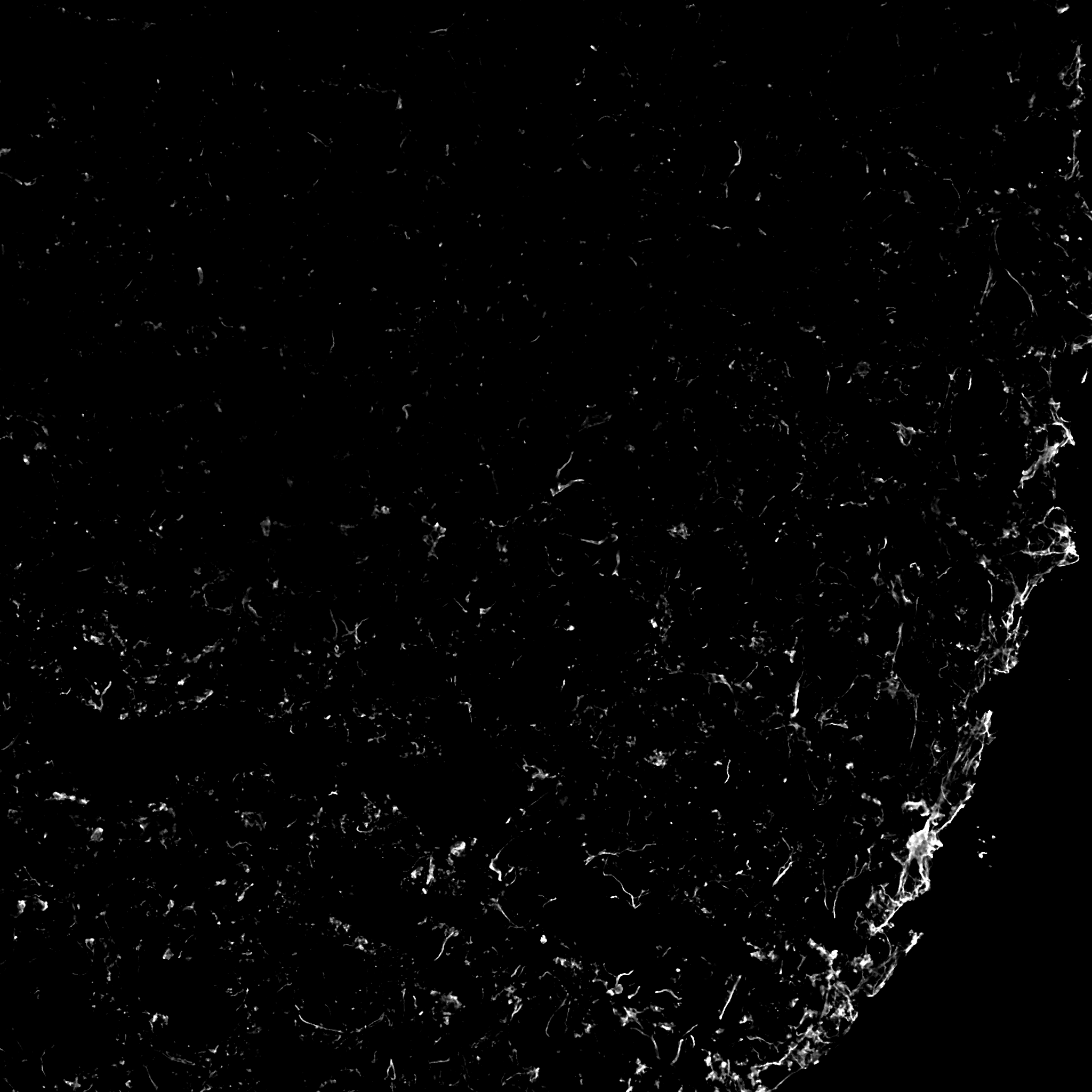

Supplement: Supplementary file 8 — Source Data for Figure 3 [file EMMM-15-e18199-s011.zip › Figure_3/3C/C'_PDO_T#9_D28_GFAP,_B3tubulin_GFAP.tif]

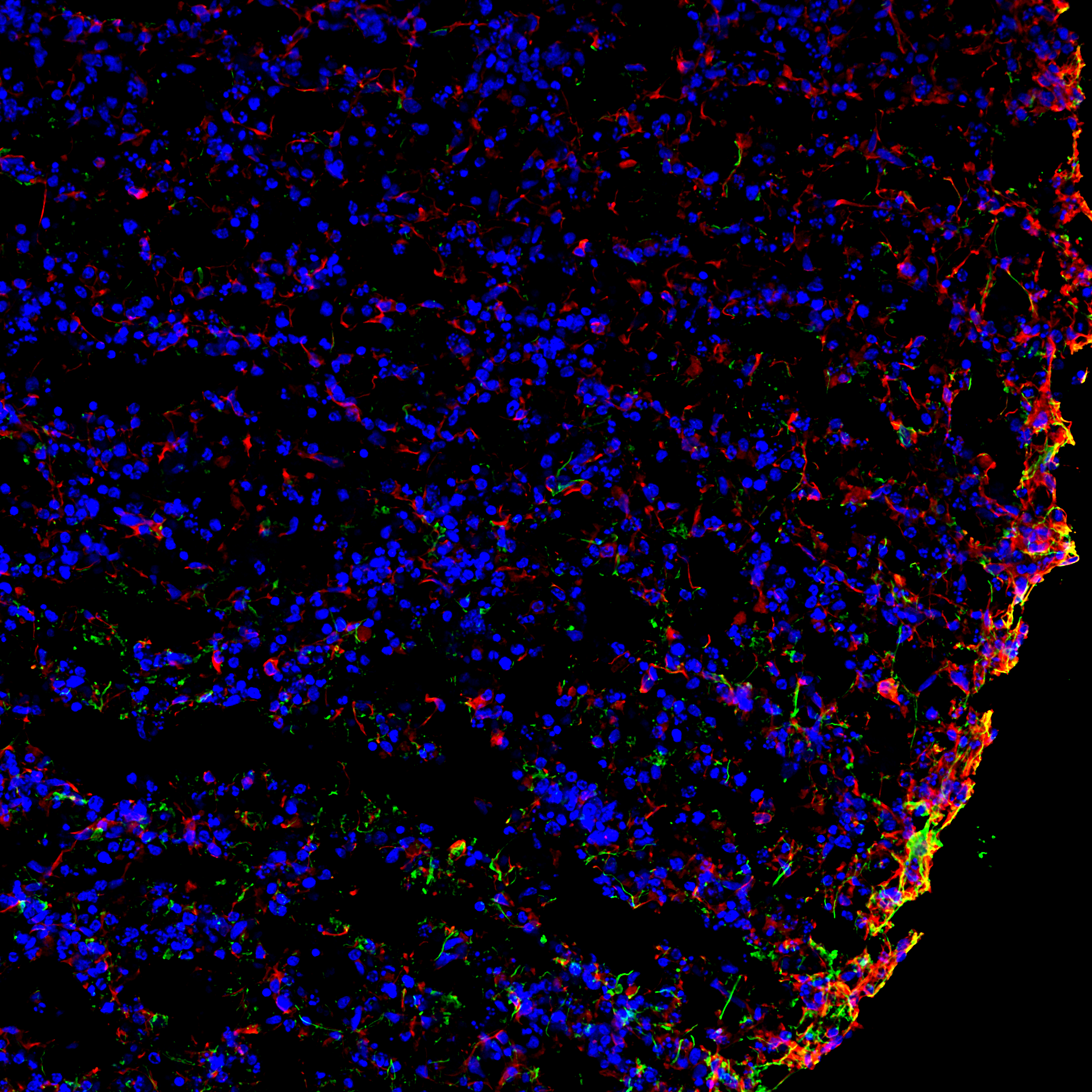

Supplement: Supplementary file 8 — Source Data for Figure 3 [file EMMM-15-e18199-s011.zip › Figure_3/3C/C'_PDO_T#9_D28_GFAP,_B3tubulin_merge.tif]

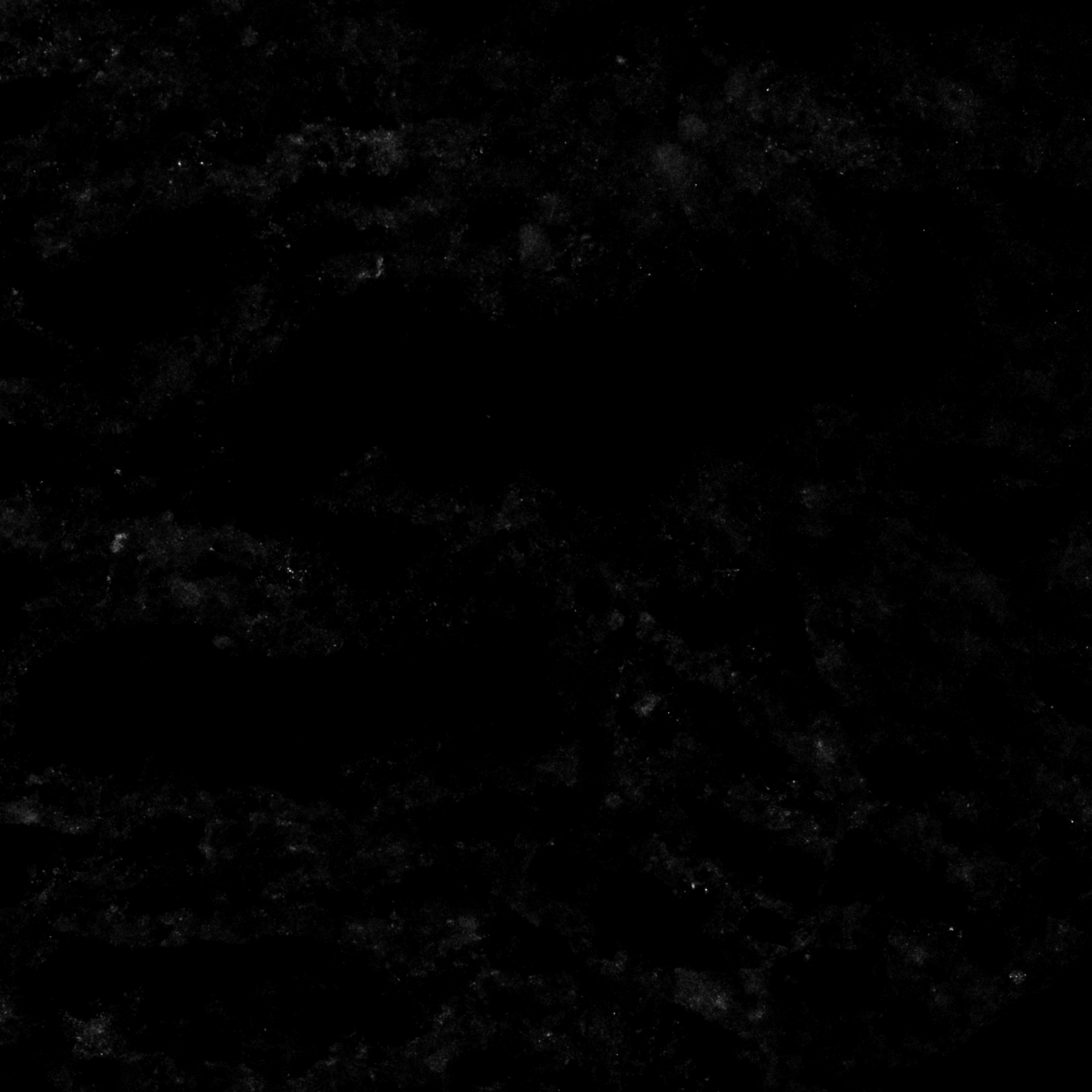

Supplement: Supplementary file 8 — Source Data for Figure 3 [file EMMM-15-e18199-s011.zip › Figure_3/3C/C'_PDO_T#9_D28_IBA1,_CD3_CD3.tif]

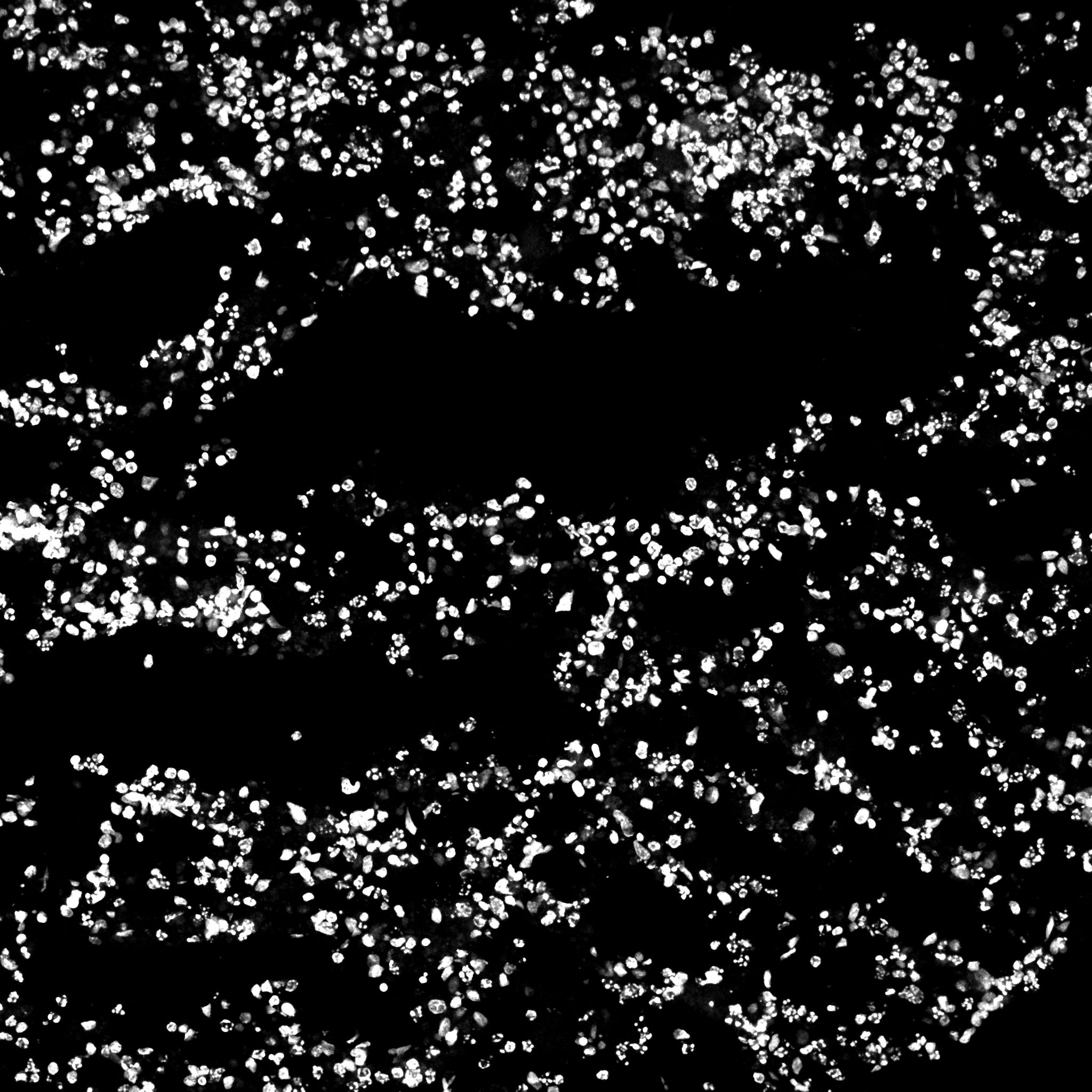

Supplement: Supplementary file 8 — Source Data for Figure 3 [file EMMM-15-e18199-s011.zip › Figure_3/3C/C'_PDO_T#9_D28_IBA1,_CD3_DAPI.tif]

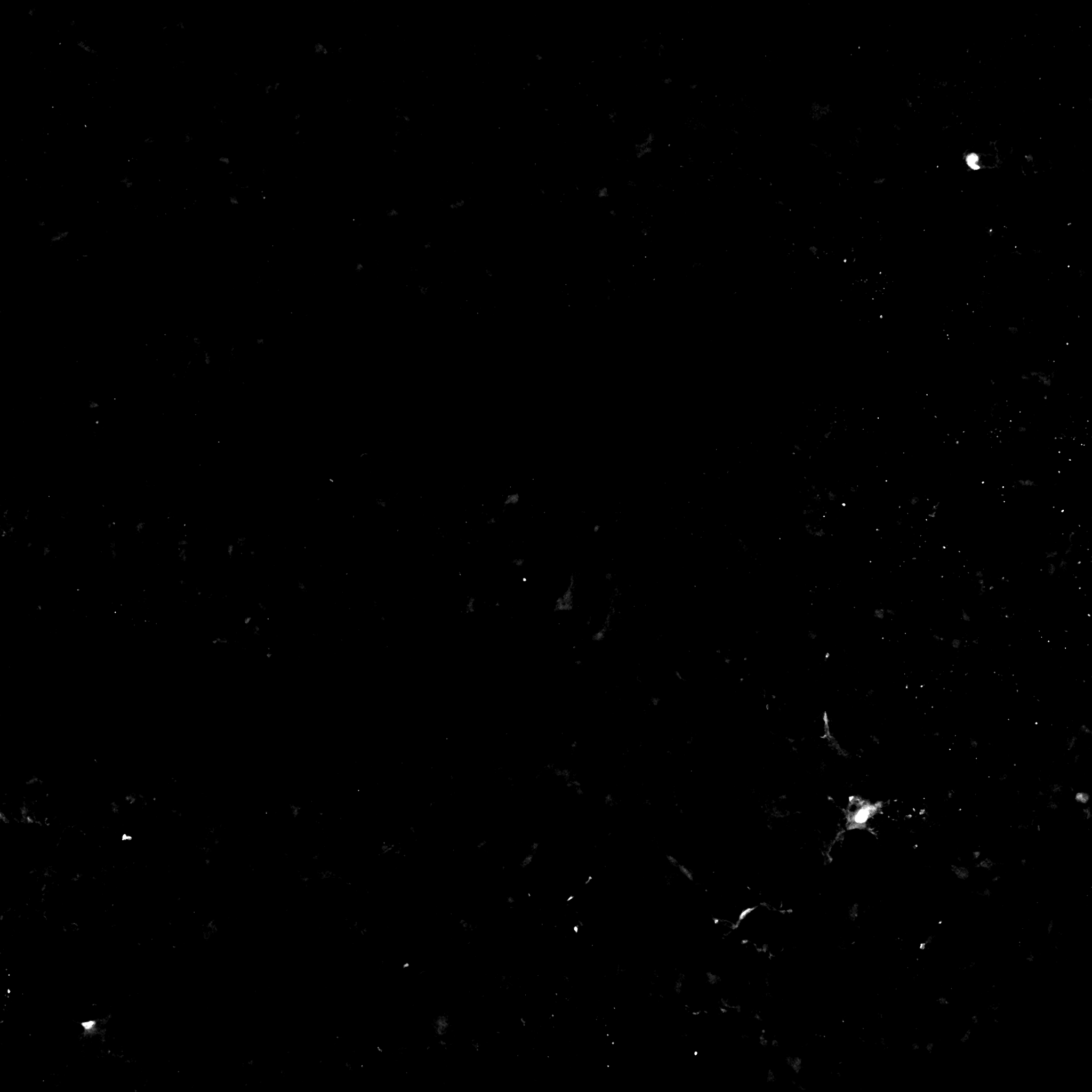

Supplement: Supplementary file 8 — Source Data for Figure 3 [file EMMM-15-e18199-s011.zip › Figure_3/3C/C'_PDO_T#9_D28_IBA1,_CD3_IBA1.tif]

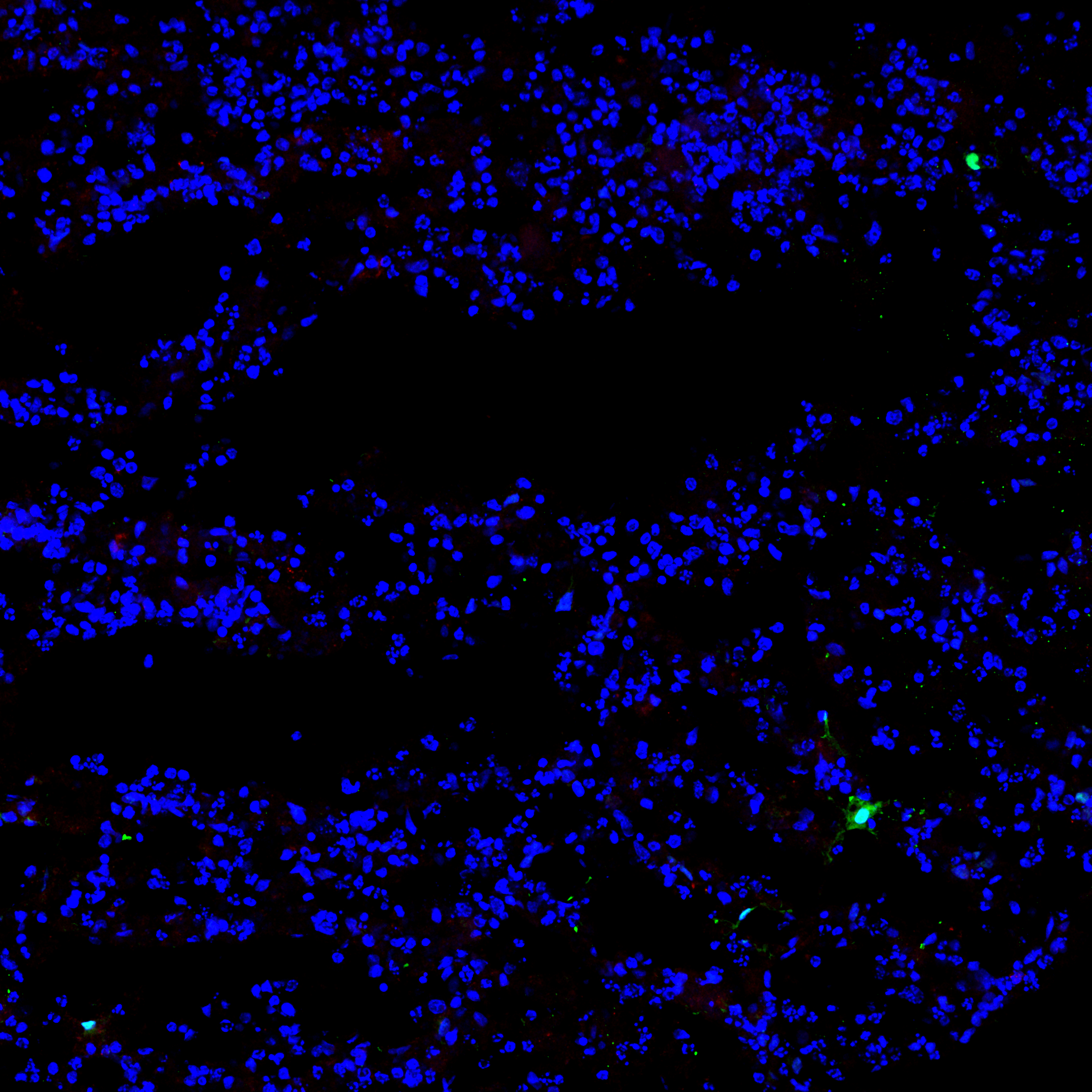

Supplement: Supplementary file 8 — Source Data for Figure 3 [file EMMM-15-e18199-s011.zip › Figure_3/3C/C'_PDO_T#9_D28_IBA1,_CD3_merge.tif]

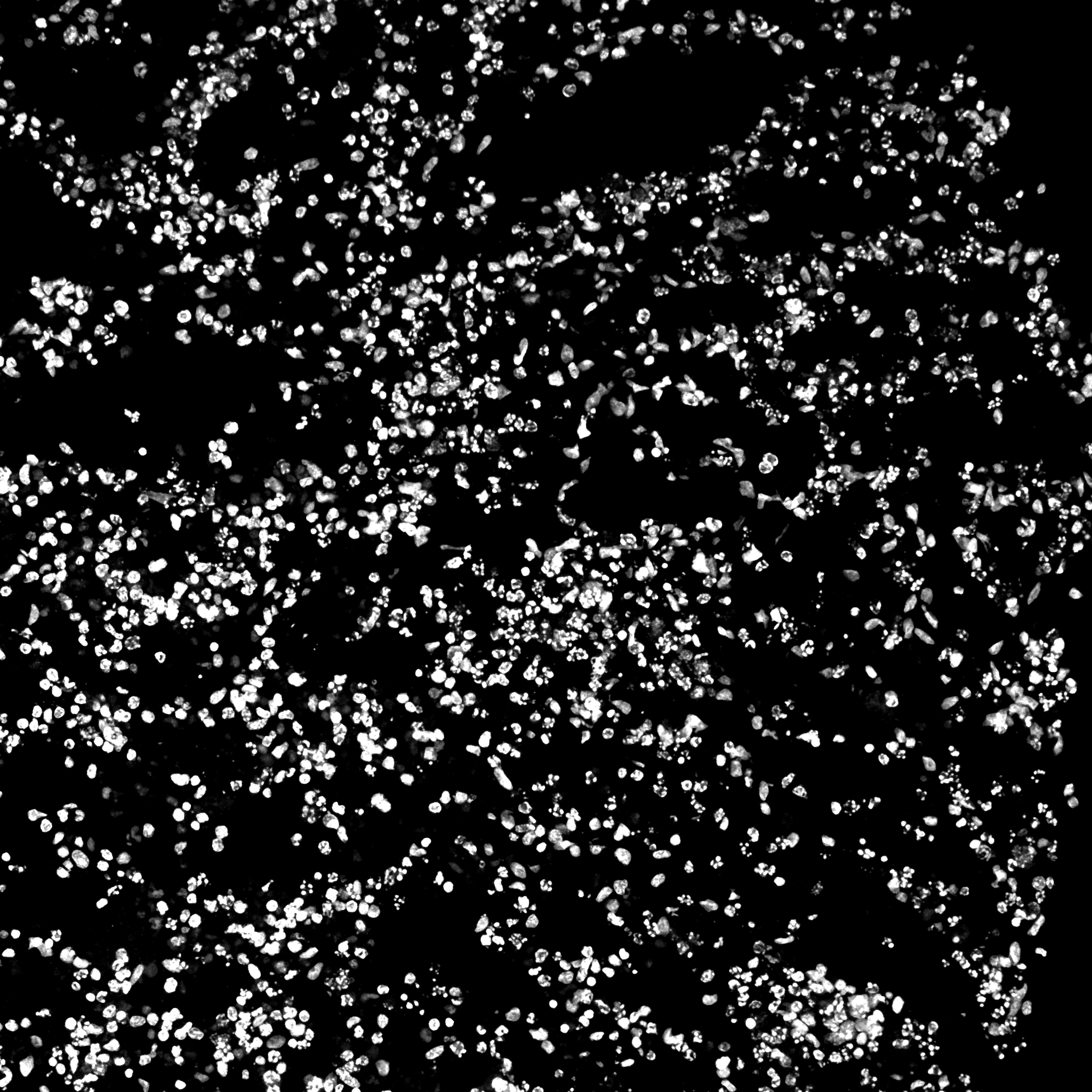

Supplement: Supplementary file 8 — Source Data for Figure 3 [file EMMM-15-e18199-s011.zip › Figure_3/3C/C'_PDO_T#9_D28_Ki67,_SOX2_DAPI.tif]

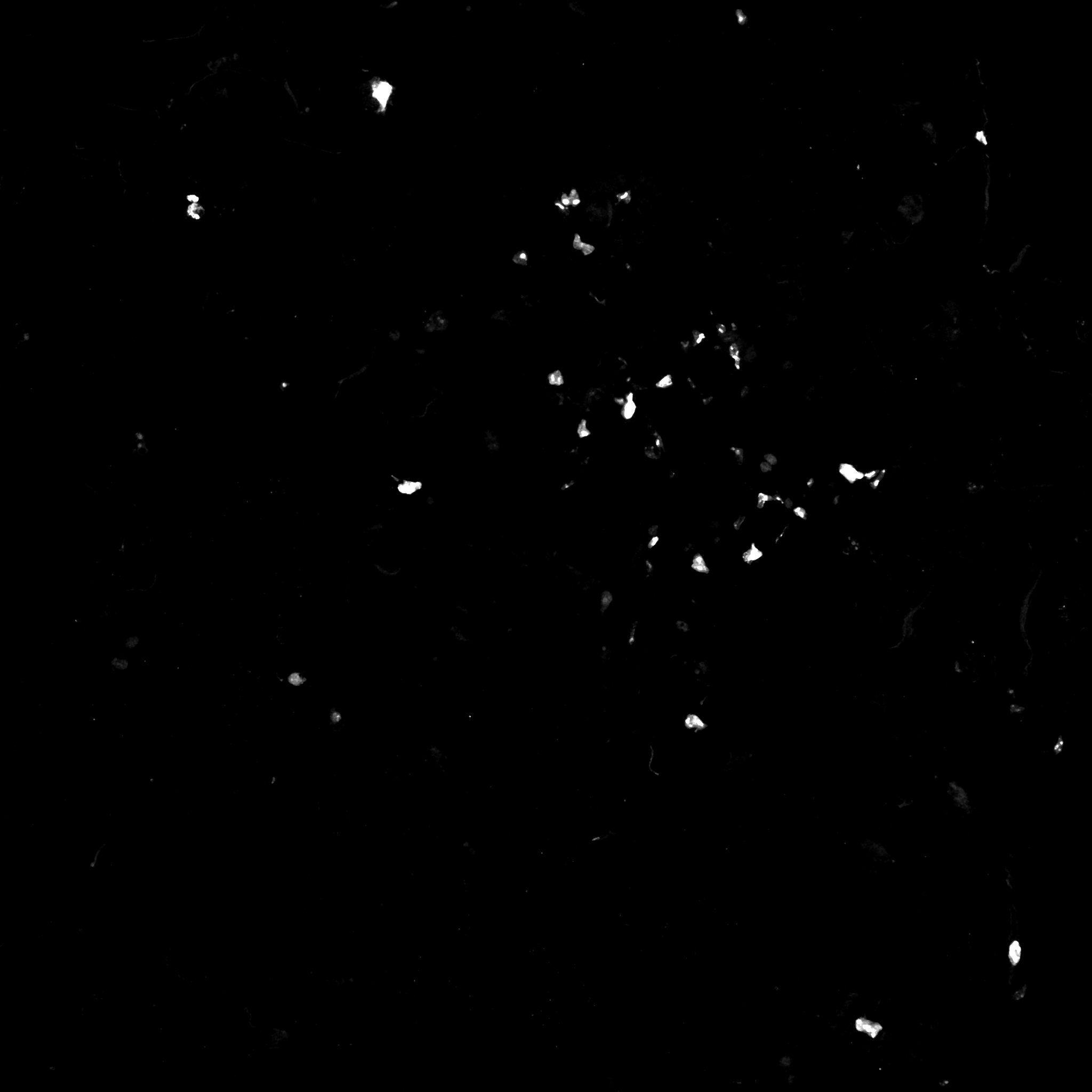

Supplement: Supplementary file 8 — Source Data for Figure 3 [file EMMM-15-e18199-s011.zip › Figure_3/3C/C'_PDO_T#9_D28_Ki67,_SOX2_Ki67.tif]

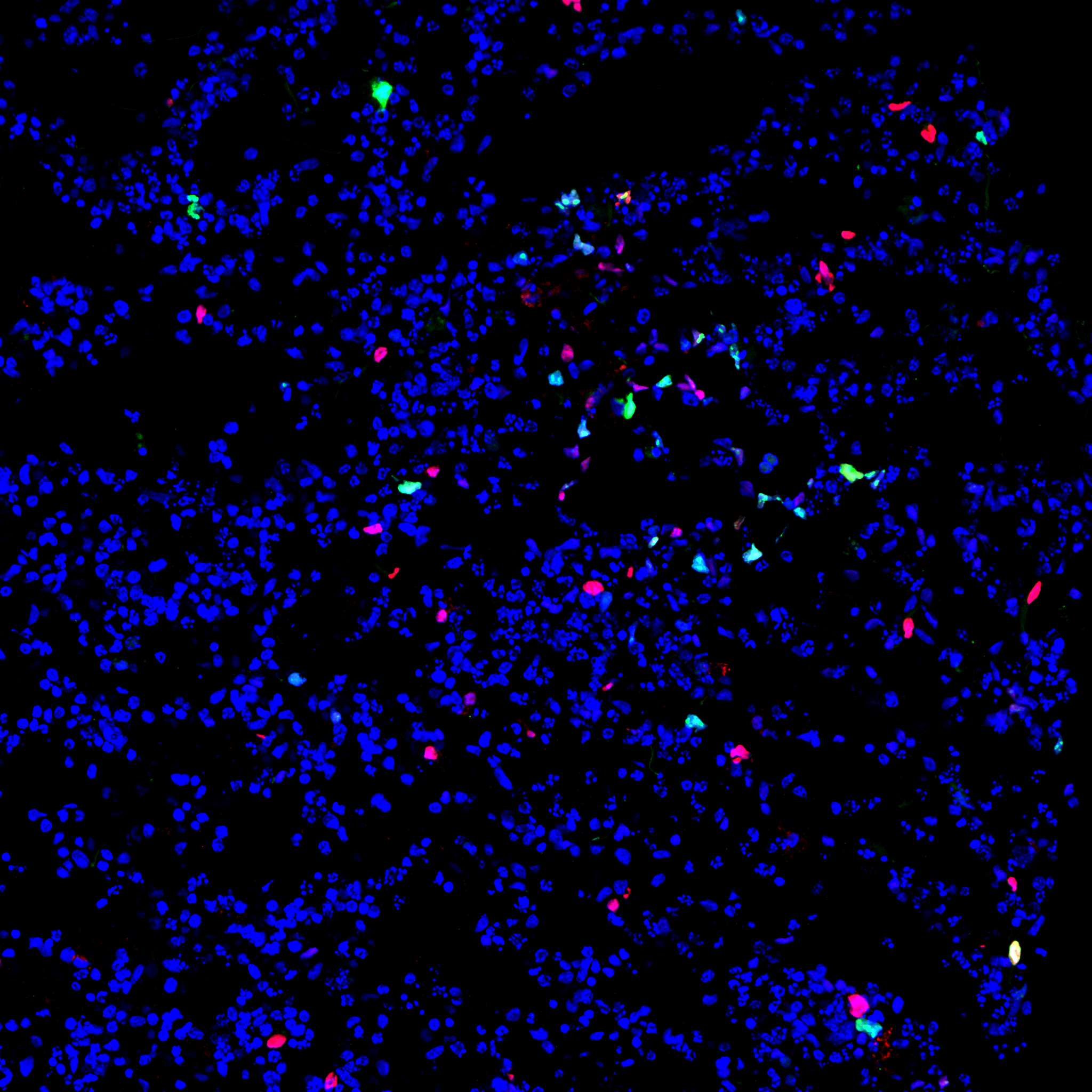

Supplement: Supplementary file 8 — Source Data for Figure 3 [file EMMM-15-e18199-s011.zip › Figure_3/3C/C'_PDO_T#9_D28_Ki67,_SOX2_merge.tif]

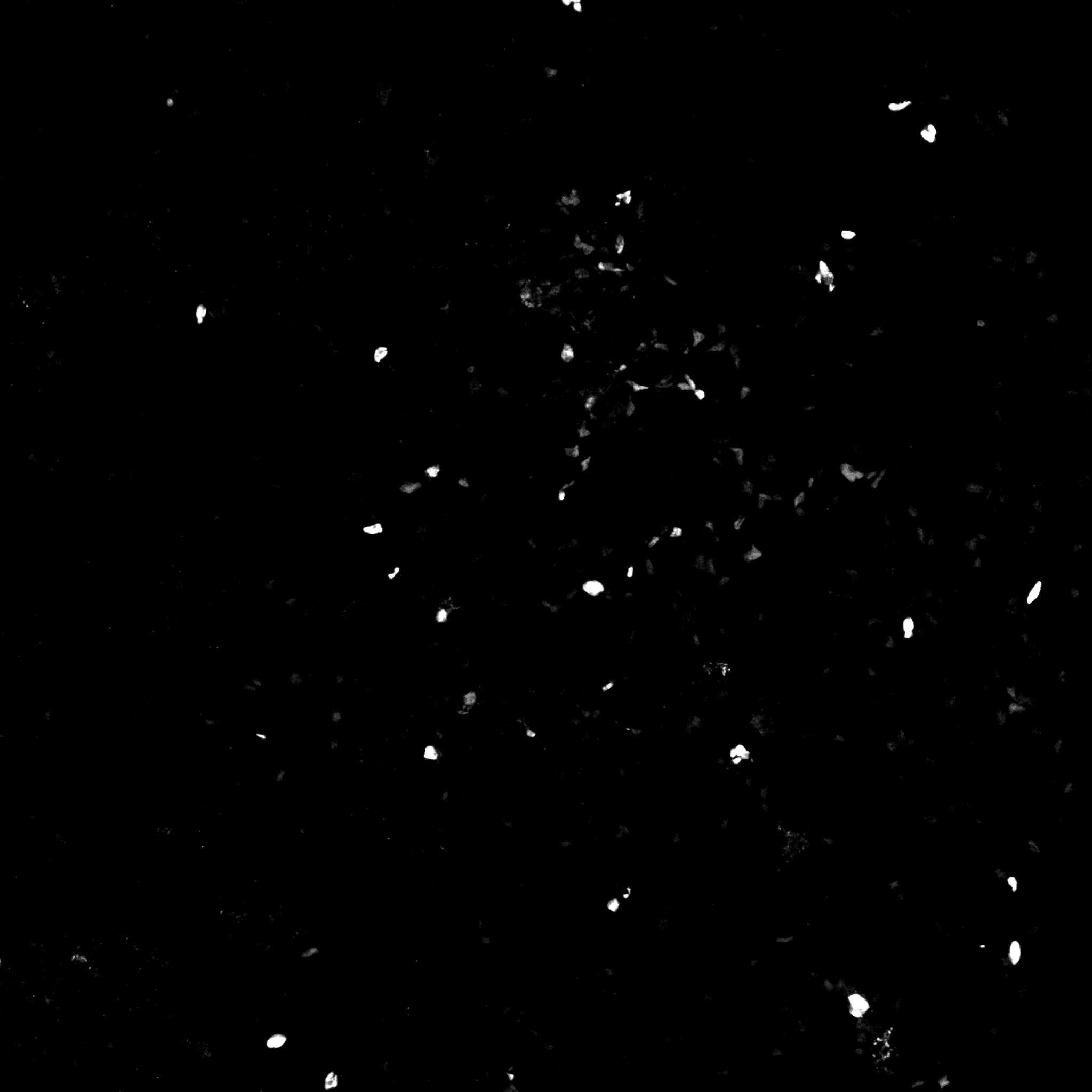

Supplement: Supplementary file 8 — Source Data for Figure 3 [file EMMM-15-e18199-s011.zip › Figure_3/3C/C'_PDO_T#9_D28_Ki67,_SOX2_SOX2.tif]

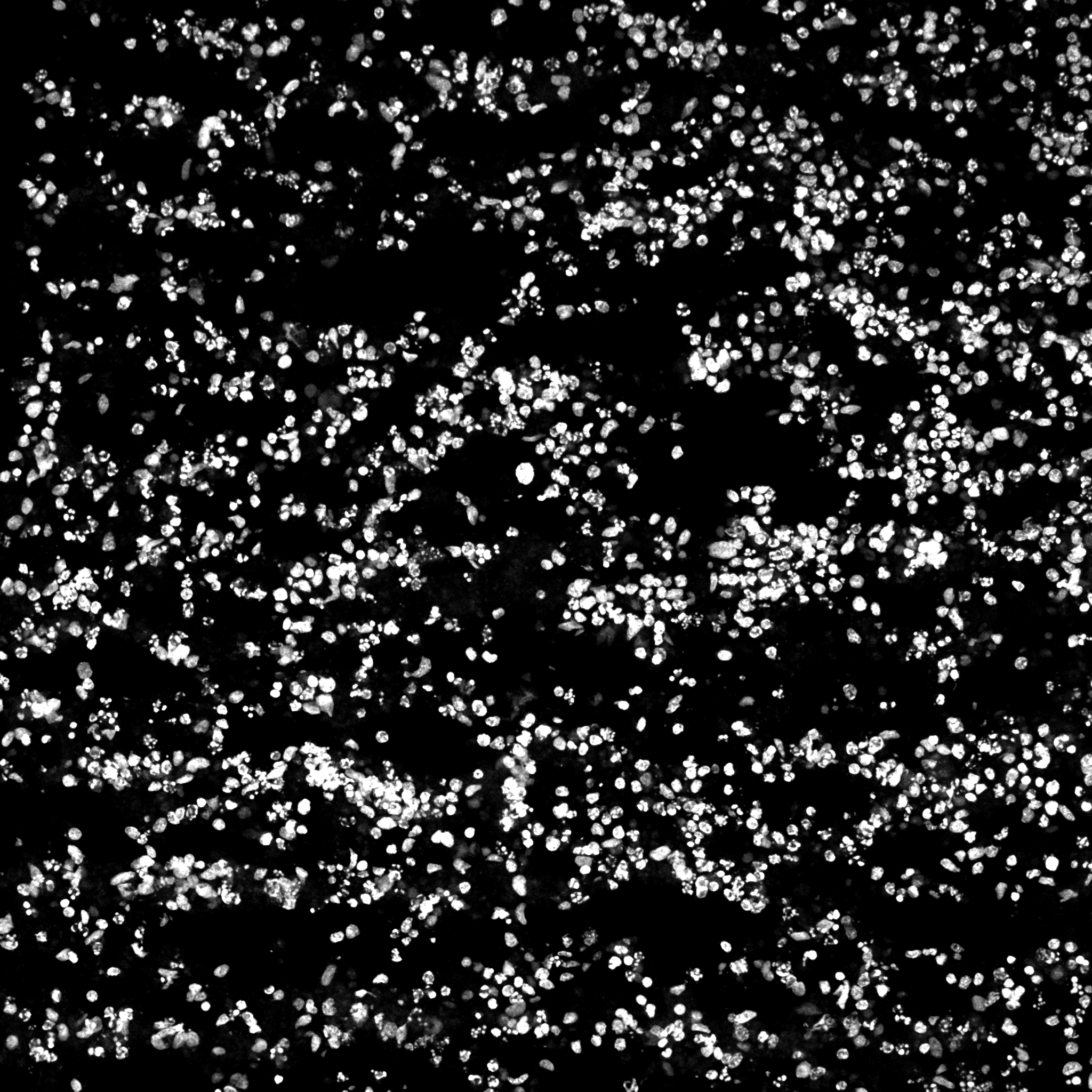

Supplement: Supplementary file 8 — Source Data for Figure 3 [file EMMM-15-e18199-s011.zip › Figure_3/3C/C'_PDO_T#9_D28_OLIG2,_Nestin_DAPI.tif]

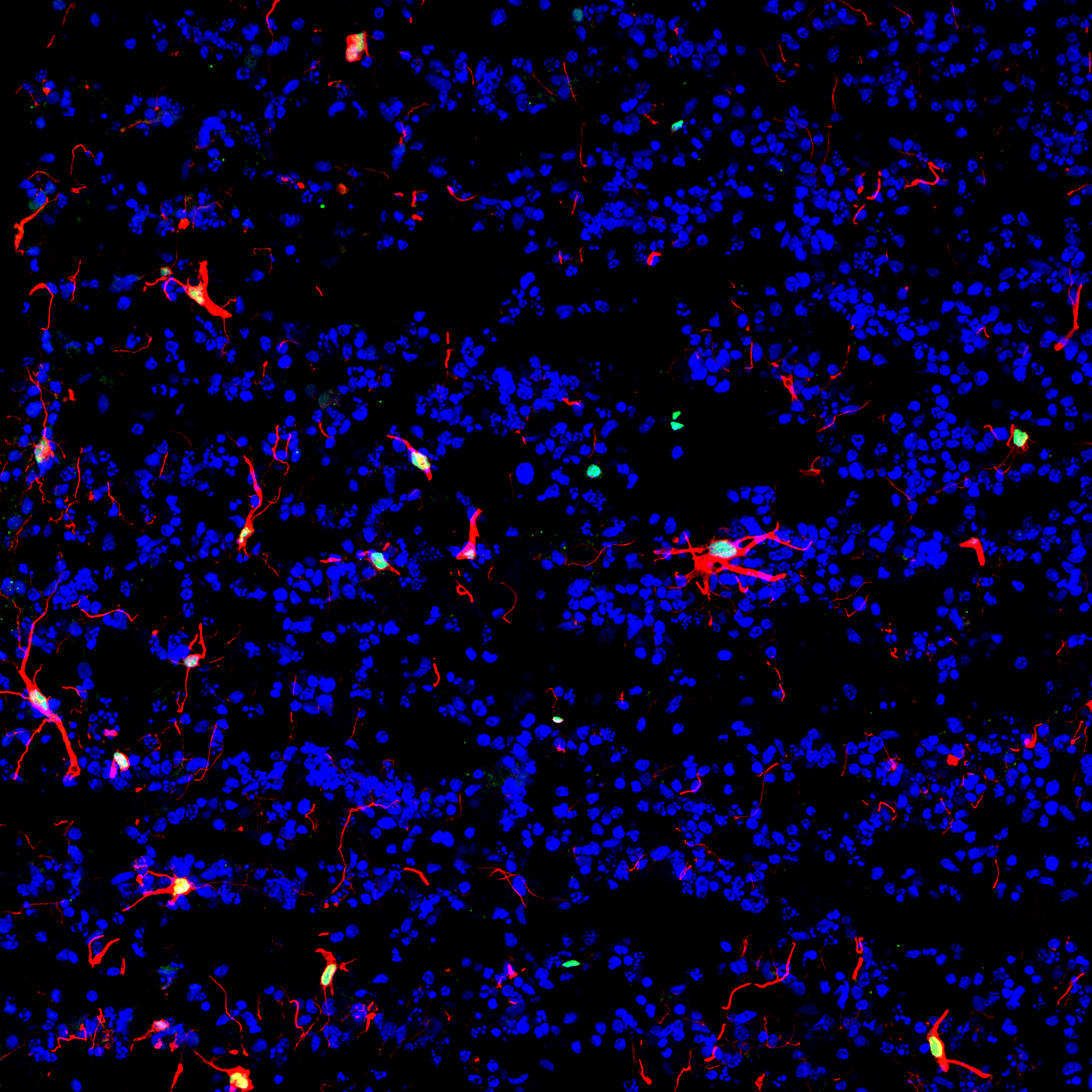

Supplement: Supplementary file 8 — Source Data for Figure 3 [file EMMM-15-e18199-s011.zip › Figure_3/3C/C'_PDO_T#9_D28_OLIG2,_Nestin_merge.tif]

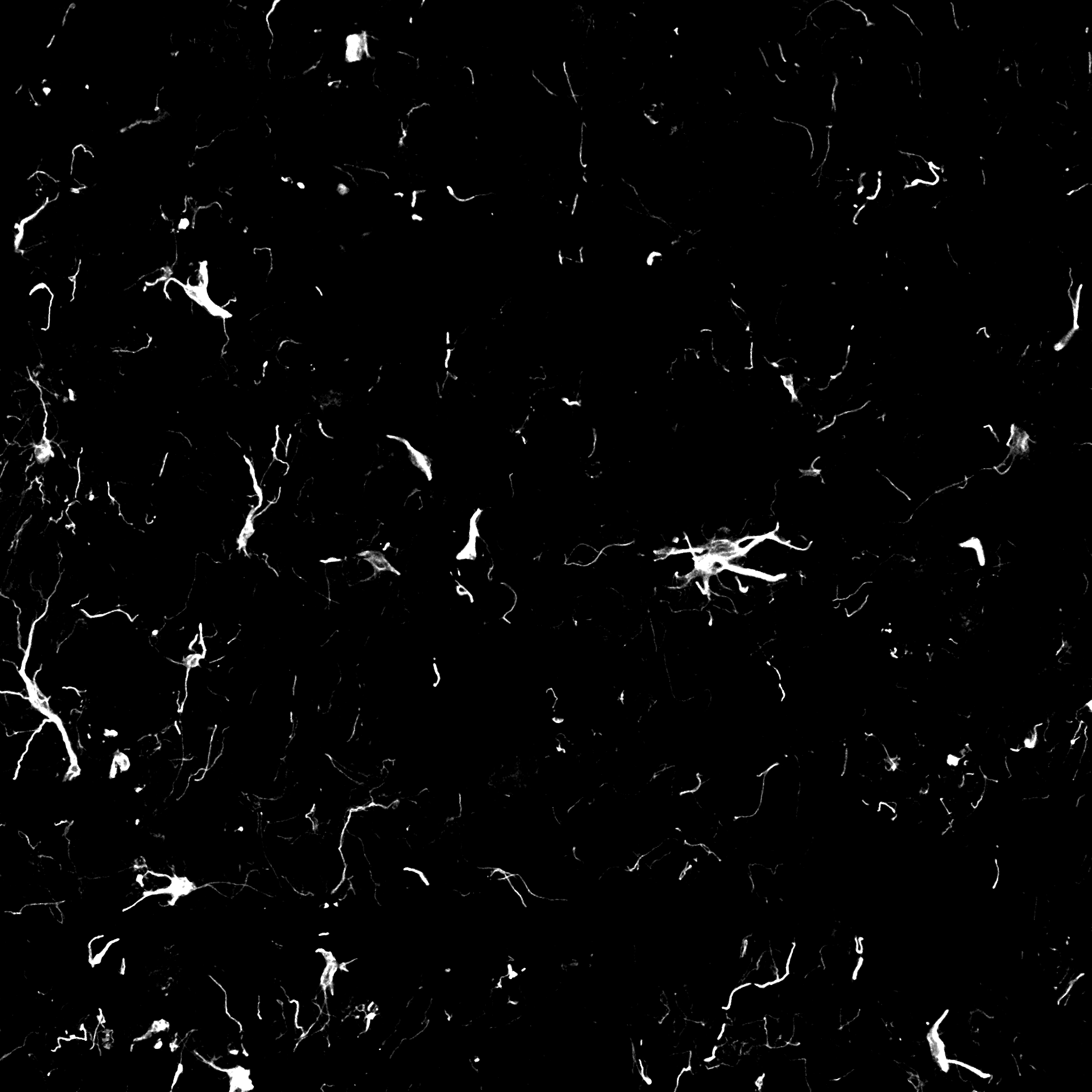

Supplement: Supplementary file 8 — Source Data for Figure 3 [file EMMM-15-e18199-s011.zip › Figure_3/3C/C'_PDO_T#9_D28_OLIG2,_Nestin_Nestin.tif]

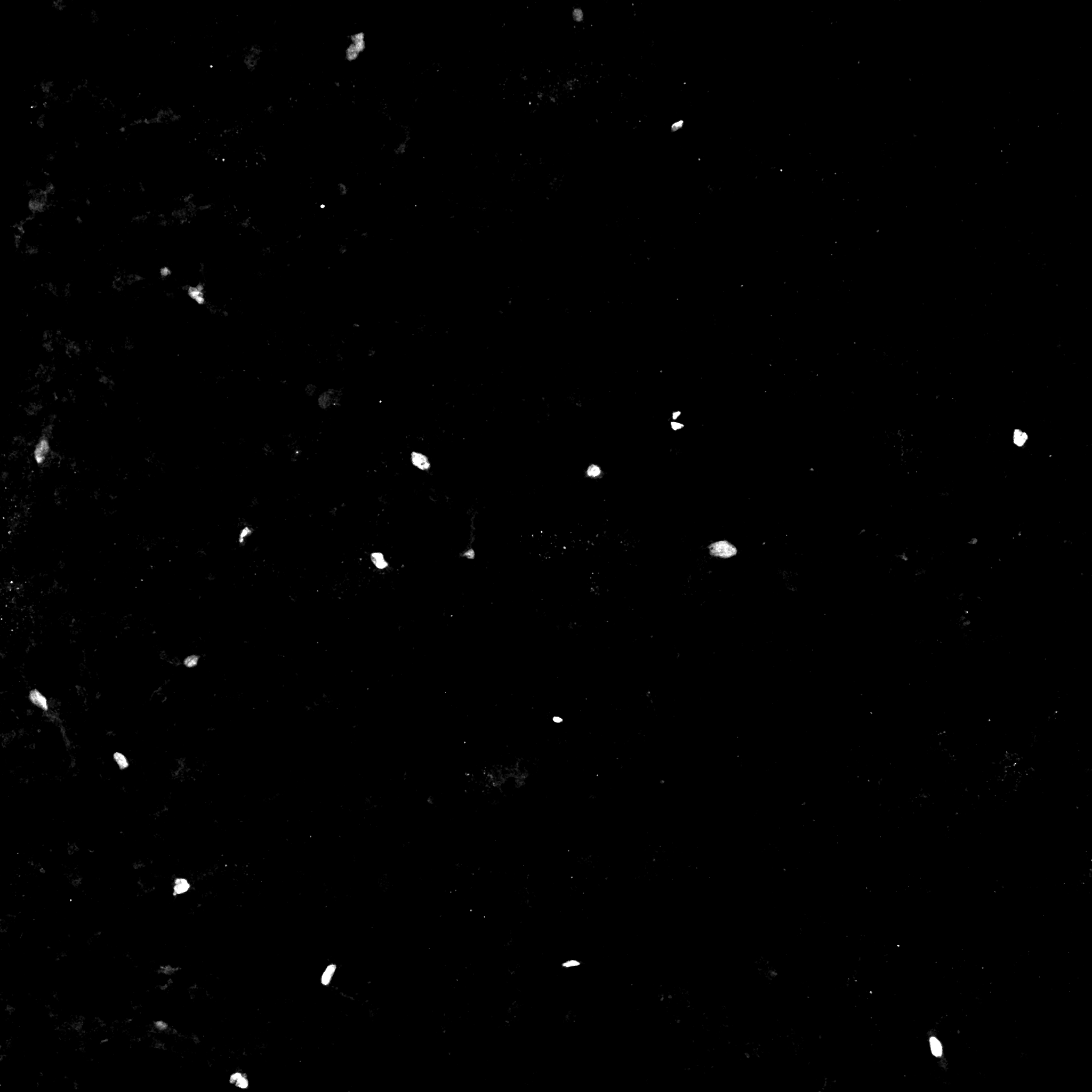

Supplement: Supplementary file 8 — Source Data for Figure 3 [file EMMM-15-e18199-s011.zip › Figure_3/3C/C'_PDO_T#9_D28_OLIG2,_Nestin_OLIG2.tif]

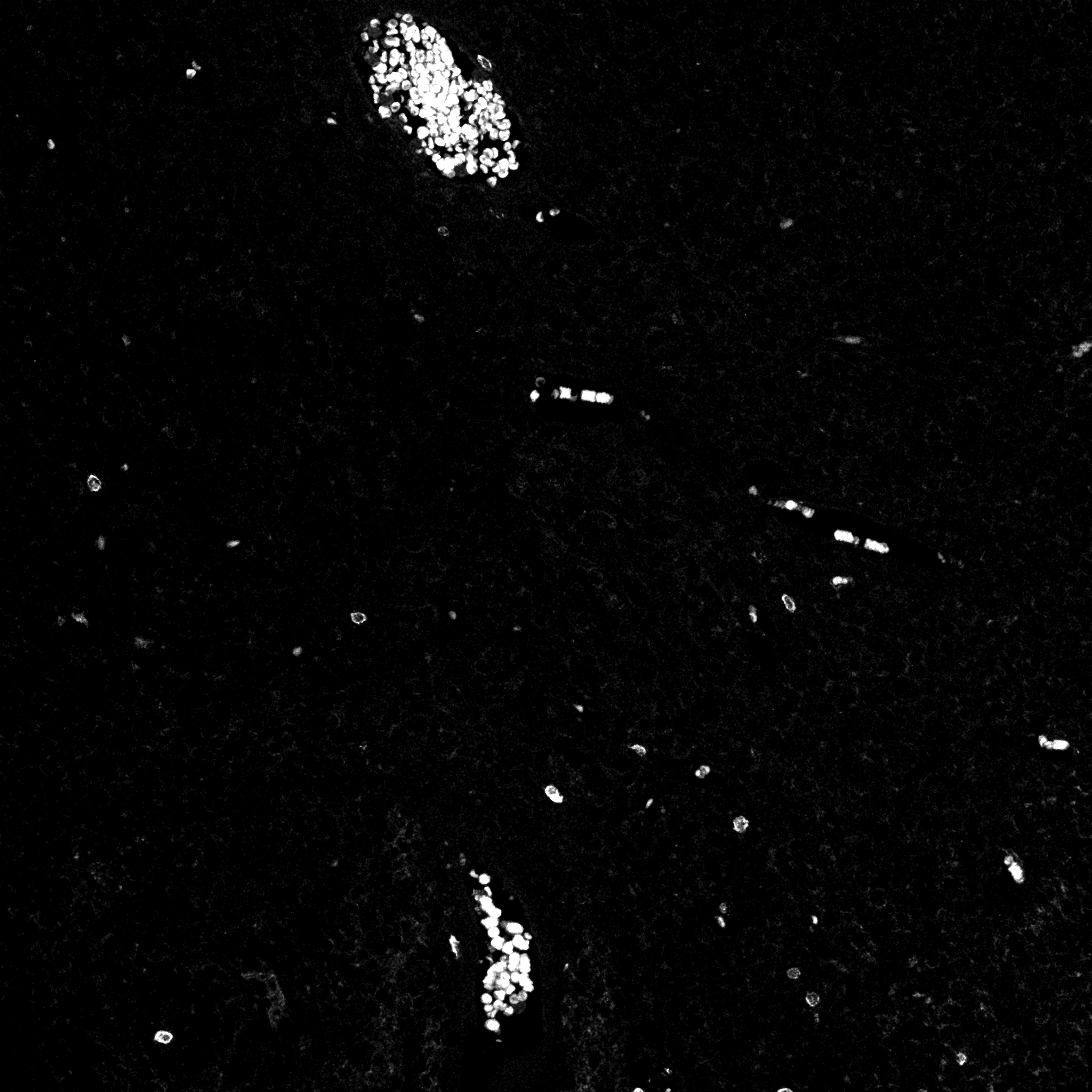

Supplement: Supplementary file 8 — Source Data for Figure 3 [file EMMM-15-e18199-s011.zip › Figure_3/3C/C'_Primary_T#9_CD34,_CD3_CD3.tif]

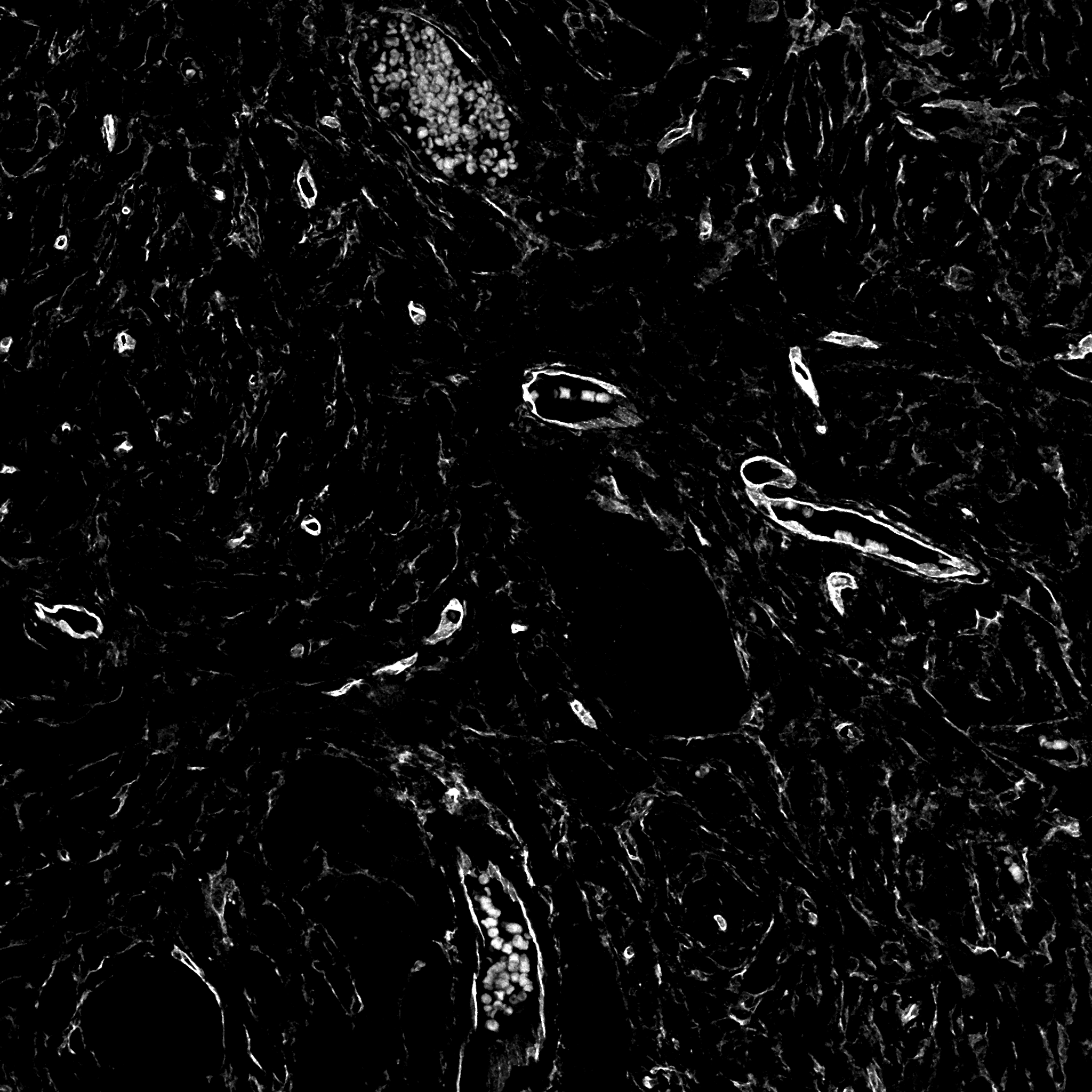

Supplement: Supplementary file 8 — Source Data for Figure 3 [file EMMM-15-e18199-s011.zip › Figure_3/3C/C'_Primary_T#9_CD34,_CD3_CD34.tif]

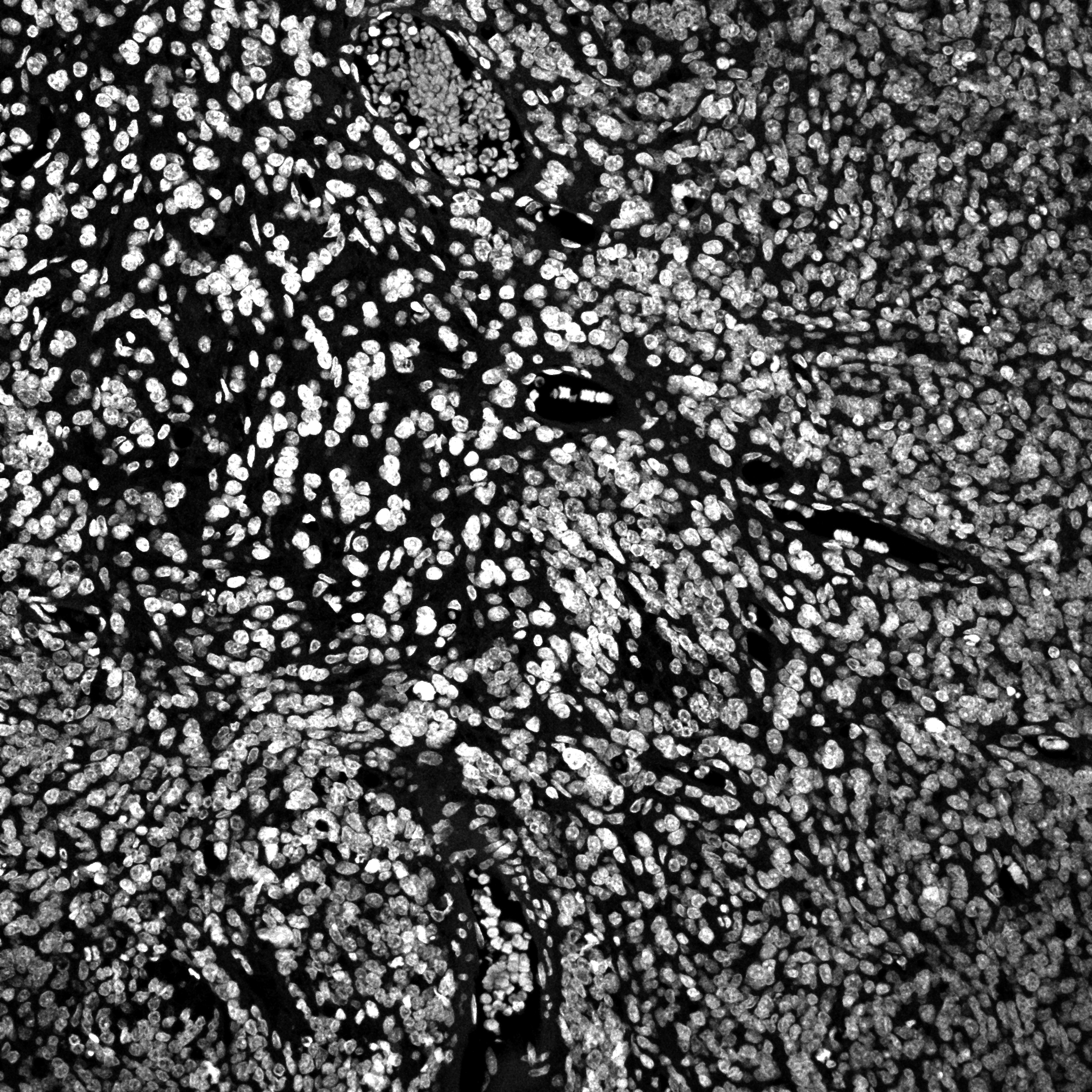

Supplement: Supplementary file 8 — Source Data for Figure 3 [file EMMM-15-e18199-s011.zip › Figure_3/3C/C'_Primary_T#9_CD34,_CD3_DAPI.tif]

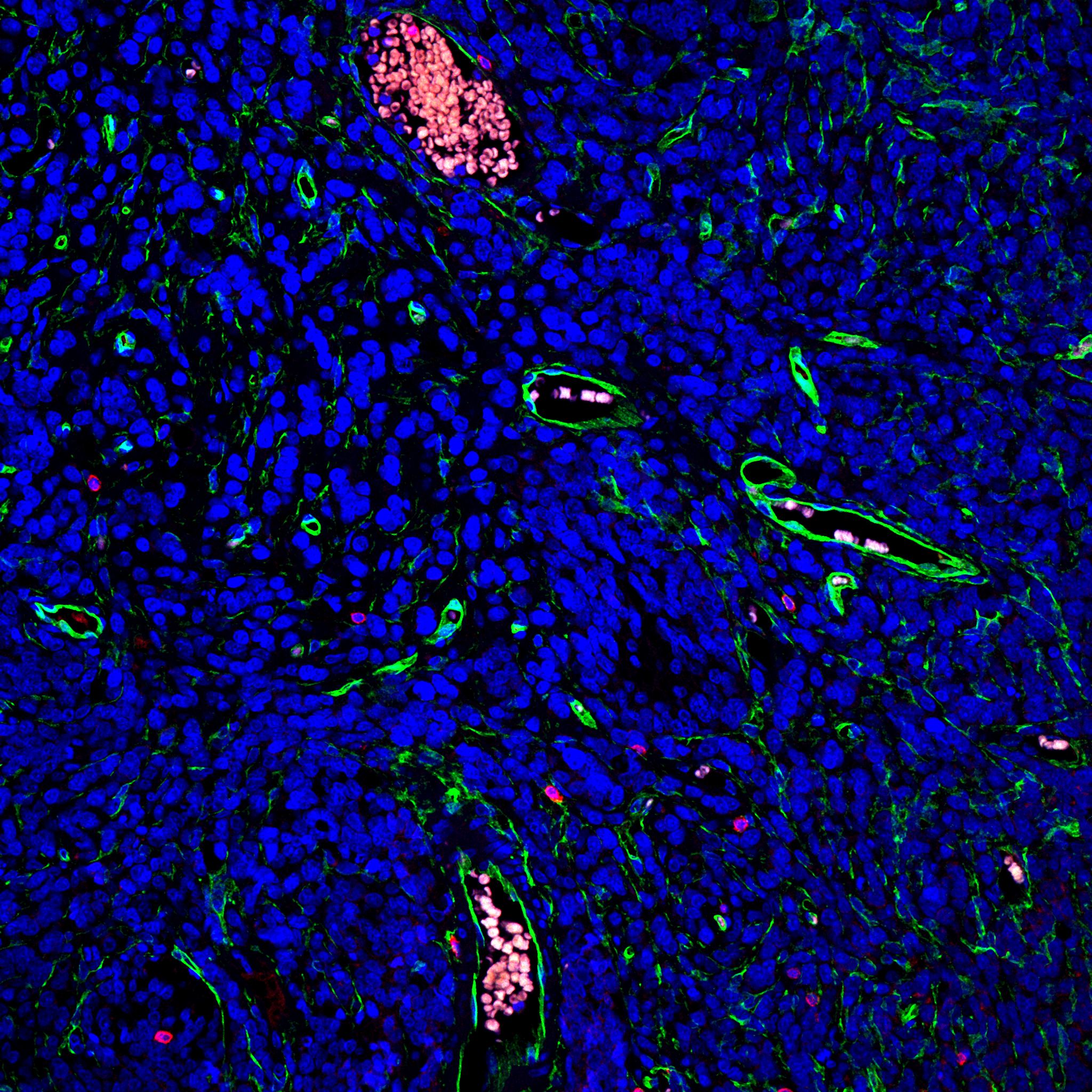

Supplement: Supplementary file 8 — Source Data for Figure 3 [file EMMM-15-e18199-s011.zip › Figure_3/3C/C'_Primary_T#9_CD34,_CD3_merge.tif]

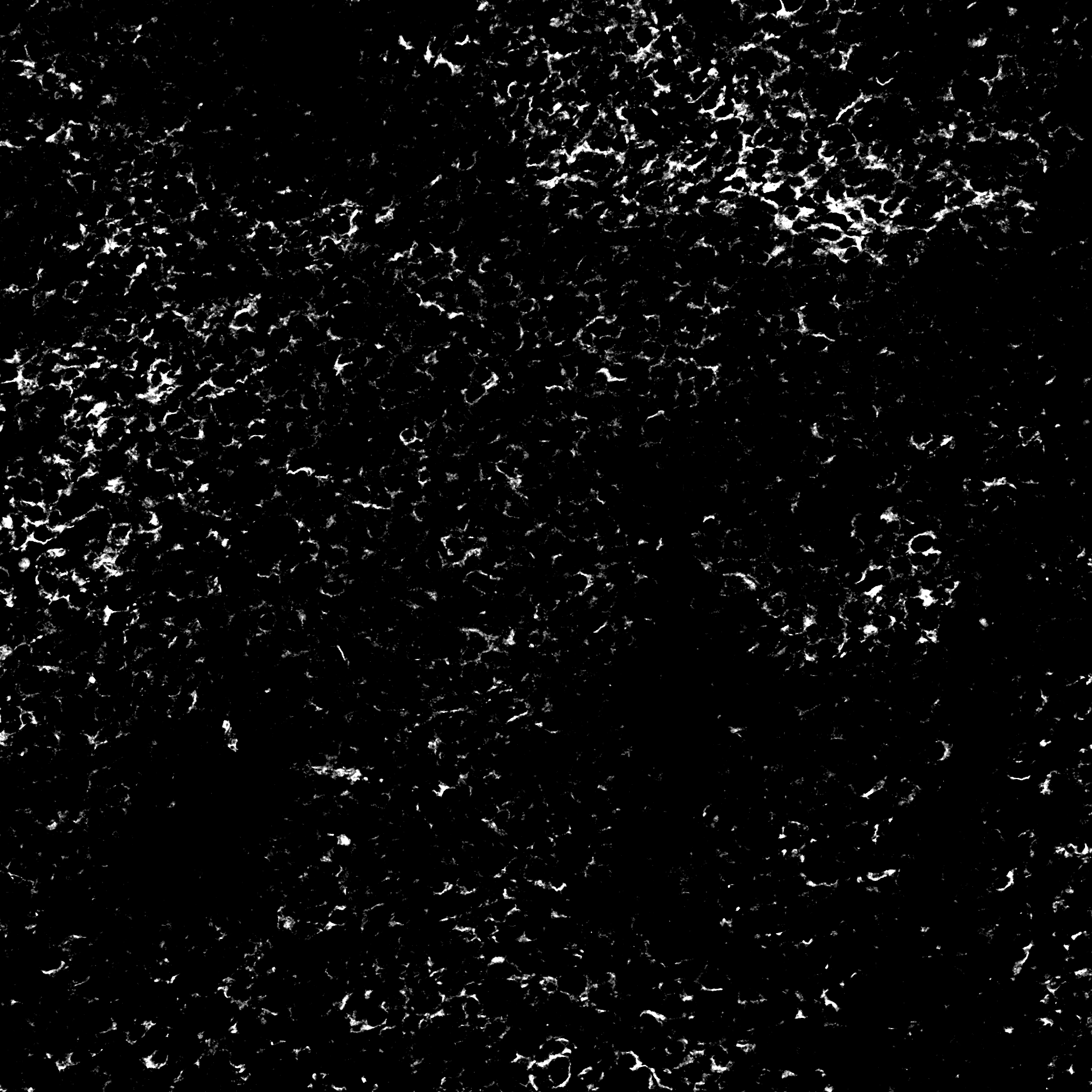

Supplement: Supplementary file 8 — Source Data for Figure 3 [file EMMM-15-e18199-s011.zip › Figure_3/3C/C'_Primary_T#9_GFAP,_B3tubulin_B3tubulin.tif]

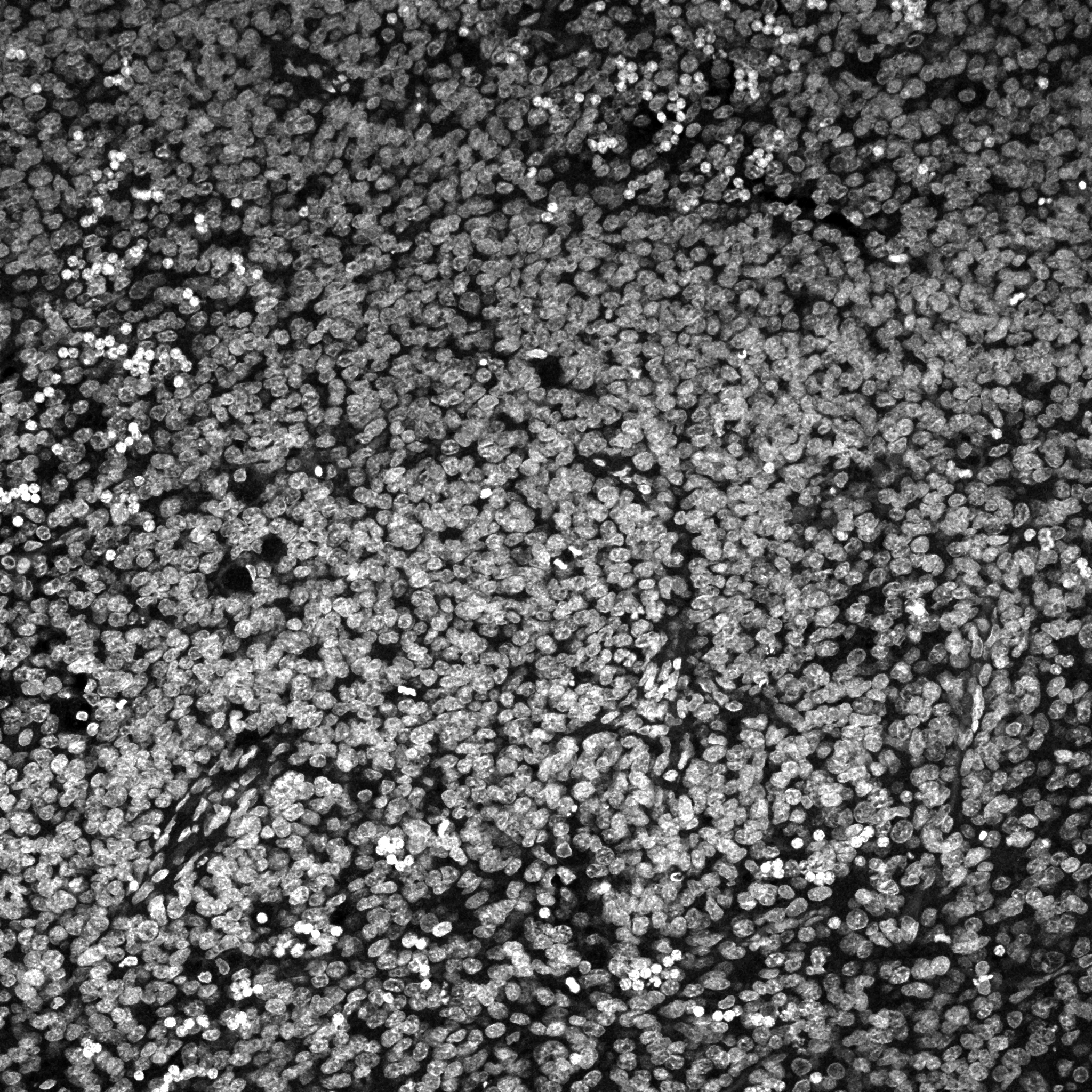

Supplement: Supplementary file 8 — Source Data for Figure 3 [file EMMM-15-e18199-s011.zip › Figure_3/3C/C'_Primary_T#9_GFAP,_B3tubulin_DAPI.tif]

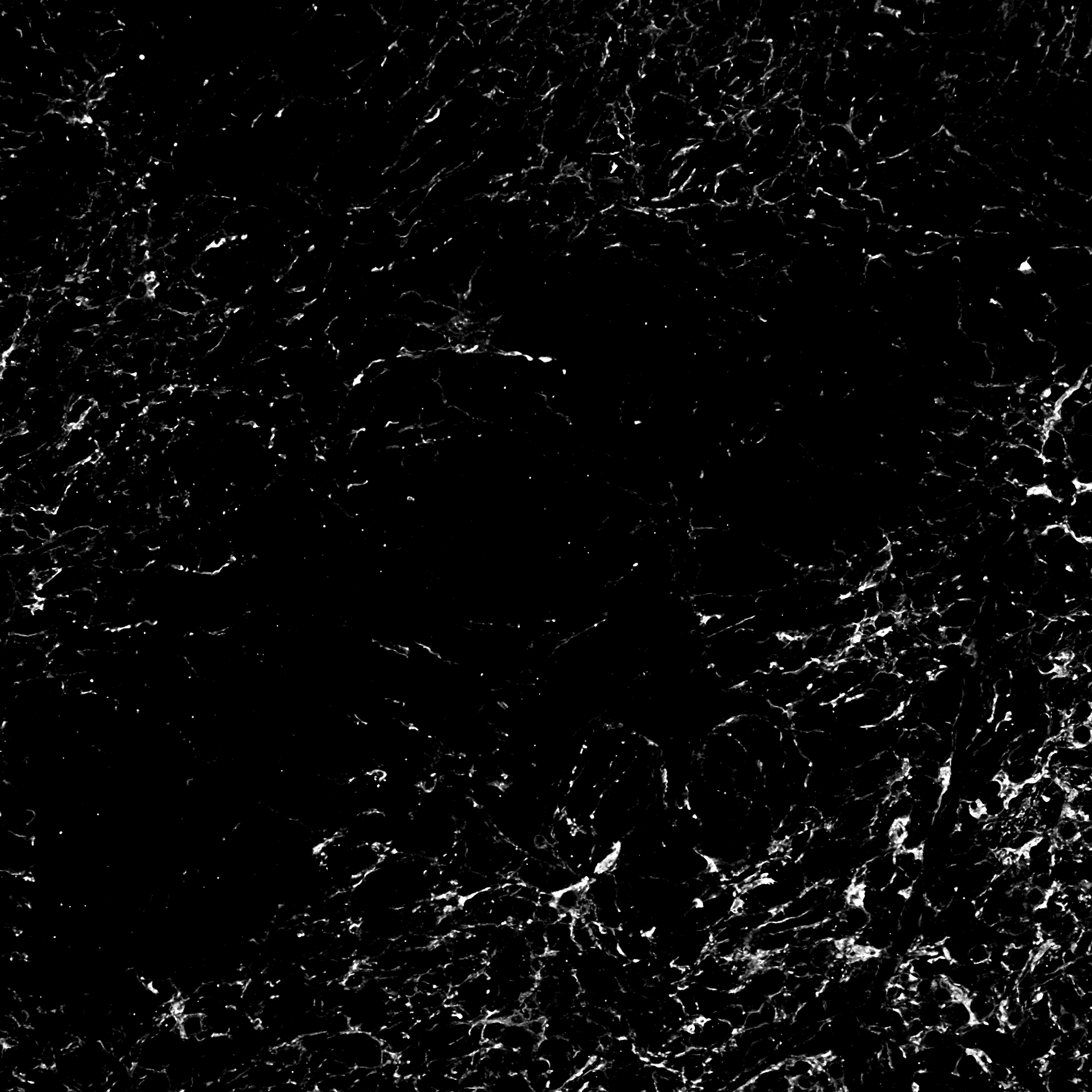

Supplement: Supplementary file 8 — Source Data for Figure 3 [file EMMM-15-e18199-s011.zip › Figure_3/3C/C'_Primary_T#9_GFAP,_B3tubulin_GFAP.tif]

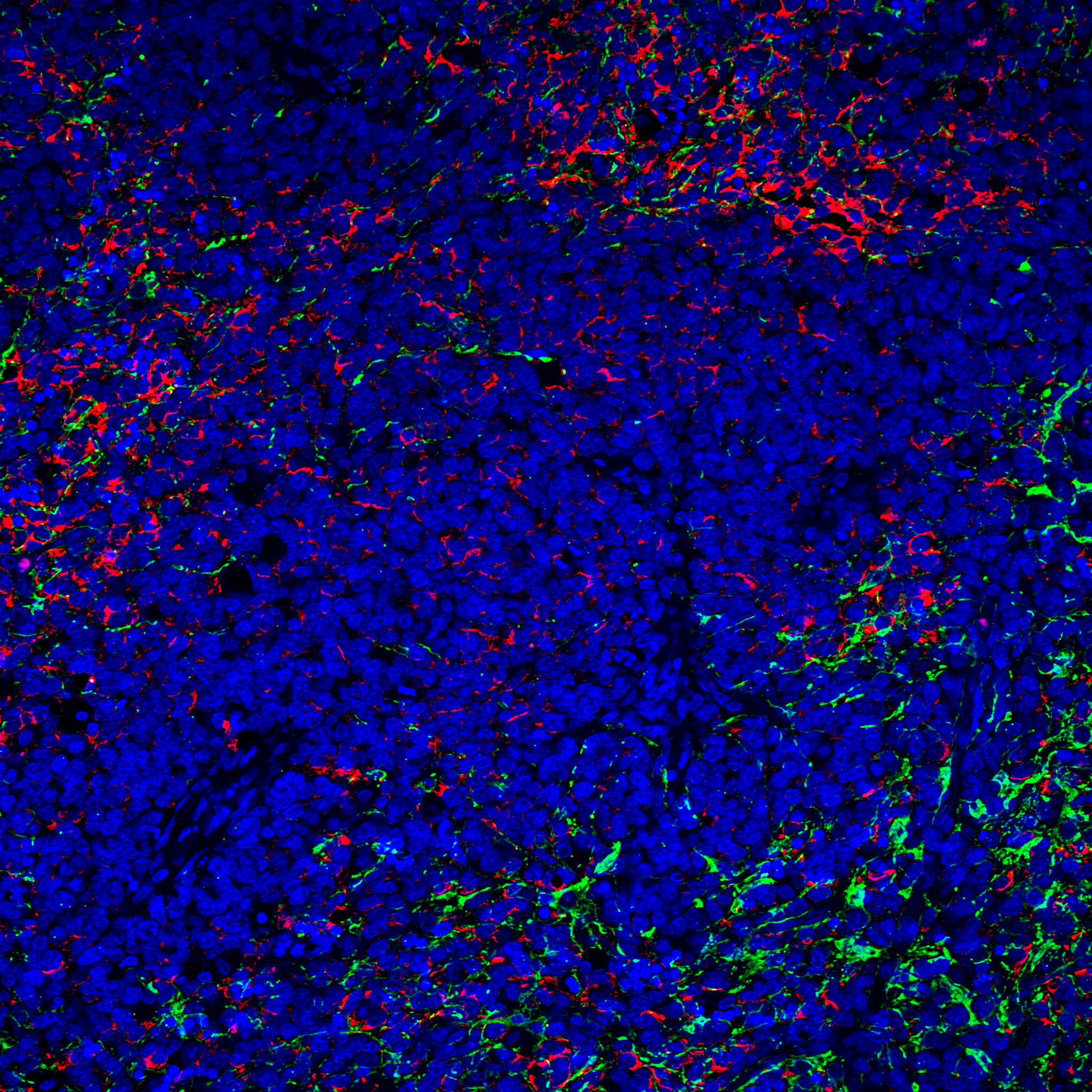

Supplement: Supplementary file 8 — Source Data for Figure 3 [file EMMM-15-e18199-s011.zip › Figure_3/3C/C'_Primary_T#9_GFAP,_B3tubulin_merge.tif]

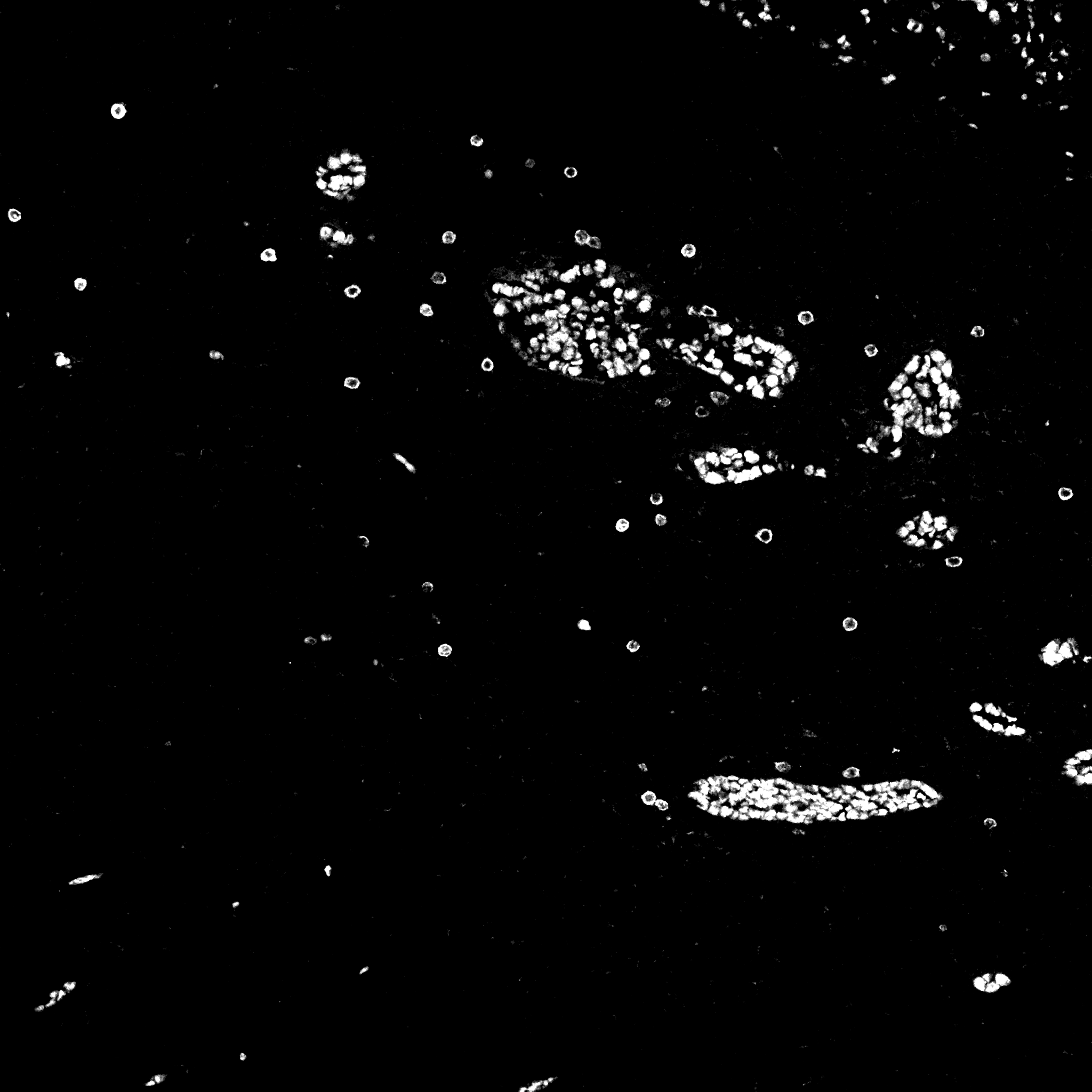

Supplement: Supplementary file 8 — Source Data for Figure 3 [file EMMM-15-e18199-s011.zip › Figure_3/3C/C'_Primary_T#9_IBA1,_CD3_CD3.tif]

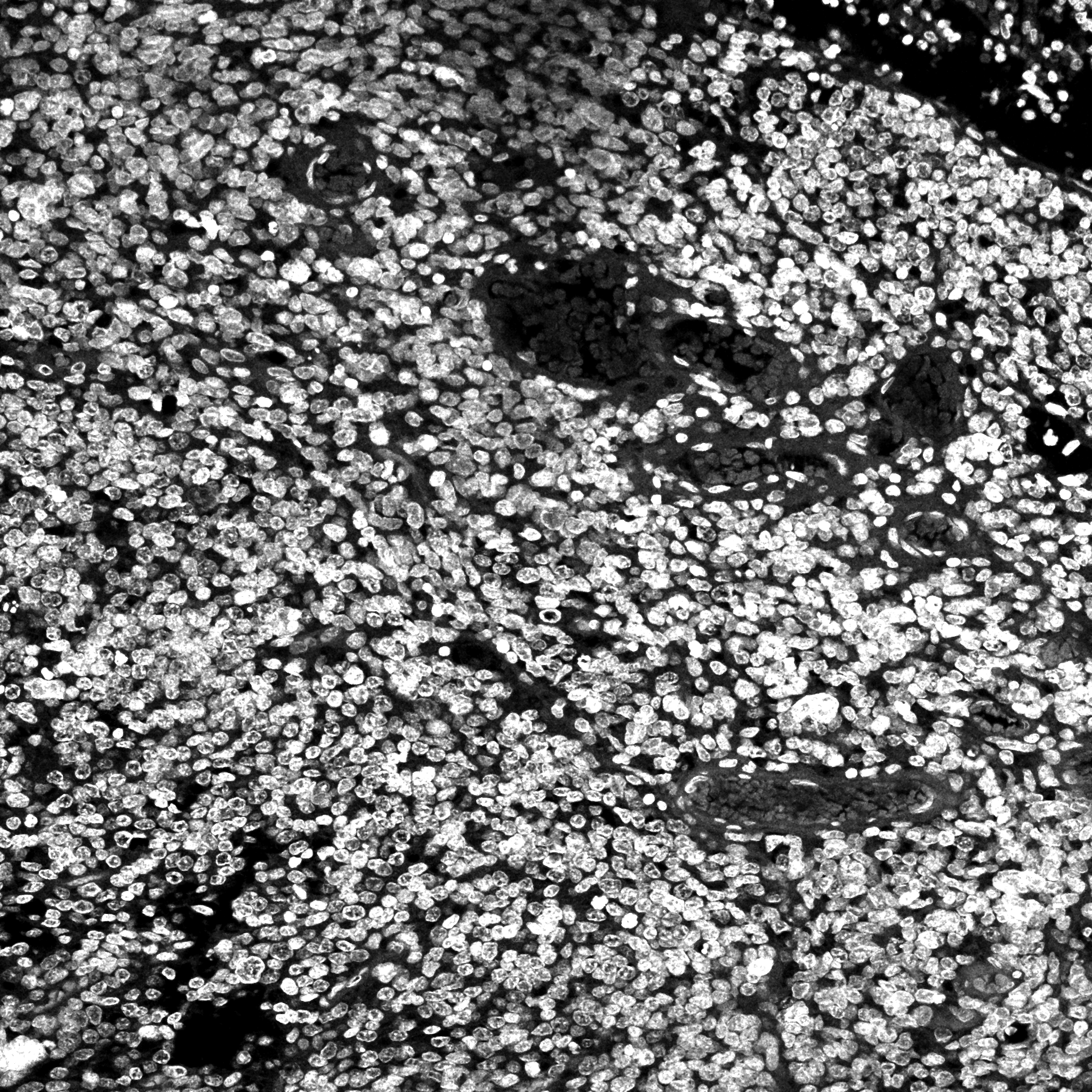

Supplement: Supplementary file 8 — Source Data for Figure 3 [file EMMM-15-e18199-s011.zip › Figure_3/3C/C'_Primary_T#9_IBA1,_CD3_DAPI.tif]

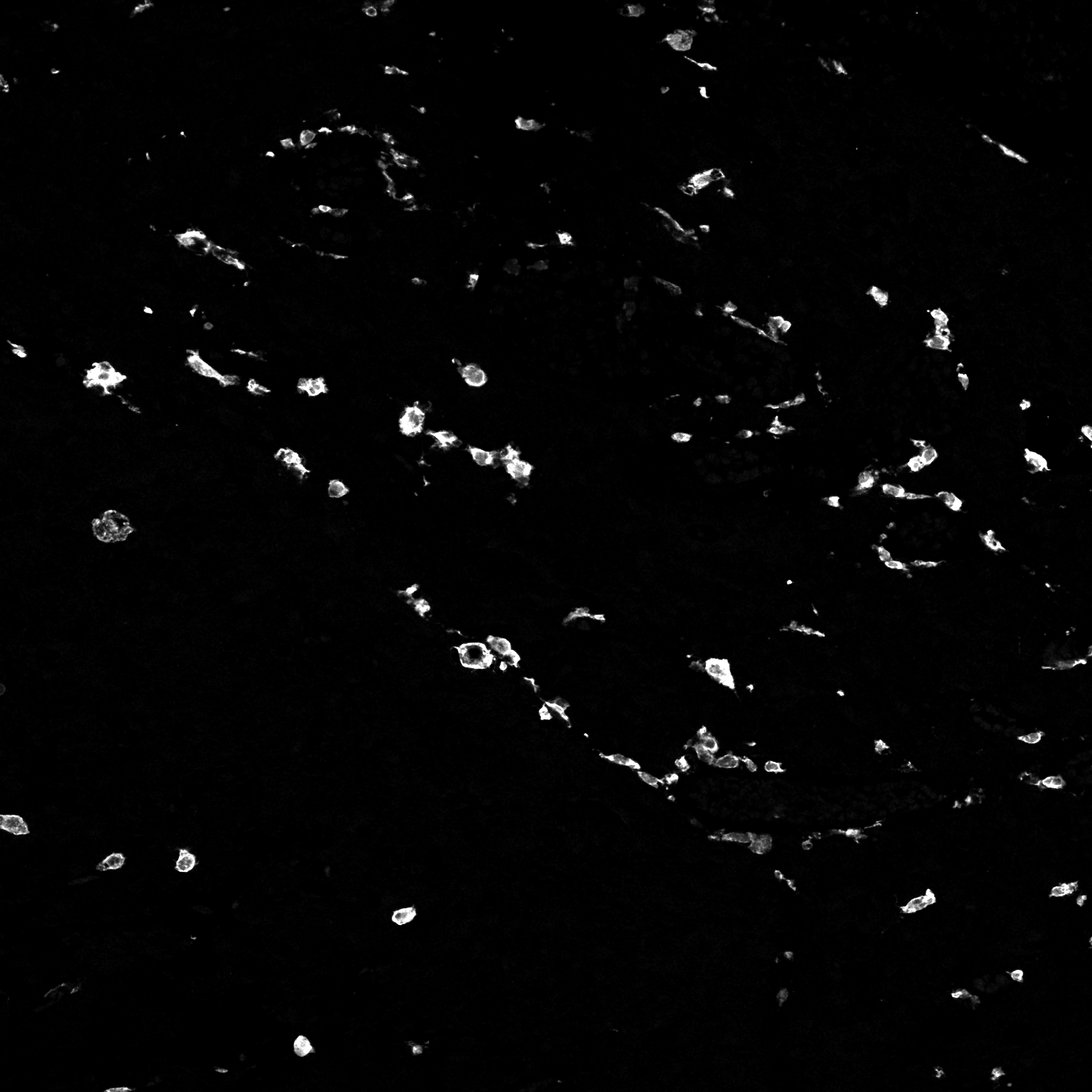

Supplement: Supplementary file 8 — Source Data for Figure 3 [file EMMM-15-e18199-s011.zip › Figure_3/3C/C'_Primary_T#9_IBA1,_CD3_IBA1.tif]

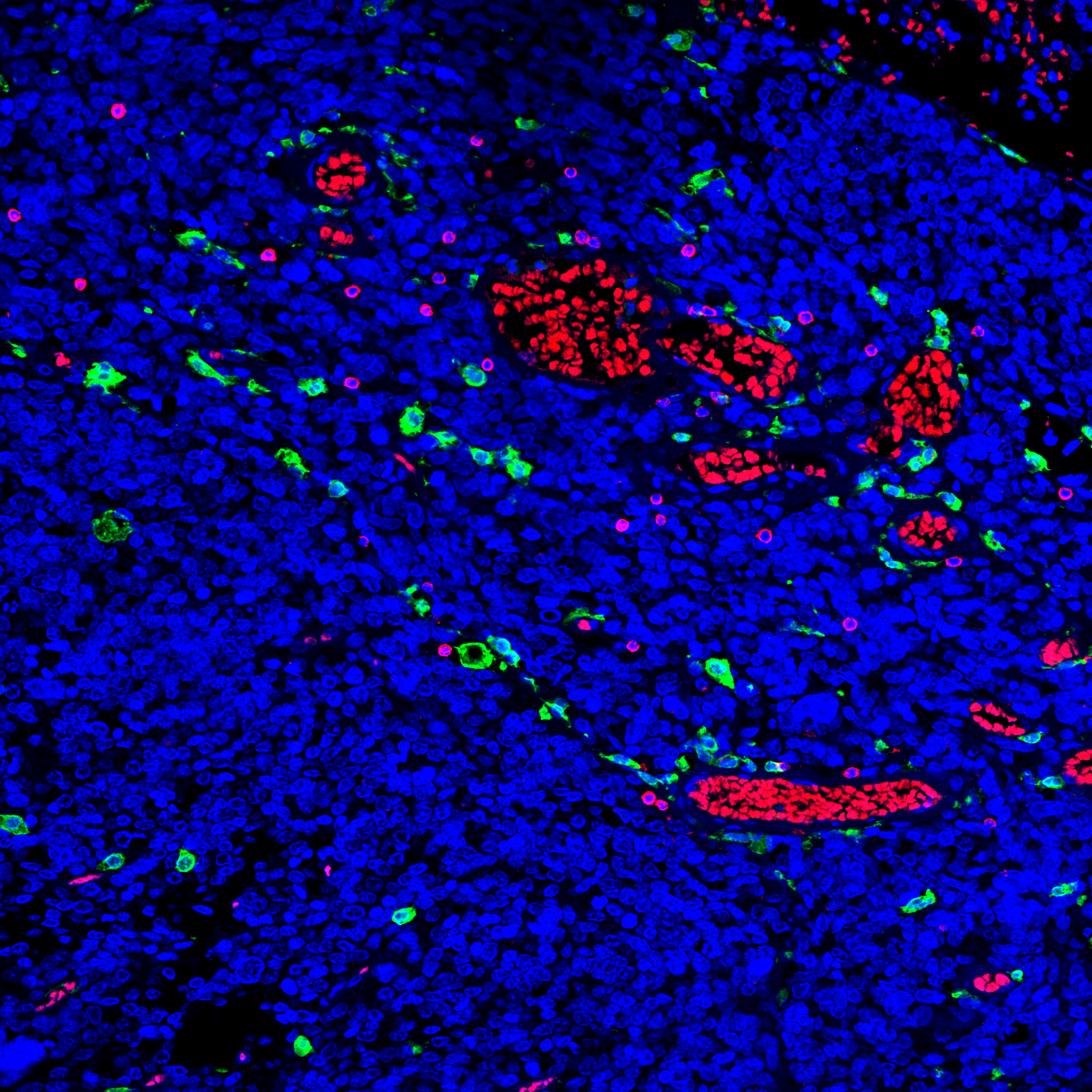

Supplement: Supplementary file 8 — Source Data for Figure 3 [file EMMM-15-e18199-s011.zip › Figure_3/3C/C'_Primary_T#9_IBA1,_CD3_merge.tif]

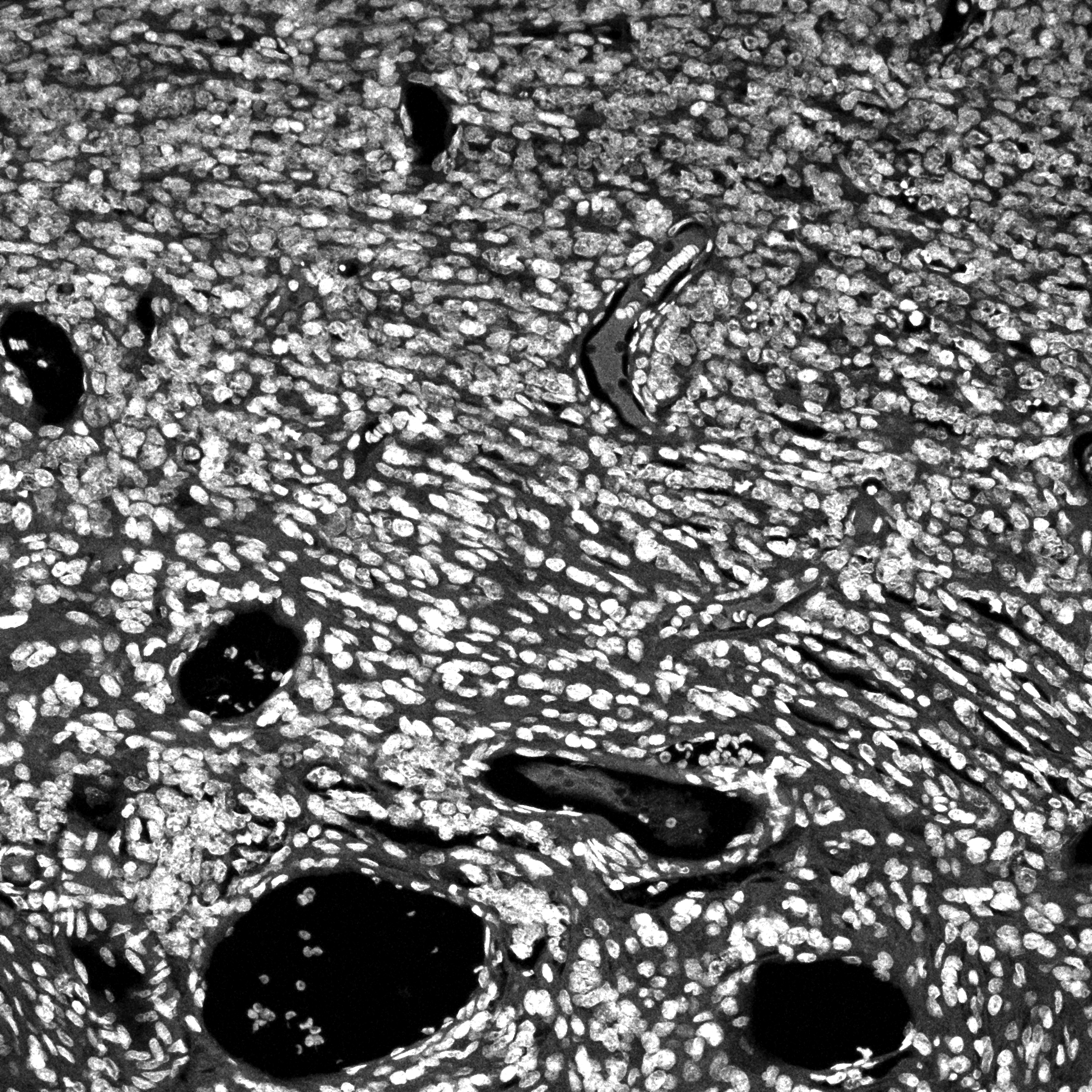

Supplement: Supplementary file 8 — Source Data for Figure 3 [file EMMM-15-e18199-s011.zip › Figure_3/3C/C'_Primary_T#9_Ki67,_SOX2_DAPI.tif]

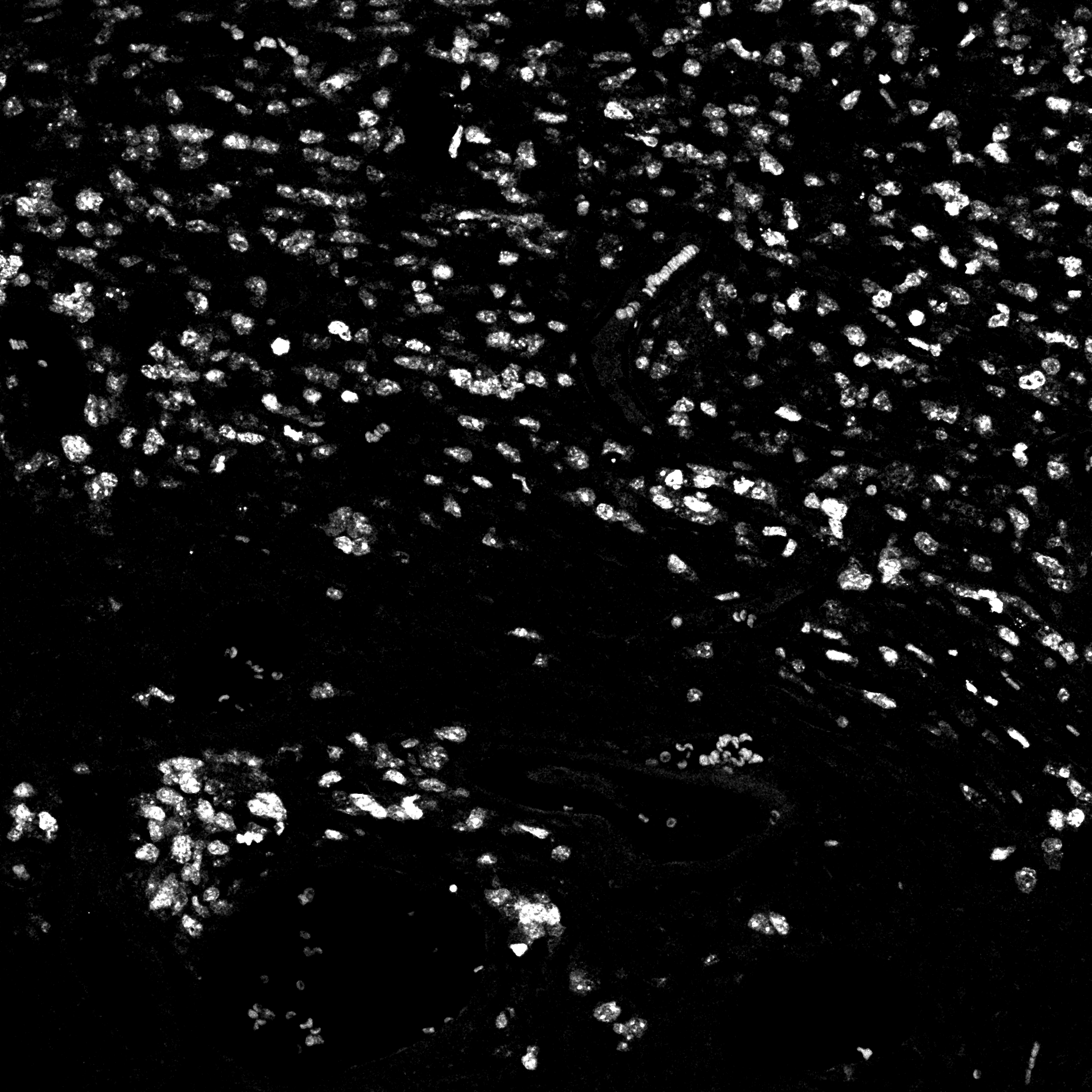

Supplement: Supplementary file 8 — Source Data for Figure 3 [file EMMM-15-e18199-s011.zip › Figure_3/3C/C'_Primary_T#9_Ki67,_SOX2_Ki67.tif]

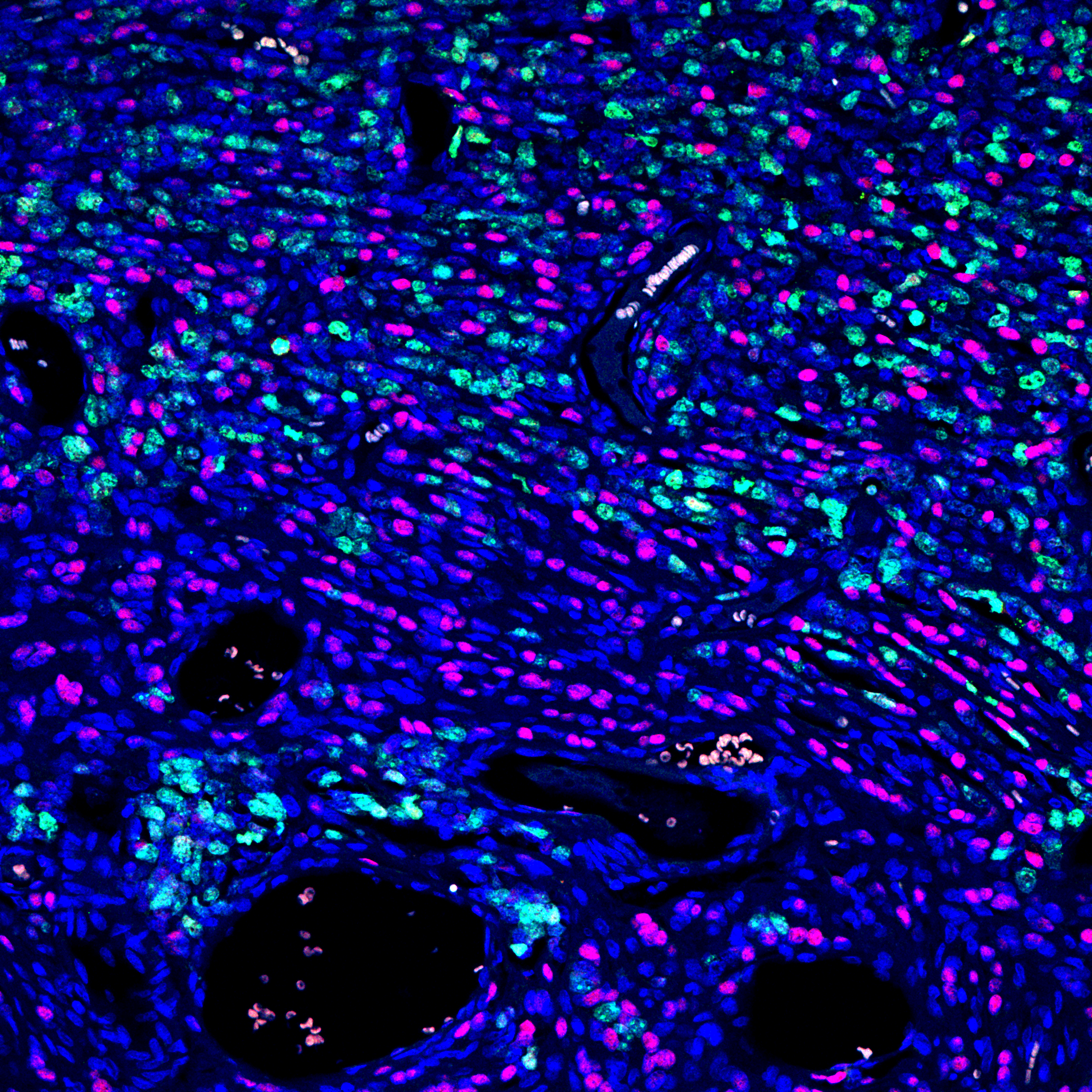

Supplement: Supplementary file 8 — Source Data for Figure 3 [file EMMM-15-e18199-s011.zip › Figure_3/3C/C'_Primary_T#9_Ki67,_SOX2_merge.tif]

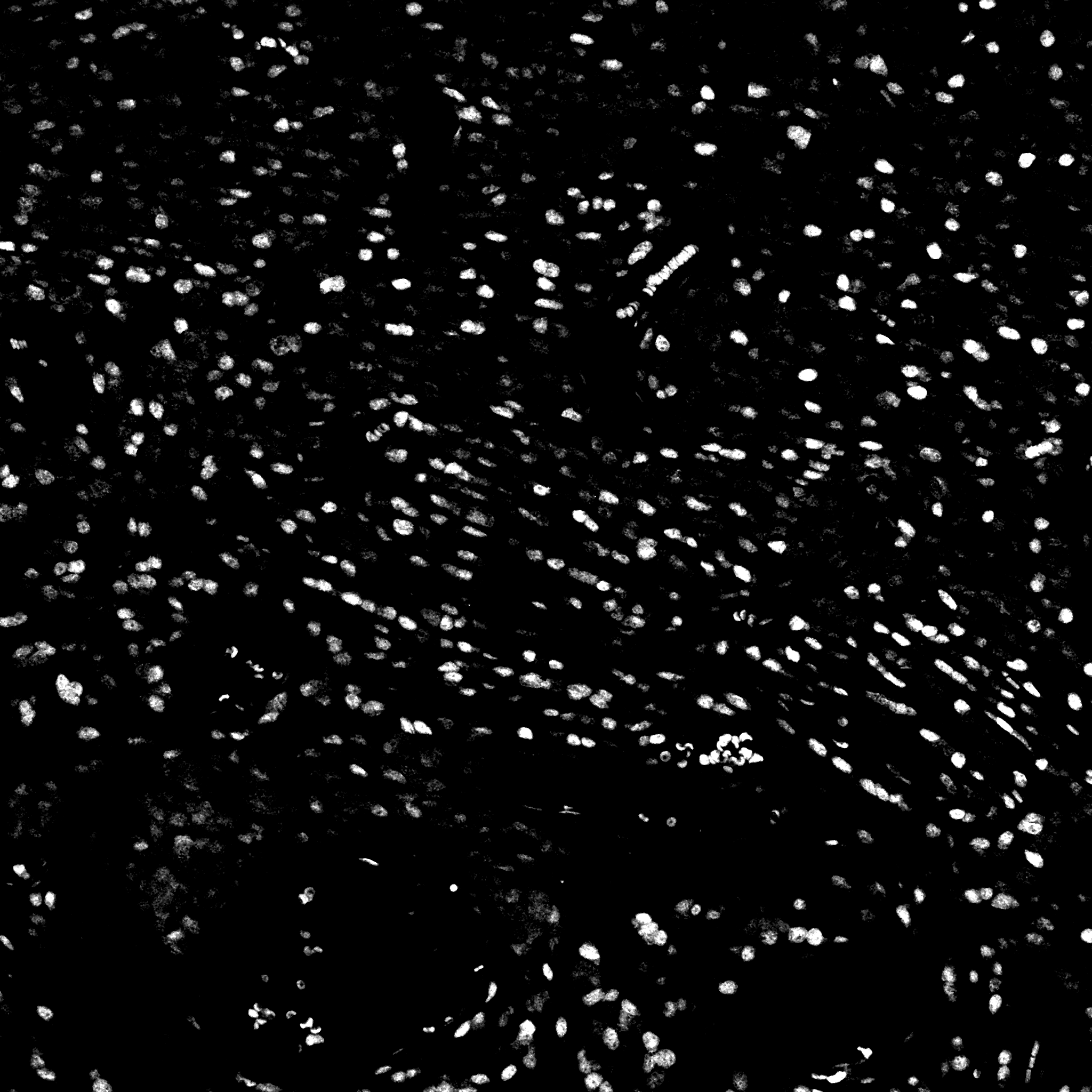

Supplement: Supplementary file 8 — Source Data for Figure 3 [file EMMM-15-e18199-s011.zip › Figure_3/3C/C'_Primary_T#9_Ki67,_SOX2_SOX2.tif]

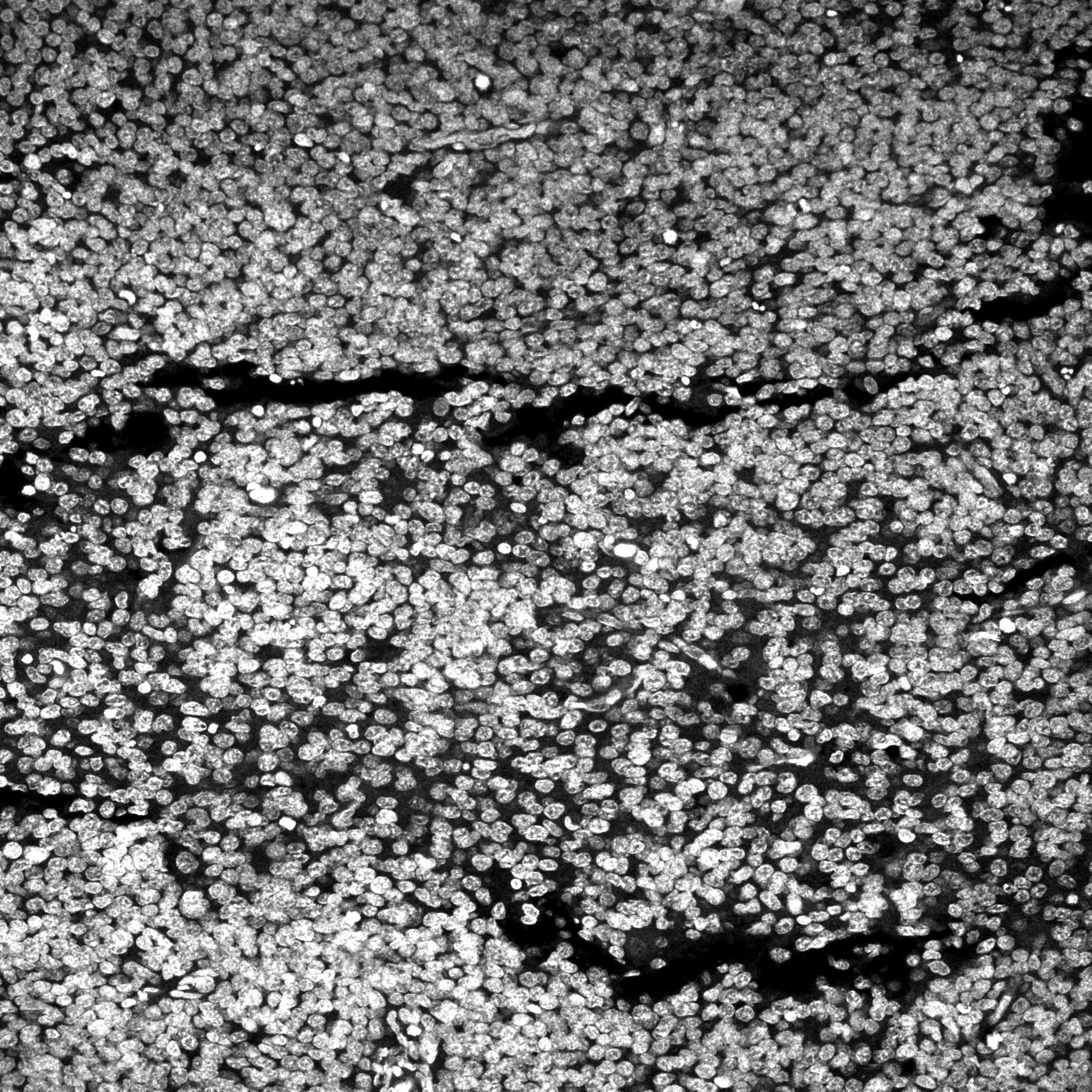

Supplement: Supplementary file 8 — Source Data for Figure 3 [file EMMM-15-e18199-s011.zip › Figure_3/3C/C'_Primary_T#9_OLIG2,_Nestin_DAPI.tif]

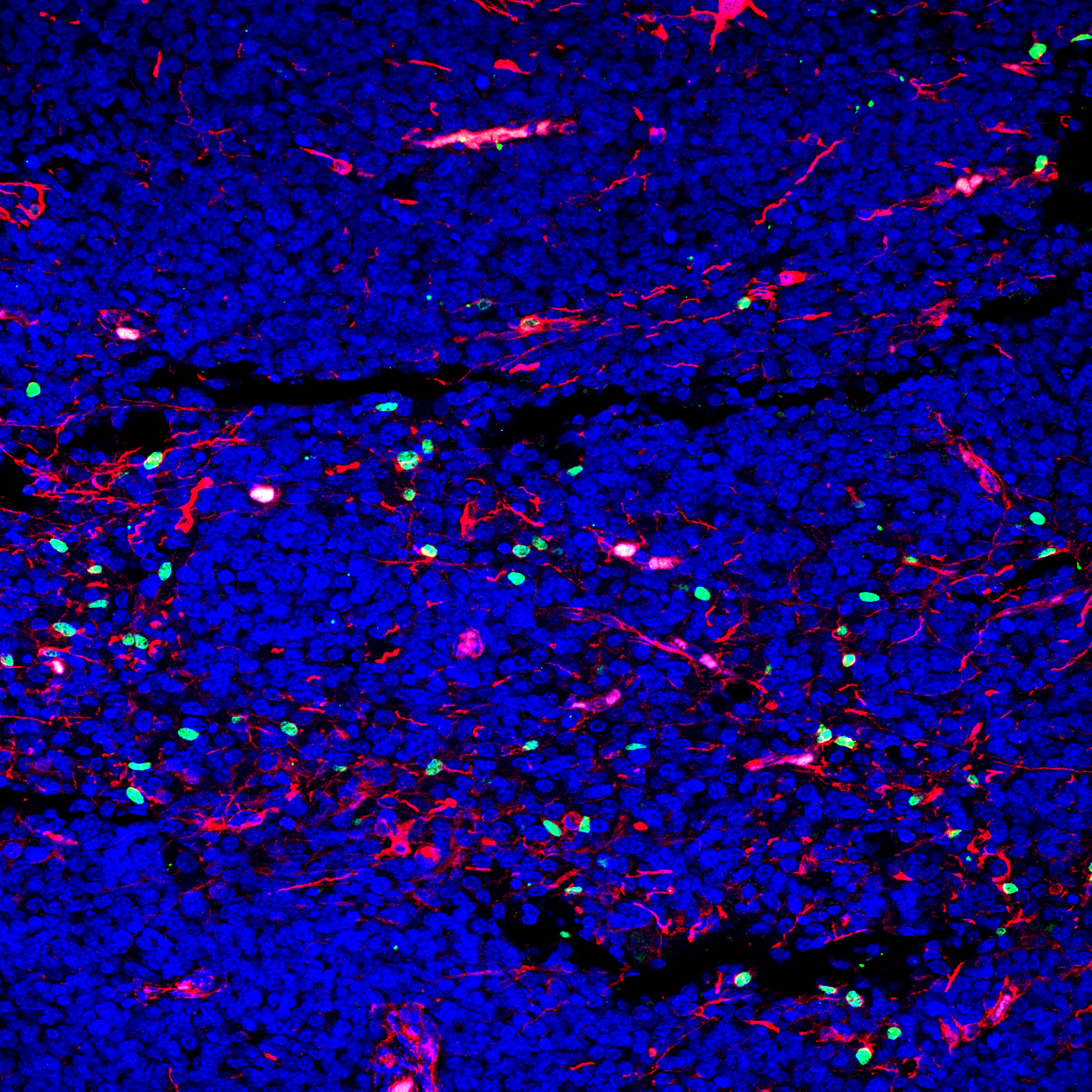

Supplement: Supplementary file 8 — Source Data for Figure 3 [file EMMM-15-e18199-s011.zip › Figure_3/3C/C'_Primary_T#9_OLIG2,_Nestin_merge.tif]

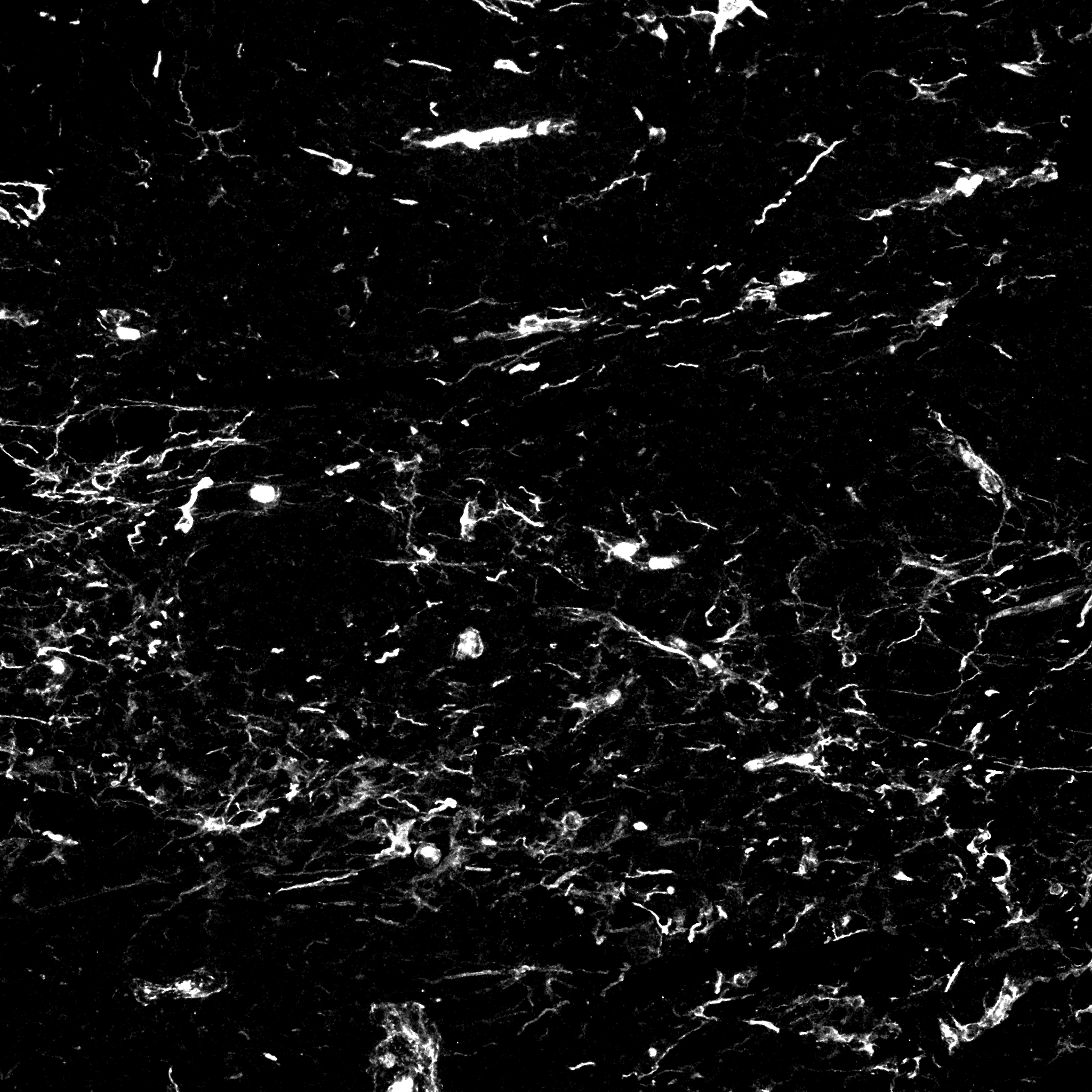

Supplement: Supplementary file 8 — Source Data for Figure 3 [file EMMM-15-e18199-s011.zip › Figure_3/3C/C'_Primary_T#9_OLIG2,_Nestin_Nestin.tif]
