# Supplementary figures and images for: Patient‐ and xenograft‐derived organoids recapitulate pediatric brain tumor features and patient treatments (part 1 of 4)
Source: EMBO Mol Med. 2023 Nov 30;15(12):e18199. doi: 10.15252/emmm.202318199 (PMC10701620; doi:10.15252/emmm.202318199)

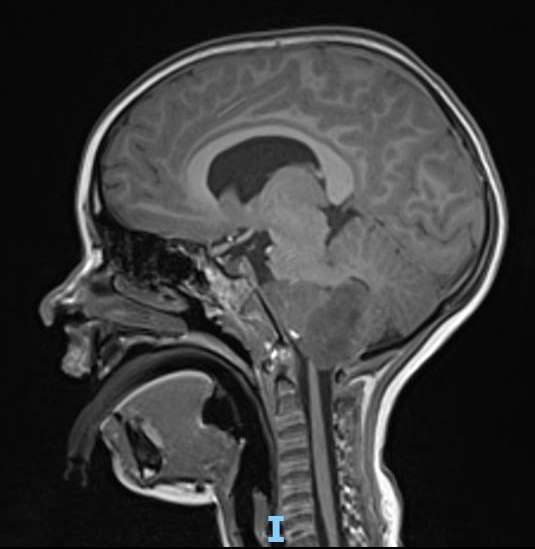

Supplement: Supplementary file 6 — Source Data for Figure 1 [file EMMM-15-e18199-s012.zip › Figure_1A,D,E/1A/Tumor_#1_MRI_sagittal_plane.png]

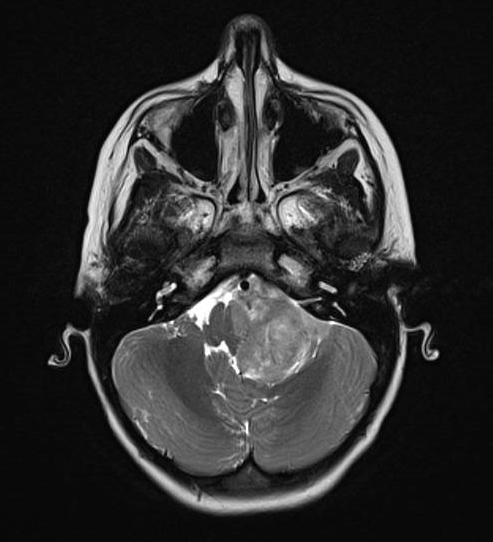

Supplement: Supplementary file 6 — Source Data for Figure 1 [file EMMM-15-e18199-s012.zip › Figure_1A,D,E/1A/Tumor_#1_MRI_transverse_plane.png]

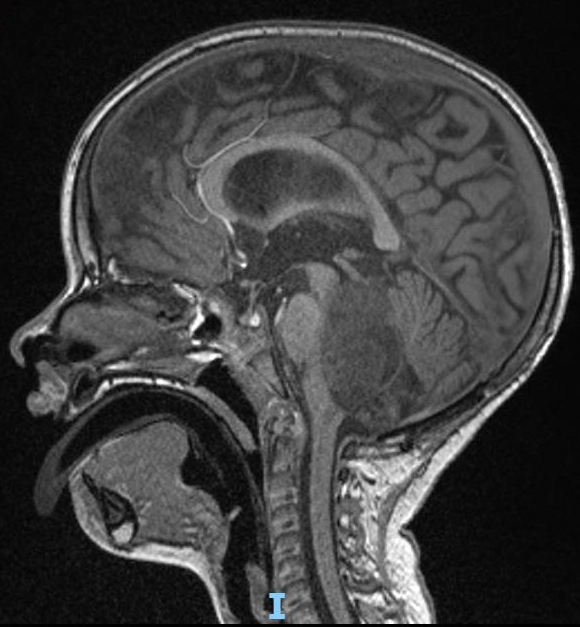

Supplement: Supplementary file 6 — Source Data for Figure 1 [file EMMM-15-e18199-s012.zip › Figure_1A,D,E/1A/Tumor_#21_MRI_sagittal_plane.png]

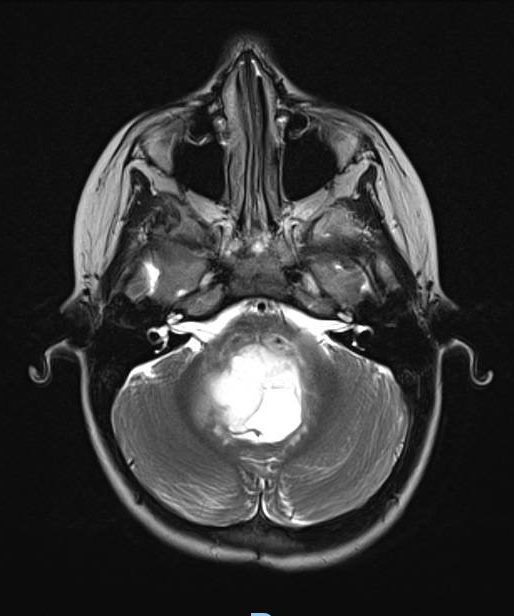

Supplement: Supplementary file 6 — Source Data for Figure 1 [file EMMM-15-e18199-s012.zip › Figure_1A,D,E/1A/Tumor_#21_MRI_transverse_plane.png]

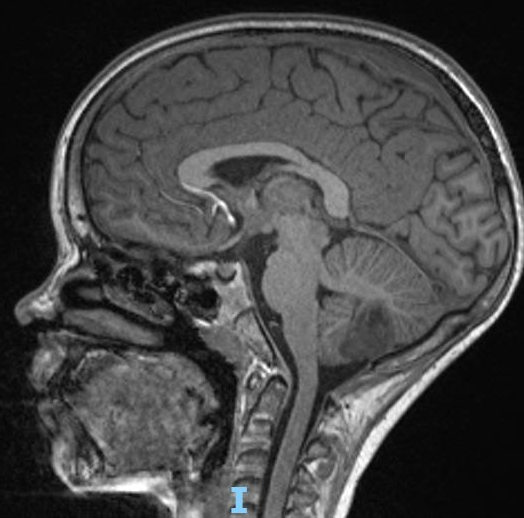

Supplement: Supplementary file 6 — Source Data for Figure 1 [file EMMM-15-e18199-s012.zip › Figure_1A,D,E/1A/Tumor_#9_MRI_sagittal_plane.png]

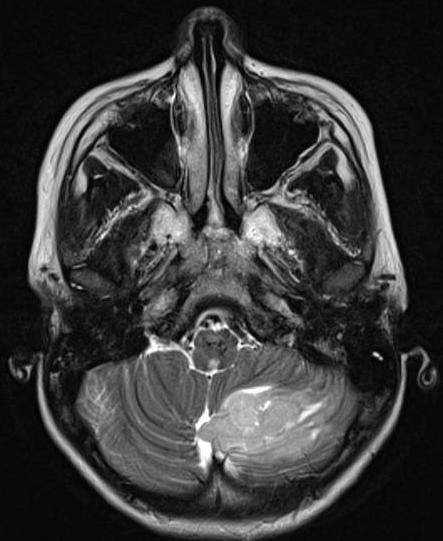

Supplement: Supplementary file 6 — Source Data for Figure 1 [file EMMM-15-e18199-s012.zip › Figure_1A,D,E/1A/Tumor_#9_MRI_transverse_plane.png]

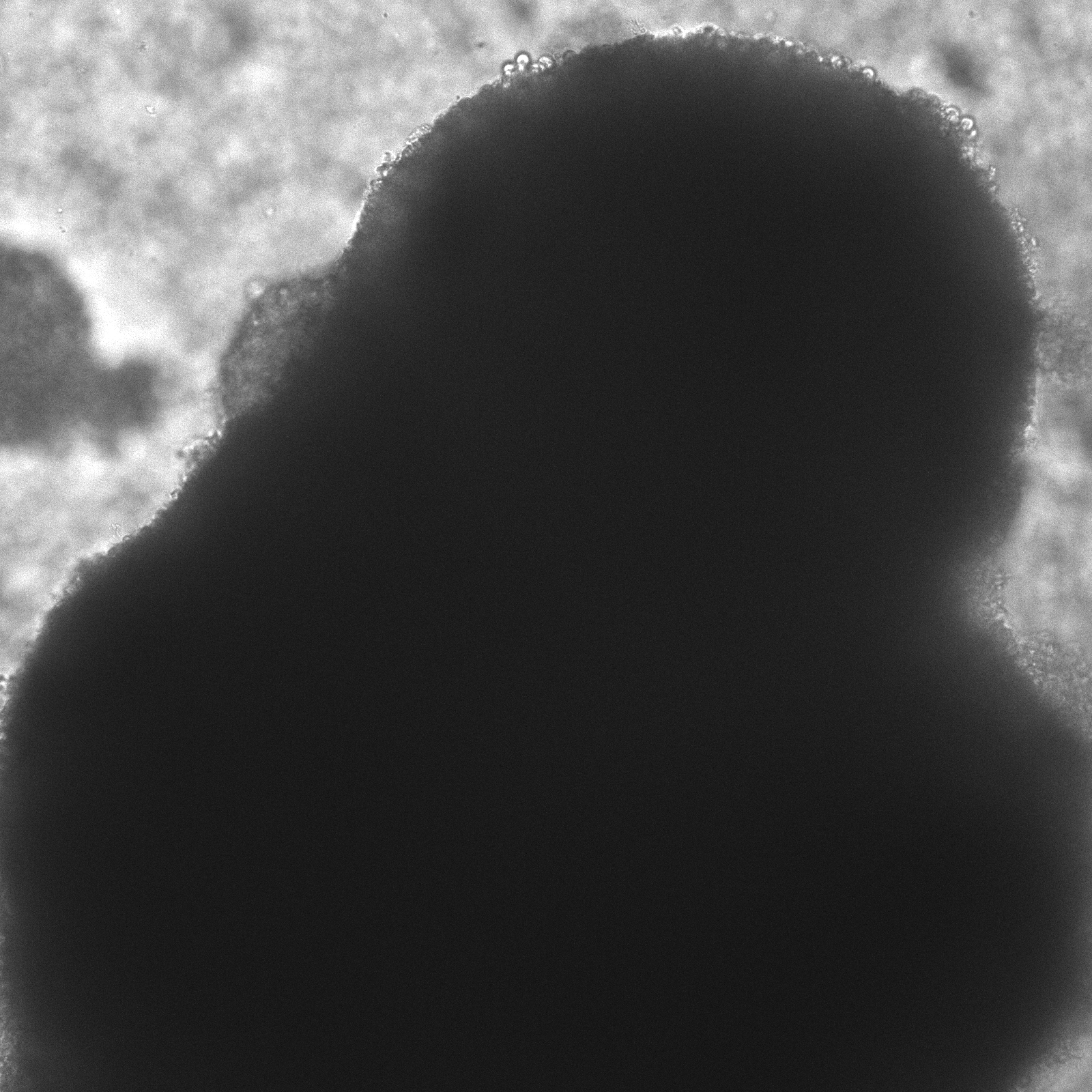

Supplement: Supplementary file 6 — Source Data for Figure 1 [file EMMM-15-e18199-s012.zip › Figure_1A,D,E/1D/Tumor_#1_D14.tif]

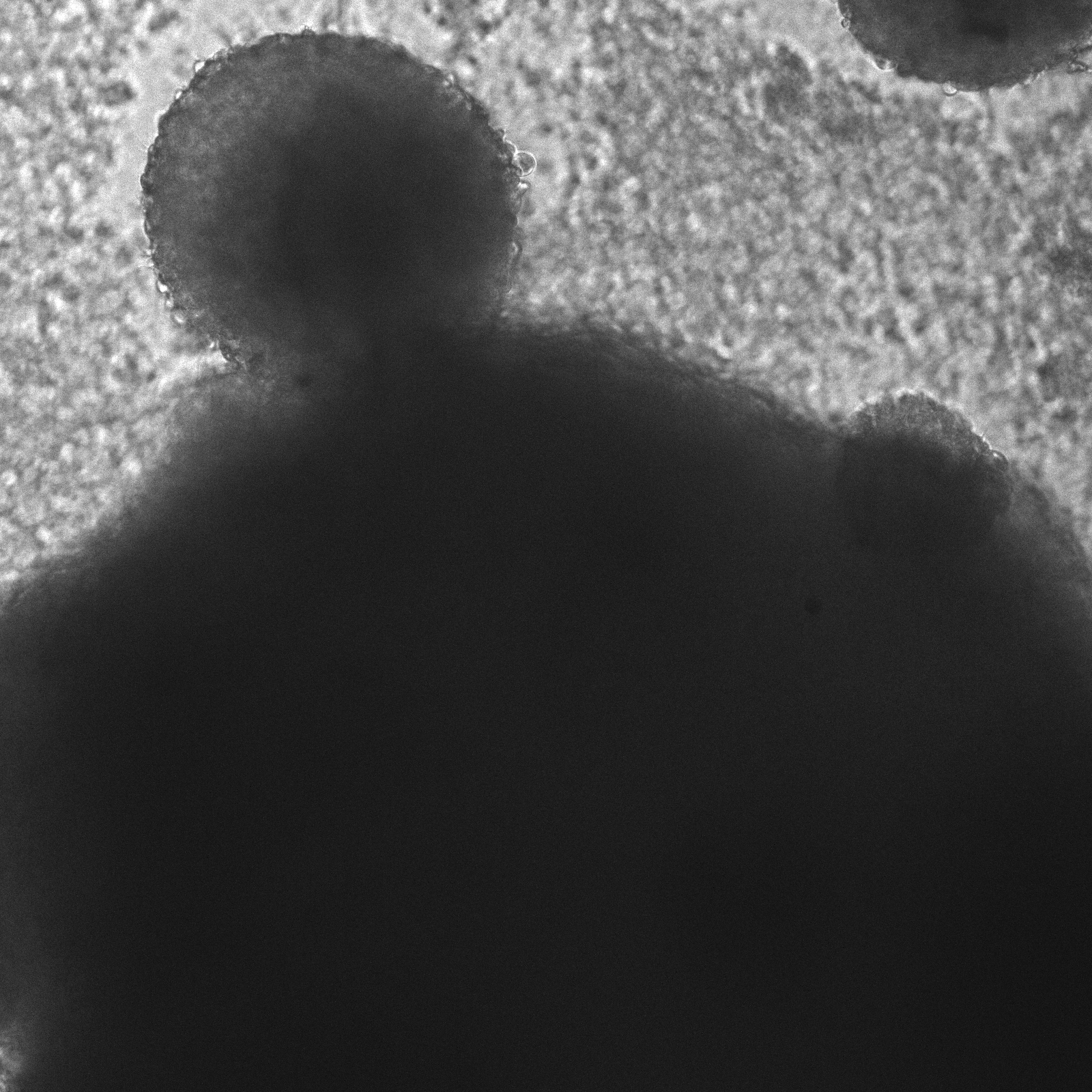

Supplement: Supplementary file 6 — Source Data for Figure 1 [file EMMM-15-e18199-s012.zip › Figure_1A,D,E/1D/Tumor_#1_D21.tif]

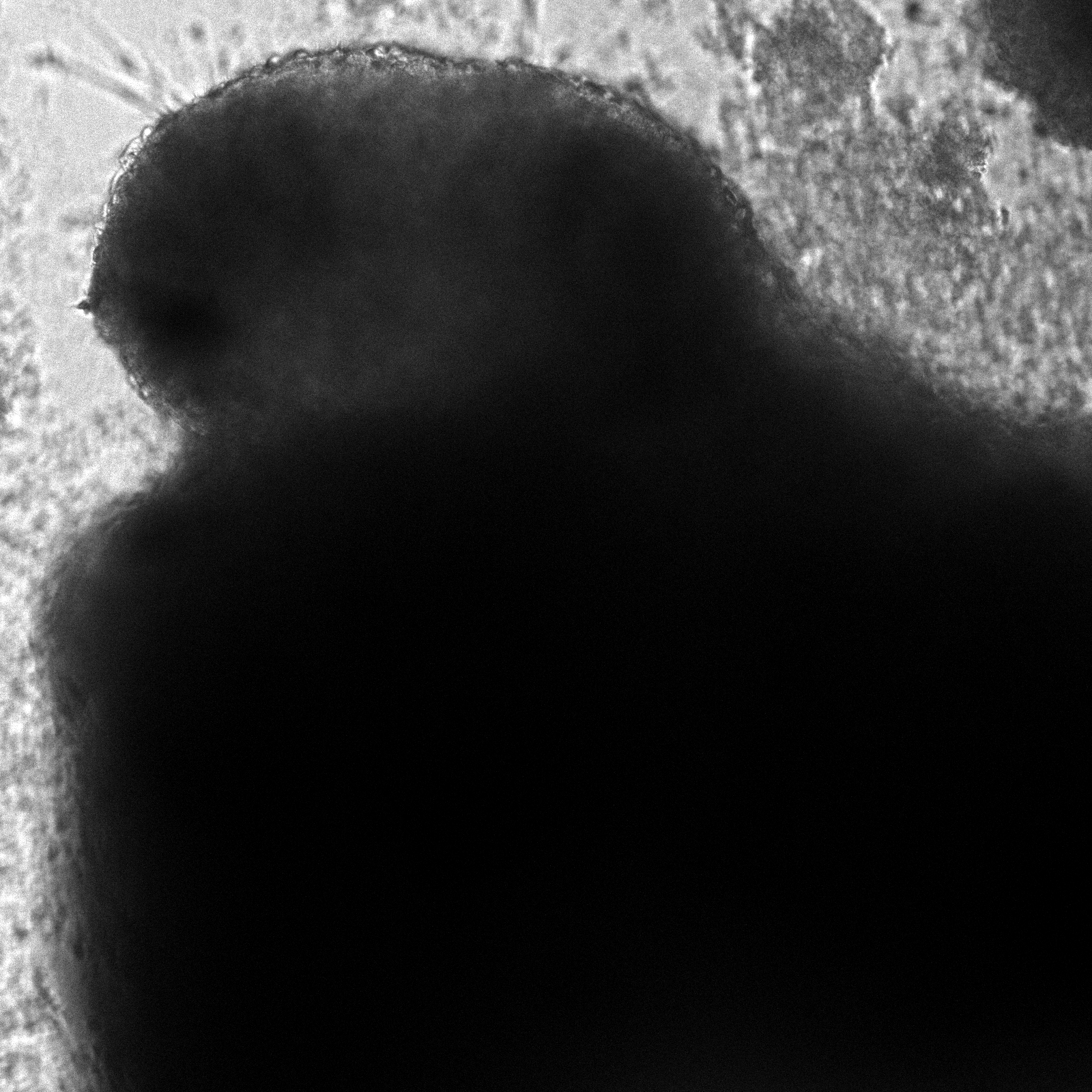

Supplement: Supplementary file 6 — Source Data for Figure 1 [file EMMM-15-e18199-s012.zip › Figure_1A,D,E/1D/Tumor_#1_D28.tif]

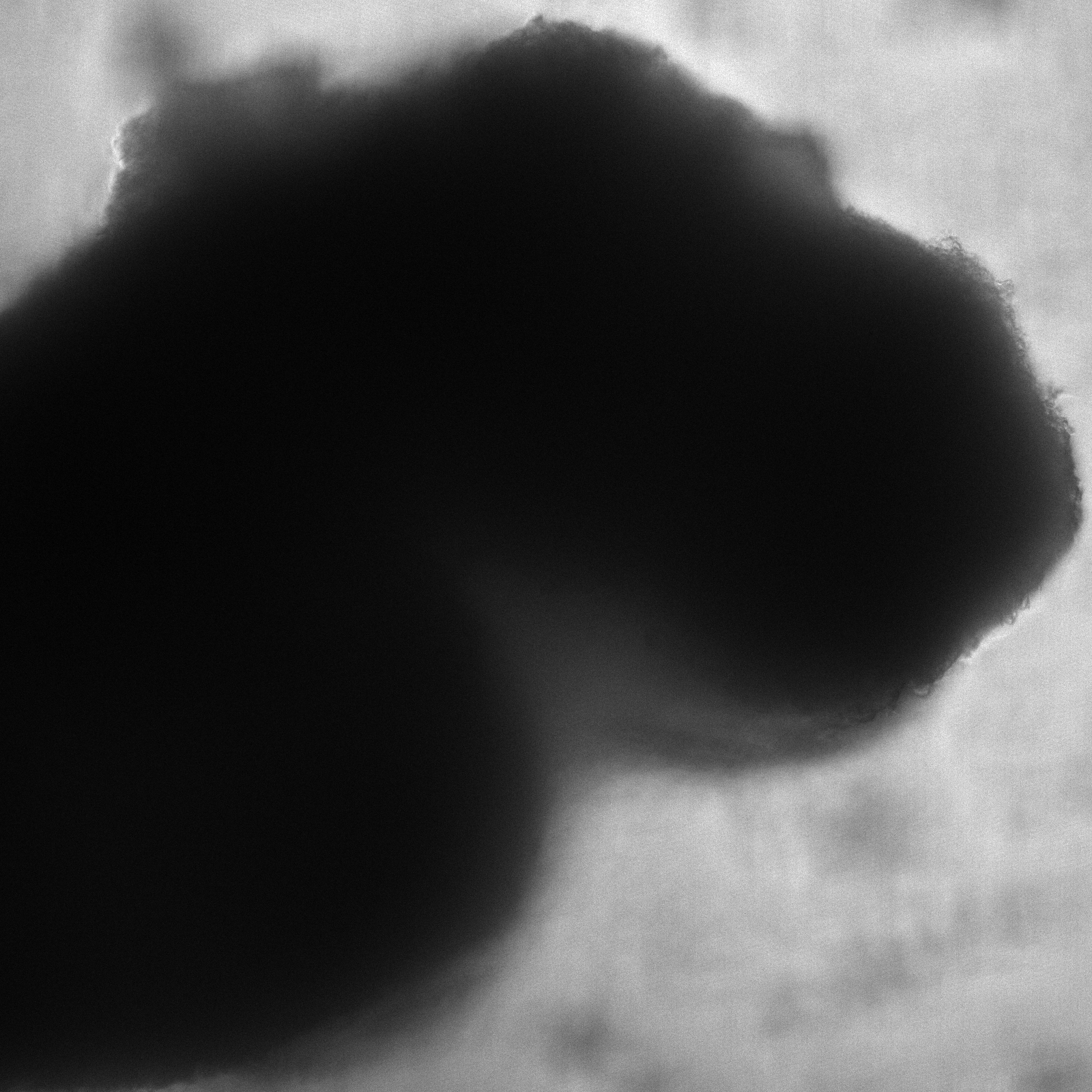

Supplement: Supplementary file 6 — Source Data for Figure 1 [file EMMM-15-e18199-s012.zip › Figure_1A,D,E/1D/Tumor_#1_D35.tif]

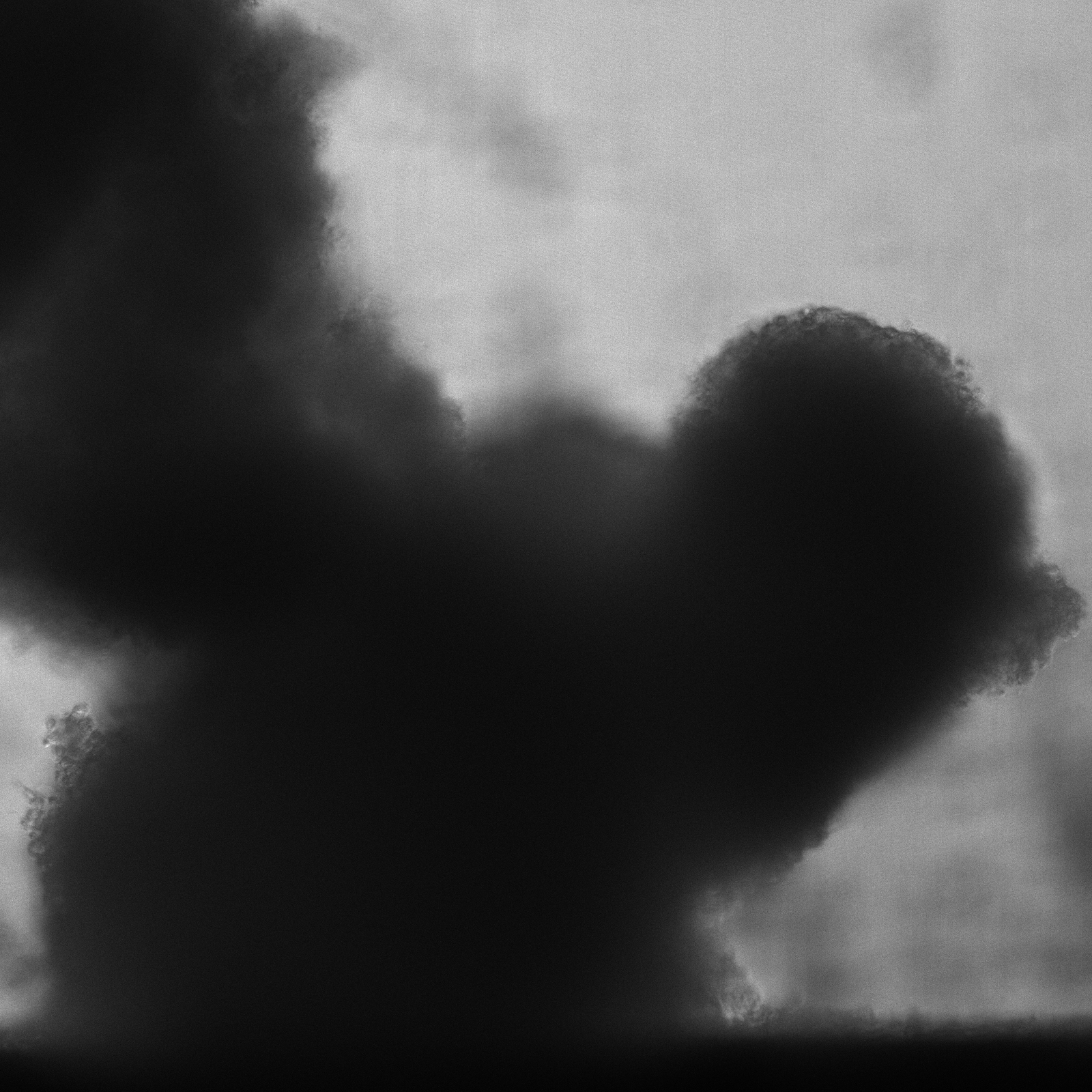

Supplement: Supplementary file 6 — Source Data for Figure 1 [file EMMM-15-e18199-s012.zip › Figure_1A,D,E/1D/Tumor_#1_D56.tif]

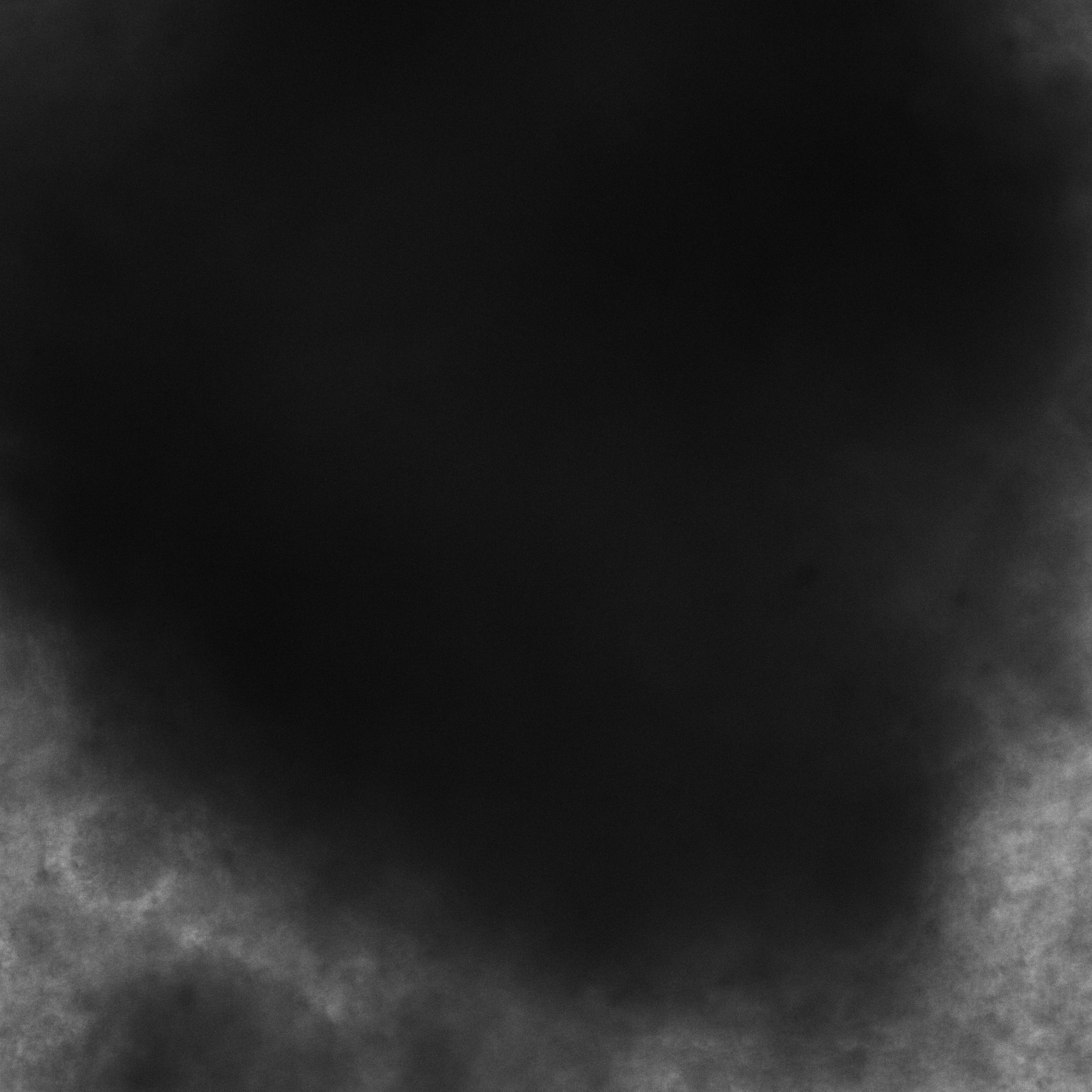

Supplement: Supplementary file 6 — Source Data for Figure 1 [file EMMM-15-e18199-s012.zip › Figure_1A,D,E/1D/Tumor_#1_D7.tif]

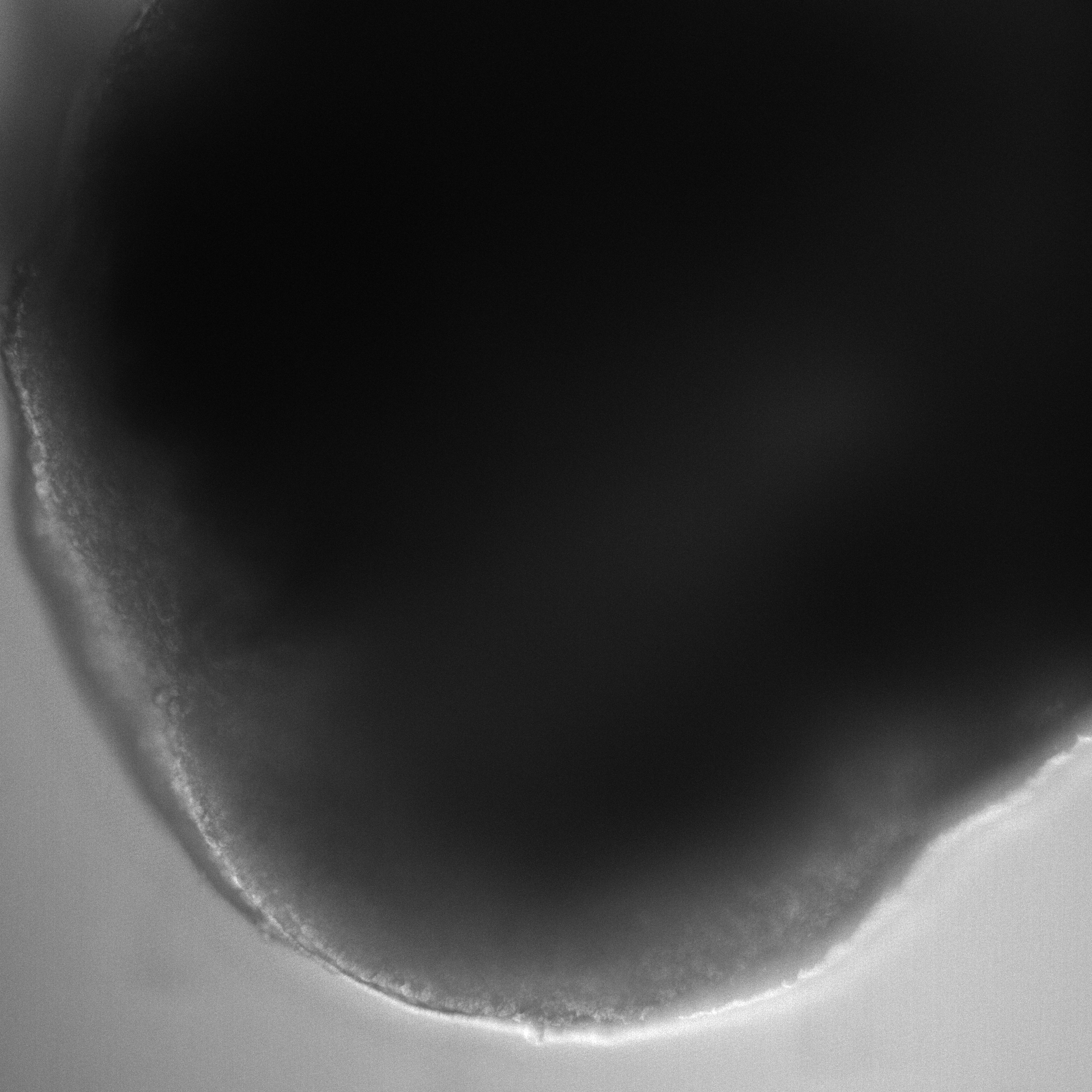

Supplement: Supplementary file 6 — Source Data for Figure 1 [file EMMM-15-e18199-s012.zip › Figure_1A,D,E/1D/Tumor_#2_D14.tif]

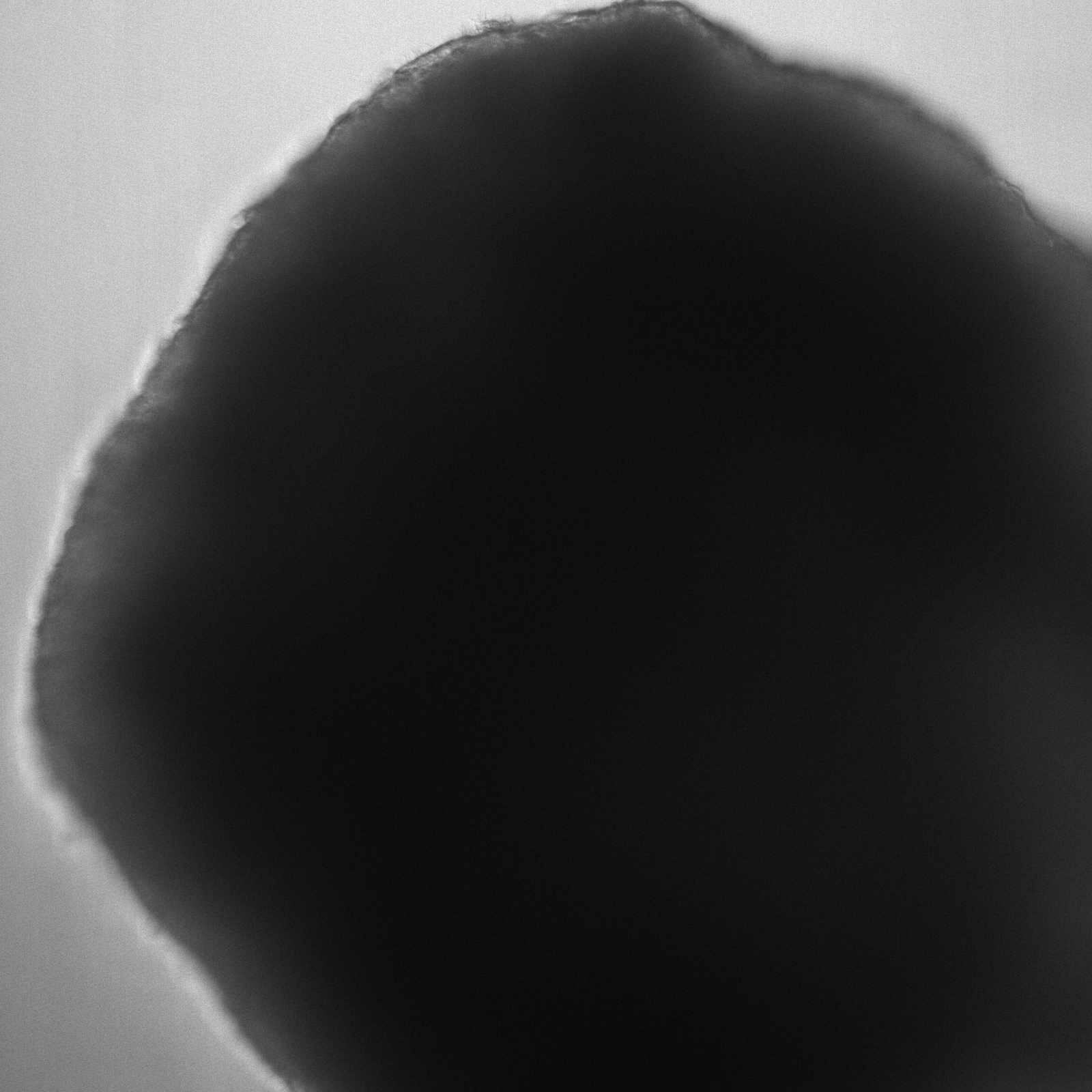

Supplement: Supplementary file 6 — Source Data for Figure 1 [file EMMM-15-e18199-s012.zip › Figure_1A,D,E/1D/Tumor_#2_D21.tif]

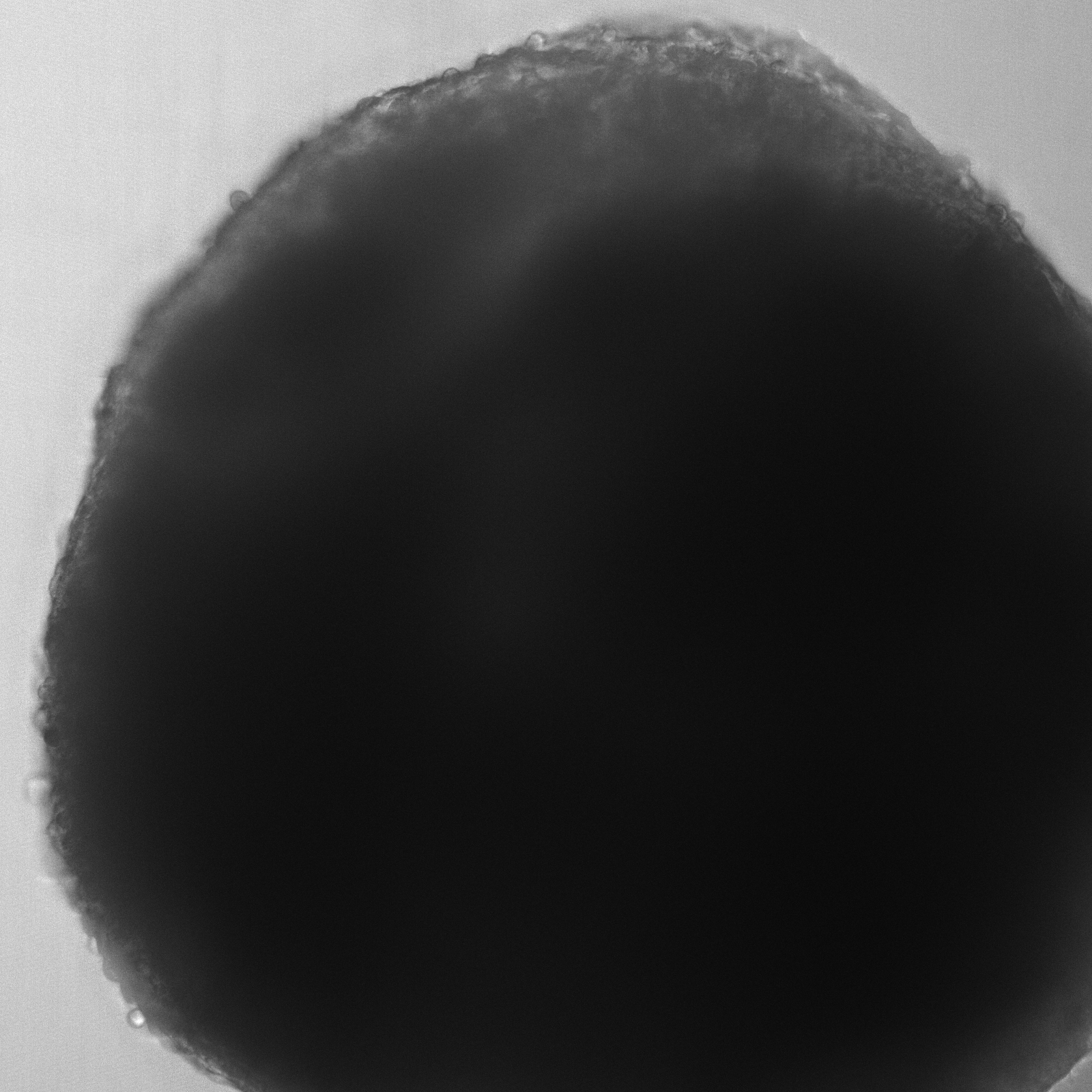

Supplement: Supplementary file 6 — Source Data for Figure 1 [file EMMM-15-e18199-s012.zip › Figure_1A,D,E/1D/Tumor_#2_D28.tif]

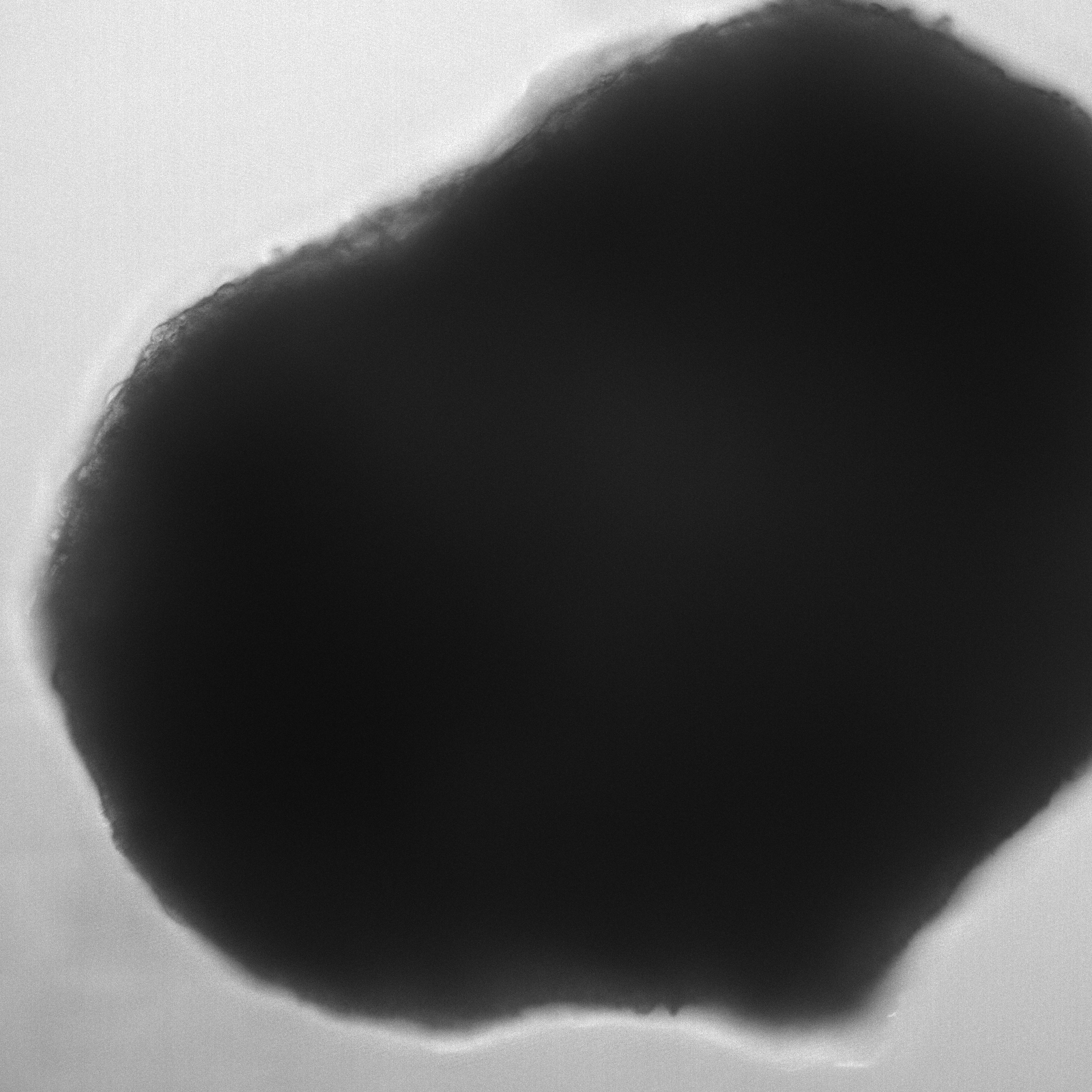

Supplement: Supplementary file 6 — Source Data for Figure 1 [file EMMM-15-e18199-s012.zip › Figure_1A,D,E/1D/Tumor_#2_D35.tif]

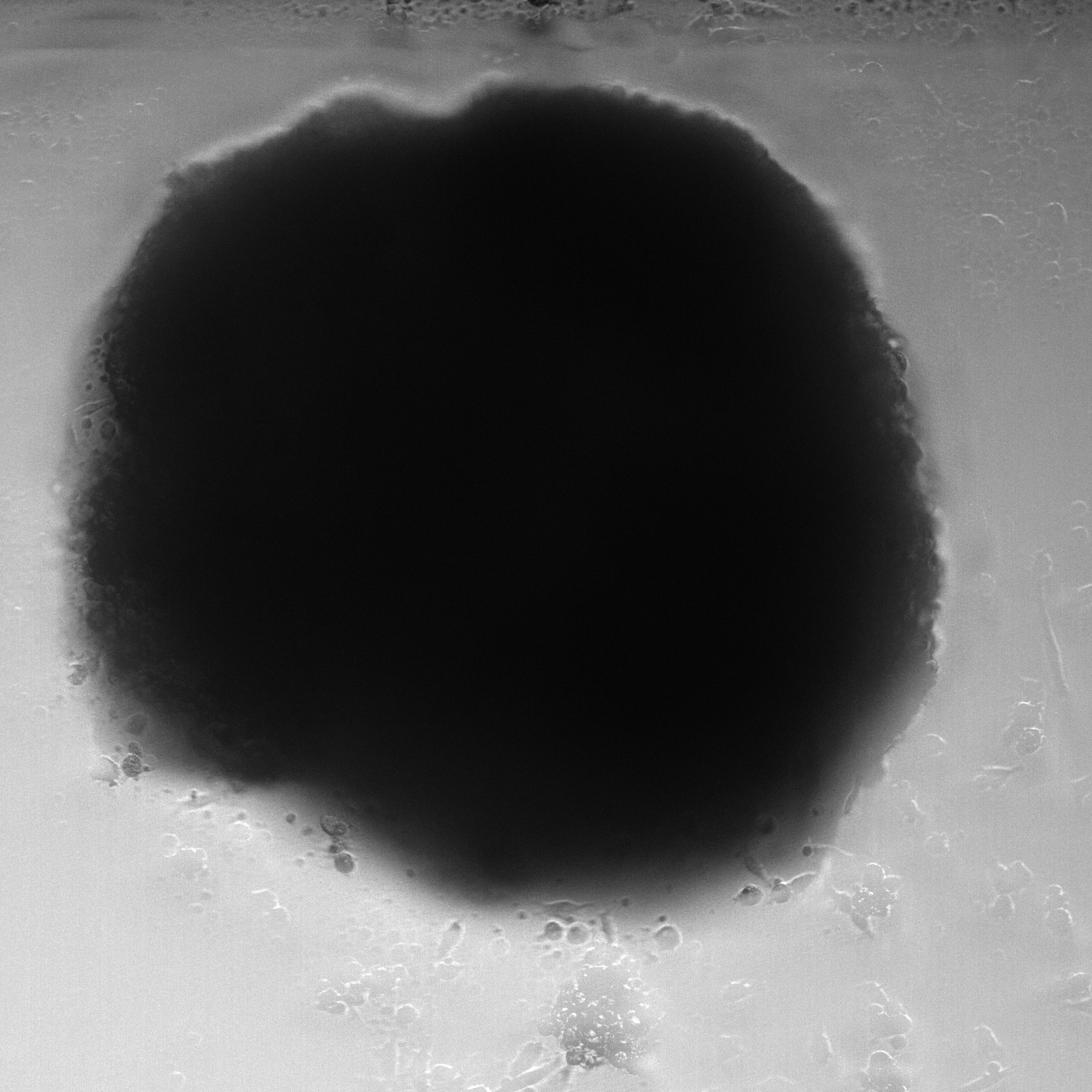

Supplement: Supplementary file 6 — Source Data for Figure 1 [file EMMM-15-e18199-s012.zip › Figure_1A,D,E/1D/Tumor_#2_D56.tif]

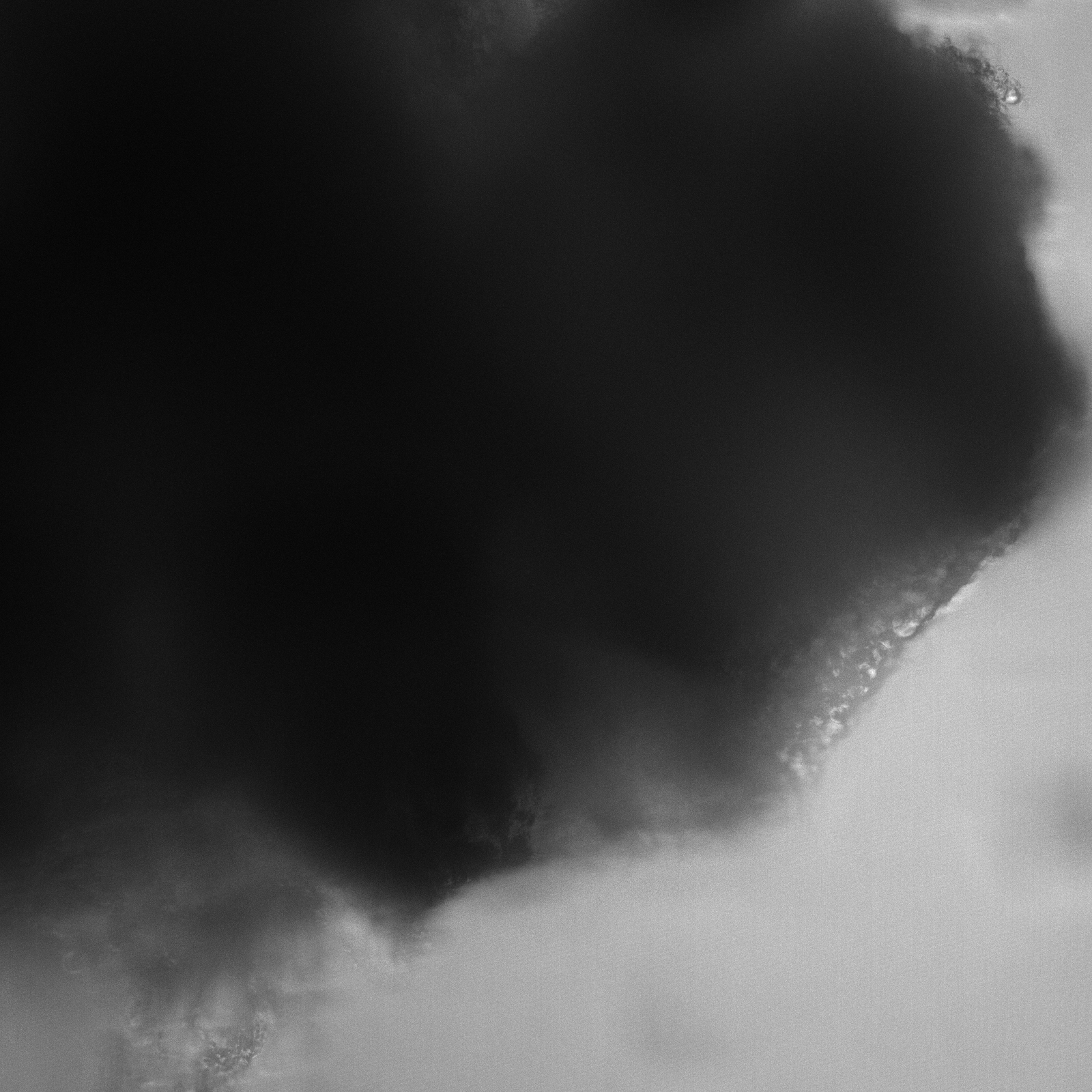

Supplement: Supplementary file 6 — Source Data for Figure 1 [file EMMM-15-e18199-s012.zip › Figure_1A,D,E/1D/Tumor_#2_D7.tif]

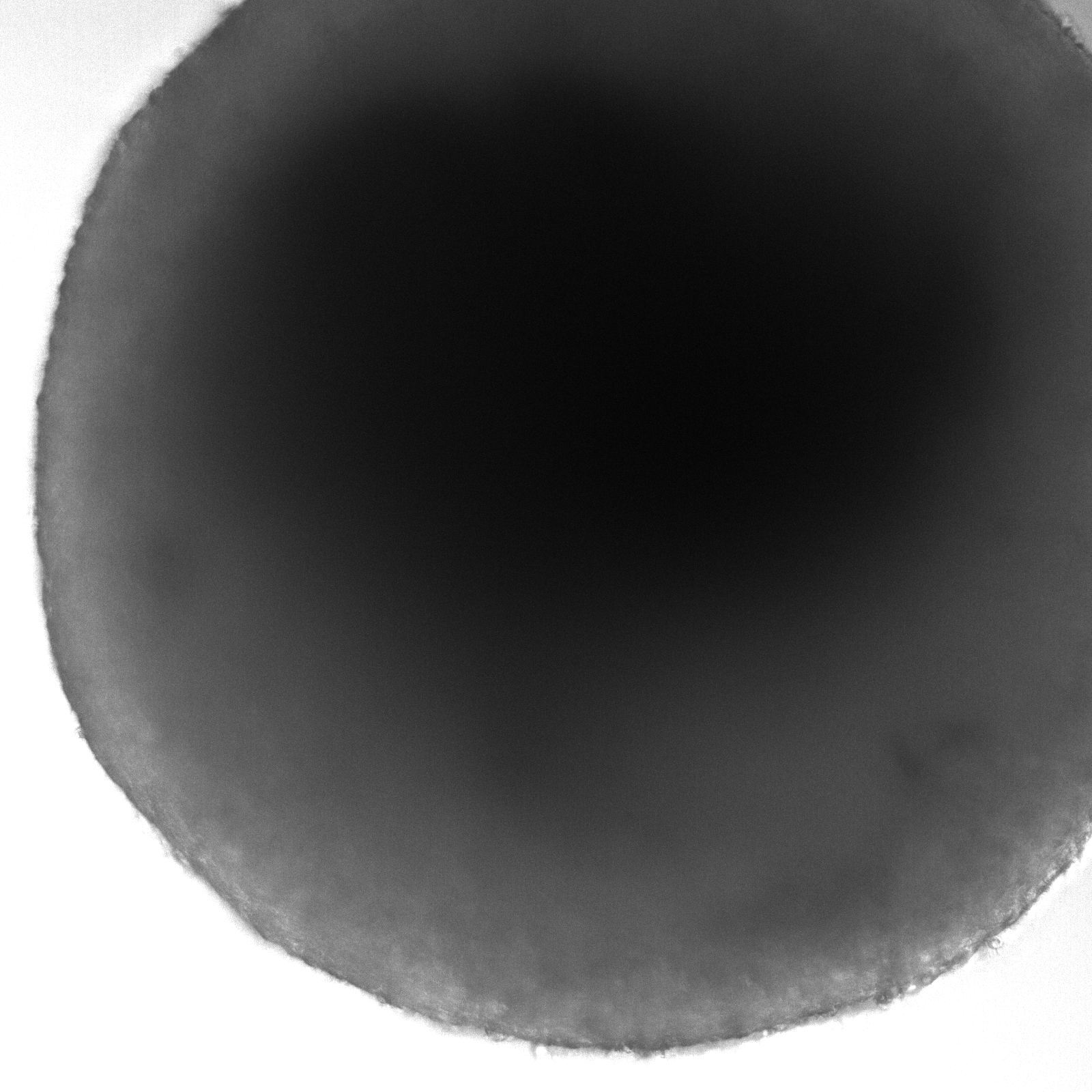

Supplement: Supplementary file 6 — Source Data for Figure 1 [file EMMM-15-e18199-s012.zip › Figure_1A,D,E/1D/Tumor_#3_D14.tif]

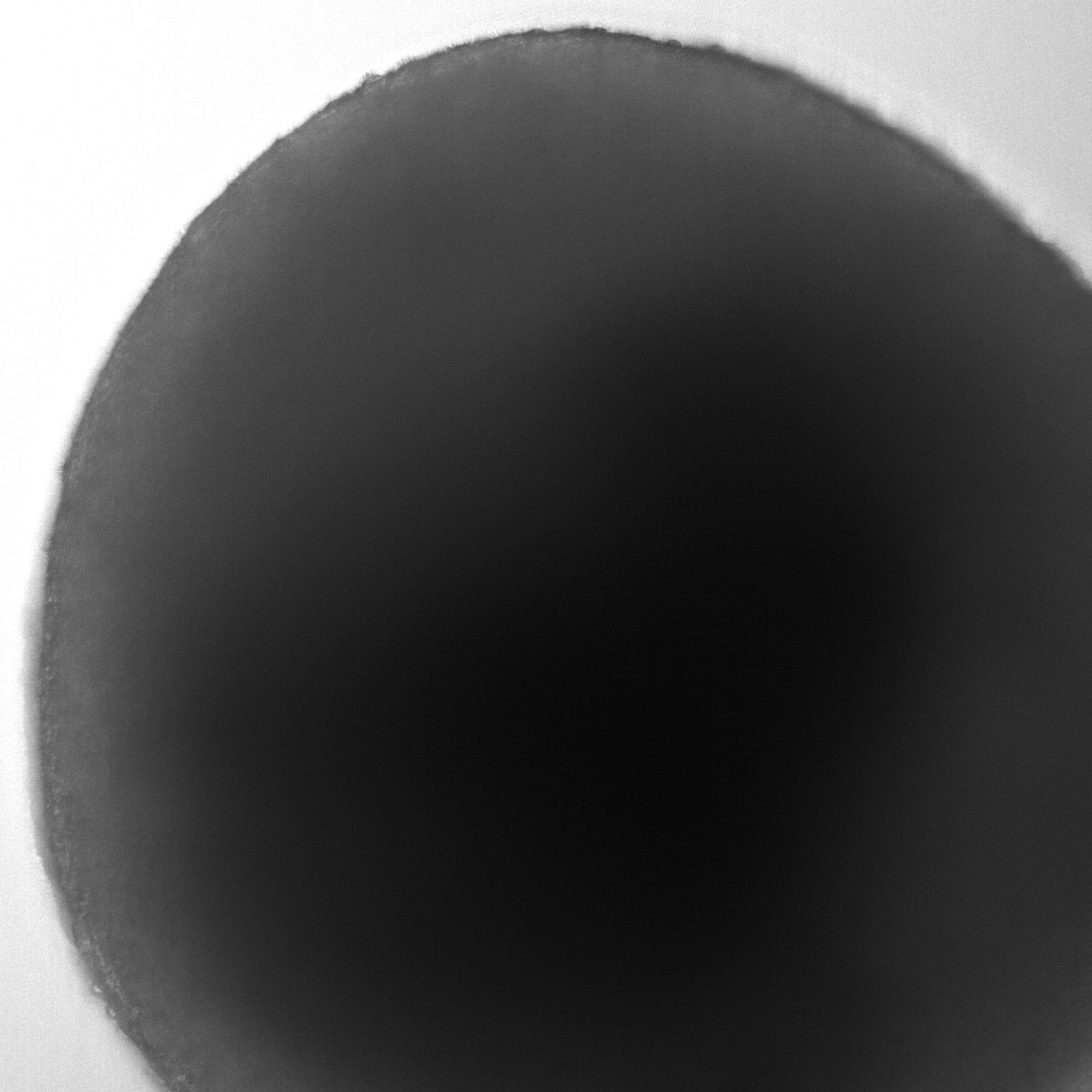

Supplement: Supplementary file 6 — Source Data for Figure 1 [file EMMM-15-e18199-s012.zip › Figure_1A,D,E/1D/Tumor_#3_D21.tif]

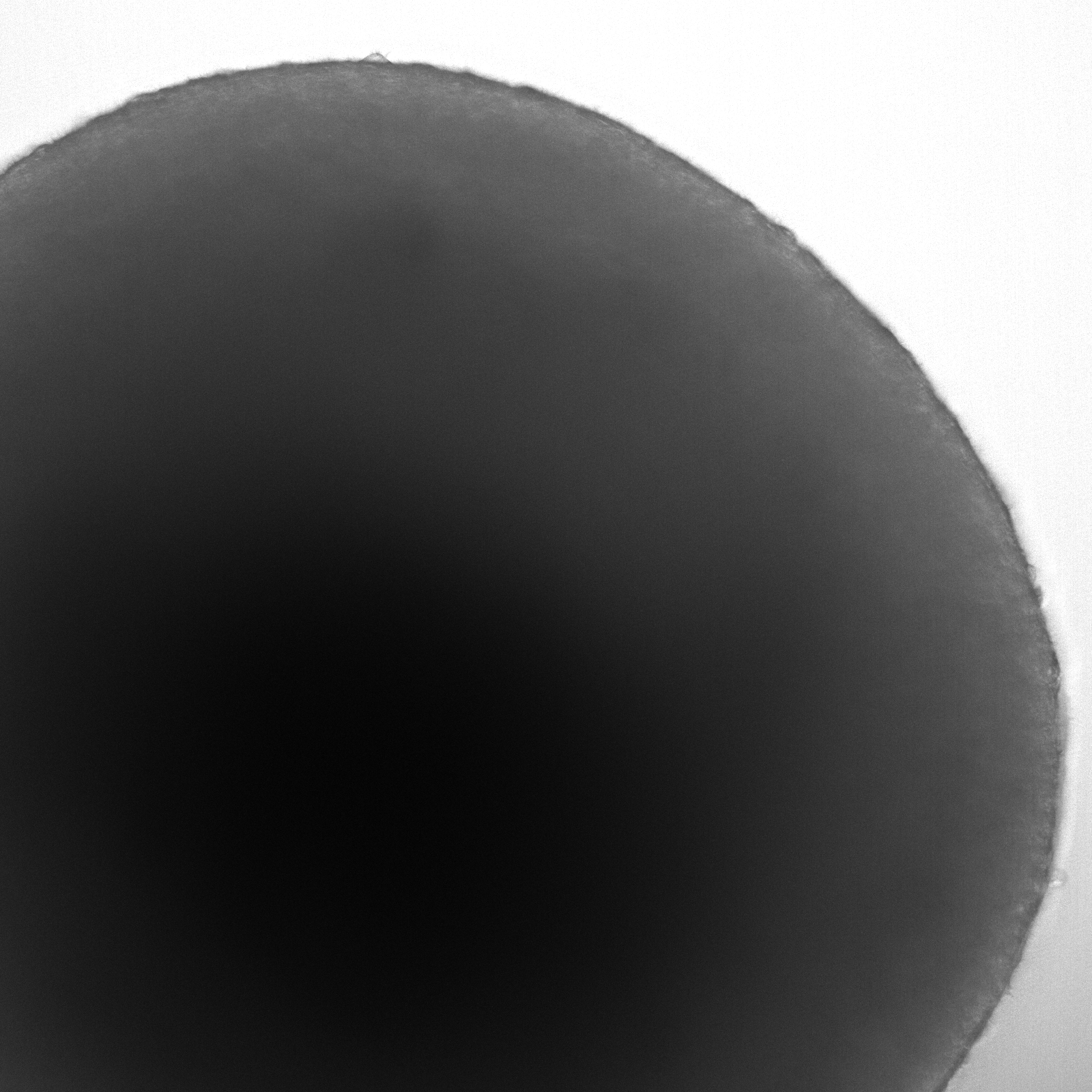

Supplement: Supplementary file 6 — Source Data for Figure 1 [file EMMM-15-e18199-s012.zip › Figure_1A,D,E/1D/Tumor_#3_D28.tif]

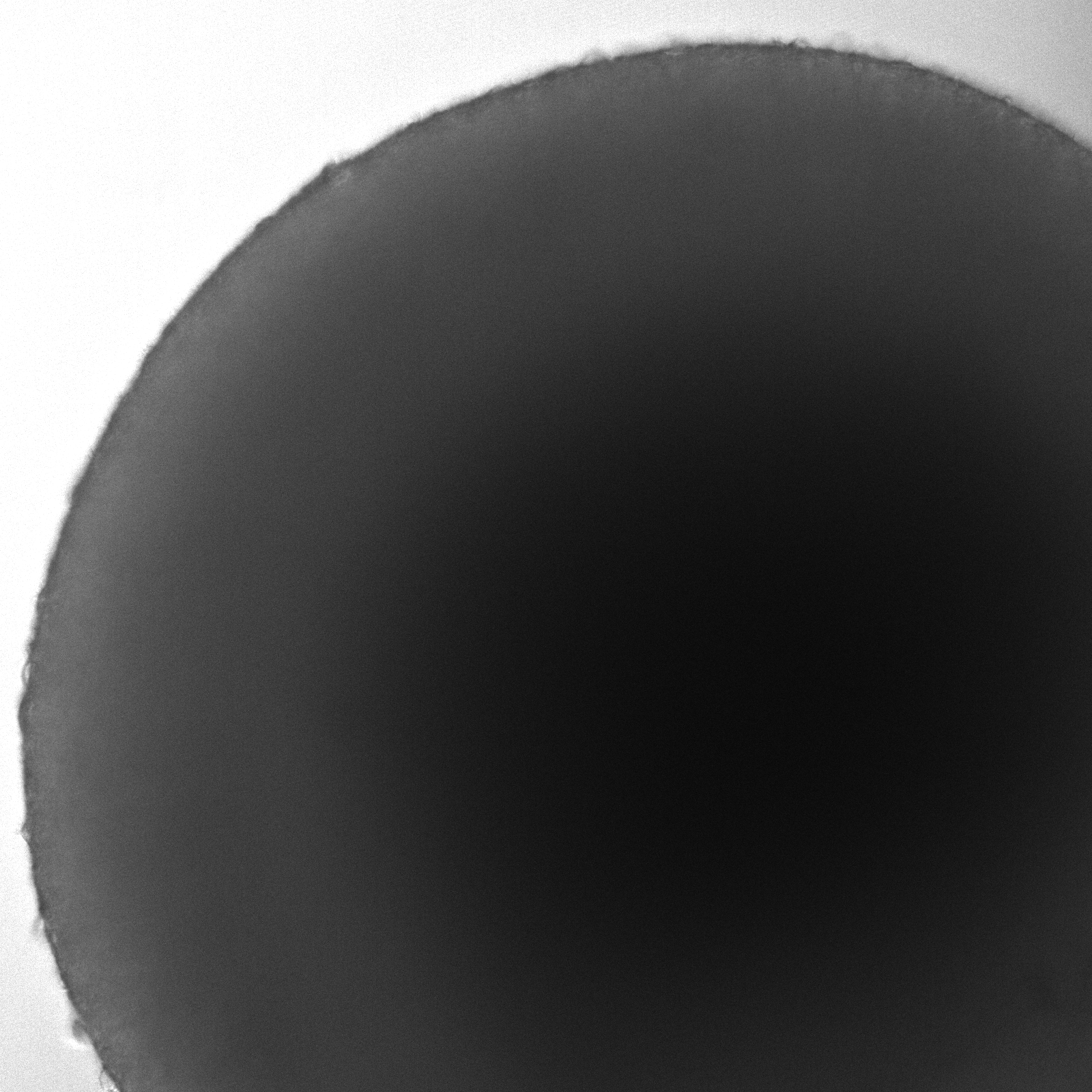

Supplement: Supplementary file 6 — Source Data for Figure 1 [file EMMM-15-e18199-s012.zip › Figure_1A,D,E/1D/Tumor_#3_D35.tif]

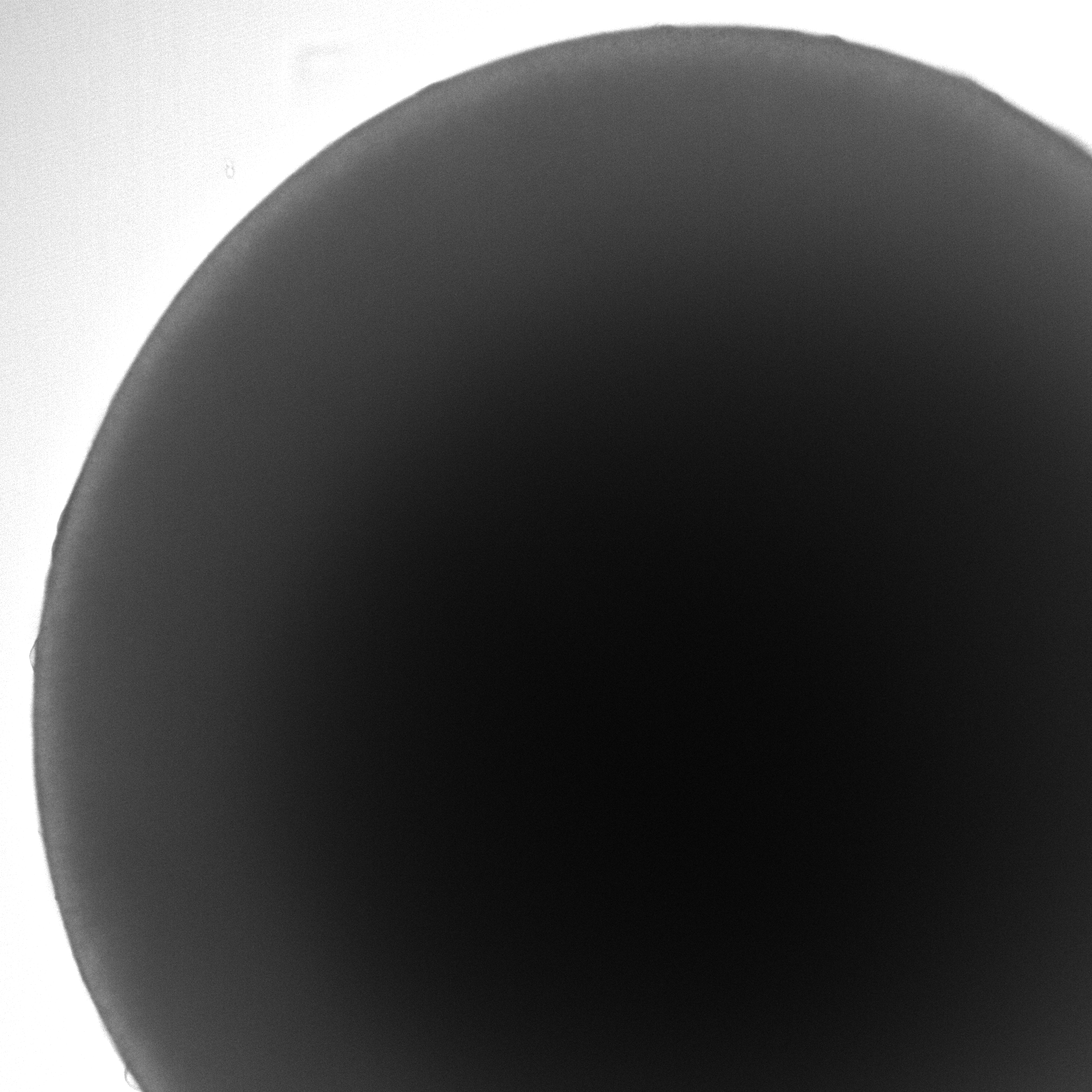

Supplement: Supplementary file 6 — Source Data for Figure 1 [file EMMM-15-e18199-s012.zip › Figure_1A,D,E/1D/Tumor_#3_D56.tif]

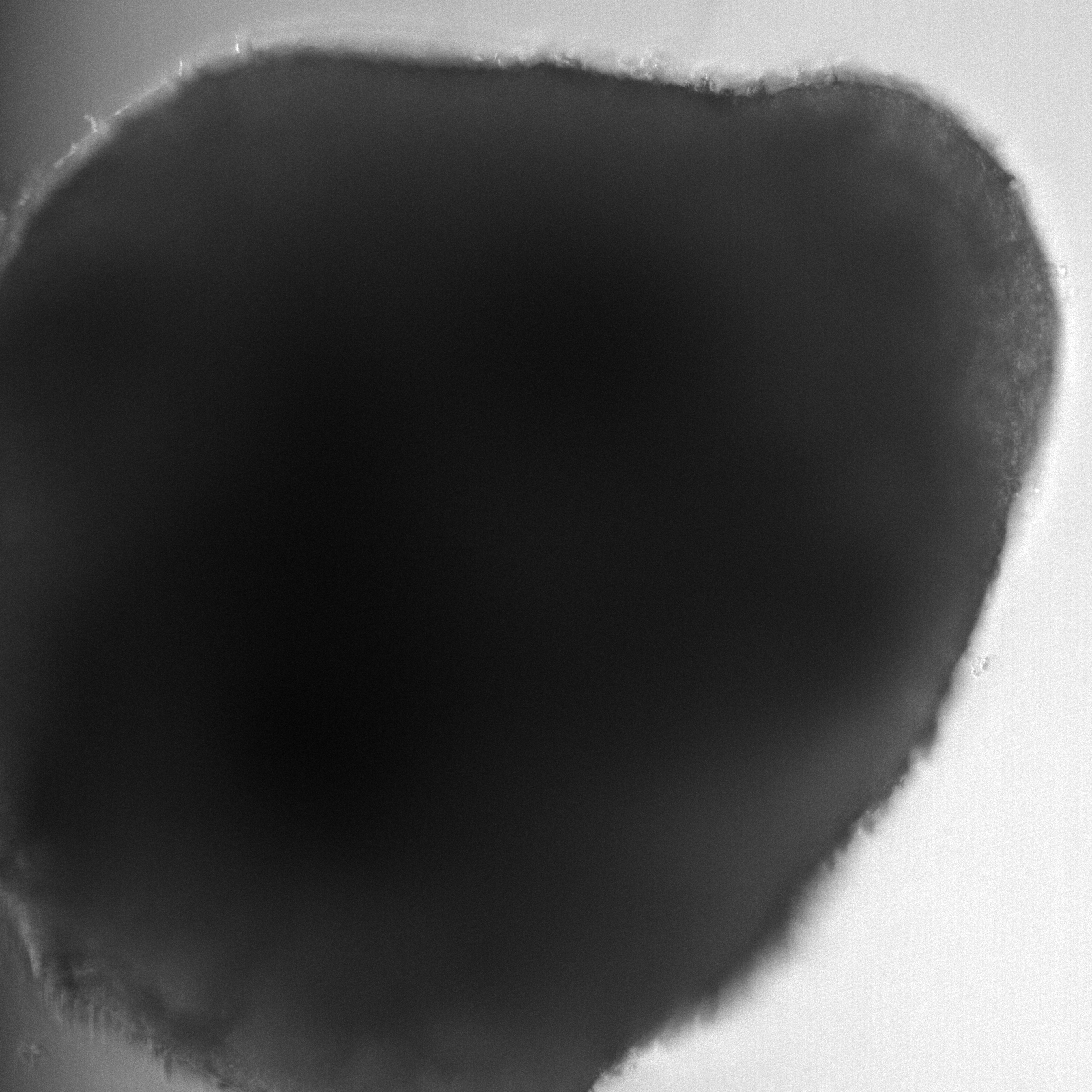

Supplement: Supplementary file 6 — Source Data for Figure 1 [file EMMM-15-e18199-s012.zip › Figure_1A,D,E/1D/Tumor_#3_D7.tif]

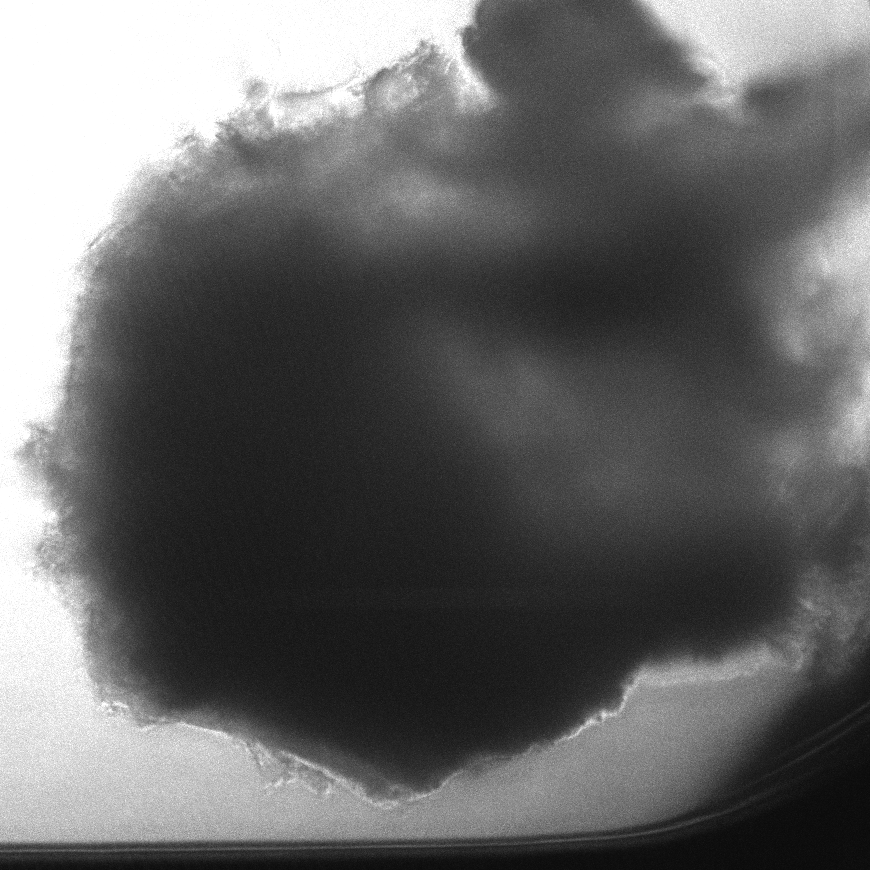

Supplement: Supplementary file 6 — Source Data for Figure 1 [file EMMM-15-e18199-s012.zip › Figure_1A,D,E/1D/Tumor_#4_D14.tif]

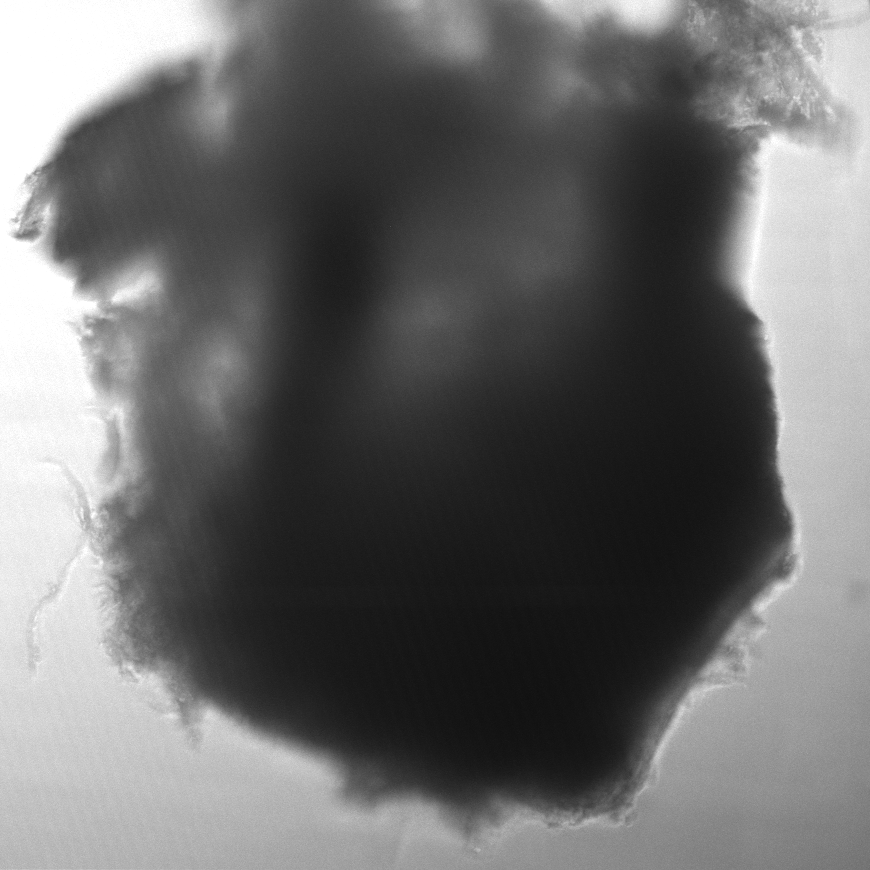

Supplement: Supplementary file 6 — Source Data for Figure 1 [file EMMM-15-e18199-s012.zip › Figure_1A,D,E/1D/Tumor_#4_D21.tif]

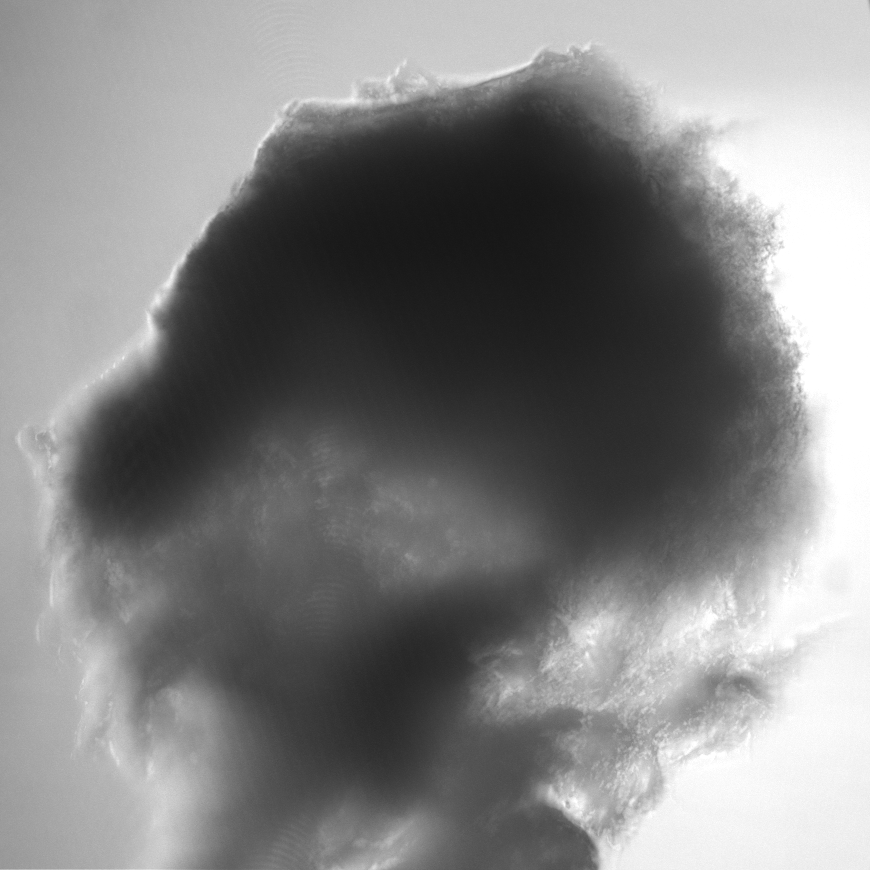

Supplement: Supplementary file 6 — Source Data for Figure 1 [file EMMM-15-e18199-s012.zip › Figure_1A,D,E/1D/Tumor_#4_D28.tif]

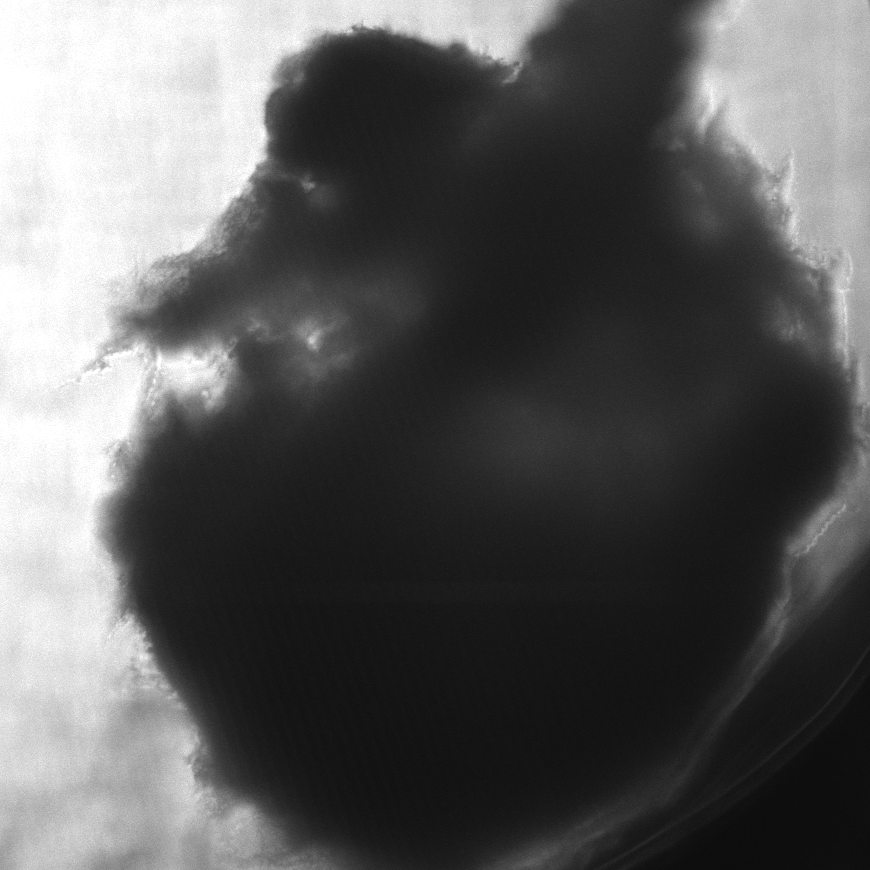

Supplement: Supplementary file 6 — Source Data for Figure 1 [file EMMM-15-e18199-s012.zip › Figure_1A,D,E/1D/Tumor_#4_D35.tif]

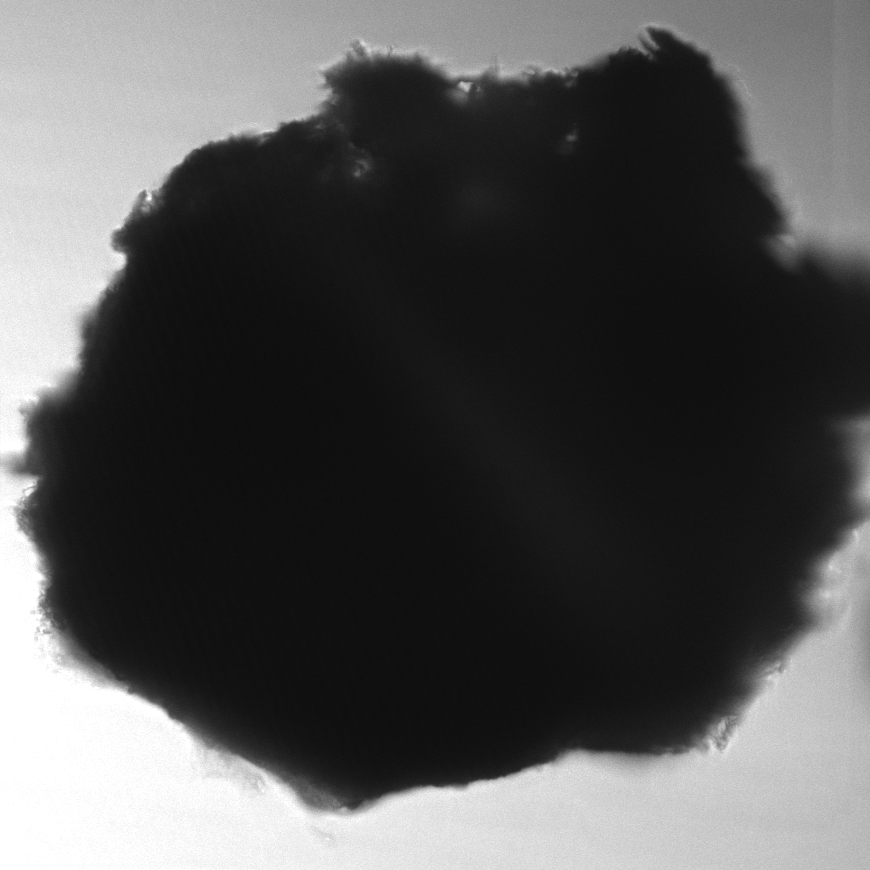

Supplement: Supplementary file 6 — Source Data for Figure 1 [file EMMM-15-e18199-s012.zip › Figure_1A,D,E/1D/Tumor_#4_D56.tif]

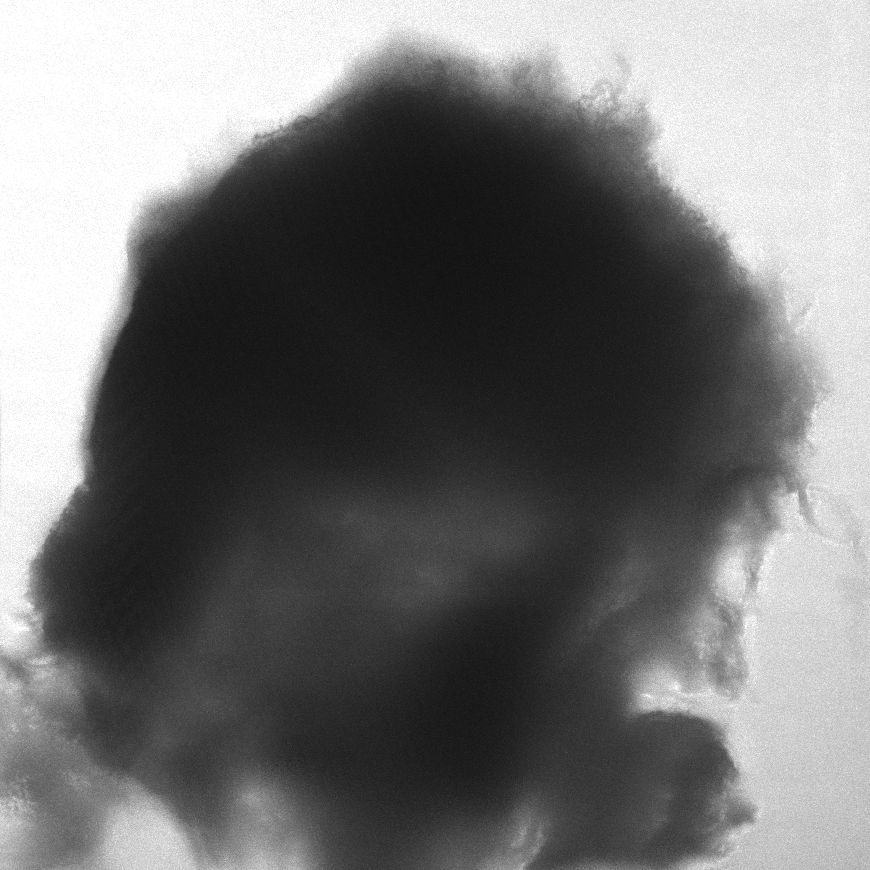

Supplement: Supplementary file 6 — Source Data for Figure 1 [file EMMM-15-e18199-s012.zip › Figure_1A,D,E/1D/Tumor_#4_D7.tif]

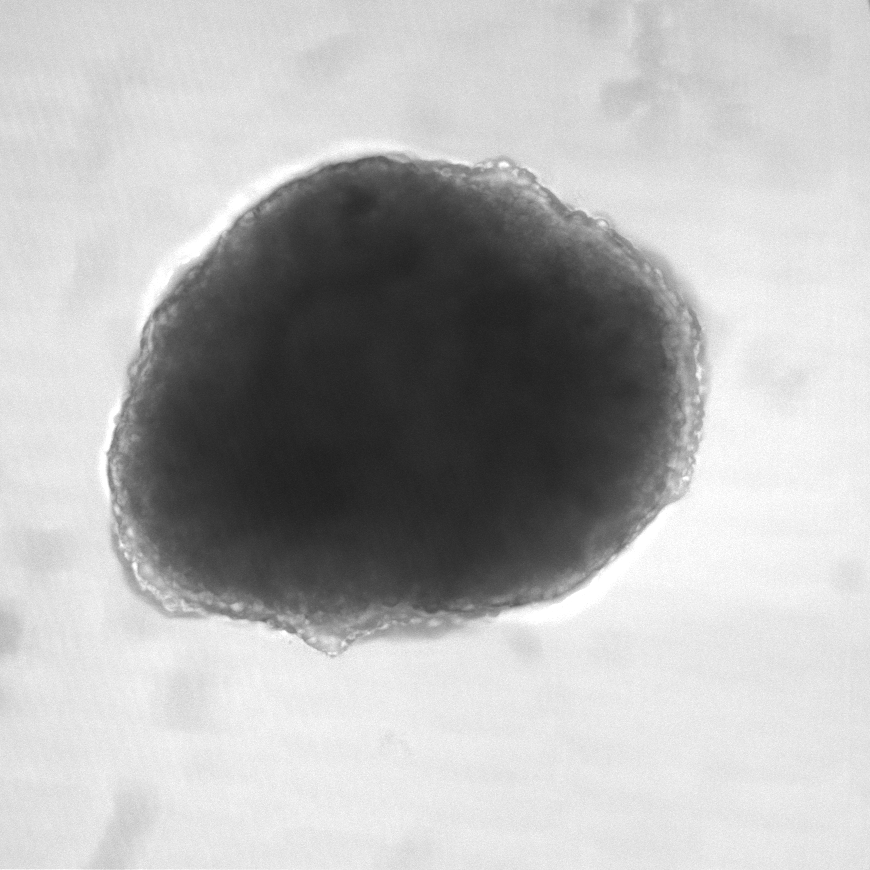

Supplement: Supplementary file 6 — Source Data for Figure 1 [file EMMM-15-e18199-s012.zip › Figure_1A,D,E/1D/Tumor_#5_D14.tif]

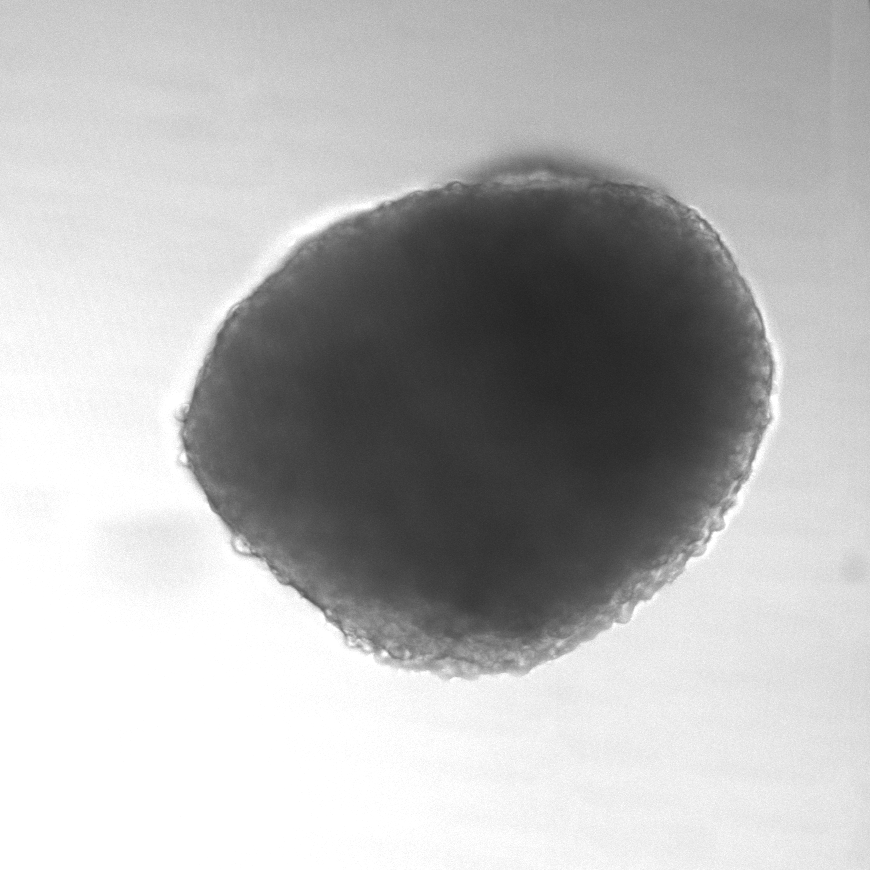

Supplement: Supplementary file 6 — Source Data for Figure 1 [file EMMM-15-e18199-s012.zip › Figure_1A,D,E/1D/Tumor_#5_D21.tif]

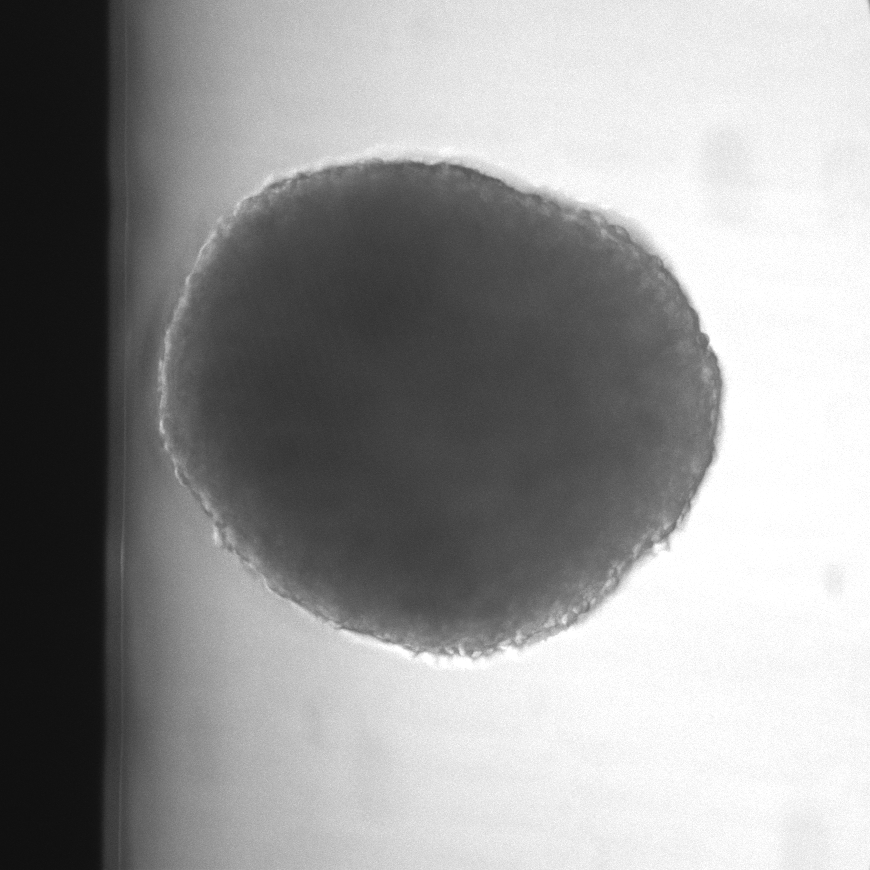

Supplement: Supplementary file 6 — Source Data for Figure 1 [file EMMM-15-e18199-s012.zip › Figure_1A,D,E/1D/Tumor_#5_D28.tif]

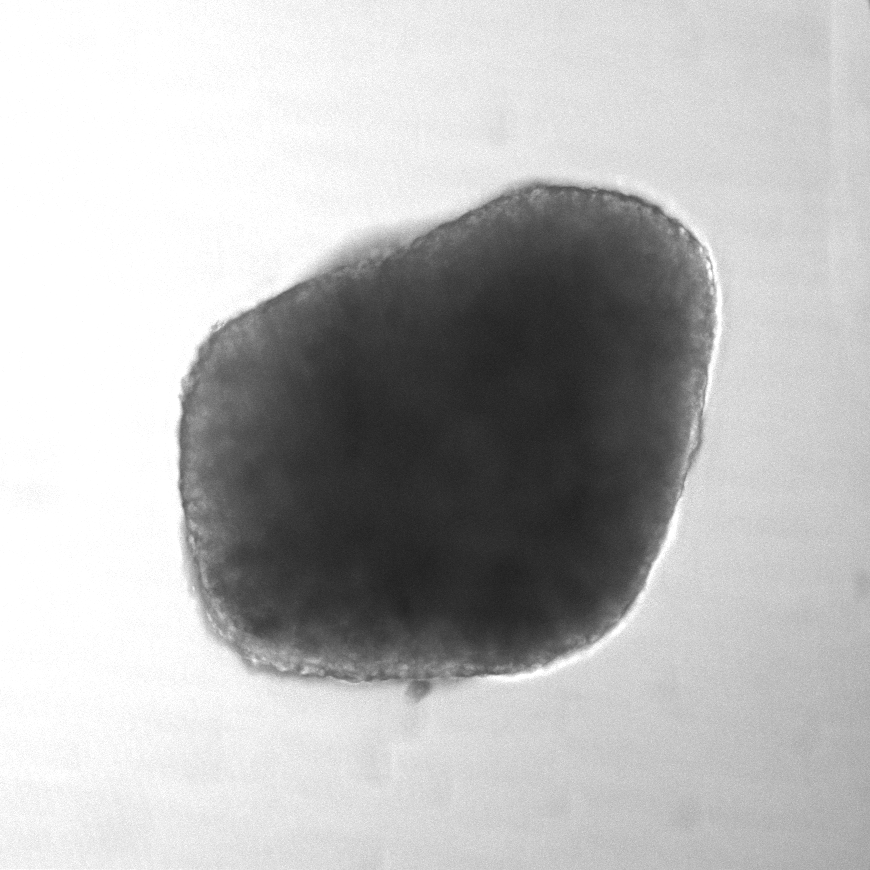

Supplement: Supplementary file 6 — Source Data for Figure 1 [file EMMM-15-e18199-s012.zip › Figure_1A,D,E/1D/Tumor_#5_D7.tif]

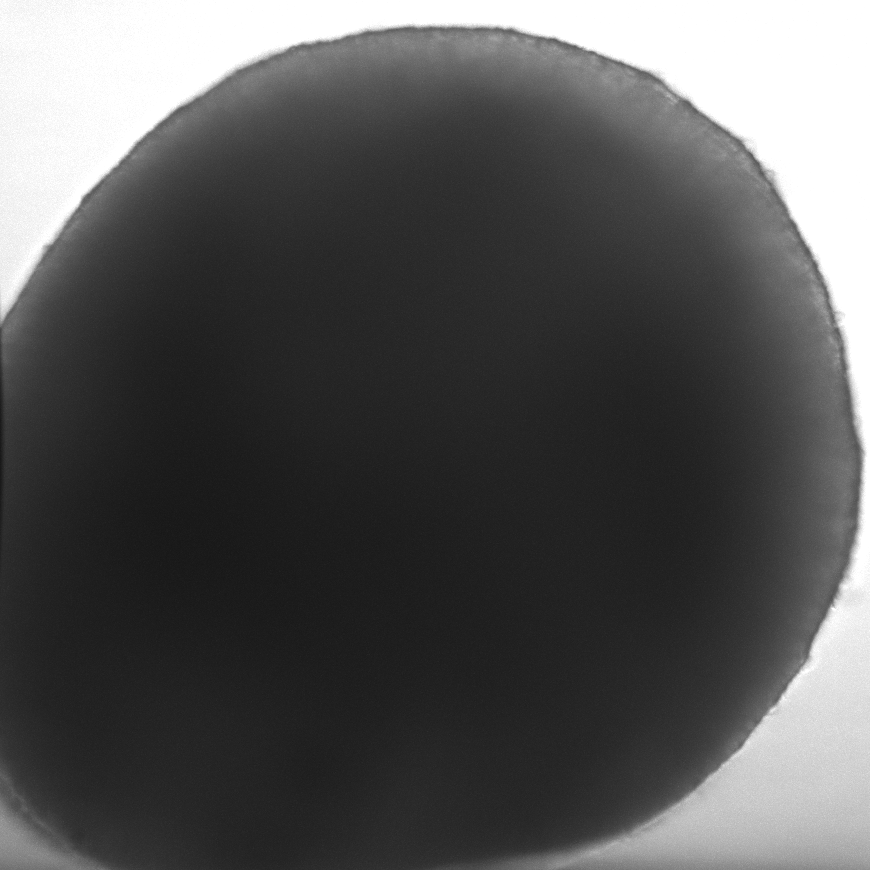

Supplement: Supplementary file 6 — Source Data for Figure 1 [file EMMM-15-e18199-s012.zip › Figure_1A,D,E/1D/Tumor_#6_D14.tif]

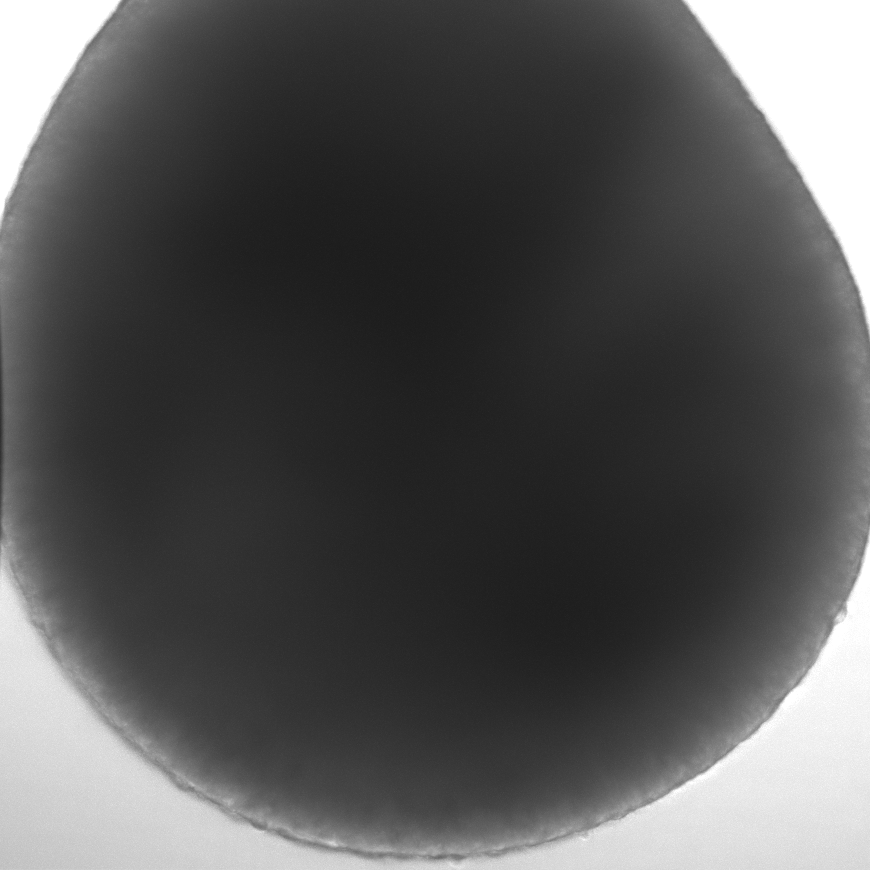

Supplement: Supplementary file 6 — Source Data for Figure 1 [file EMMM-15-e18199-s012.zip › Figure_1A,D,E/1D/Tumor_#6_D21.tif]

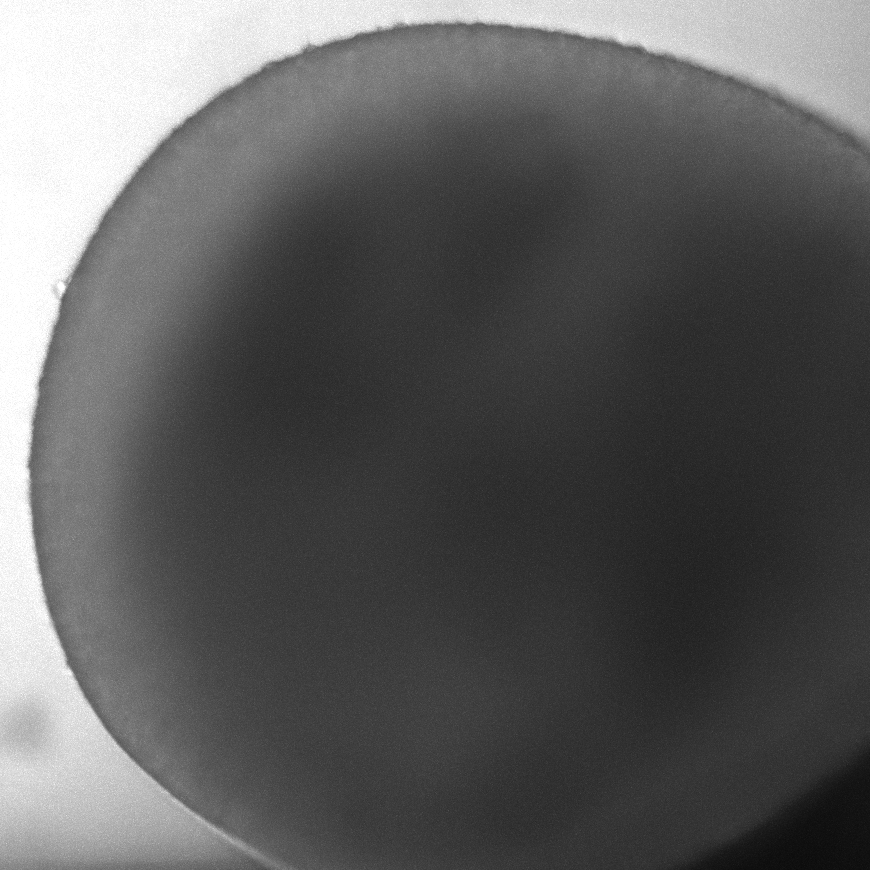

Supplement: Supplementary file 6 — Source Data for Figure 1 [file EMMM-15-e18199-s012.zip › Figure_1A,D,E/1D/Tumor_#6_D28.tif]

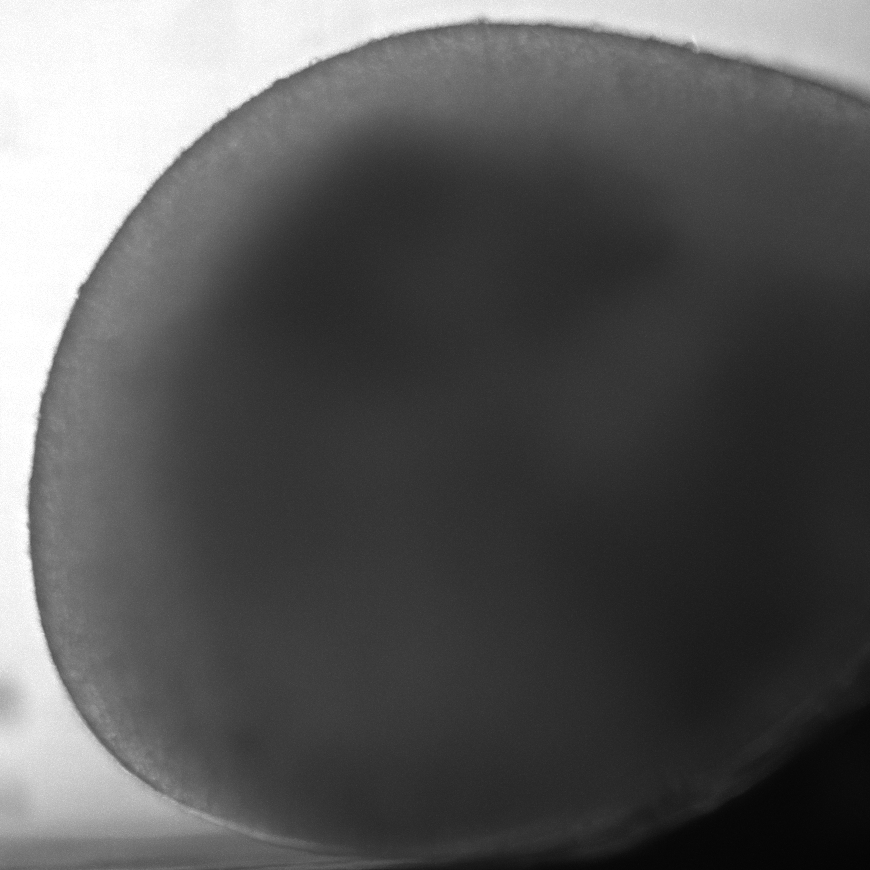

Supplement: Supplementary file 6 — Source Data for Figure 1 [file EMMM-15-e18199-s012.zip › Figure_1A,D,E/1D/Tumor_#6_D35.tif]

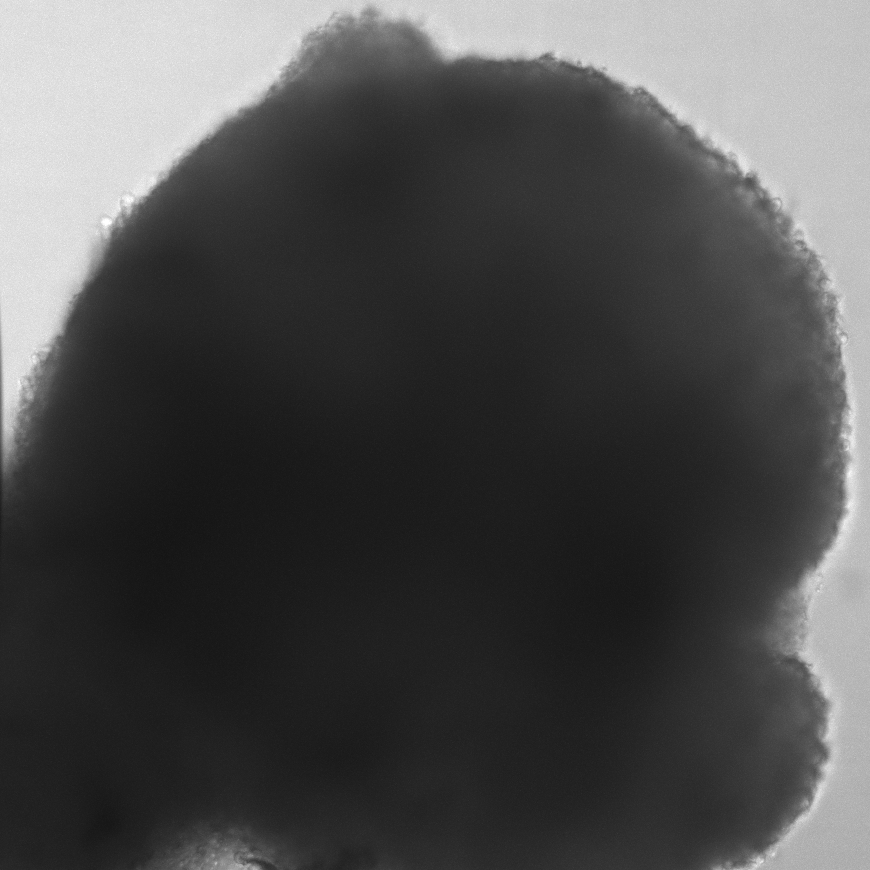

Supplement: Supplementary file 6 — Source Data for Figure 1 [file EMMM-15-e18199-s012.zip › Figure_1A,D,E/1D/Tumor_#6_D7.tif]

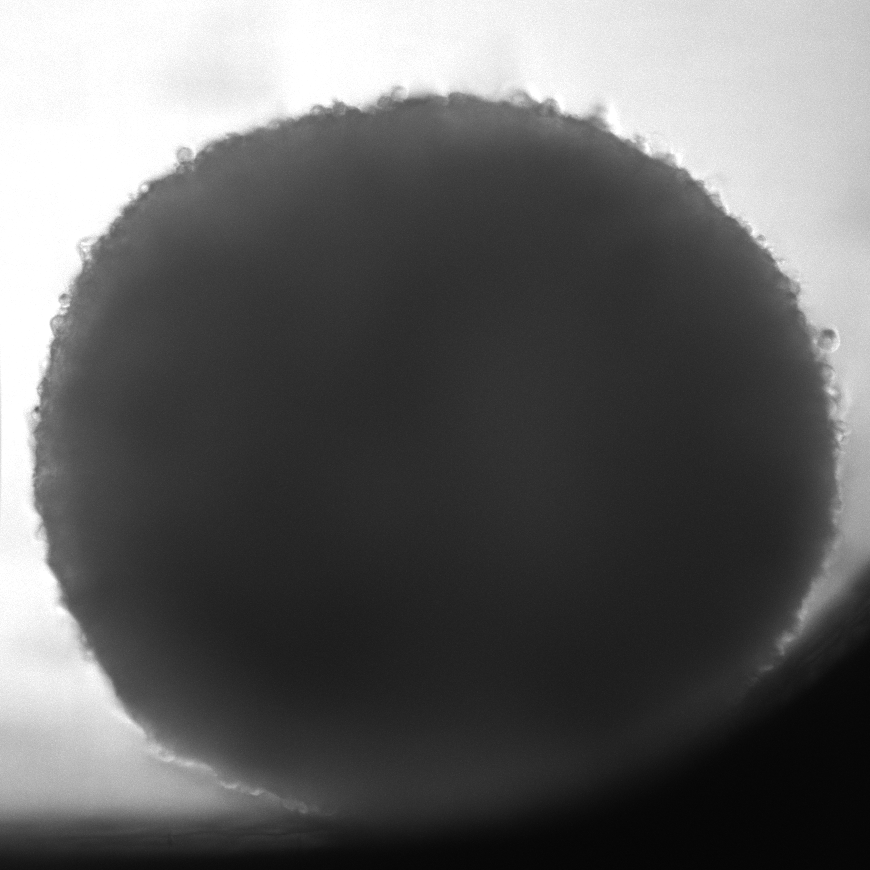

Supplement: Supplementary file 6 — Source Data for Figure 1 [file EMMM-15-e18199-s012.zip › Figure_1A,D,E/1D/Tumor_#7_D14.tif]

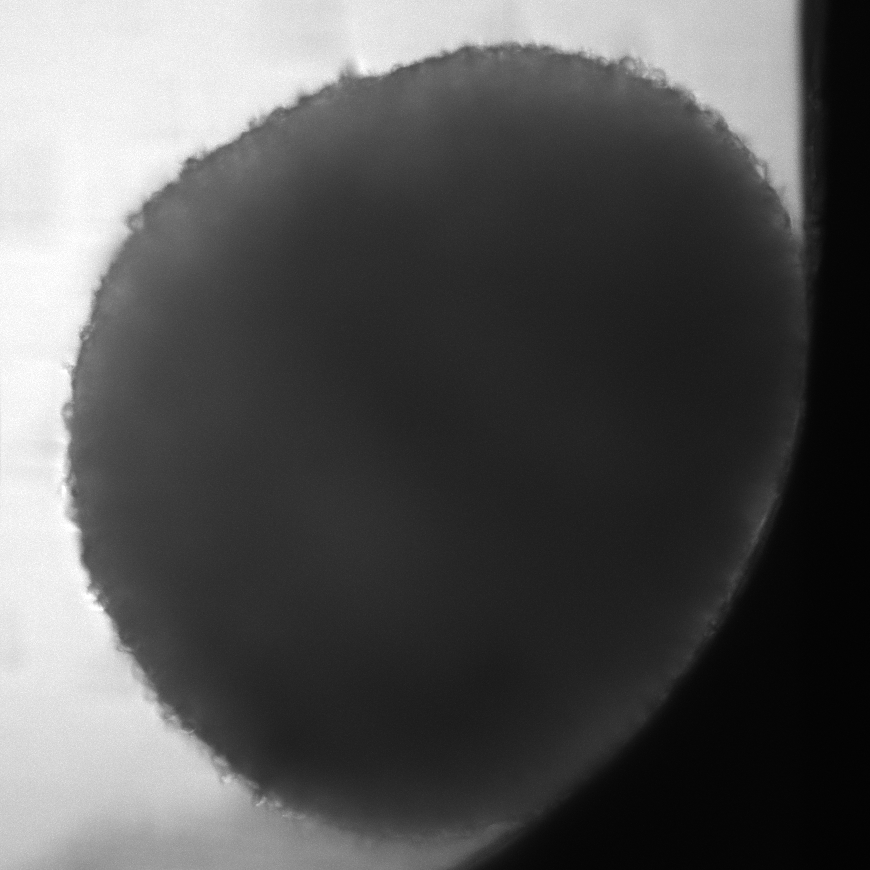

Supplement: Supplementary file 6 — Source Data for Figure 1 [file EMMM-15-e18199-s012.zip › Figure_1A,D,E/1D/Tumor_#7_D21.tif]

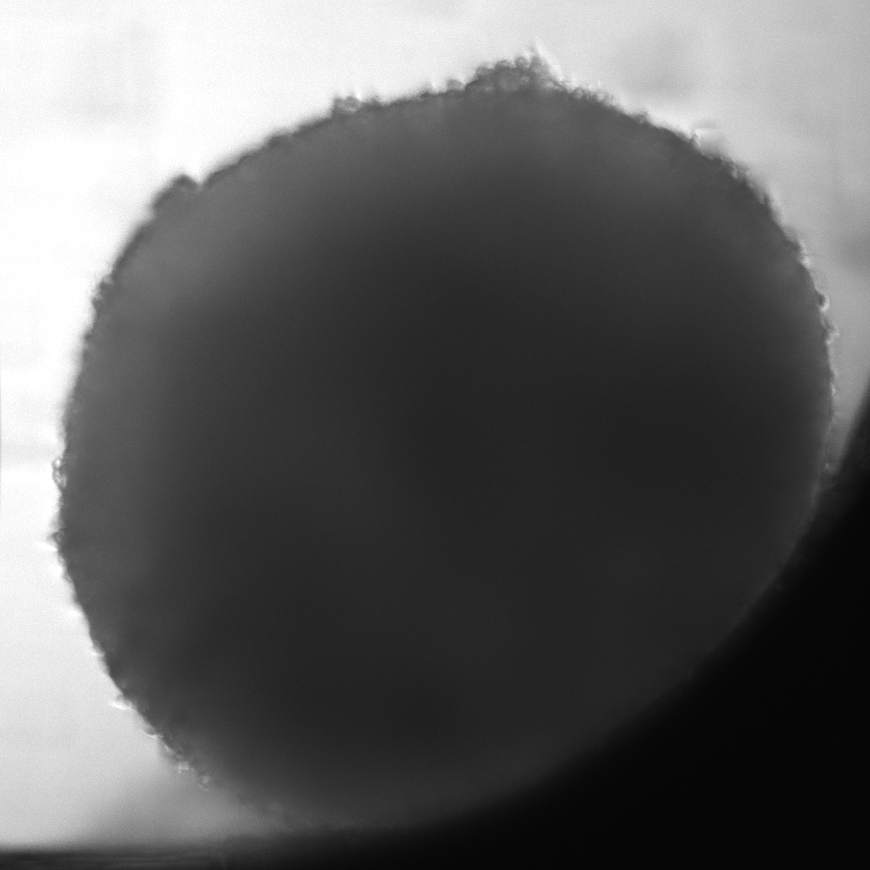

Supplement: Supplementary file 6 — Source Data for Figure 1 [file EMMM-15-e18199-s012.zip › Figure_1A,D,E/1D/Tumor_#7_D28.tif]

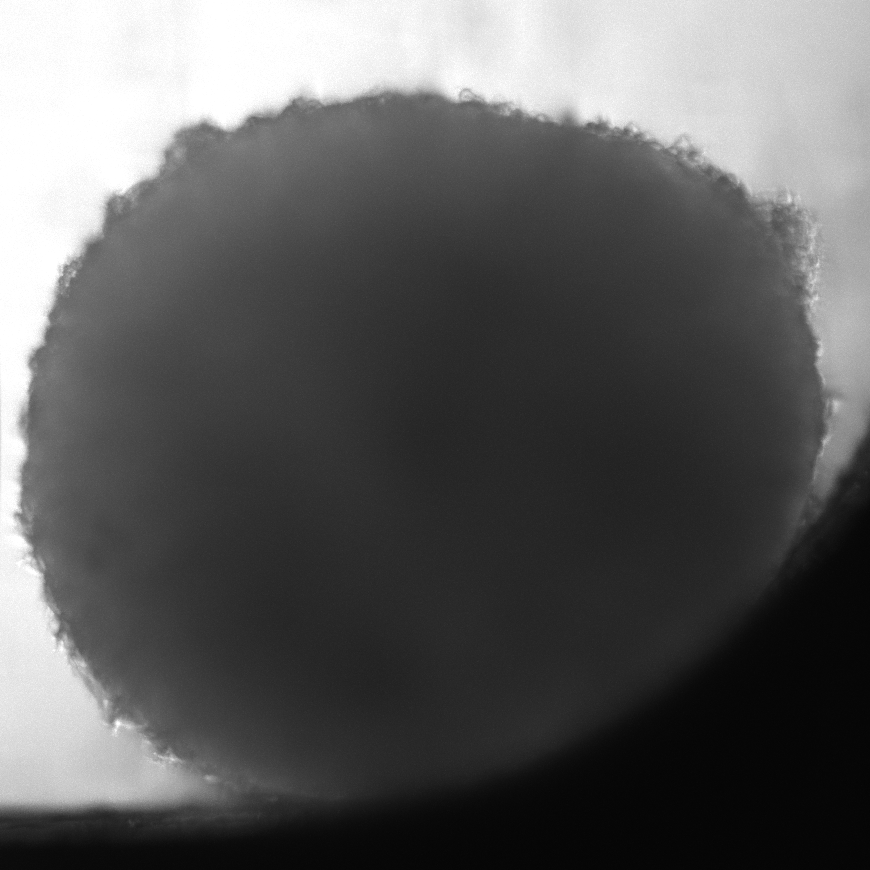

Supplement: Supplementary file 6 — Source Data for Figure 1 [file EMMM-15-e18199-s012.zip › Figure_1A,D,E/1D/Tumor_#7_D35.tif]

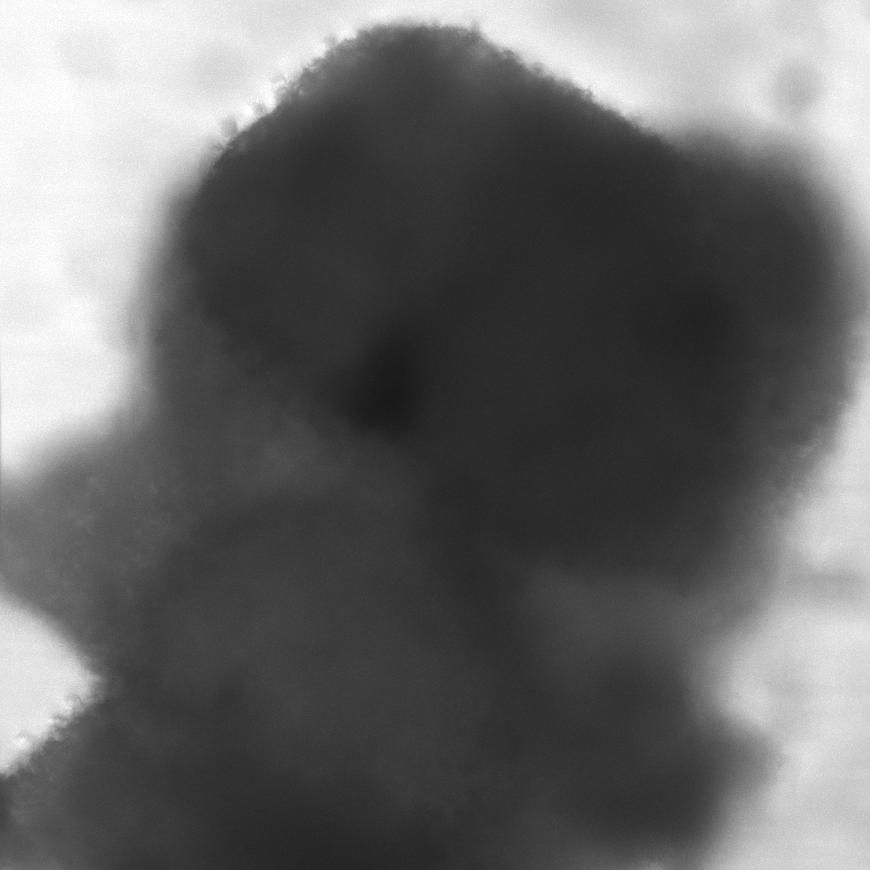

Supplement: Supplementary file 6 — Source Data for Figure 1 [file EMMM-15-e18199-s012.zip › Figure_1A,D,E/1D/Tumor_#7_D7.tif]

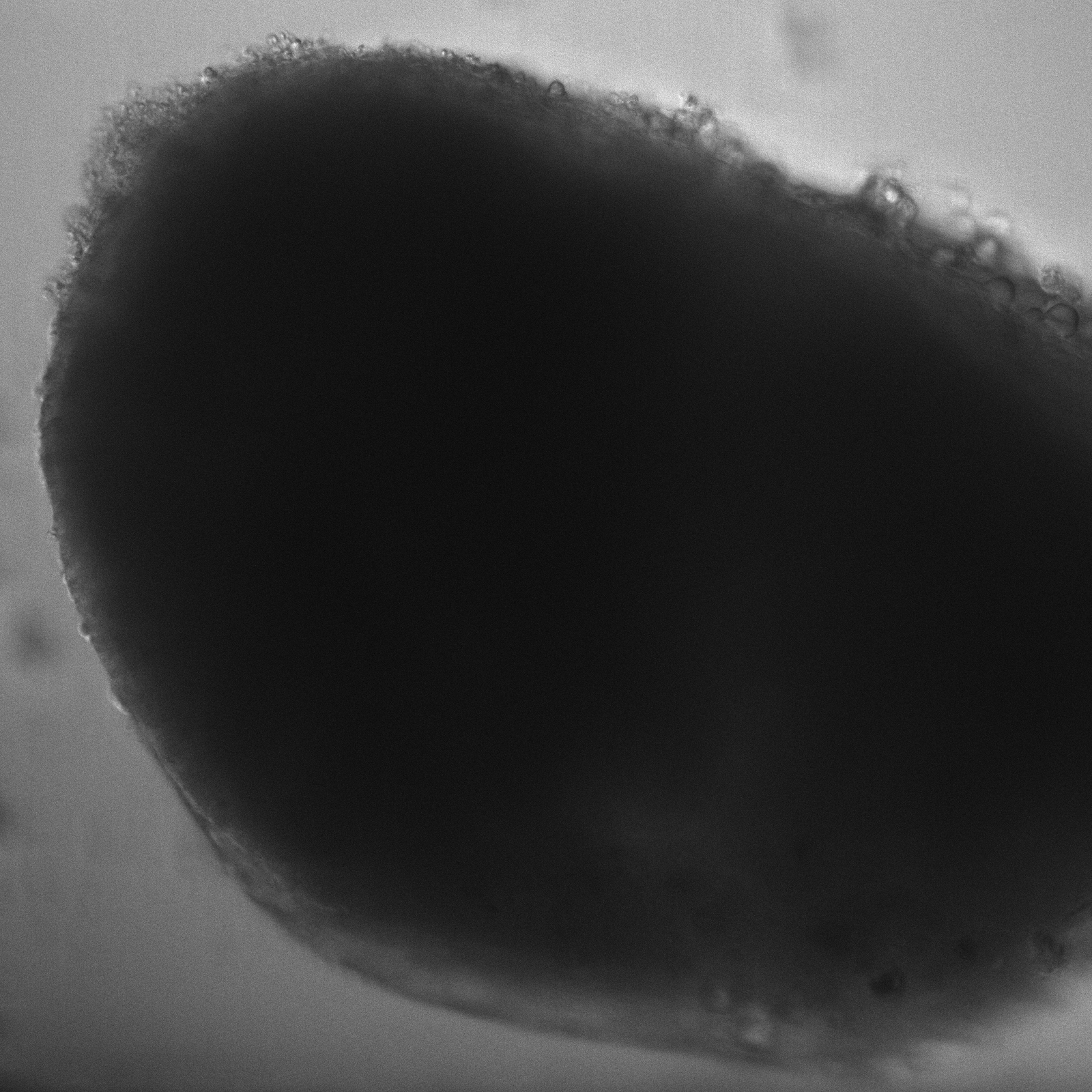

Supplement: Supplementary file 6 — Source Data for Figure 1 [file EMMM-15-e18199-s012.zip › Figure_1A,D,E/1E/Tumor_#10_D14.tif]

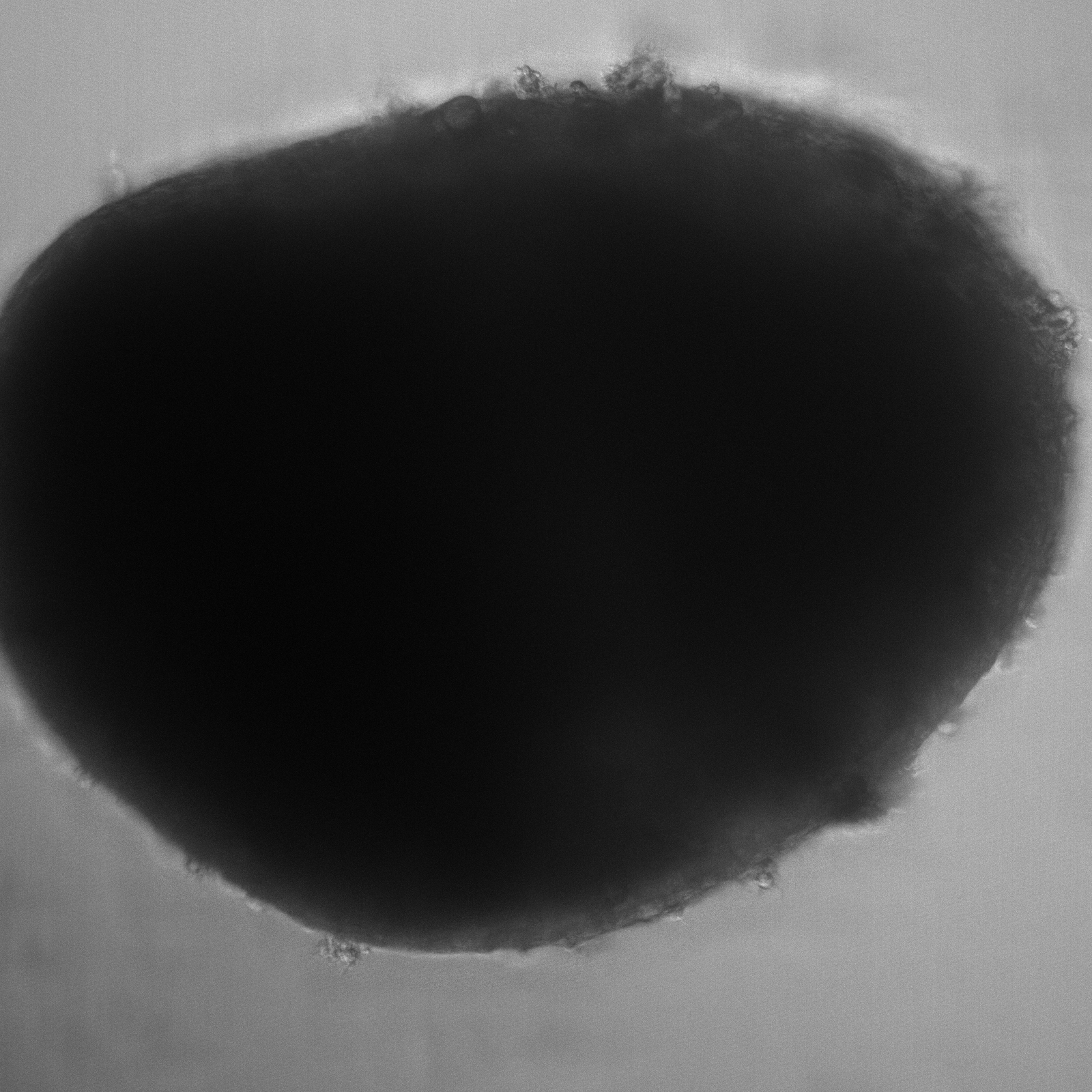

Supplement: Supplementary file 6 — Source Data for Figure 1 [file EMMM-15-e18199-s012.zip › Figure_1A,D,E/1E/Tumor_#10_D21.tif]

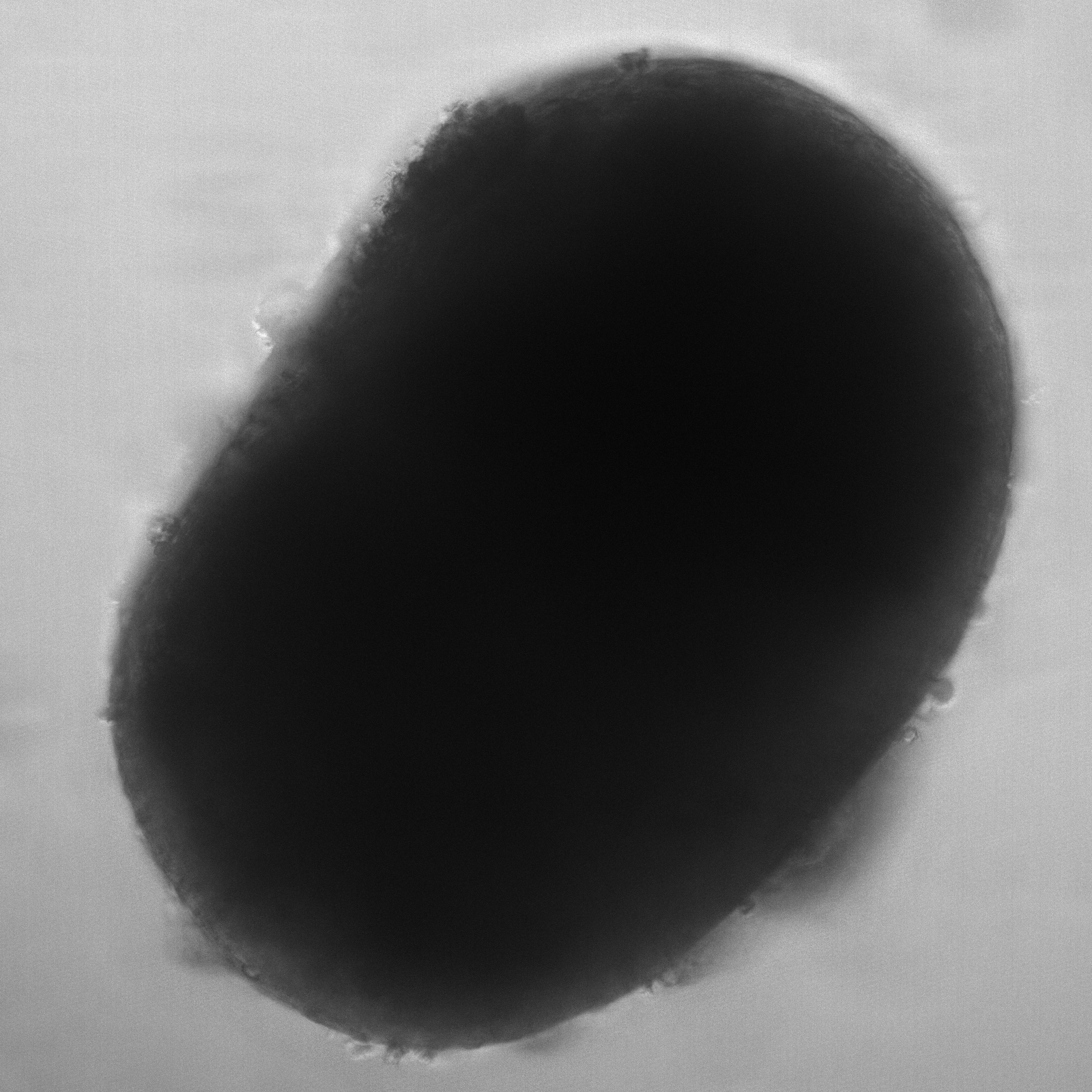

Supplement: Supplementary file 6 — Source Data for Figure 1 [file EMMM-15-e18199-s012.zip › Figure_1A,D,E/1E/Tumor_#10_D28.tif]

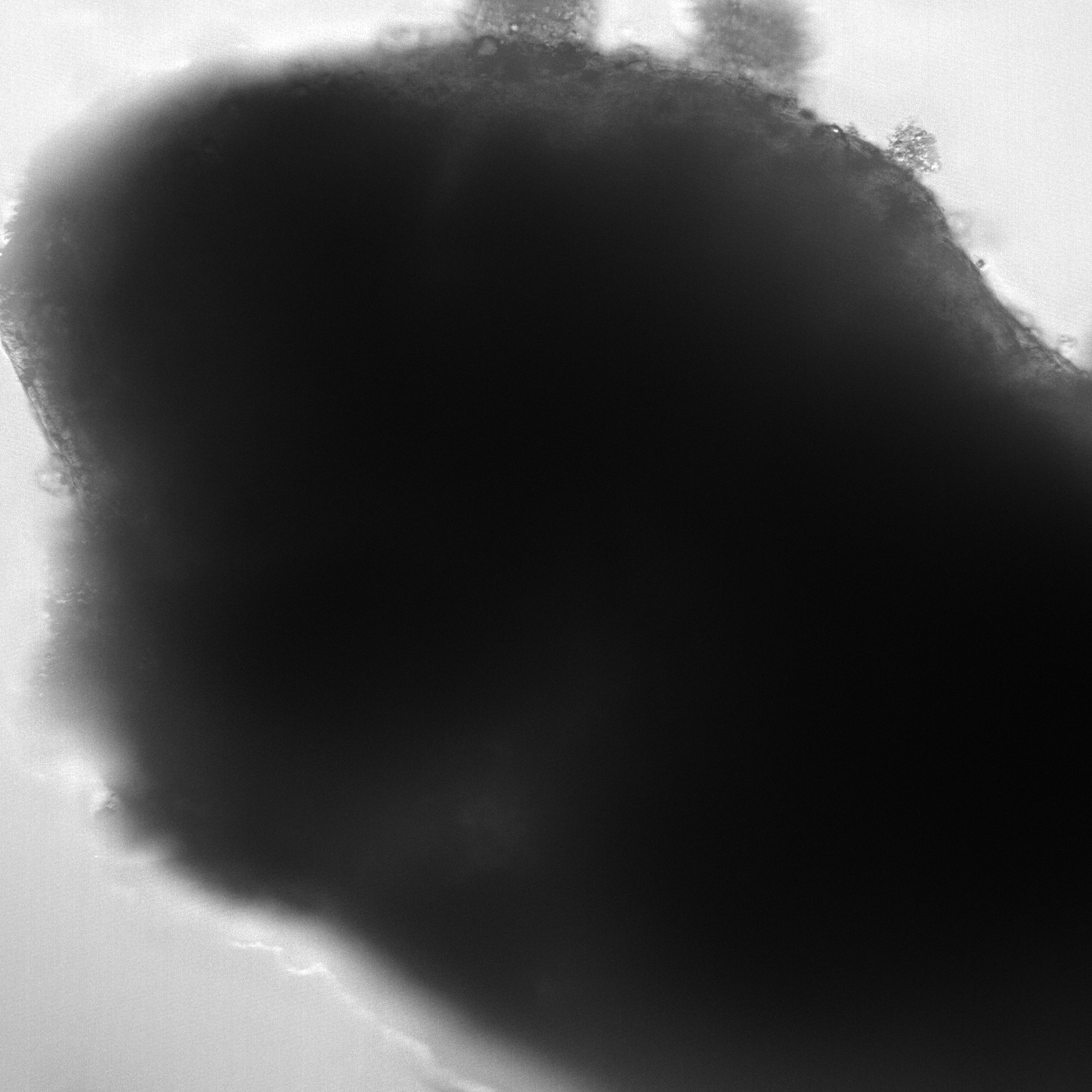

Supplement: Supplementary file 6 — Source Data for Figure 1 [file EMMM-15-e18199-s012.zip › Figure_1A,D,E/1E/Tumor_#10_D7.tif]

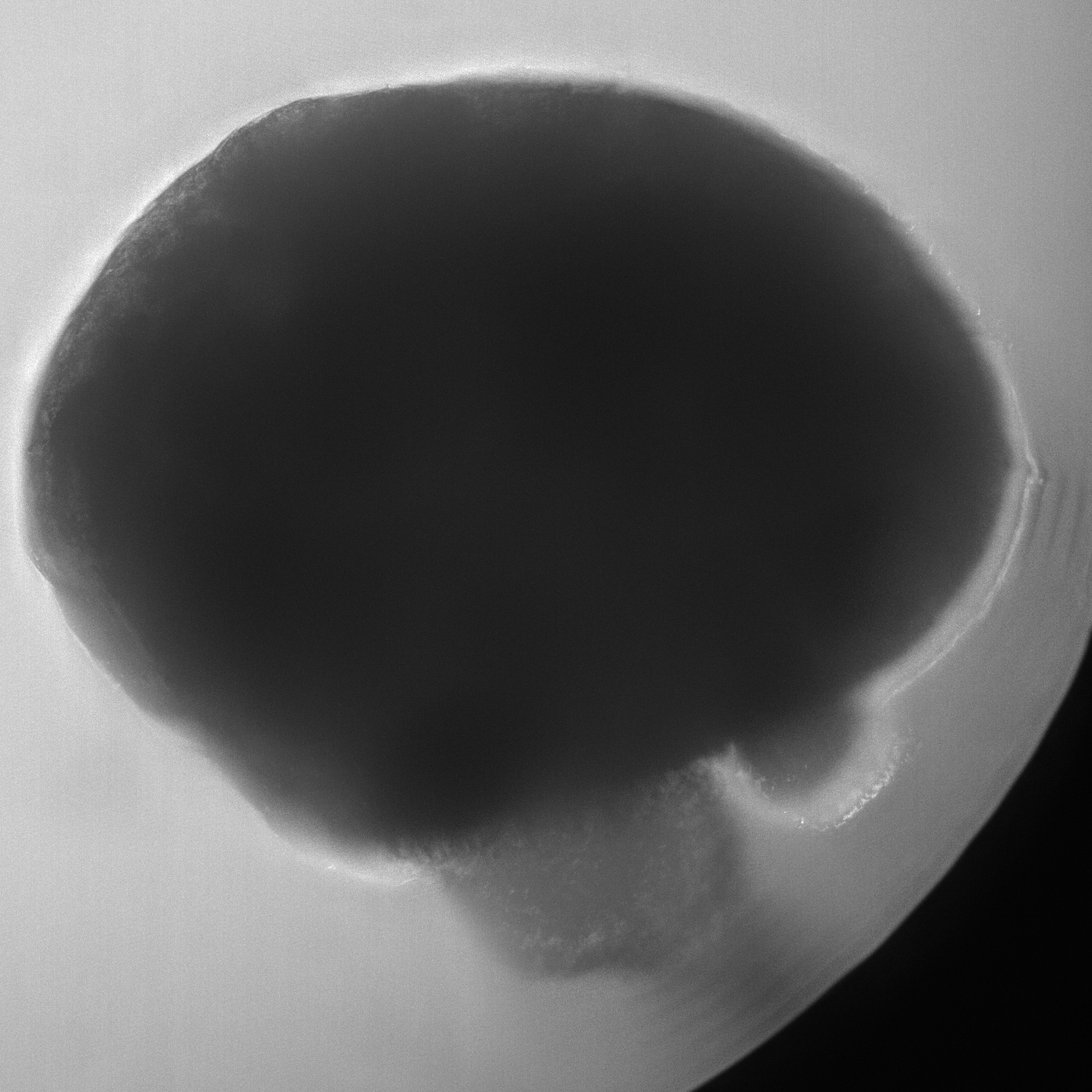

Supplement: Supplementary file 6 — Source Data for Figure 1 [file EMMM-15-e18199-s012.zip › Figure_1A,D,E/1E/Tumor_#11_D14.tif]

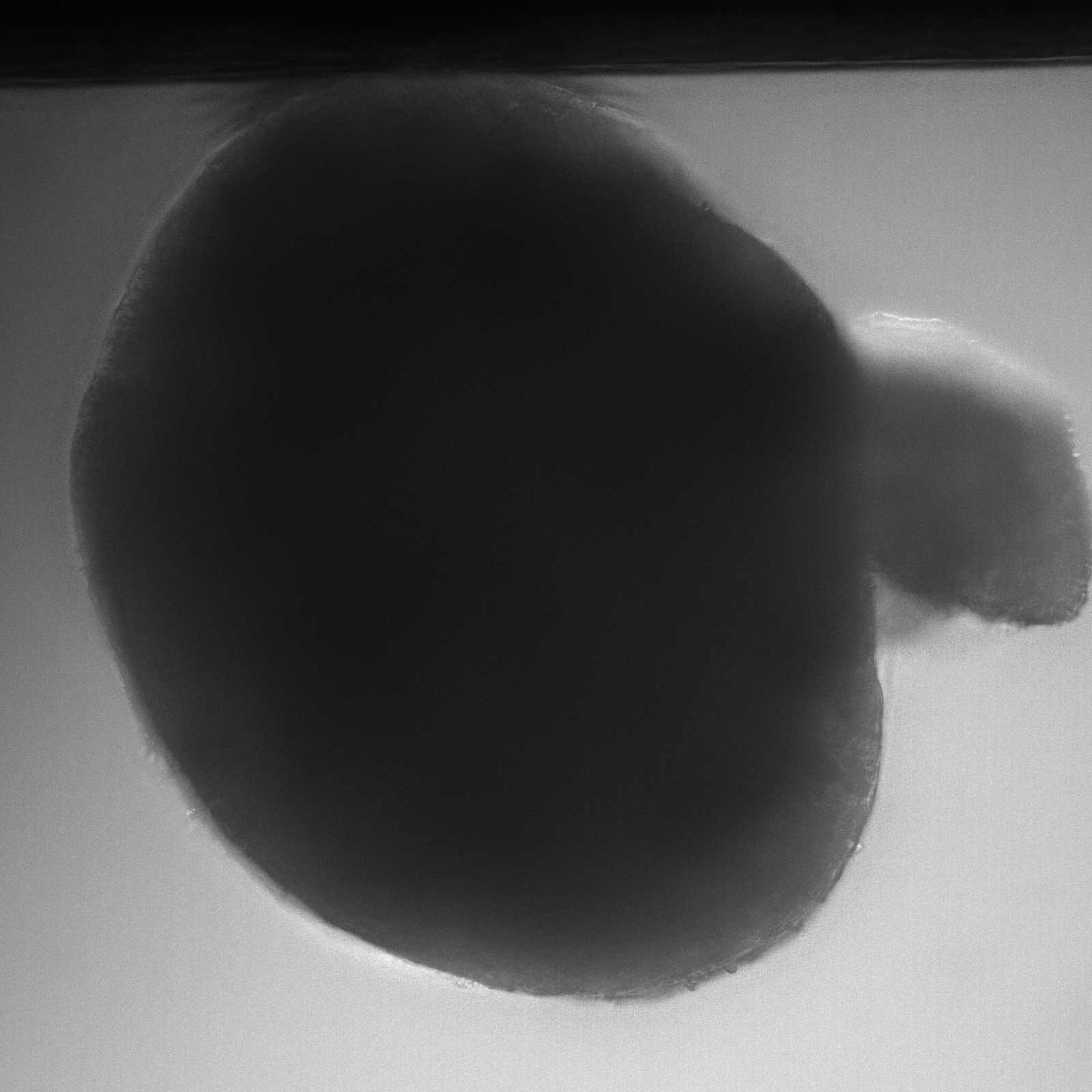

Supplement: Supplementary file 6 — Source Data for Figure 1 [file EMMM-15-e18199-s012.zip › Figure_1A,D,E/1E/Tumor_#11_D21.tif]

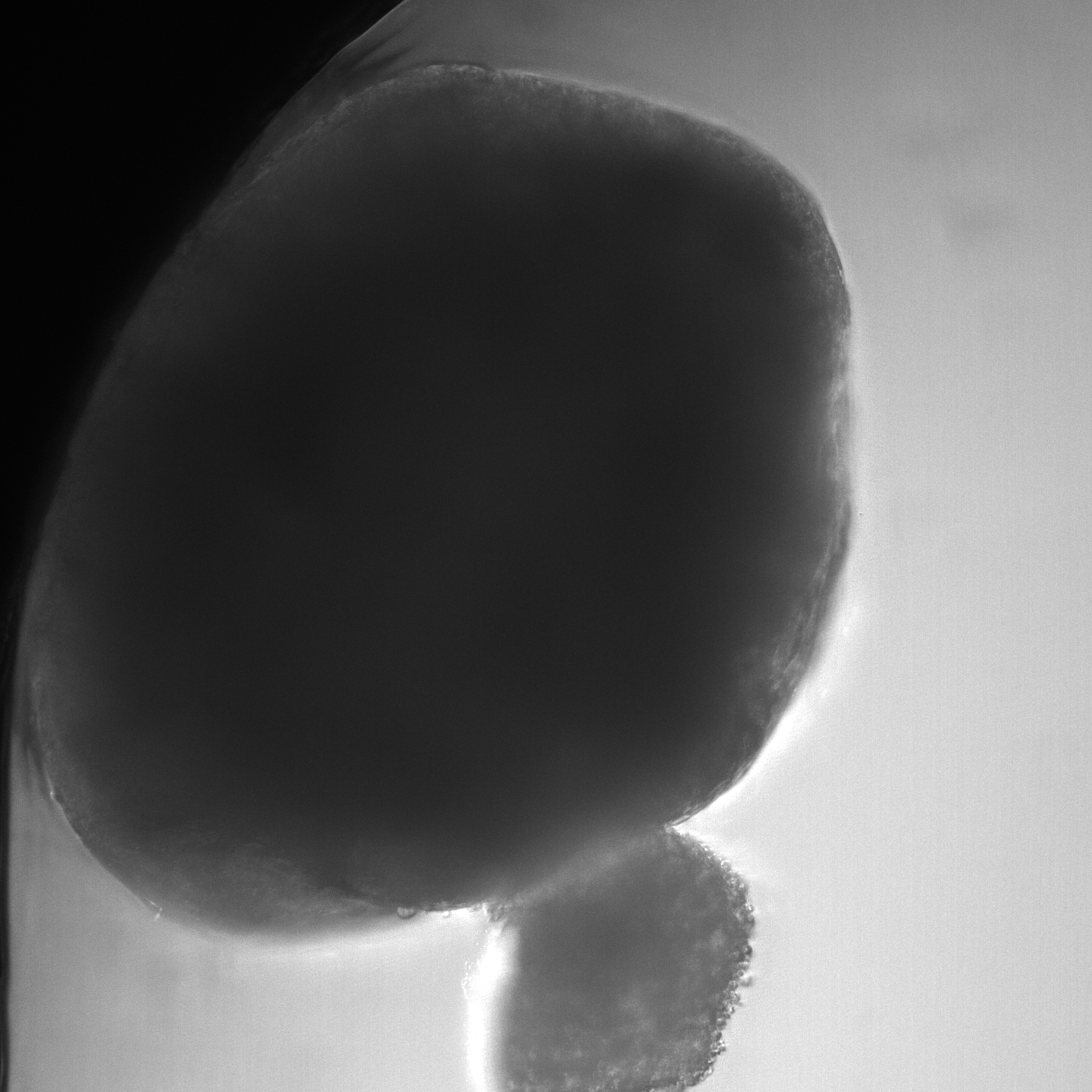

Supplement: Supplementary file 6 — Source Data for Figure 1 [file EMMM-15-e18199-s012.zip › Figure_1A,D,E/1E/Tumor_#11_D28.tif]

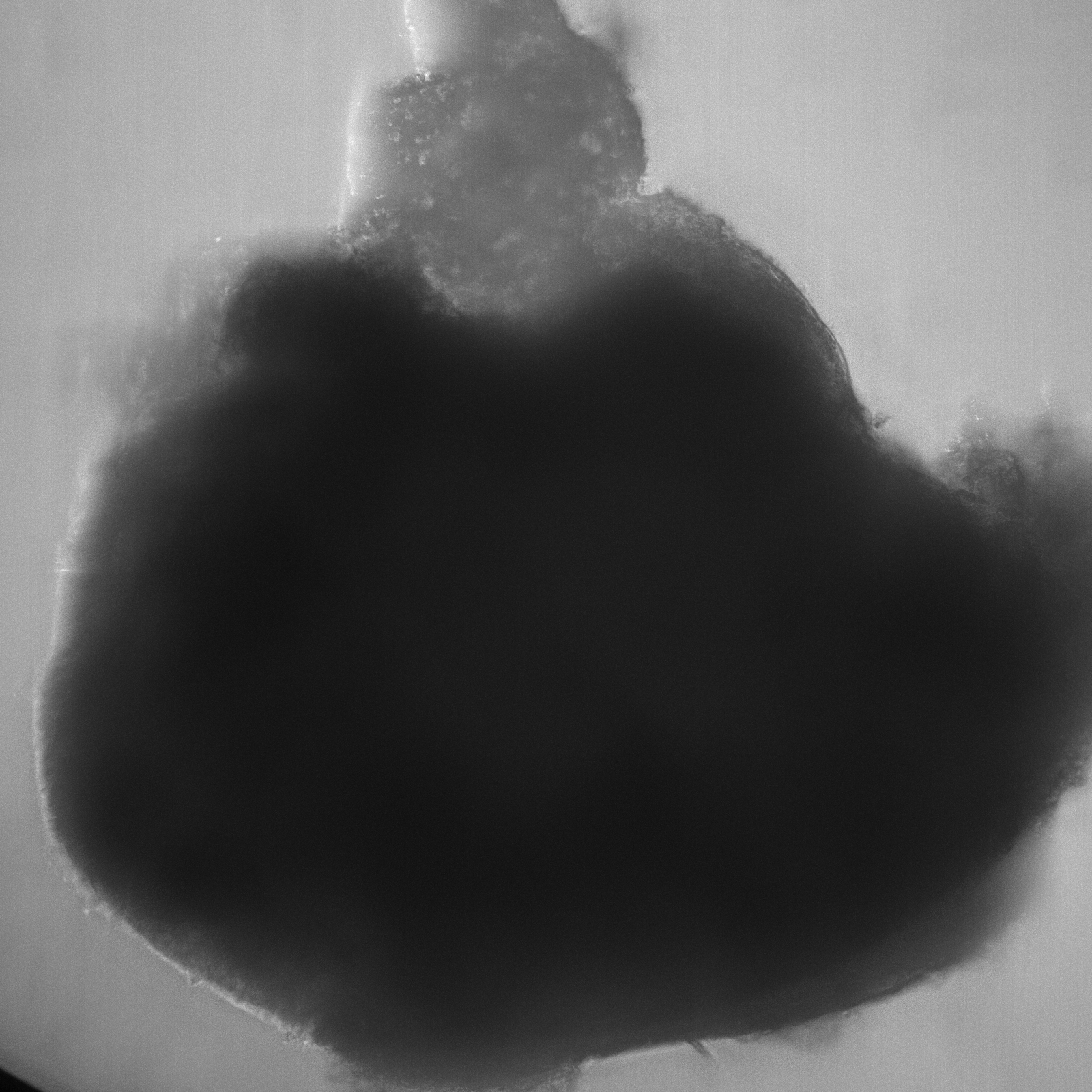

Supplement: Supplementary file 6 — Source Data for Figure 1 [file EMMM-15-e18199-s012.zip › Figure_1A,D,E/1E/Tumor_#11_D7.tif]

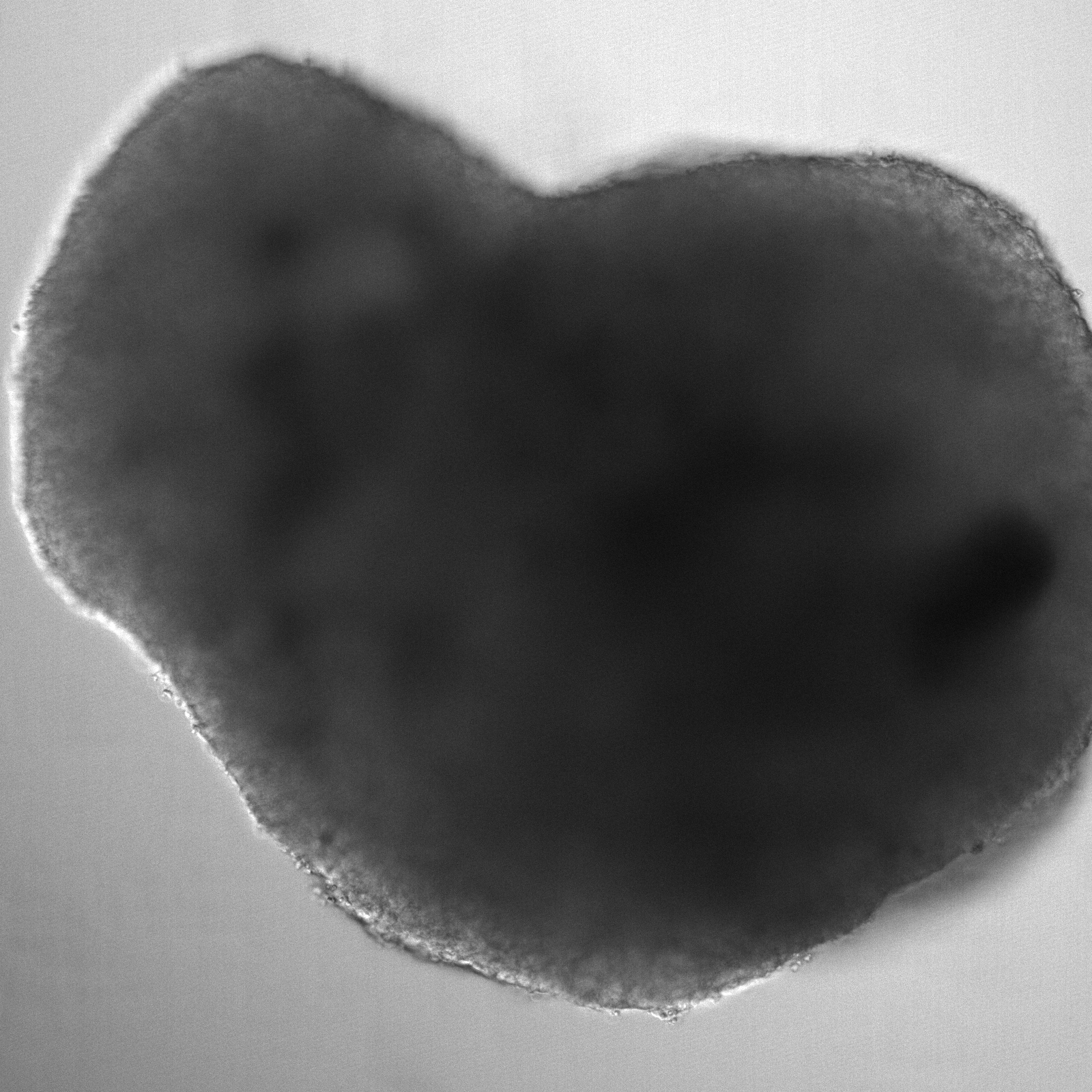

Supplement: Supplementary file 6 — Source Data for Figure 1 [file EMMM-15-e18199-s012.zip › Figure_1A,D,E/1E/Tumor_#12_D14.tif]

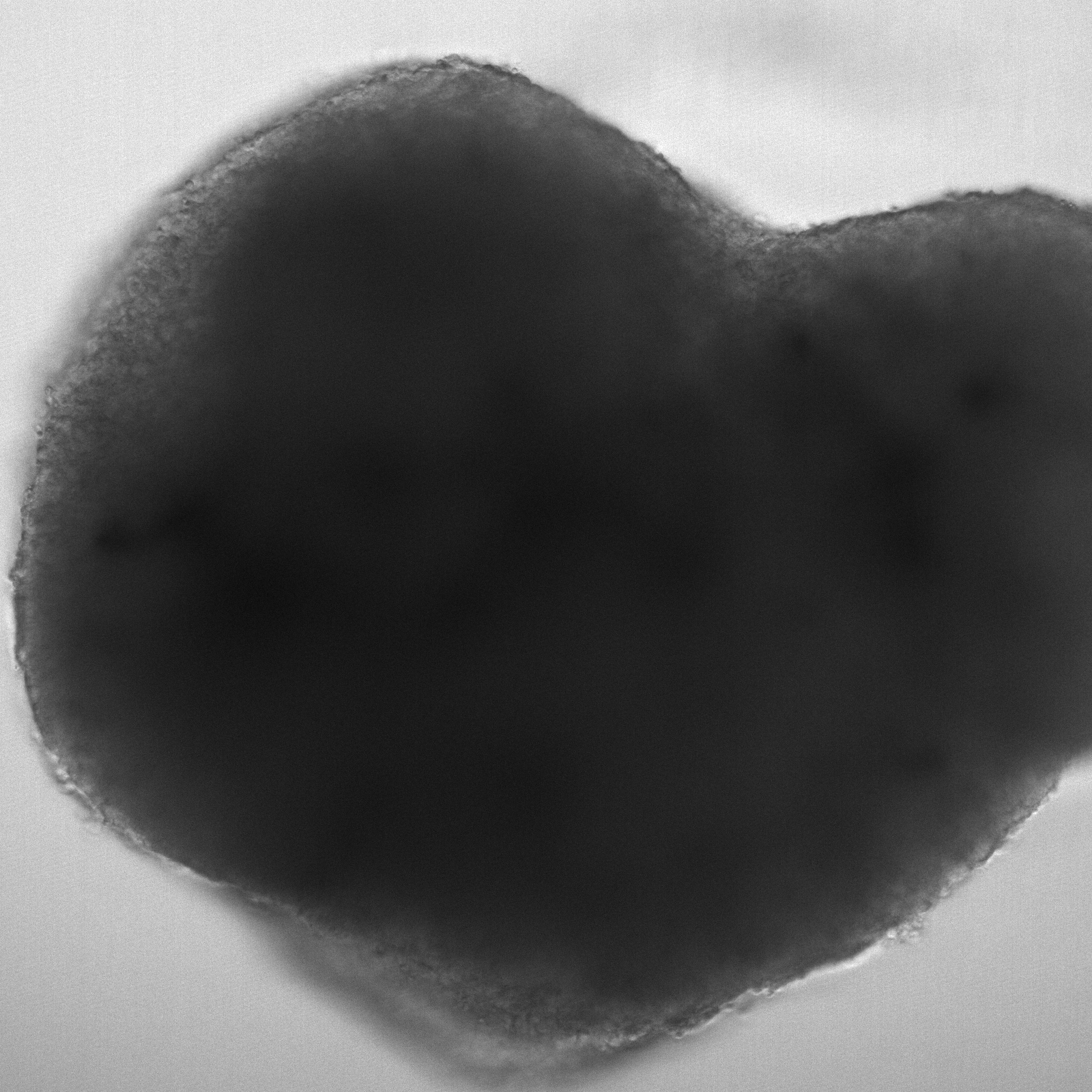

Supplement: Supplementary file 6 — Source Data for Figure 1 [file EMMM-15-e18199-s012.zip › Figure_1A,D,E/1E/Tumor_#12_D21.tif]

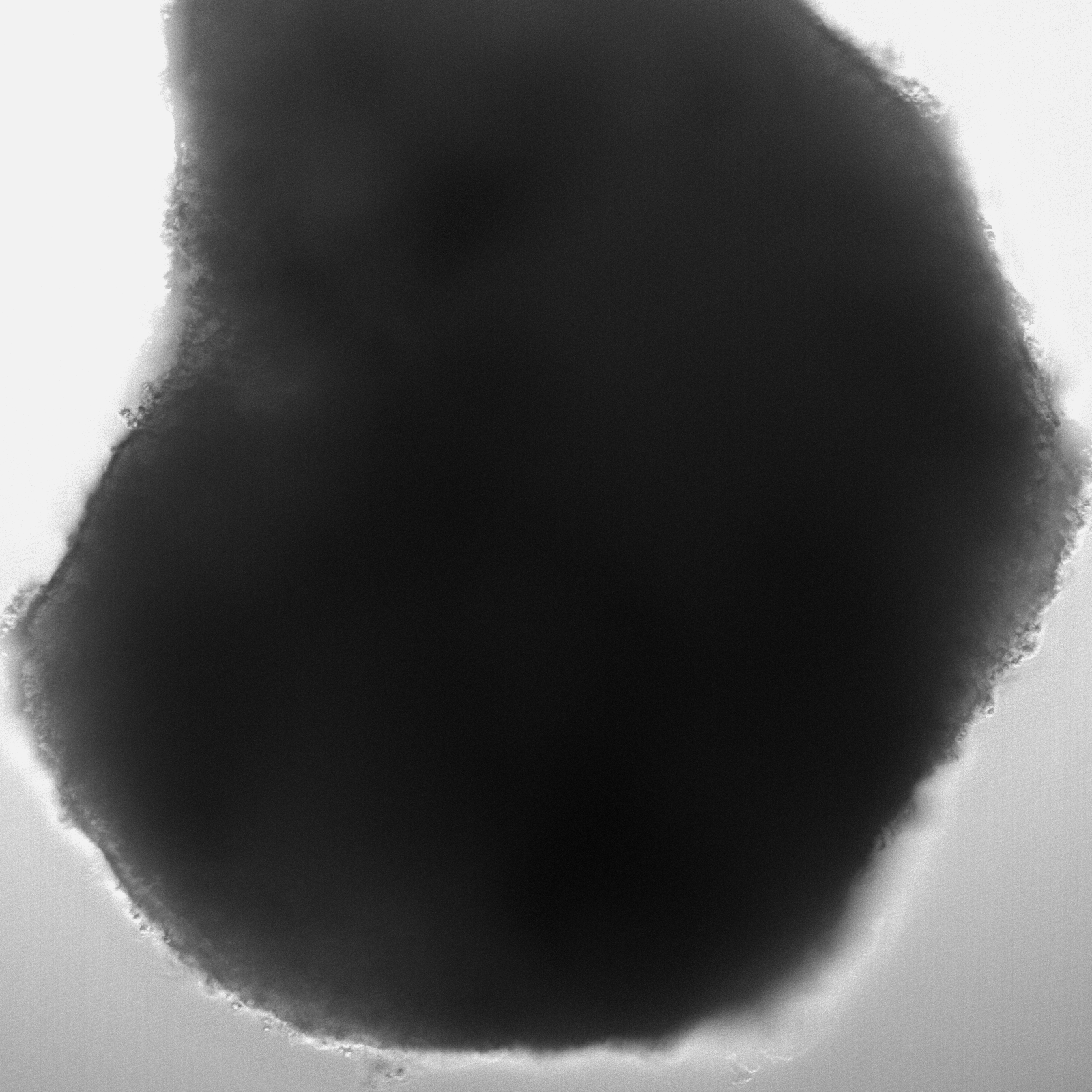

Supplement: Supplementary file 6 — Source Data for Figure 1 [file EMMM-15-e18199-s012.zip › Figure_1A,D,E/1E/Tumor_#12_D28.tif]

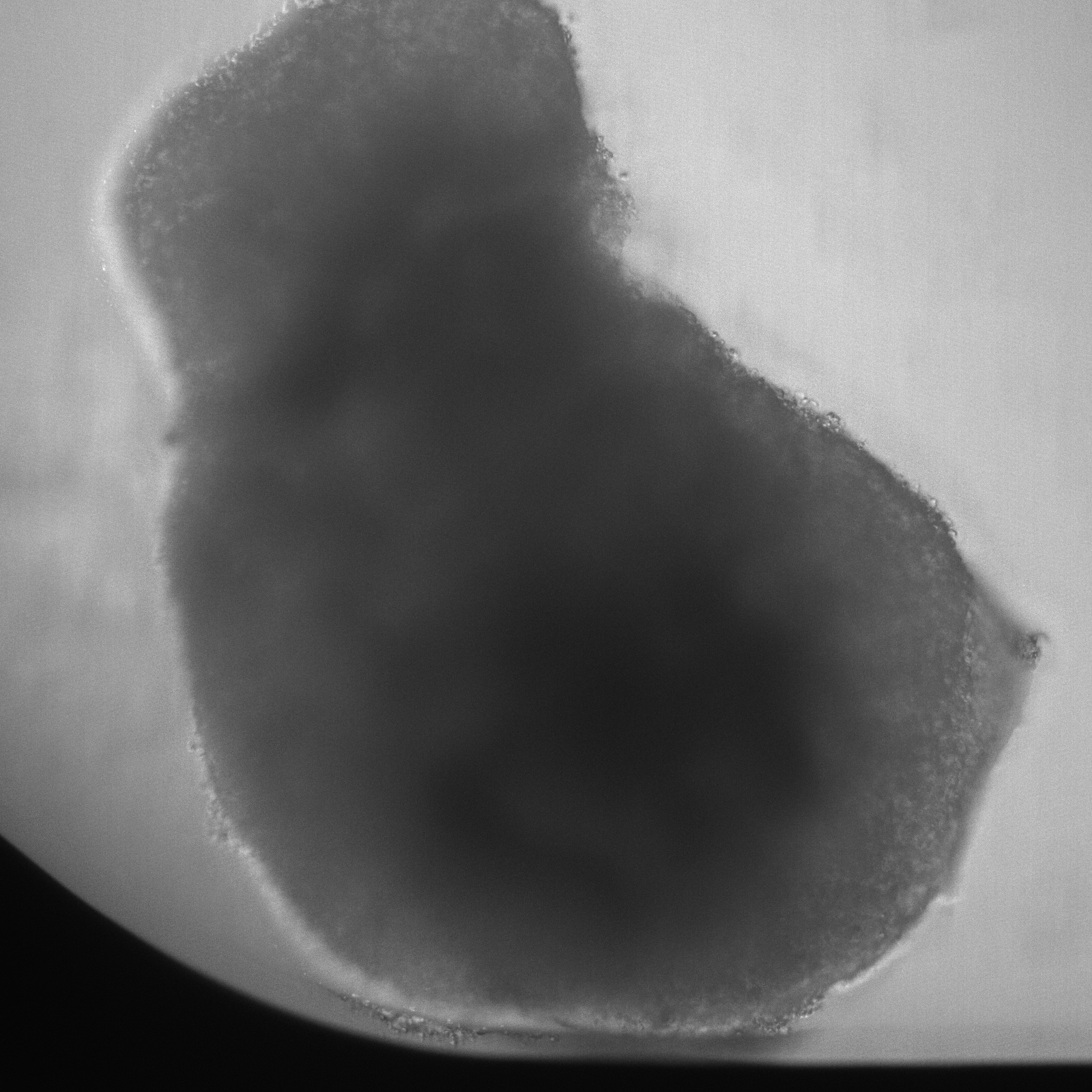

Supplement: Supplementary file 6 — Source Data for Figure 1 [file EMMM-15-e18199-s012.zip › Figure_1A,D,E/1E/Tumor_#12_D7.tif]

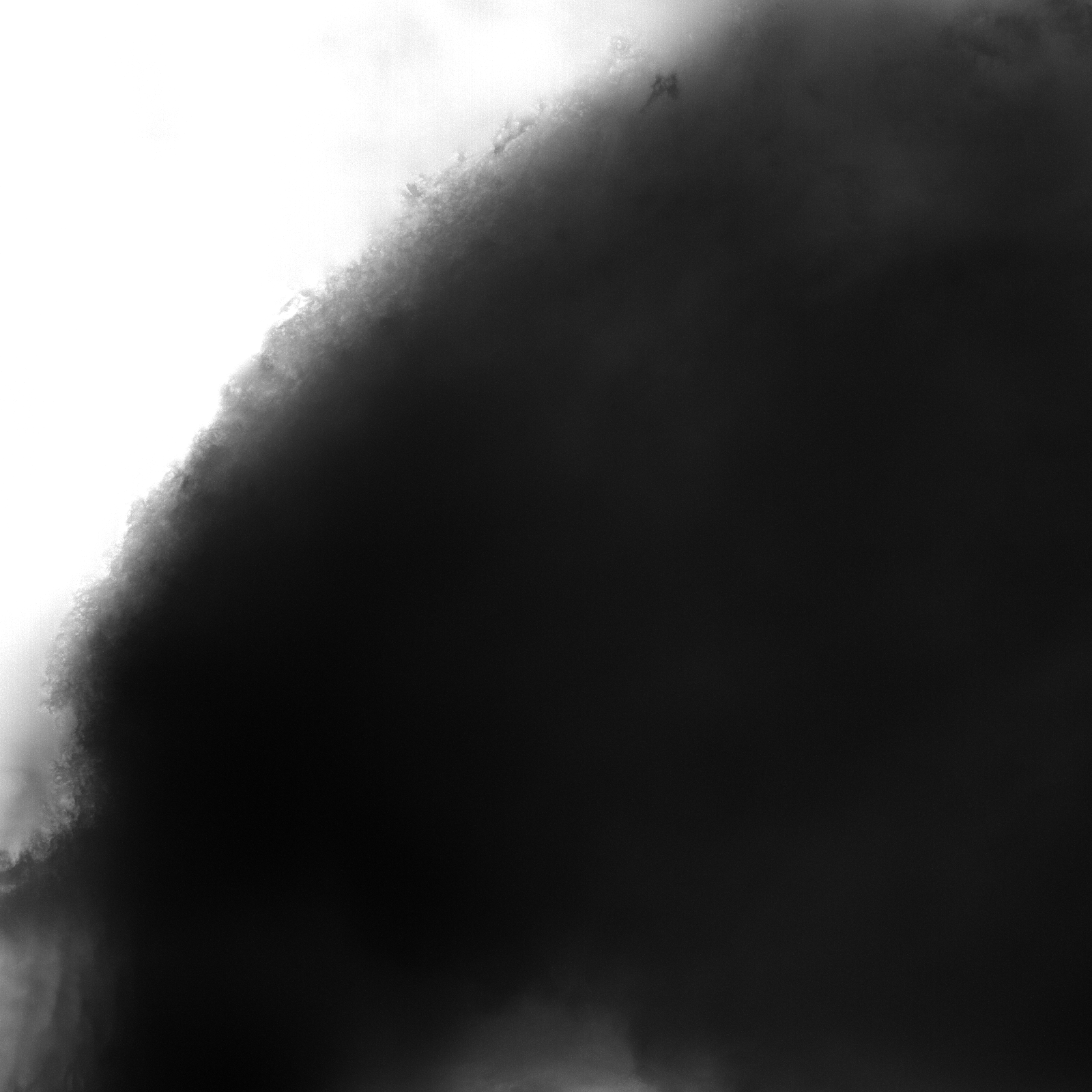

Supplement: Supplementary file 6 — Source Data for Figure 1 [file EMMM-15-e18199-s012.zip › Figure_1A,D,E/1E/Tumor_#13_D14.tif]

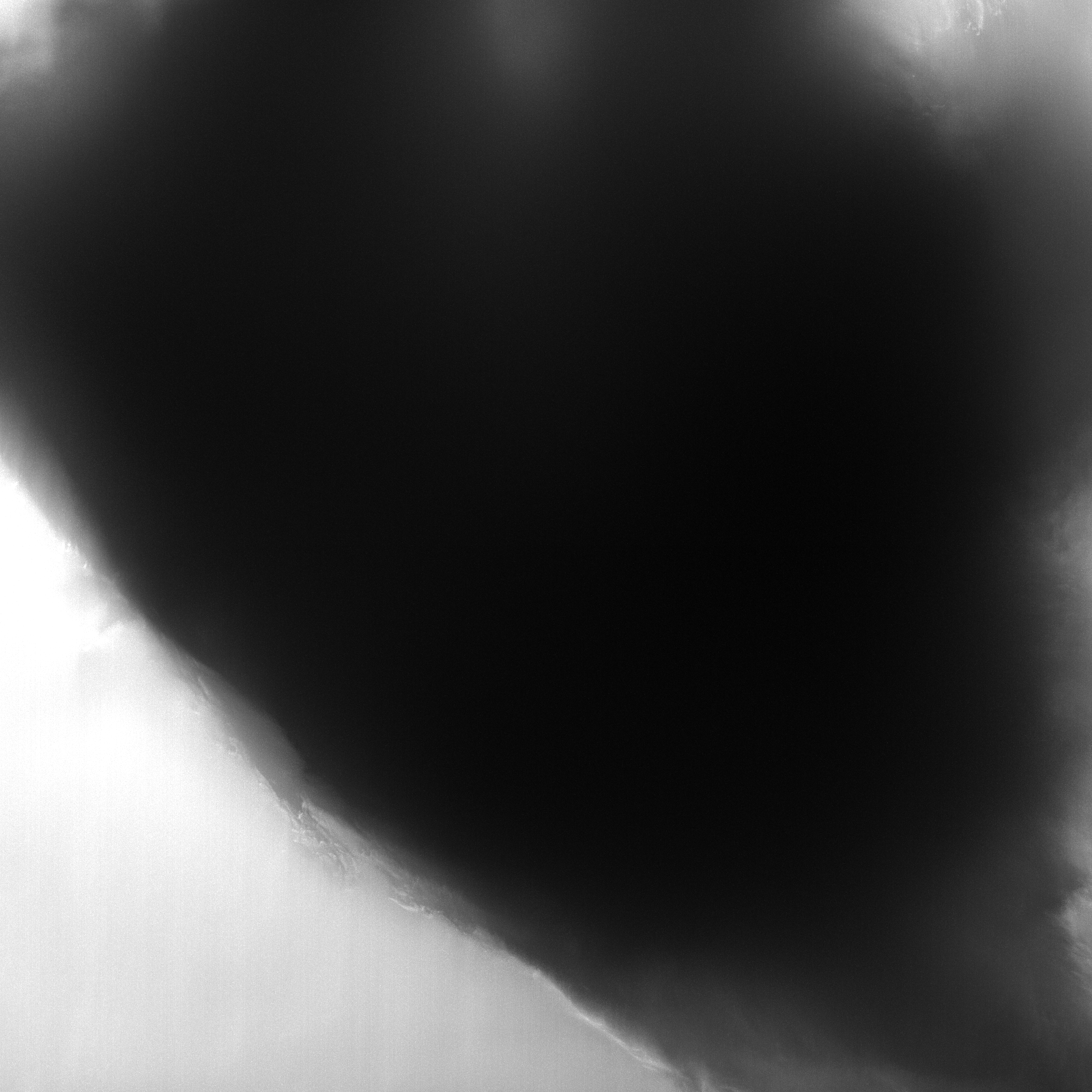

Supplement: Supplementary file 6 — Source Data for Figure 1 [file EMMM-15-e18199-s012.zip › Figure_1A,D,E/1E/Tumor_#13_D21.tif]

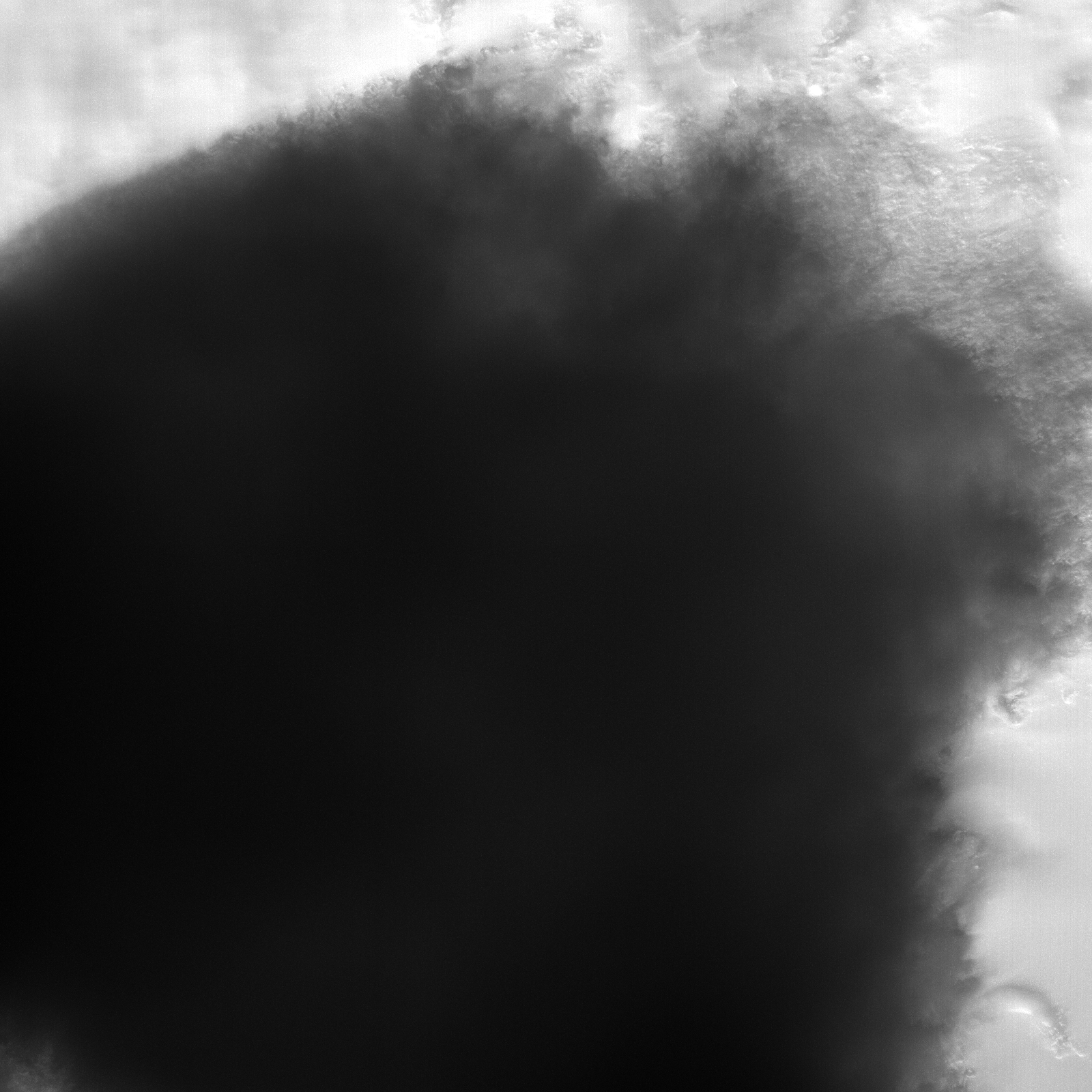

Supplement: Supplementary file 6 — Source Data for Figure 1 [file EMMM-15-e18199-s012.zip › Figure_1A,D,E/1E/Tumor_#13_D28.tif]

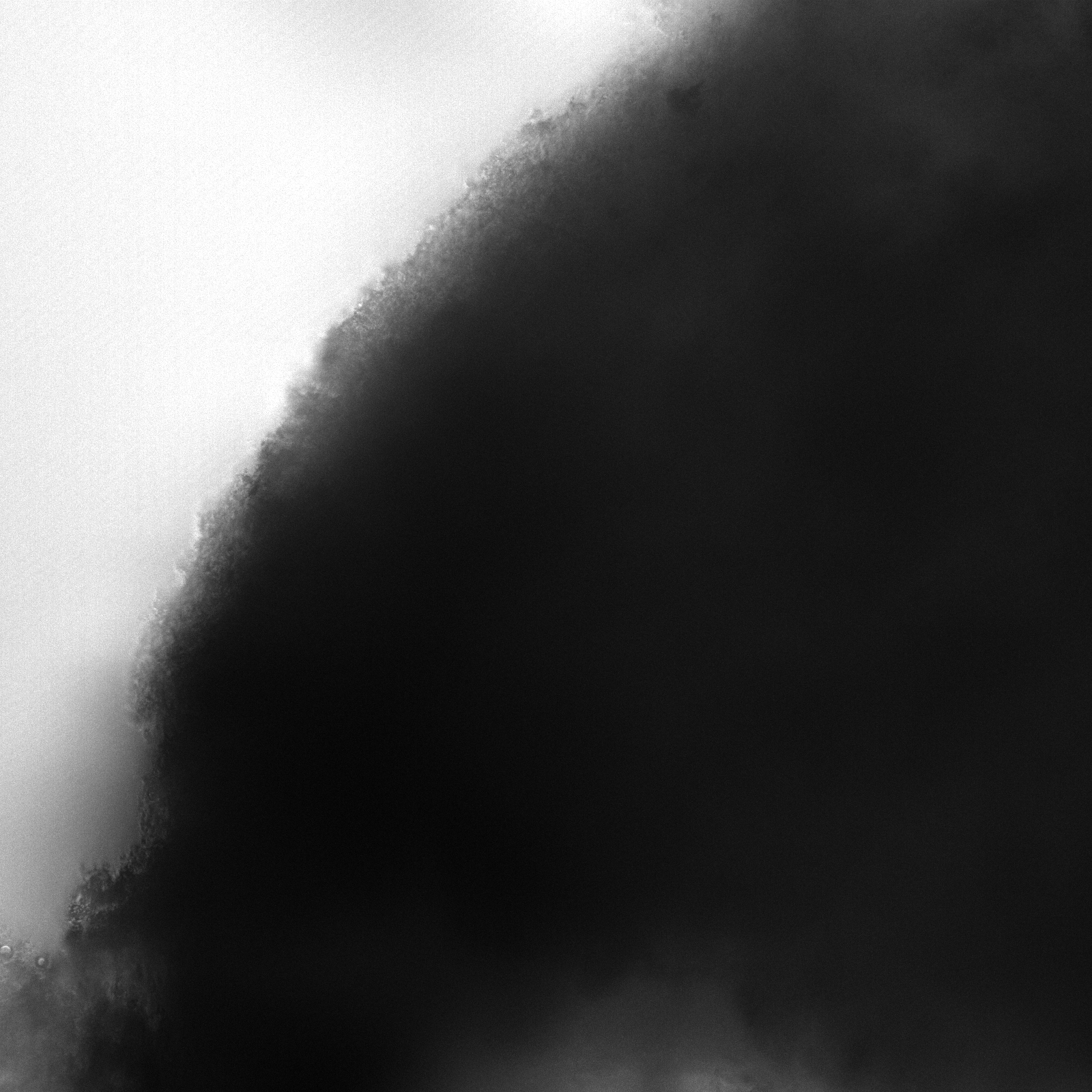

Supplement: Supplementary file 6 — Source Data for Figure 1 [file EMMM-15-e18199-s012.zip › Figure_1A,D,E/1E/Tumor_#13_D7.tif]

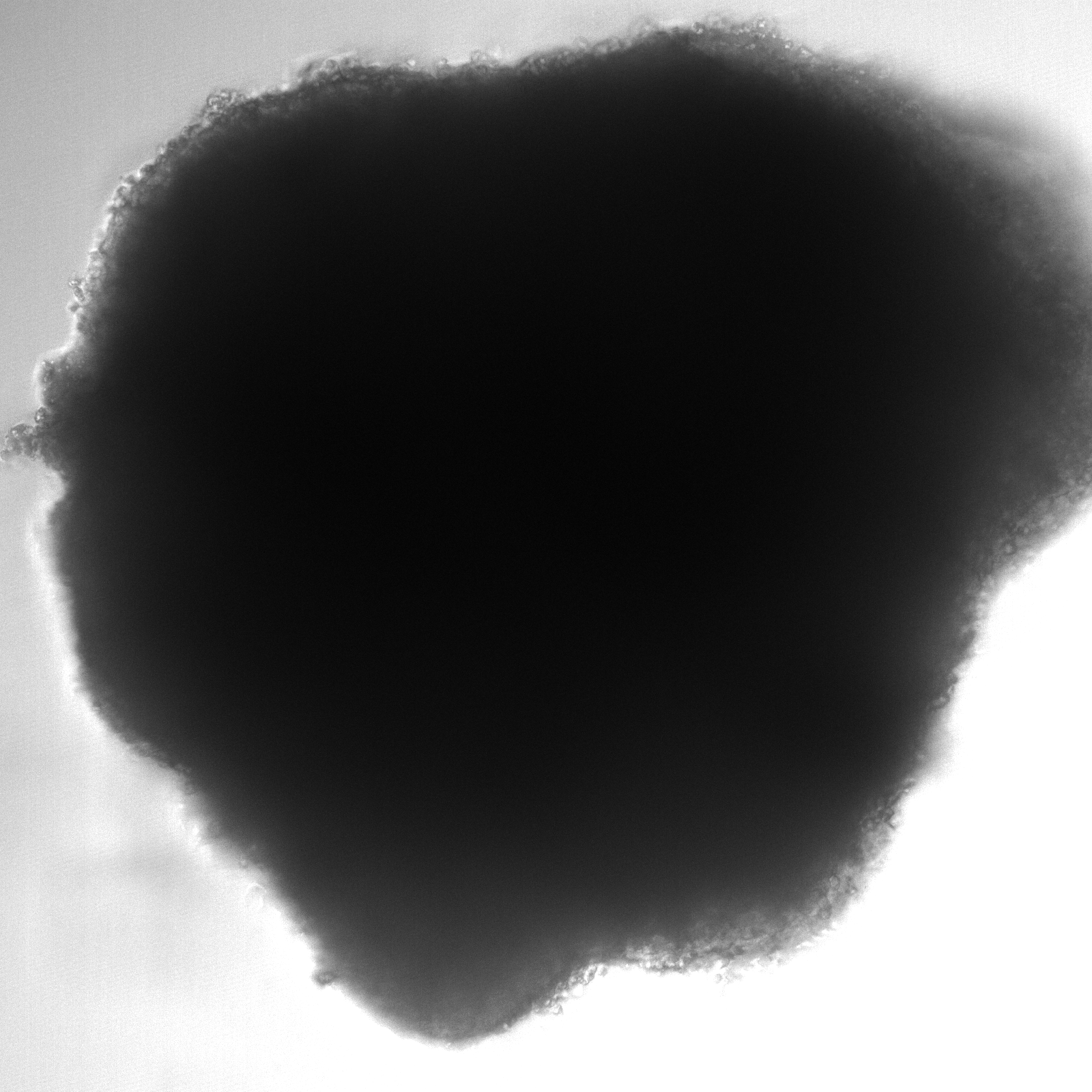

Supplement: Supplementary file 6 — Source Data for Figure 1 [file EMMM-15-e18199-s012.zip › Figure_1A,D,E/1E/Tumor_#14_D14.tif]

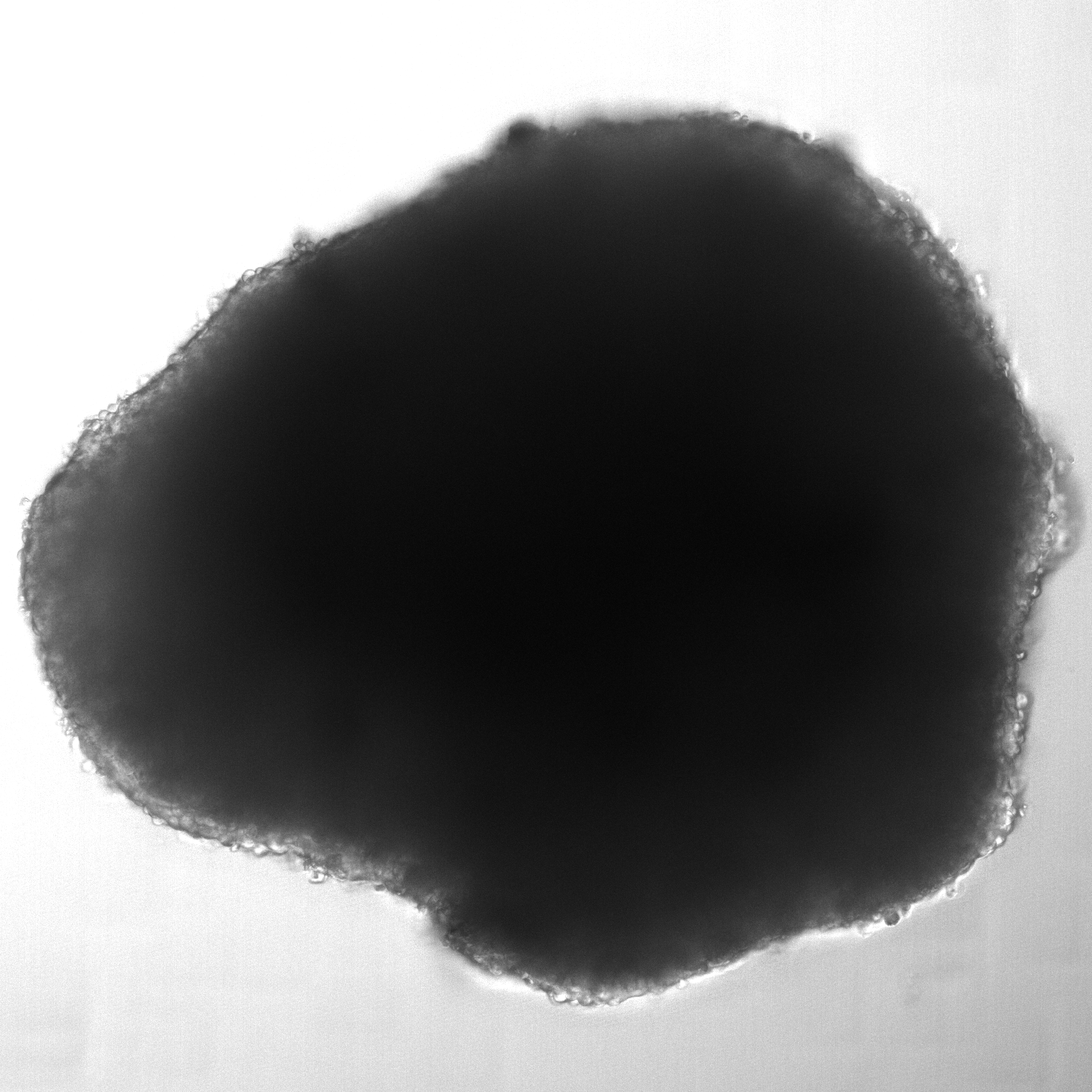

Supplement: Supplementary file 6 — Source Data for Figure 1 [file EMMM-15-e18199-s012.zip › Figure_1A,D,E/1E/Tumor_#14_D21.tif]

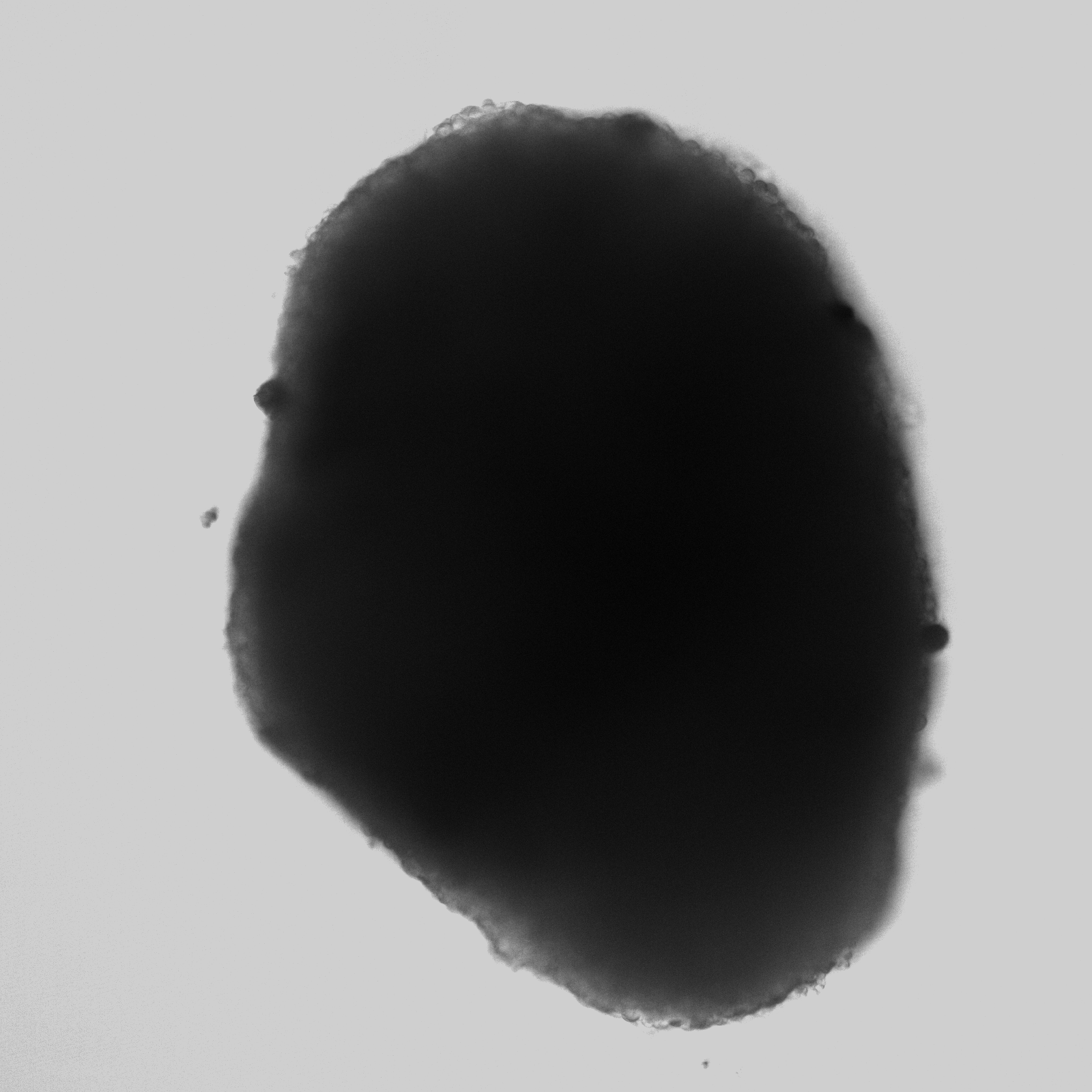

Supplement: Supplementary file 6 — Source Data for Figure 1 [file EMMM-15-e18199-s012.zip › Figure_1A,D,E/1E/Tumor_#14_D28.tif]

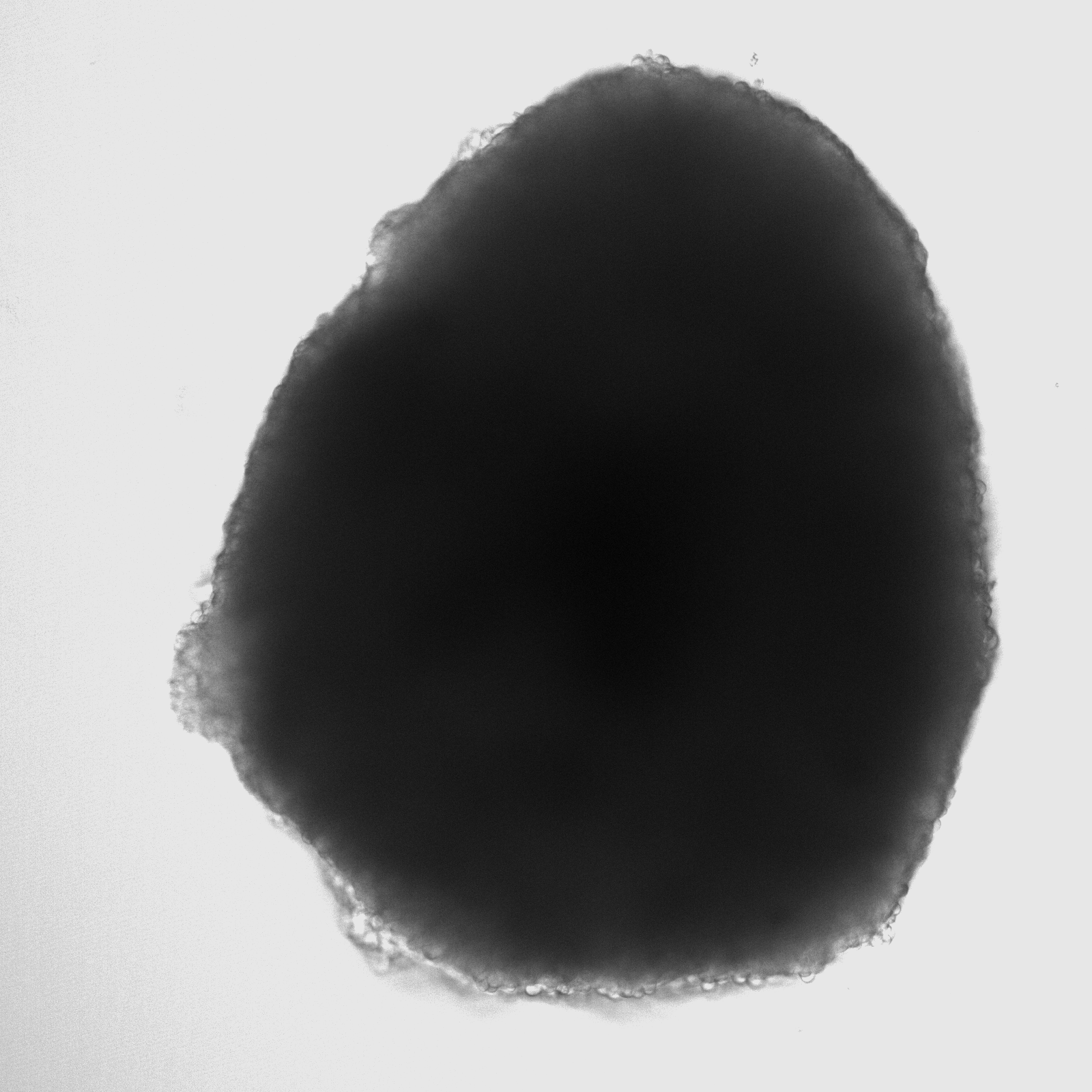

Supplement: Supplementary file 6 — Source Data for Figure 1 [file EMMM-15-e18199-s012.zip › Figure_1A,D,E/1E/Tumor_#14_D53.tif]

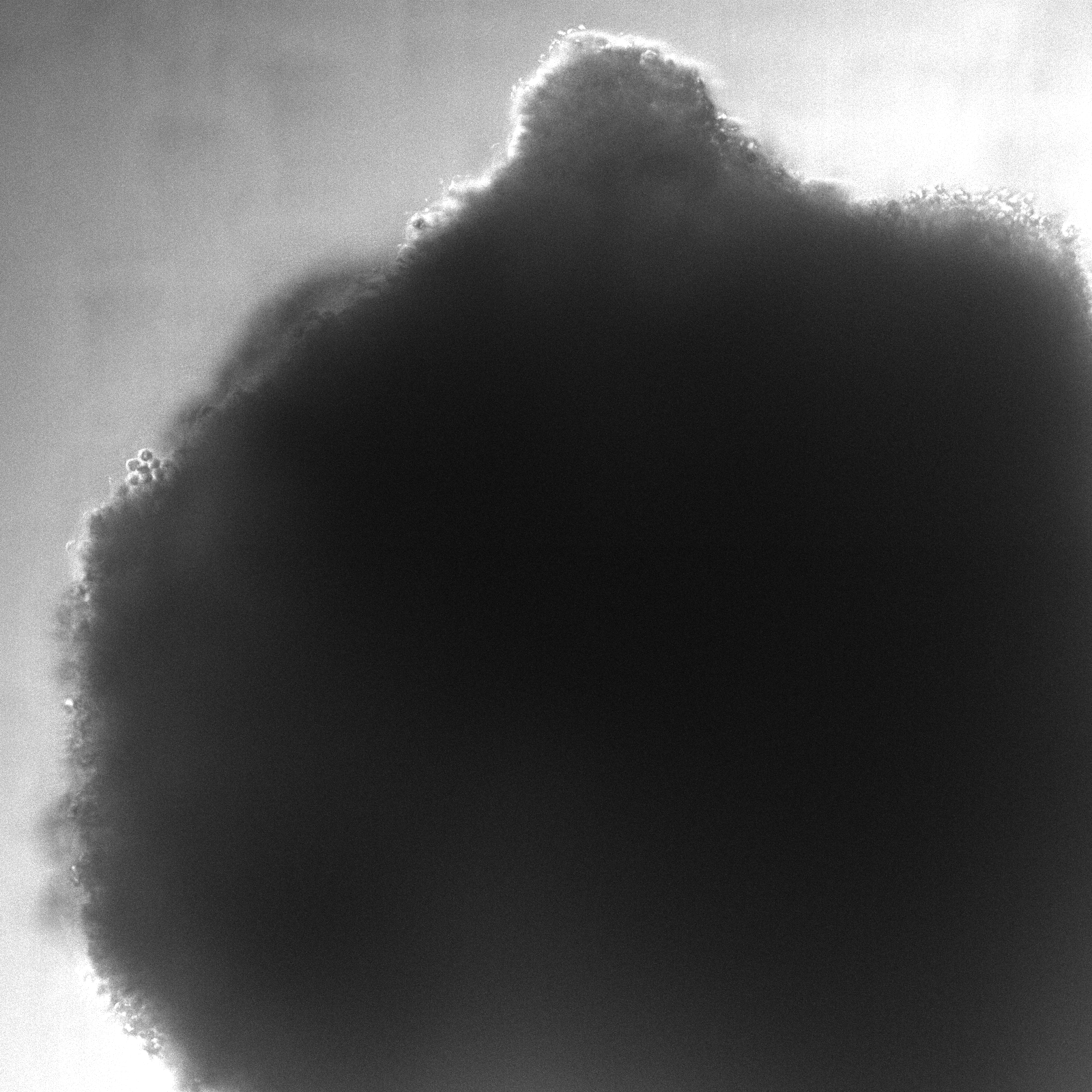

Supplement: Supplementary file 6 — Source Data for Figure 1 [file EMMM-15-e18199-s012.zip › Figure_1A,D,E/1E/Tumor_#14_D7.tif]

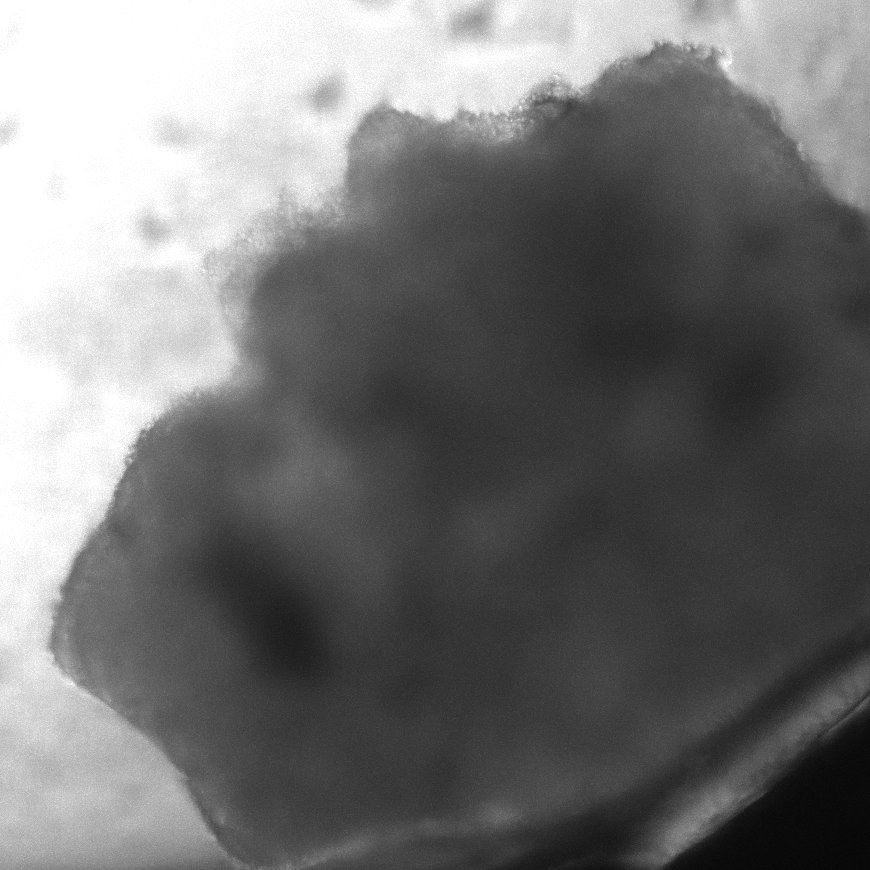

Supplement: Supplementary file 6 — Source Data for Figure 1 [file EMMM-15-e18199-s012.zip › Figure_1A,D,E/1E/Tumor_#15_D14.tif]

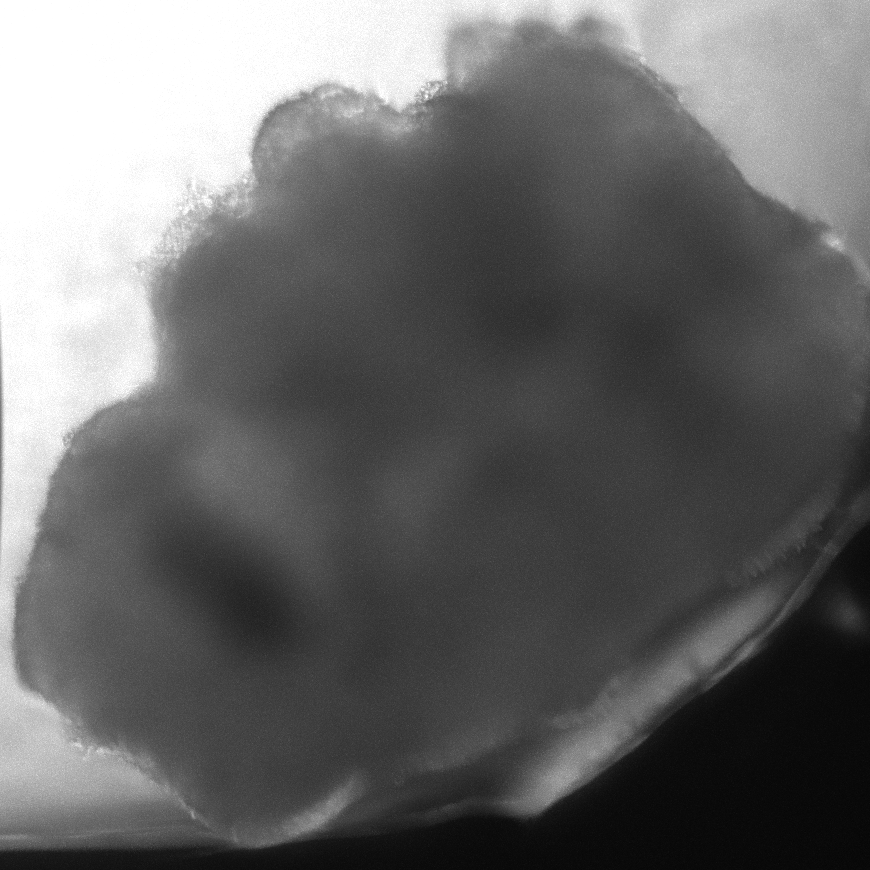

Supplement: Supplementary file 6 — Source Data for Figure 1 [file EMMM-15-e18199-s012.zip › Figure_1A,D,E/1E/Tumor_#15_D21.tif]

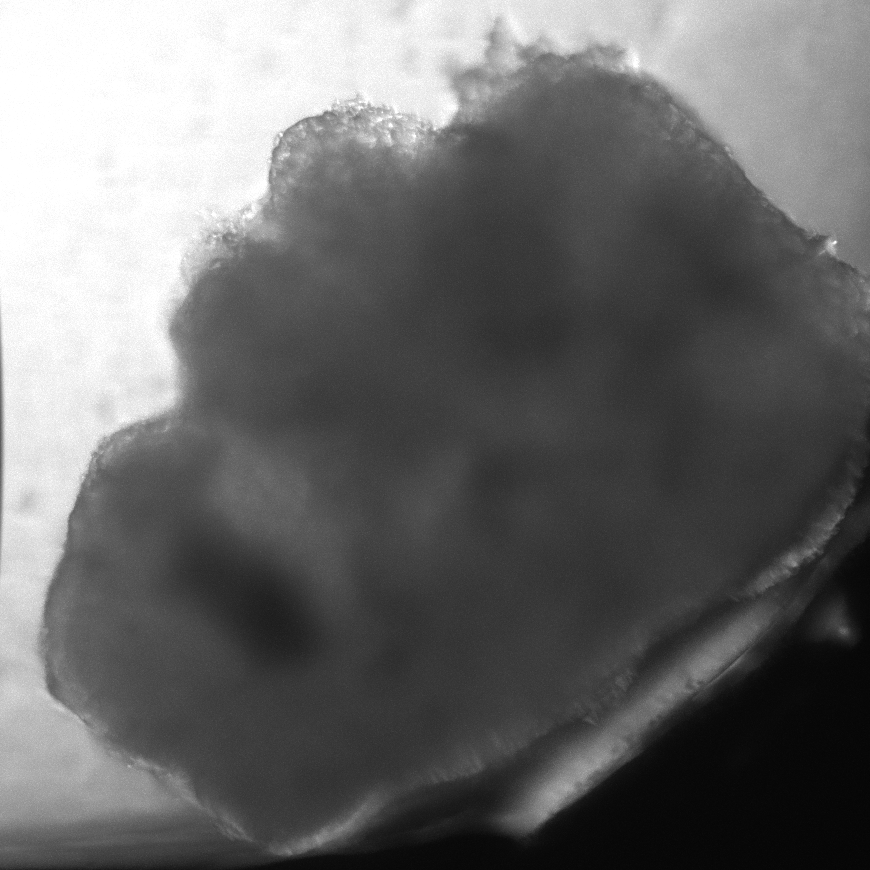

Supplement: Supplementary file 6 — Source Data for Figure 1 [file EMMM-15-e18199-s012.zip › Figure_1A,D,E/1E/Tumor_#15_D28.tif]

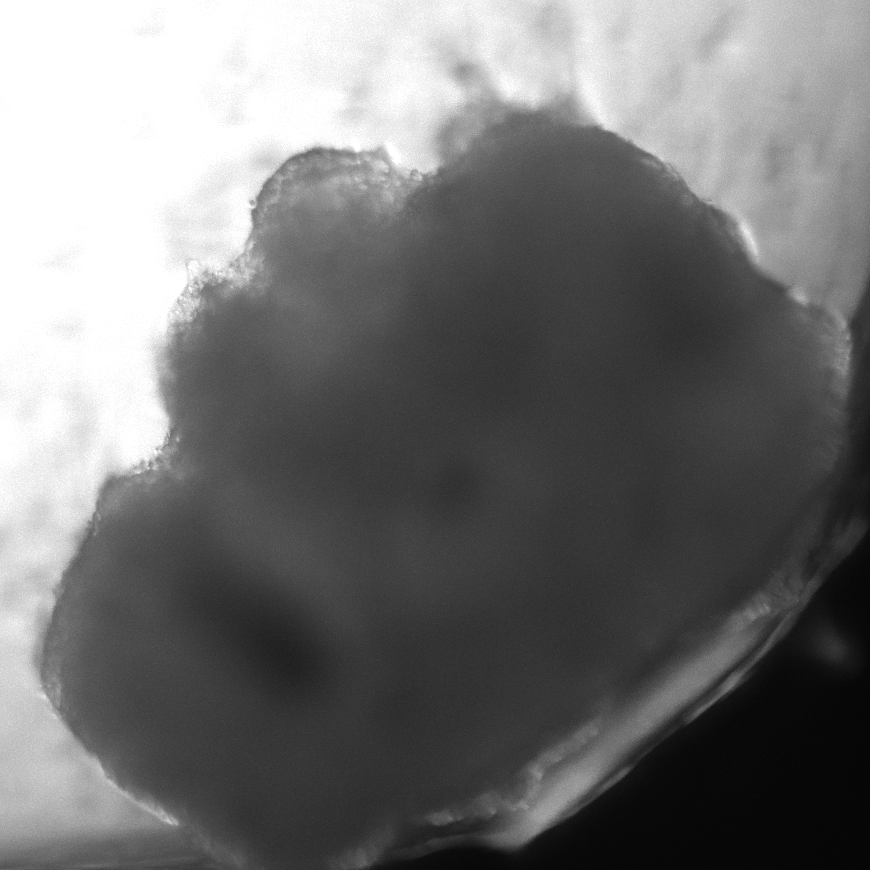

Supplement: Supplementary file 6 — Source Data for Figure 1 [file EMMM-15-e18199-s012.zip › Figure_1A,D,E/1E/Tumor_#15_D53.tif]

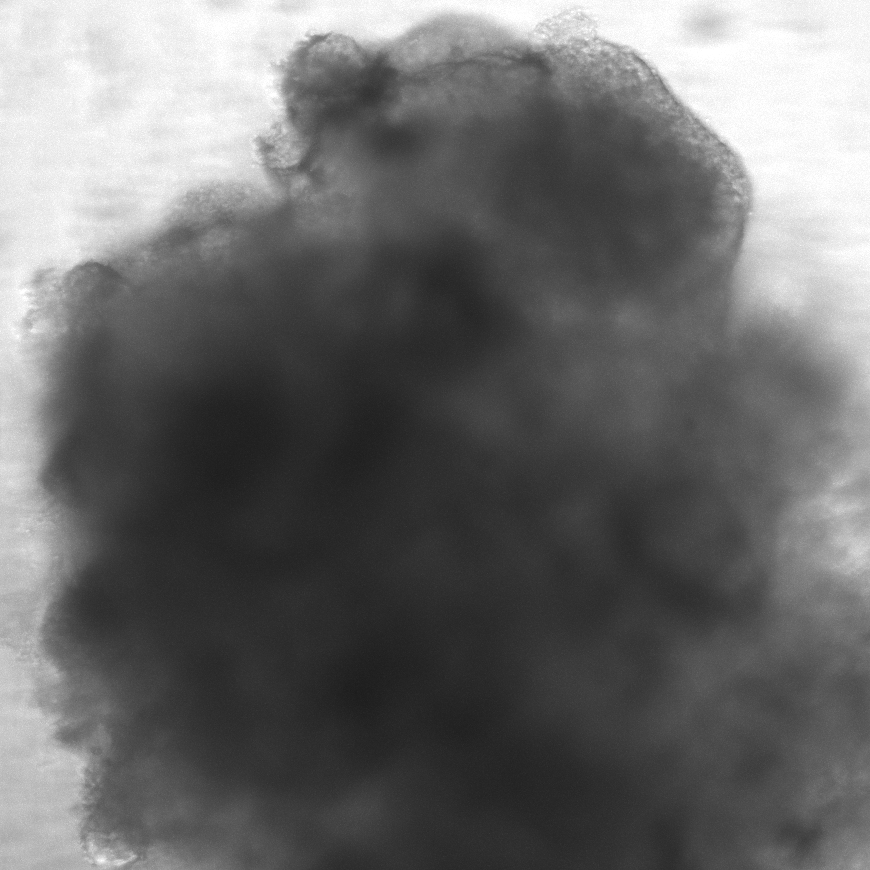

Supplement: Supplementary file 6 — Source Data for Figure 1 [file EMMM-15-e18199-s012.zip › Figure_1A,D,E/1E/Tumor_#15_D7.tif]

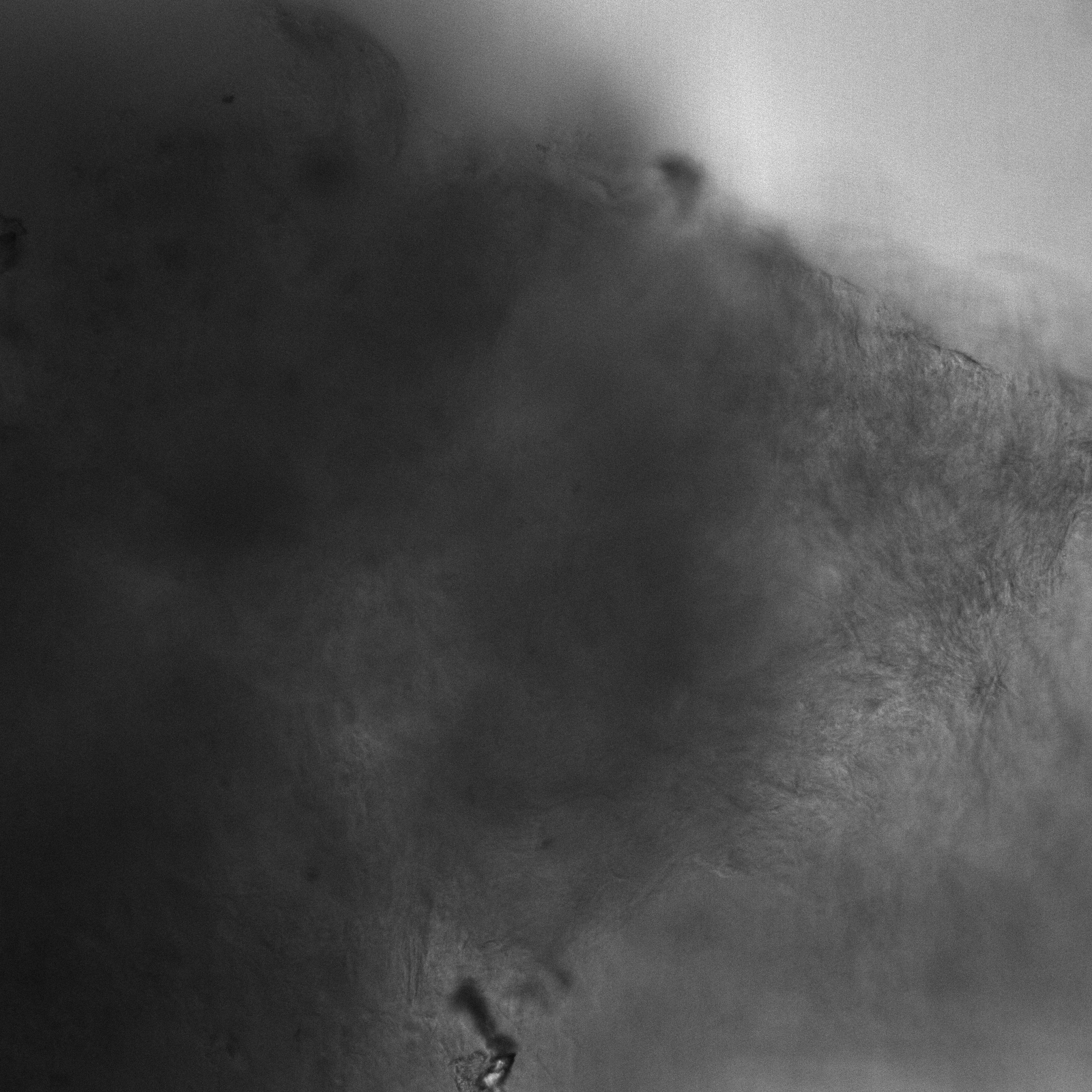

Supplement: Supplementary file 6 — Source Data for Figure 1 [file EMMM-15-e18199-s012.zip › Figure_1A,D,E/1E/Tumor_#8_D14.tif]

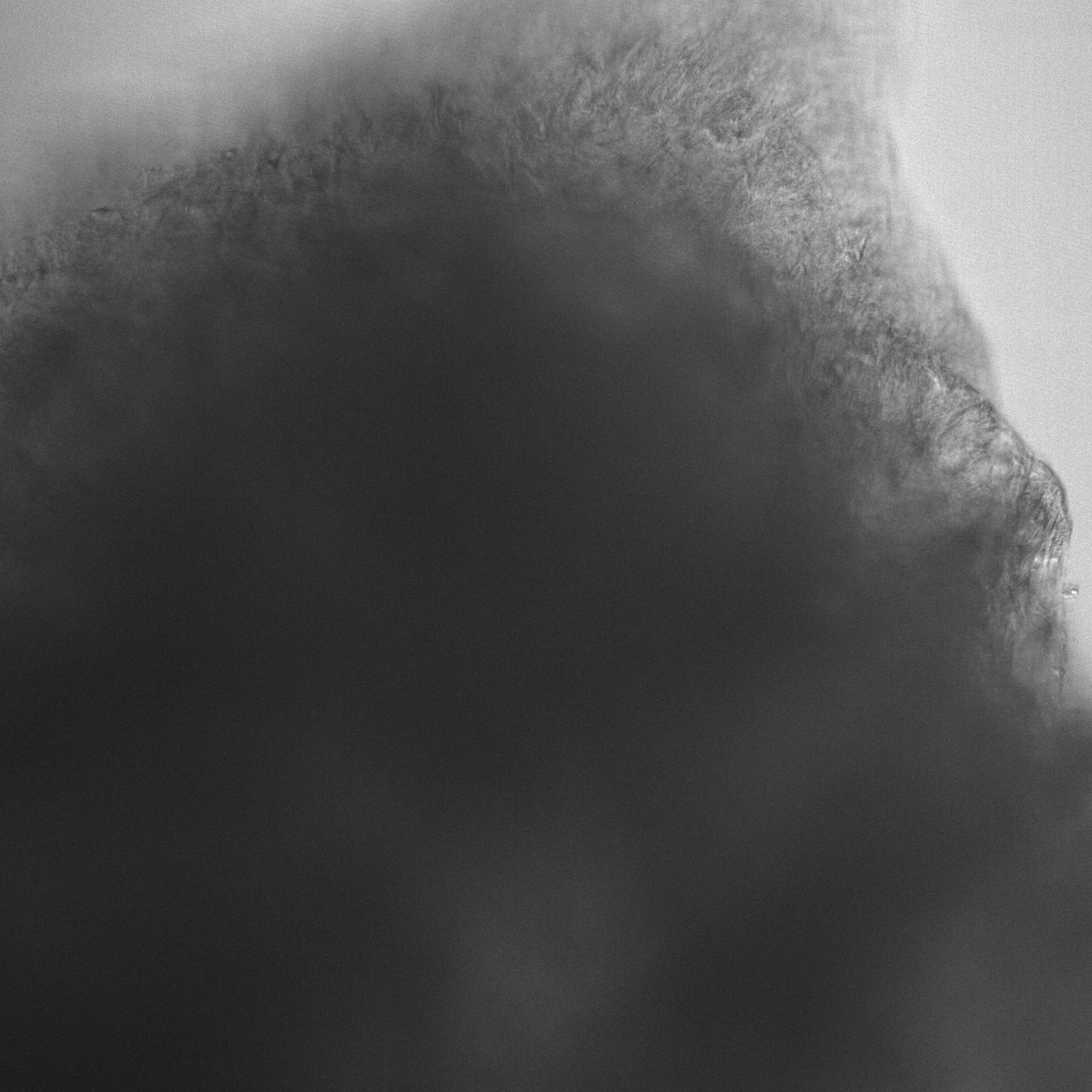

Supplement: Supplementary file 6 — Source Data for Figure 1 [file EMMM-15-e18199-s012.zip › Figure_1A,D,E/1E/Tumor_#8_D21.tif]

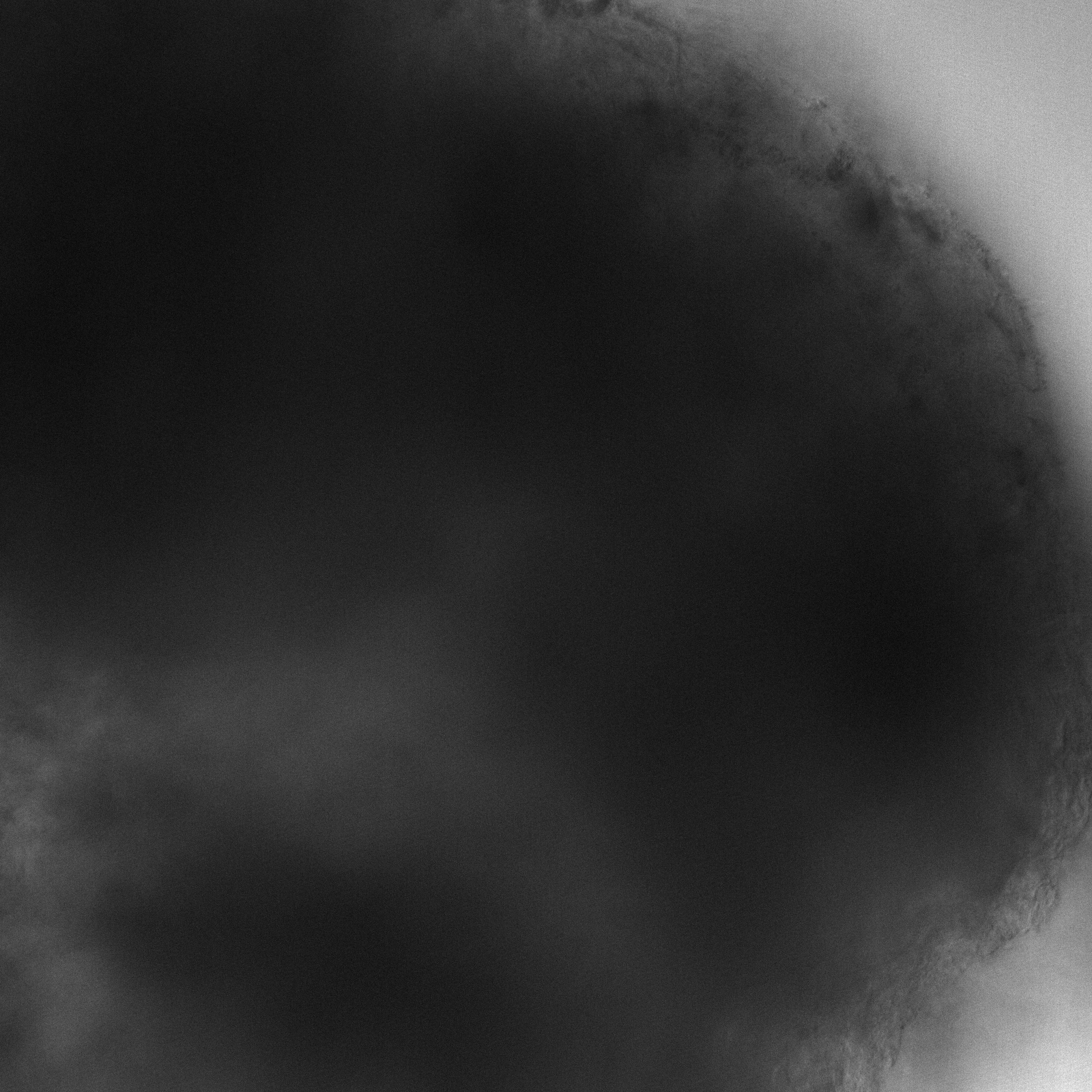

Supplement: Supplementary file 6 — Source Data for Figure 1 [file EMMM-15-e18199-s012.zip › Figure_1A,D,E/1E/Tumor_#8_D28.tif]

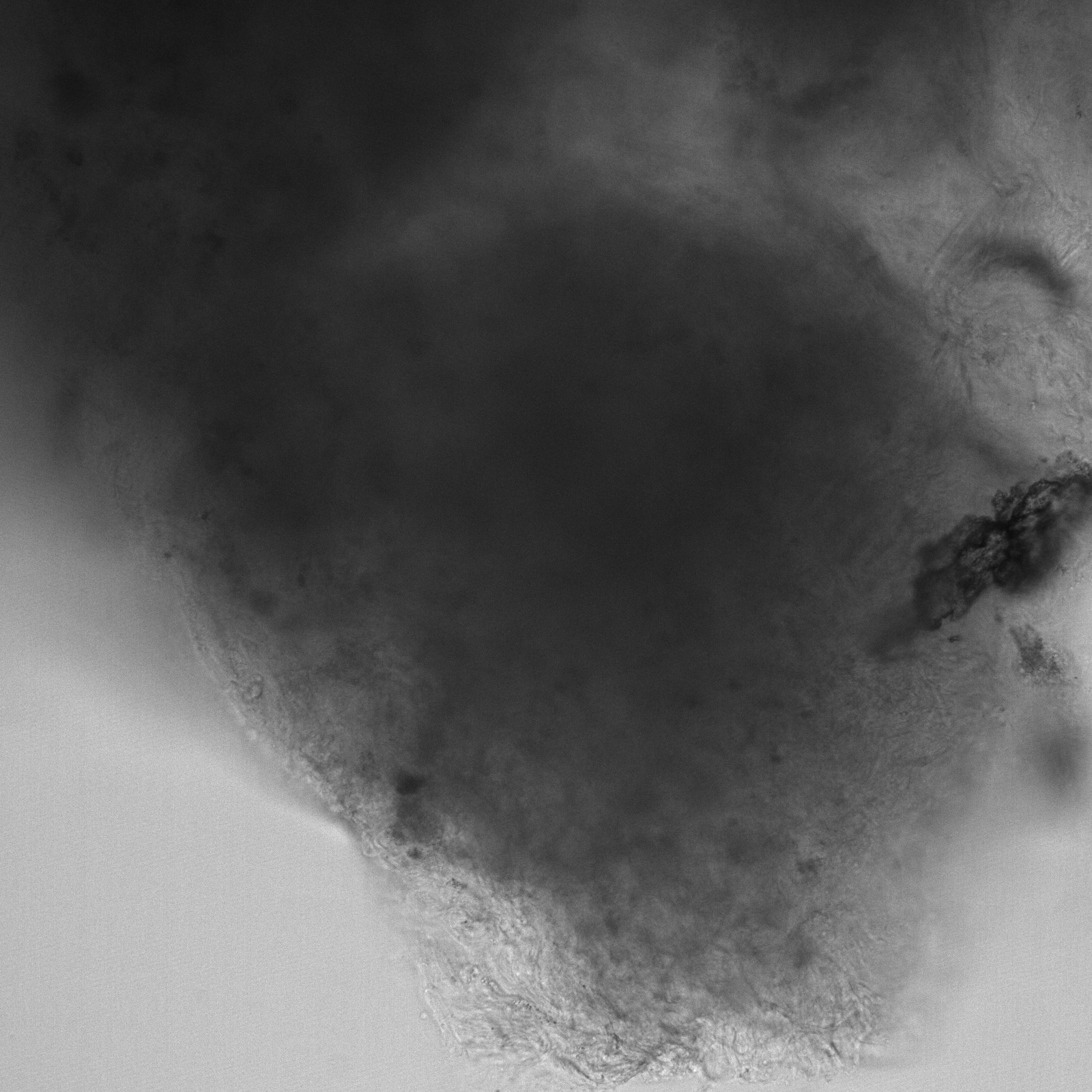

Supplement: Supplementary file 6 — Source Data for Figure 1 [file EMMM-15-e18199-s012.zip › Figure_1A,D,E/1E/Tumor_#8_D7.tif]

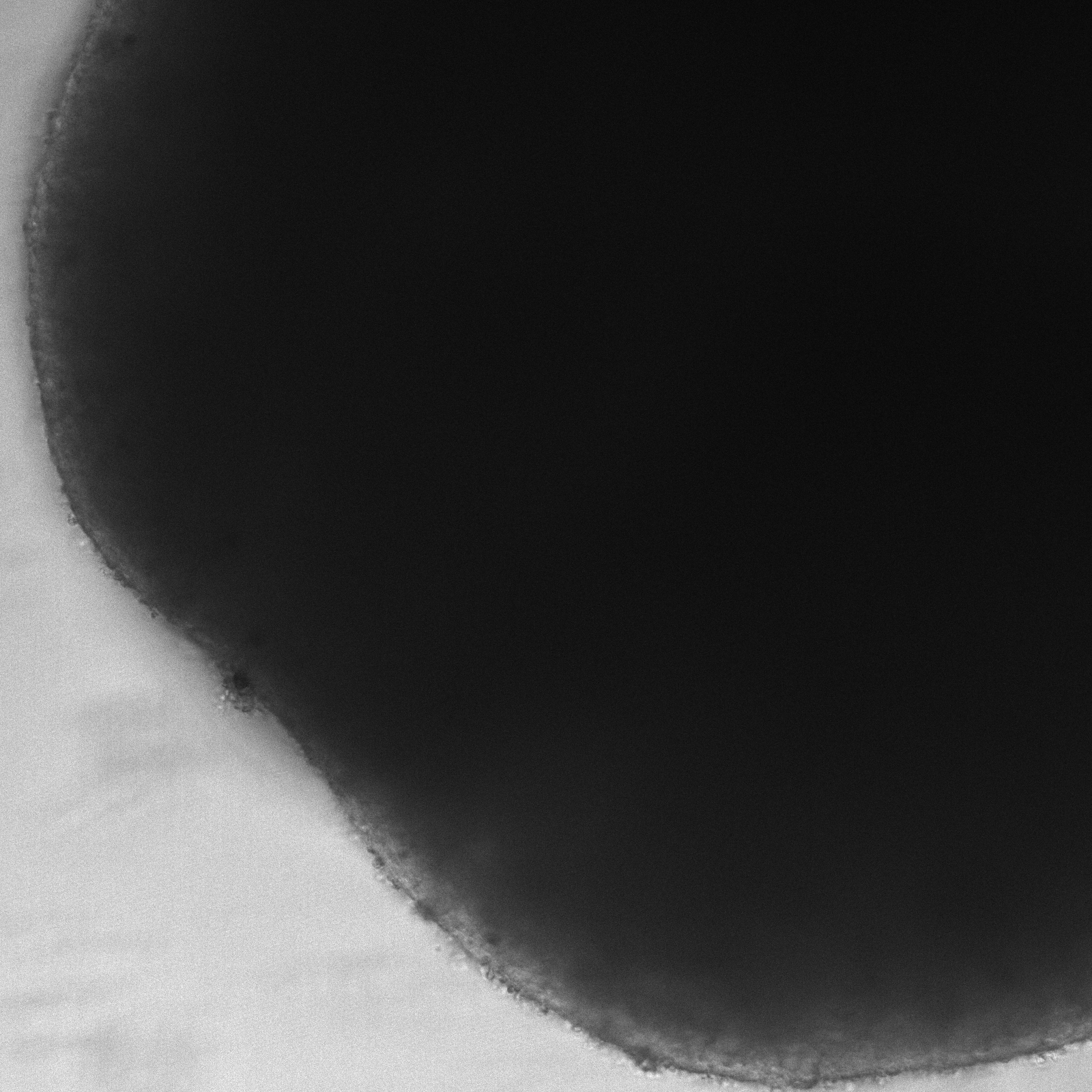

Supplement: Supplementary file 6 — Source Data for Figure 1 [file EMMM-15-e18199-s012.zip › Figure_1A,D,E/1E/Tumor_#9_D14.tif]

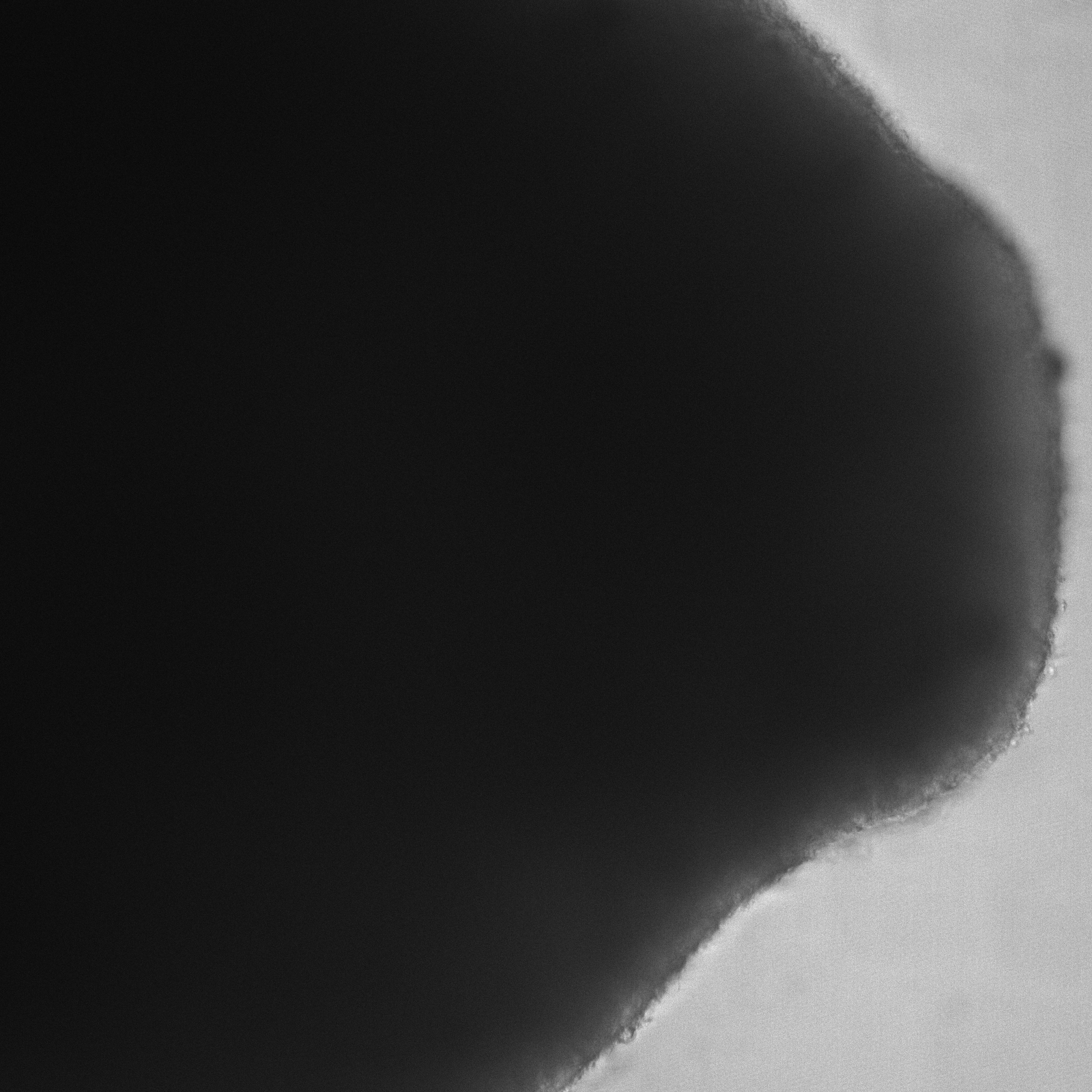

Supplement: Supplementary file 6 — Source Data for Figure 1 [file EMMM-15-e18199-s012.zip › Figure_1A,D,E/1E/Tumor_#9_D21.tif]

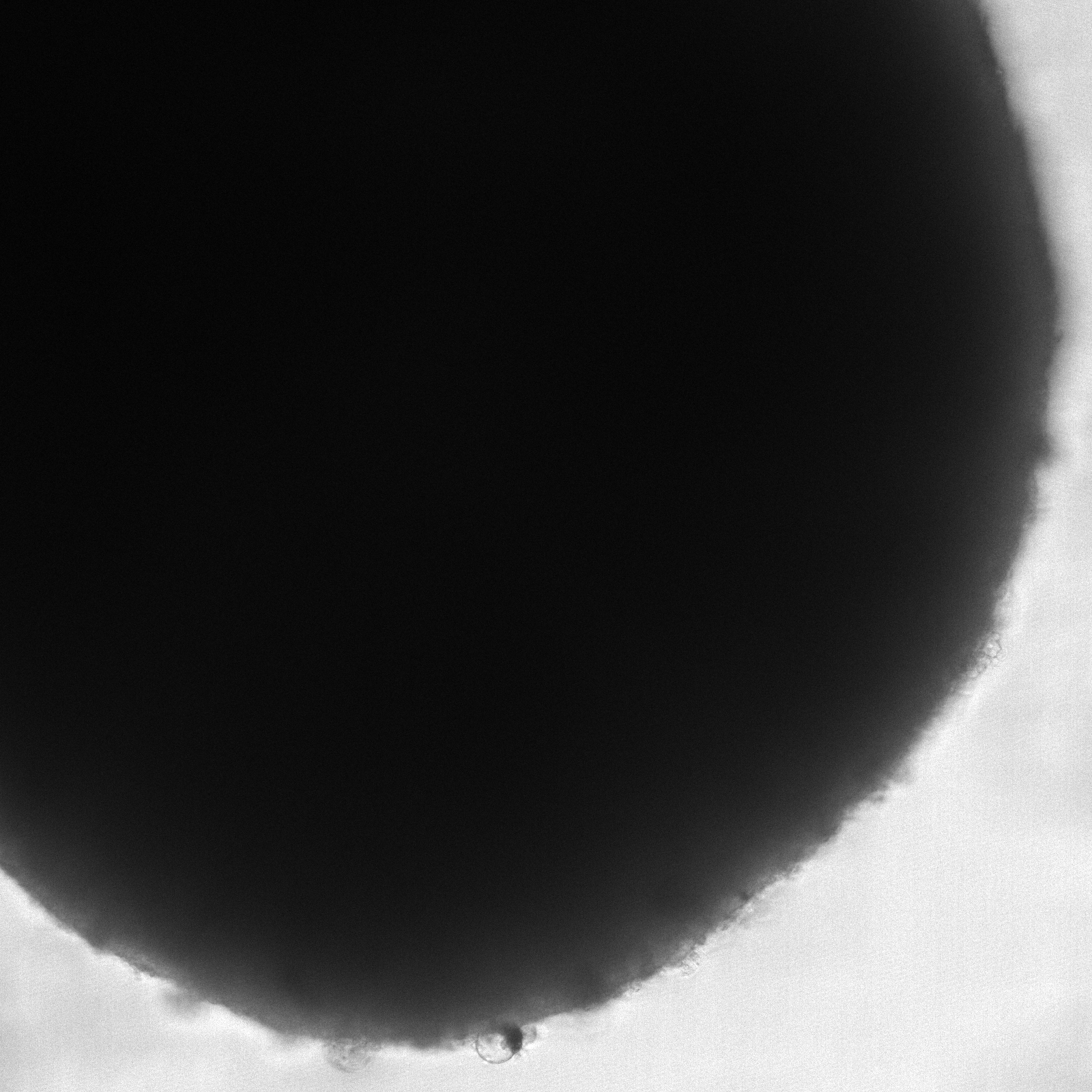

Supplement: Supplementary file 6 — Source Data for Figure 1 [file EMMM-15-e18199-s012.zip › Figure_1A,D,E/1E/Tumor_#9_D28.tif]

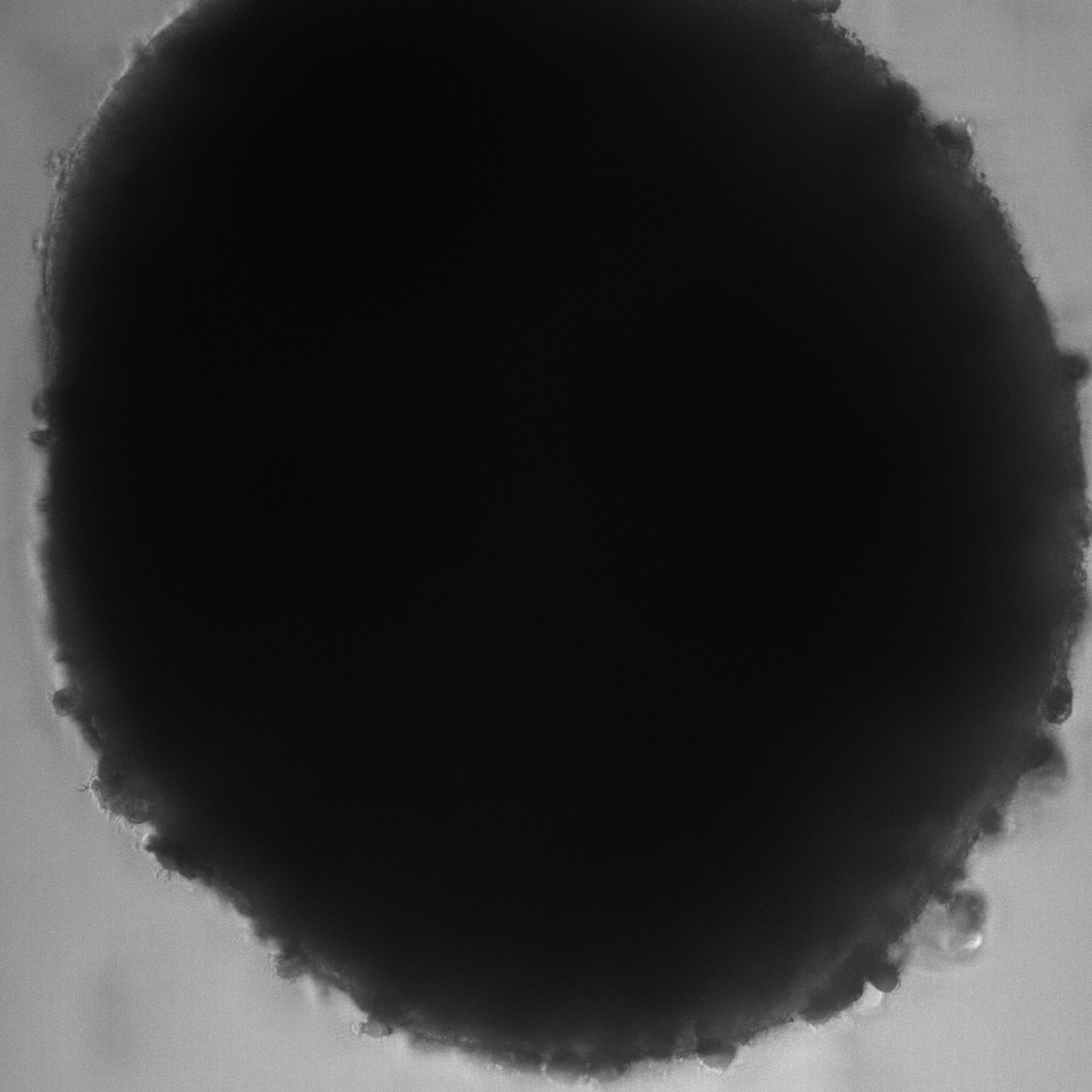

Supplement: Supplementary file 6 — Source Data for Figure 1 [file EMMM-15-e18199-s012.zip › Figure_1A,D,E/1E/Tumor_#9_D35.tif]

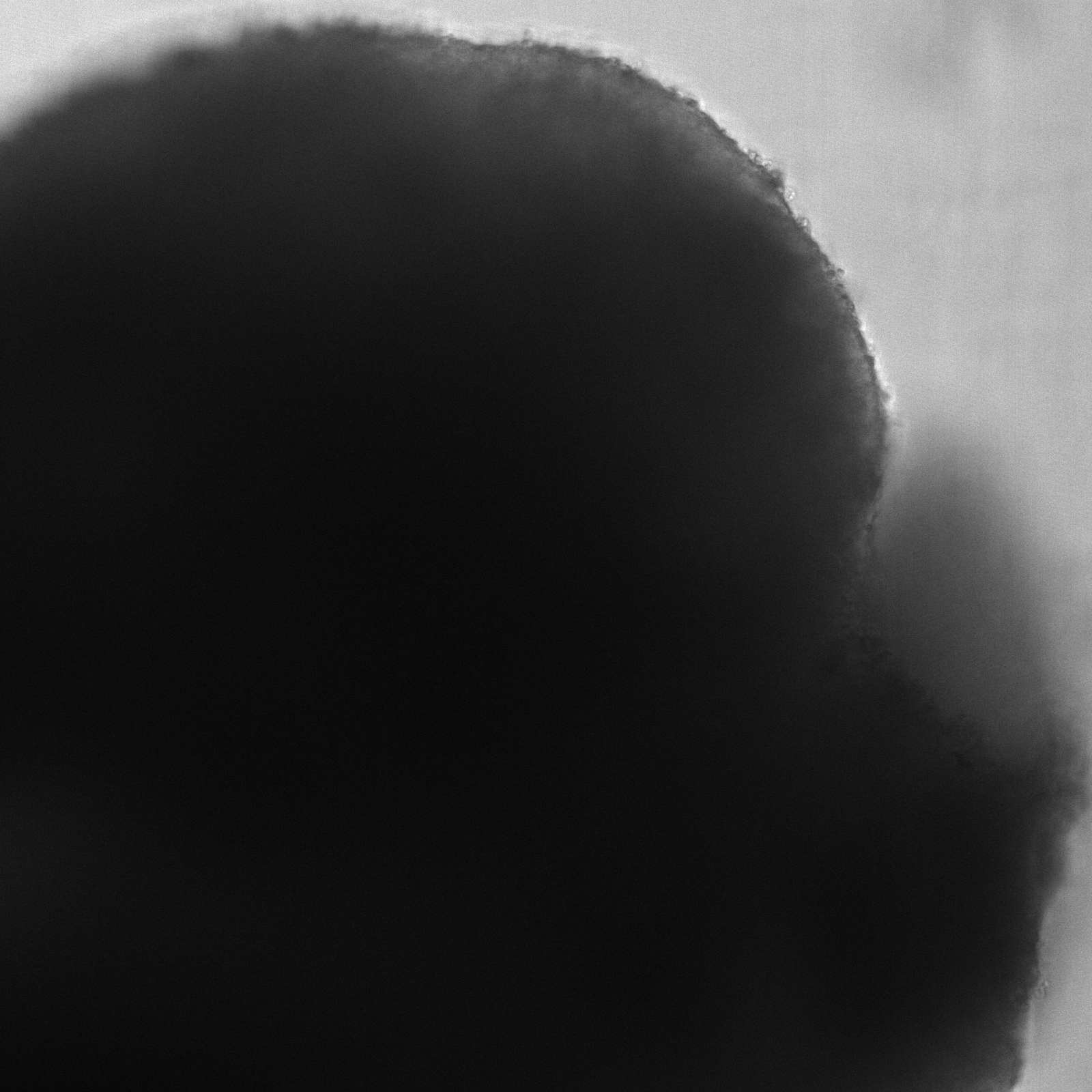

Supplement: Supplementary file 6 — Source Data for Figure 1 [file EMMM-15-e18199-s012.zip › Figure_1A,D,E/1E/Tumor_#9_D7.tif]

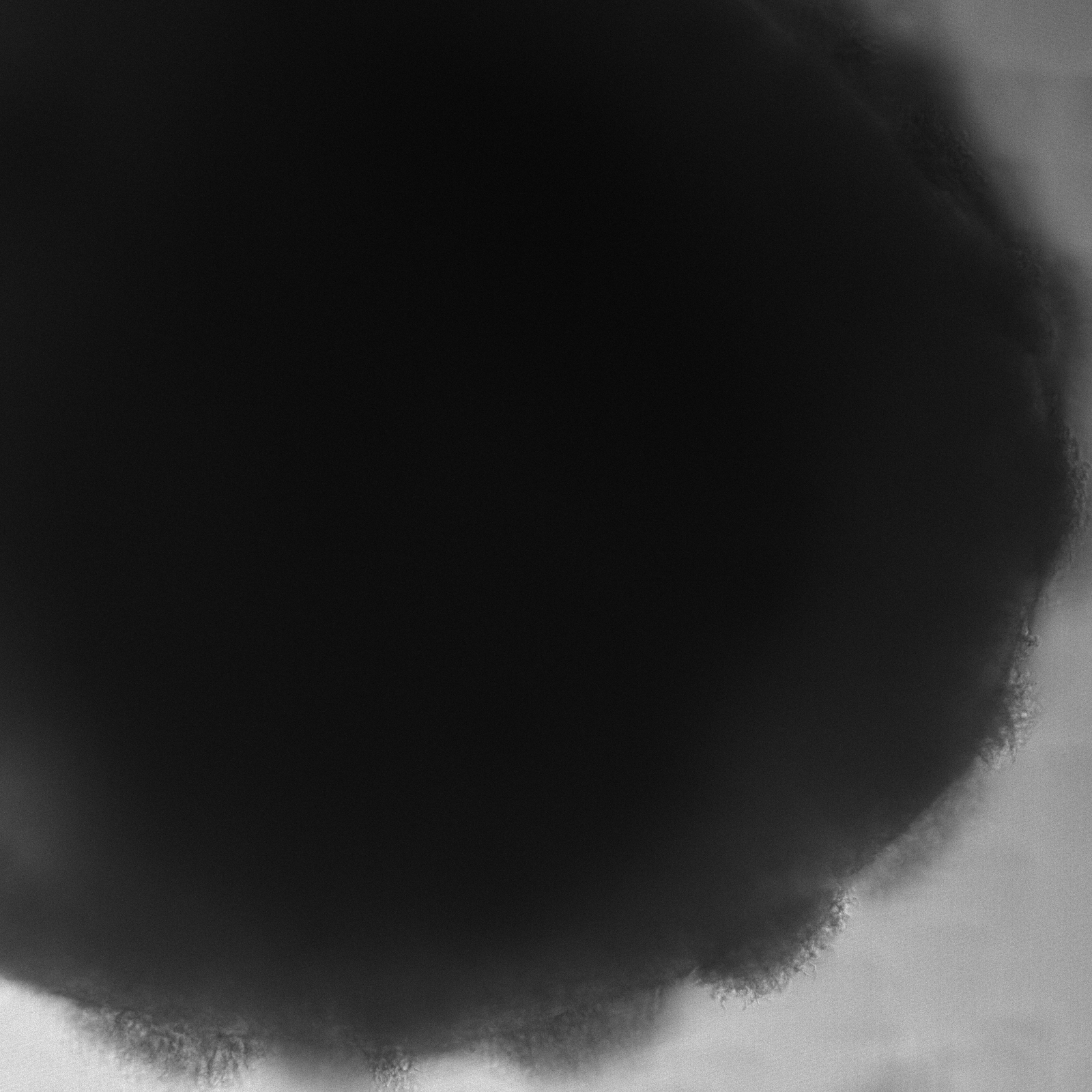

Supplement: Supplementary file 6 — Source Data for Figure 1 [file EMMM-15-e18199-s012.zip › Figure_1F,G,H/1F/Tumor_#16_D14.tif]

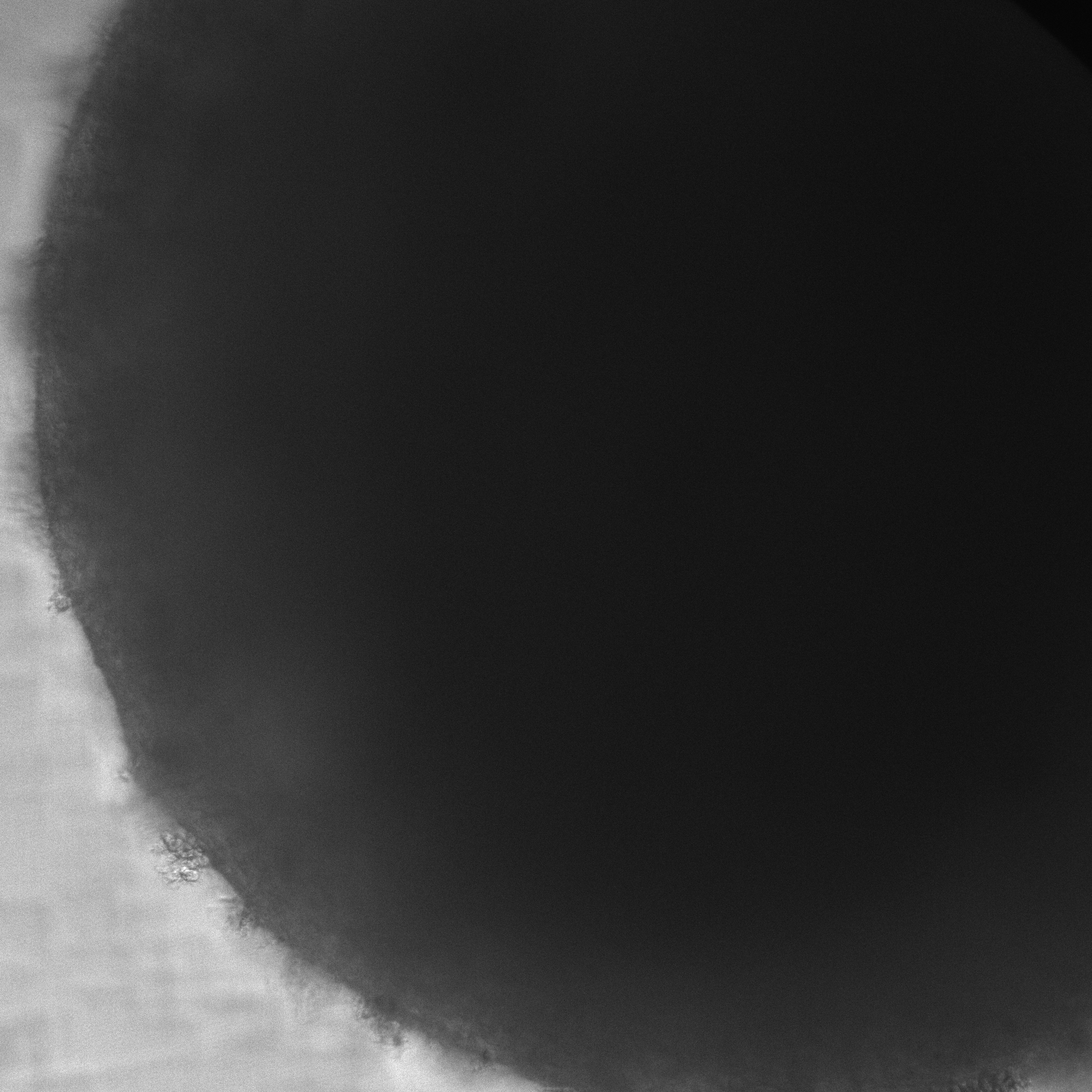

Supplement: Supplementary file 6 — Source Data for Figure 1 [file EMMM-15-e18199-s012.zip › Figure_1F,G,H/1F/Tumor_#16_D21.tif]

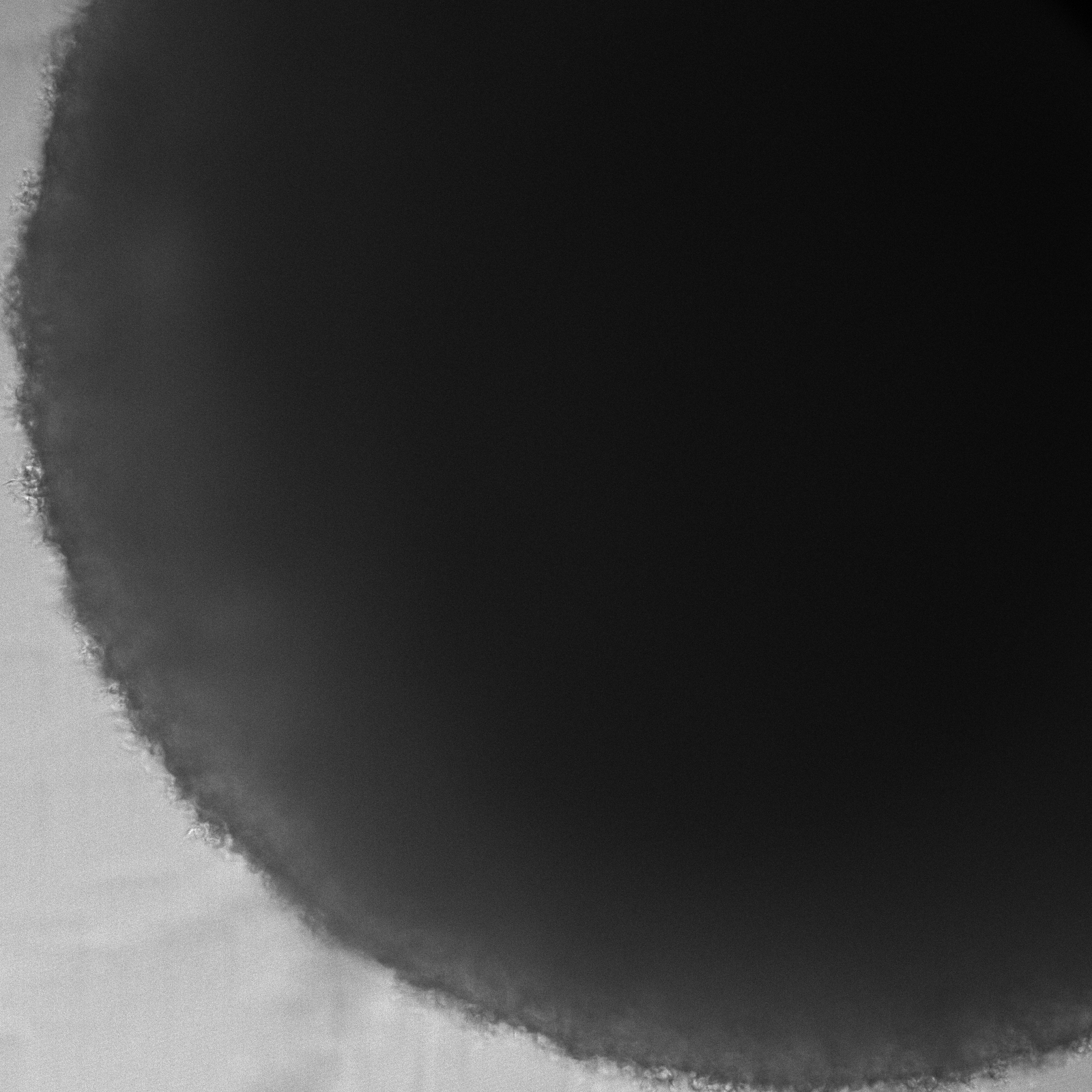

Supplement: Supplementary file 6 — Source Data for Figure 1 [file EMMM-15-e18199-s012.zip › Figure_1F,G,H/1F/Tumor_#16_D28.tif]

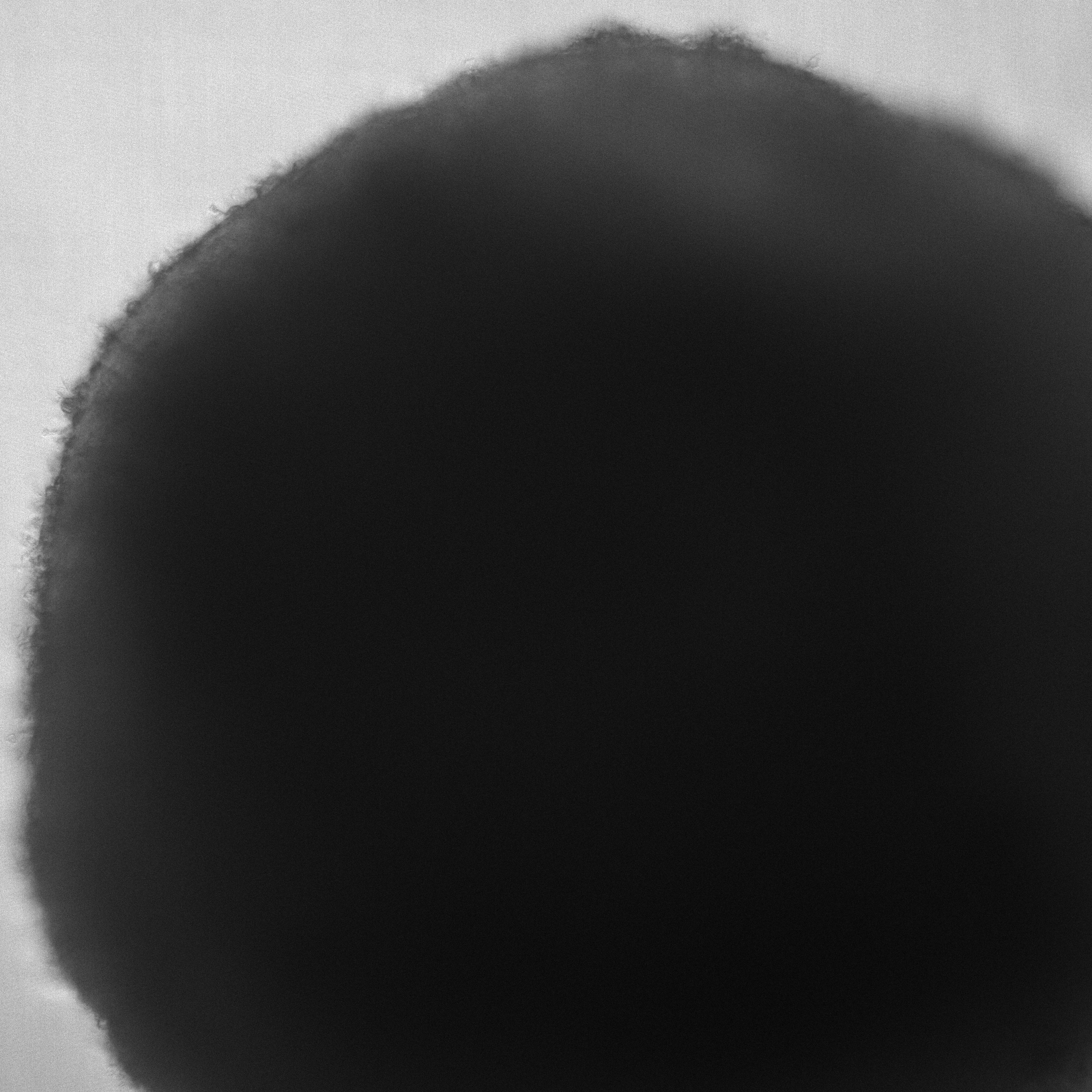

Supplement: Supplementary file 6 — Source Data for Figure 1 [file EMMM-15-e18199-s012.zip › Figure_1F,G,H/1F/Tumor_#16_D7.tif]

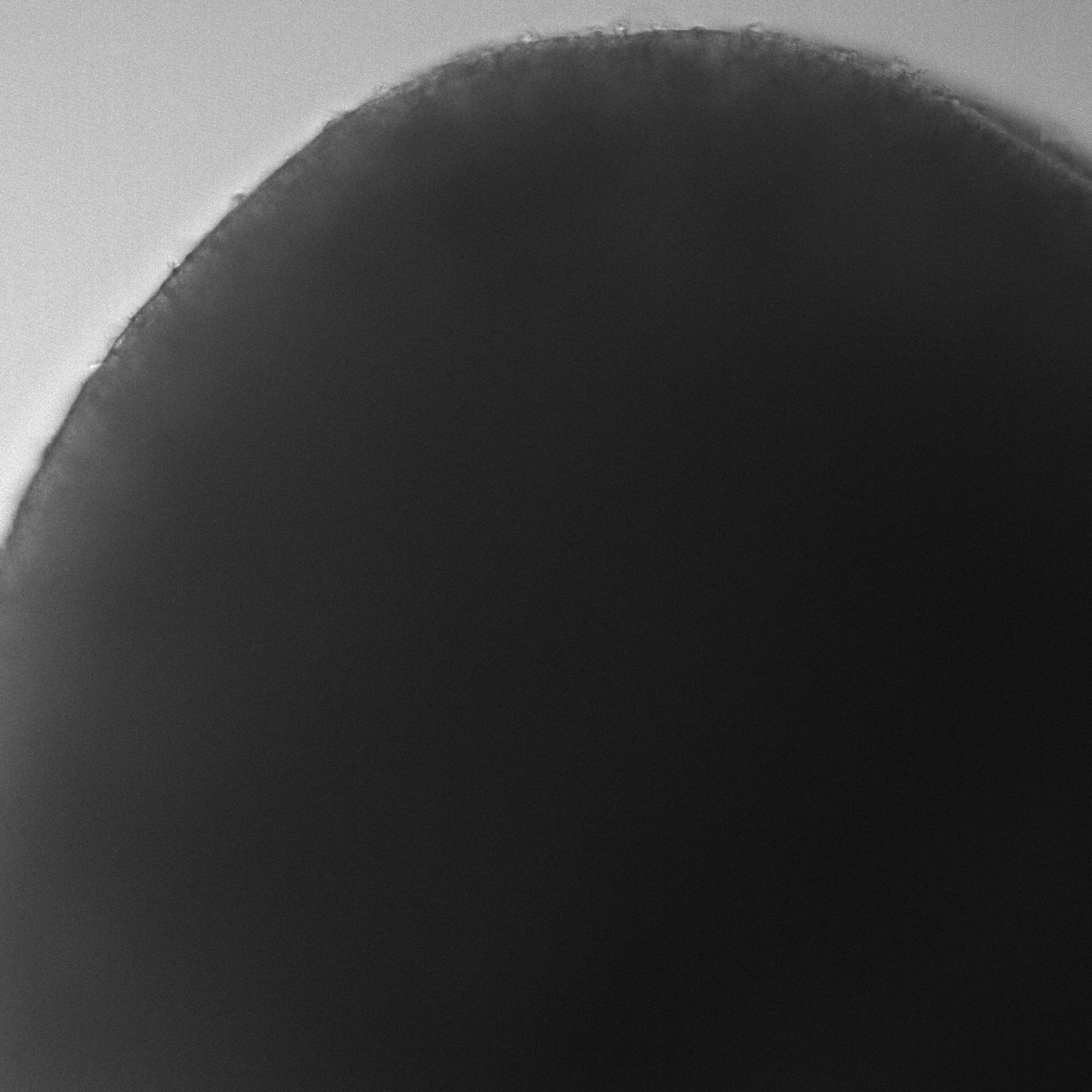

Supplement: Supplementary file 6 — Source Data for Figure 1 [file EMMM-15-e18199-s012.zip › Figure_1F,G,H/1F/Tumor_#17_D14.tif]

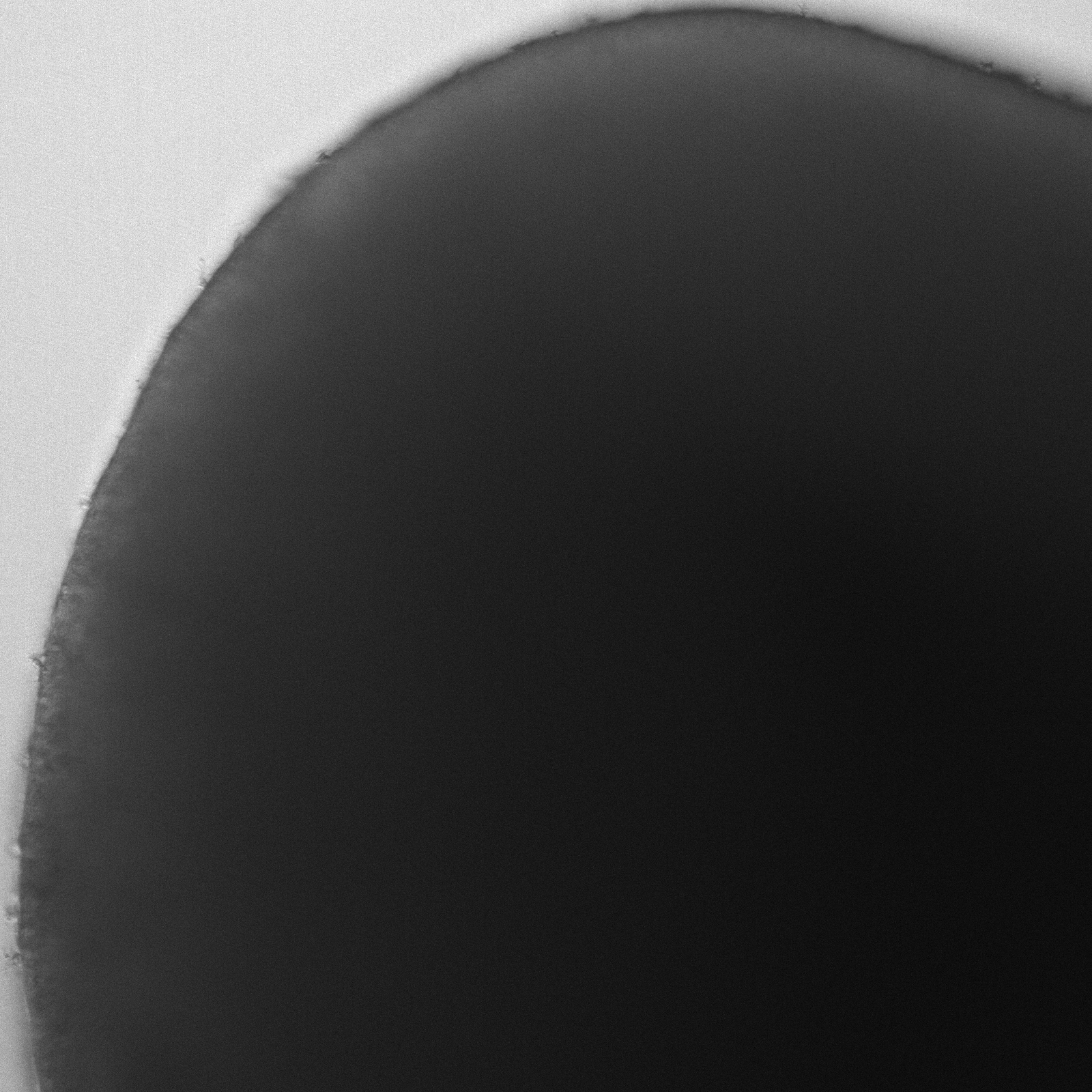

Supplement: Supplementary file 6 — Source Data for Figure 1 [file EMMM-15-e18199-s012.zip › Figure_1F,G,H/1F/Tumor_#17_D21.tif]

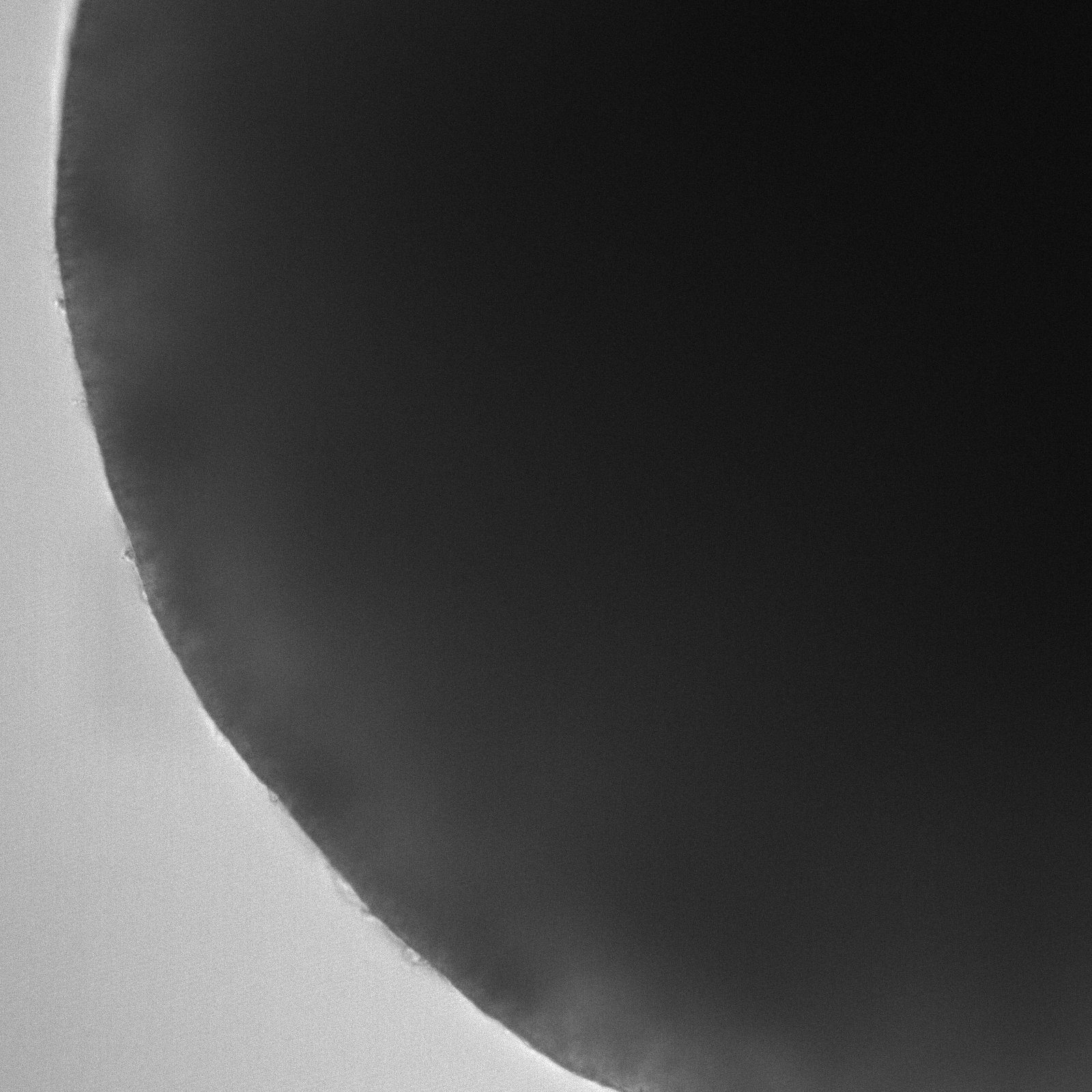

Supplement: Supplementary file 6 — Source Data for Figure 1 [file EMMM-15-e18199-s012.zip › Figure_1F,G,H/1F/Tumor_#17_D28.tif]

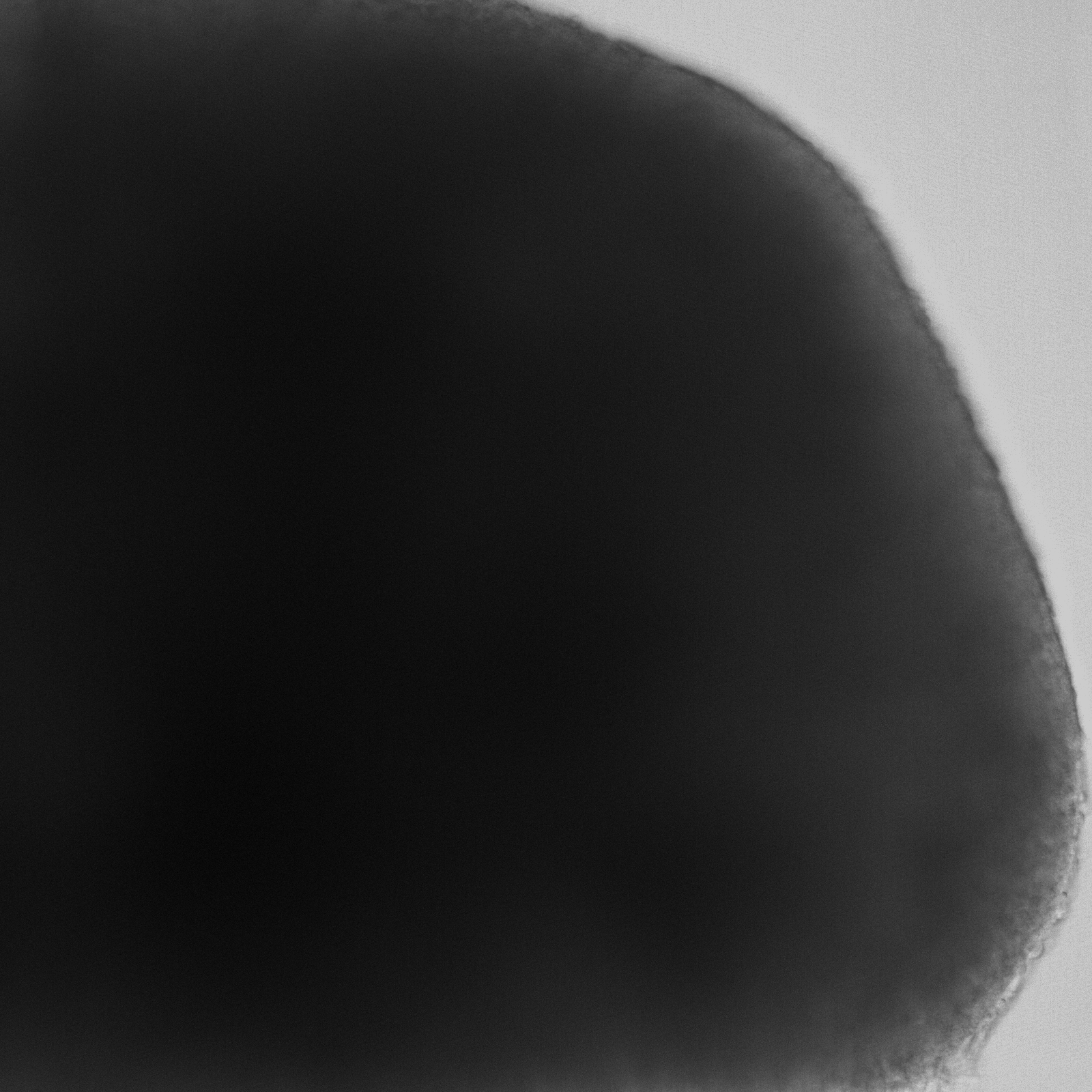

Supplement: Supplementary file 6 — Source Data for Figure 1 [file EMMM-15-e18199-s012.zip › Figure_1F,G,H/1F/Tumor_#17_D7.tif]

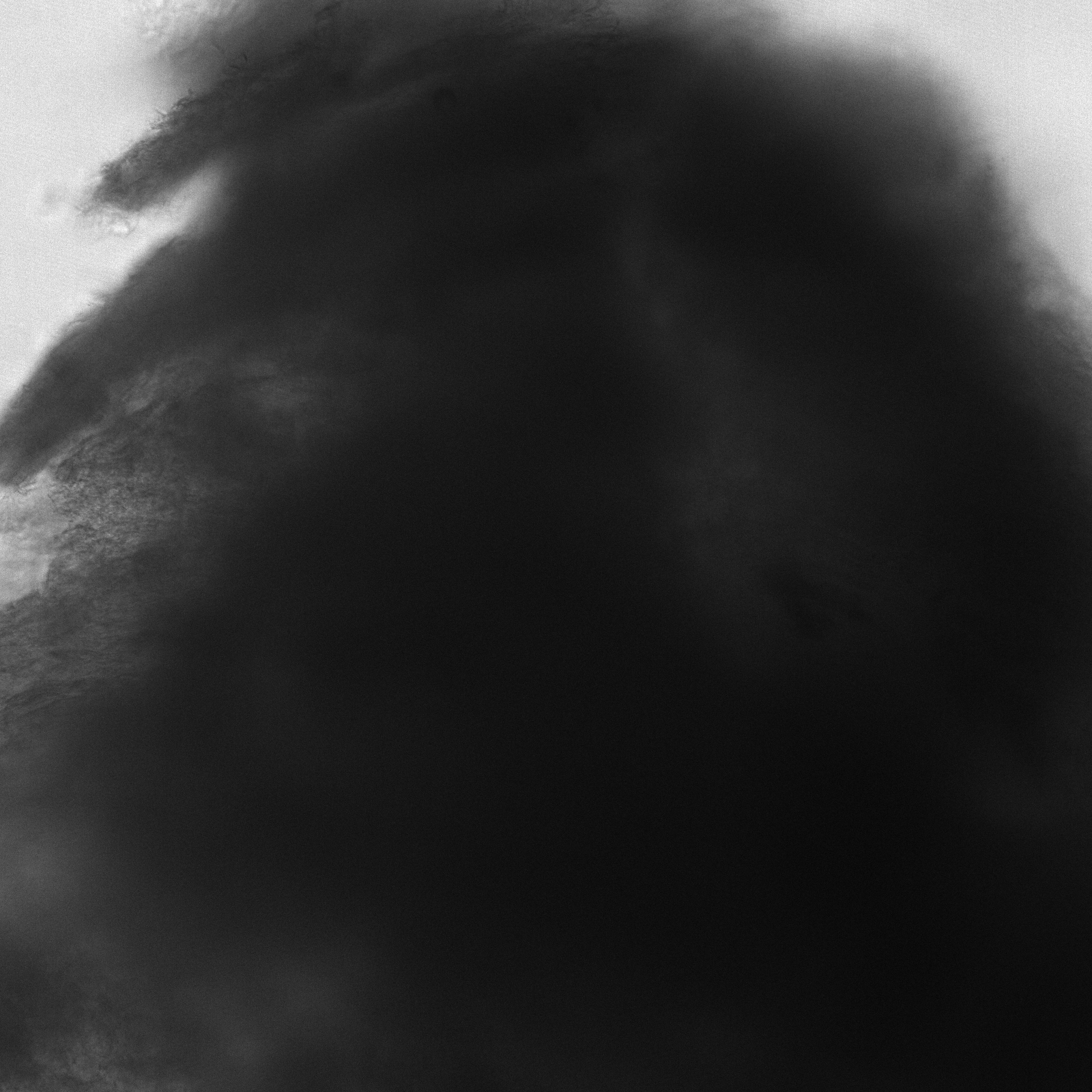

Supplement: Supplementary file 6 — Source Data for Figure 1 [file EMMM-15-e18199-s012.zip › Figure_1F,G,H/1F/Tumor_#18_D14.tif]

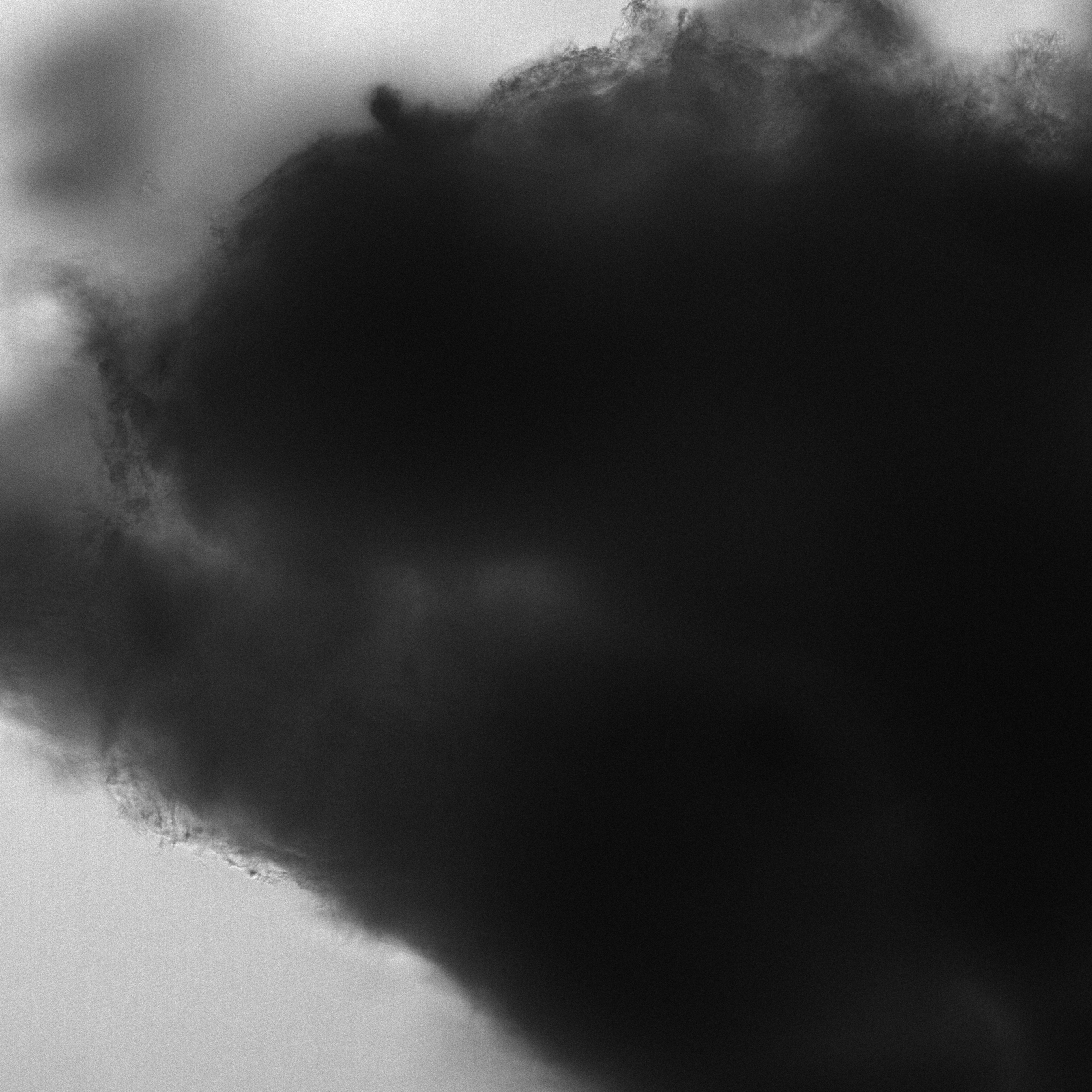

Supplement: Supplementary file 6 — Source Data for Figure 1 [file EMMM-15-e18199-s012.zip › Figure_1F,G,H/1F/Tumor_#18_D21.tif]

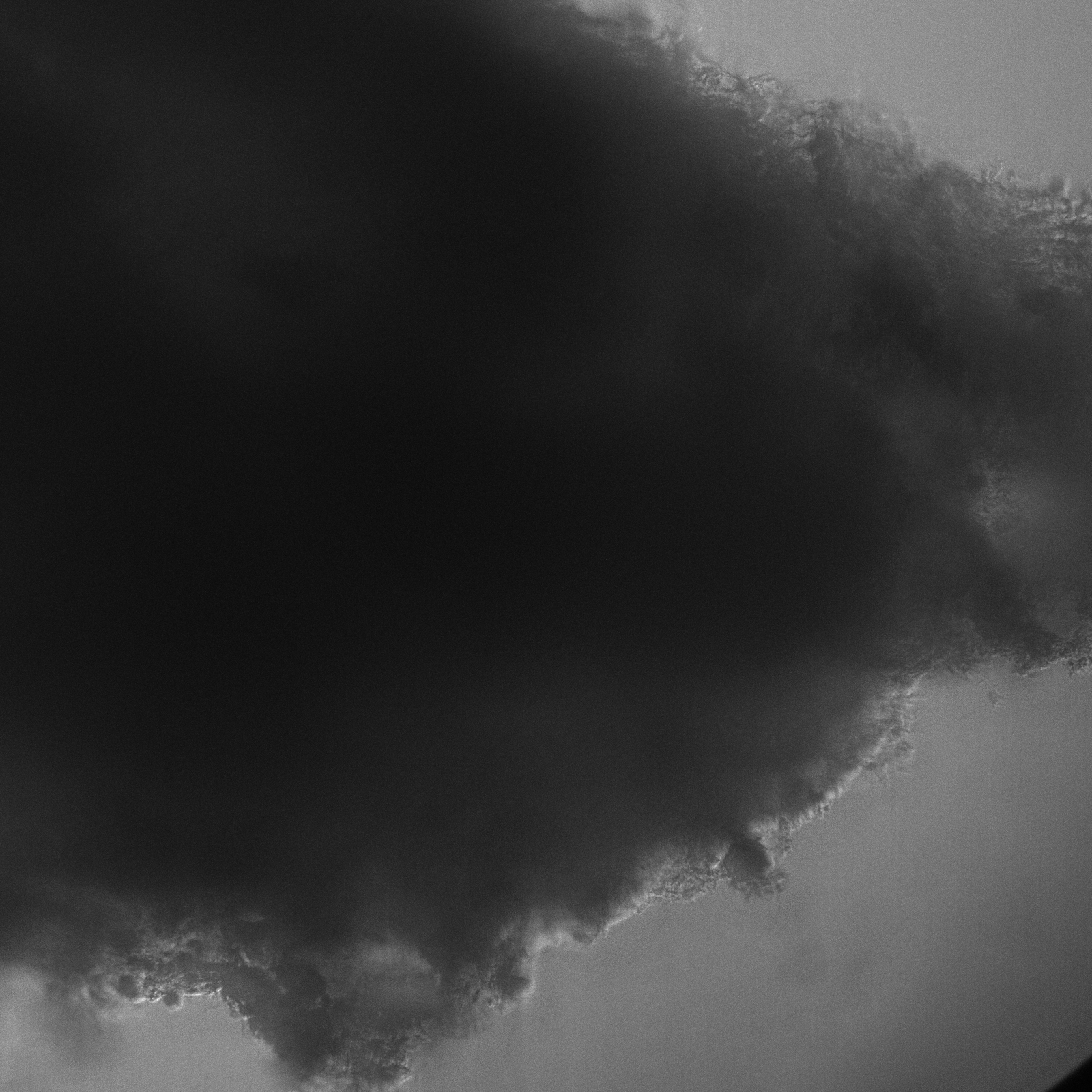

Supplement: Supplementary file 6 — Source Data for Figure 1 [file EMMM-15-e18199-s012.zip › Figure_1F,G,H/1F/Tumor_#18_D28.tif]

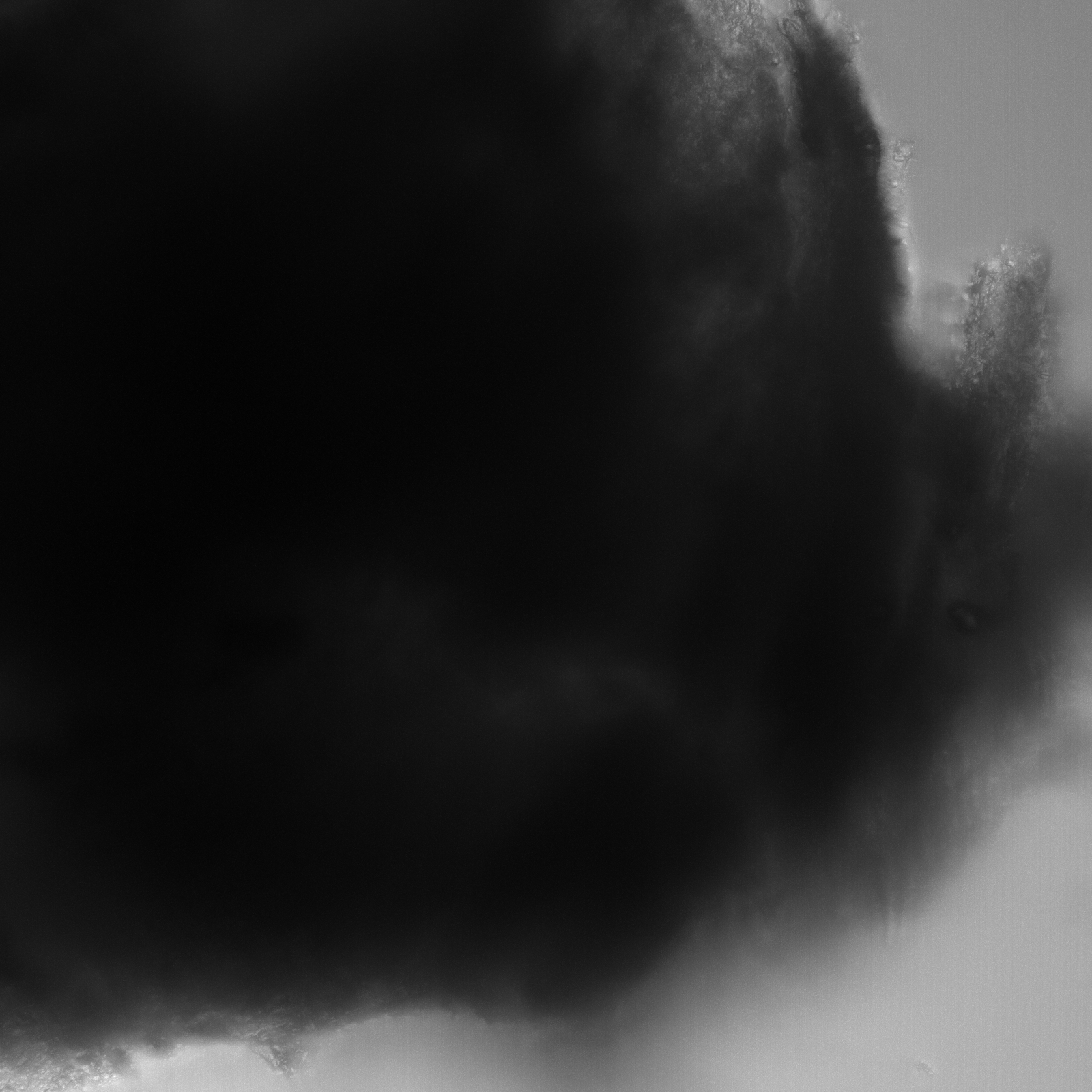

Supplement: Supplementary file 6 — Source Data for Figure 1 [file EMMM-15-e18199-s012.zip › Figure_1F,G,H/1F/Tumor_#18_D7.tif]

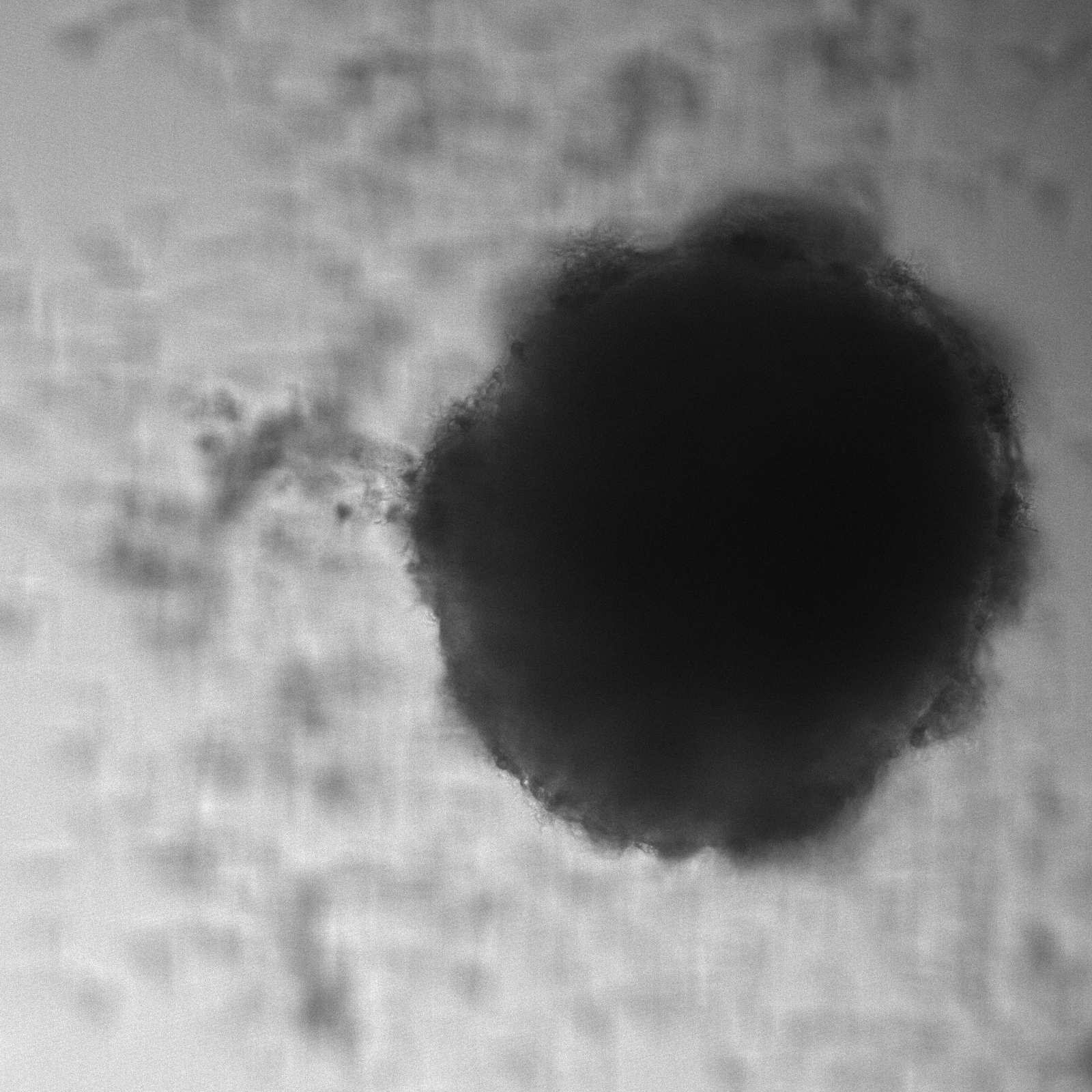

Supplement: Supplementary file 6 — Source Data for Figure 1 [file EMMM-15-e18199-s012.zip › Figure_1F,G,H/1F/Tumor_#19_D14.tif]

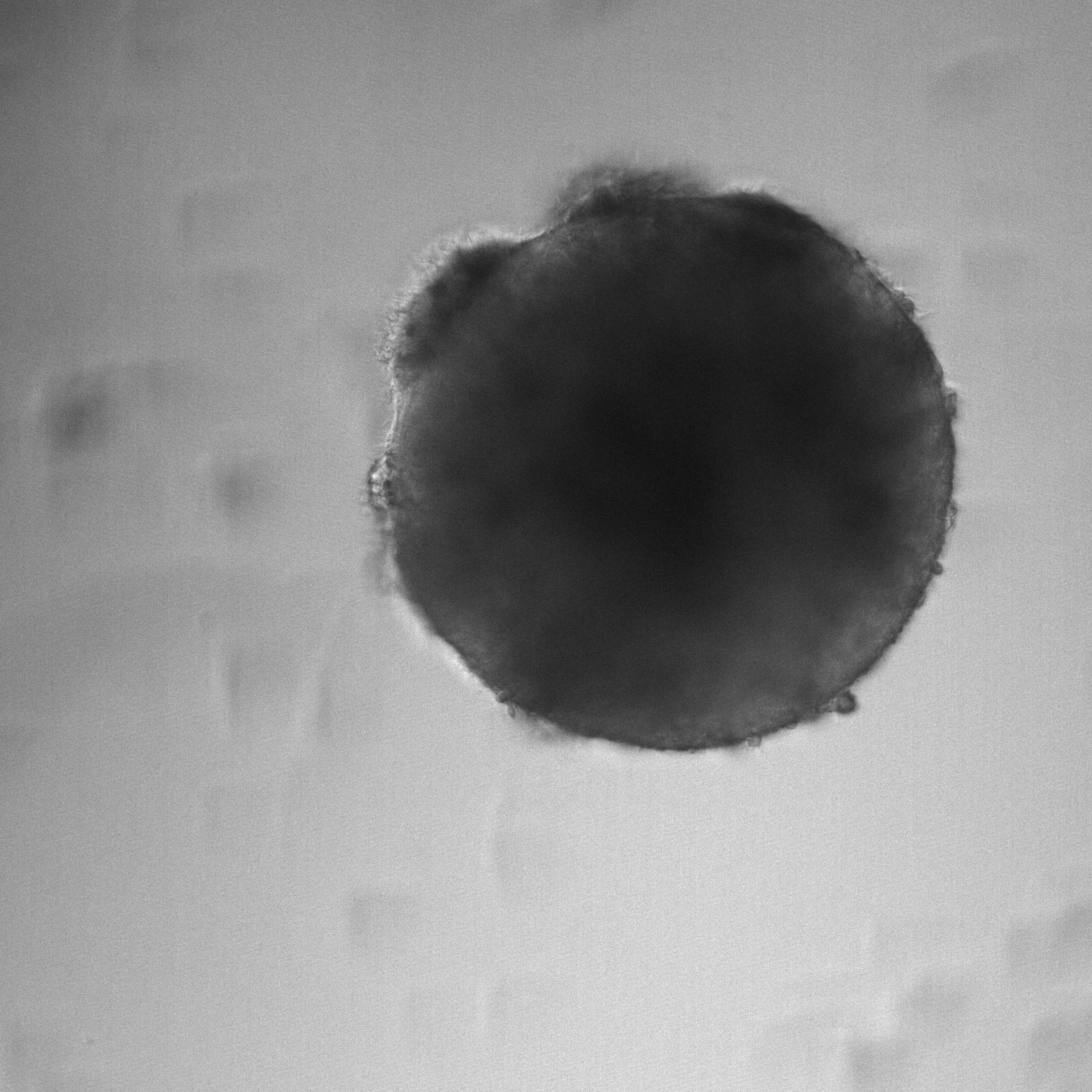

Supplement: Supplementary file 6 — Source Data for Figure 1 [file EMMM-15-e18199-s012.zip › Figure_1F,G,H/1F/Tumor_#19_D21.tif]

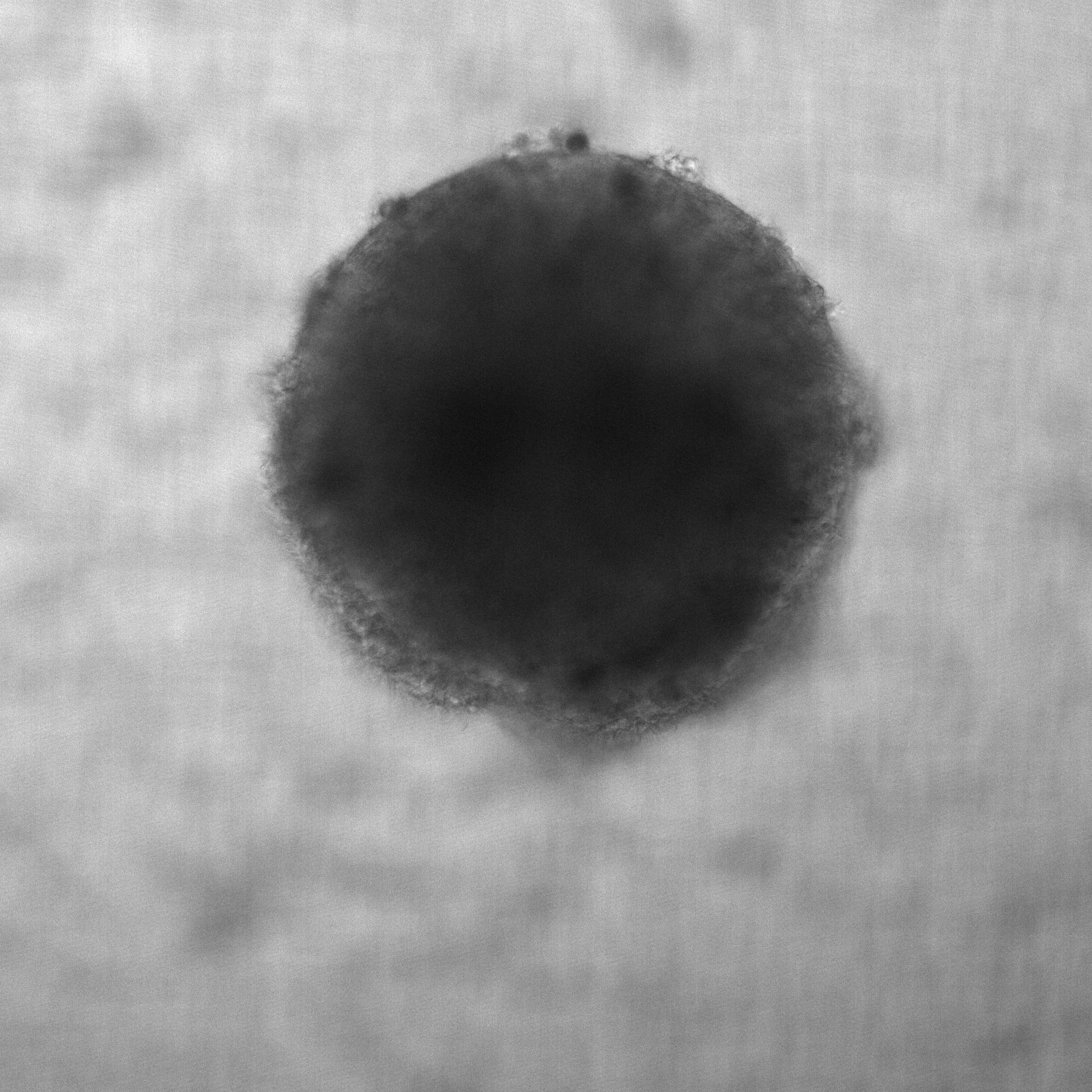

Supplement: Supplementary file 6 — Source Data for Figure 1 [file EMMM-15-e18199-s012.zip › Figure_1F,G,H/1F/Tumor_#19_D28.tif]

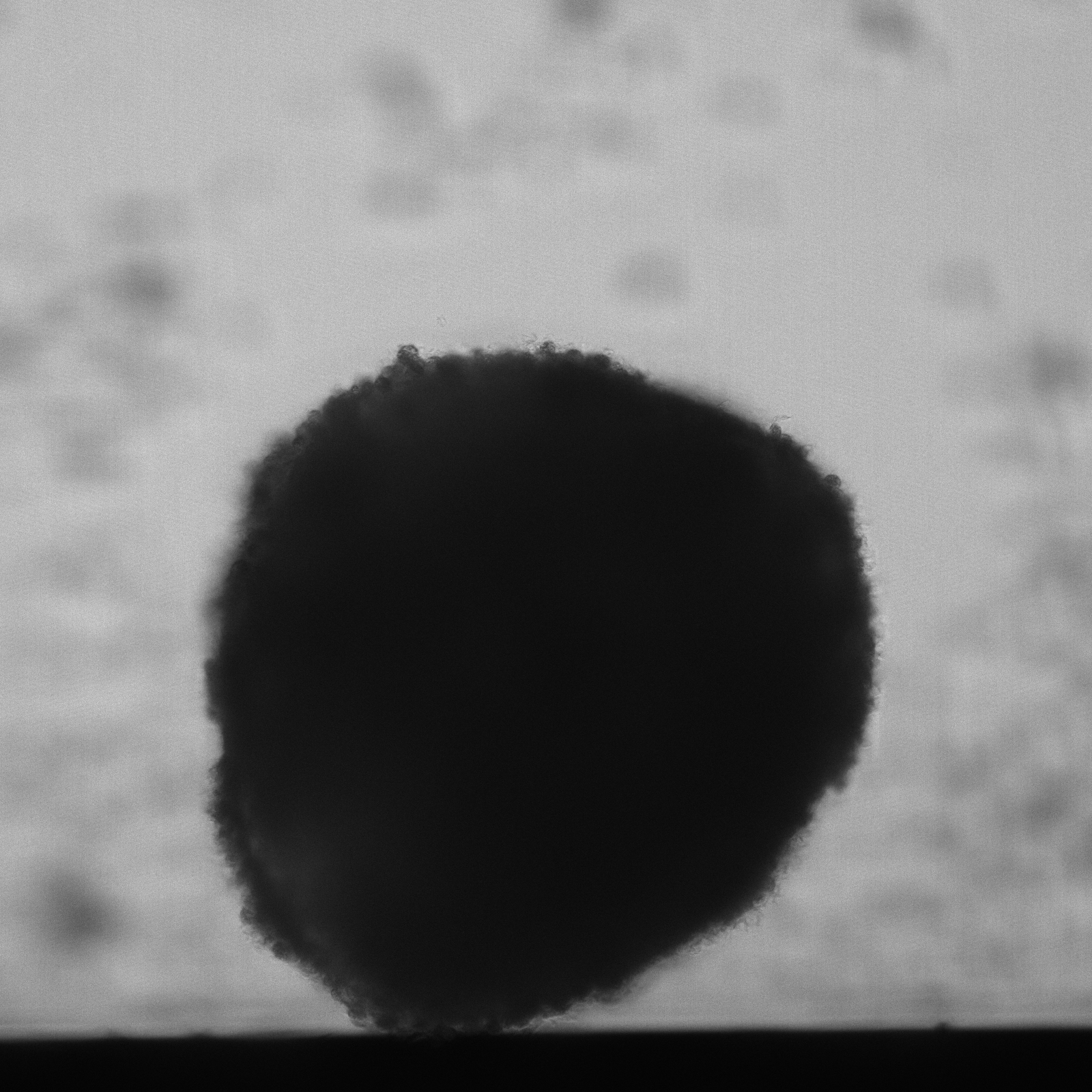

Supplement: Supplementary file 6 — Source Data for Figure 1 [file EMMM-15-e18199-s012.zip › Figure_1F,G,H/1F/Tumor_#19_D7.tif]

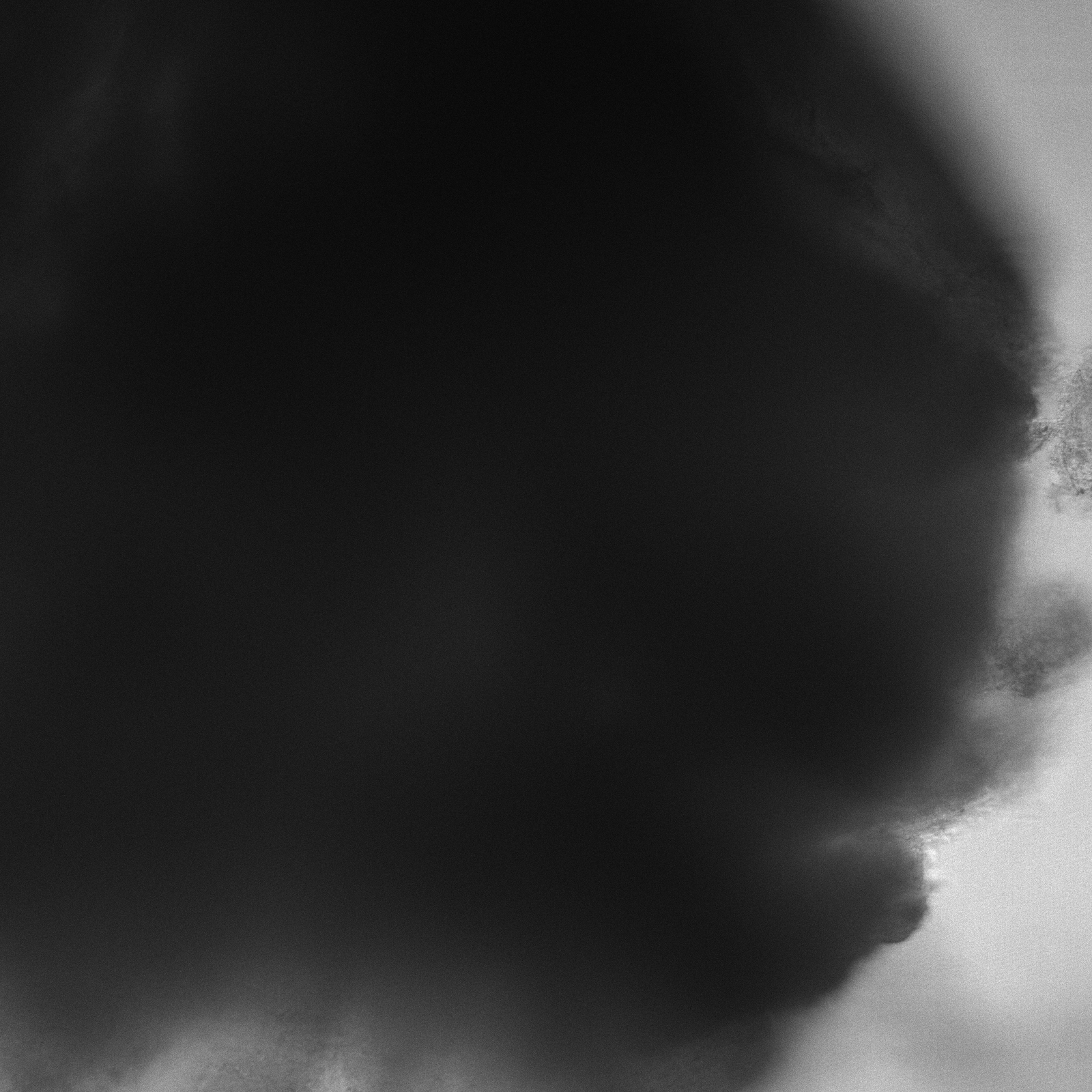

Supplement: Supplementary file 6 — Source Data for Figure 1 [file EMMM-15-e18199-s012.zip › Figure_1F,G,H/1F/Tumor_#20_D14.tif]

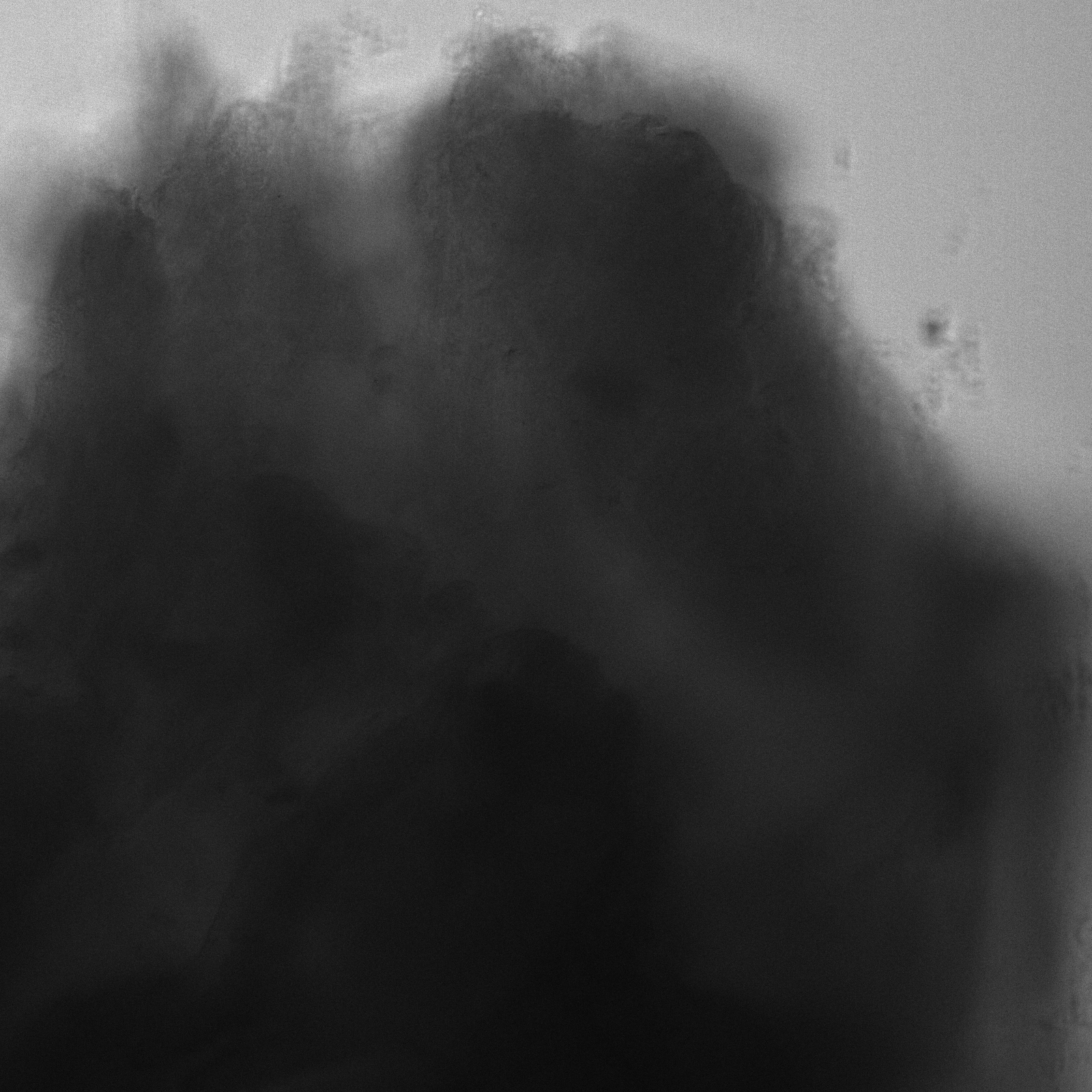

Supplement: Supplementary file 6 — Source Data for Figure 1 [file EMMM-15-e18199-s012.zip › Figure_1F,G,H/1F/Tumor_#20_D21.tif]

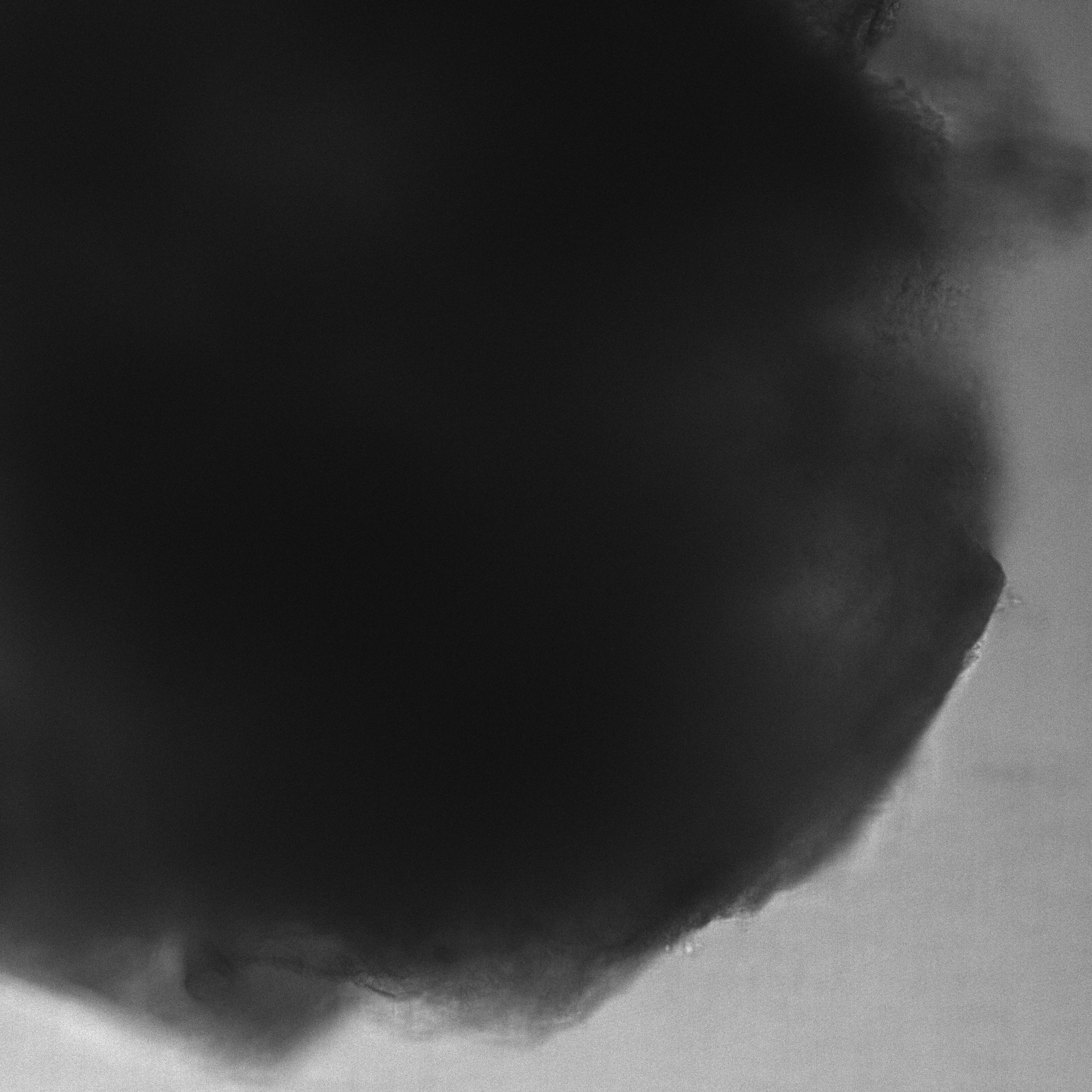

Supplement: Supplementary file 6 — Source Data for Figure 1 [file EMMM-15-e18199-s012.zip › Figure_1F,G,H/1F/Tumor_#20_D28.tif]

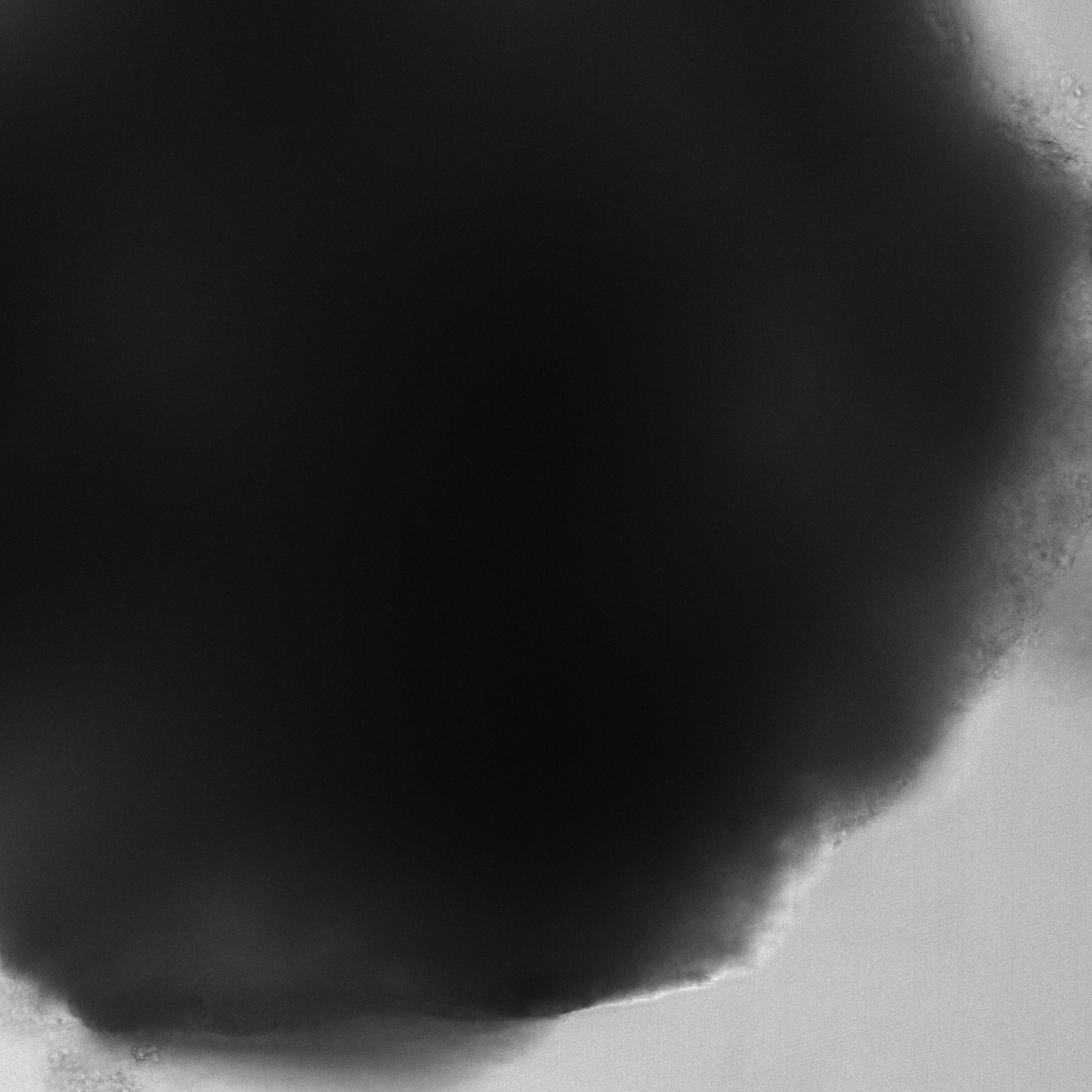

Supplement: Supplementary file 6 — Source Data for Figure 1 [file EMMM-15-e18199-s012.zip › Figure_1F,G,H/1F/Tumor_#20_D7.tif]

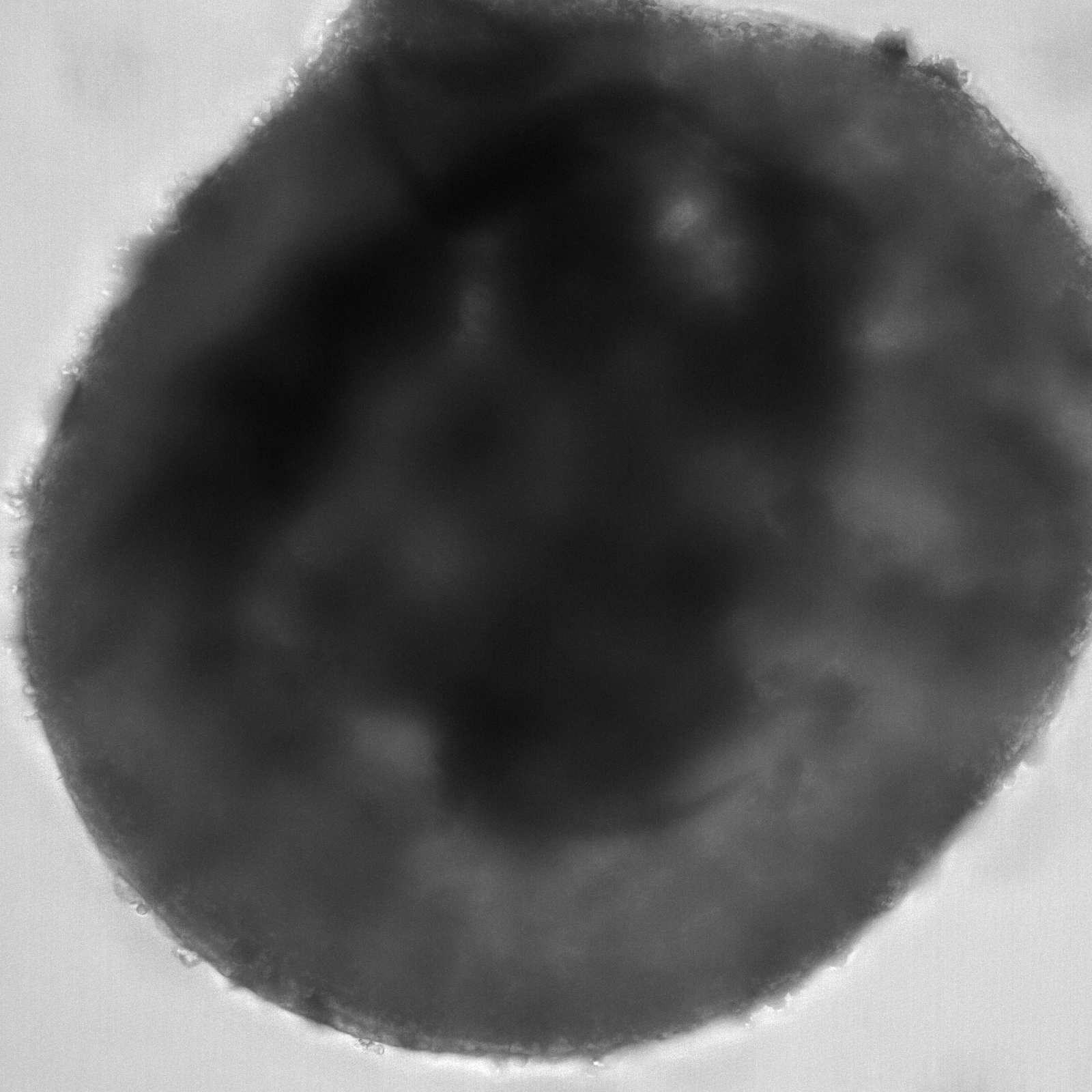

Supplement: Supplementary file 6 — Source Data for Figure 1 [file EMMM-15-e18199-s012.zip › Figure_1F,G,H/1F/Tumor_#21_D14.tif]
