# Supplementary figures and images for: Patient‐ and xenograft‐derived organoids recapitulate pediatric brain tumor features and patient treatments (part 3 of 4)
Source: EMBO Mol Med. 2023 Nov 30;15(12):e18199. doi: 10.15252/emmm.202318199 (PMC10701620; doi:10.15252/emmm.202318199)

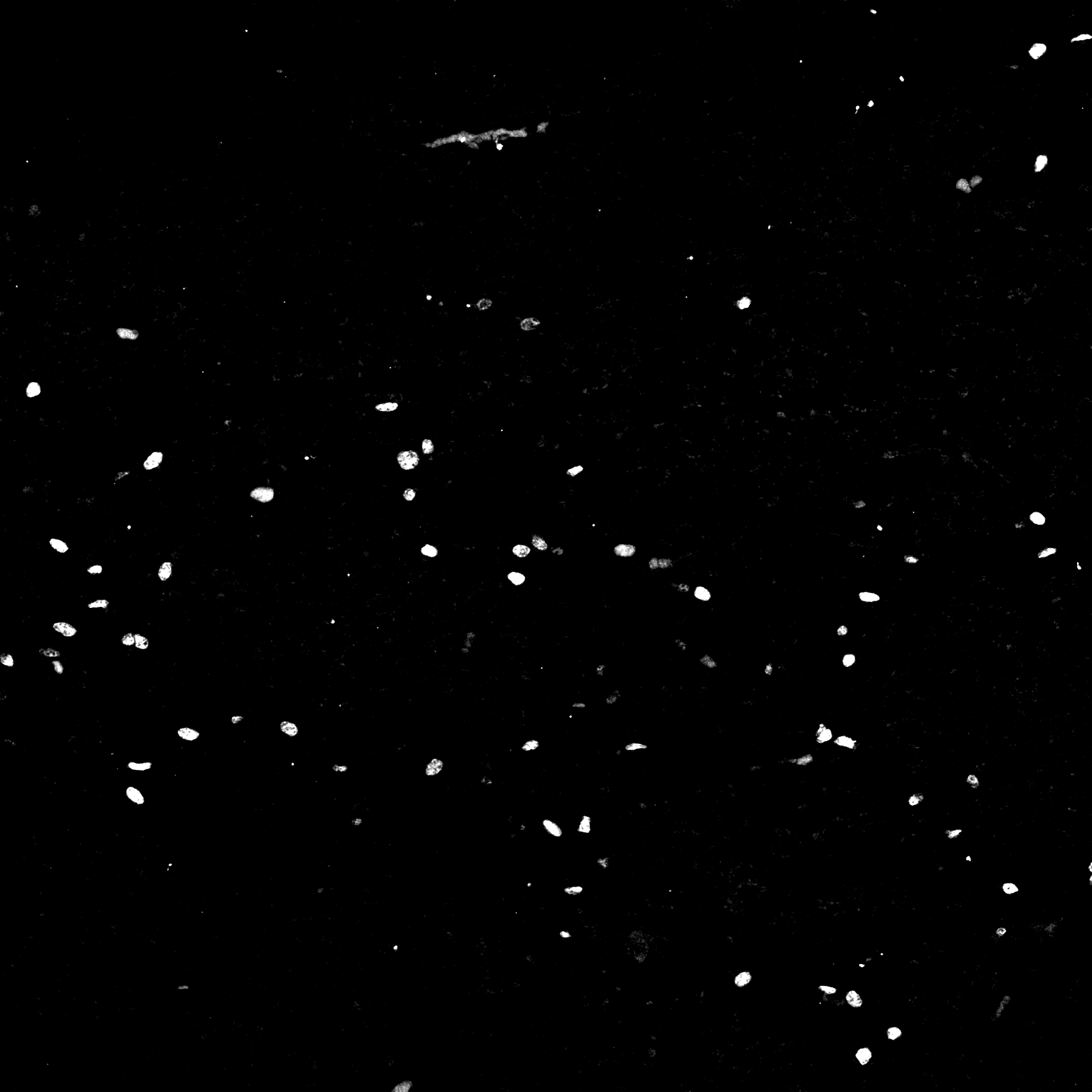

Supplement: Supplementary file 8 — Source Data for Figure 3 [file EMMM-15-e18199-s011.zip › Figure_3/3C/C'_Primary_T#9_OLIG2,_Nestin_OLIG2.tif]

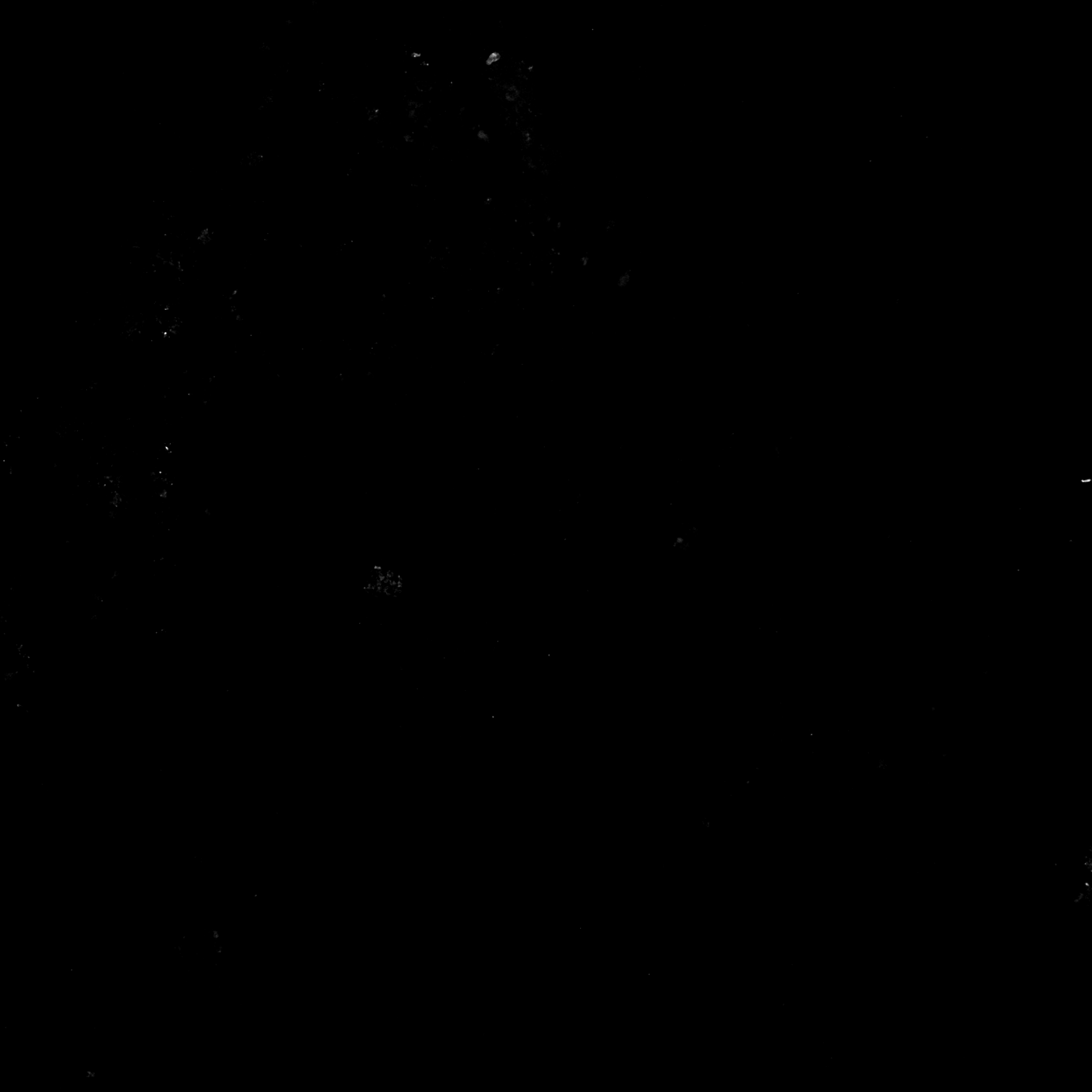

Supplement: Supplementary file 8 — Source Data for Figure 3 [file EMMM-15-e18199-s011.zip › Figure_3/3E/E'_PDO_T#21_D28_CD34,_CD3_CD3.tif]

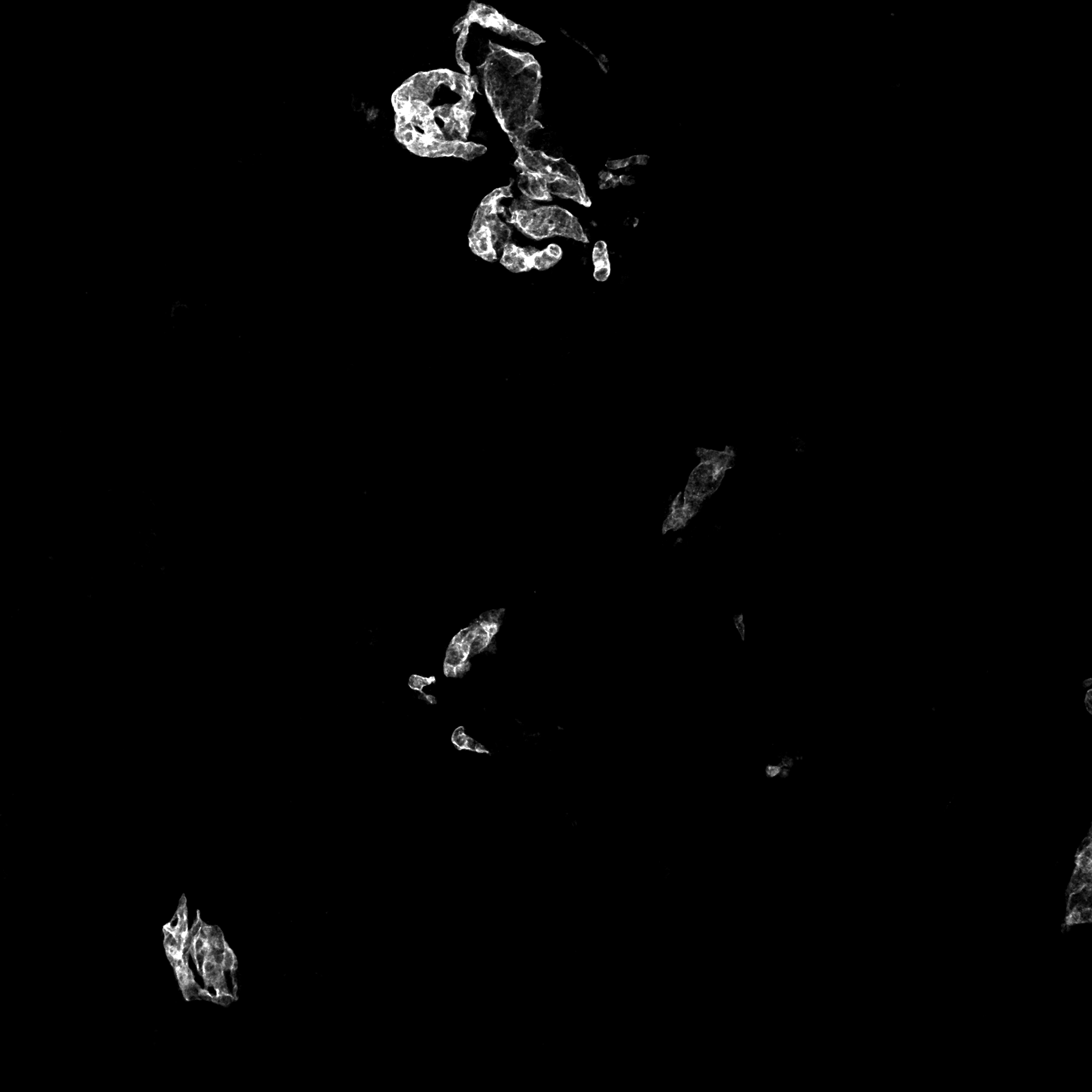

Supplement: Supplementary file 8 — Source Data for Figure 3 [file EMMM-15-e18199-s011.zip › Figure_3/3E/E'_PDO_T#21_D28_CD34,_CD3_CD34.tif]

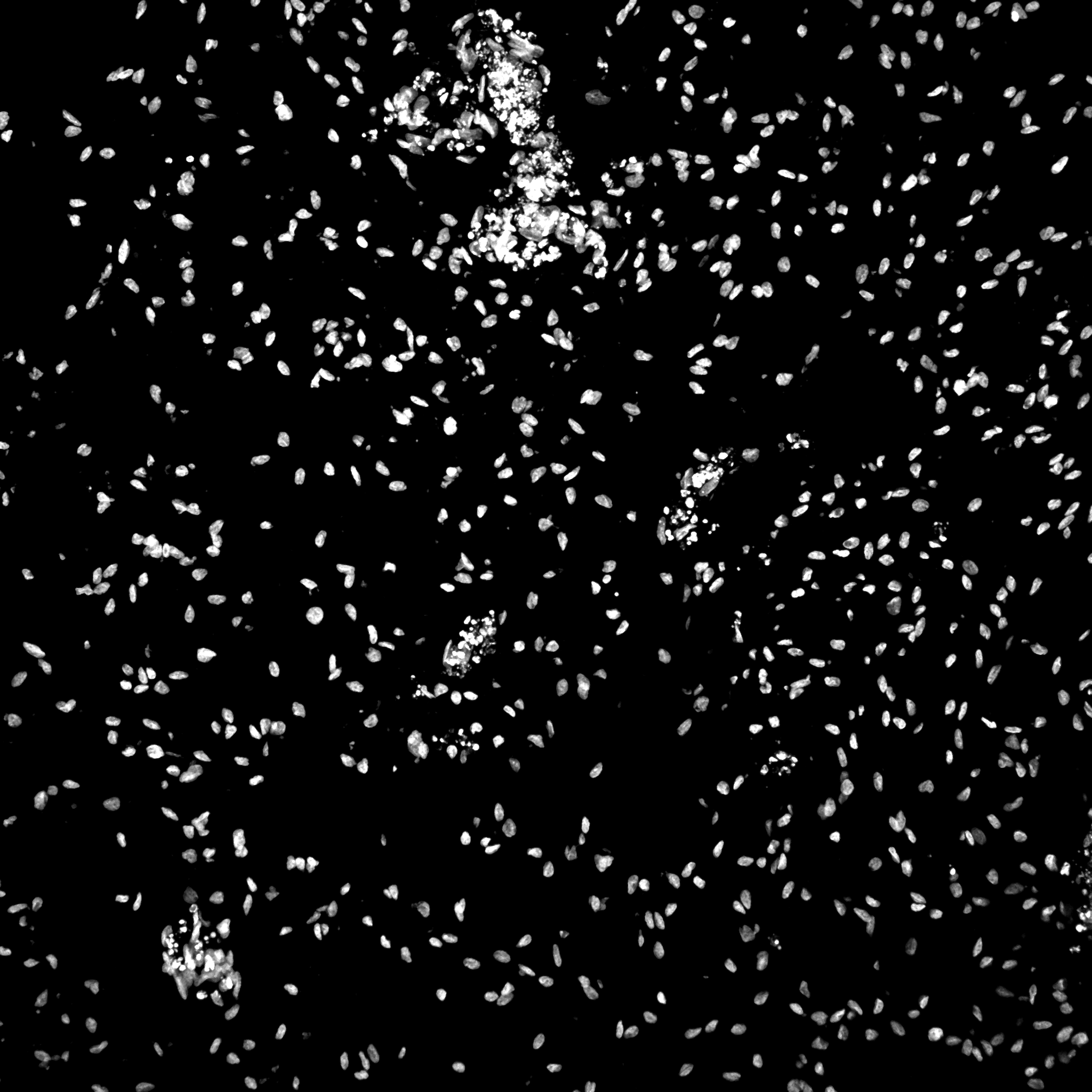

Supplement: Supplementary file 8 — Source Data for Figure 3 [file EMMM-15-e18199-s011.zip › Figure_3/3E/E'_PDO_T#21_D28_CD34,_CD3_DAPI.tif]

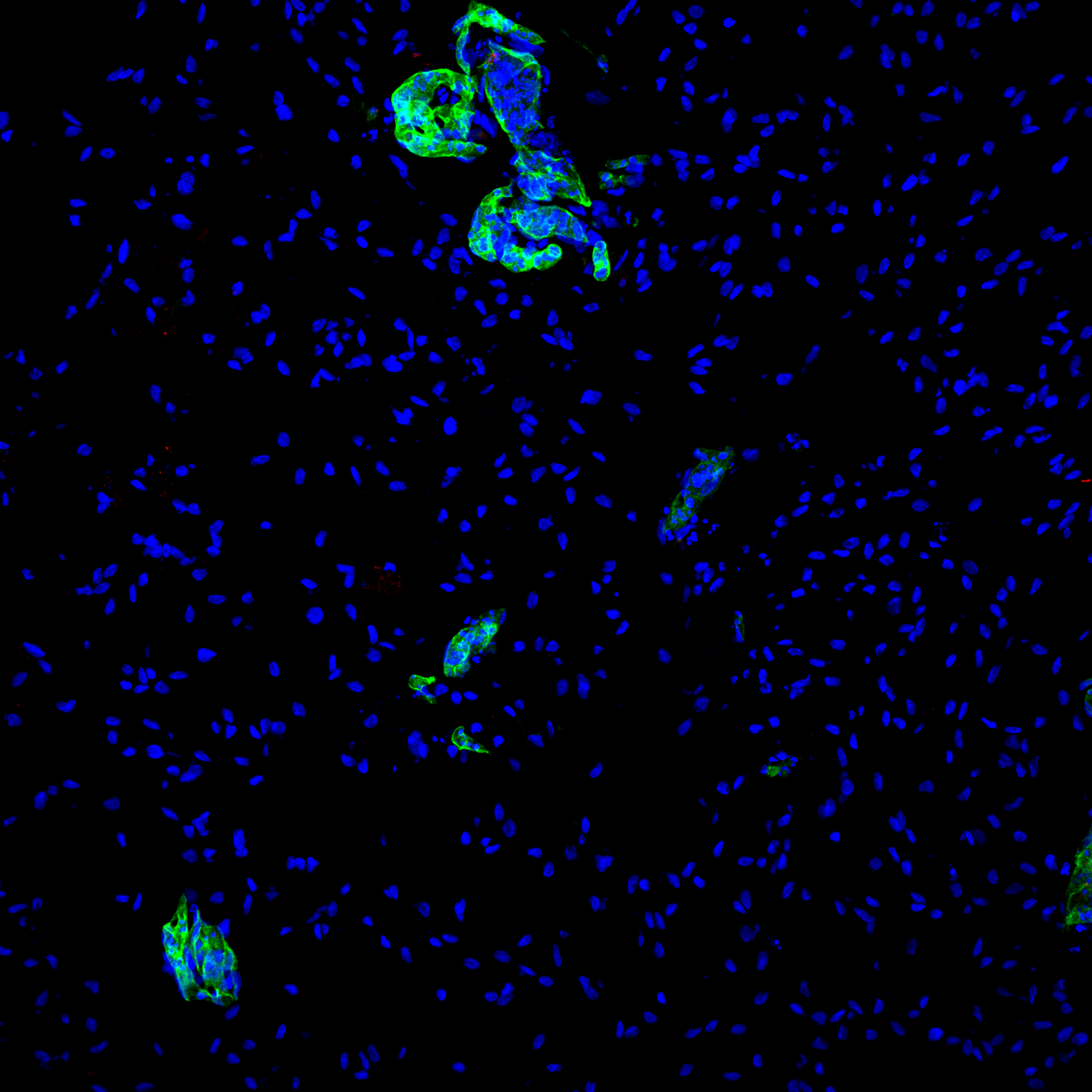

Supplement: Supplementary file 8 — Source Data for Figure 3 [file EMMM-15-e18199-s011.zip › Figure_3/3E/E'_PDO_T#21_D28_CD34,_CD3_merge.tif]

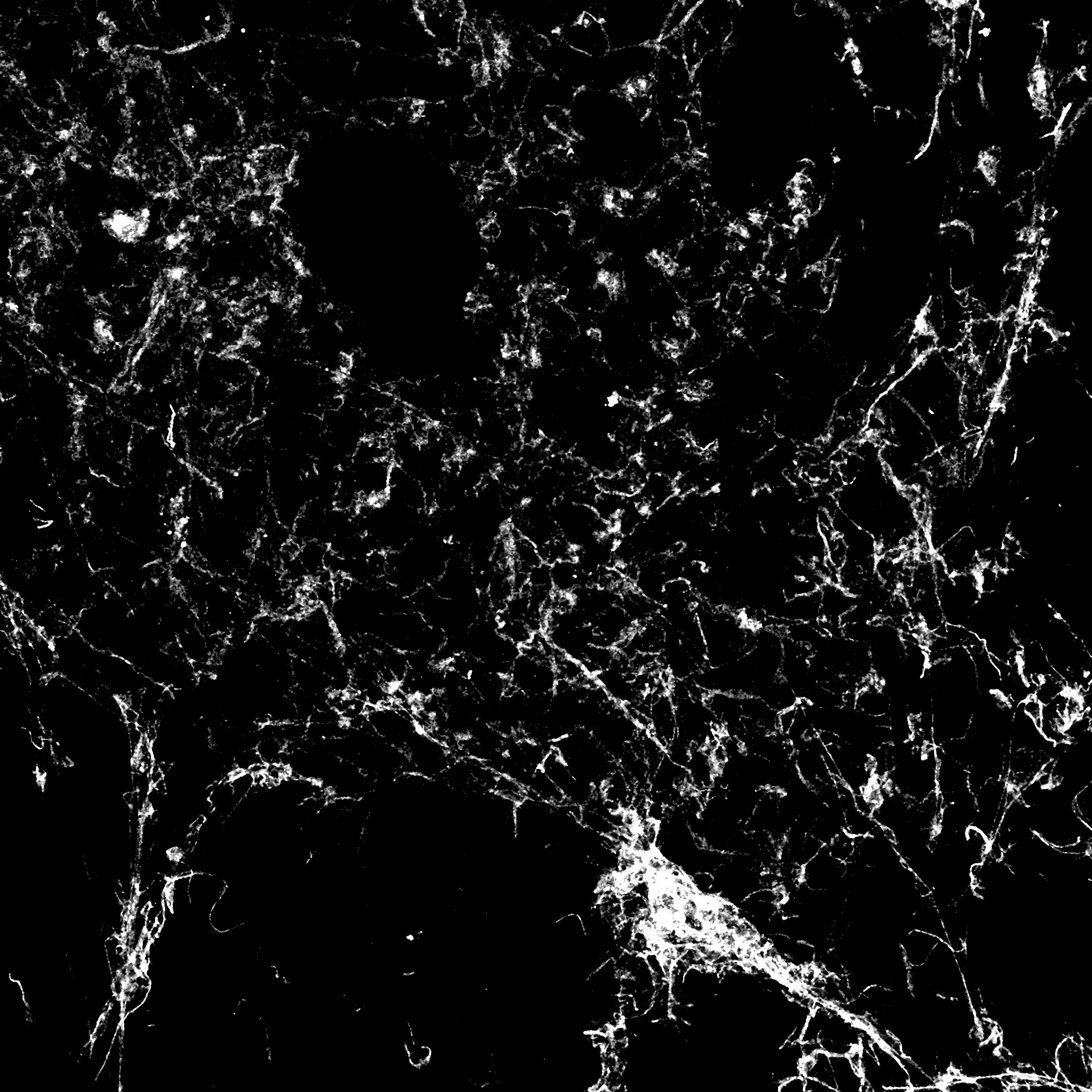

Supplement: Supplementary file 8 — Source Data for Figure 3 [file EMMM-15-e18199-s011.zip › Figure_3/3E/E'_PDO_T#21_D28_GFAP,_B3tubulin_B3tubulin.tif]

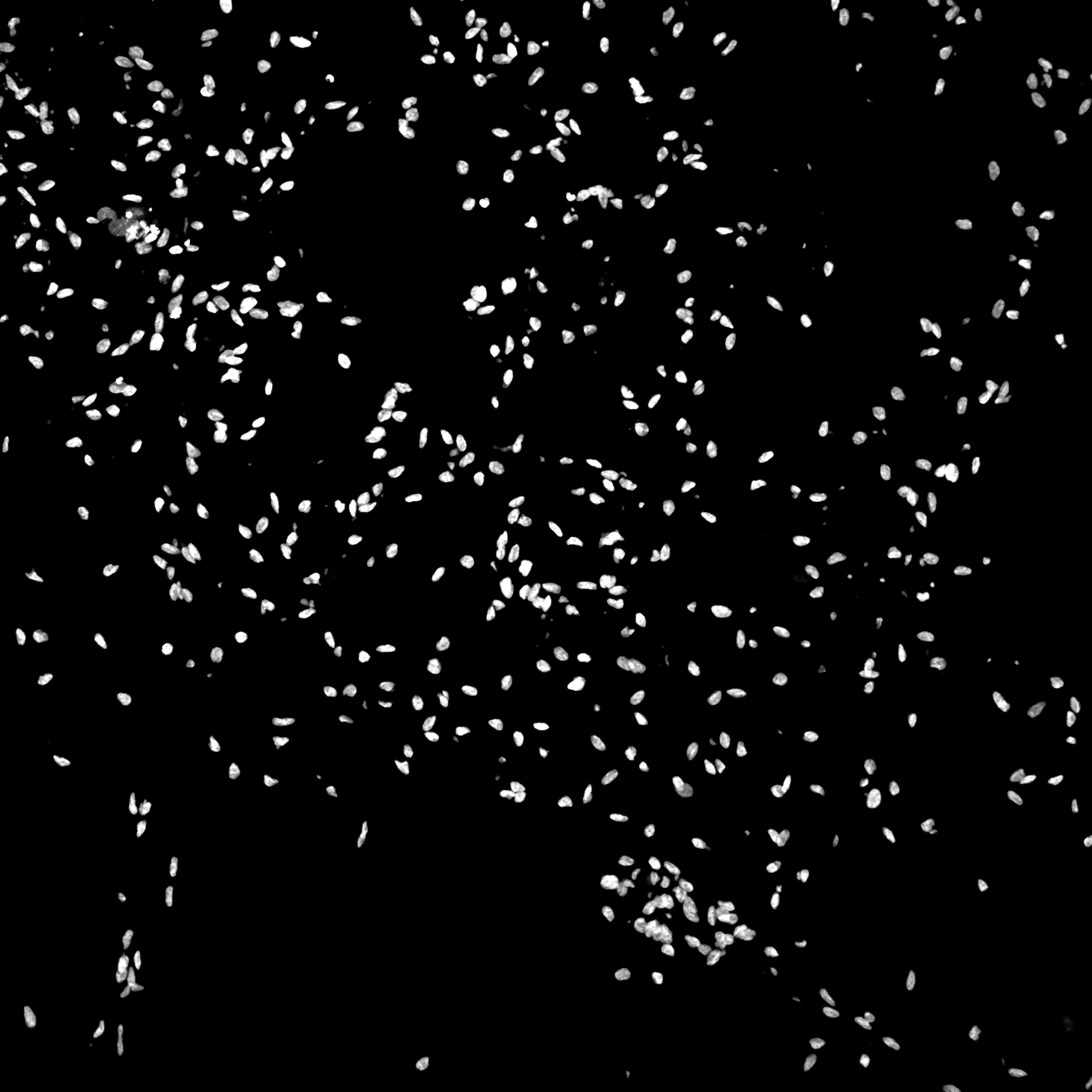

Supplement: Supplementary file 8 — Source Data for Figure 3 [file EMMM-15-e18199-s011.zip › Figure_3/3E/E'_PDO_T#21_D28_GFAP,_B3tubulin_DAPI.tif]

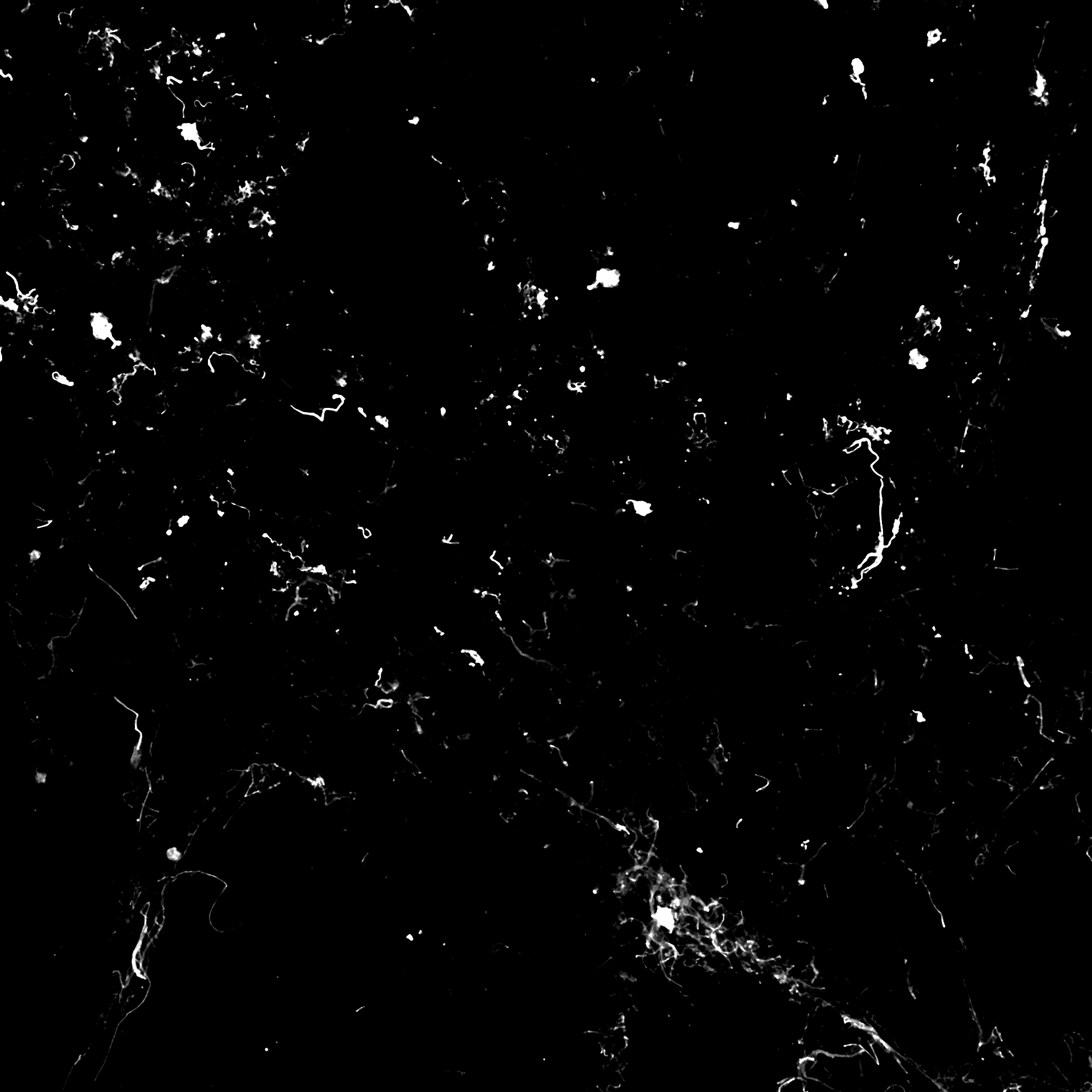

Supplement: Supplementary file 8 — Source Data for Figure 3 [file EMMM-15-e18199-s011.zip › Figure_3/3E/E'_PDO_T#21_D28_GFAP,_B3tubulin_GFAP.tif]

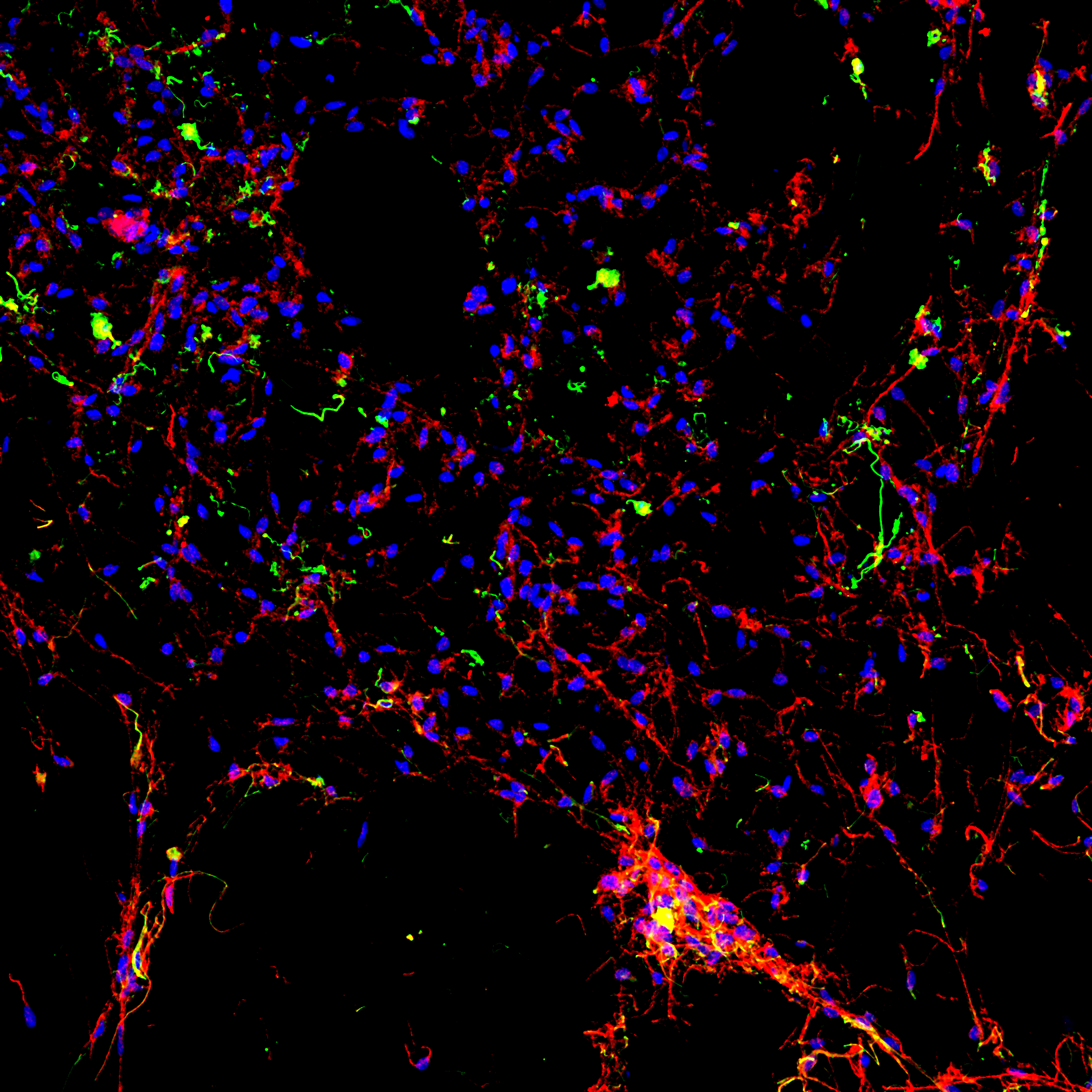

Supplement: Supplementary file 8 — Source Data for Figure 3 [file EMMM-15-e18199-s011.zip › Figure_3/3E/E'_PDO_T#21_D28_GFAP,_B3tubulin_merge.tif]

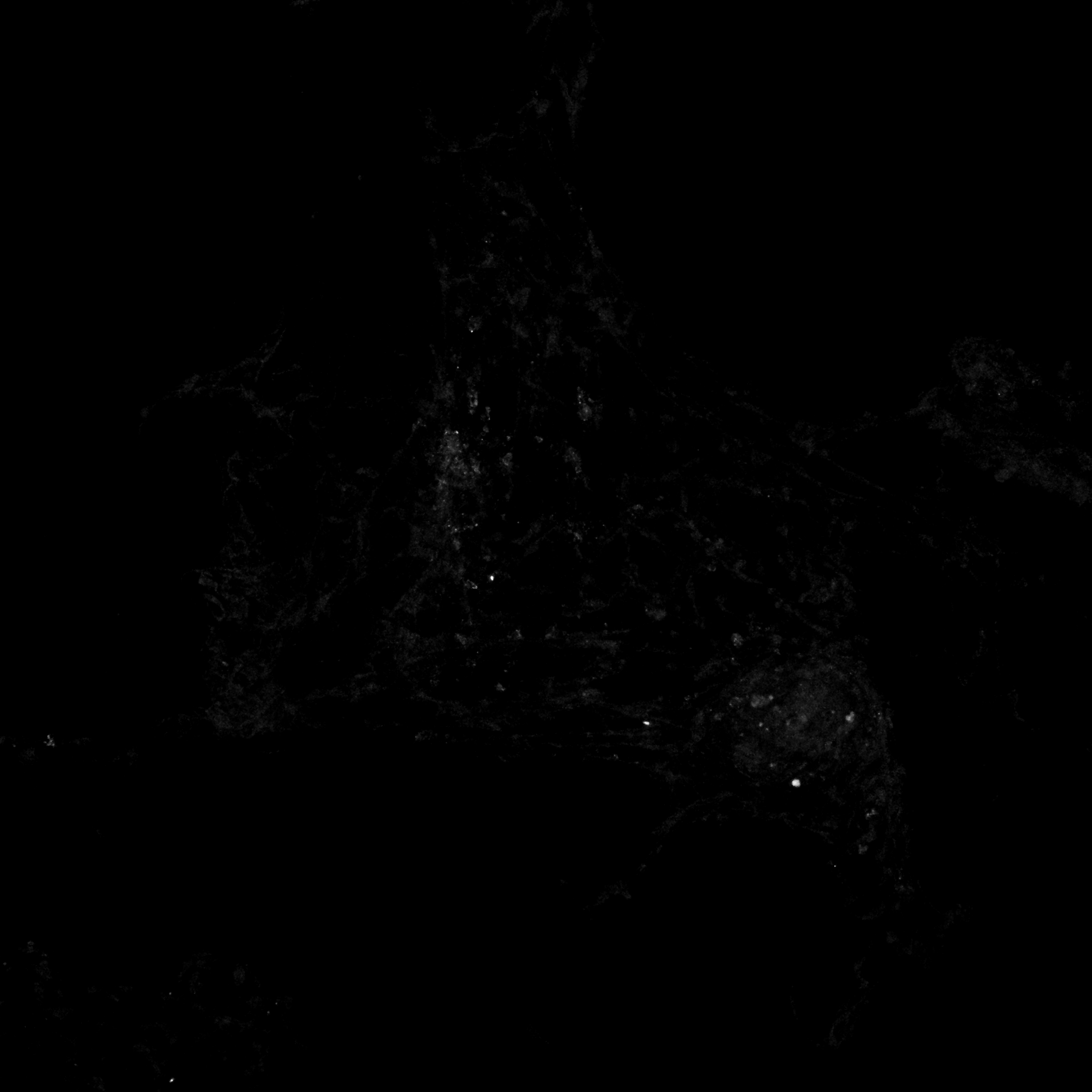

Supplement: Supplementary file 8 — Source Data for Figure 3 [file EMMM-15-e18199-s011.zip › Figure_3/3E/E'_PDO_T#21_D28_IBA1,_CD3_CD3.tif]

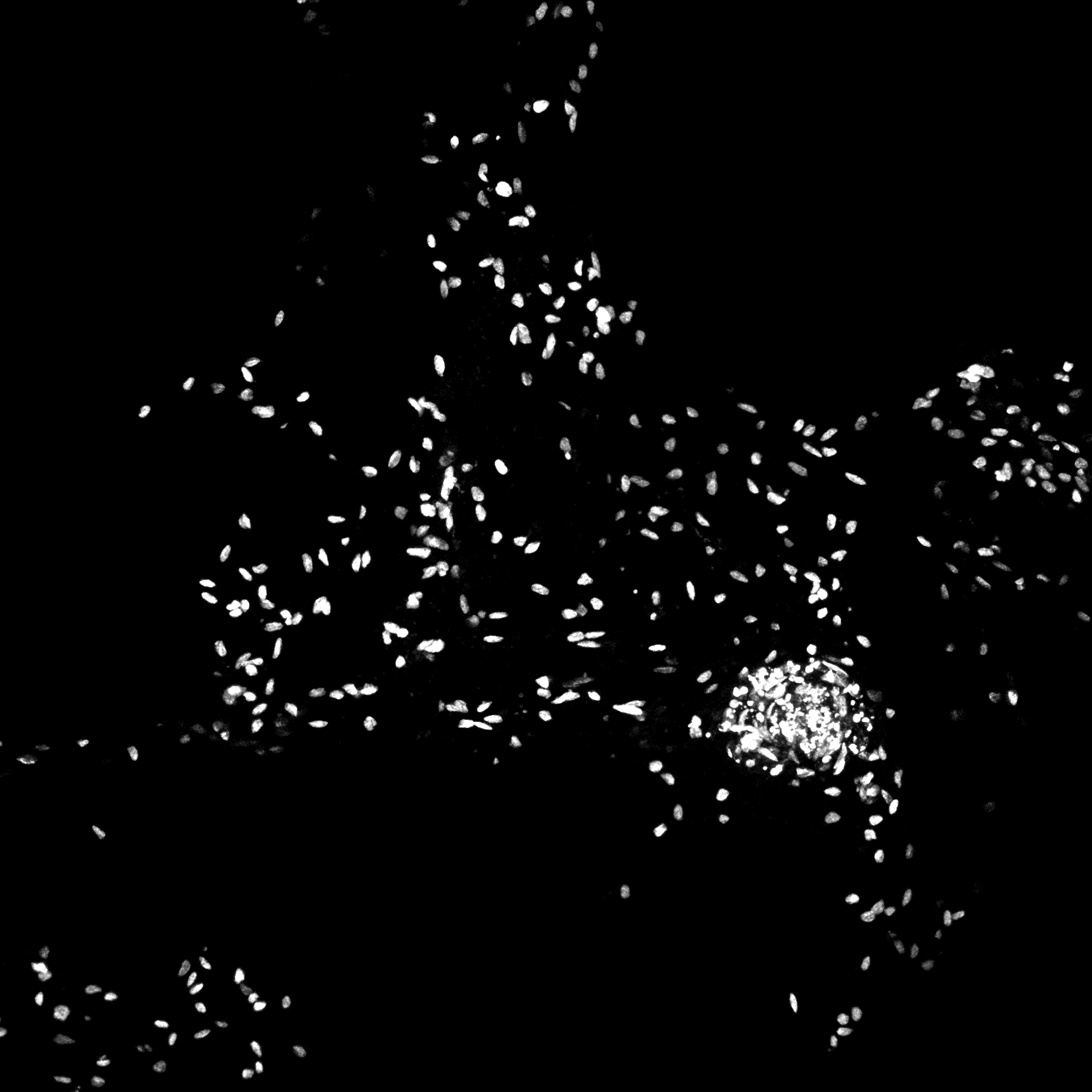

Supplement: Supplementary file 8 — Source Data for Figure 3 [file EMMM-15-e18199-s011.zip › Figure_3/3E/E'_PDO_T#21_D28_IBA1,_CD3_DAPI.tif]

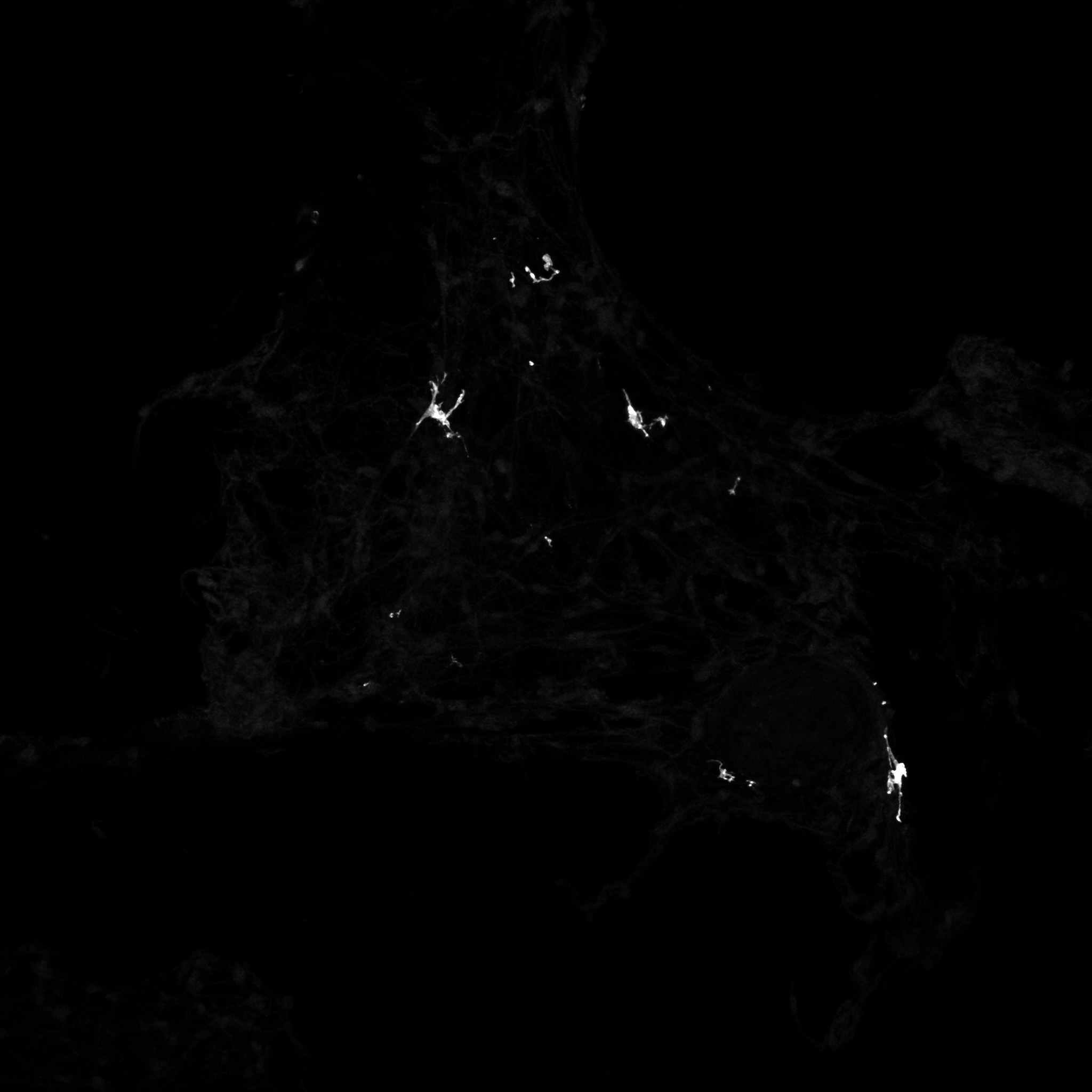

Supplement: Supplementary file 8 — Source Data for Figure 3 [file EMMM-15-e18199-s011.zip › Figure_3/3E/E'_PDO_T#21_D28_IBA1,_CD3_IBA1.tif]

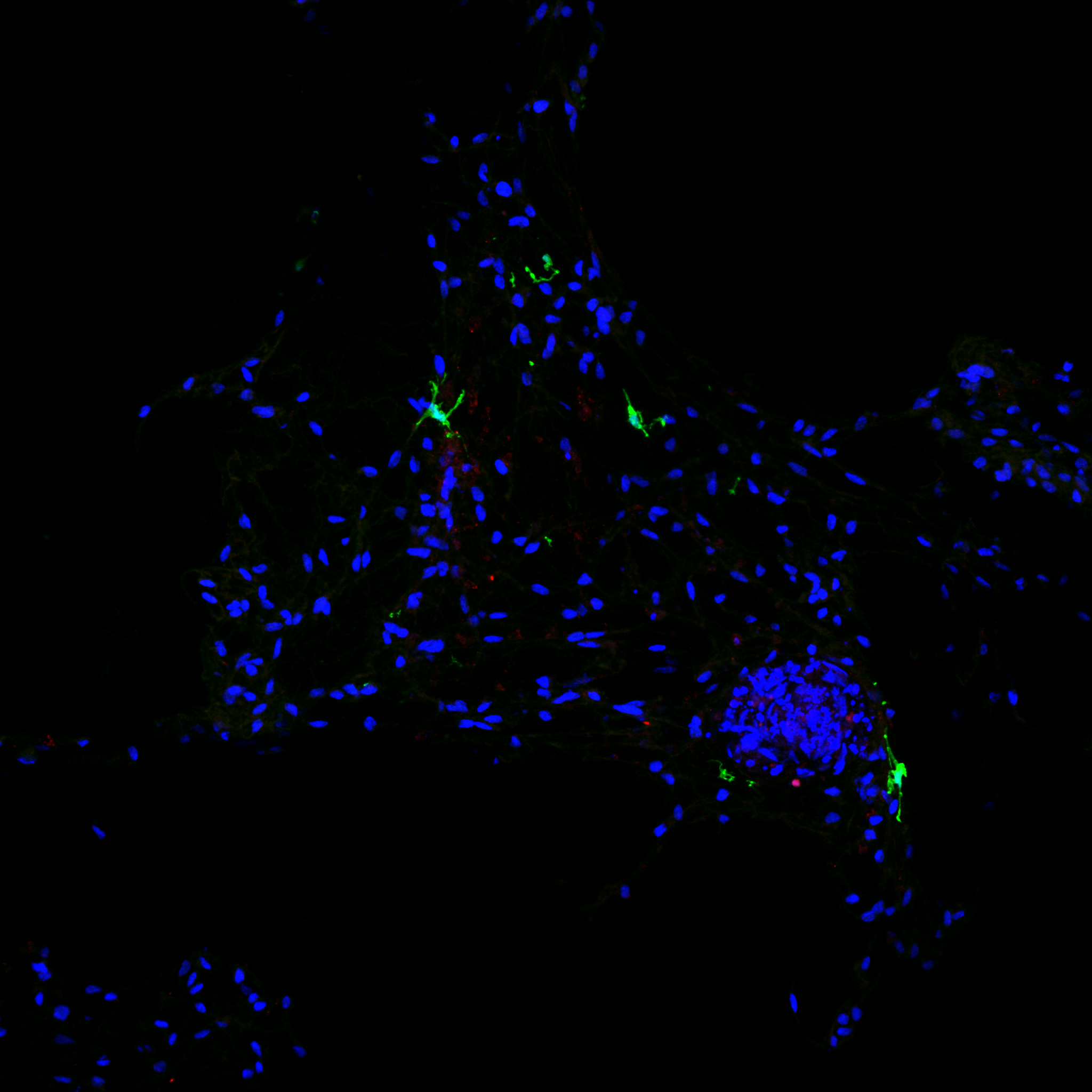

Supplement: Supplementary file 8 — Source Data for Figure 3 [file EMMM-15-e18199-s011.zip › Figure_3/3E/E'_PDO_T#21_D28_IBA1,_CD3_merge.tif]

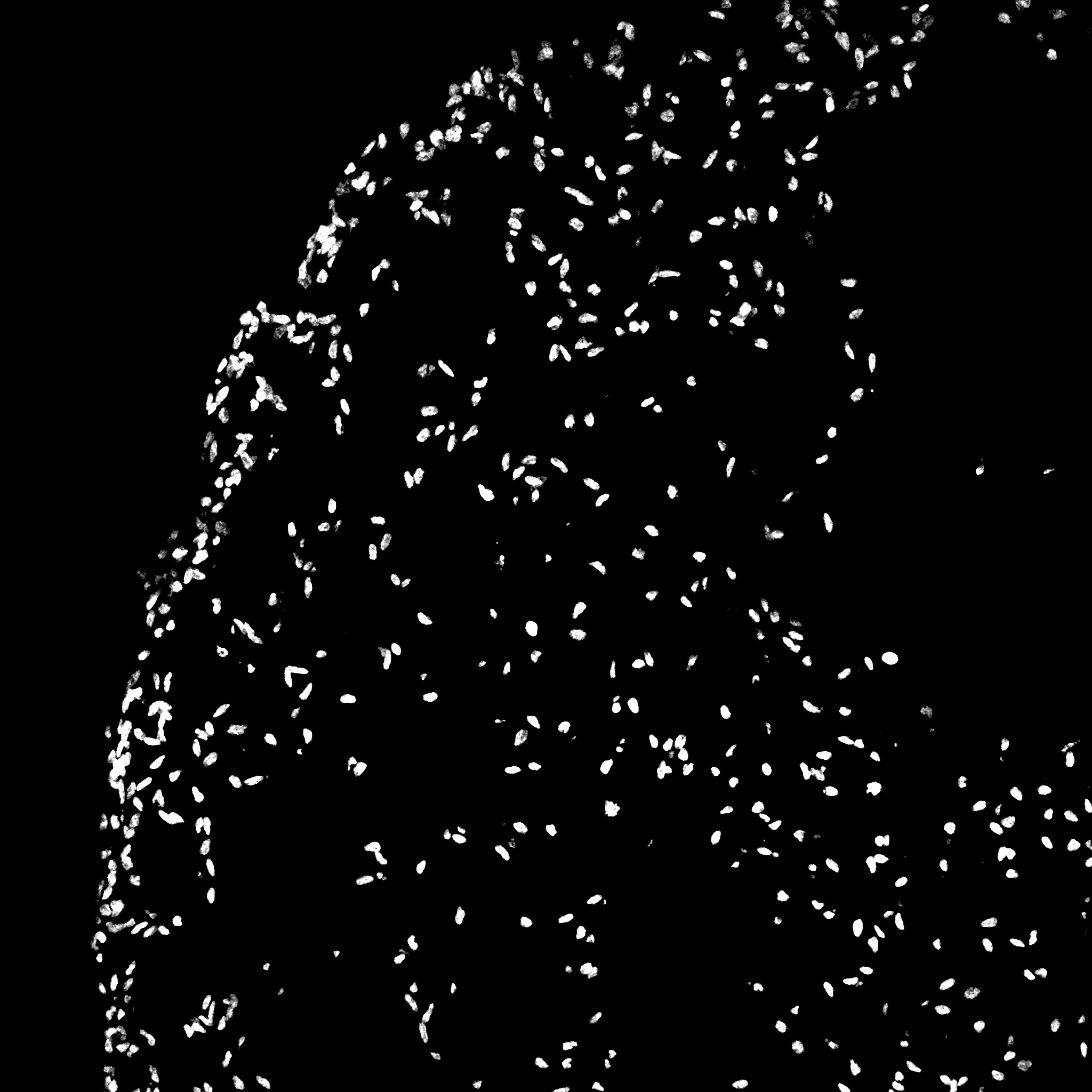

Supplement: Supplementary file 8 — Source Data for Figure 3 [file EMMM-15-e18199-s011.zip › Figure_3/3E/E'_PDO_T#21_D28_Ki67,_SOX2_DAPI.tif]

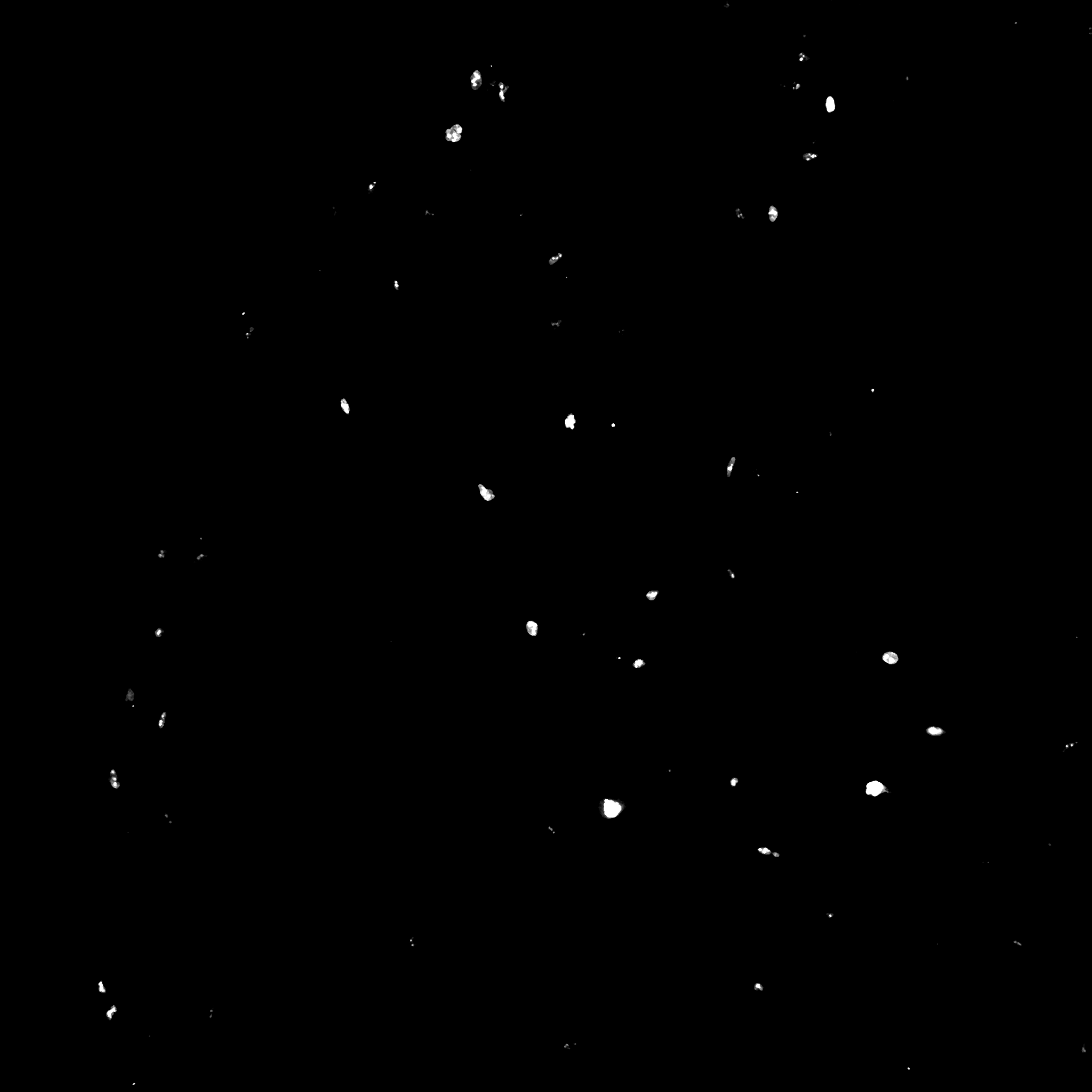

Supplement: Supplementary file 8 — Source Data for Figure 3 [file EMMM-15-e18199-s011.zip › Figure_3/3E/E'_PDO_T#21_D28_Ki67,_SOX2_Ki67.tif]

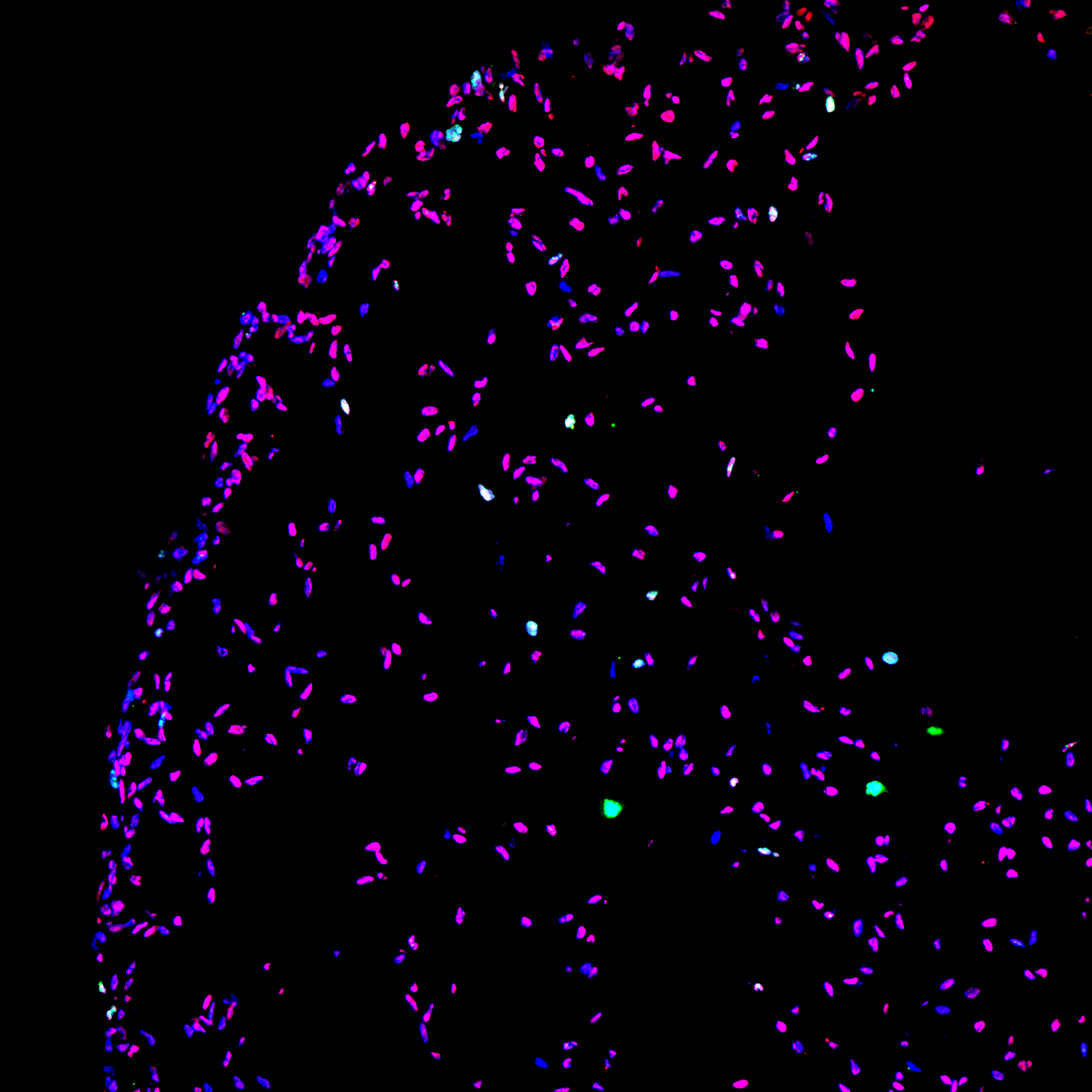

Supplement: Supplementary file 8 — Source Data for Figure 3 [file EMMM-15-e18199-s011.zip › Figure_3/3E/E'_PDO_T#21_D28_Ki67,_SOX2_merge.tif]

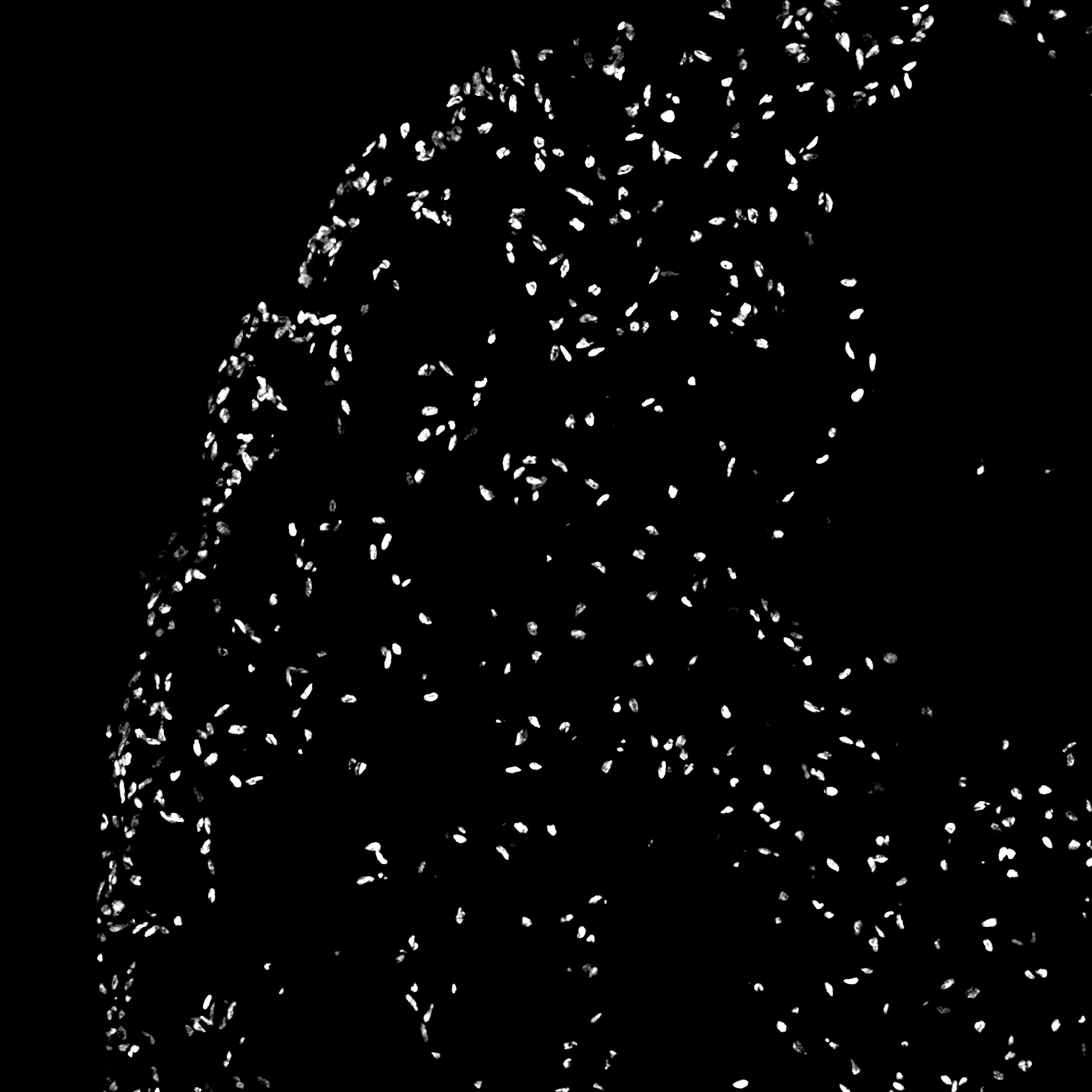

Supplement: Supplementary file 8 — Source Data for Figure 3 [file EMMM-15-e18199-s011.zip › Figure_3/3E/E'_PDO_T#21_D28_Ki67,_SOX2_SOX2.tif]

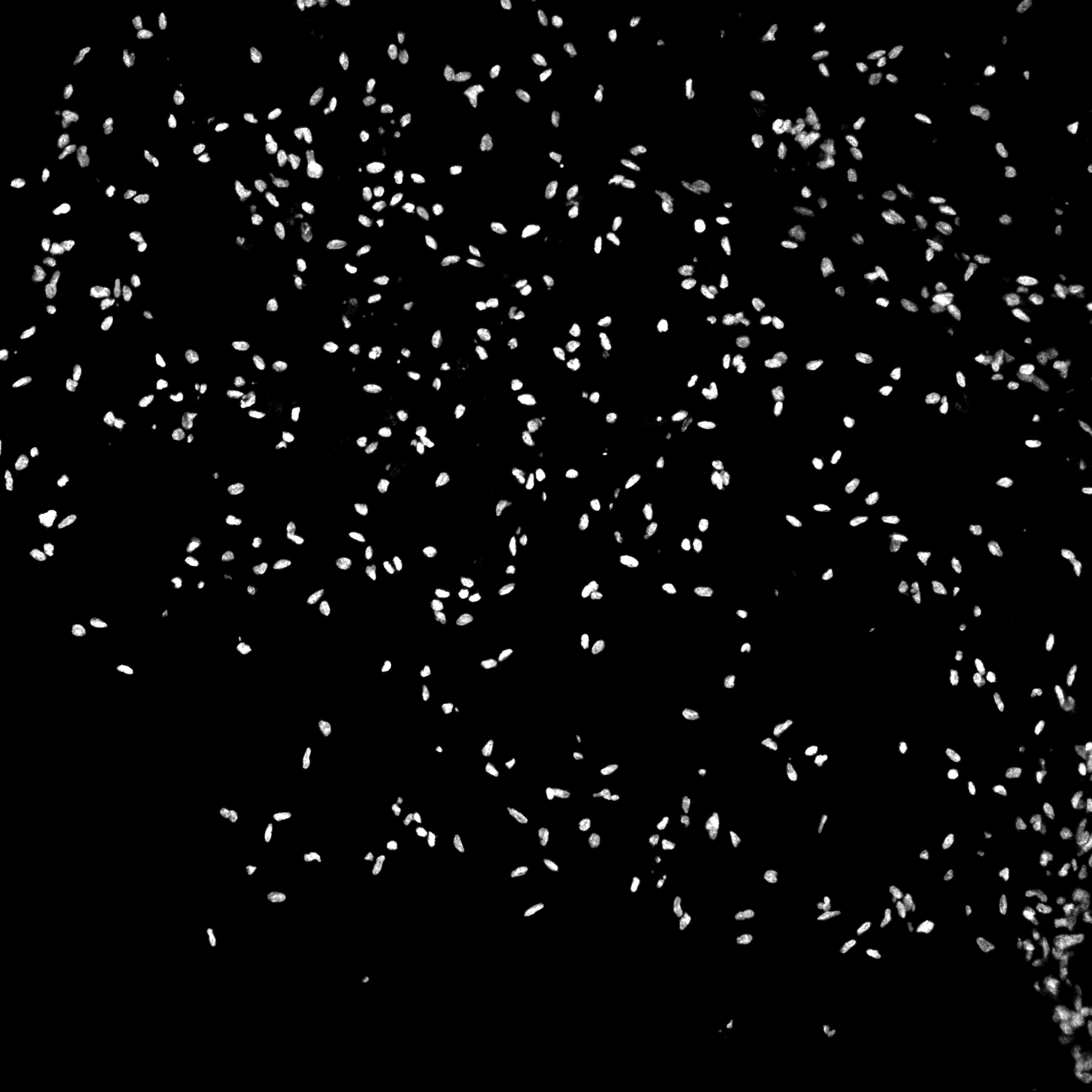

Supplement: Supplementary file 8 — Source Data for Figure 3 [file EMMM-15-e18199-s011.zip › Figure_3/3E/E'_PDO_T#21_D28_OLIG2,_Nestin_DAPI.tif]

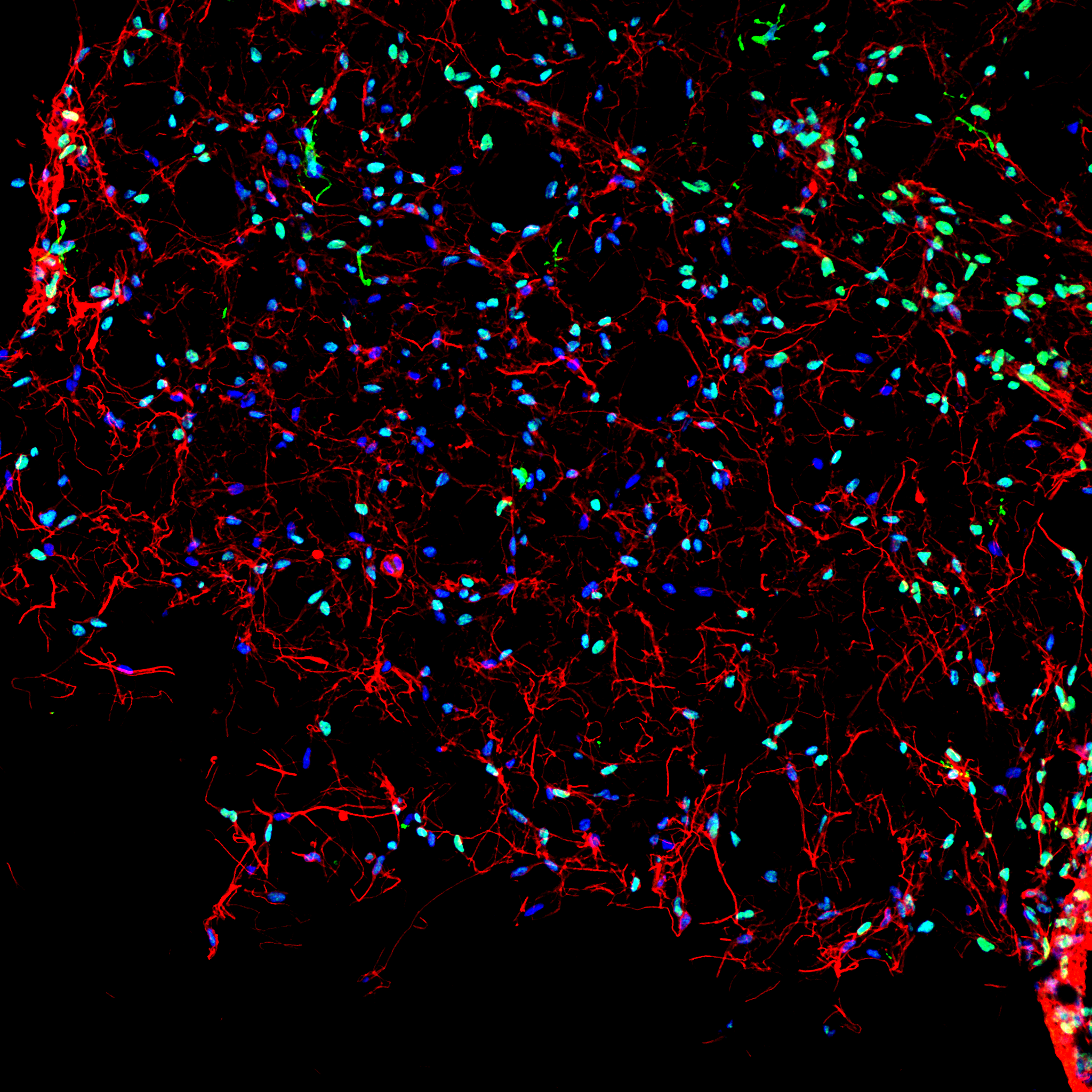

Supplement: Supplementary file 8 — Source Data for Figure 3 [file EMMM-15-e18199-s011.zip › Figure_3/3E/E'_PDO_T#21_D28_OLIG2,_Nestin_merge.tif]

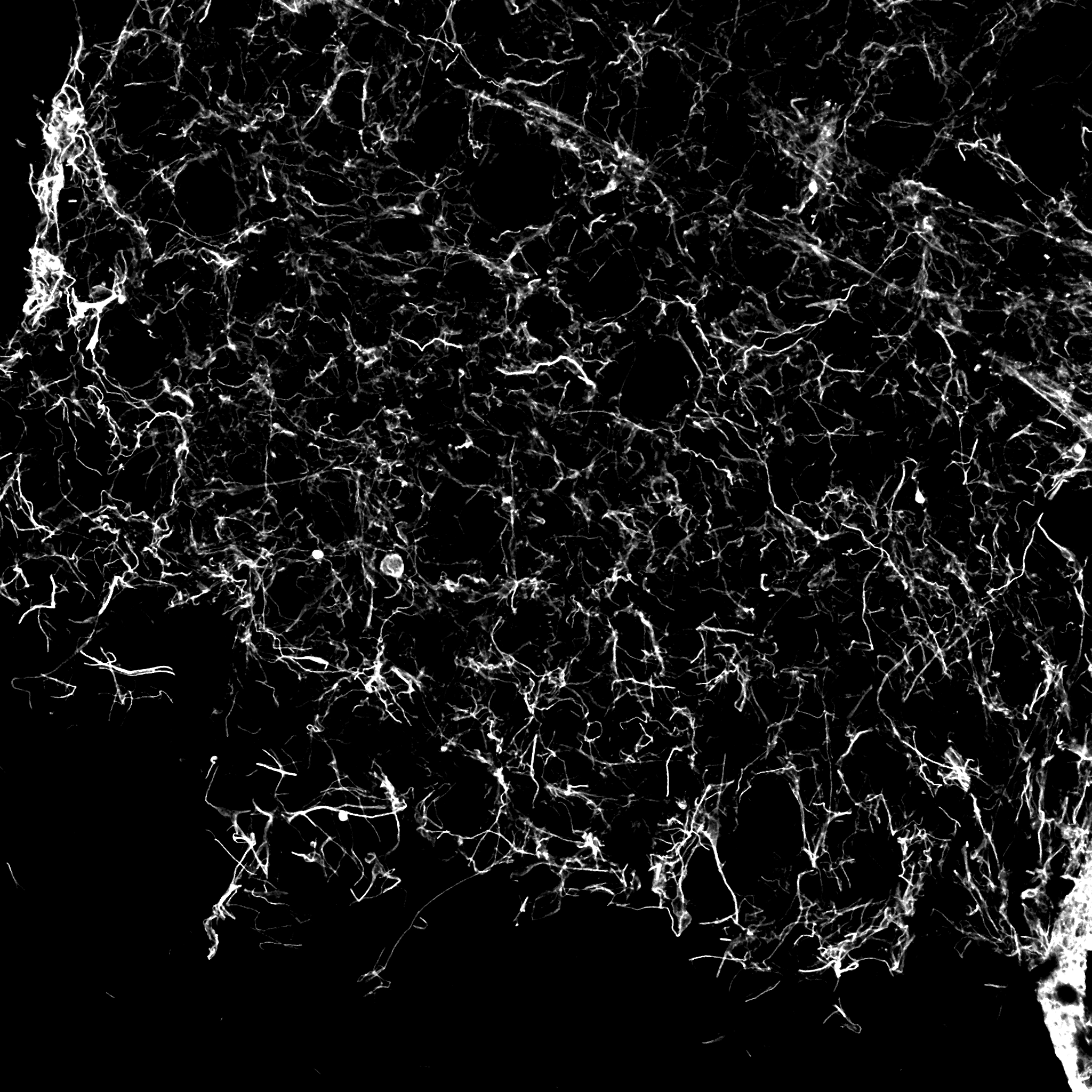

Supplement: Supplementary file 8 — Source Data for Figure 3 [file EMMM-15-e18199-s011.zip › Figure_3/3E/E'_PDO_T#21_D28_OLIG2,_Nestin_Nestin.tif]

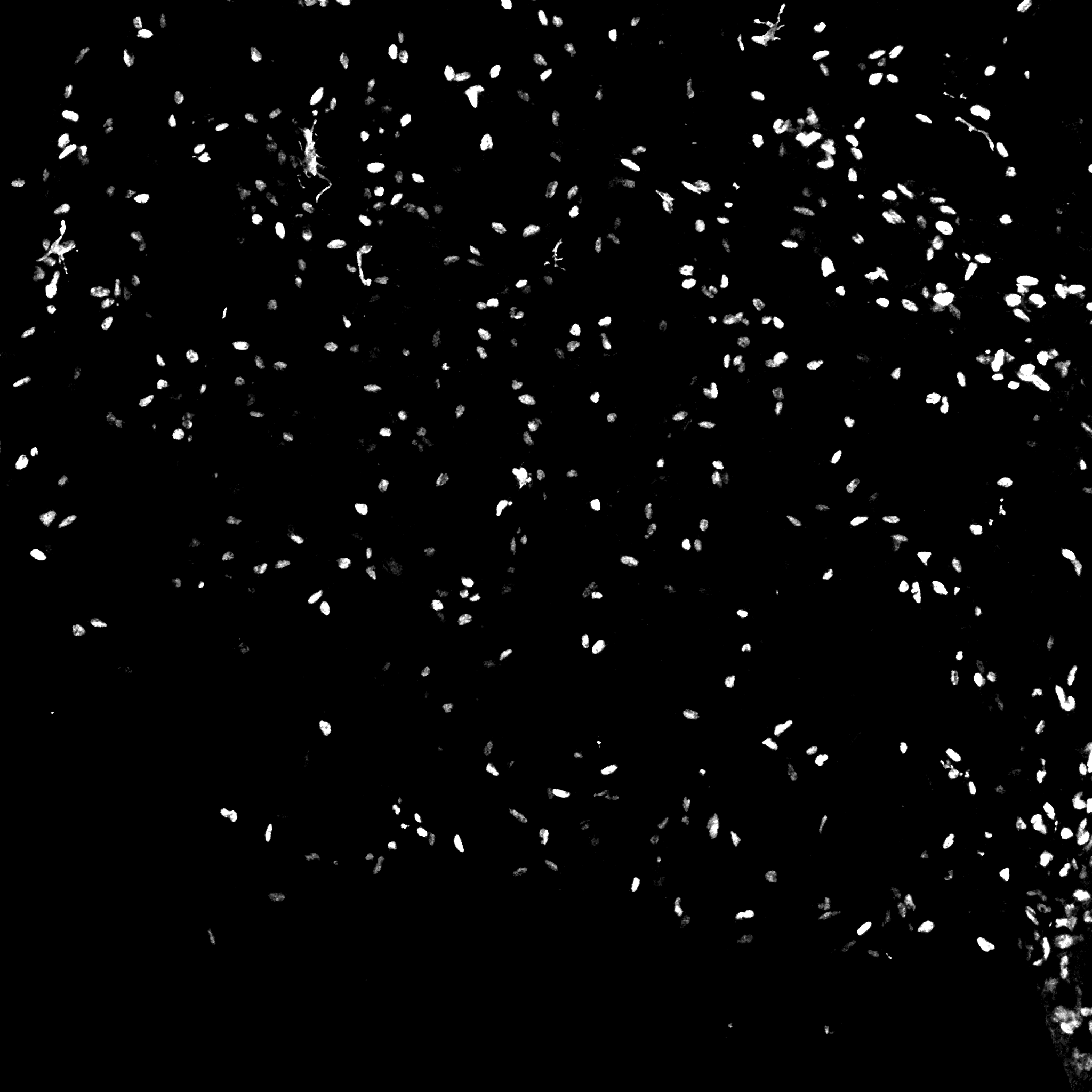

Supplement: Supplementary file 8 — Source Data for Figure 3 [file EMMM-15-e18199-s011.zip › Figure_3/3E/E'_PDO_T#21_D28_OLIG2,_Nestin_OLIG2.tif]

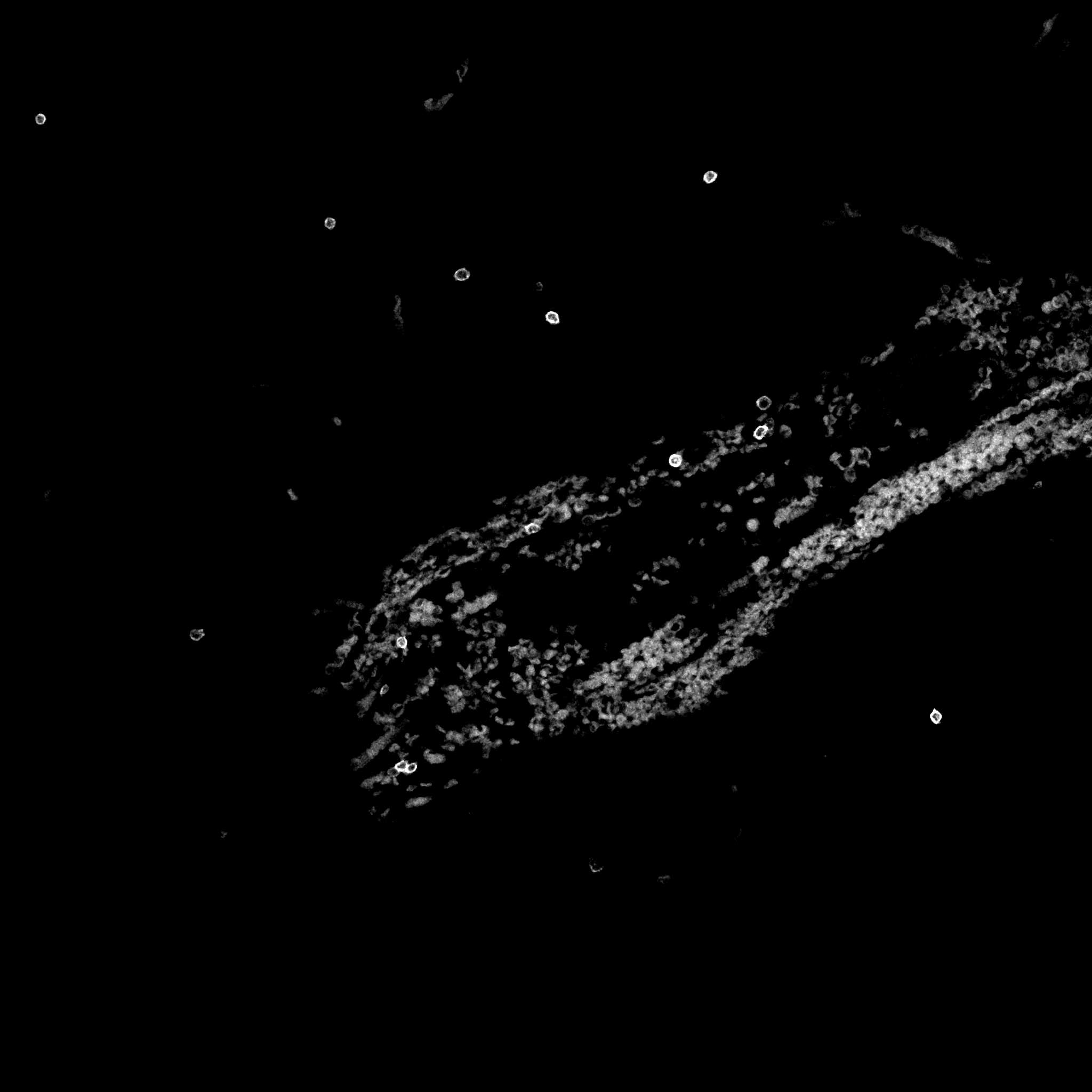

Supplement: Supplementary file 8 — Source Data for Figure 3 [file EMMM-15-e18199-s011.zip › Figure_3/3E/E'_Primary_T#21_CD34,_CD3_CD3.tif]

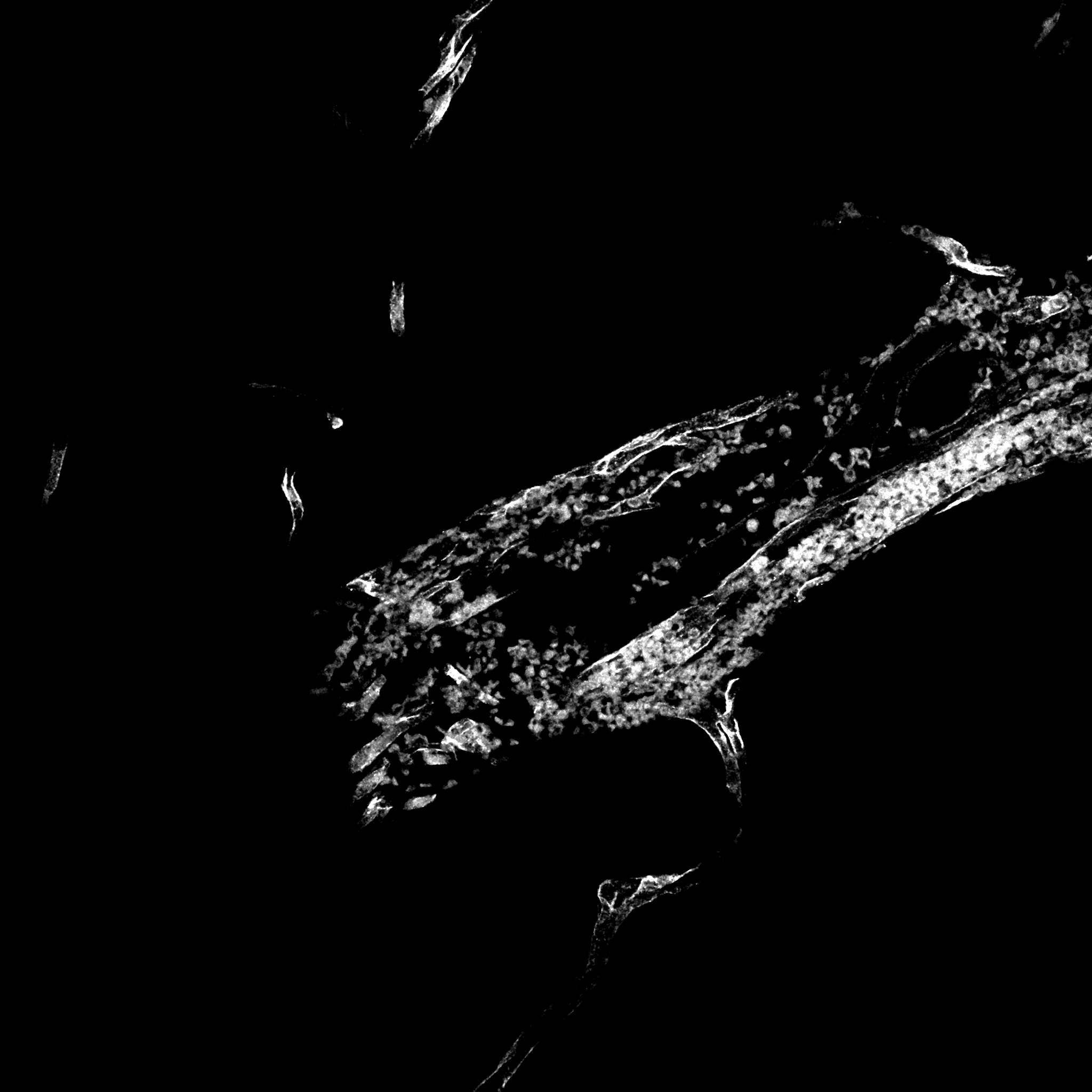

Supplement: Supplementary file 8 — Source Data for Figure 3 [file EMMM-15-e18199-s011.zip › Figure_3/3E/E'_Primary_T#21_CD34,_CD3_CD34.tif]

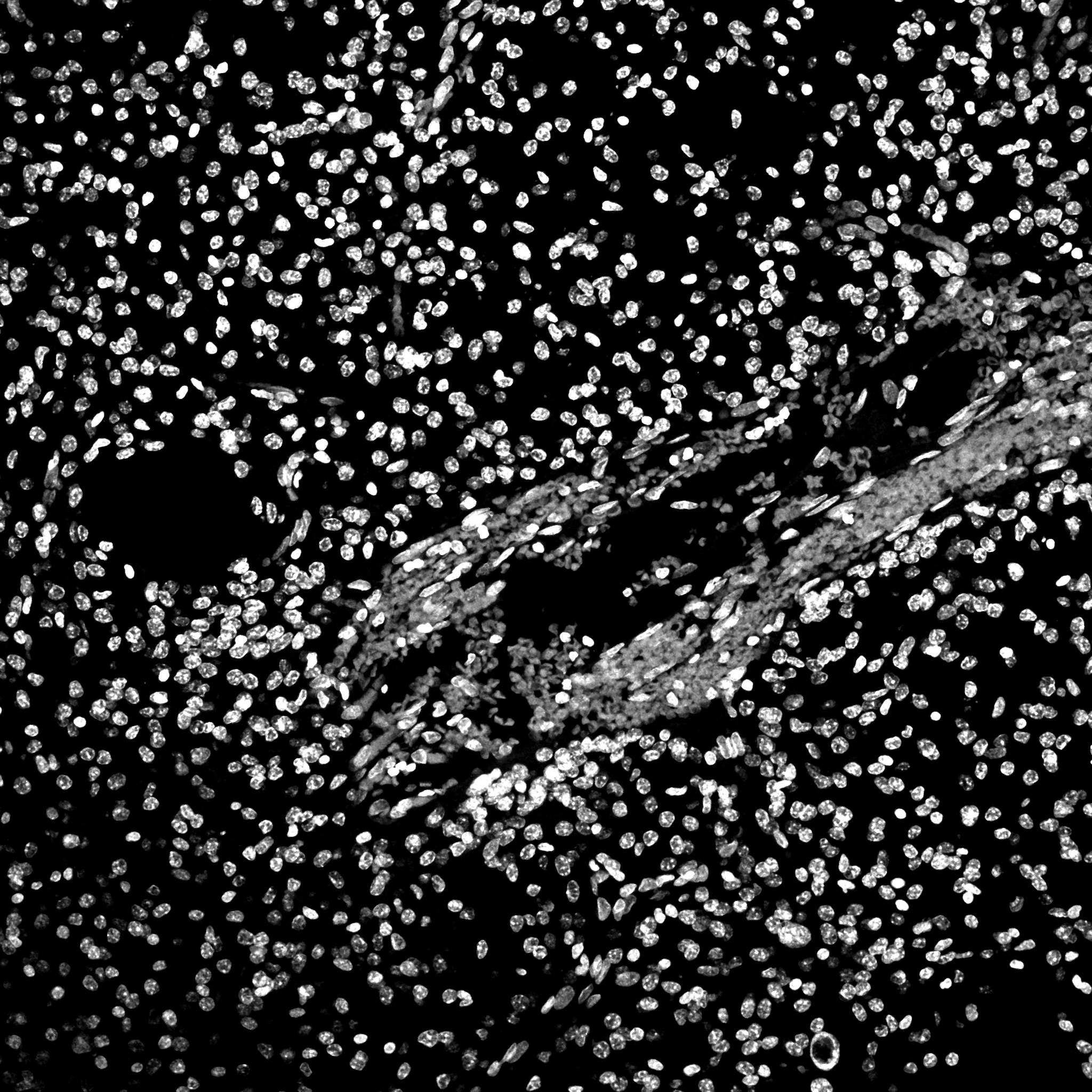

Supplement: Supplementary file 8 — Source Data for Figure 3 [file EMMM-15-e18199-s011.zip › Figure_3/3E/E'_Primary_T#21_CD34,_CD3_DAPI.tif]

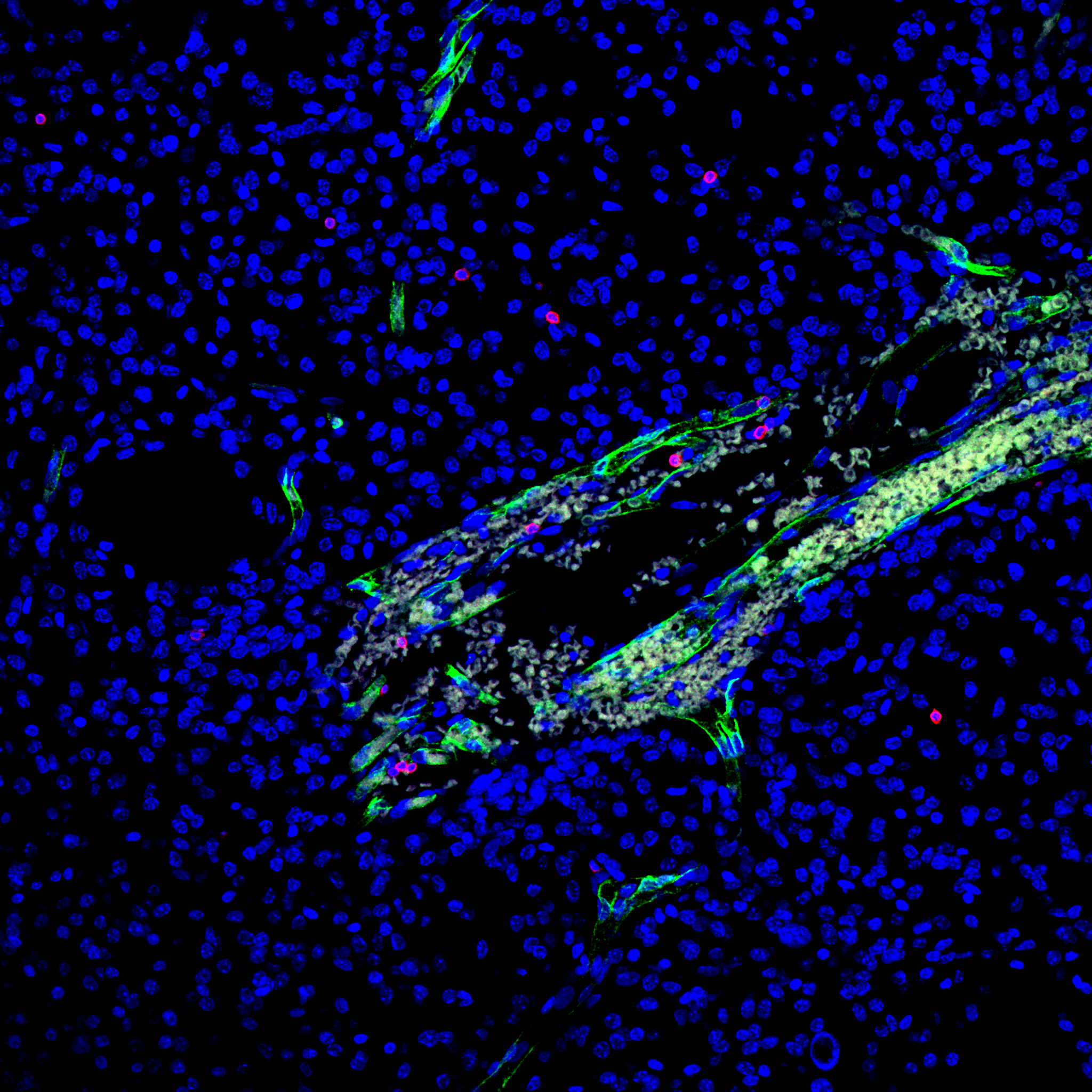

Supplement: Supplementary file 8 — Source Data for Figure 3 [file EMMM-15-e18199-s011.zip › Figure_3/3E/E'_Primary_T#21_CD34,_CD3_merge.tif]

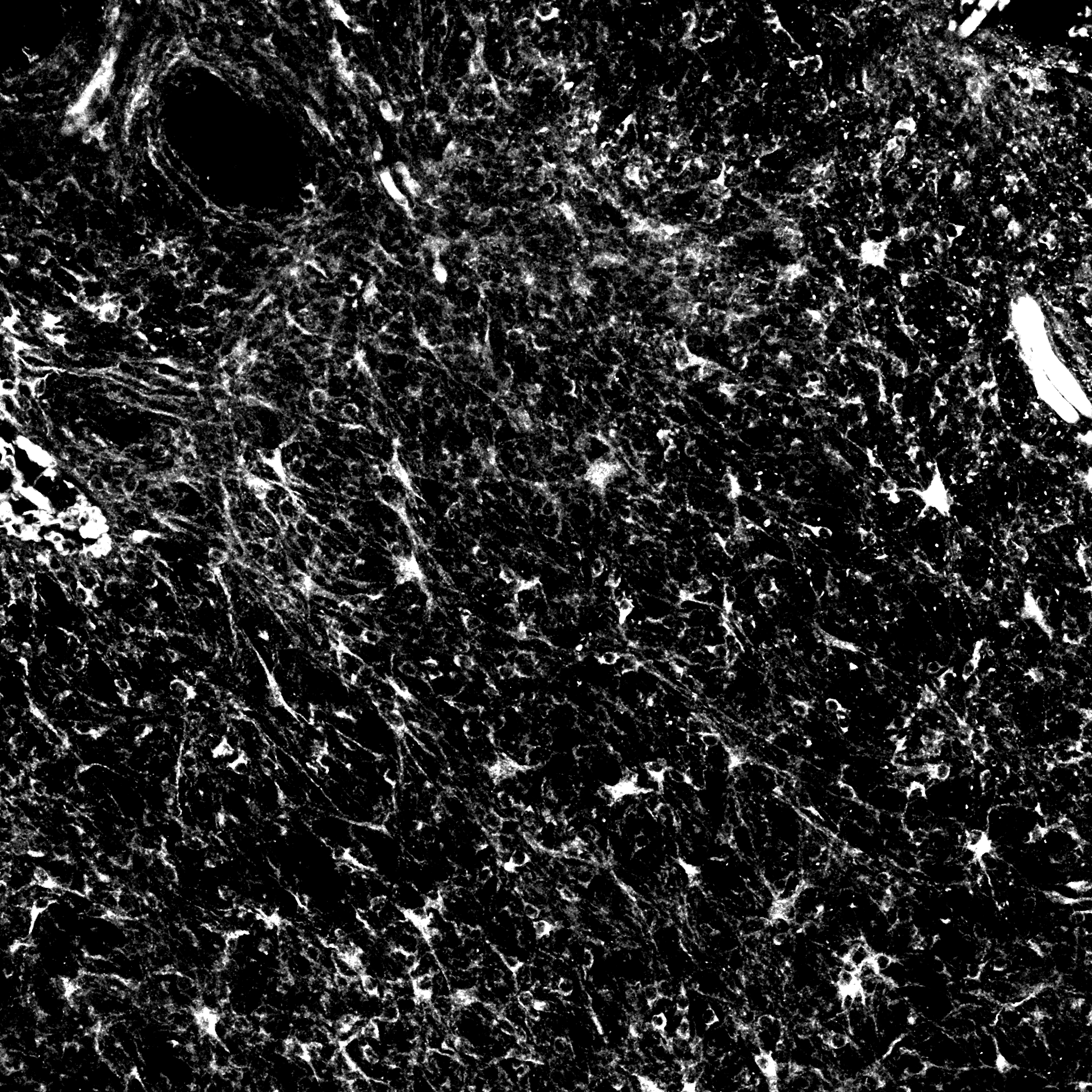

Supplement: Supplementary file 8 — Source Data for Figure 3 [file EMMM-15-e18199-s011.zip › Figure_3/3E/E'_Primary_T#21_GFAP,_B3tubulin_B3tubulin.tif]

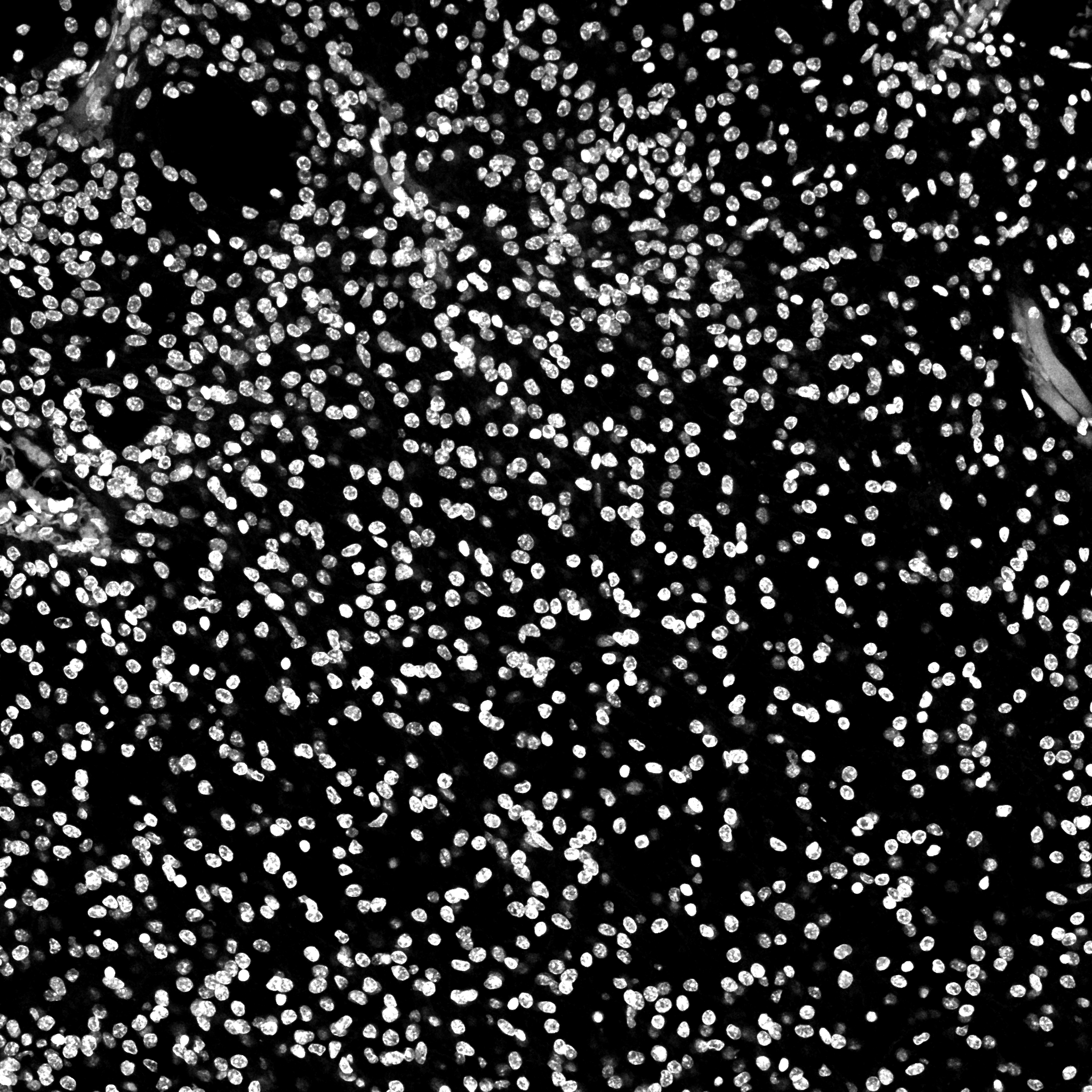

Supplement: Supplementary file 8 — Source Data for Figure 3 [file EMMM-15-e18199-s011.zip › Figure_3/3E/E'_Primary_T#21_GFAP,_B3tubulin_DAPI.tif]

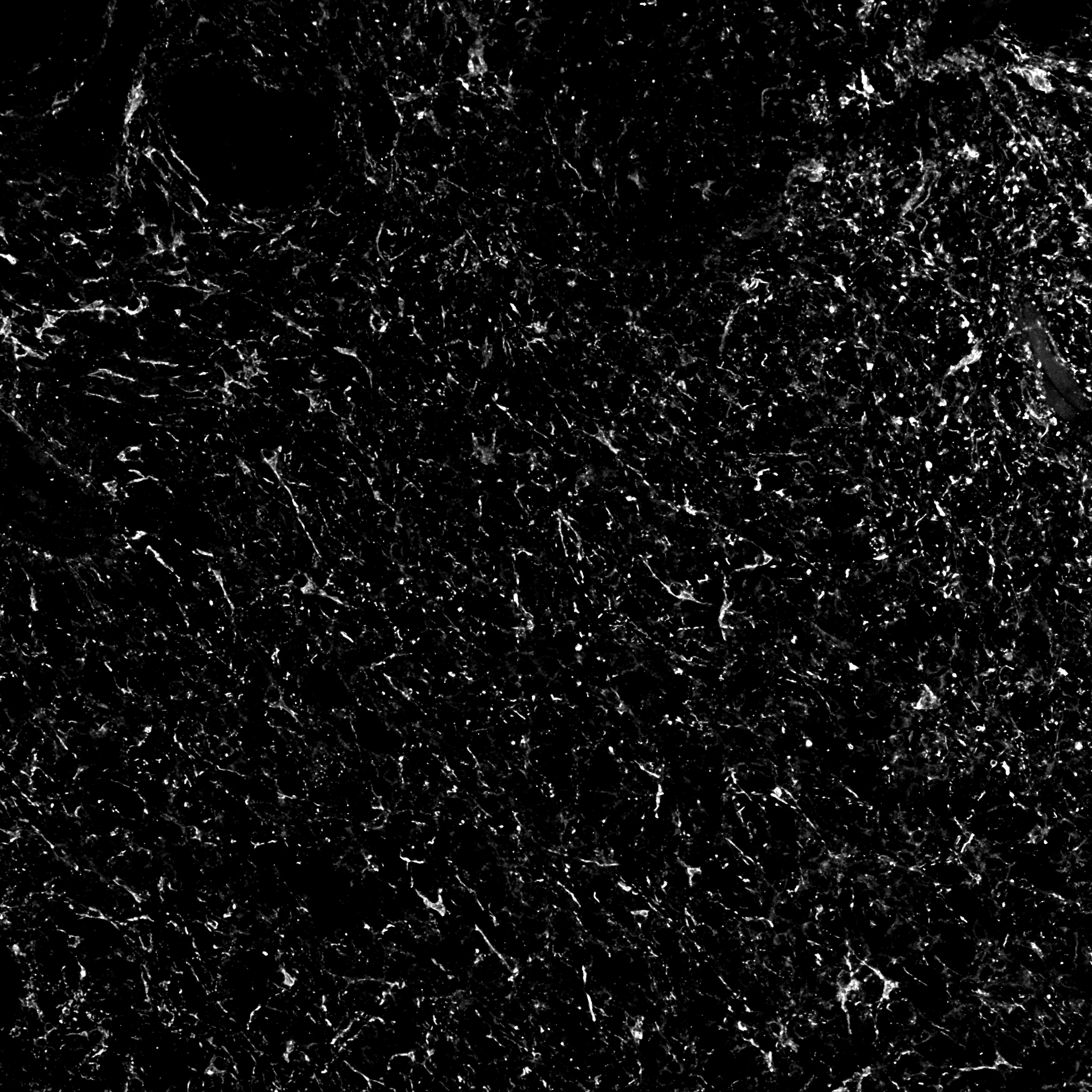

Supplement: Supplementary file 8 — Source Data for Figure 3 [file EMMM-15-e18199-s011.zip › Figure_3/3E/E'_Primary_T#21_GFAP,_B3tubulin_GFAP.tif]

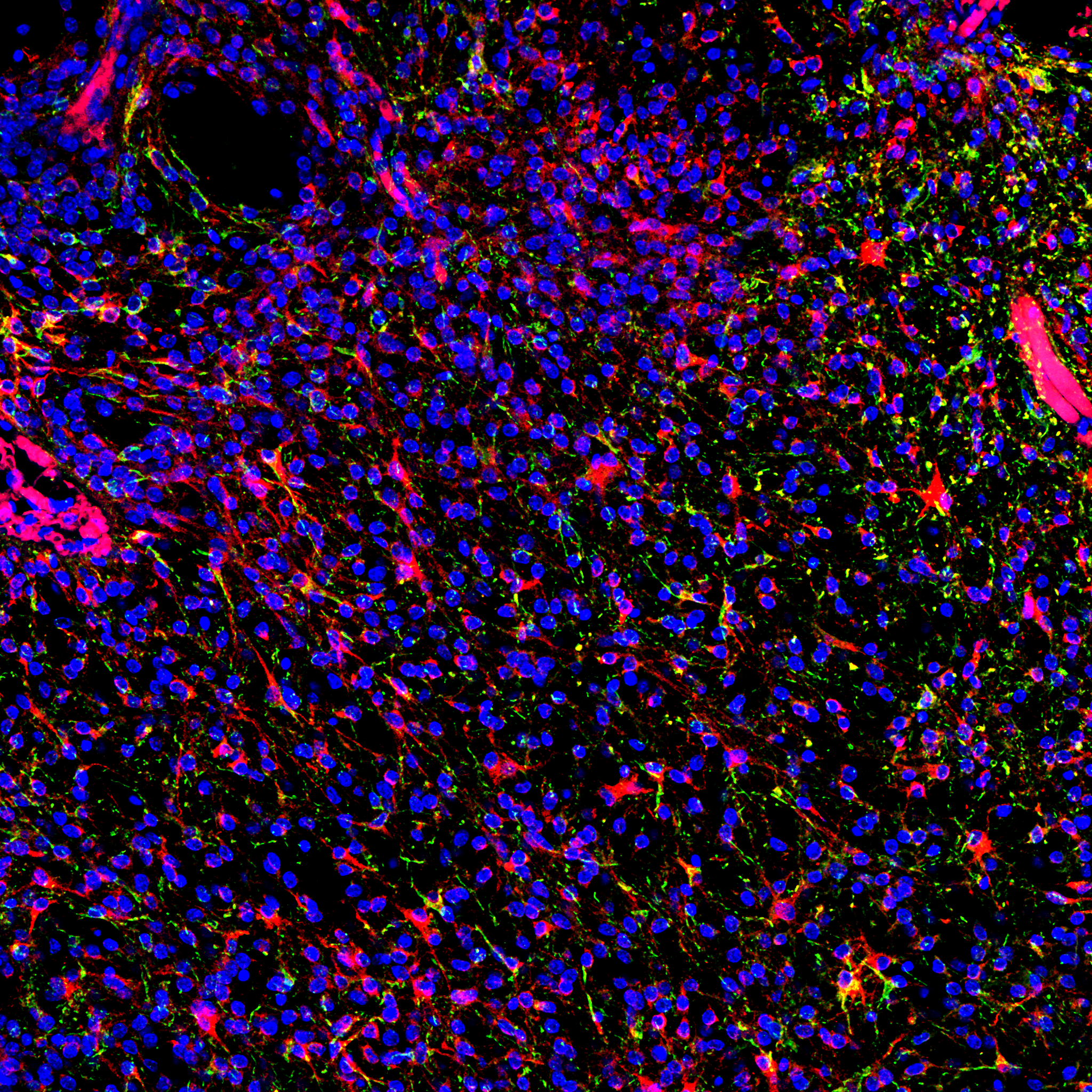

Supplement: Supplementary file 8 — Source Data for Figure 3 [file EMMM-15-e18199-s011.zip › Figure_3/3E/E'_Primary_T#21_GFAP,_B3tubulin_merge.tif]

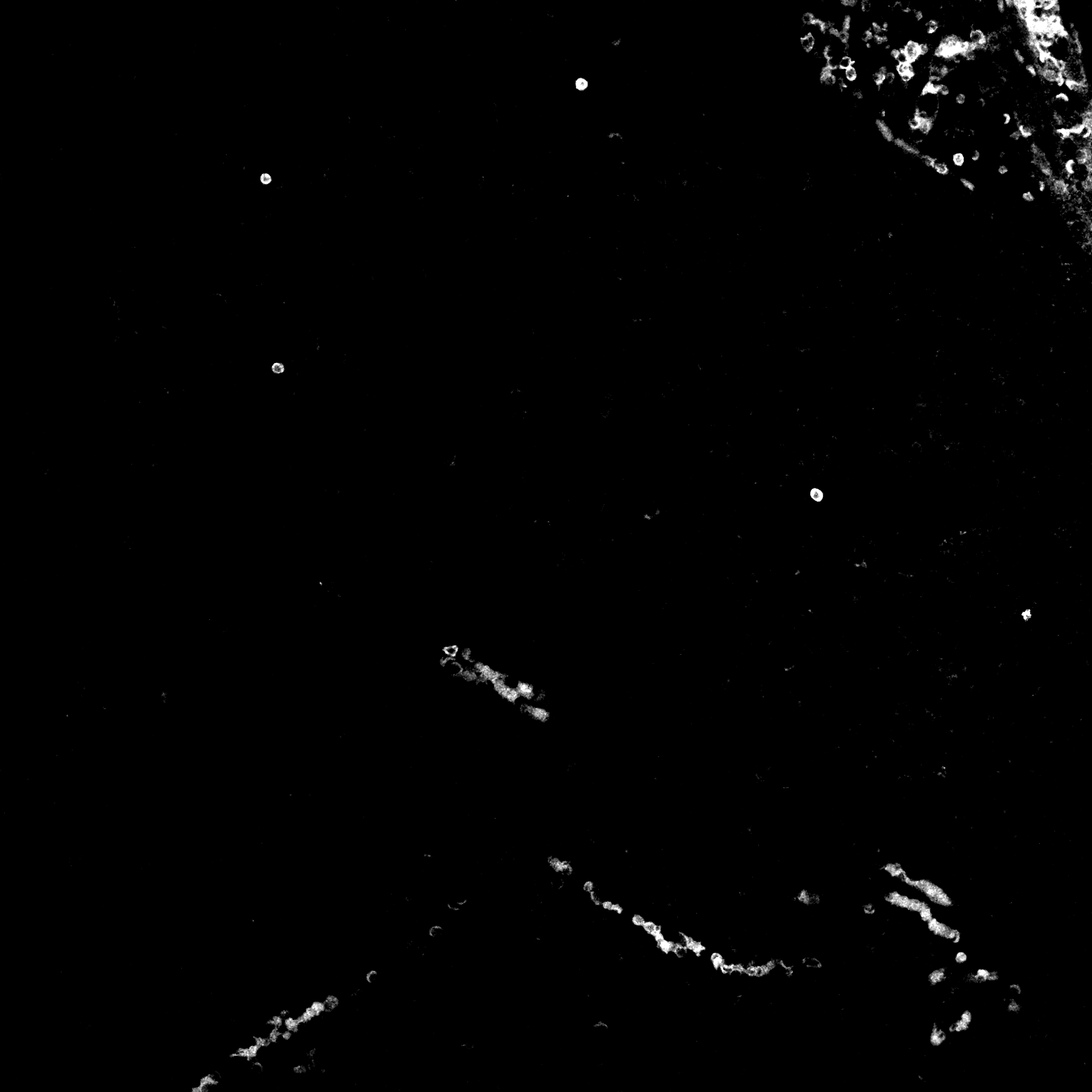

Supplement: Supplementary file 8 — Source Data for Figure 3 [file EMMM-15-e18199-s011.zip › Figure_3/3E/E'_Primary_T#21_IBA1,_CD3_CD3.tif]

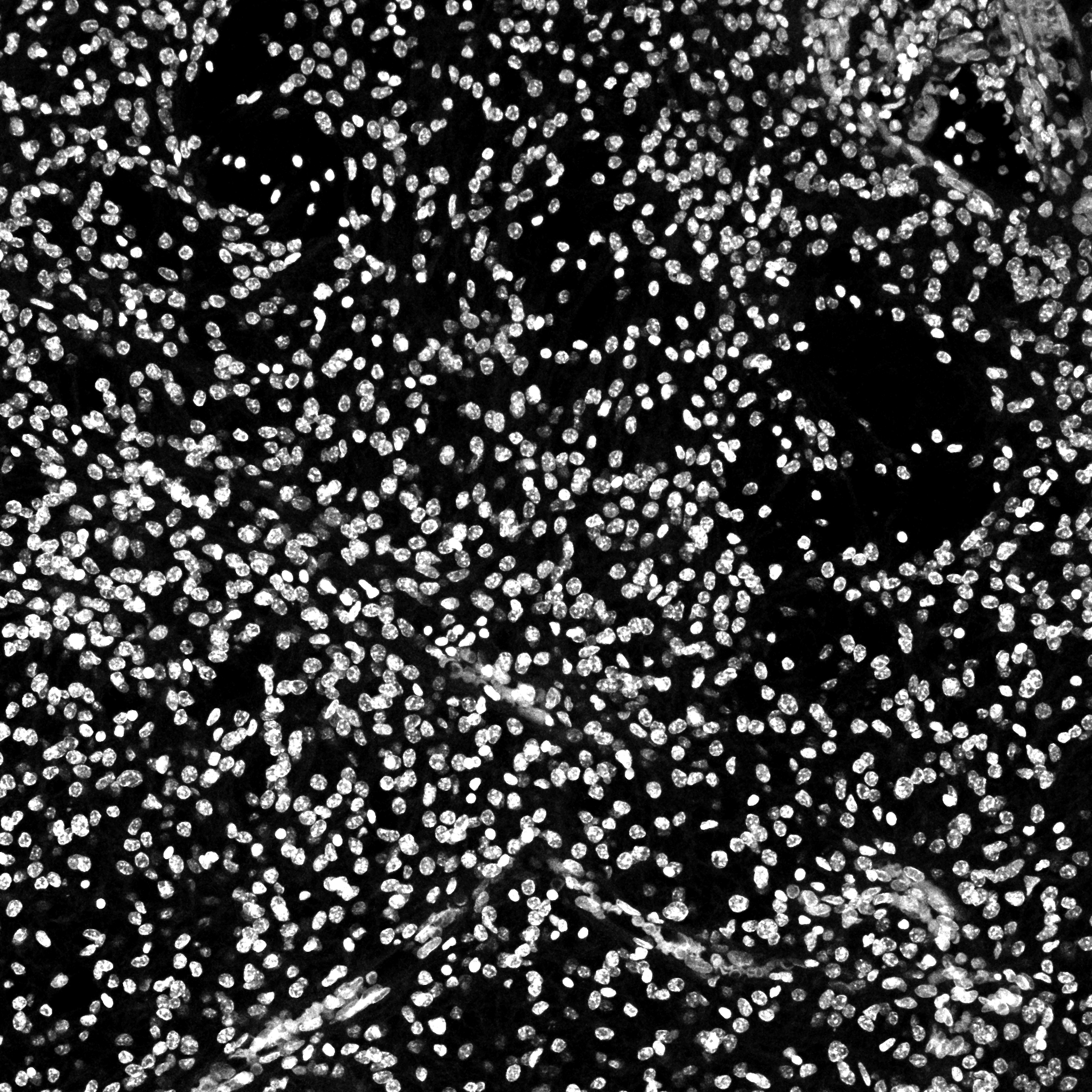

Supplement: Supplementary file 8 — Source Data for Figure 3 [file EMMM-15-e18199-s011.zip › Figure_3/3E/E'_Primary_T#21_IBA1,_CD3_DAPI.tif]

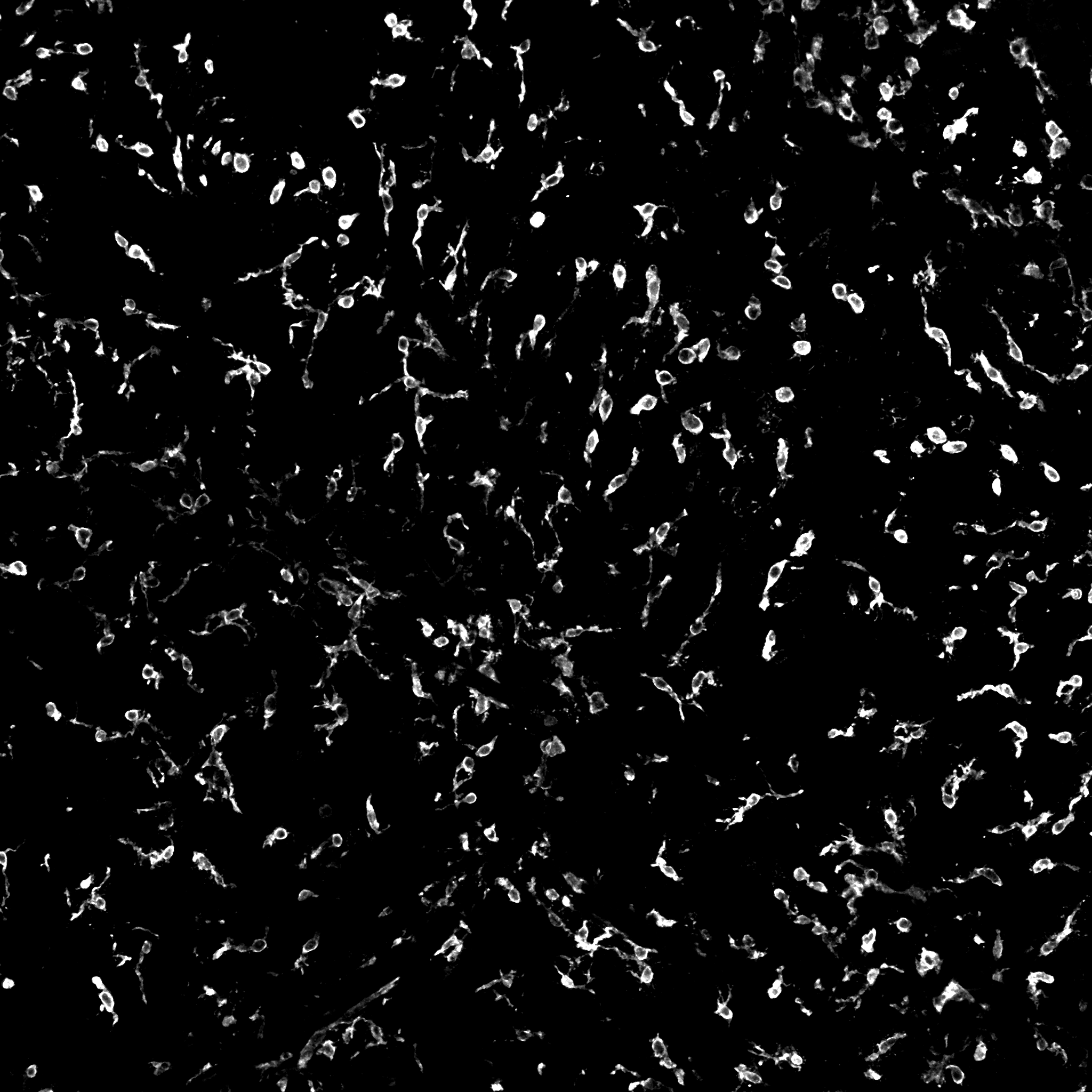

Supplement: Supplementary file 8 — Source Data for Figure 3 [file EMMM-15-e18199-s011.zip › Figure_3/3E/E'_Primary_T#21_IBA1,_CD3_IBA1.tif]

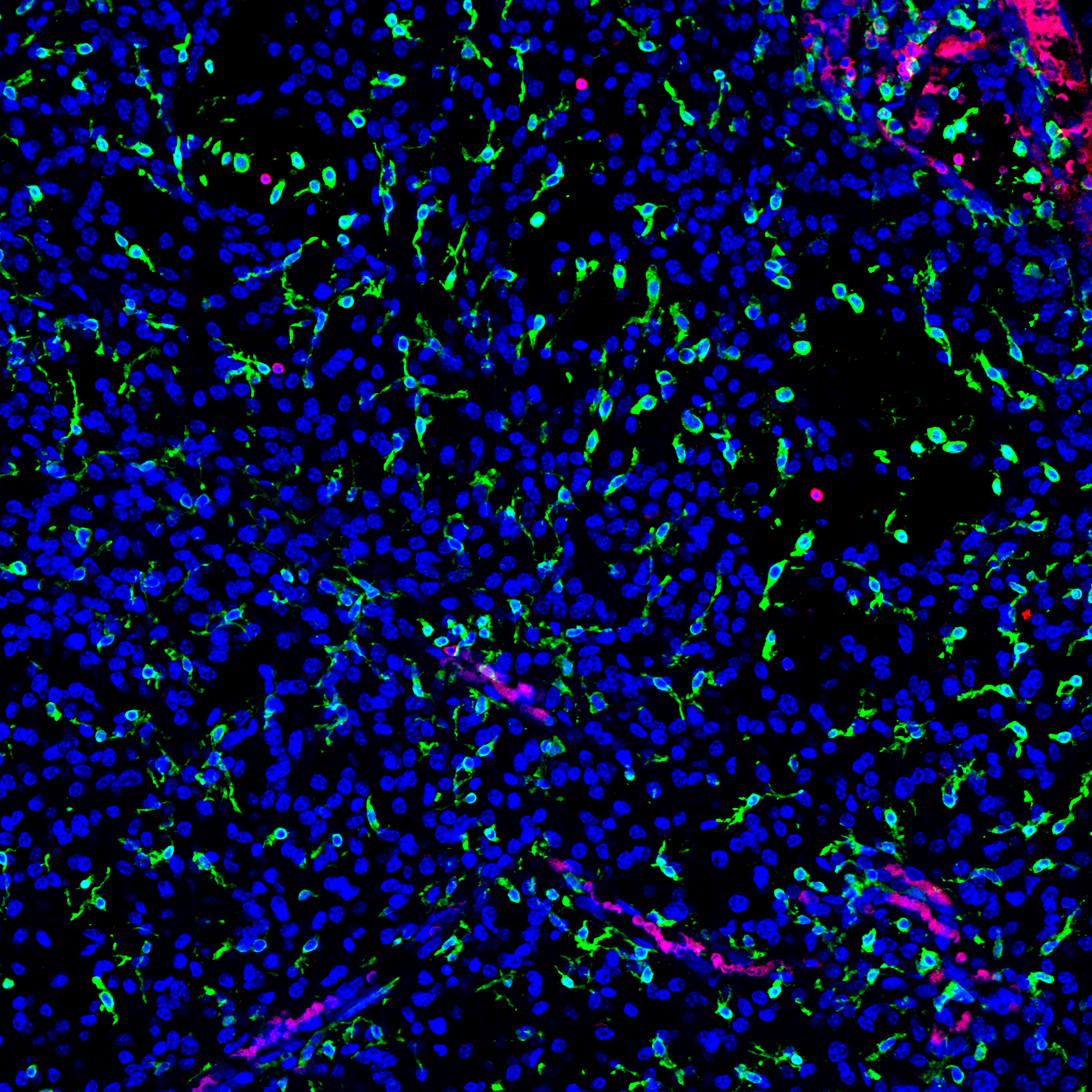

Supplement: Supplementary file 8 — Source Data for Figure 3 [file EMMM-15-e18199-s011.zip › Figure_3/3E/E'_Primary_T#21_IBA1,_CD3_merge.tif]

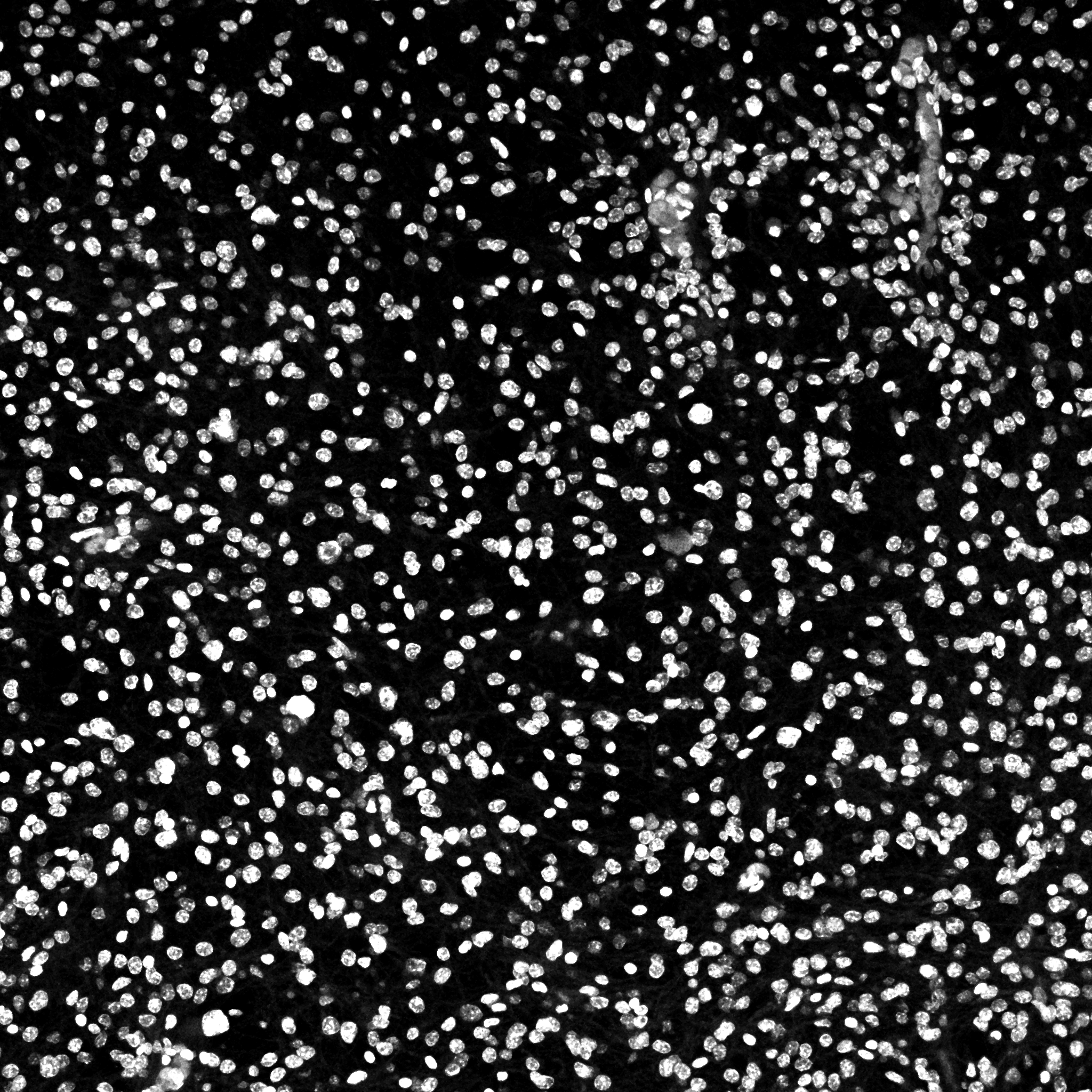

Supplement: Supplementary file 8 — Source Data for Figure 3 [file EMMM-15-e18199-s011.zip › Figure_3/3E/E'_Primary_T#21_Ki67,_SOX2_DAPI.tif]

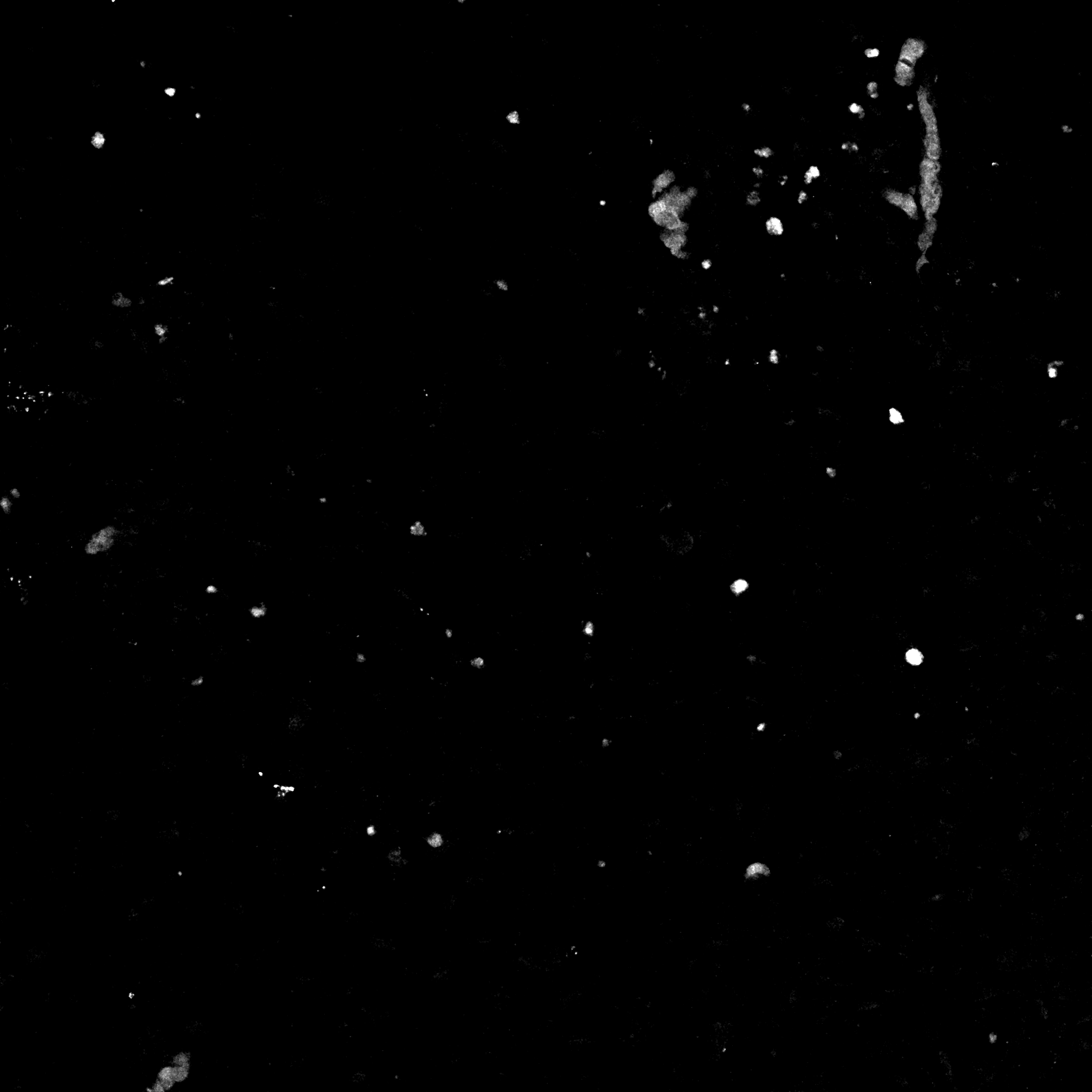

Supplement: Supplementary file 8 — Source Data for Figure 3 [file EMMM-15-e18199-s011.zip › Figure_3/3E/E'_Primary_T#21_Ki67,_SOX2_Ki67.tif]

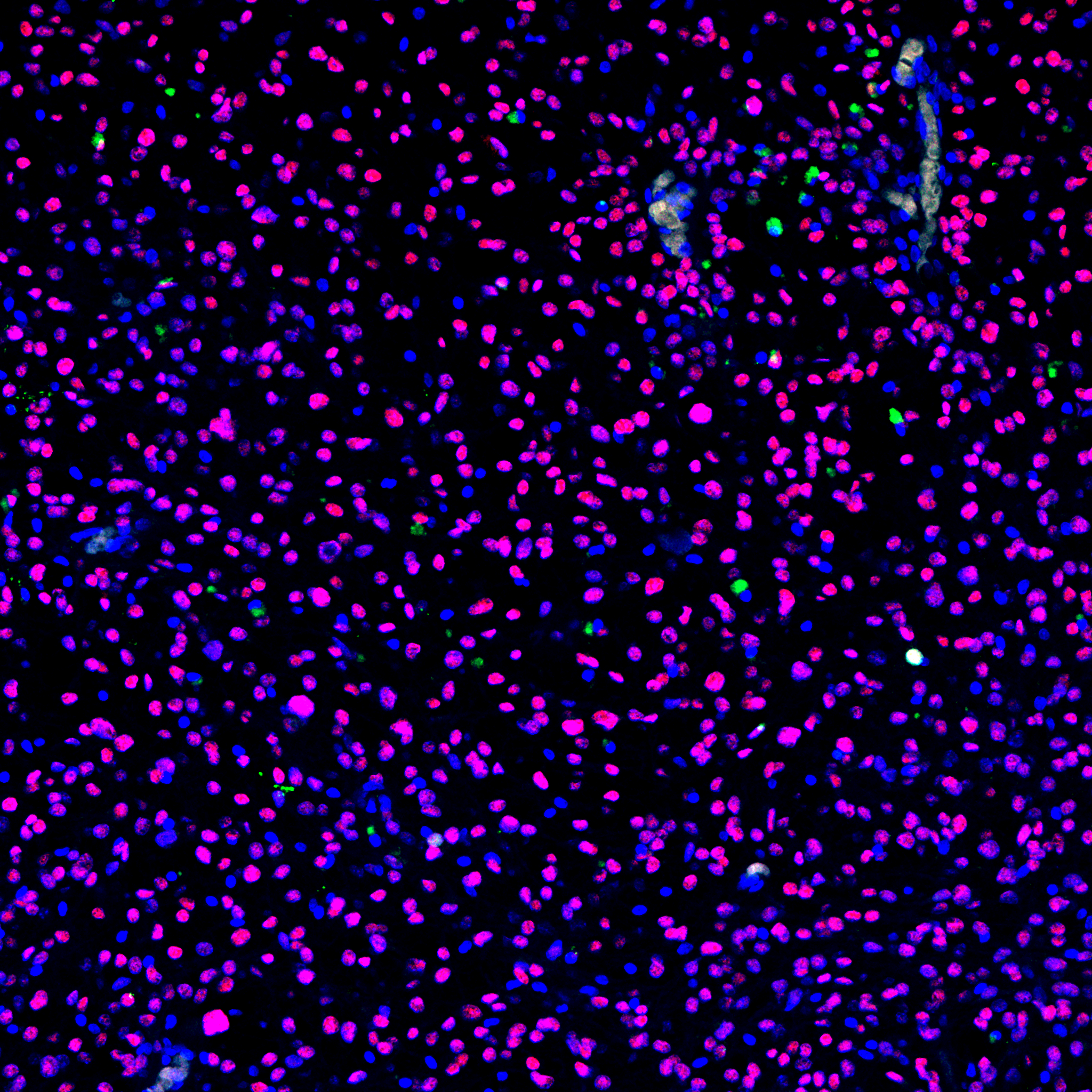

Supplement: Supplementary file 8 — Source Data for Figure 3 [file EMMM-15-e18199-s011.zip › Figure_3/3E/E'_Primary_T#21_Ki67,_SOX2_merge.tif]

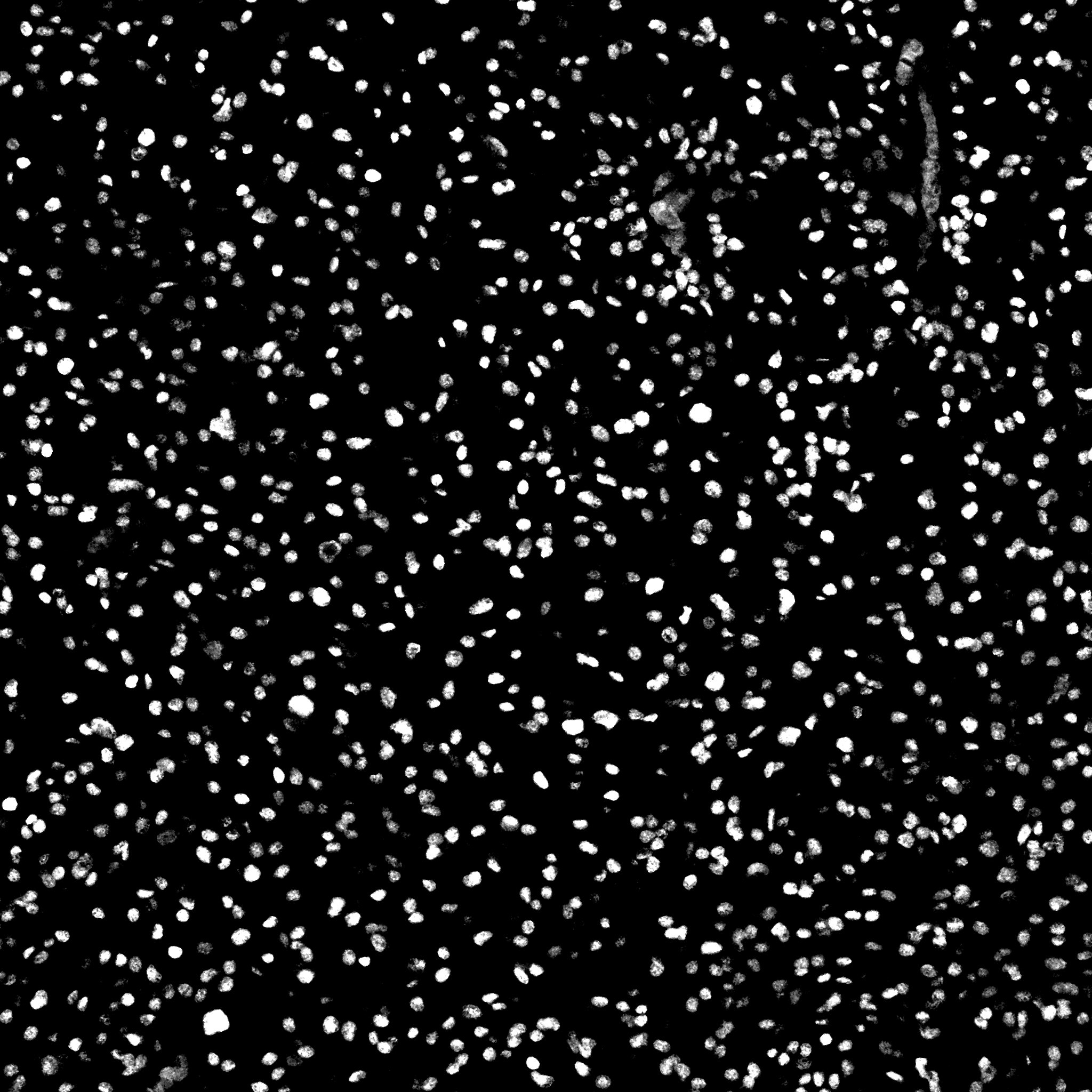

Supplement: Supplementary file 8 — Source Data for Figure 3 [file EMMM-15-e18199-s011.zip › Figure_3/3E/E'_Primary_T#21_Ki67,_SOX2_SOX2.tif]

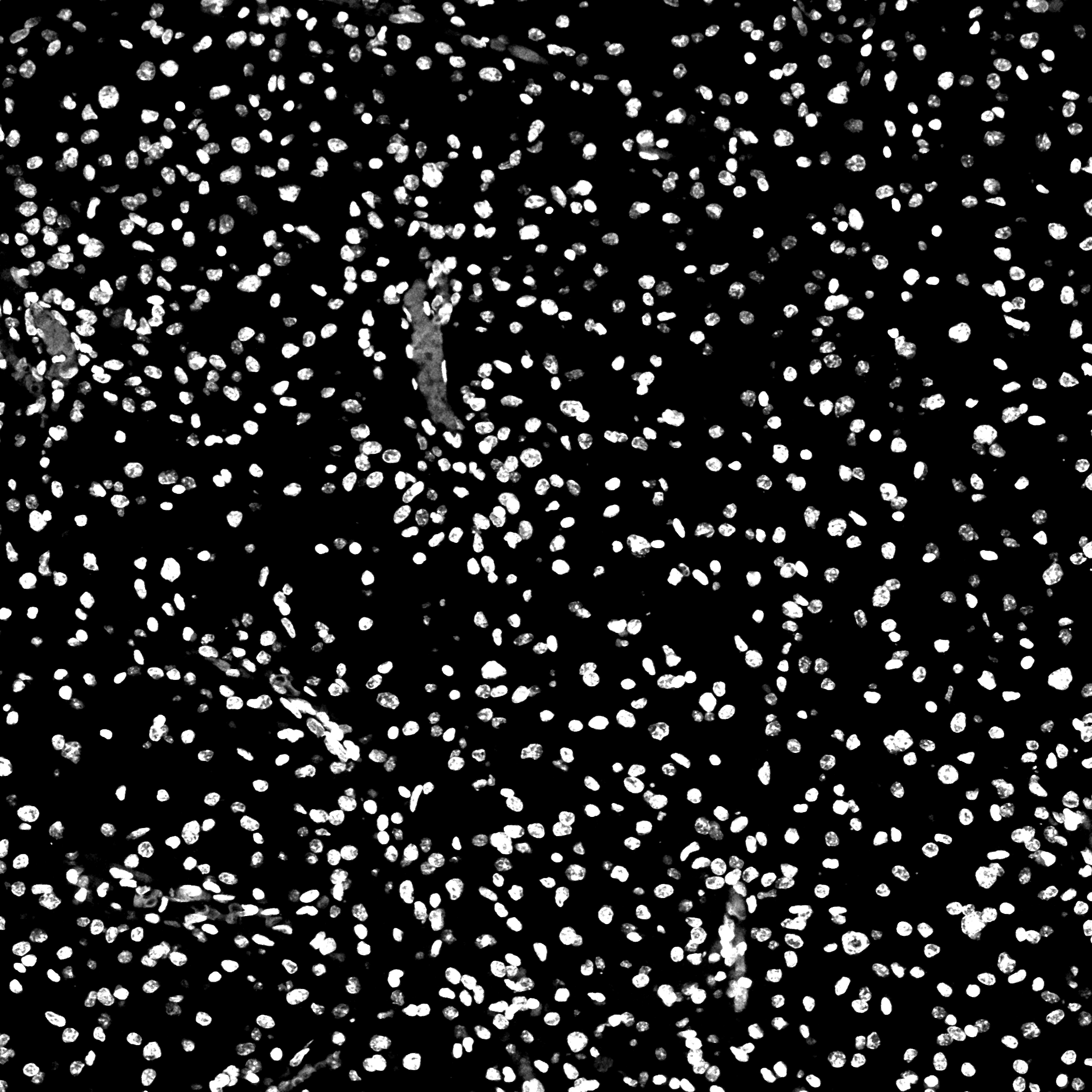

Supplement: Supplementary file 8 — Source Data for Figure 3 [file EMMM-15-e18199-s011.zip › Figure_3/3E/E'_Primary_T#21_OLIG2,_Nestin_DAPI.tif]

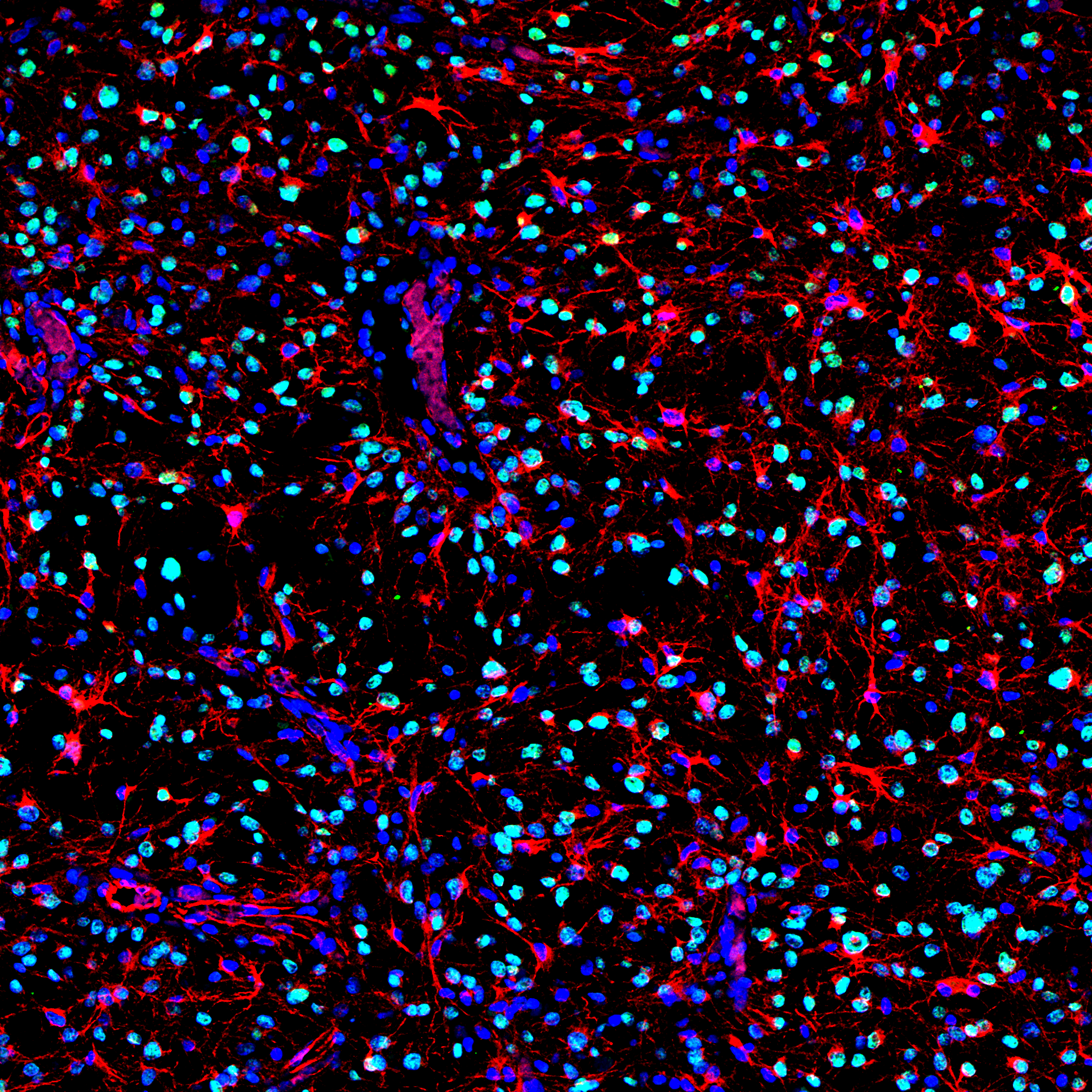

Supplement: Supplementary file 8 — Source Data for Figure 3 [file EMMM-15-e18199-s011.zip › Figure_3/3E/E'_Primary_T#21_OLIG2,_Nestin_merge.tif]

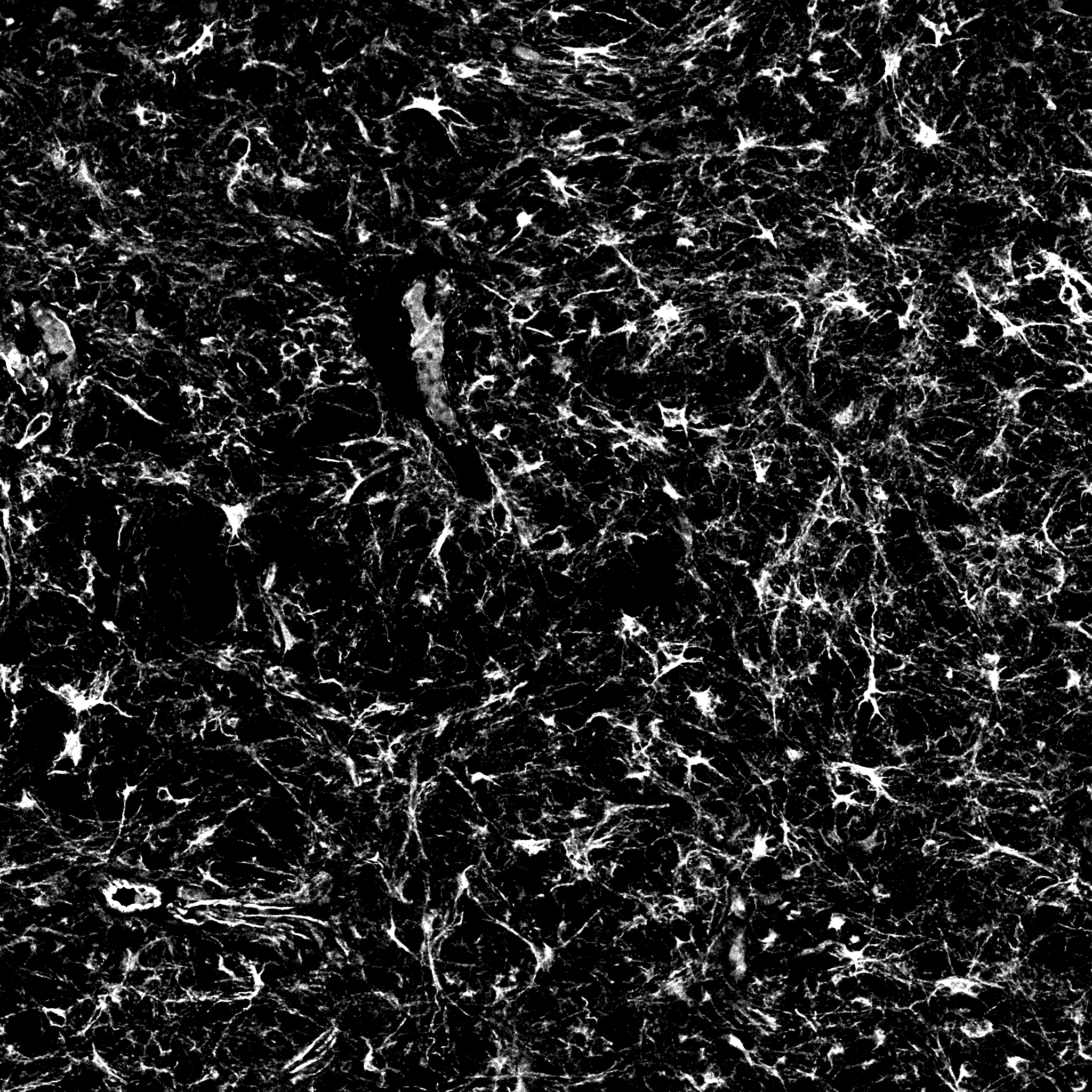

Supplement: Supplementary file 8 — Source Data for Figure 3 [file EMMM-15-e18199-s011.zip › Figure_3/3E/E'_Primary_T#21_OLIG2,_Nestin_Nestin.tif]

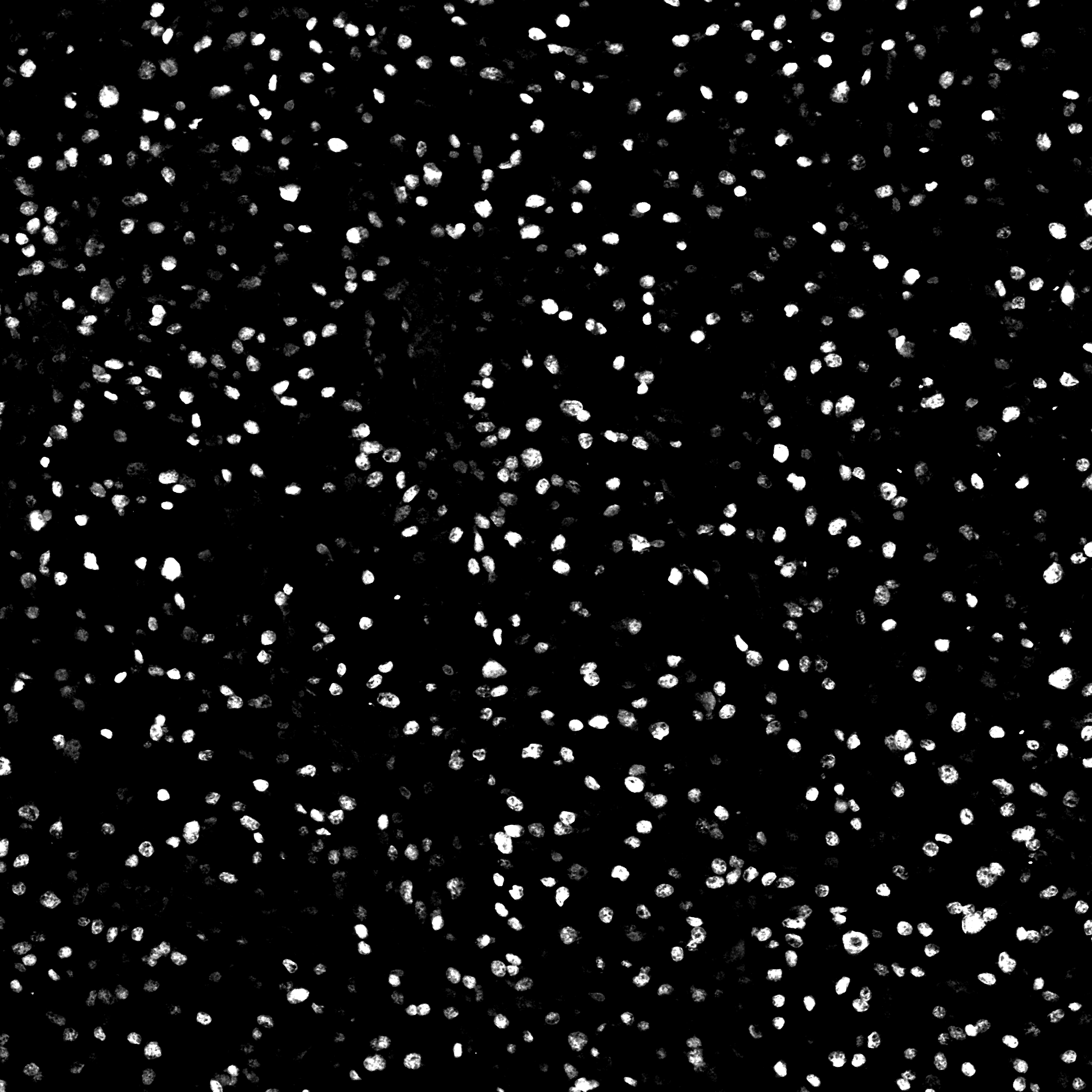

Supplement: Supplementary file 8 — Source Data for Figure 3 [file EMMM-15-e18199-s011.zip › Figure_3/3E/E'_Primary_T#21_OLIG2,_Nestin_OLIG2.tif]

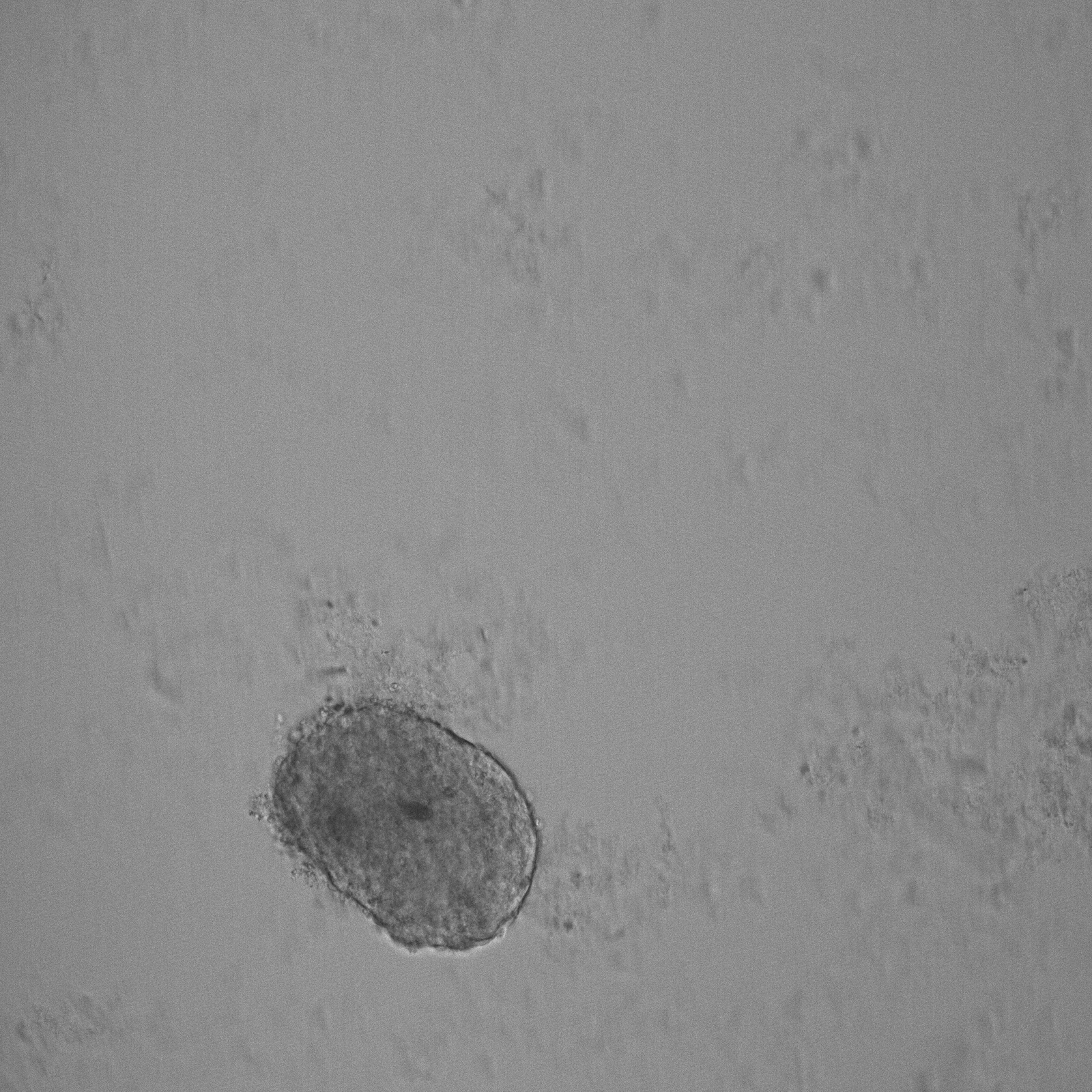

Supplement: Supplementary file 10 — Source Data for Figure 5 [file EMMM-15-e18199-s008.zip › Figure_5/5D/Tumor_A_single_cells_spheroids_D14.tif]

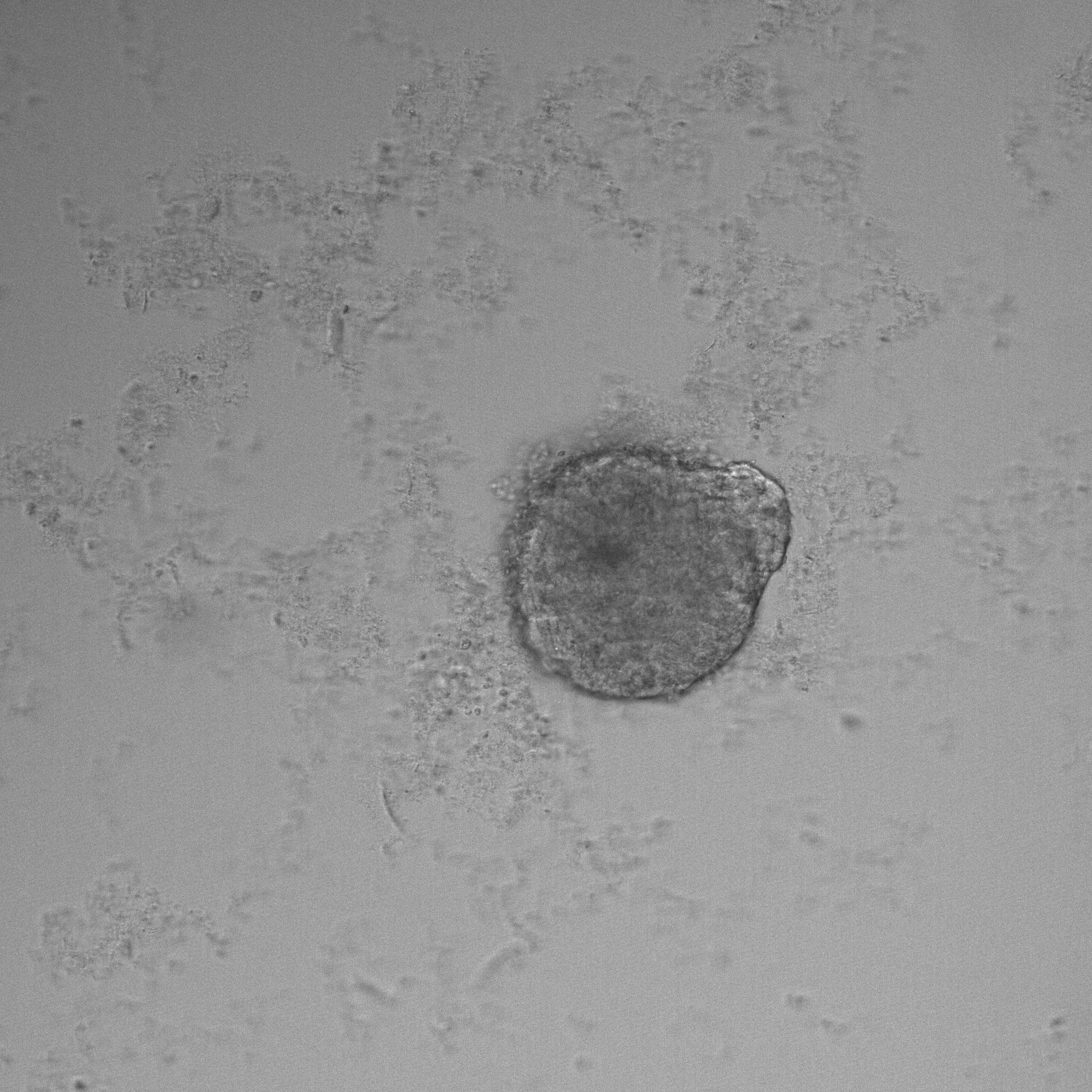

Supplement: Supplementary file 10 — Source Data for Figure 5 [file EMMM-15-e18199-s008.zip › Figure_5/5D/Tumor_A_single_cells_spheroids_D21.tif]

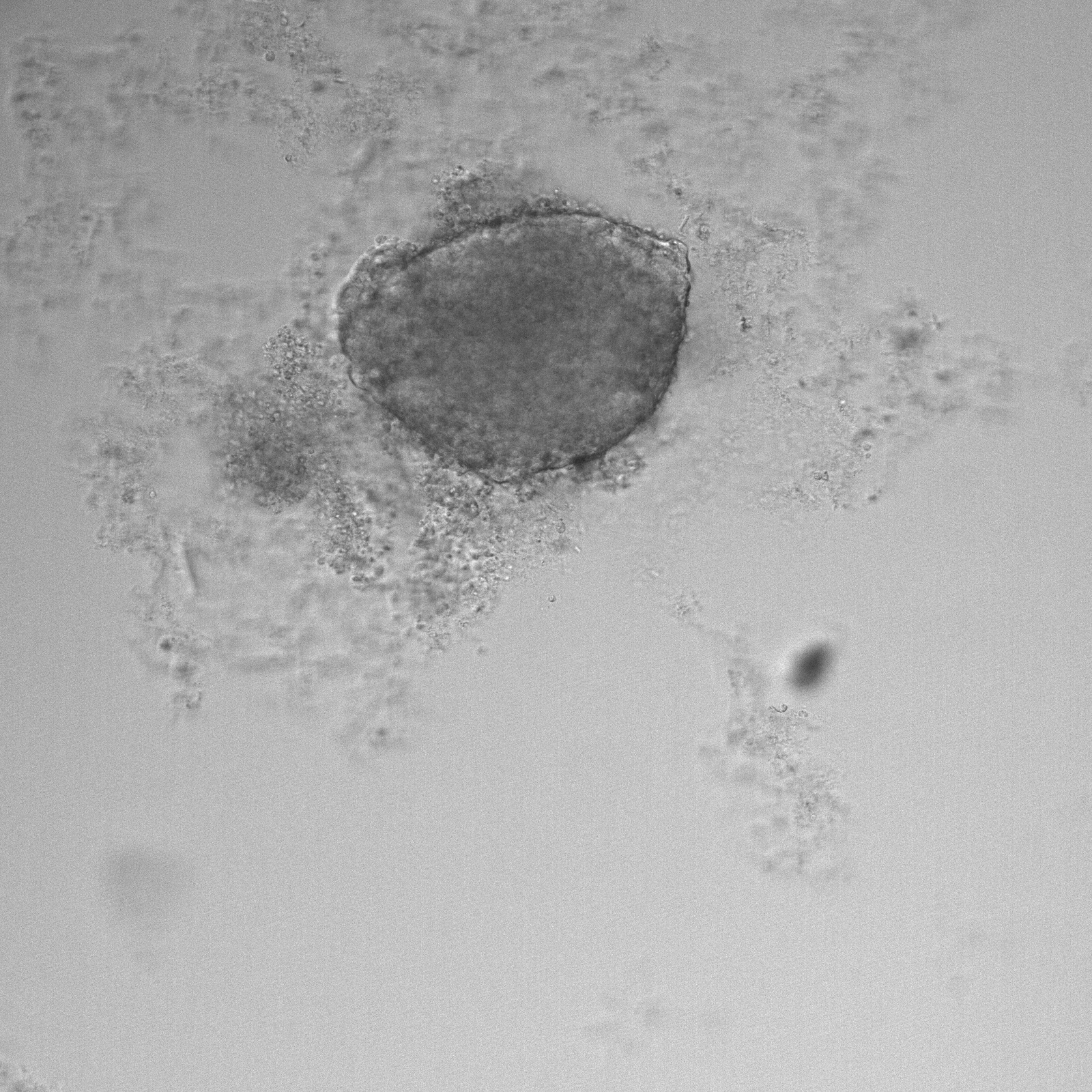

Supplement: Supplementary file 10 — Source Data for Figure 5 [file EMMM-15-e18199-s008.zip › Figure_5/5D/Tumor_A_single_cells_spheroids_D28.tif]

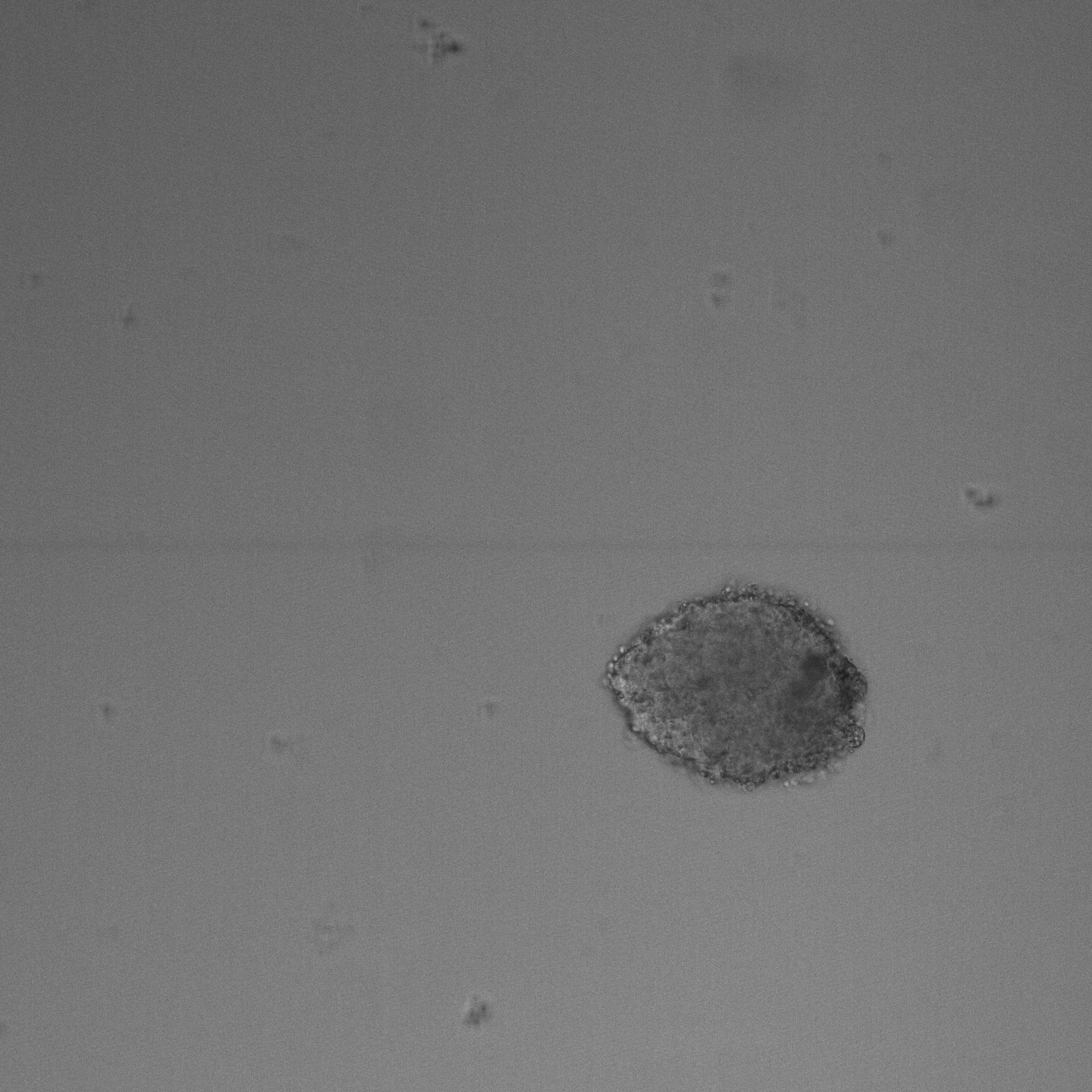

Supplement: Supplementary file 10 — Source Data for Figure 5 [file EMMM-15-e18199-s008.zip › Figure_5/5D/Tumor_A_single_cells_spheroids_D7.tif]

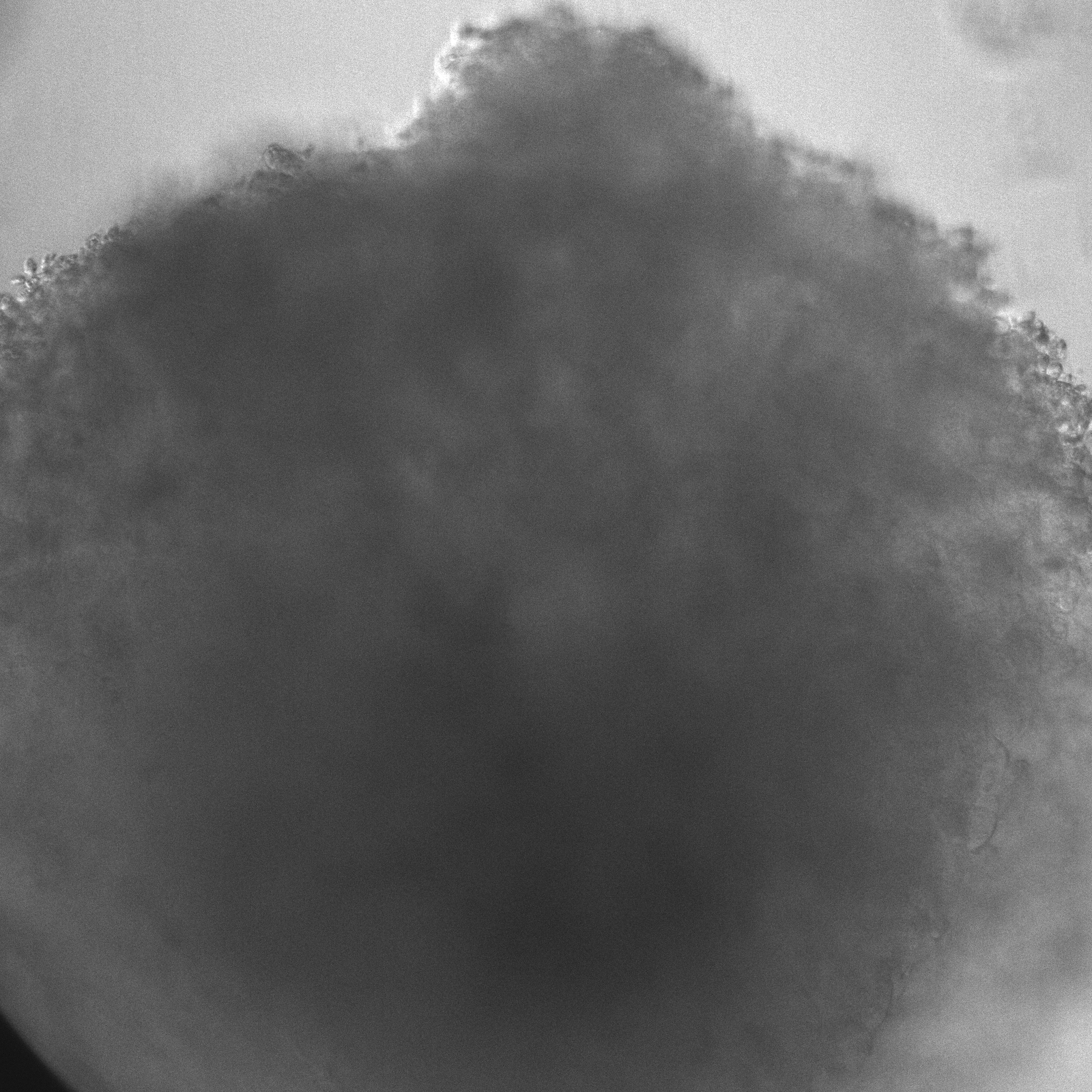

Supplement: Supplementary file 10 — Source Data for Figure 5 [file EMMM-15-e18199-s008.zip › Figure_5/5D/Tumor_B_single_cells_spheroids_D14.tif]

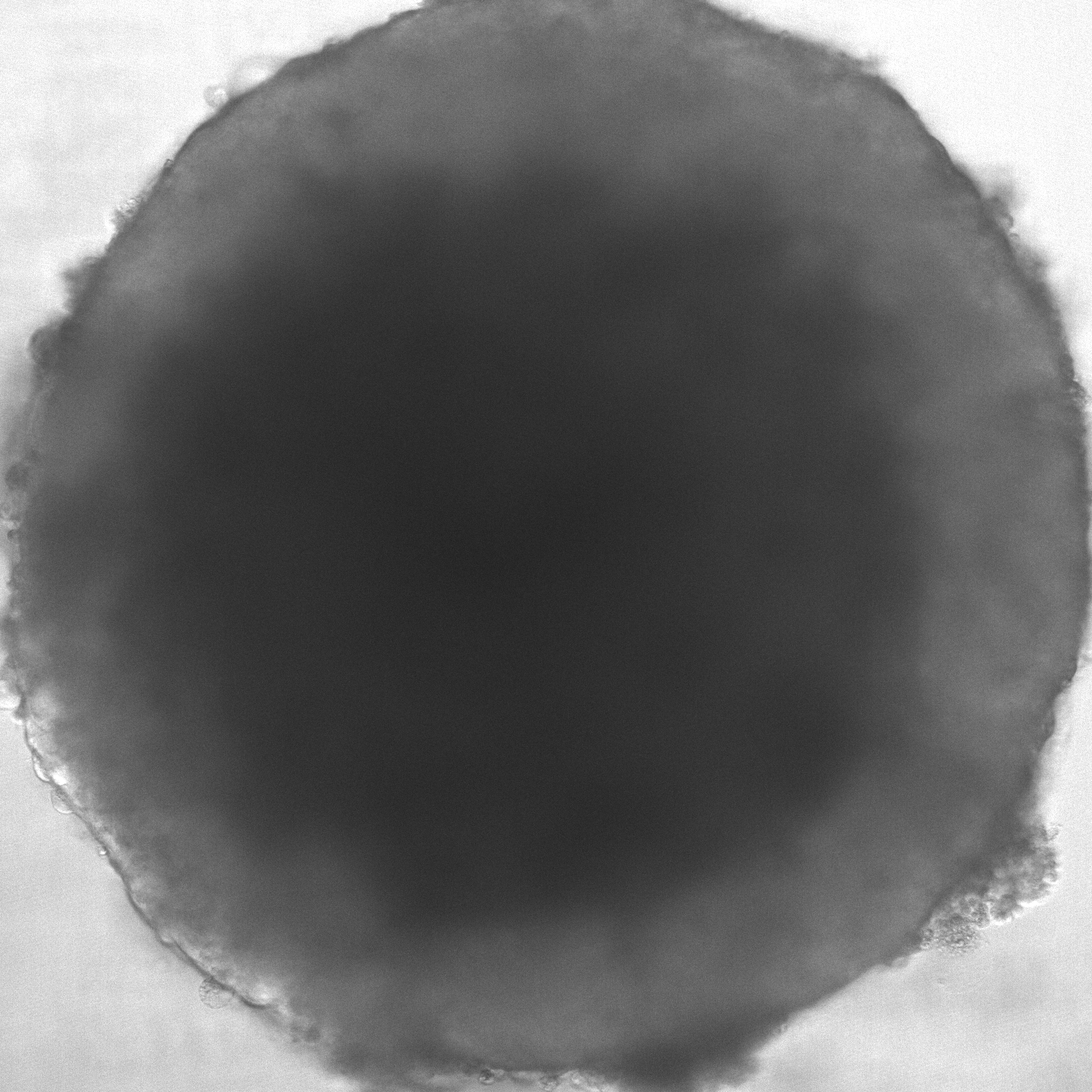

Supplement: Supplementary file 10 — Source Data for Figure 5 [file EMMM-15-e18199-s008.zip › Figure_5/5D/Tumor_B_single_cells_spheroids_D21.tif]

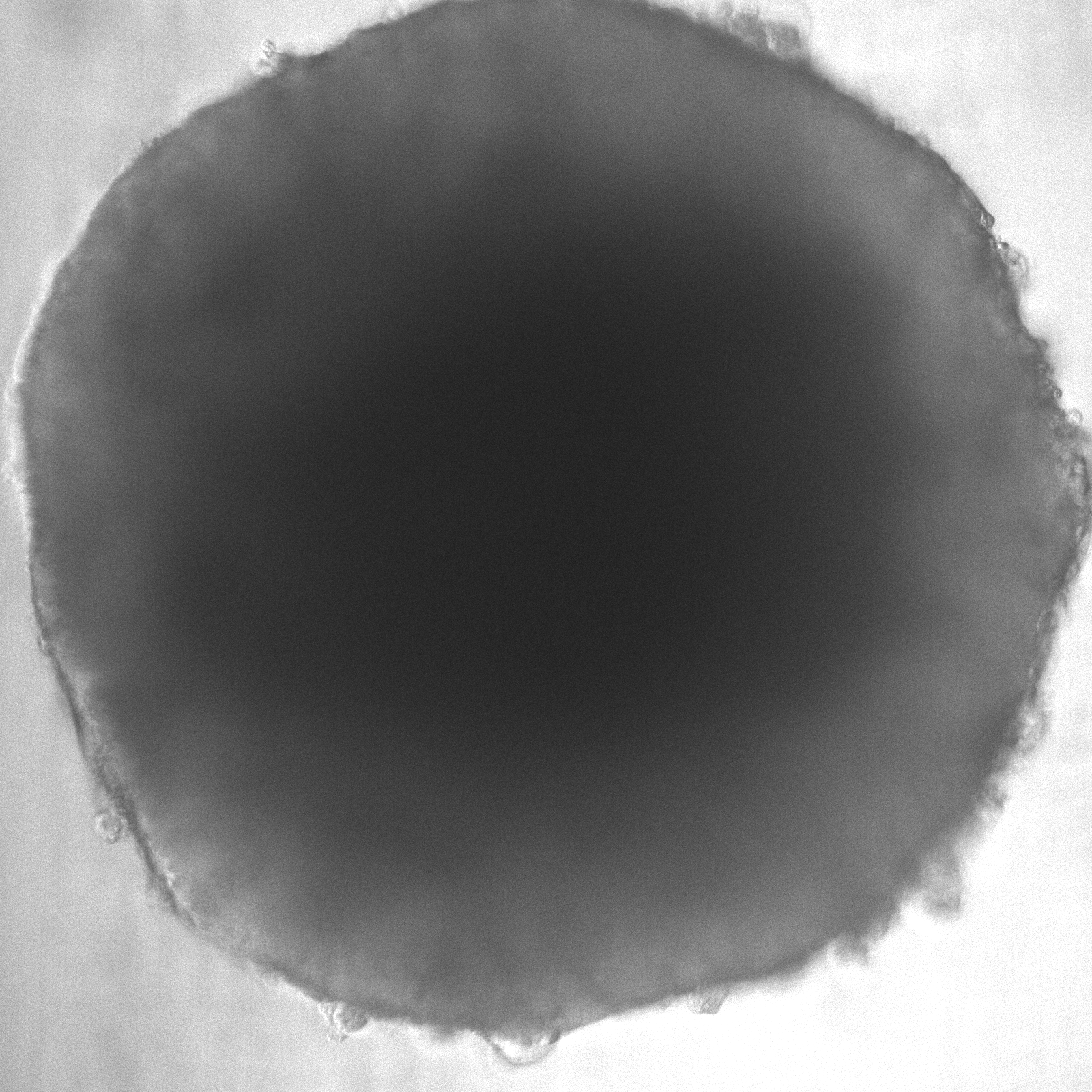

Supplement: Supplementary file 10 — Source Data for Figure 5 [file EMMM-15-e18199-s008.zip › Figure_5/5D/Tumor_B_single_cells_spheroids_D28.tif]

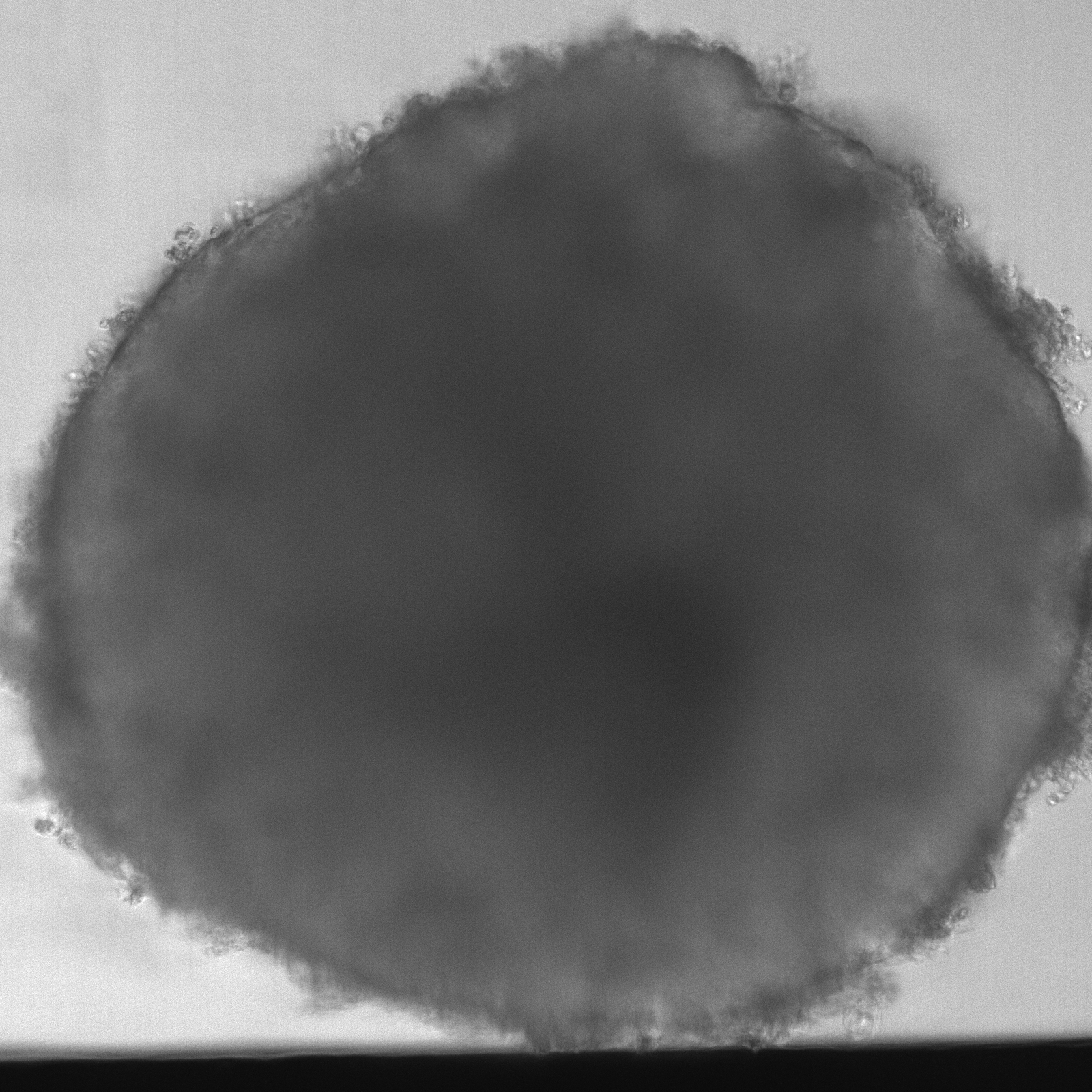

Supplement: Supplementary file 10 — Source Data for Figure 5 [file EMMM-15-e18199-s008.zip › Figure_5/5D/Tumor_B_single_cells_spheroids_D7.tif]

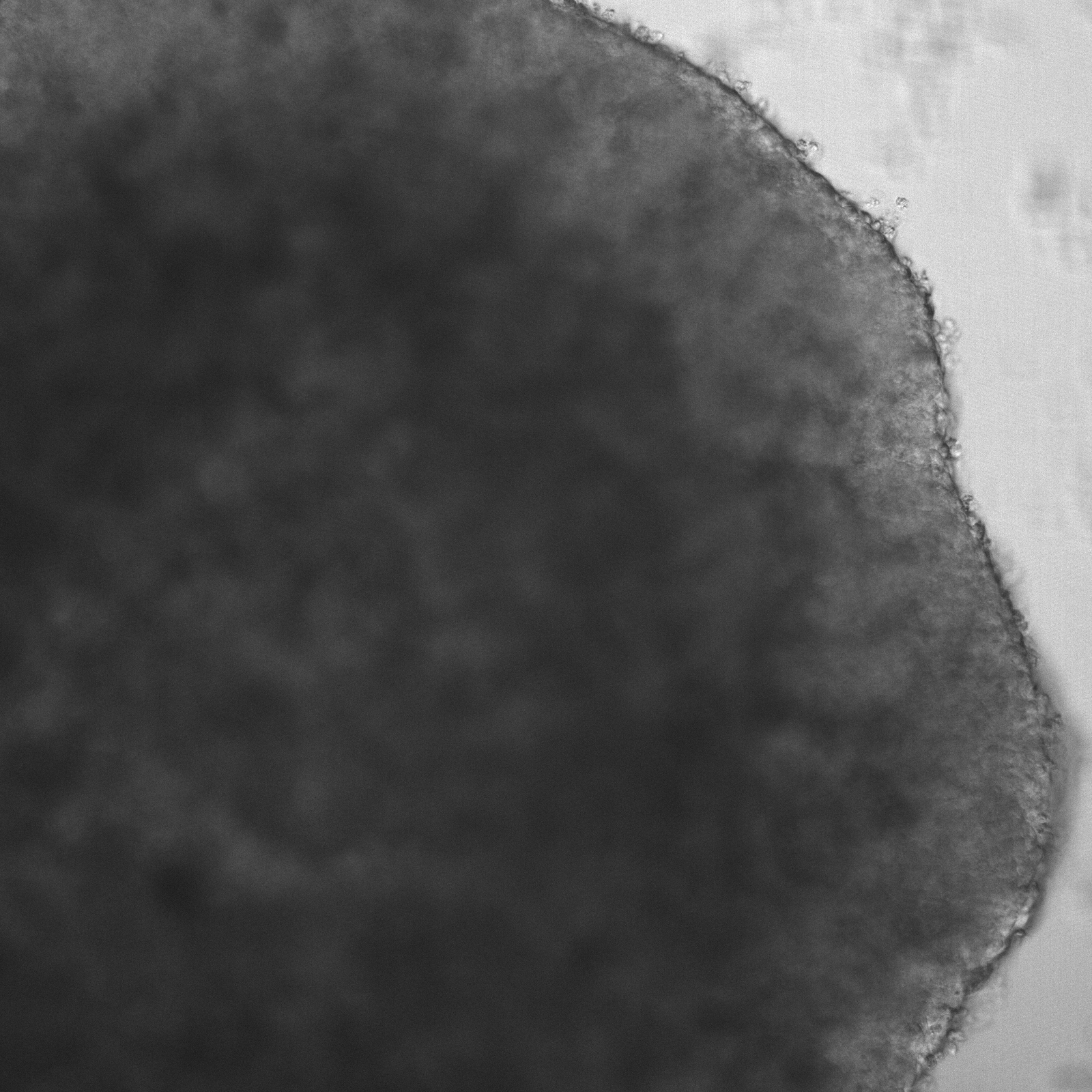

Supplement: Supplementary file 10 — Source Data for Figure 5 [file EMMM-15-e18199-s008.zip › Figure_5/5E/Tumor_C_single_cells_spheroids_D14.tif]

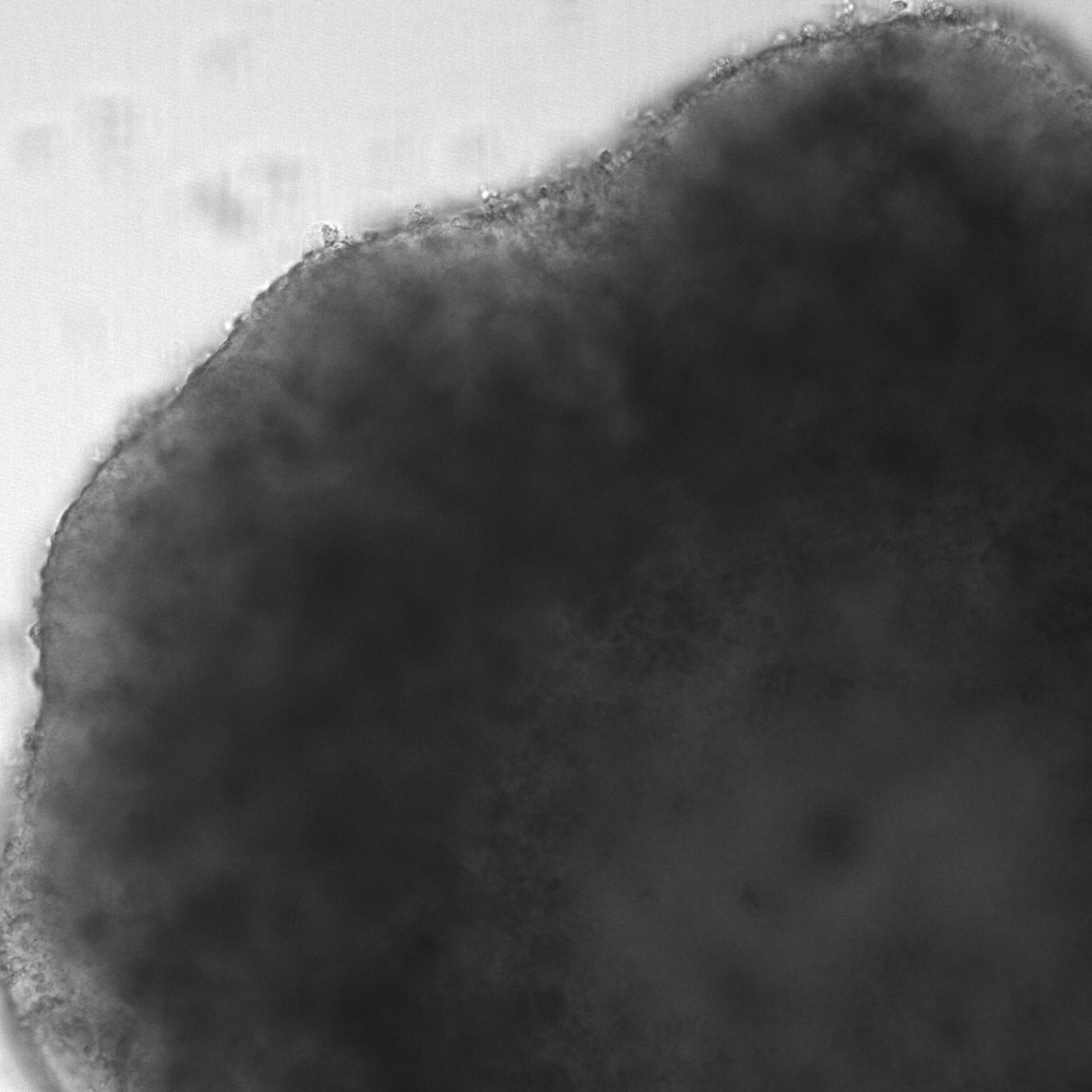

Supplement: Supplementary file 10 — Source Data for Figure 5 [file EMMM-15-e18199-s008.zip › Figure_5/5E/Tumor_C_single_cells_spheroids_D21.tif]

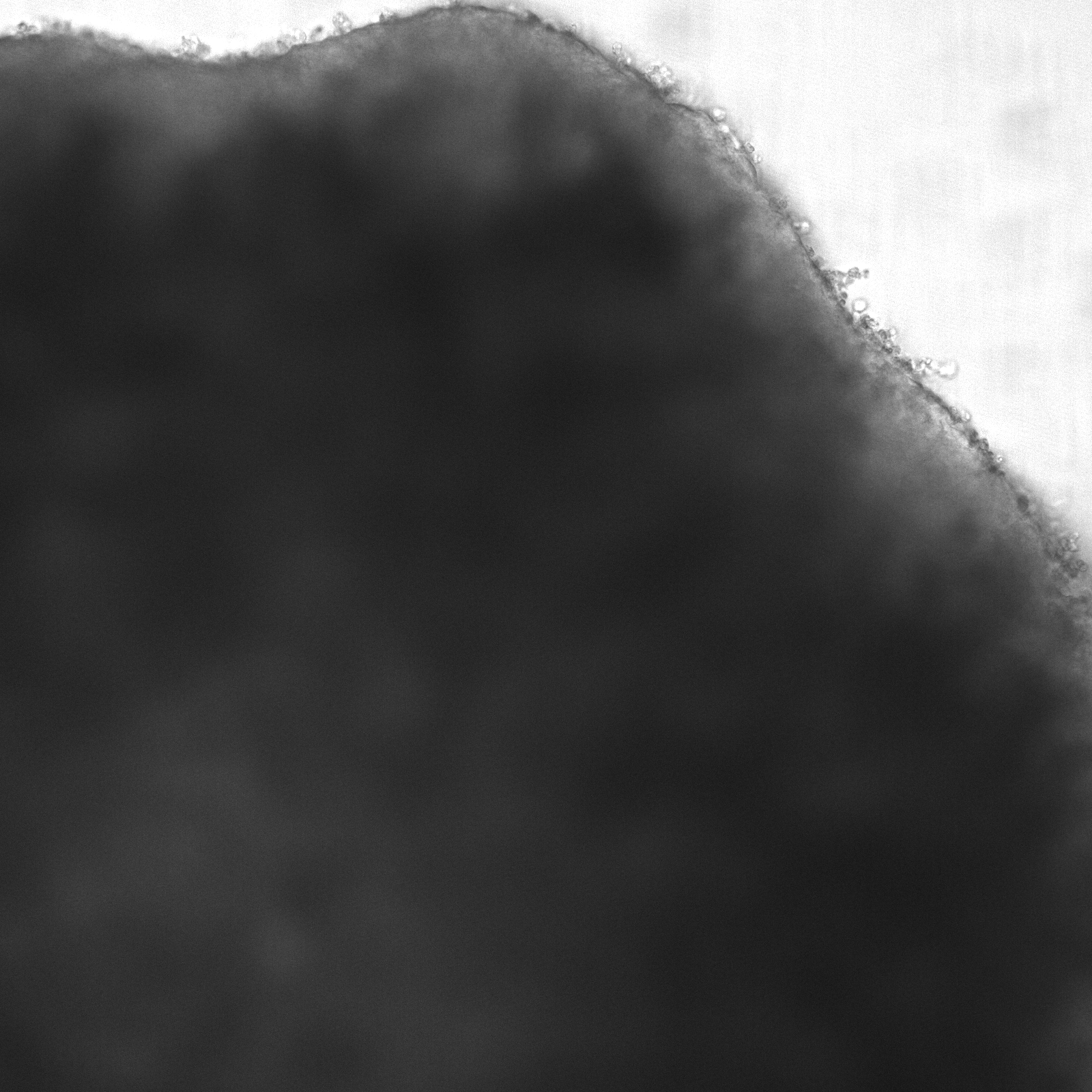

Supplement: Supplementary file 10 — Source Data for Figure 5 [file EMMM-15-e18199-s008.zip › Figure_5/5E/Tumor_C_single_cells_spheroids_D28.tif]

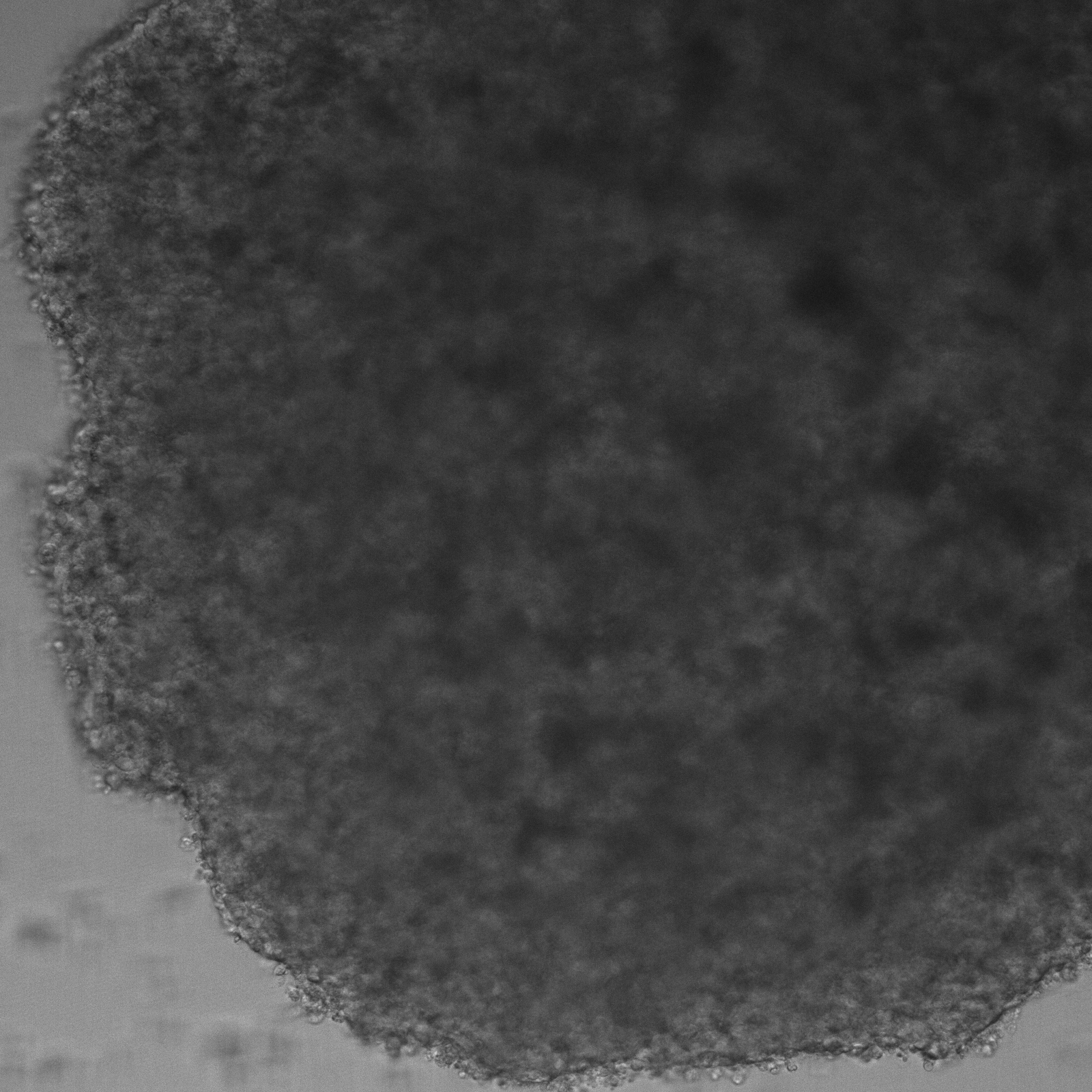

Supplement: Supplementary file 10 — Source Data for Figure 5 [file EMMM-15-e18199-s008.zip › Figure_5/5E/Tumor_C_single_cells_spheroids_D7.tif]

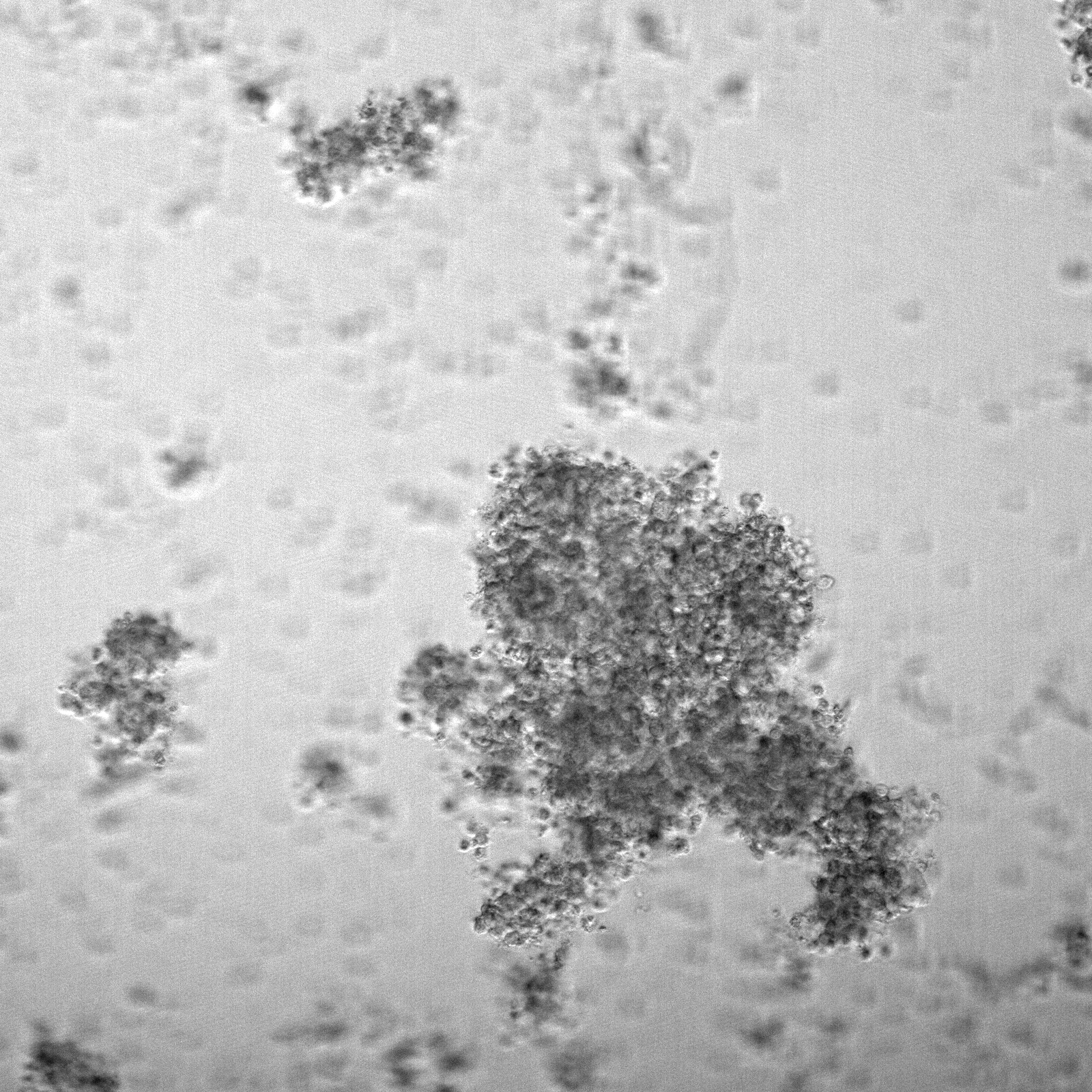

Supplement: Supplementary file 10 — Source Data for Figure 5 [file EMMM-15-e18199-s008.zip › Figure_5/5E/Tumor_D_single_cells_spheroids_D14.tif]

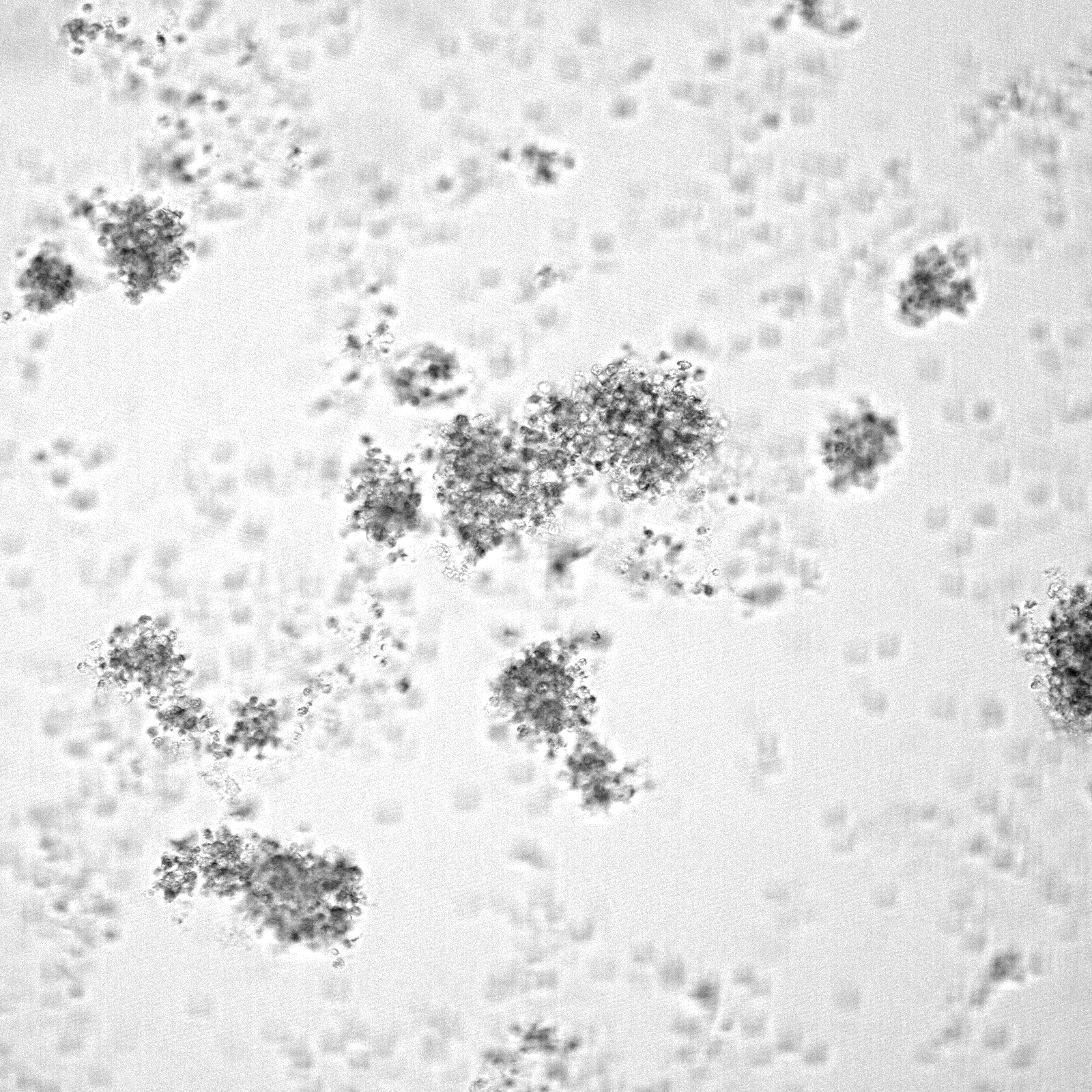

Supplement: Supplementary file 10 — Source Data for Figure 5 [file EMMM-15-e18199-s008.zip › Figure_5/5E/Tumor_D_single_cells_spheroids_D21.tif]

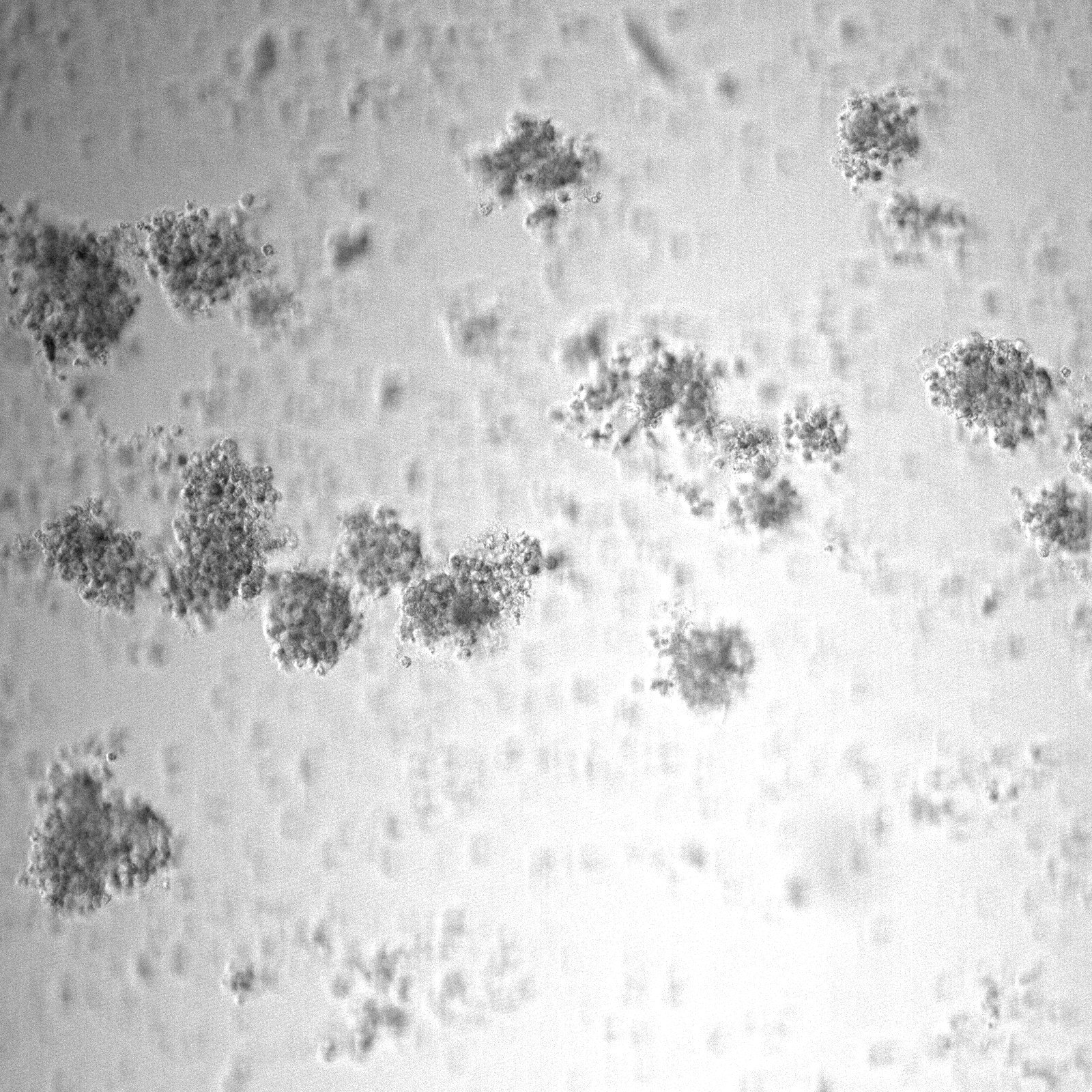

Supplement: Supplementary file 10 — Source Data for Figure 5 [file EMMM-15-e18199-s008.zip › Figure_5/5E/Tumor_D_single_cells_spheroids_D28.tif]

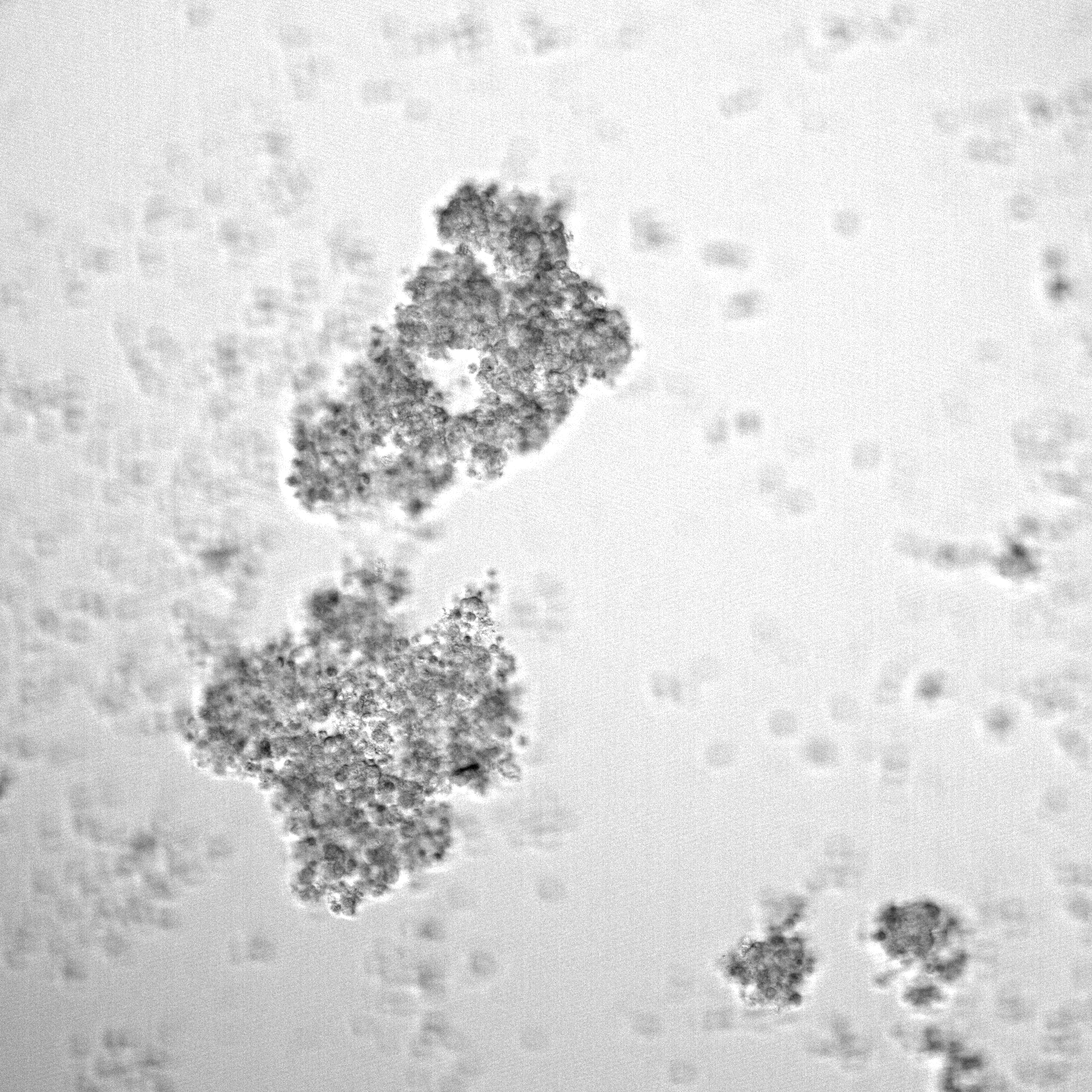

Supplement: Supplementary file 10 — Source Data for Figure 5 [file EMMM-15-e18199-s008.zip › Figure_5/5E/Tumor_D_single_cells_spheroids_D7.tif]

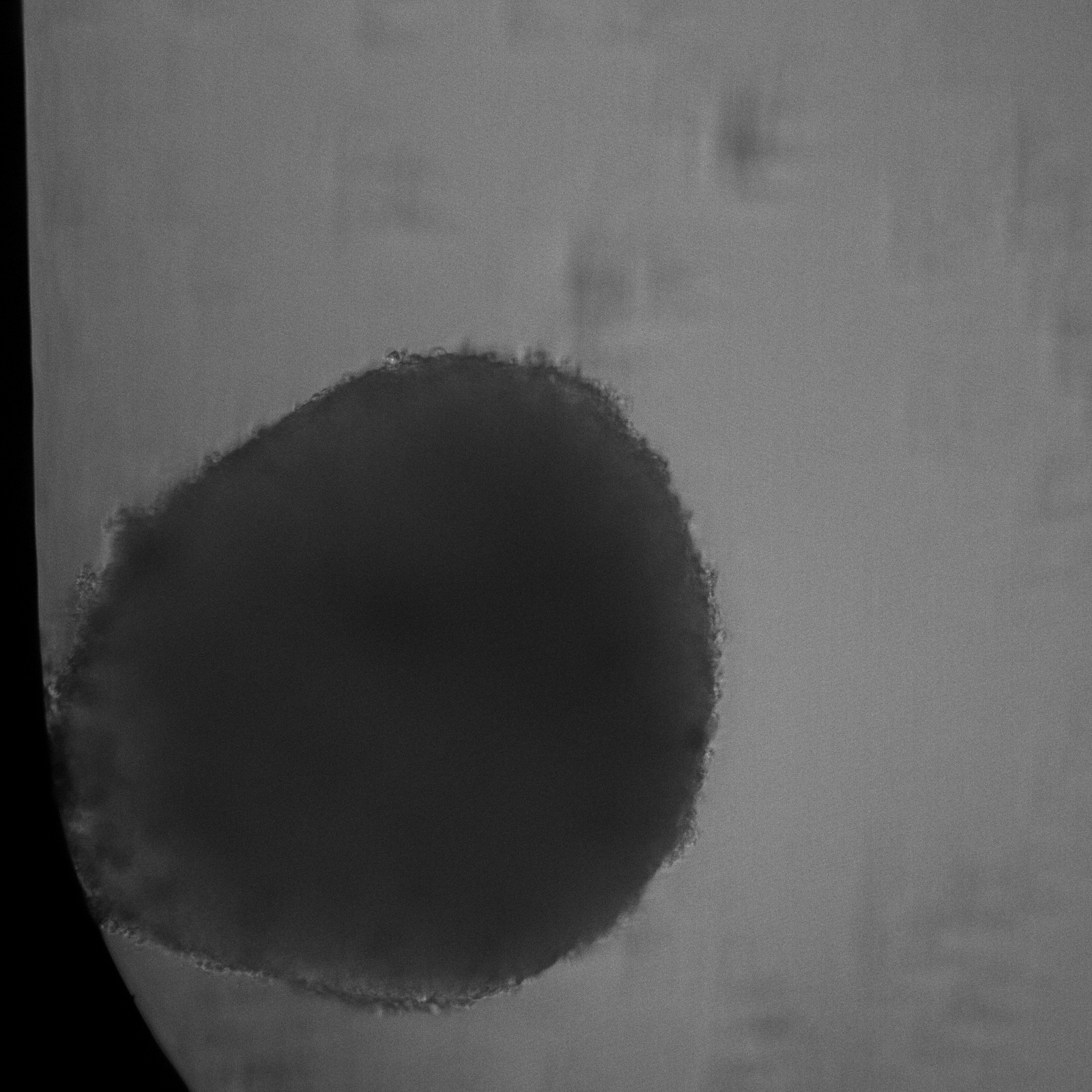

Supplement: Supplementary file 10 — Source Data for Figure 5 [file EMMM-15-e18199-s008.zip › Figure_5/5G/Tumor_A_tumor_pieces_D14.tif]

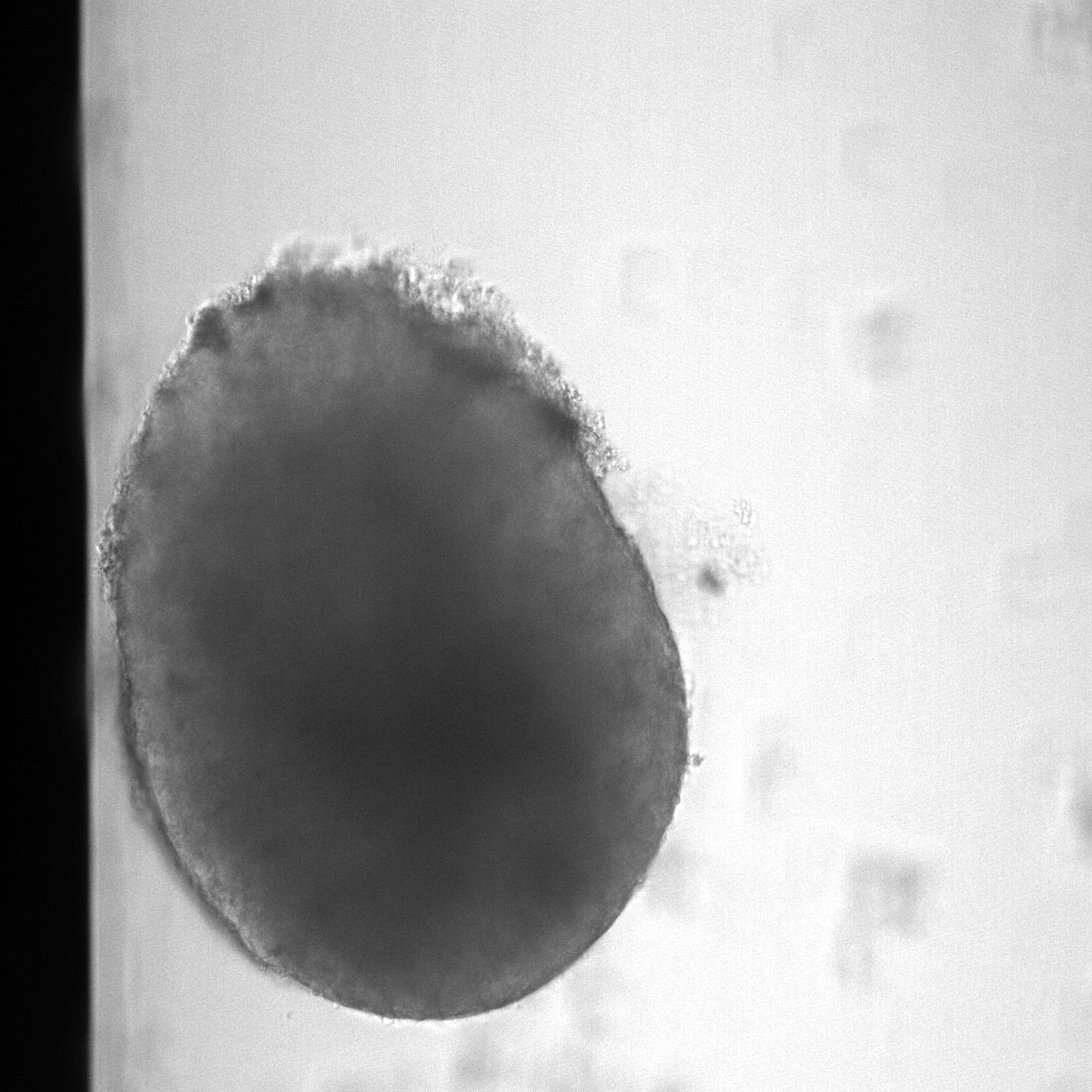

Supplement: Supplementary file 10 — Source Data for Figure 5 [file EMMM-15-e18199-s008.zip › Figure_5/5G/Tumor_A_tumor_pieces_D21.tif]

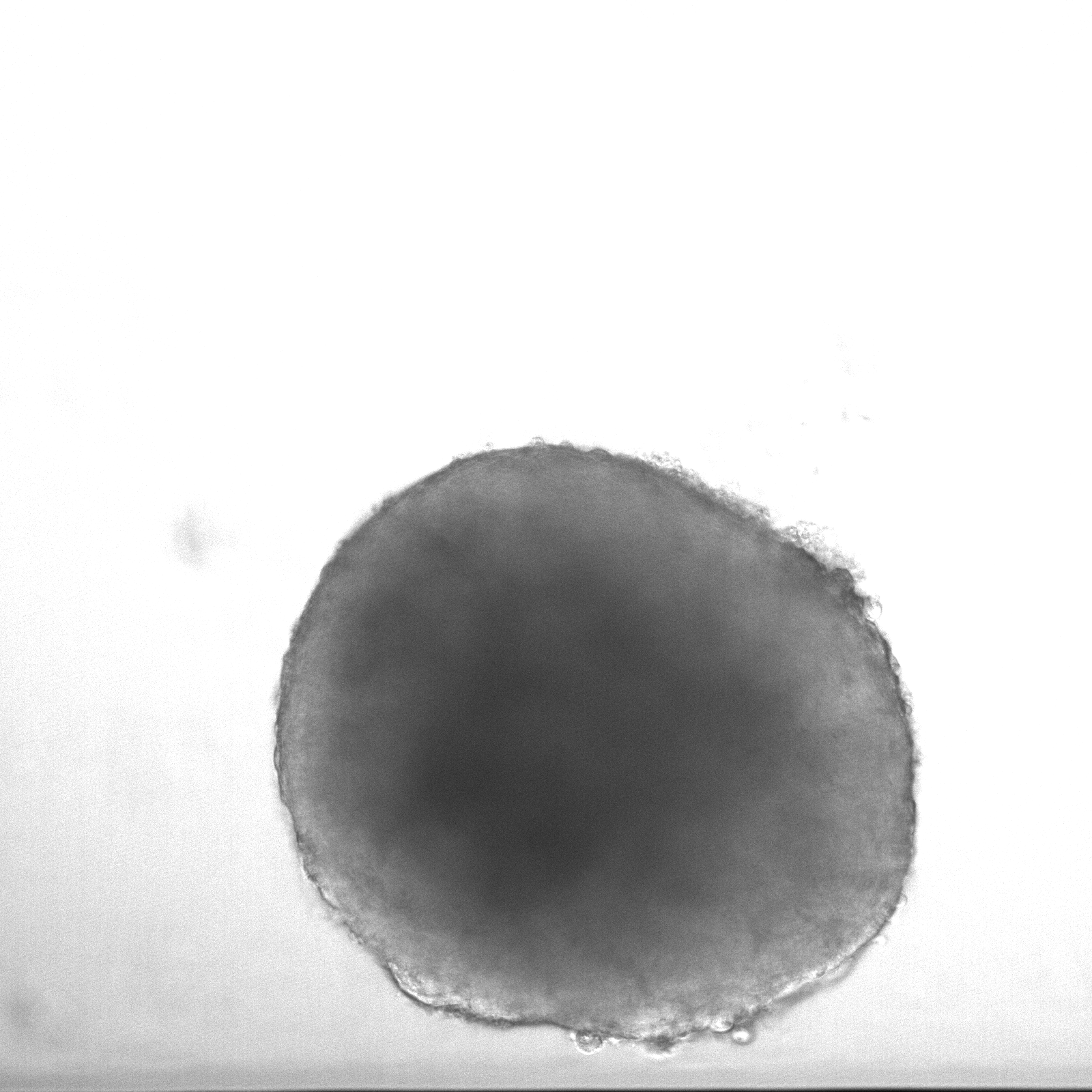

Supplement: Supplementary file 10 — Source Data for Figure 5 [file EMMM-15-e18199-s008.zip › Figure_5/5G/Tumor_A_tumor_pieces_D28.tif]

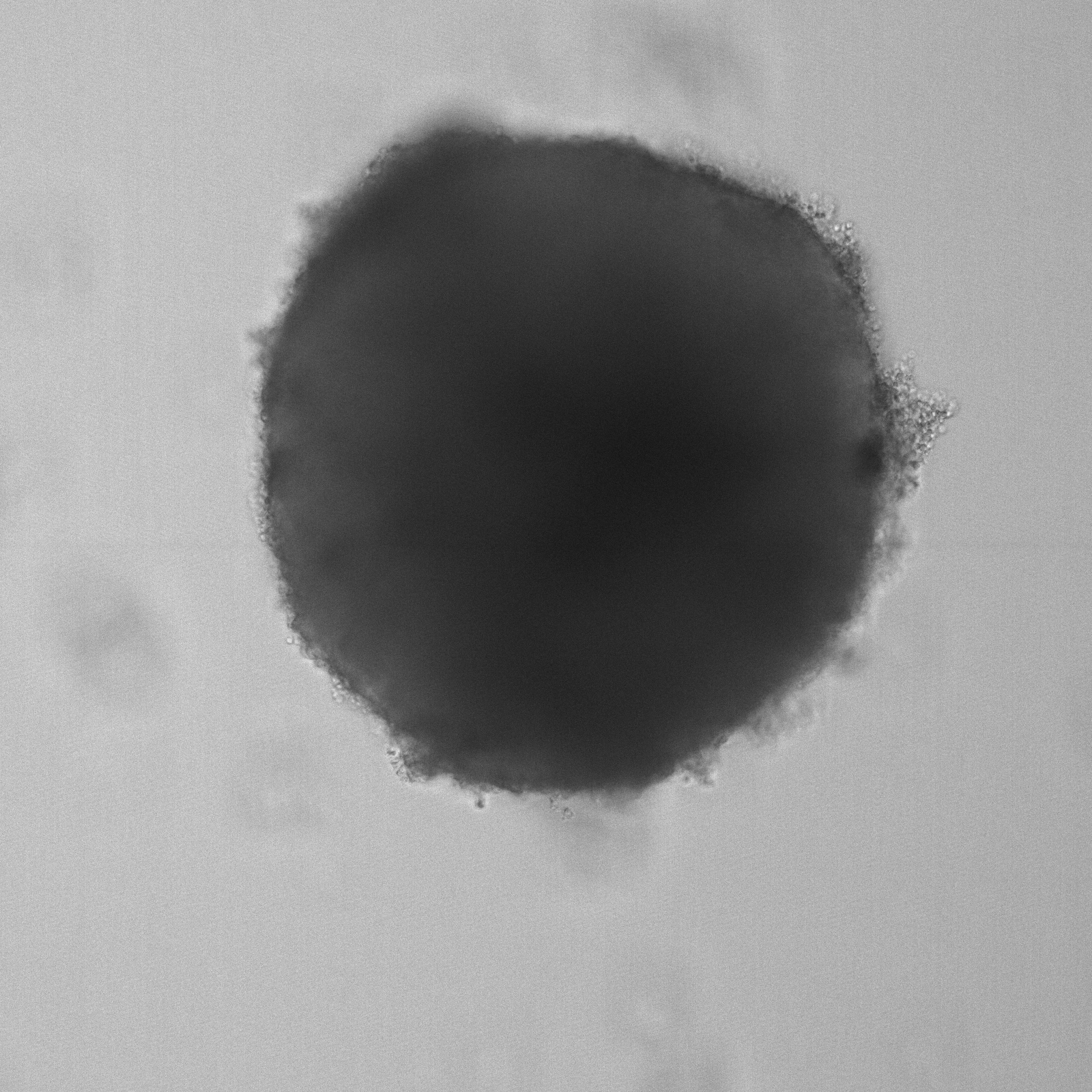

Supplement: Supplementary file 10 — Source Data for Figure 5 [file EMMM-15-e18199-s008.zip › Figure_5/5G/Tumor_A_tumor_pieces_D7.tif]

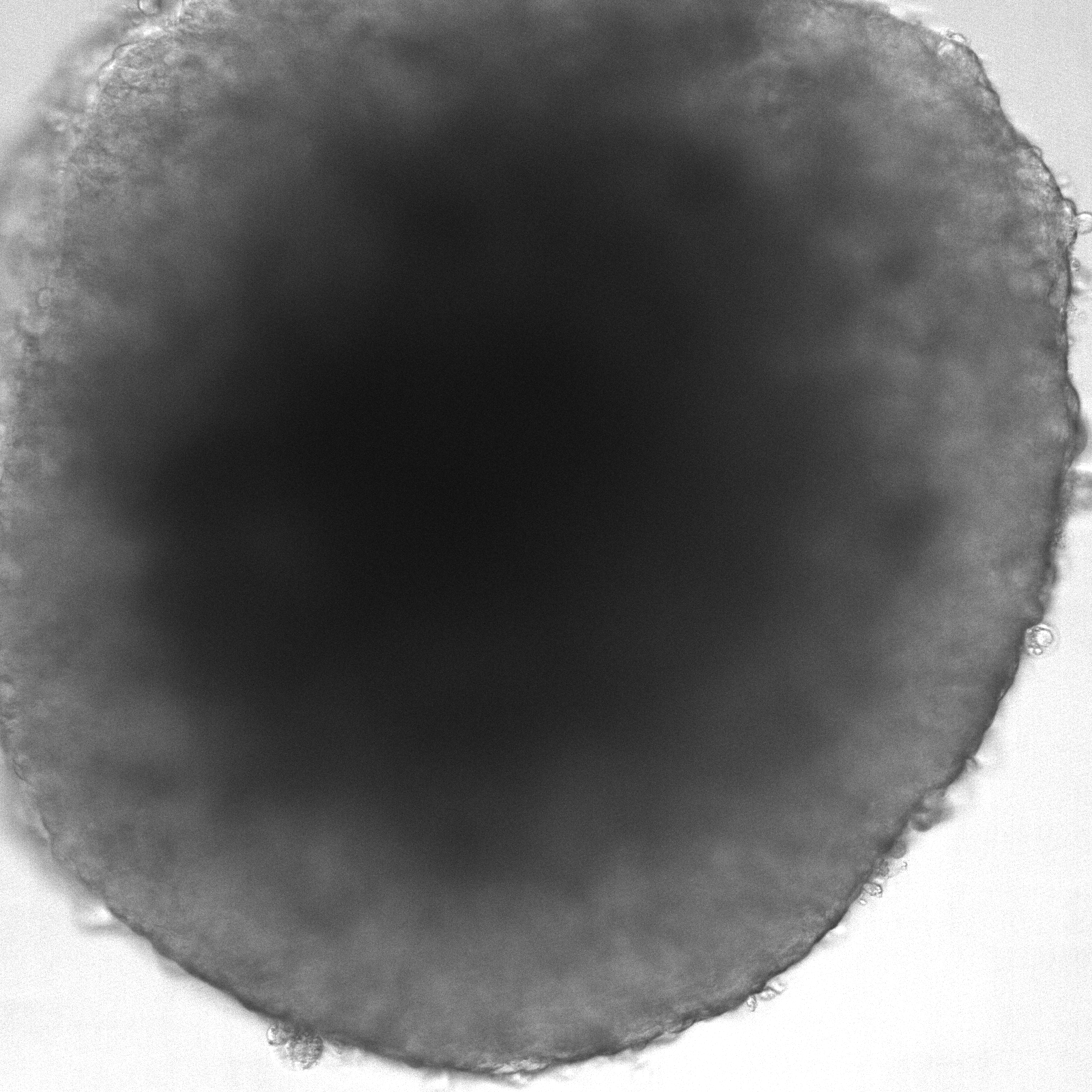

Supplement: Supplementary file 10 — Source Data for Figure 5 [file EMMM-15-e18199-s008.zip › Figure_5/5G/Tumor_B_tumor_pieces_D14.tif]

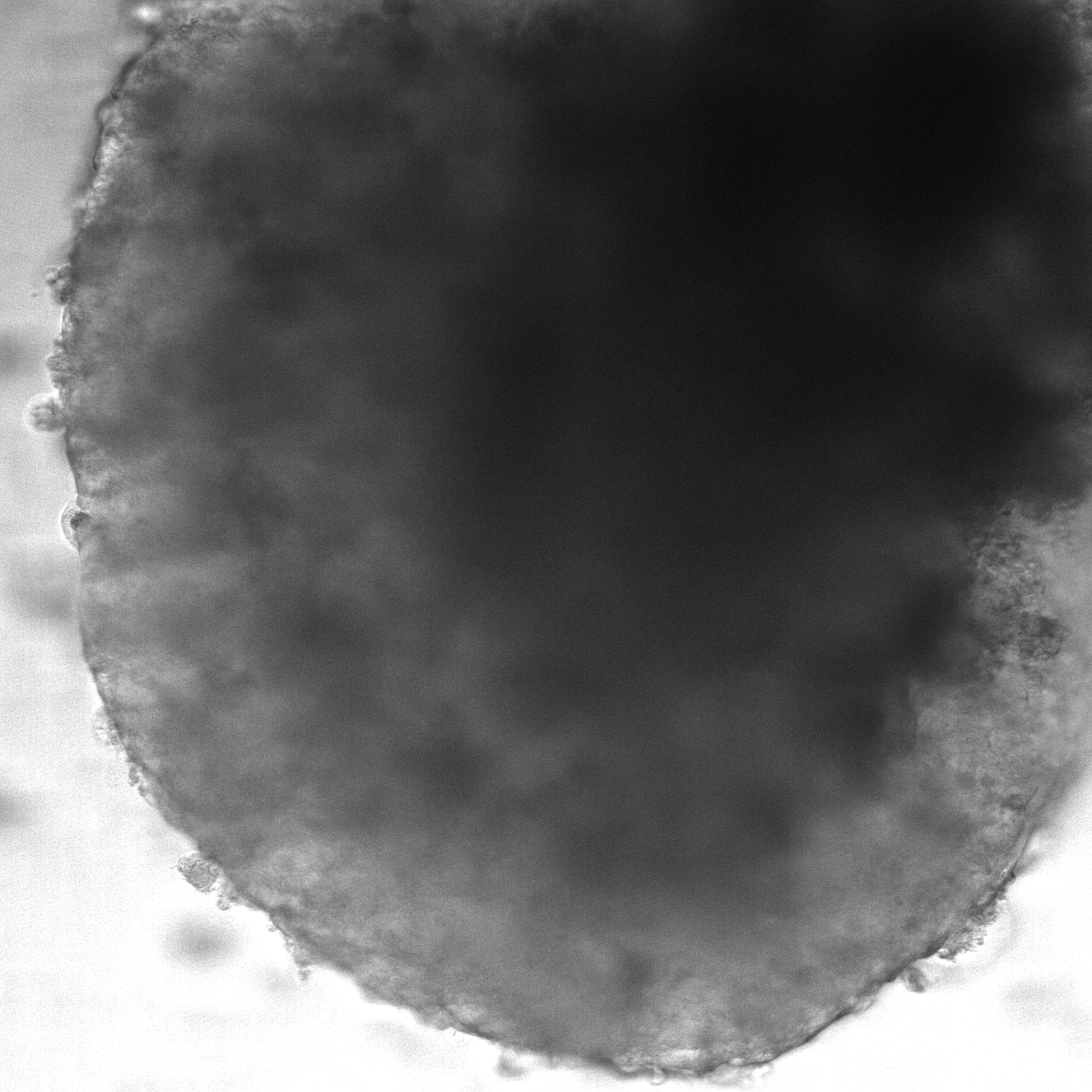

Supplement: Supplementary file 10 — Source Data for Figure 5 [file EMMM-15-e18199-s008.zip › Figure_5/5G/Tumor_B_tumor_pieces_D21.tif]

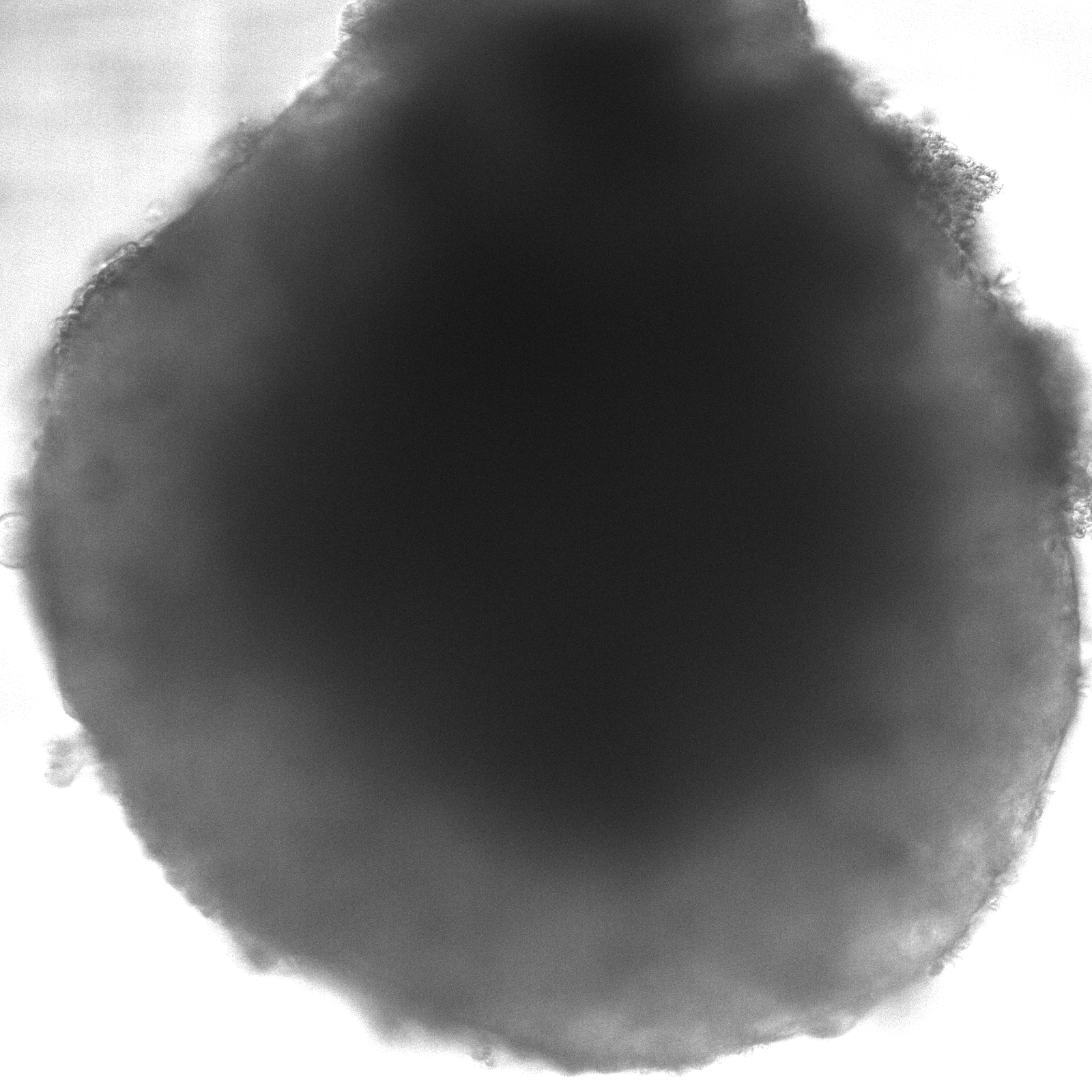

Supplement: Supplementary file 10 — Source Data for Figure 5 [file EMMM-15-e18199-s008.zip › Figure_5/5G/Tumor_B_tumor_pieces_D28.tif]

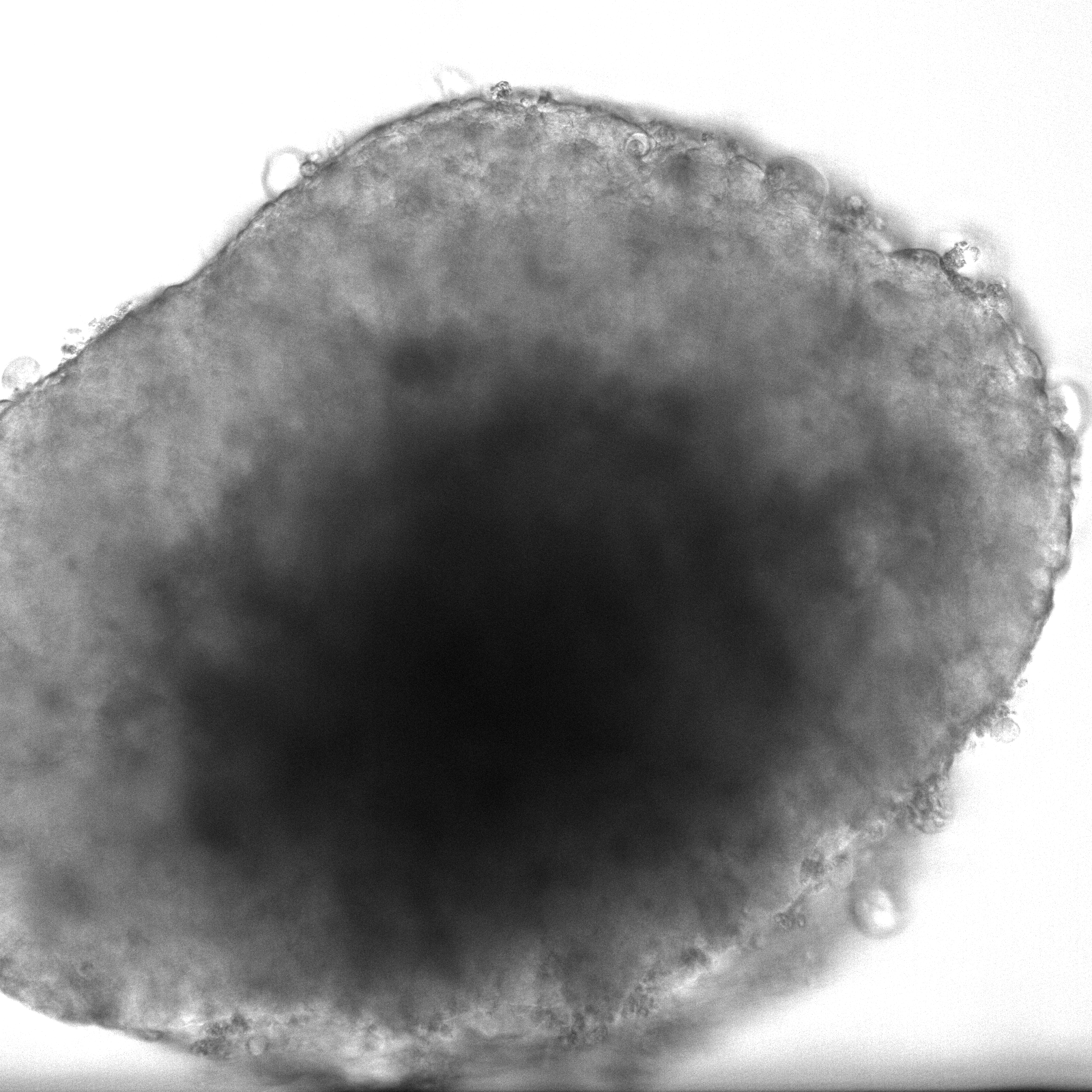

Supplement: Supplementary file 10 — Source Data for Figure 5 [file EMMM-15-e18199-s008.zip › Figure_5/5G/Tumor_B_tumor_pieces_D7.tif]

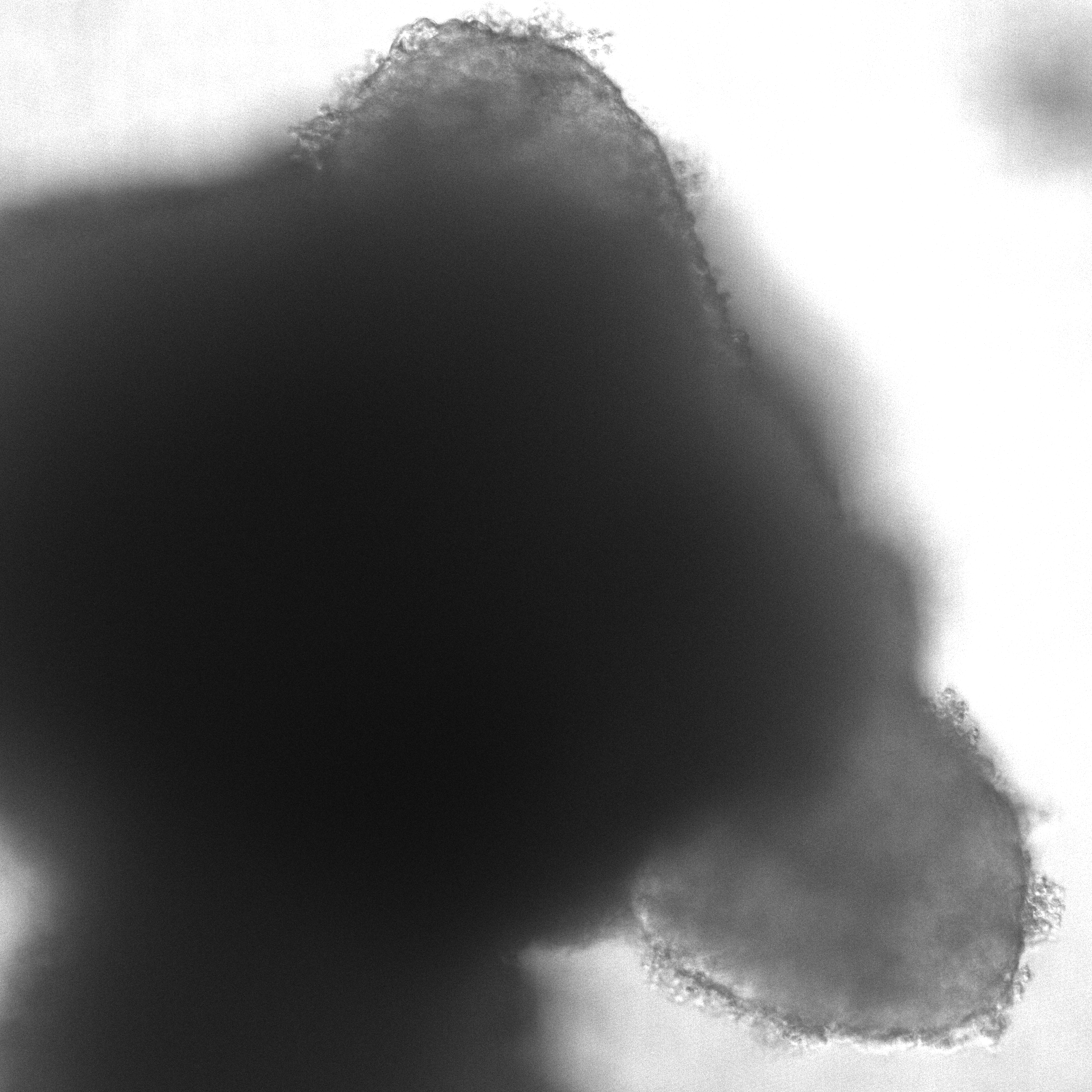

Supplement: Supplementary file 10 — Source Data for Figure 5 [file EMMM-15-e18199-s008.zip › Figure_5/5H/Tumor_C_tumor_pieces_D14.tif]

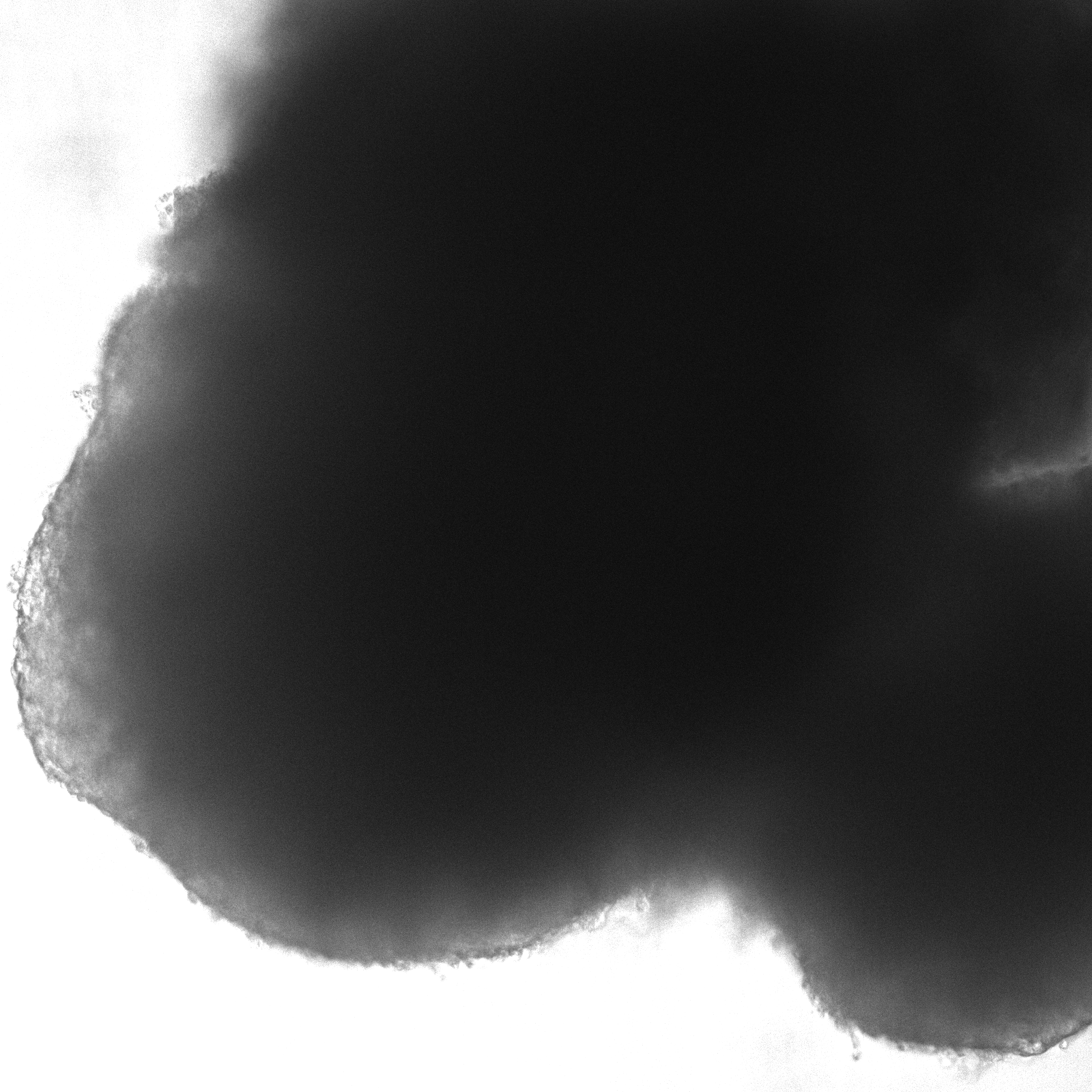

Supplement: Supplementary file 10 — Source Data for Figure 5 [file EMMM-15-e18199-s008.zip › Figure_5/5H/Tumor_C_tumor_pieces_D21.tif]

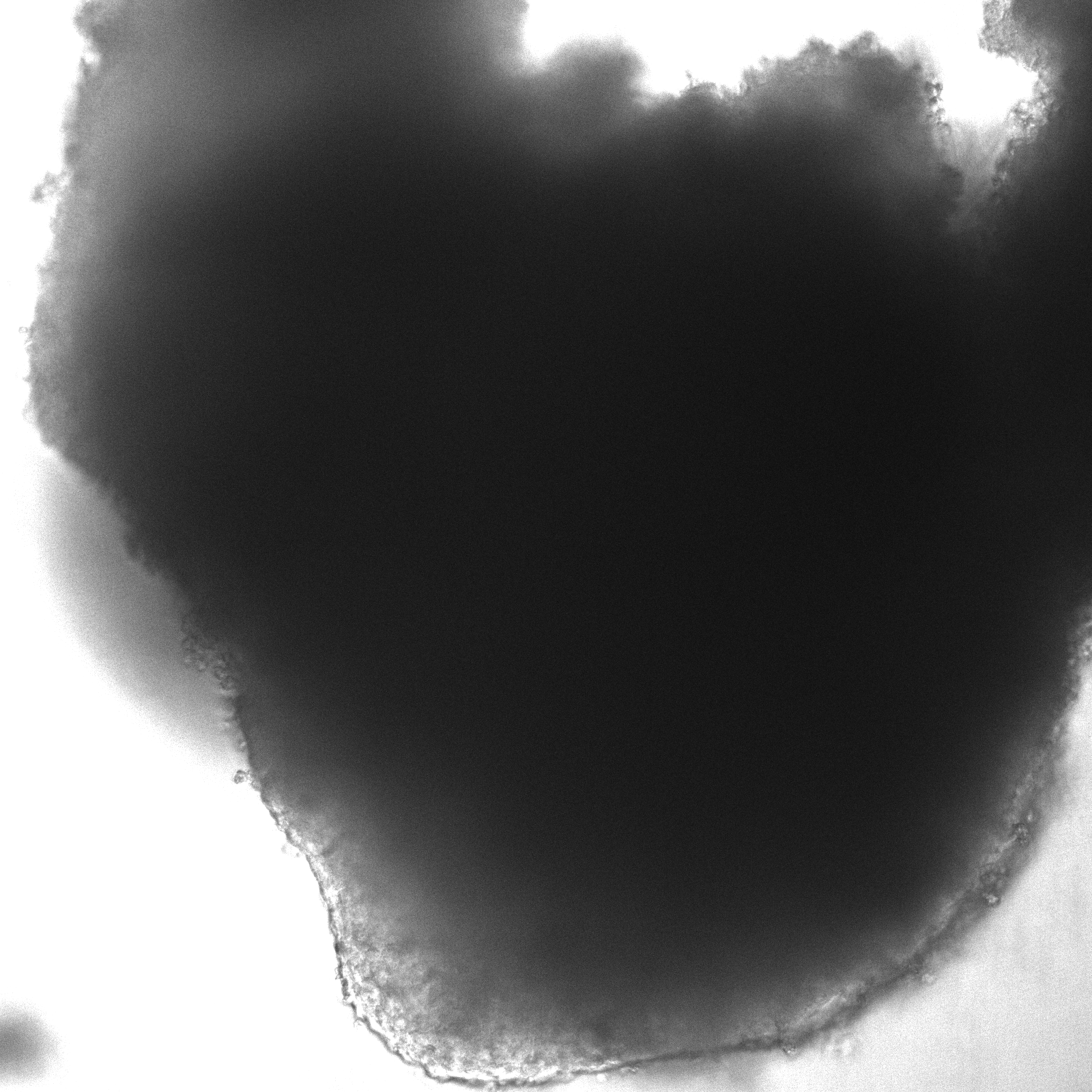

Supplement: Supplementary file 10 — Source Data for Figure 5 [file EMMM-15-e18199-s008.zip › Figure_5/5H/Tumor_C_tumor_pieces_D28.tif]

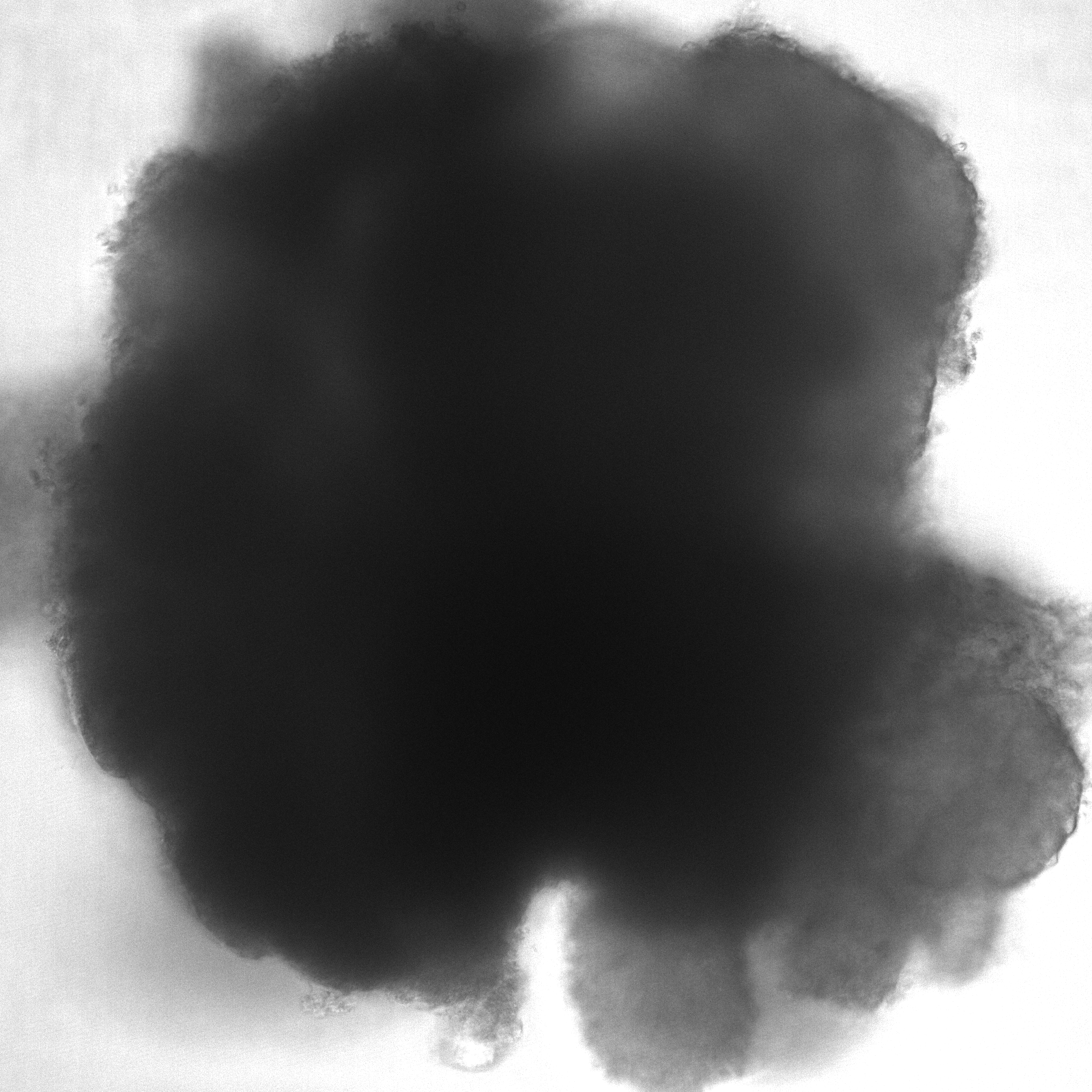

Supplement: Supplementary file 10 — Source Data for Figure 5 [file EMMM-15-e18199-s008.zip › Figure_5/5H/Tumor_C_tumor_pieces_D7.tif]

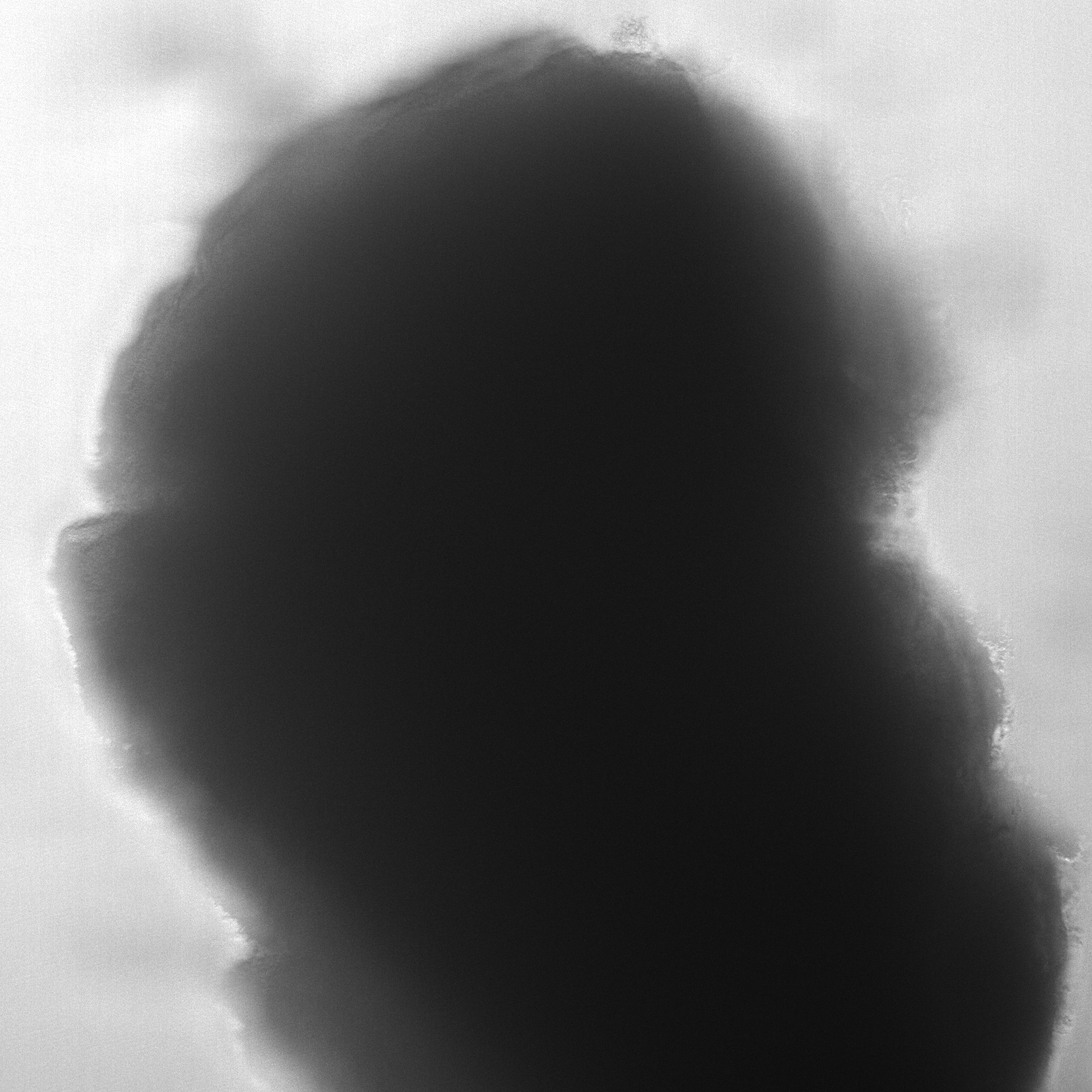

Supplement: Supplementary file 10 — Source Data for Figure 5 [file EMMM-15-e18199-s008.zip › Figure_5/5H/Tumor_D_tumor_pieces_D14.tif]

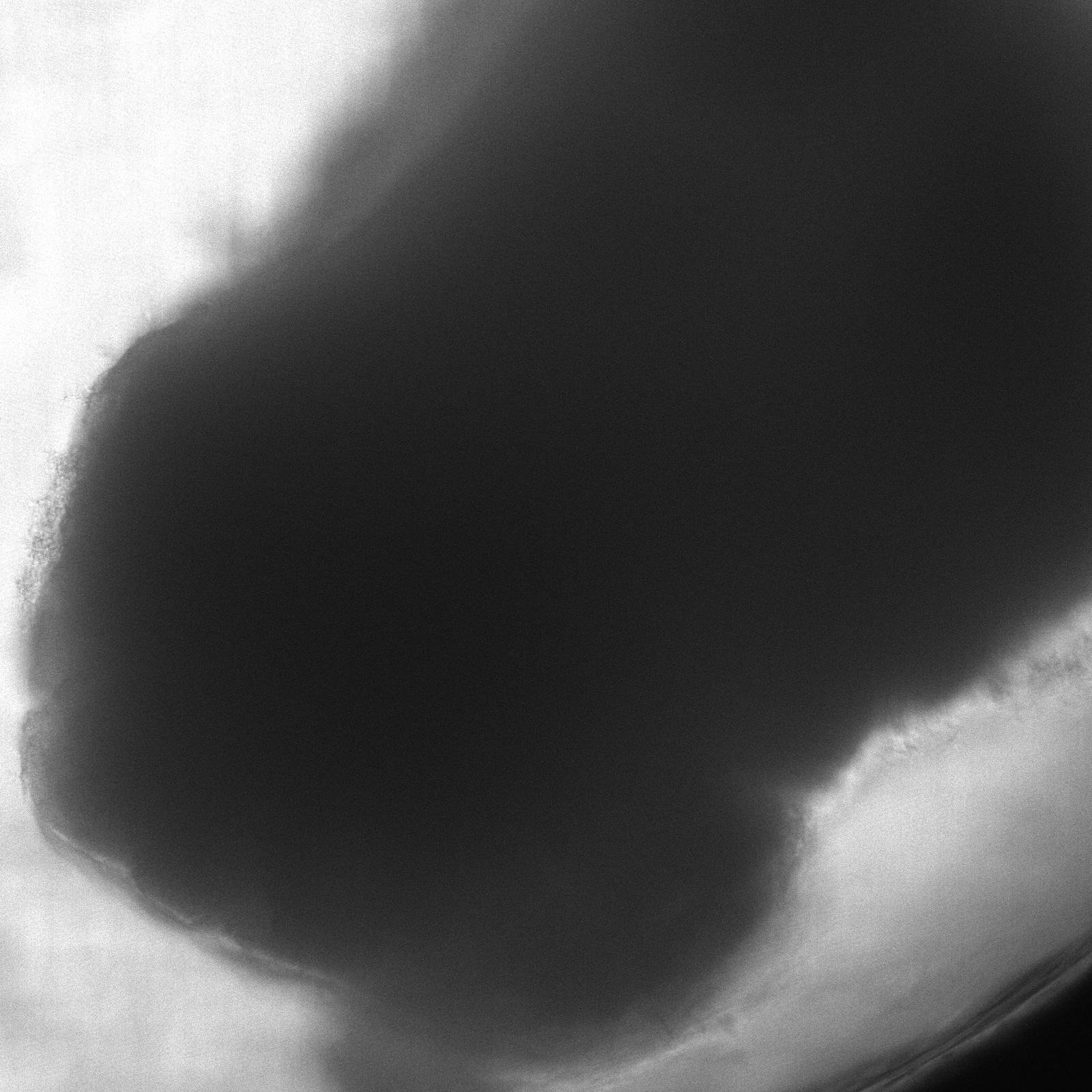

Supplement: Supplementary file 10 — Source Data for Figure 5 [file EMMM-15-e18199-s008.zip › Figure_5/5H/Tumor_D_tumor_pieces_D21.tif]

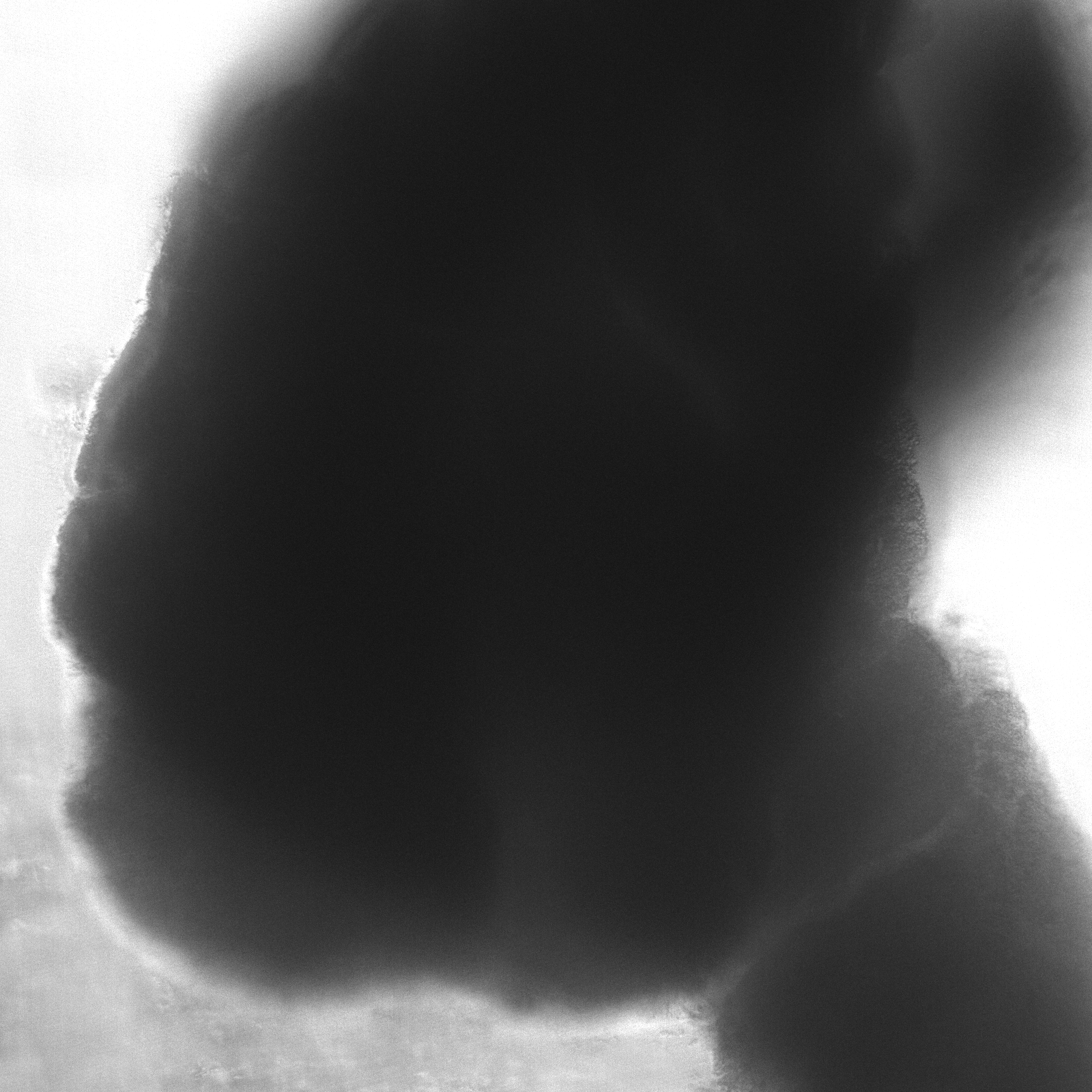

Supplement: Supplementary file 10 — Source Data for Figure 5 [file EMMM-15-e18199-s008.zip › Figure_5/5H/Tumor_D_tumor_pieces_D28.tif]

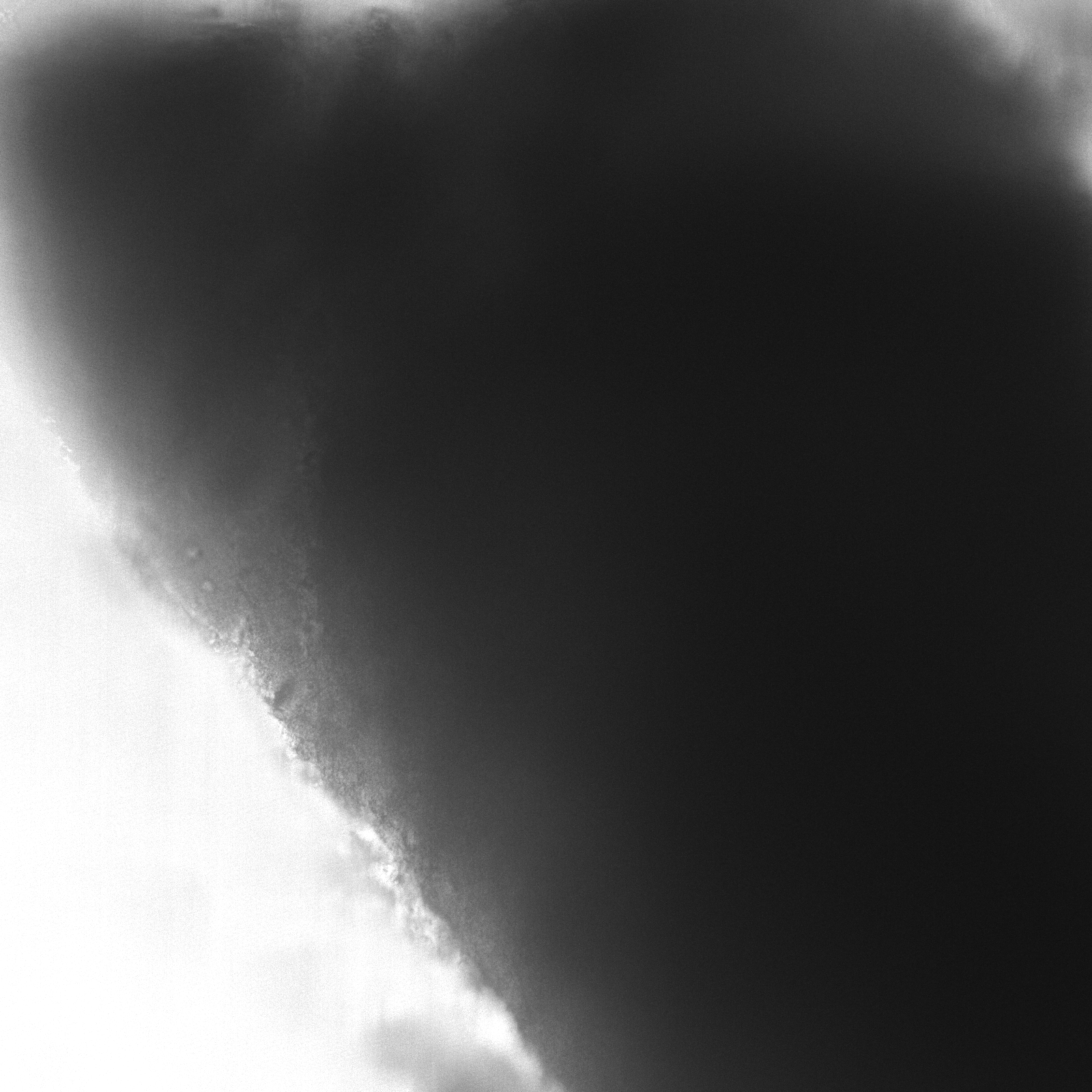

Supplement: Supplementary file 10 — Source Data for Figure 5 [file EMMM-15-e18199-s008.zip › Figure_5/5H/Tumor_D_tumor_pieces_D7.tif]

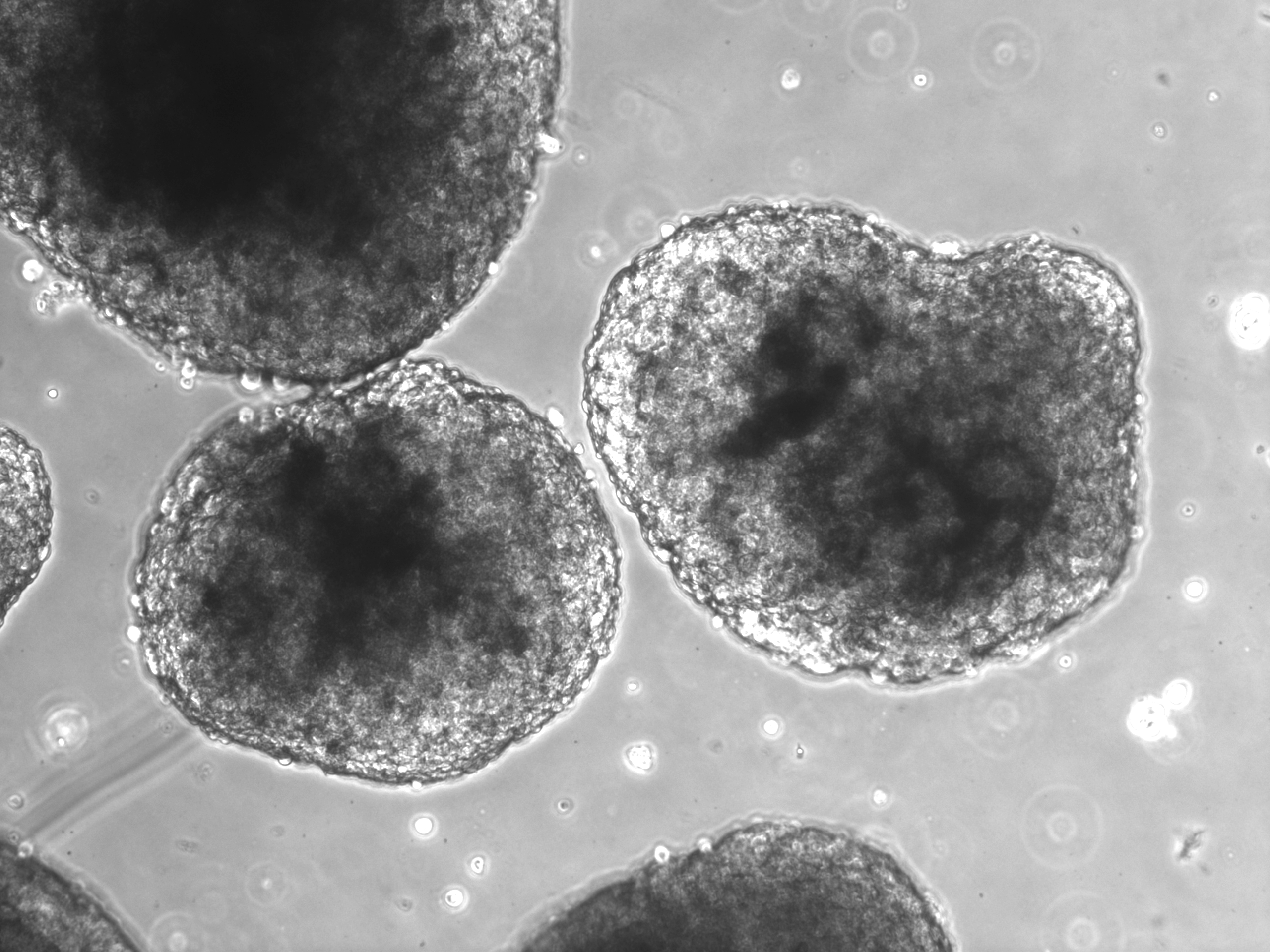

Supplement: Supplementary file 10 — Source Data for Figure 5 [file EMMM-15-e18199-s008.zip › Figure_5/5J/Tumor_B_tumor_pieces_susp_D120a.tif]

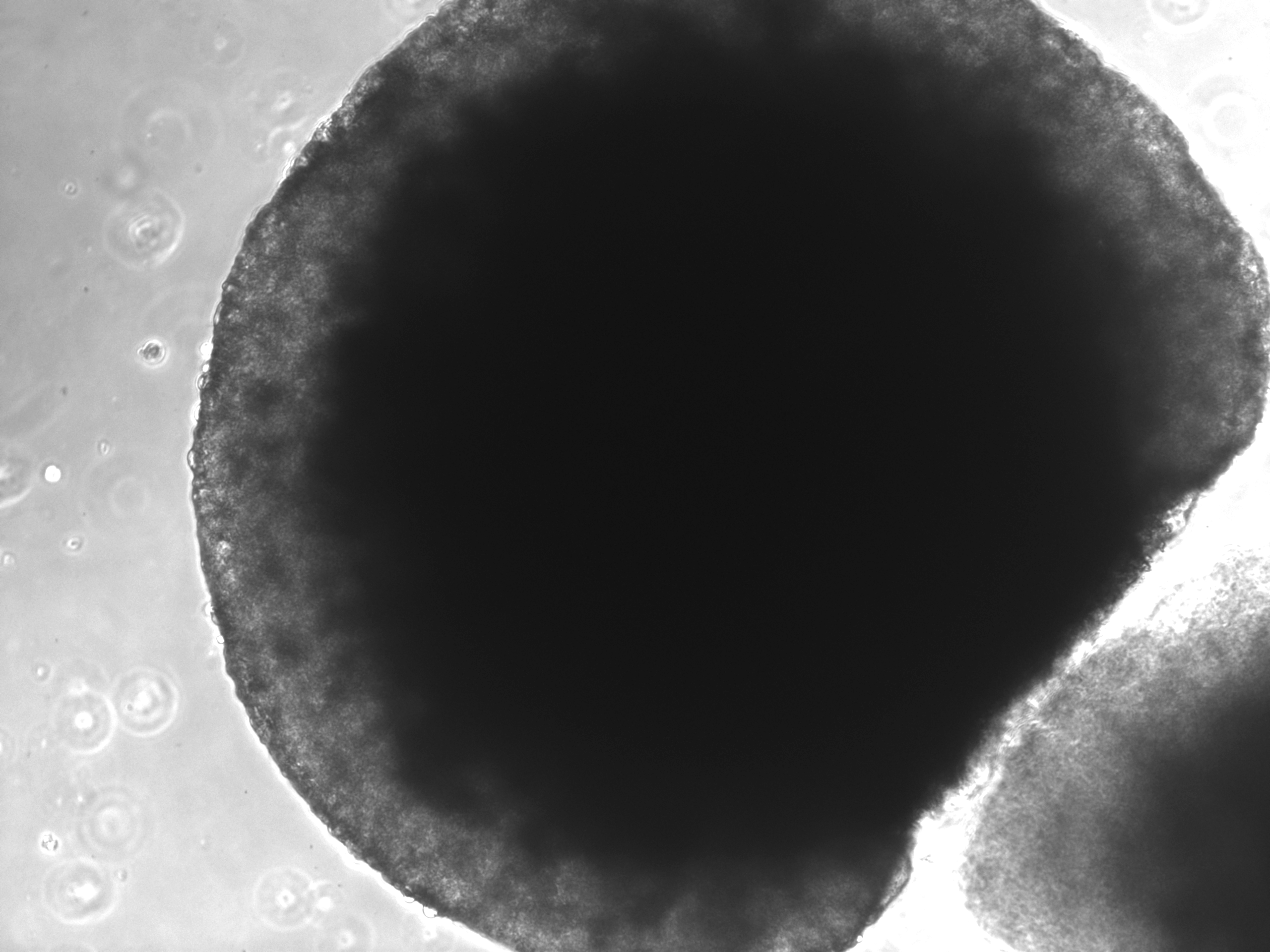

Supplement: Supplementary file 10 — Source Data for Figure 5 [file EMMM-15-e18199-s008.zip › Figure_5/5J/Tumor_B_tumor_pieces_susp_D120b.tif]

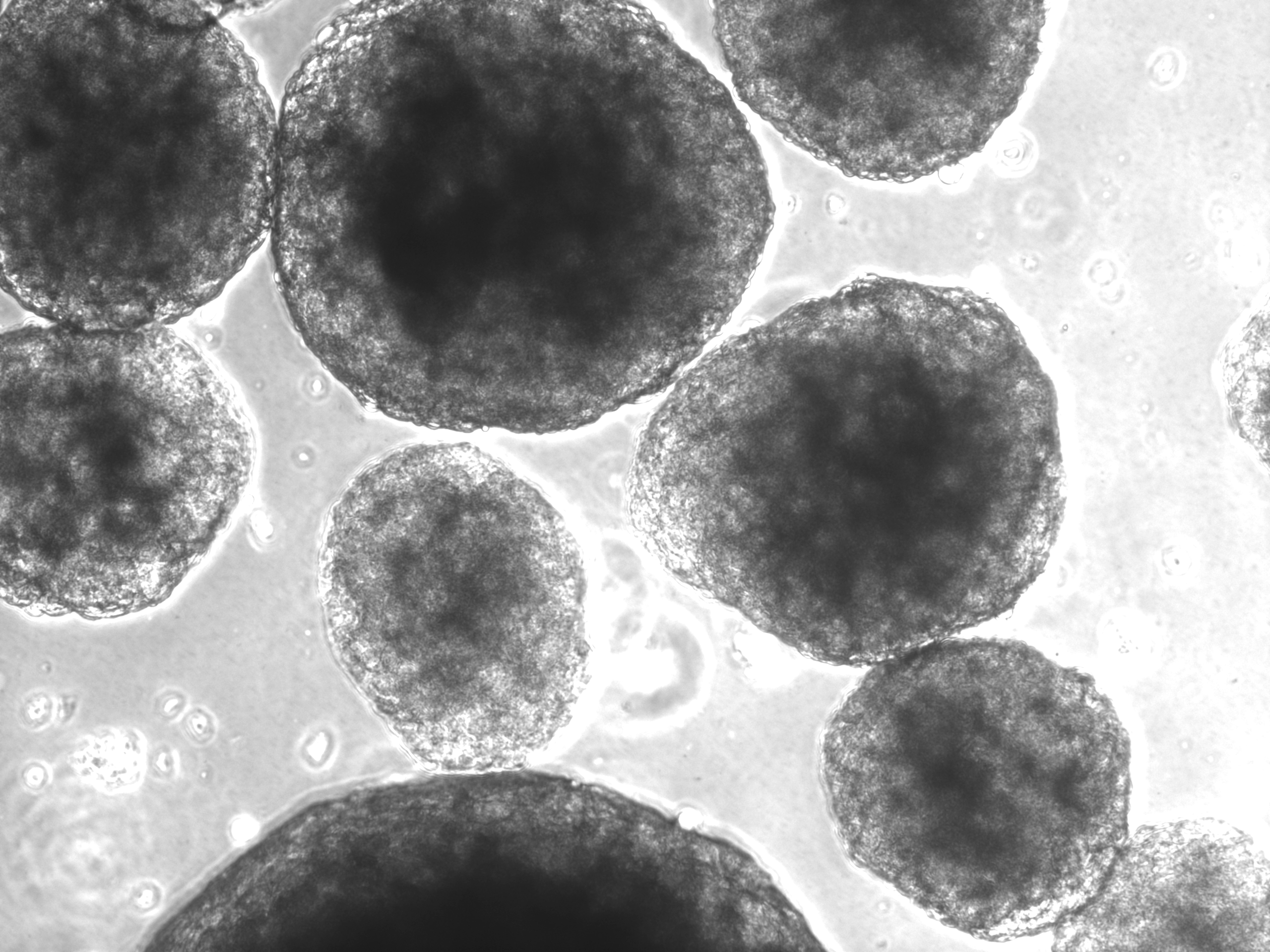

Supplement: Supplementary file 10 — Source Data for Figure 5 [file EMMM-15-e18199-s008.zip › Figure_5/5J/Tumor_B_tumor_pieces_susp_D20a.tif]

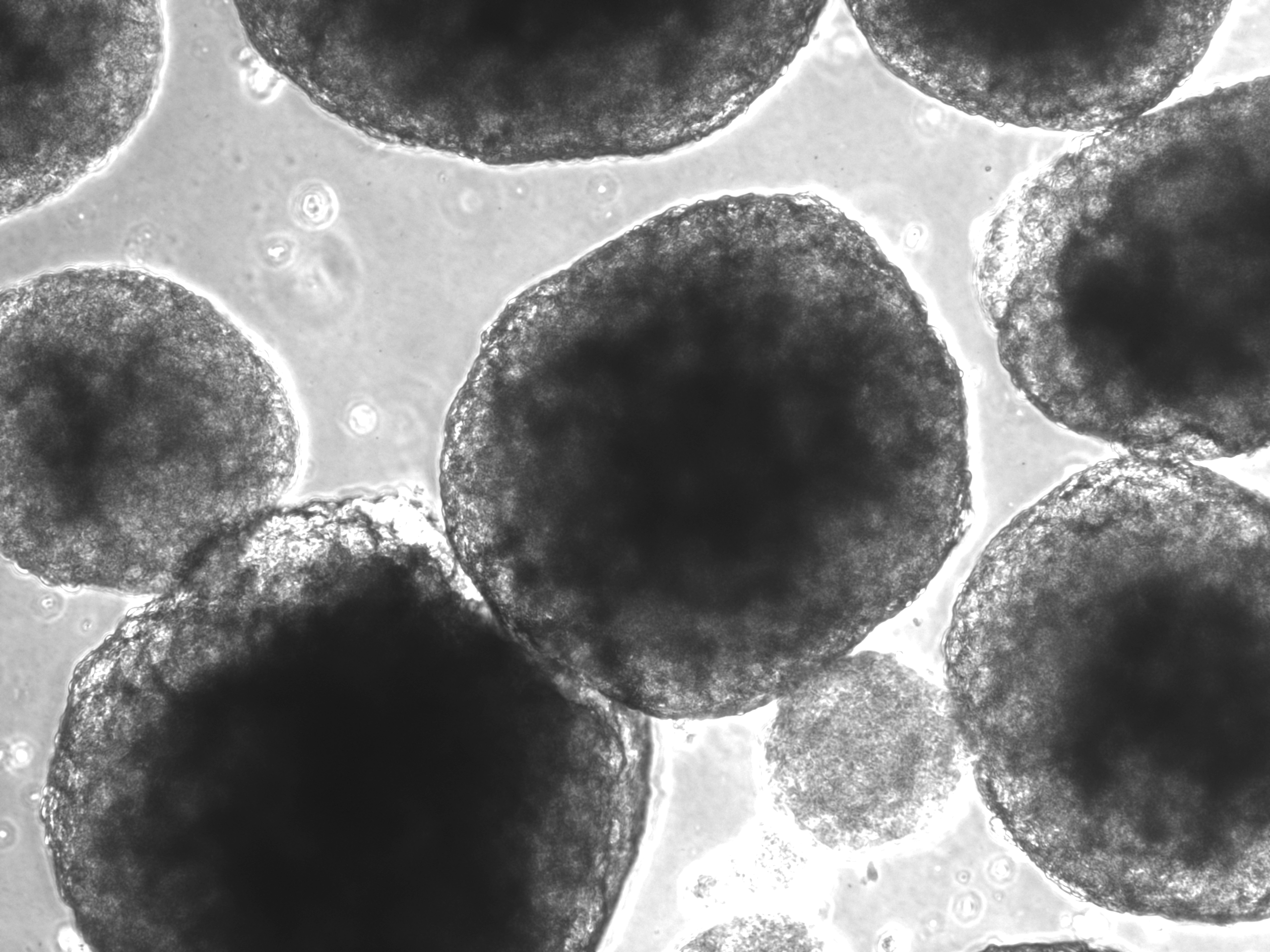

Supplement: Supplementary file 10 — Source Data for Figure 5 [file EMMM-15-e18199-s008.zip › Figure_5/5J/Tumor_B_tumor_pieces_susp_D20b.tif]

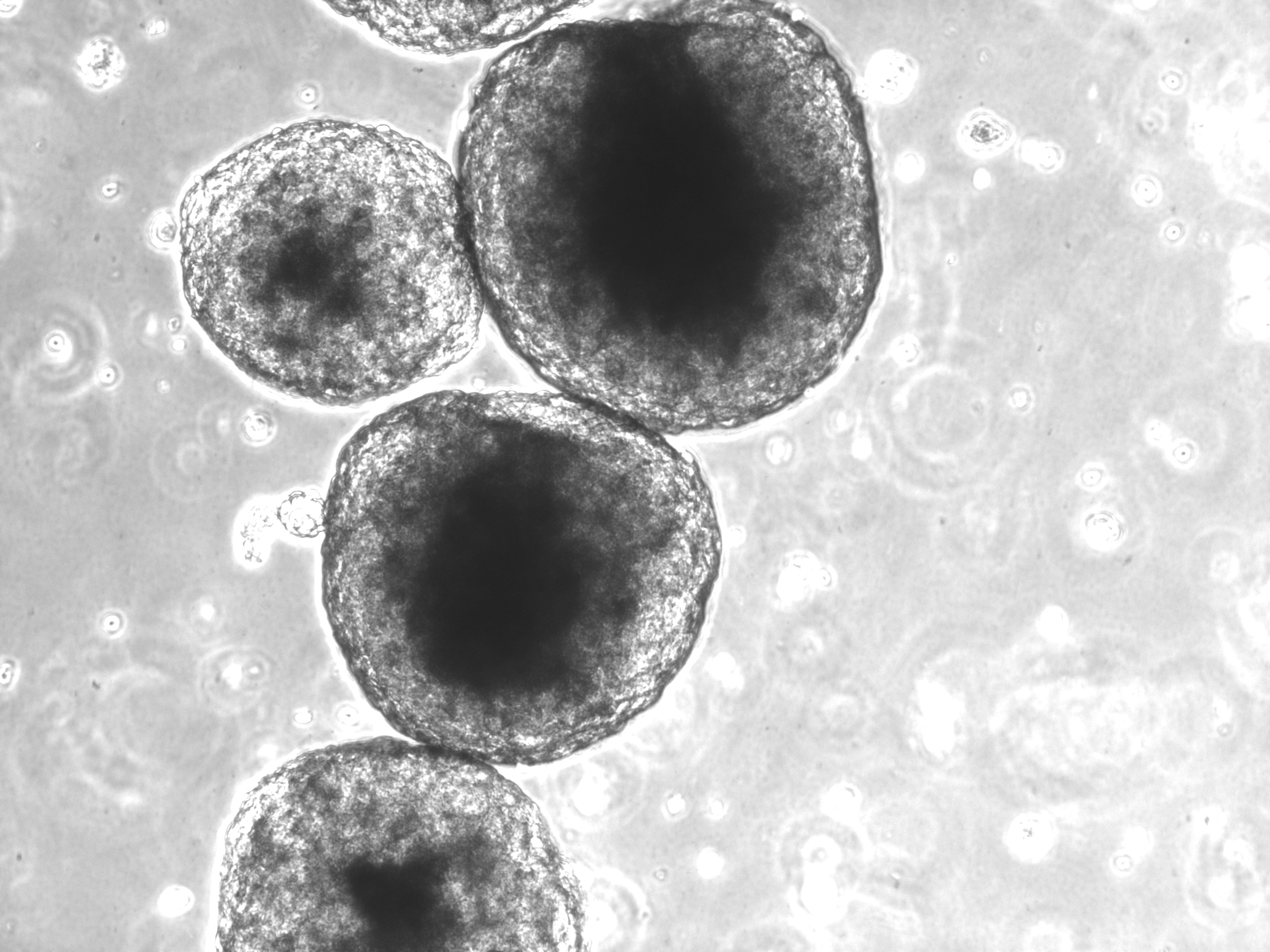

Supplement: Supplementary file 10 — Source Data for Figure 5 [file EMMM-15-e18199-s008.zip › Figure_5/5J/Tumor_B_tumor_pieces_susp_D30a.tif]

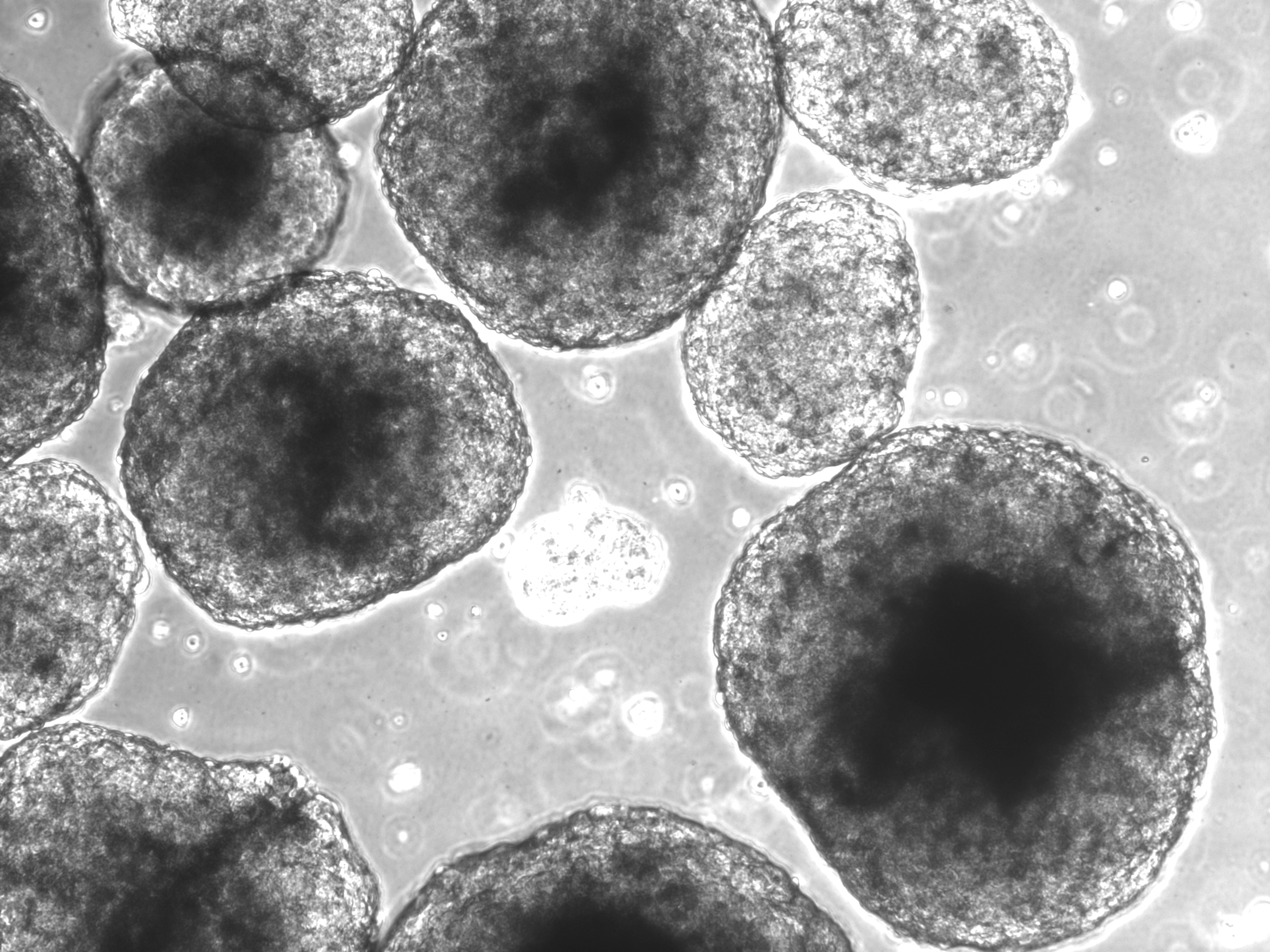

Supplement: Supplementary file 10 — Source Data for Figure 5 [file EMMM-15-e18199-s008.zip › Figure_5/5J/Tumor_B_tumor_pieces_susp_D30b.tif]

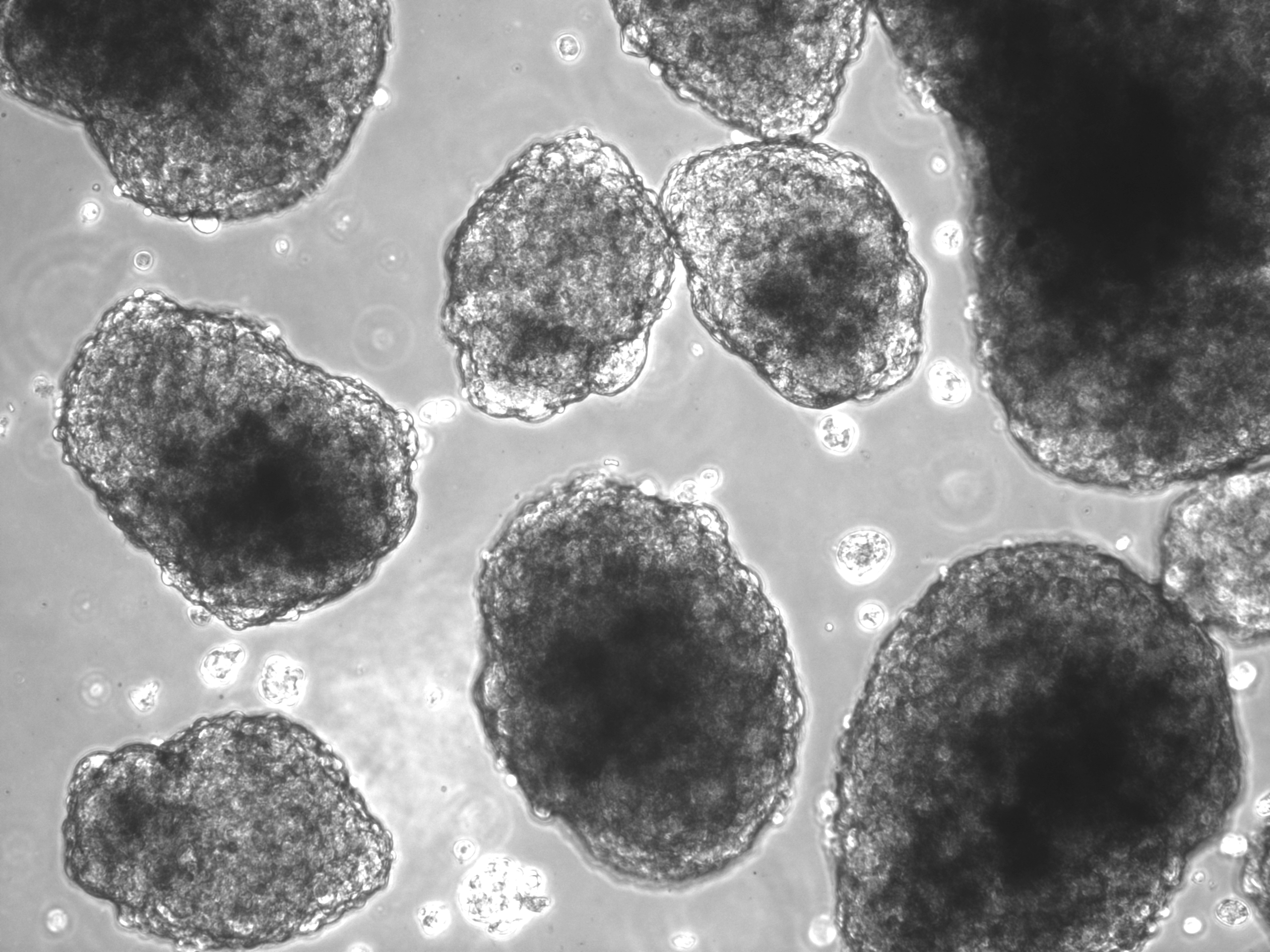

Supplement: Supplementary file 10 — Source Data for Figure 5 [file EMMM-15-e18199-s008.zip › Figure_5/5J/Tumor_B_tumor_pieces_susp_D61a.tif]

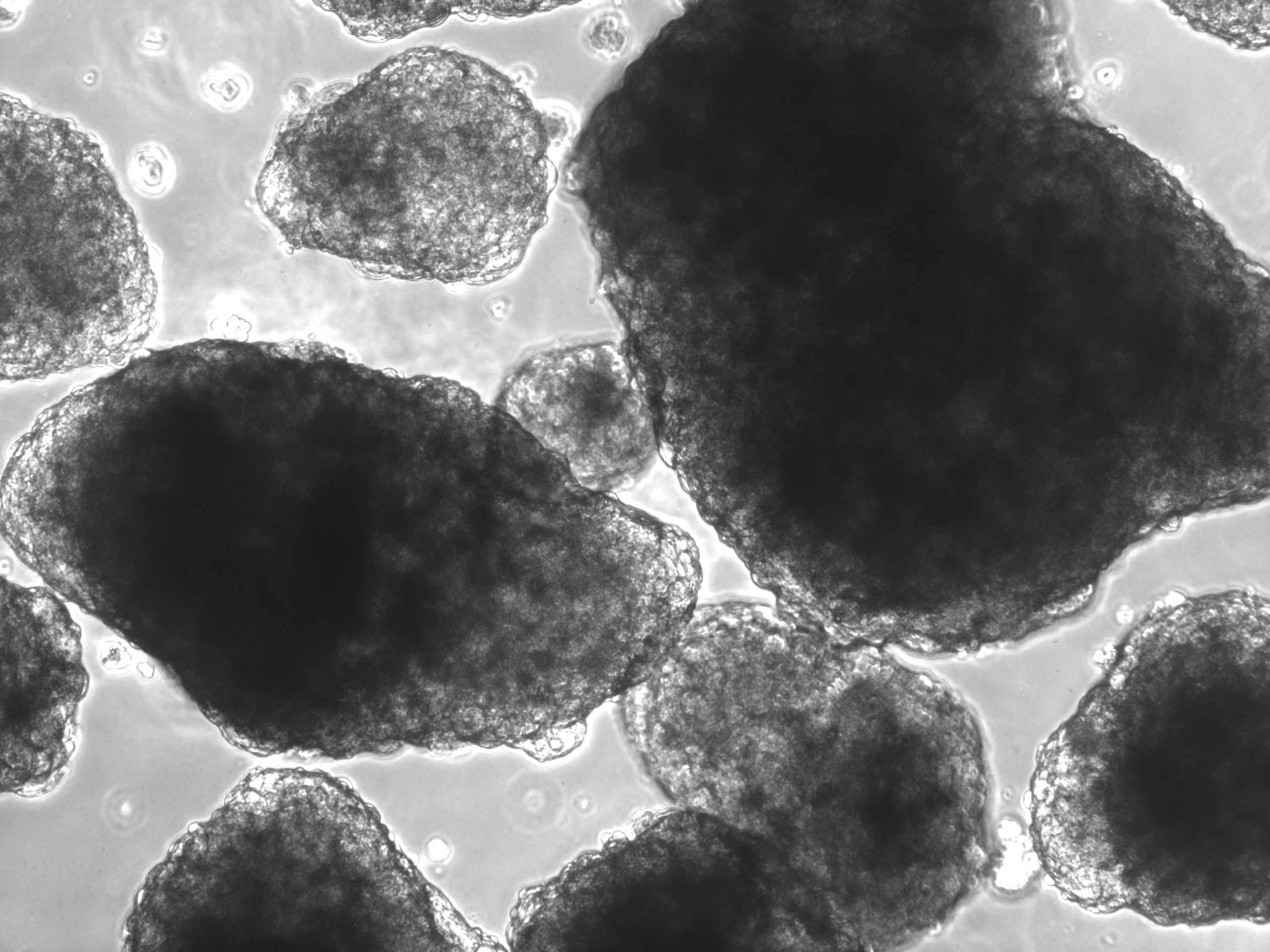

Supplement: Supplementary file 10 — Source Data for Figure 5 [file EMMM-15-e18199-s008.zip › Figure_5/5J/Tumor_B_tumor_pieces_susp_D61b.tif]

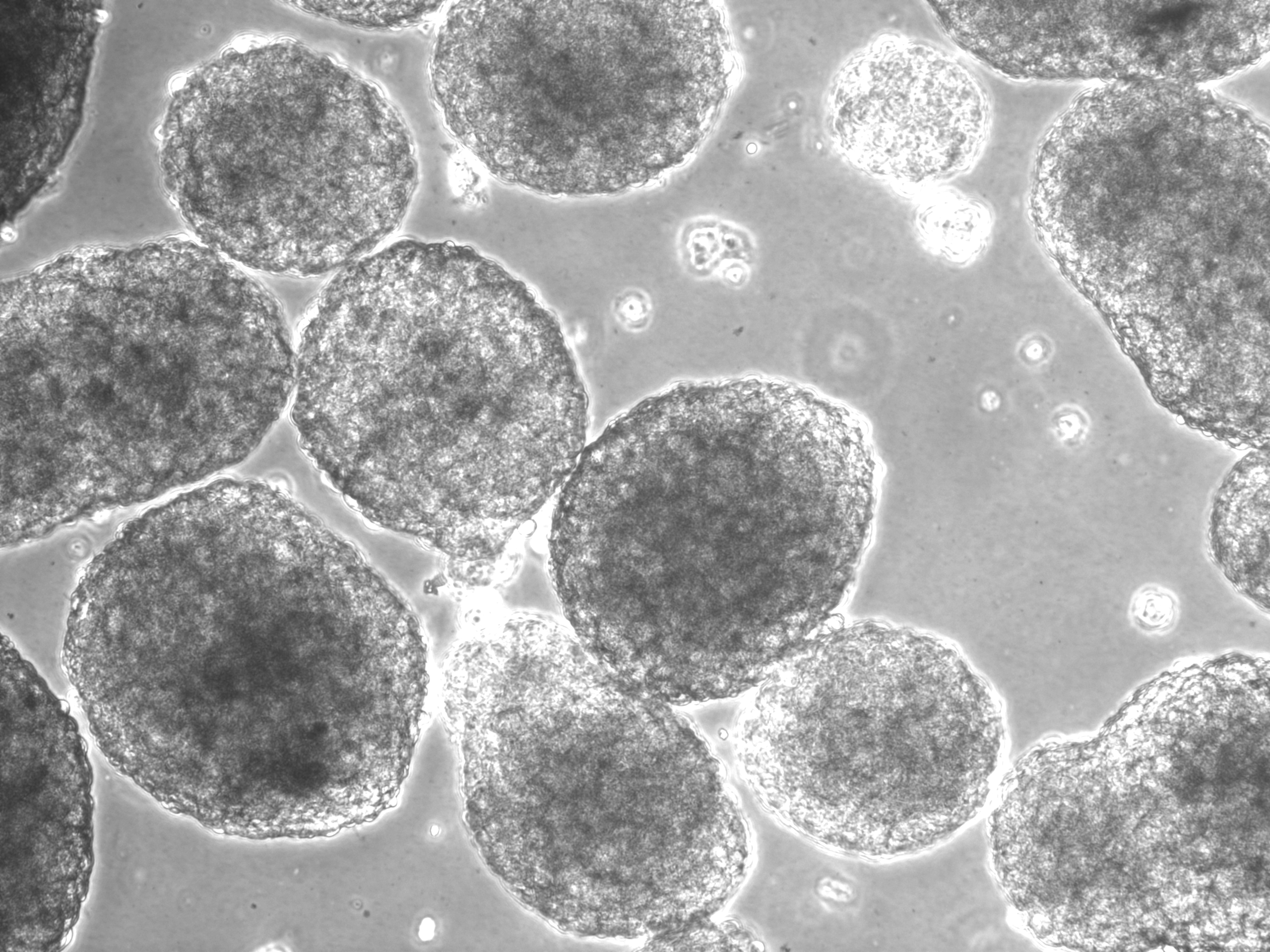

Supplement: Supplementary file 10 — Source Data for Figure 5 [file EMMM-15-e18199-s008.zip › Figure_5/5J/Tumor_B_tumor_pieces_susp_D9a.tif]

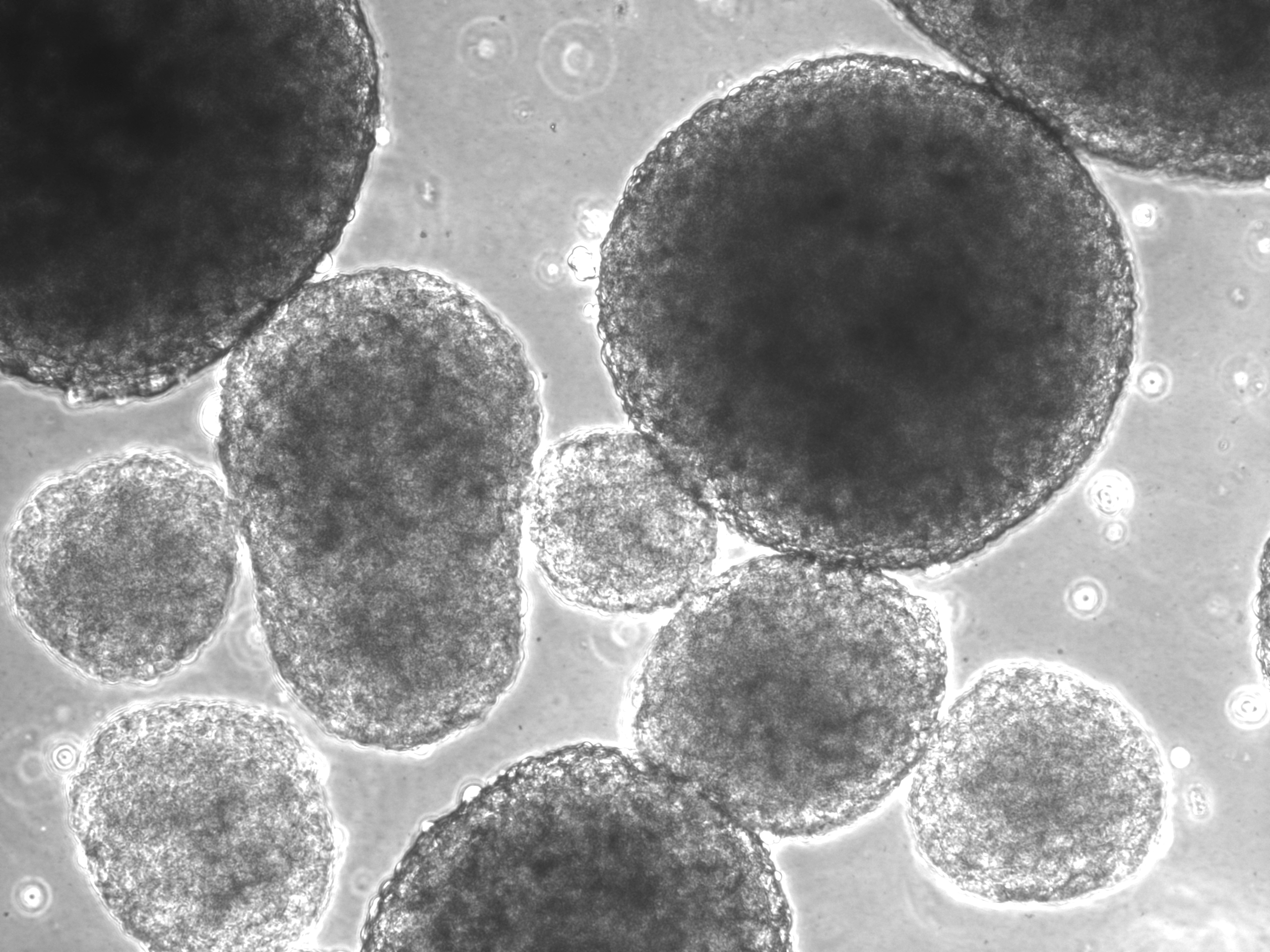

Supplement: Supplementary file 10 — Source Data for Figure 5 [file EMMM-15-e18199-s008.zip › Figure_5/5J/Tumor_B_tumor_pieces_susp_D9b.tif]

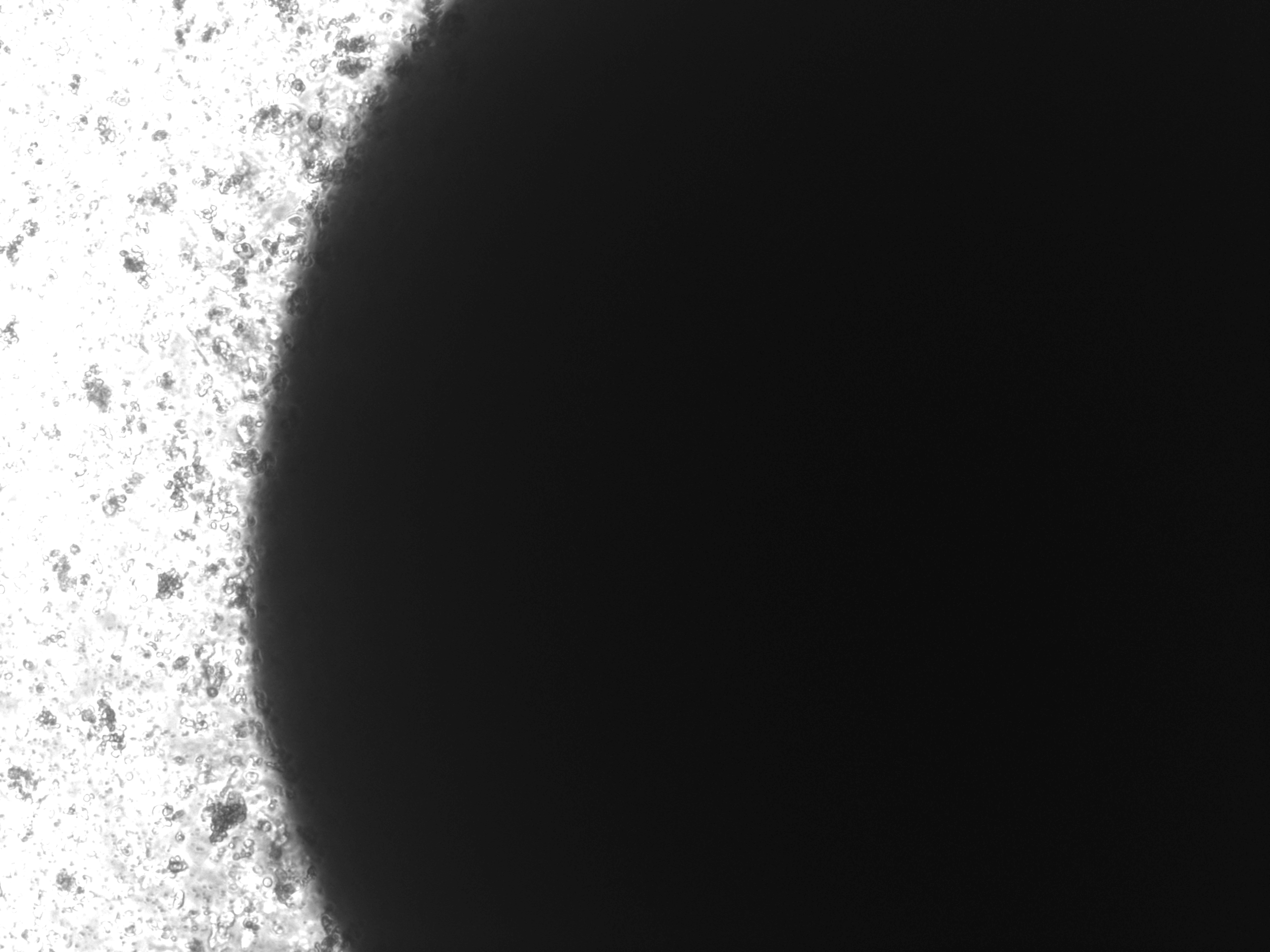

Supplement: Supplementary file 10 — Source Data for Figure 5 [file EMMM-15-e18199-s008.zip › Figure_5/5K/Tumor_C_tumor_pieces_susp_D120a.tif]

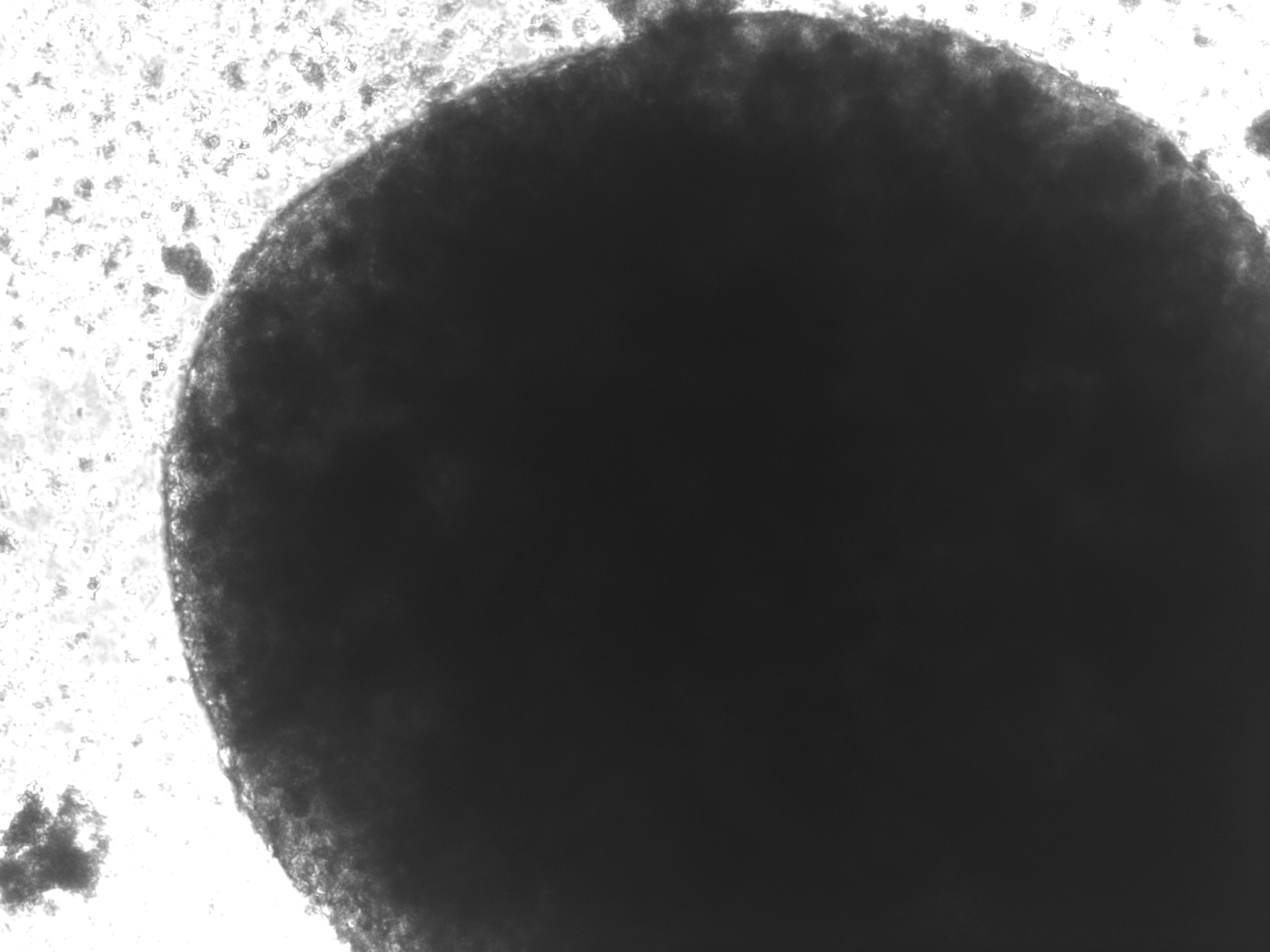

Supplement: Supplementary file 10 — Source Data for Figure 5 [file EMMM-15-e18199-s008.zip › Figure_5/5K/Tumor_C_tumor_pieces_susp_D120b.tif]

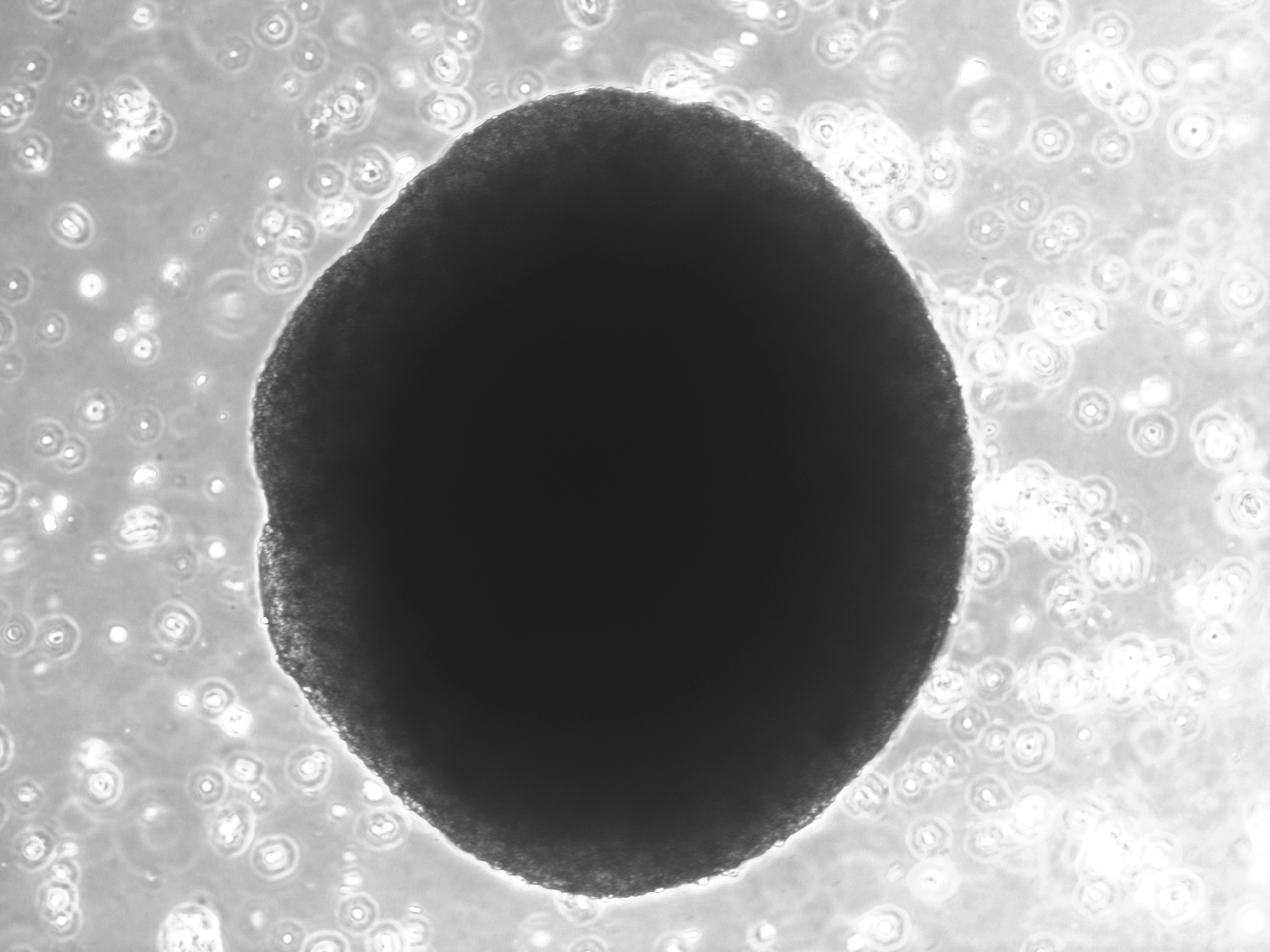

Supplement: Supplementary file 10 — Source Data for Figure 5 [file EMMM-15-e18199-s008.zip › Figure_5/5K/Tumor_C_tumor_pieces_susp_D43a.tif]

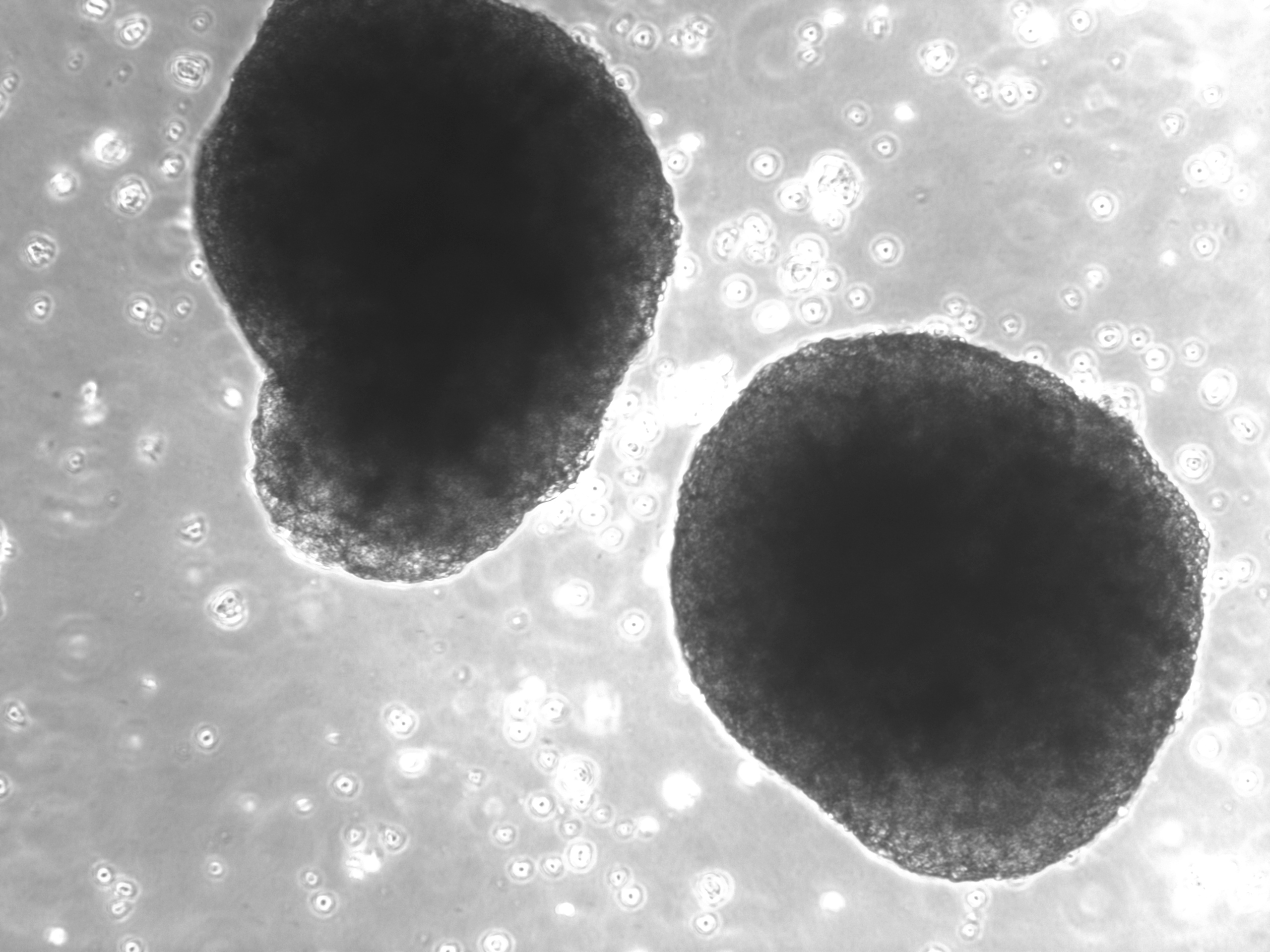

Supplement: Supplementary file 10 — Source Data for Figure 5 [file EMMM-15-e18199-s008.zip › Figure_5/5K/Tumor_C_tumor_pieces_susp_D43b.tif]

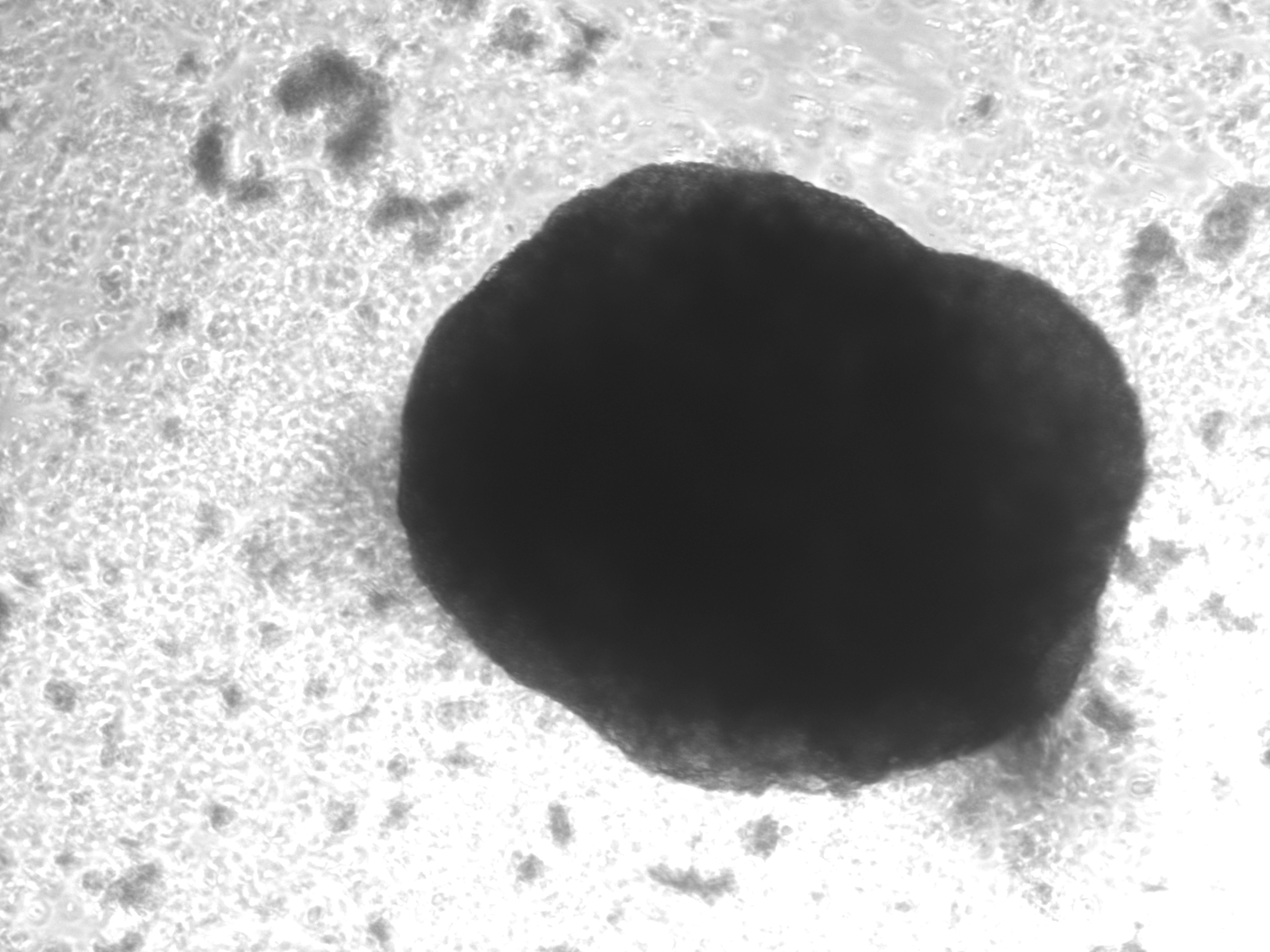

Supplement: Supplementary file 10 — Source Data for Figure 5 [file EMMM-15-e18199-s008.zip › Figure_5/5K/Tumor_C_tumor_pieces_susp_D50a.tif]

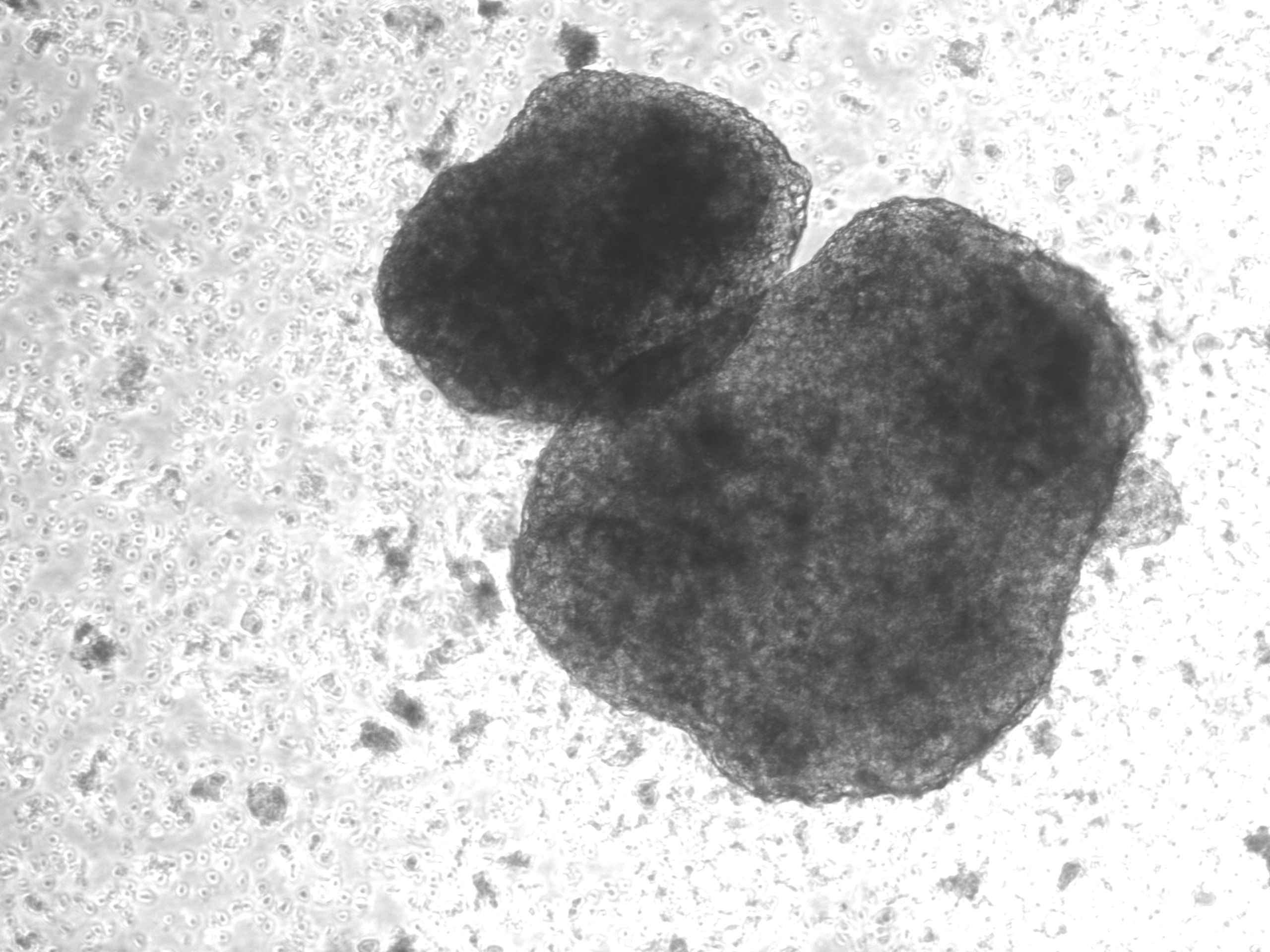

Supplement: Supplementary file 10 — Source Data for Figure 5 [file EMMM-15-e18199-s008.zip › Figure_5/5K/Tumor_C_tumor_pieces_susp_D50b.tif]

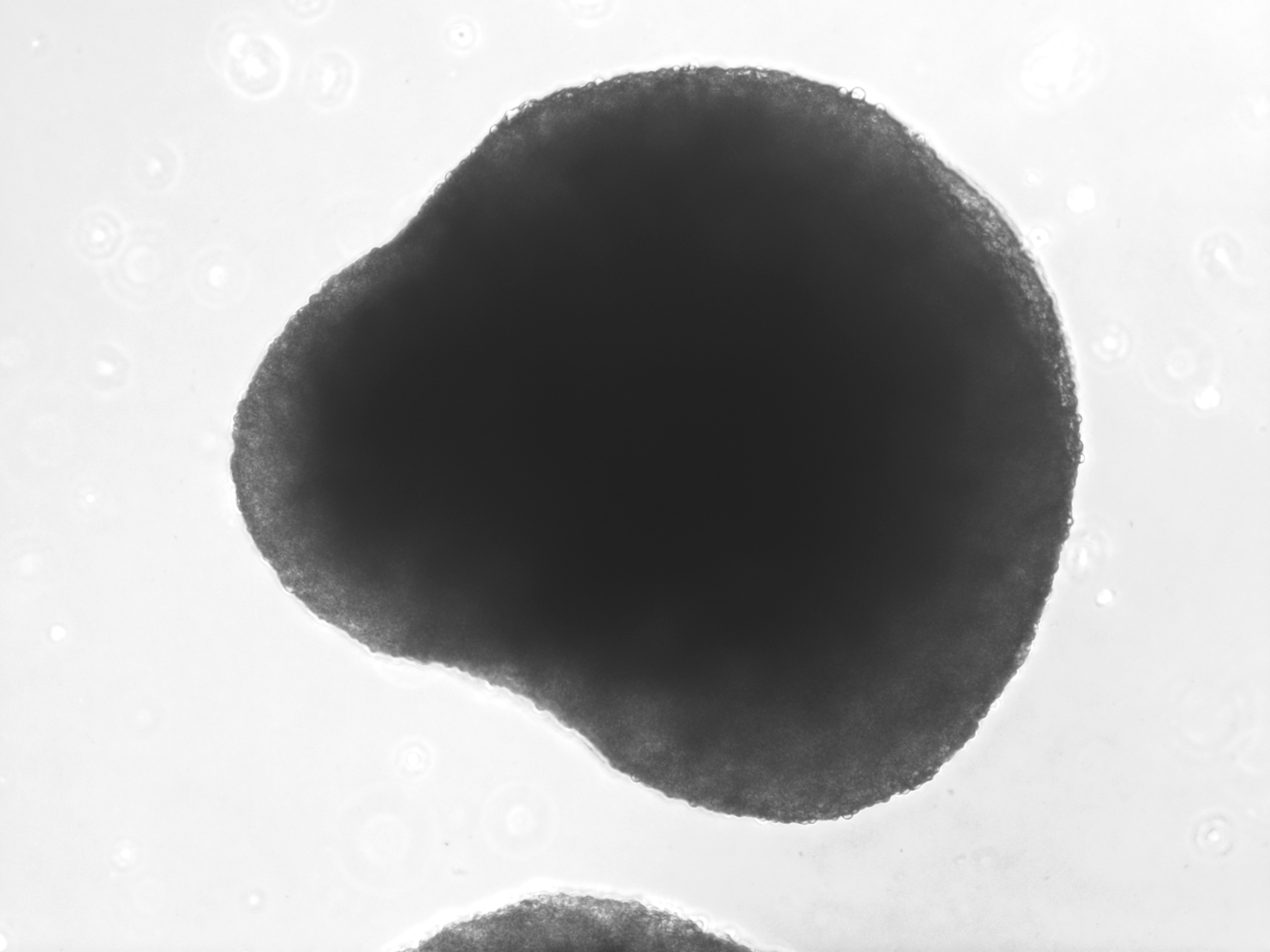

Supplement: Supplementary file 10 — Source Data for Figure 5 [file EMMM-15-e18199-s008.zip › Figure_5/5K/Tumor_C_tumor_pieces_susp_D71a.tif]

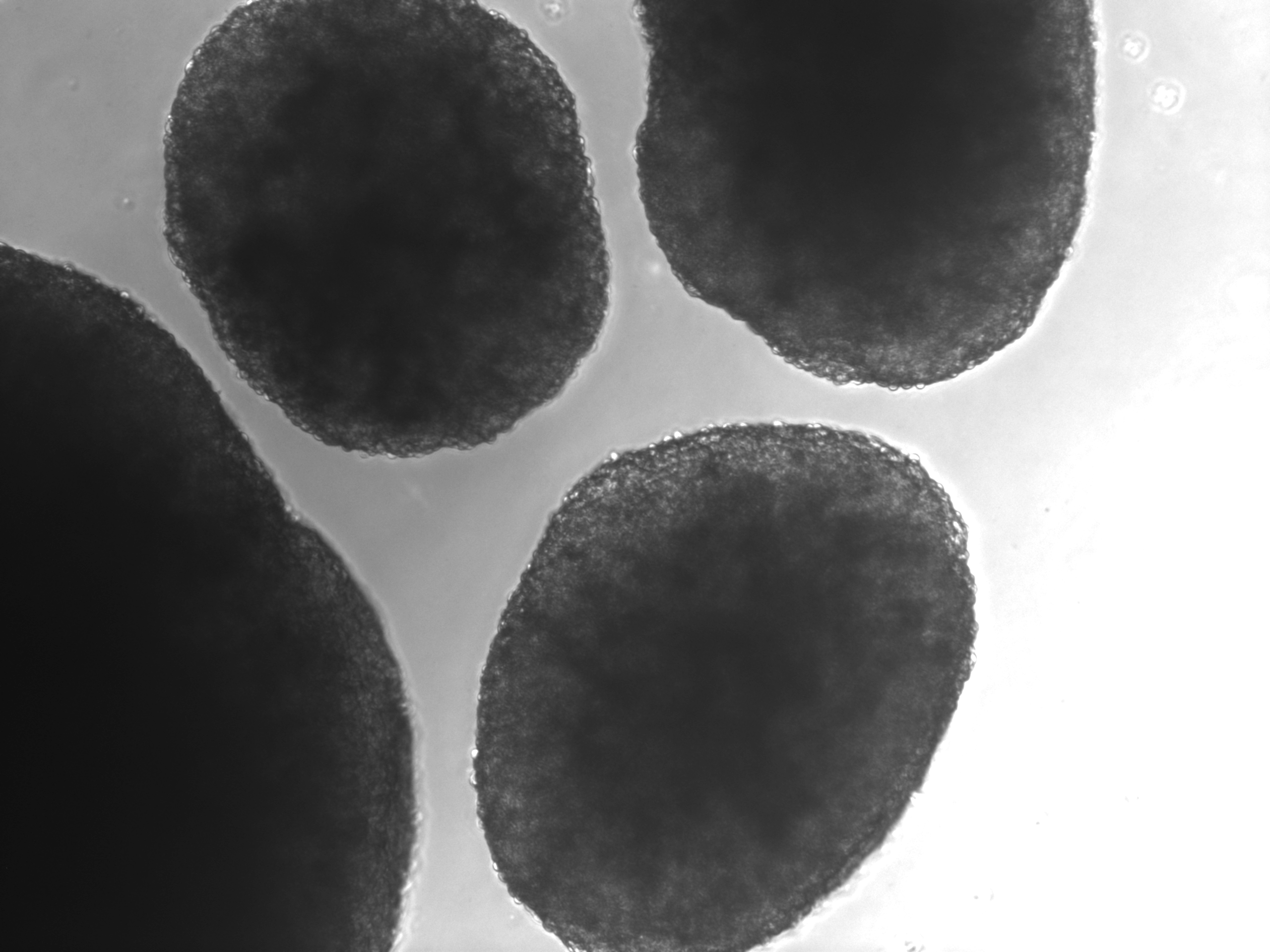

Supplement: Supplementary file 10 — Source Data for Figure 5 [file EMMM-15-e18199-s008.zip › Figure_5/5K/Tumor_C_tumor_pieces_susp_D71b.tif]

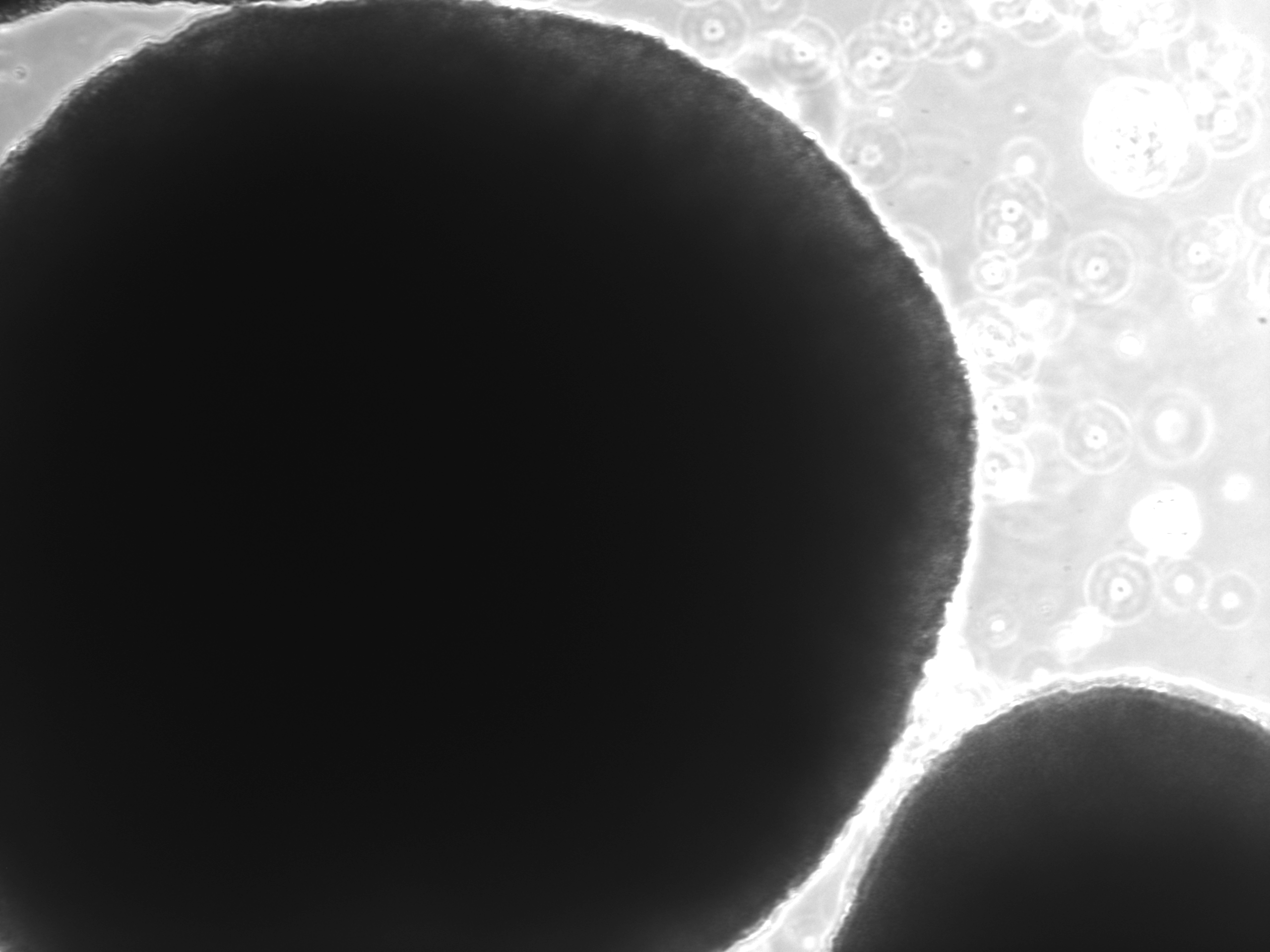

Supplement: Supplementary file 10 — Source Data for Figure 5 [file EMMM-15-e18199-s008.zip › Figure_5/5K/Tumor_C_tumor_pieces_susp_D85a.tif]

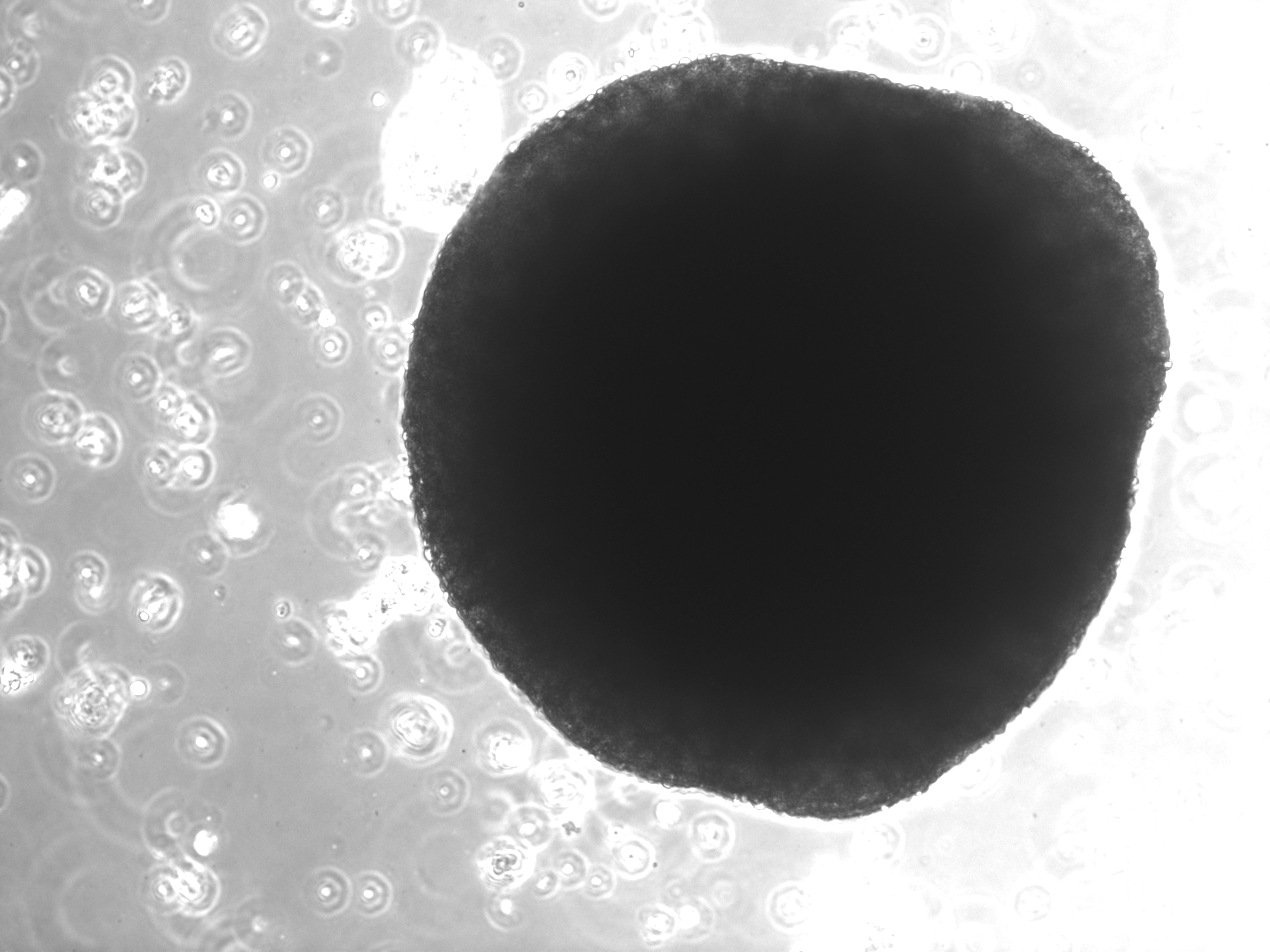

Supplement: Supplementary file 10 — Source Data for Figure 5 [file EMMM-15-e18199-s008.zip › Figure_5/5K/Tumor_C_tumor_pieces_susp_D85b.tif]

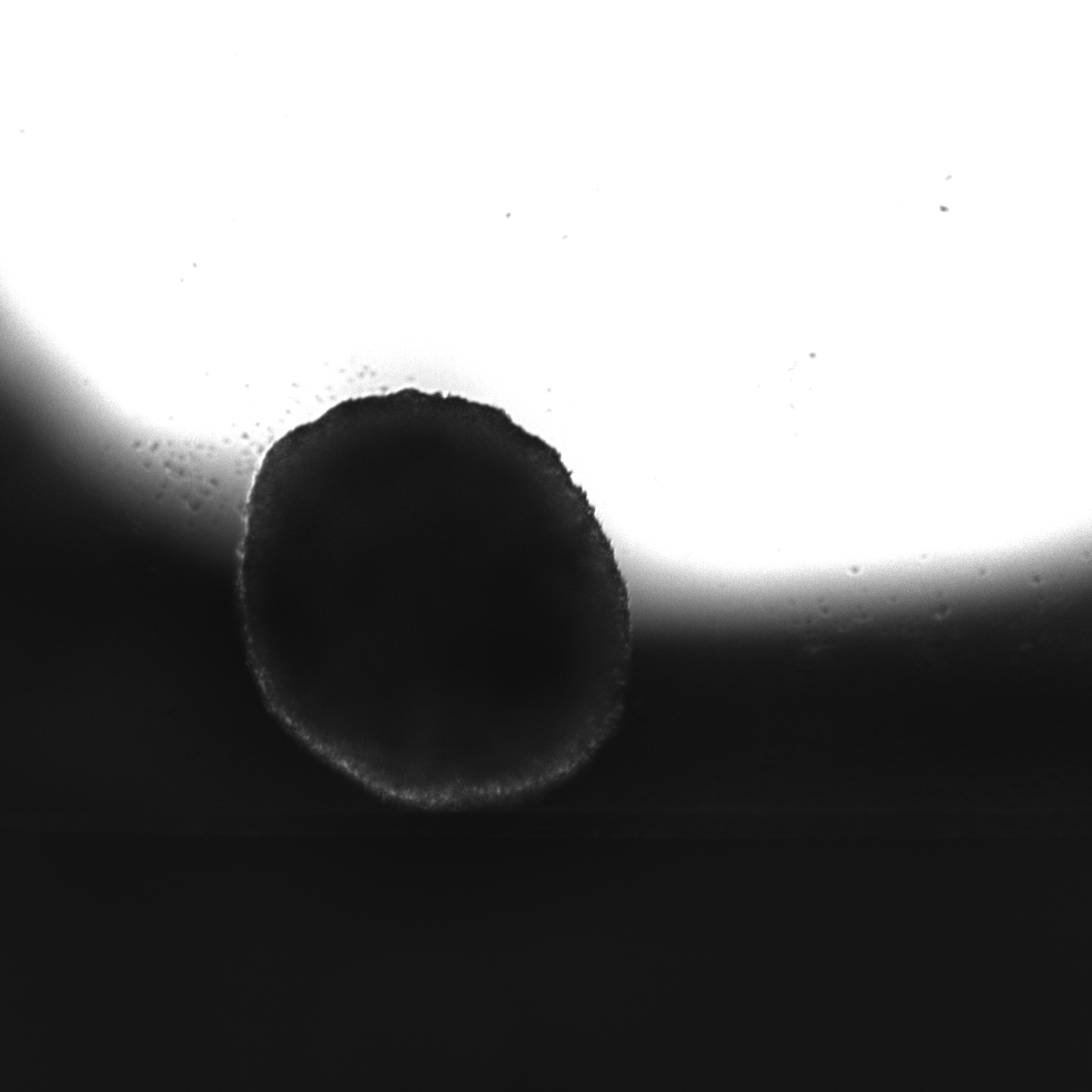

Supplement: Supplementary file 11 — Source Data for Figure 6 [file EMMM-15-e18199-s003.zip › Figure_6/6B/B'_CTRL_PDO_T#5_BF_1.tif]

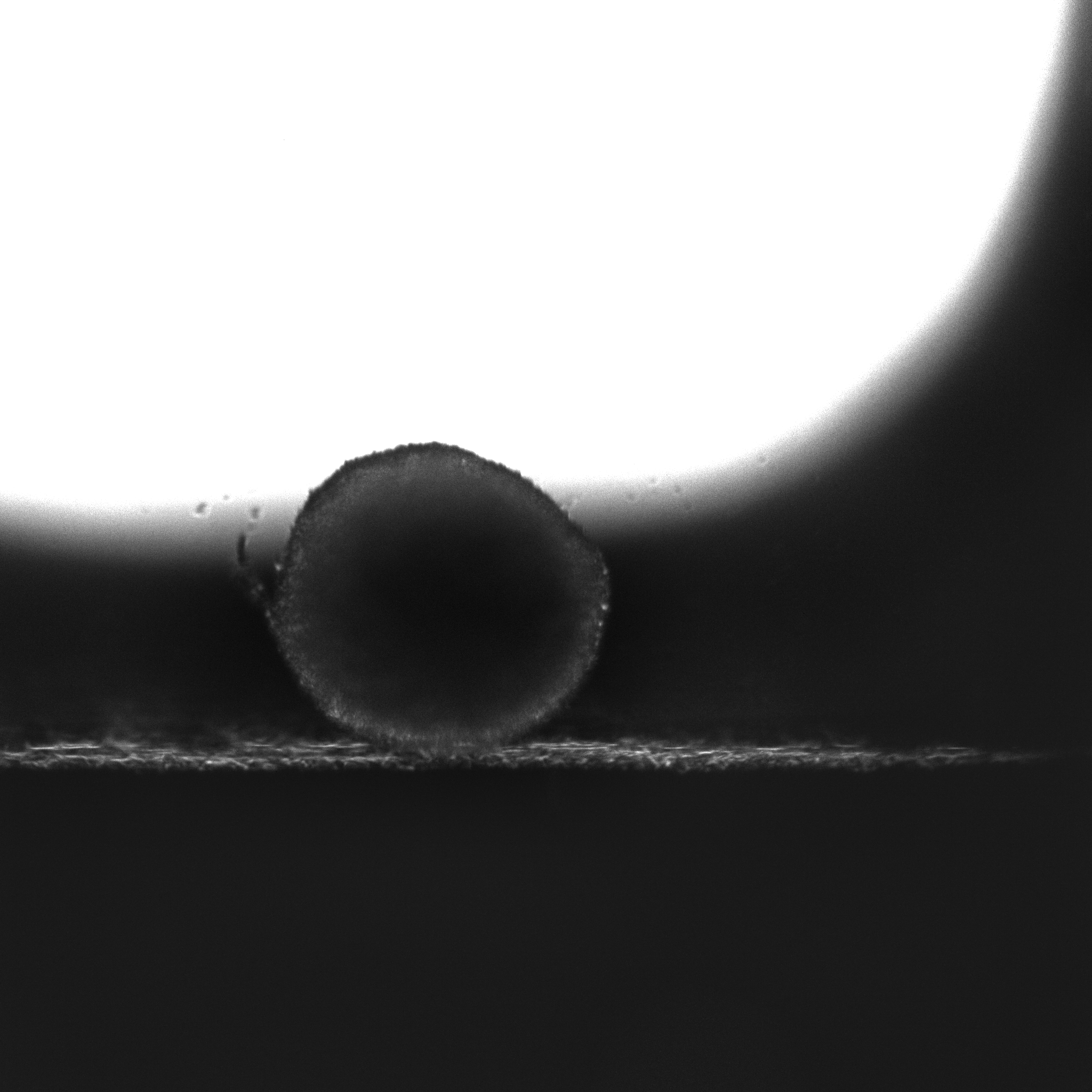

Supplement: Supplementary file 11 — Source Data for Figure 6 [file EMMM-15-e18199-s003.zip › Figure_6/6B/B'_CTRL_PDO_T#5_BF_2.tif]

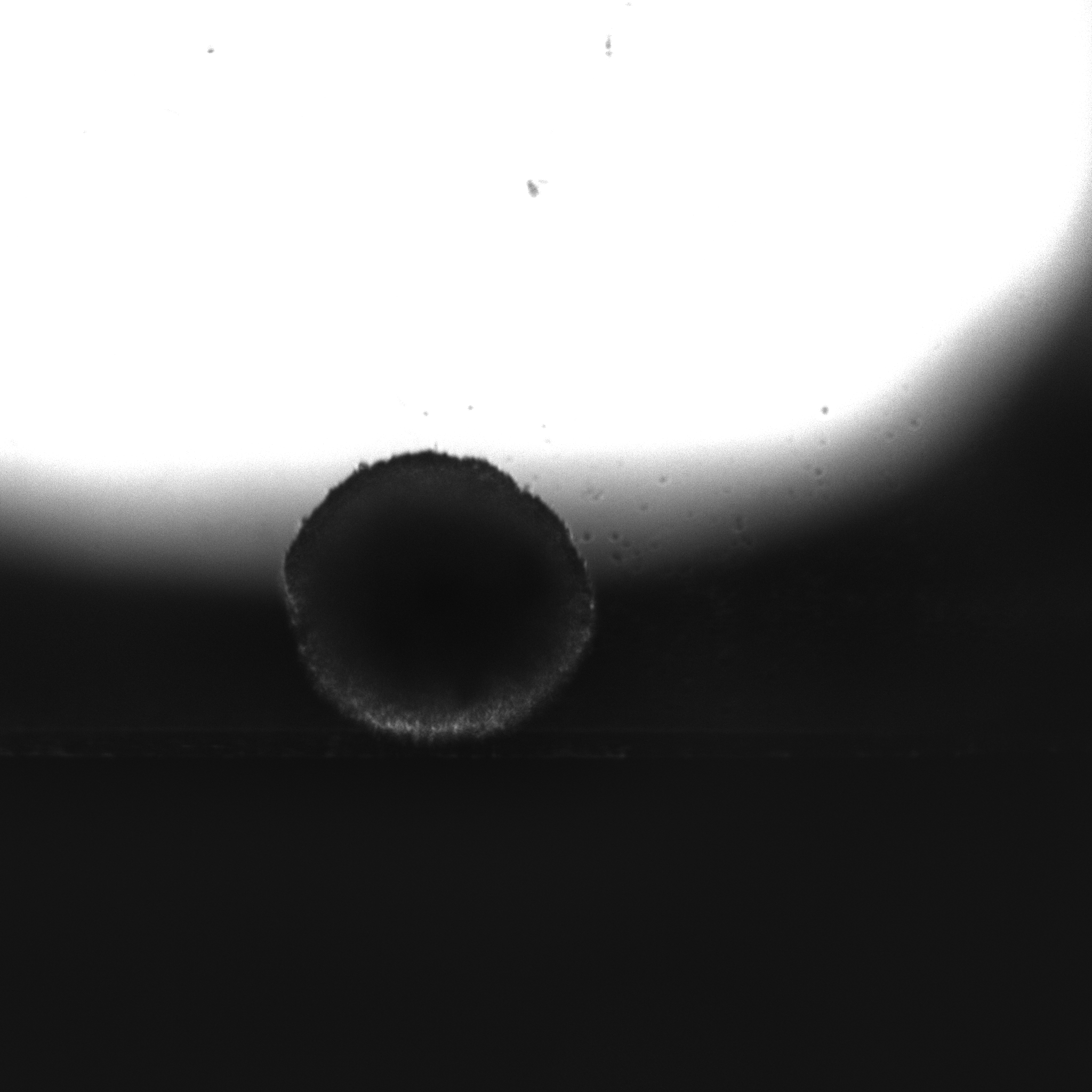

Supplement: Supplementary file 11 — Source Data for Figure 6 [file EMMM-15-e18199-s003.zip › Figure_6/6B/B'_CTRL_PDO_T#5_BF_3.tif]

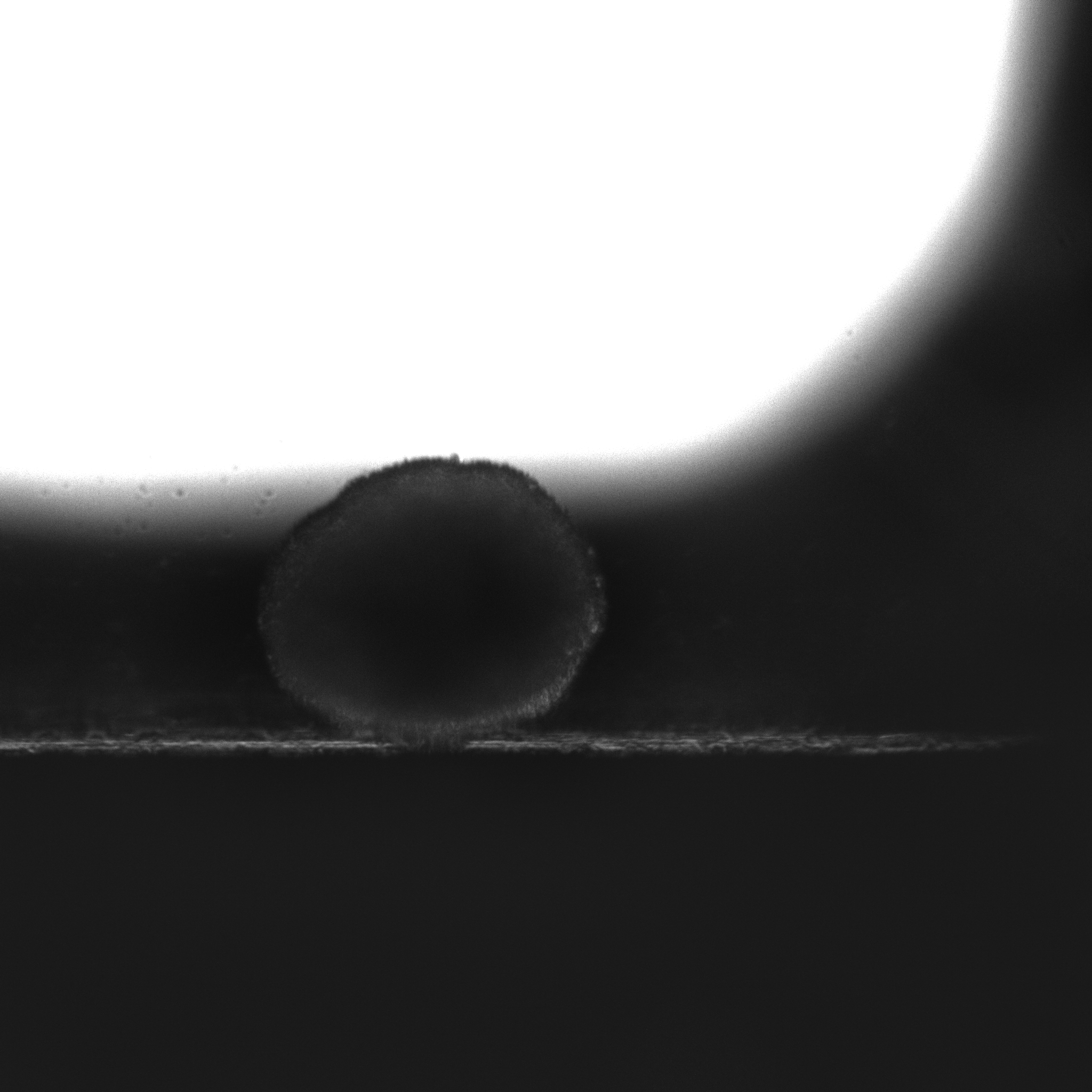

Supplement: Supplementary file 11 — Source Data for Figure 6 [file EMMM-15-e18199-s003.zip › Figure_6/6B/B'_CTRL_PDO_T#5_BF_4.tif]

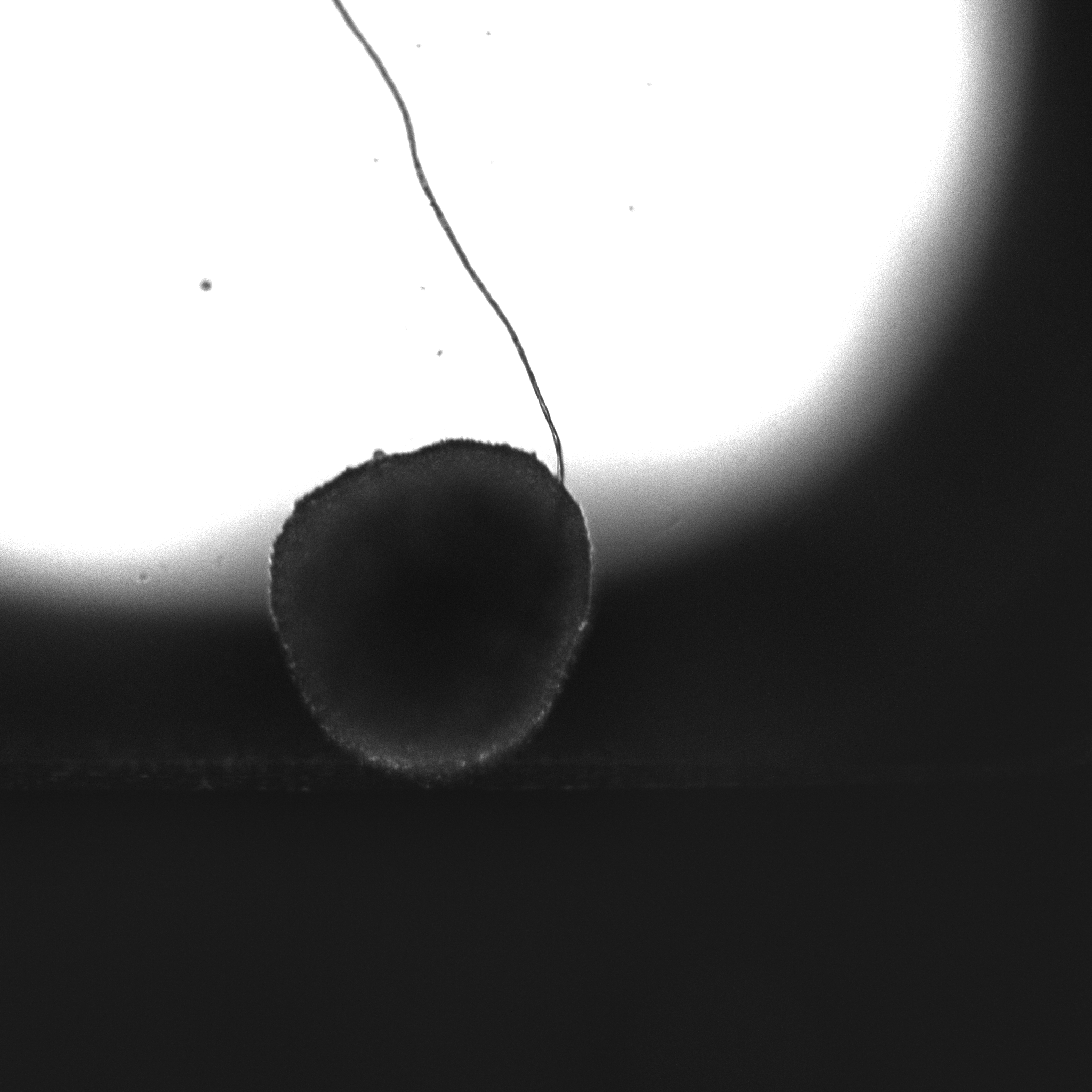

Supplement: Supplementary file 11 — Source Data for Figure 6 [file EMMM-15-e18199-s003.zip › Figure_6/6B/B'_CTRL_PDO_T#5_BF_5.tif]

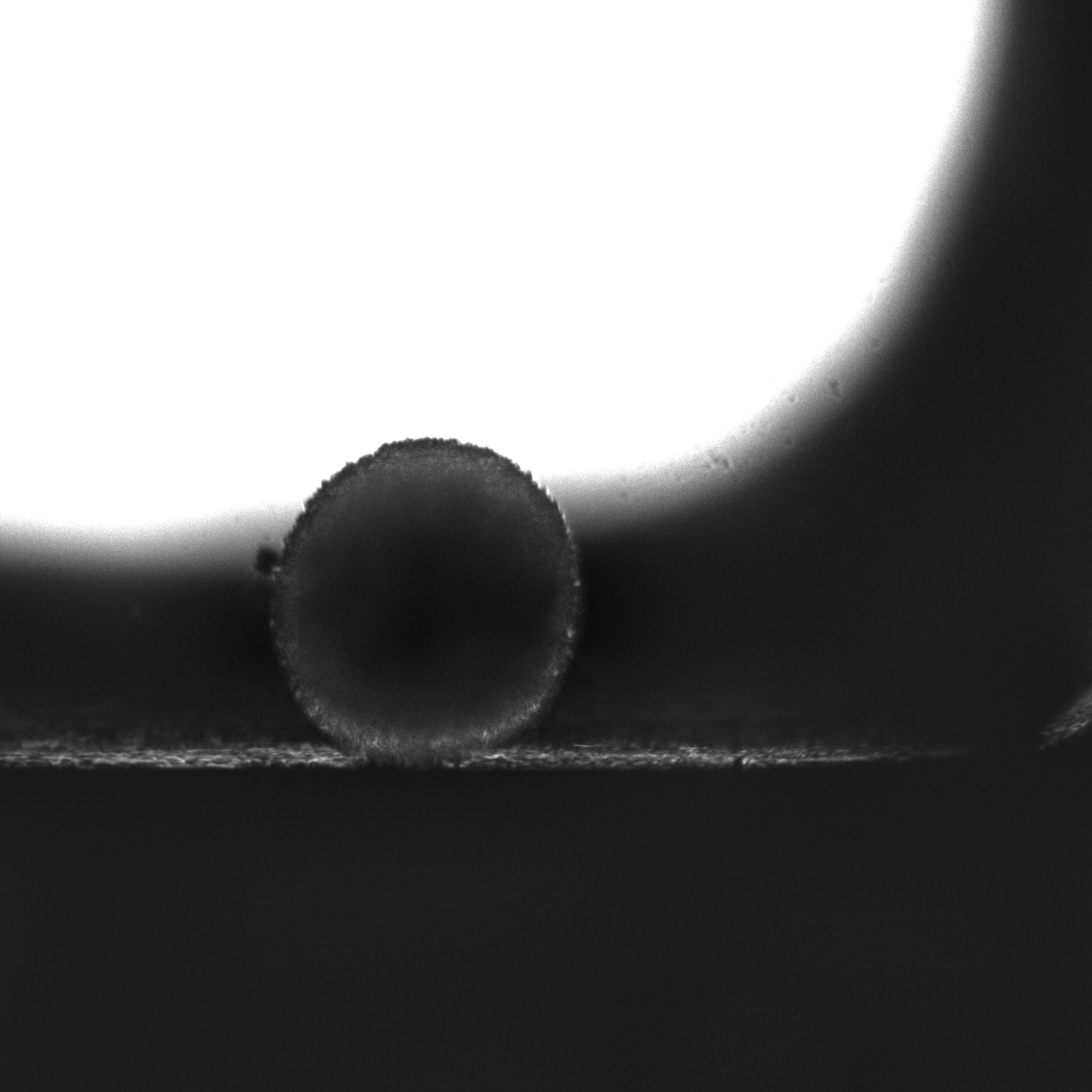

Supplement: Supplementary file 11 — Source Data for Figure 6 [file EMMM-15-e18199-s003.zip › Figure_6/6B/B'_CTRL_PDO_T#5_BF_6.tif]

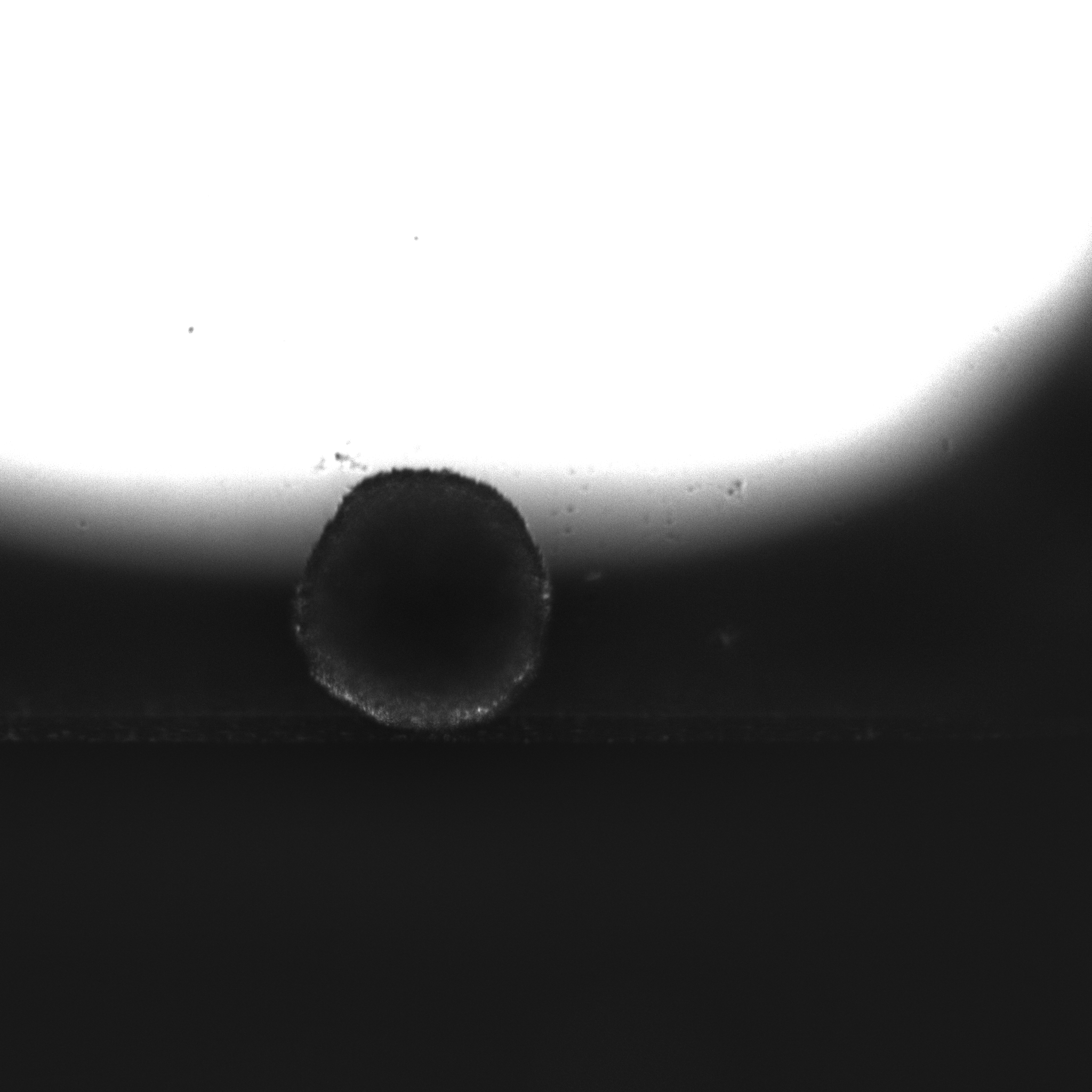

Supplement: Supplementary file 11 — Source Data for Figure 6 [file EMMM-15-e18199-s003.zip › Figure_6/6B/B'_CTRL_PDO_T#5_BF_7.tif]
