# Supplementary figures and images for: Patient‐ and xenograft‐derived organoids recapitulate pediatric brain tumor features and patient treatments (part 4 of 4)
Source: EMBO Mol Med. 2023 Nov 30;15(12):e18199. doi: 10.15252/emmm.202318199 (PMC10701620; doi:10.15252/emmm.202318199)

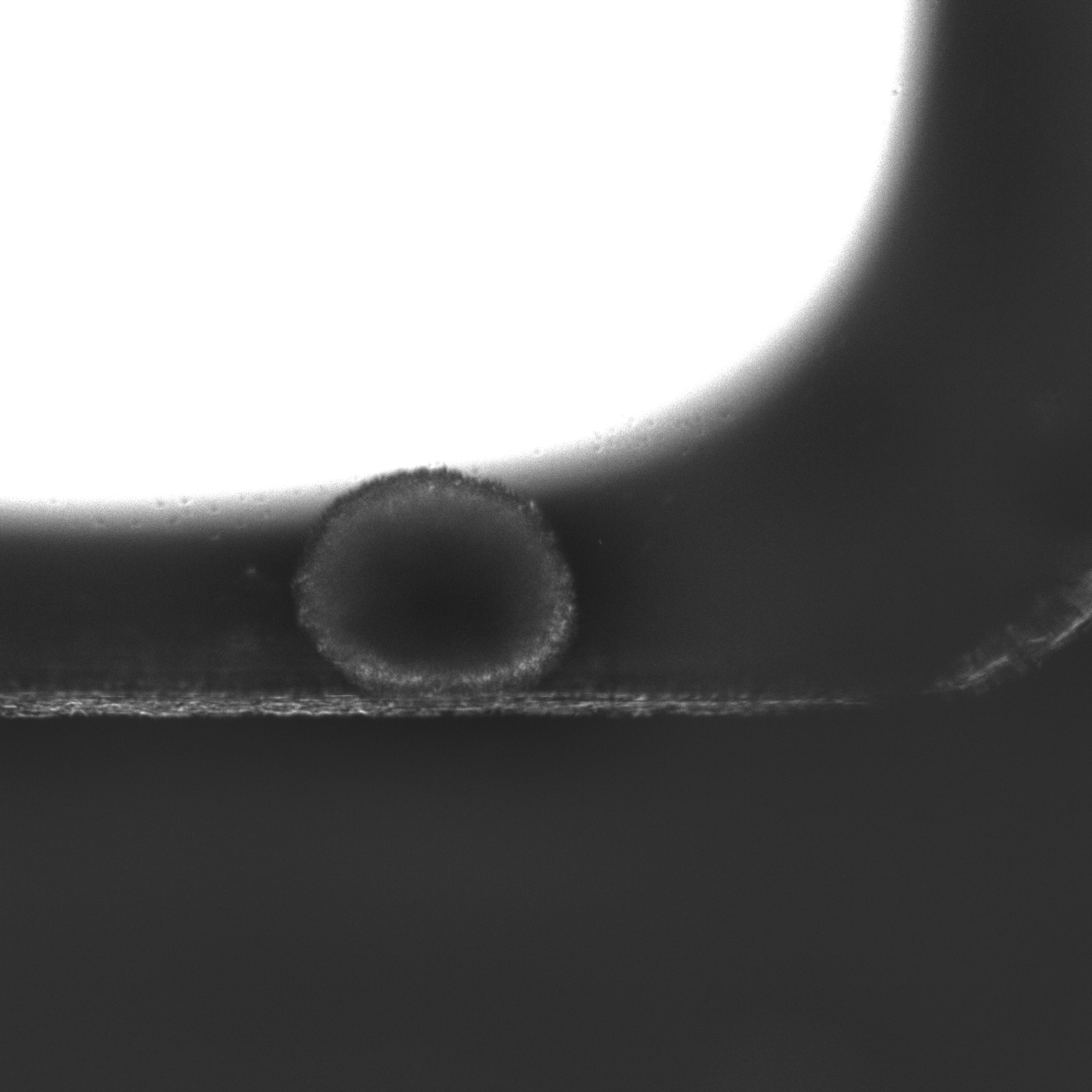

Supplement: Supplementary file 11 — Source Data for Figure 6 [file EMMM-15-e18199-s003.zip › Figure_6/6B/B'_CTRL_PDO_T#5_BF_8.tif]

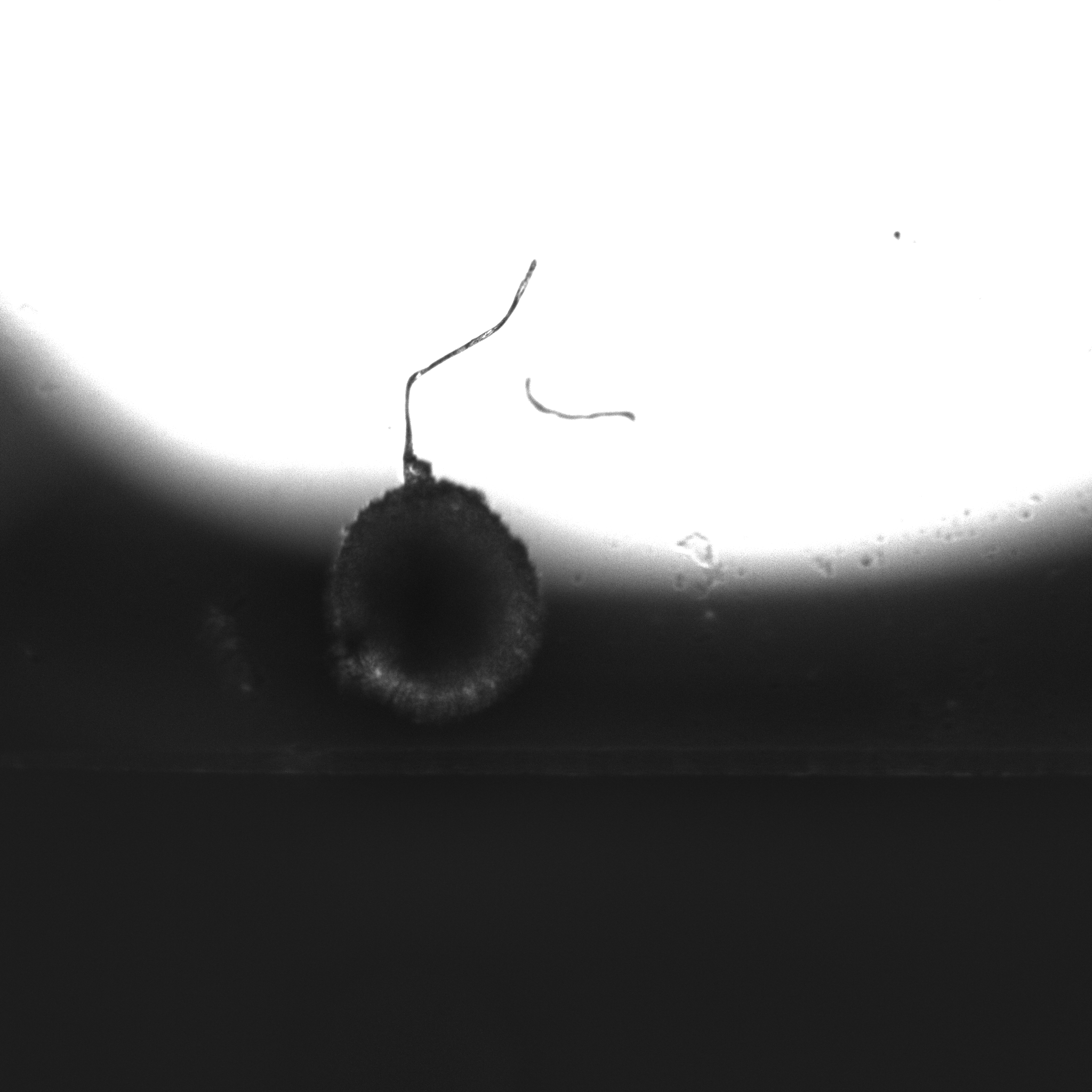

Supplement: Supplementary file 11 — Source Data for Figure 6 [file EMMM-15-e18199-s003.zip › Figure_6/6B/B'_CTRL_PDO_T#5_BF_9.tif]

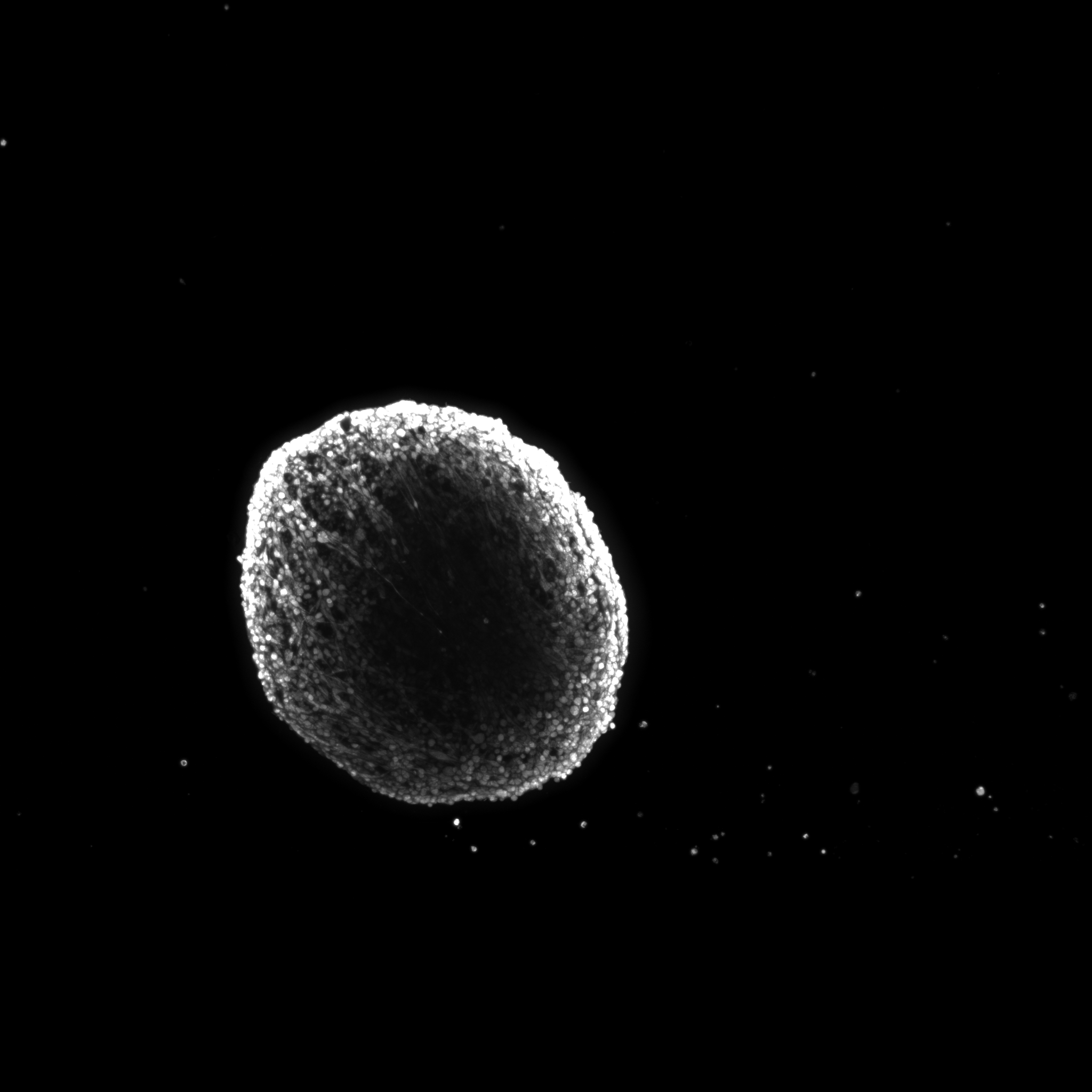

Supplement: Supplementary file 11 — Source Data for Figure 6 [file EMMM-15-e18199-s003.zip › Figure_6/6B/B'_CTRL_PDO_T#5_FLUO_1.tif]

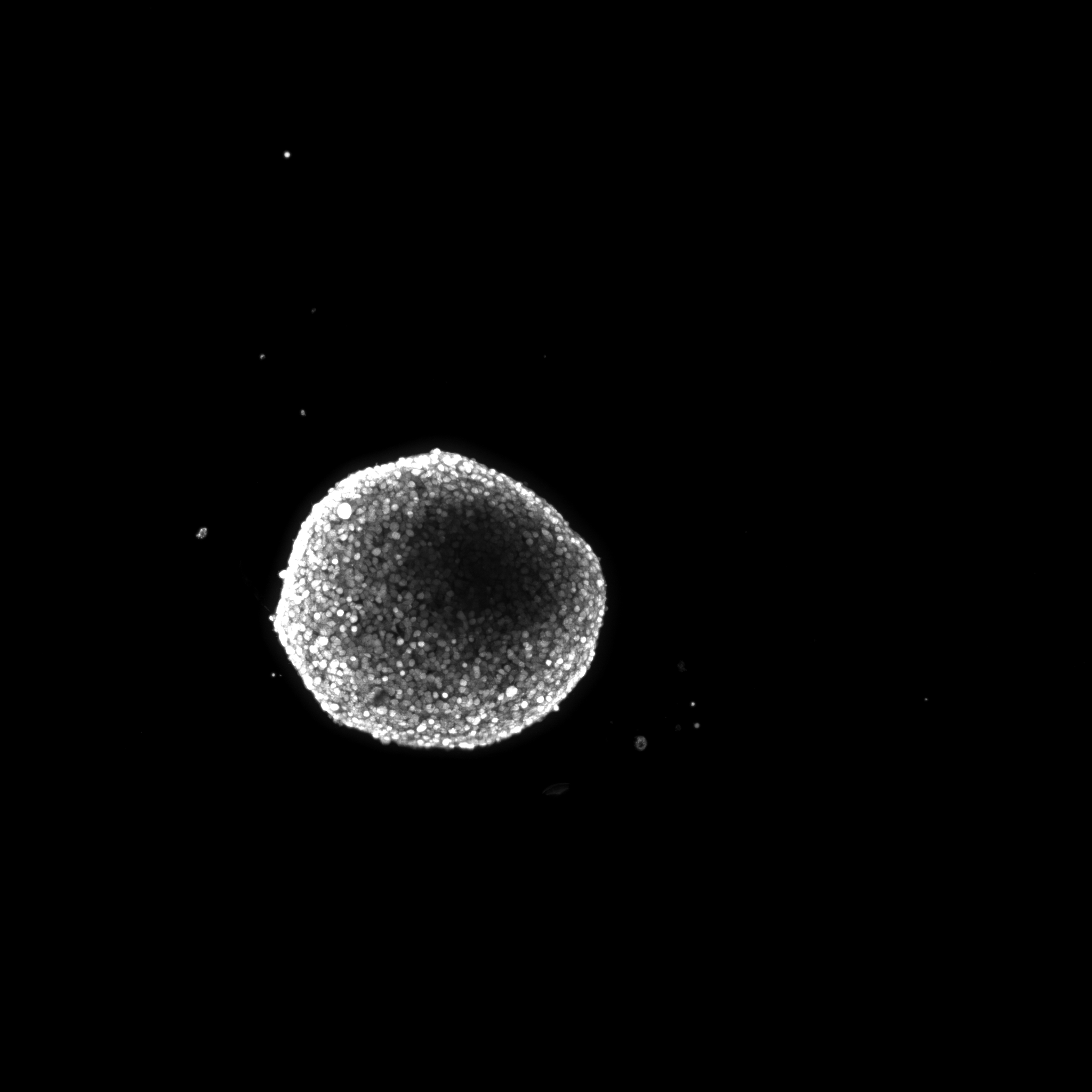

Supplement: Supplementary file 11 — Source Data for Figure 6 [file EMMM-15-e18199-s003.zip › Figure_6/6B/B'_CTRL_PDO_T#5_FLUO_2.tif]

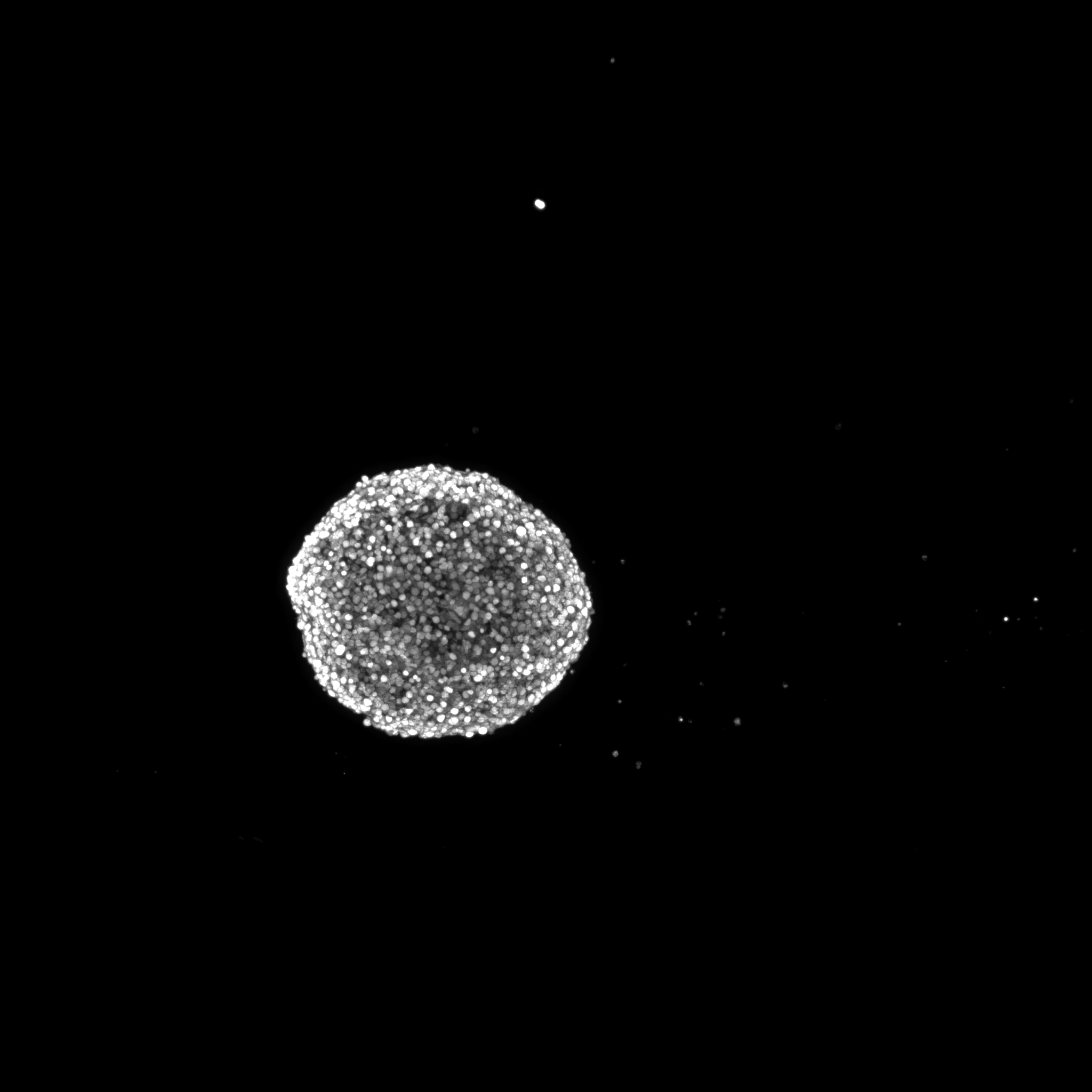

Supplement: Supplementary file 11 — Source Data for Figure 6 [file EMMM-15-e18199-s003.zip › Figure_6/6B/B'_CTRL_PDO_T#5_FLUO_3.tif]

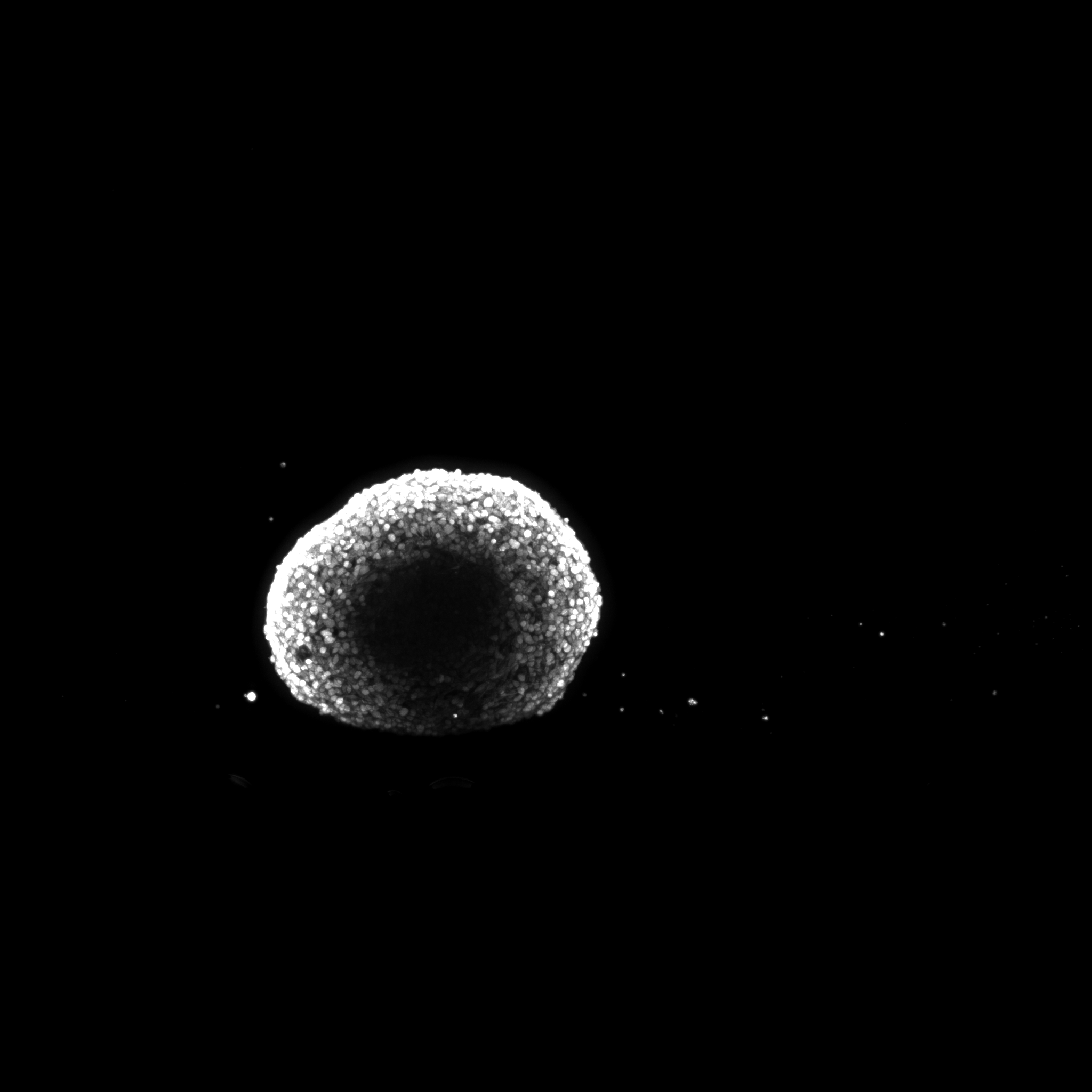

Supplement: Supplementary file 11 — Source Data for Figure 6 [file EMMM-15-e18199-s003.zip › Figure_6/6B/B'_CTRL_PDO_T#5_FLUO_4.tif]

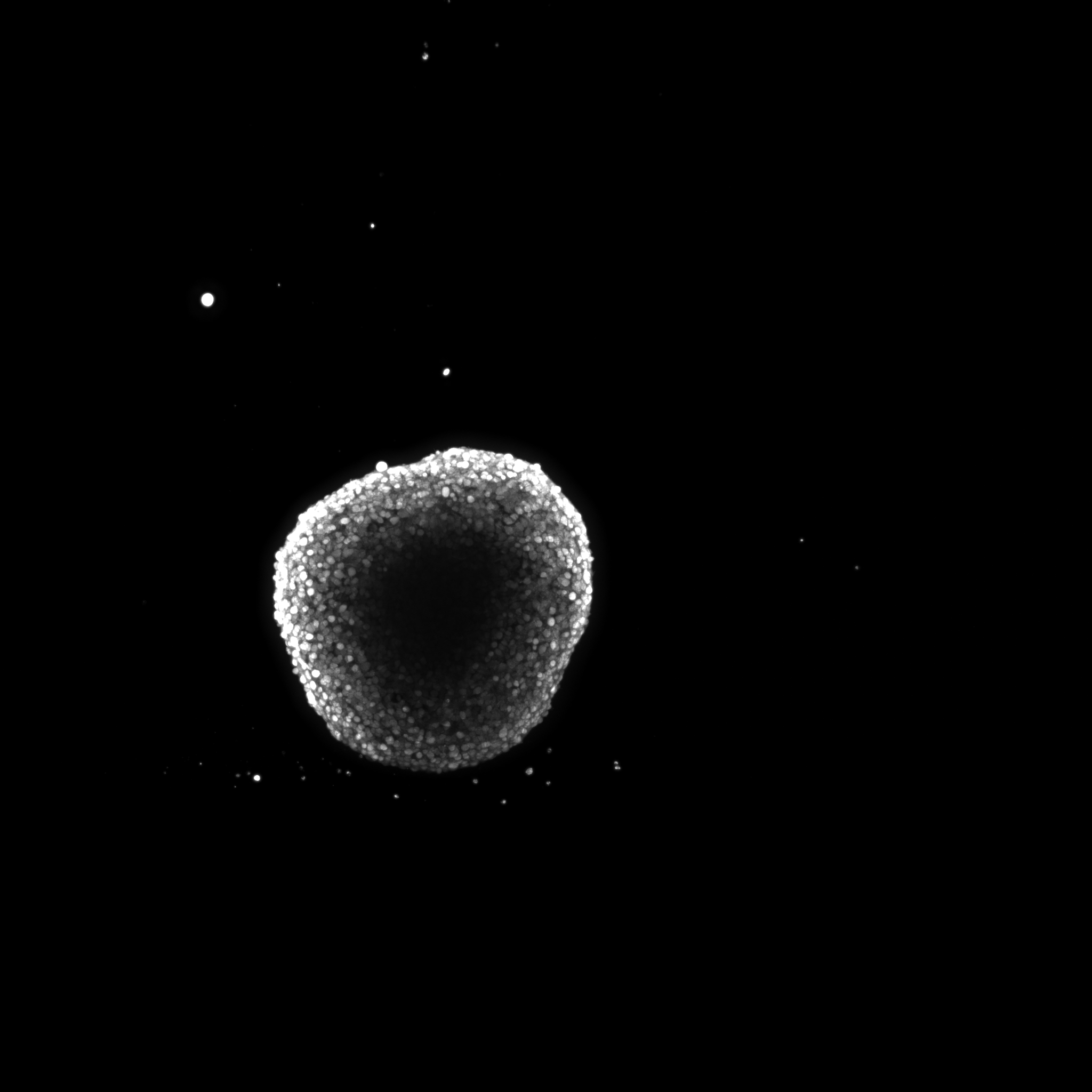

Supplement: Supplementary file 11 — Source Data for Figure 6 [file EMMM-15-e18199-s003.zip › Figure_6/6B/B'_CTRL_PDO_T#5_FLUO_5.tif]

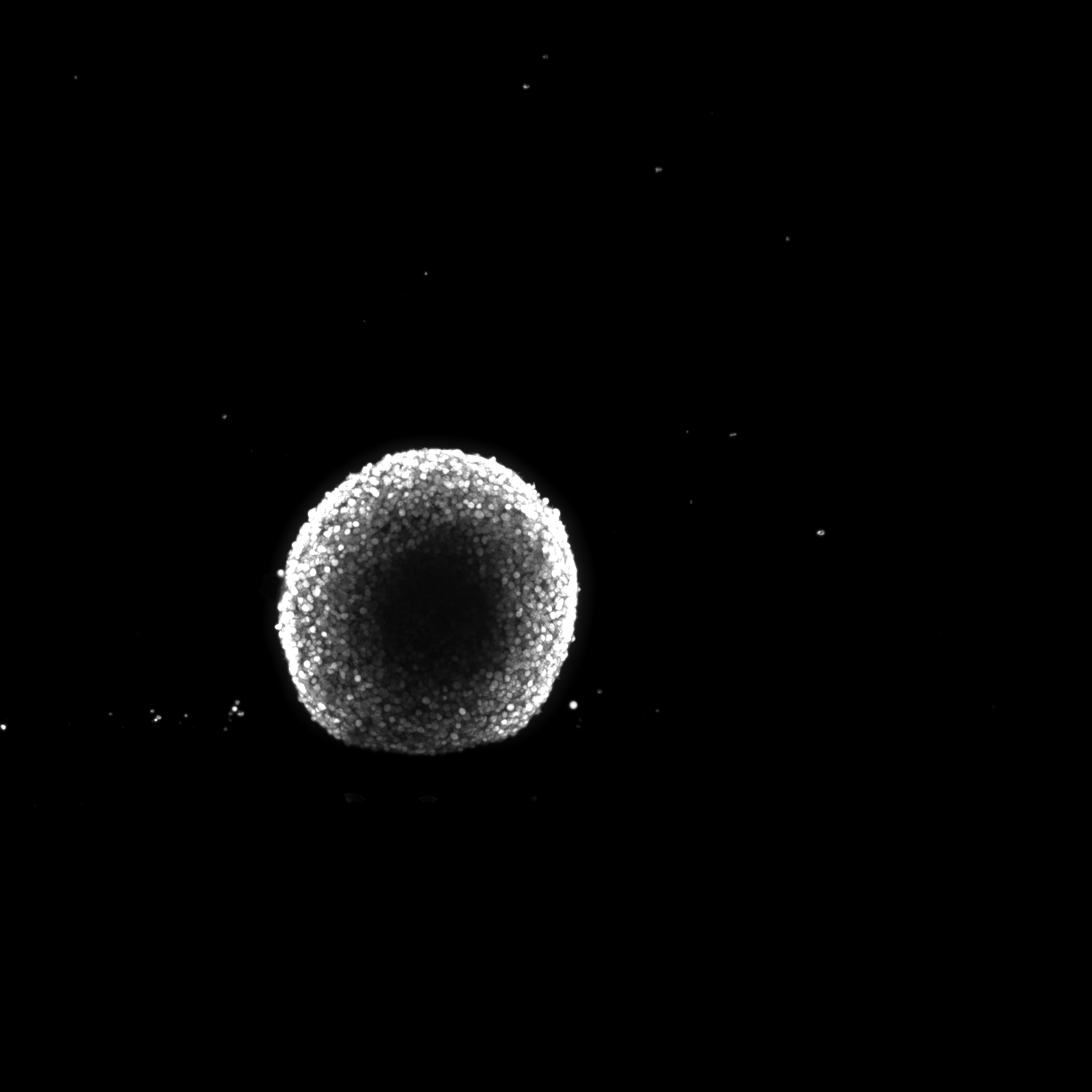

Supplement: Supplementary file 11 — Source Data for Figure 6 [file EMMM-15-e18199-s003.zip › Figure_6/6B/B'_CTRL_PDO_T#5_FLUO_6.tif]

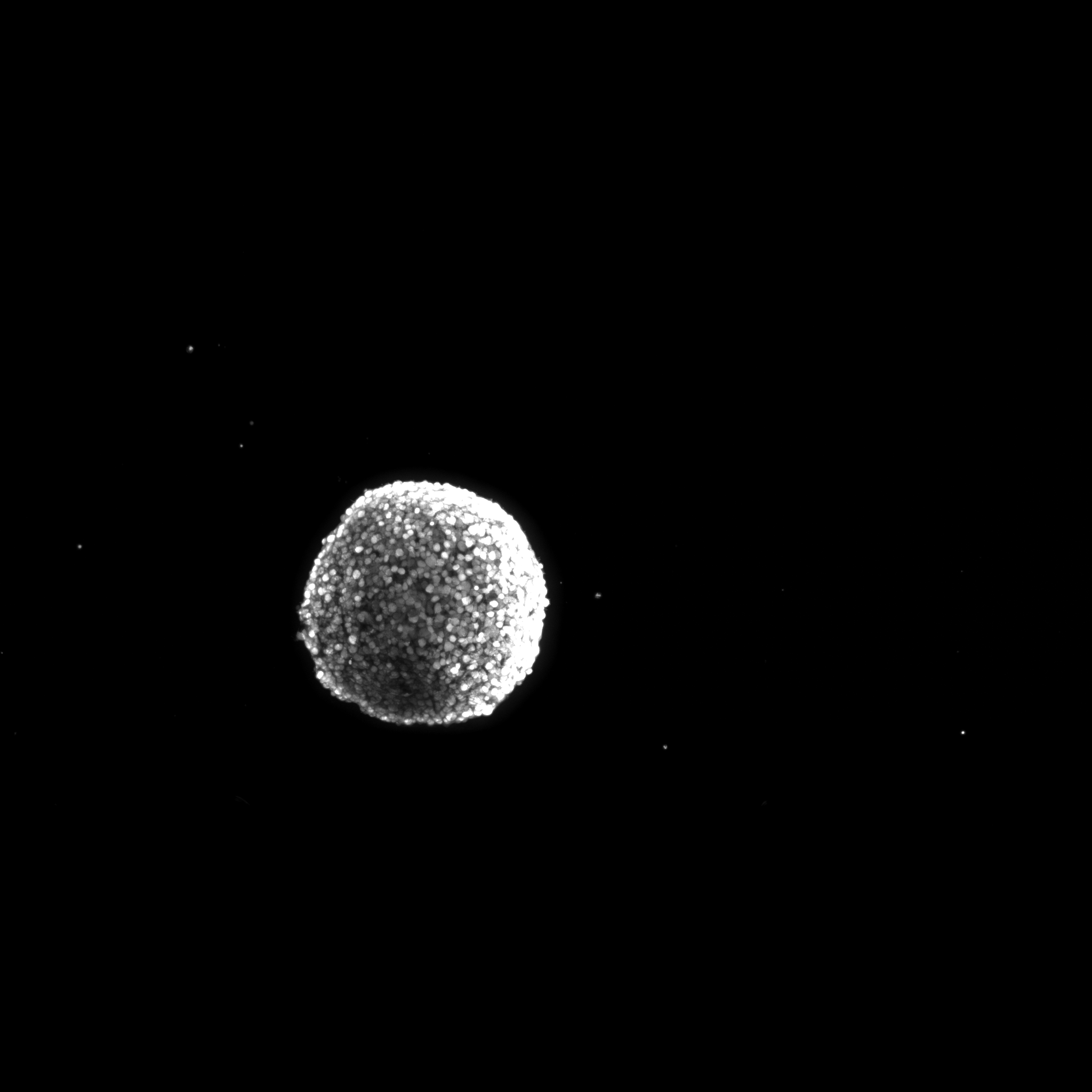

Supplement: Supplementary file 11 — Source Data for Figure 6 [file EMMM-15-e18199-s003.zip › Figure_6/6B/B'_CTRL_PDO_T#5_FLUO_7.tif]

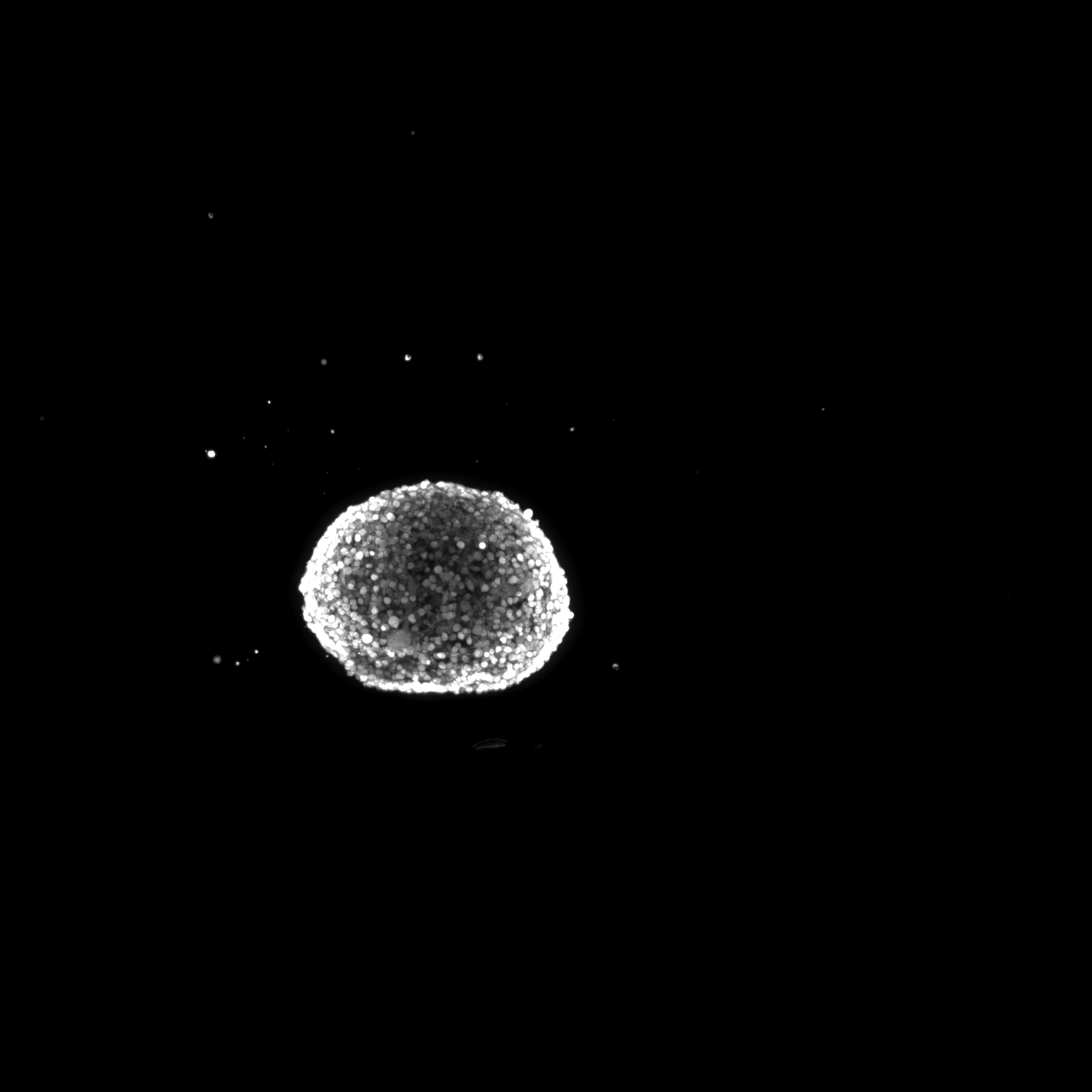

Supplement: Supplementary file 11 — Source Data for Figure 6 [file EMMM-15-e18199-s003.zip › Figure_6/6B/B'_CTRL_PDO_T#5_FLUO_8.tif]

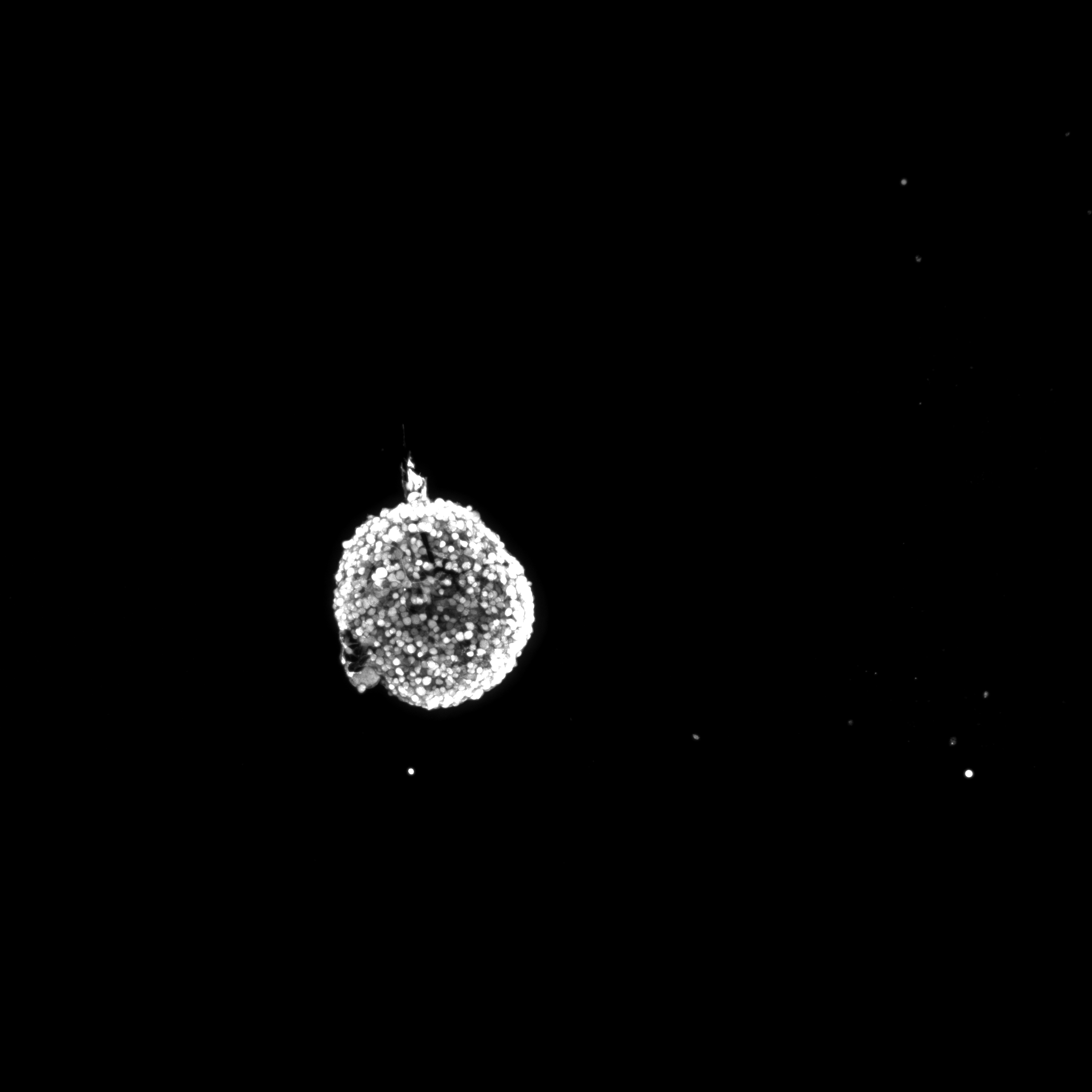

Supplement: Supplementary file 11 — Source Data for Figure 6 [file EMMM-15-e18199-s003.zip › Figure_6/6B/B'_CTRL_PDO_T#5_FLUO_9.tif]

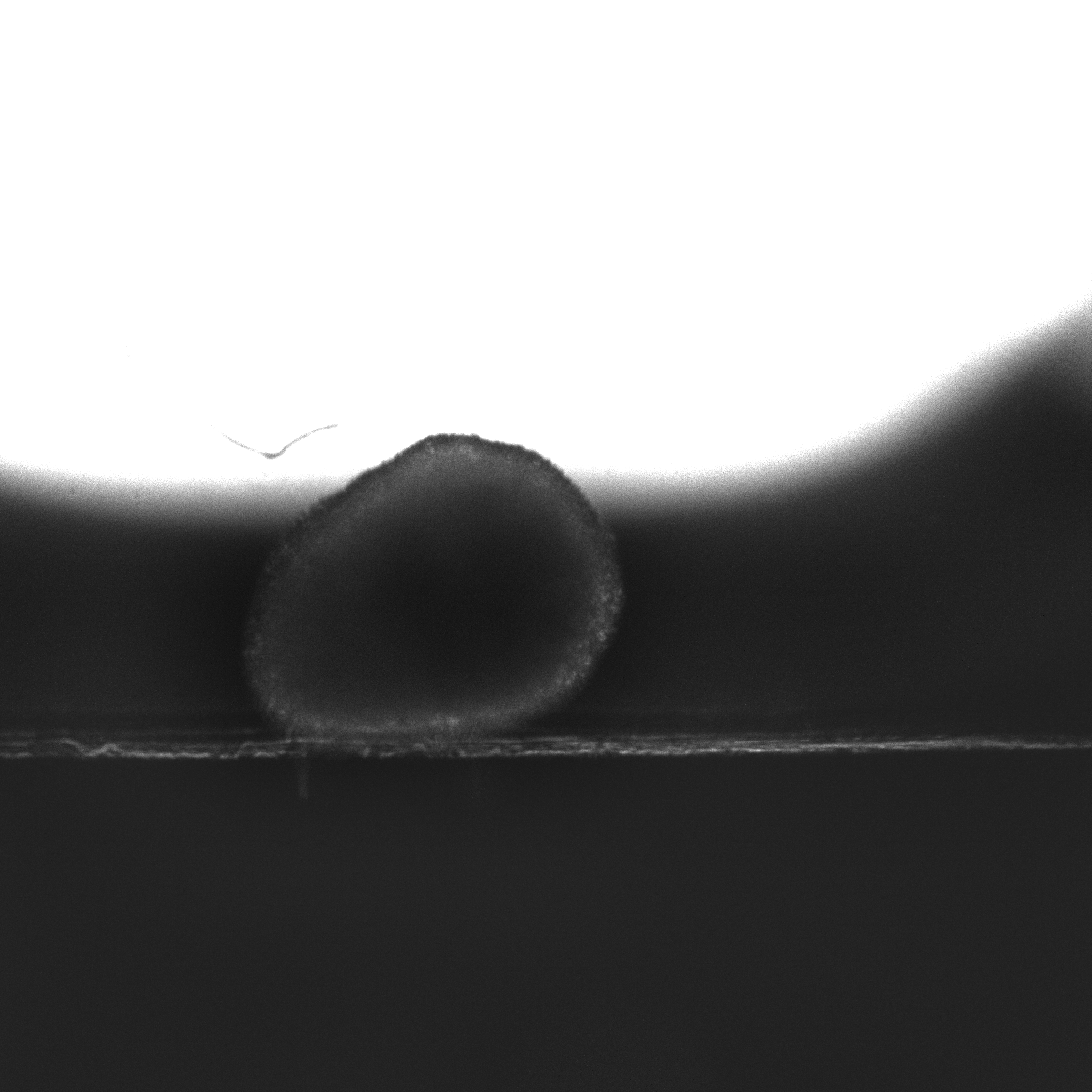

Supplement: Supplementary file 11 — Source Data for Figure 6 [file EMMM-15-e18199-s003.zip › Figure_6/6B/B'_Treatment_PDO_T#5_BF_1.tif]

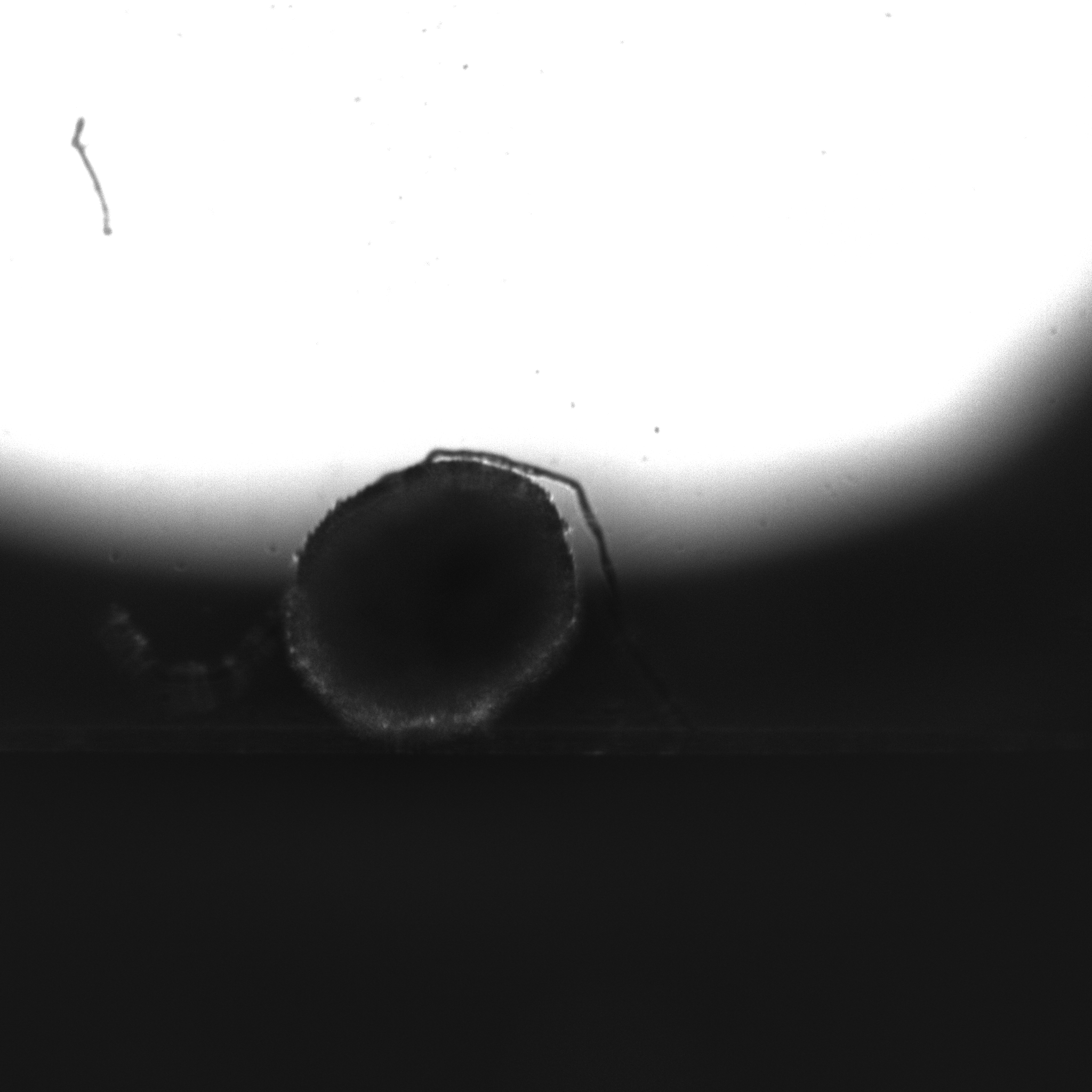

Supplement: Supplementary file 11 — Source Data for Figure 6 [file EMMM-15-e18199-s003.zip › Figure_6/6B/B'_Treatment_PDO_T#5_BF_2.tif]

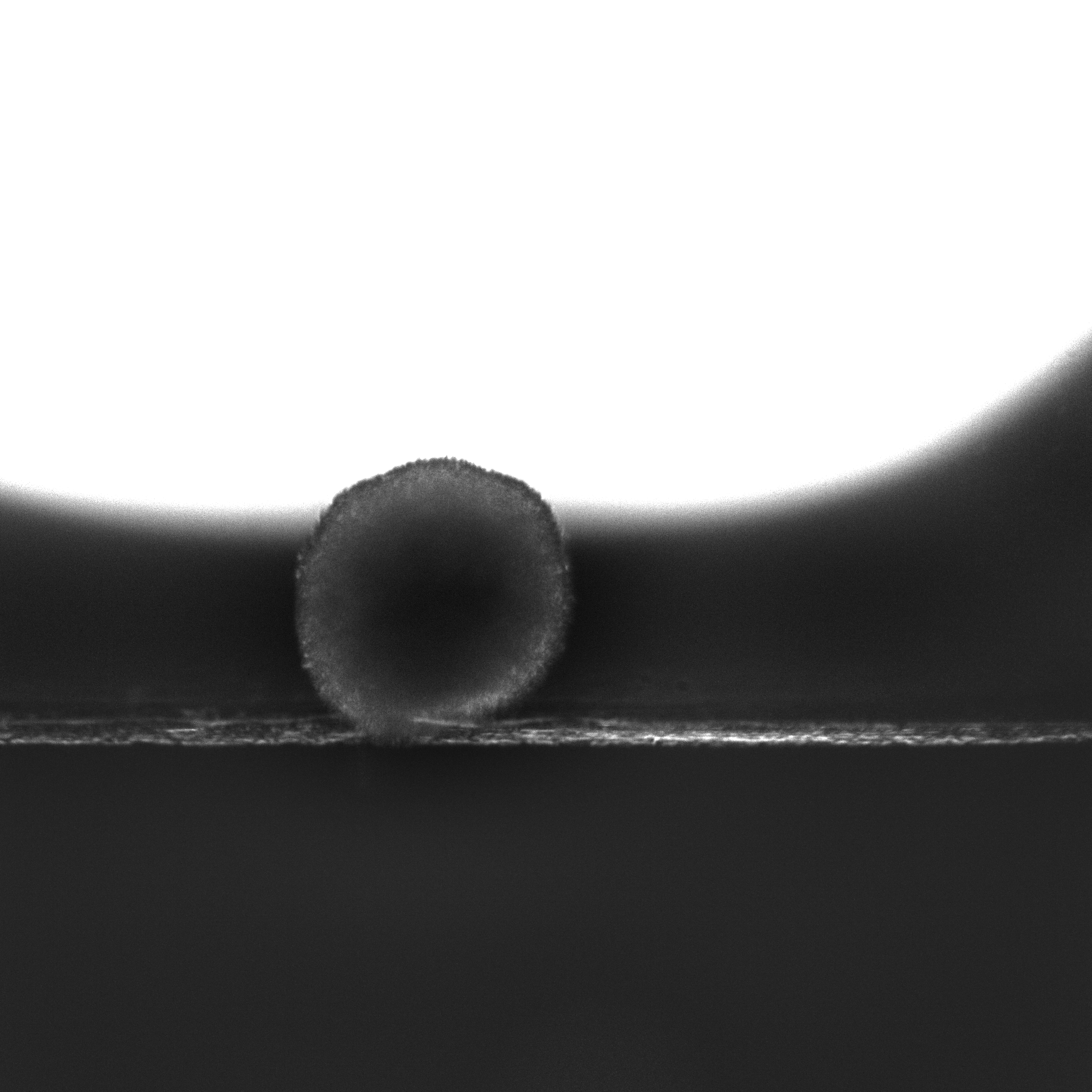

Supplement: Supplementary file 11 — Source Data for Figure 6 [file EMMM-15-e18199-s003.zip › Figure_6/6B/B'_Treatment_PDO_T#5_BF_3.tif]

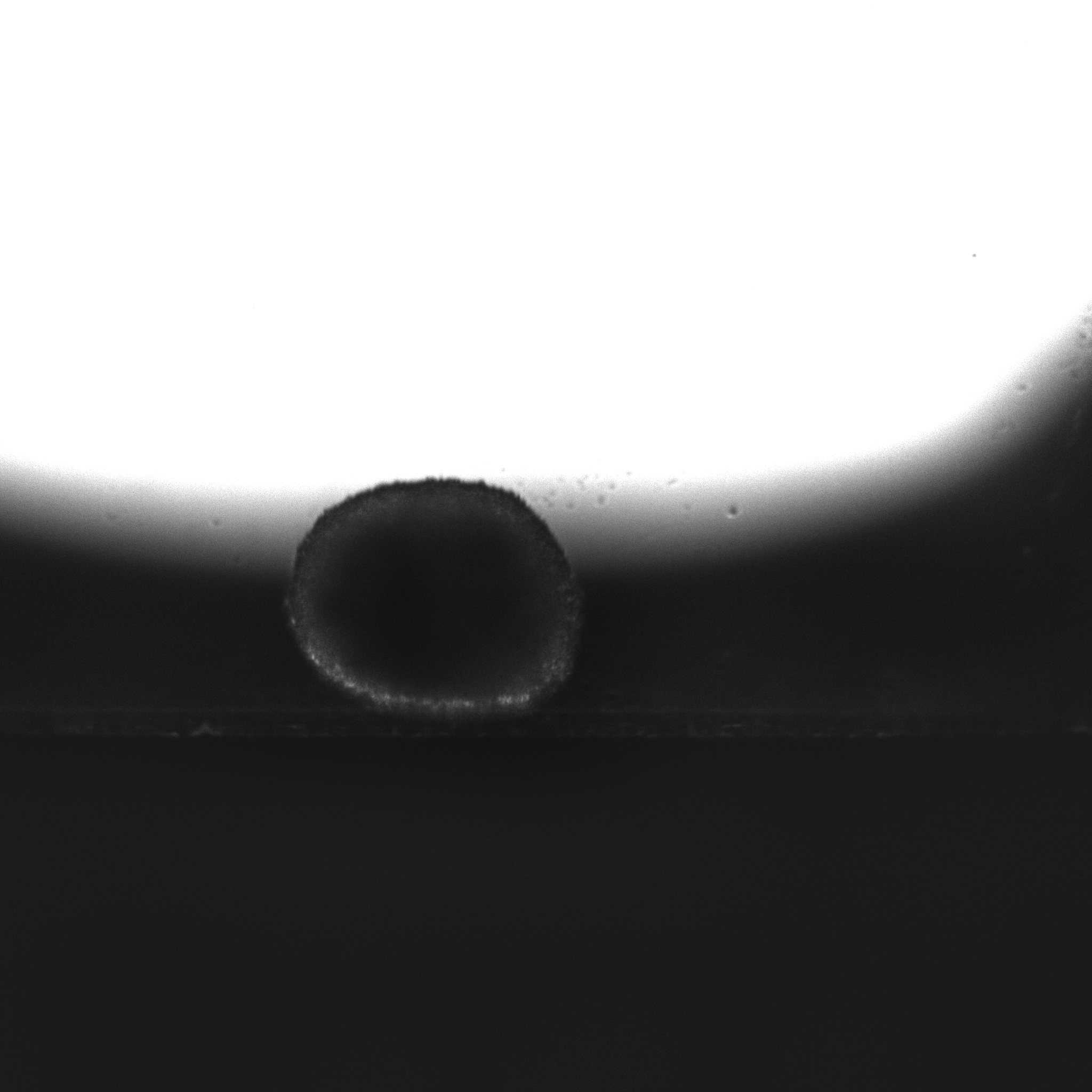

Supplement: Supplementary file 11 — Source Data for Figure 6 [file EMMM-15-e18199-s003.zip › Figure_6/6B/B'_Treatment_PDO_T#5_BF_4.tif]

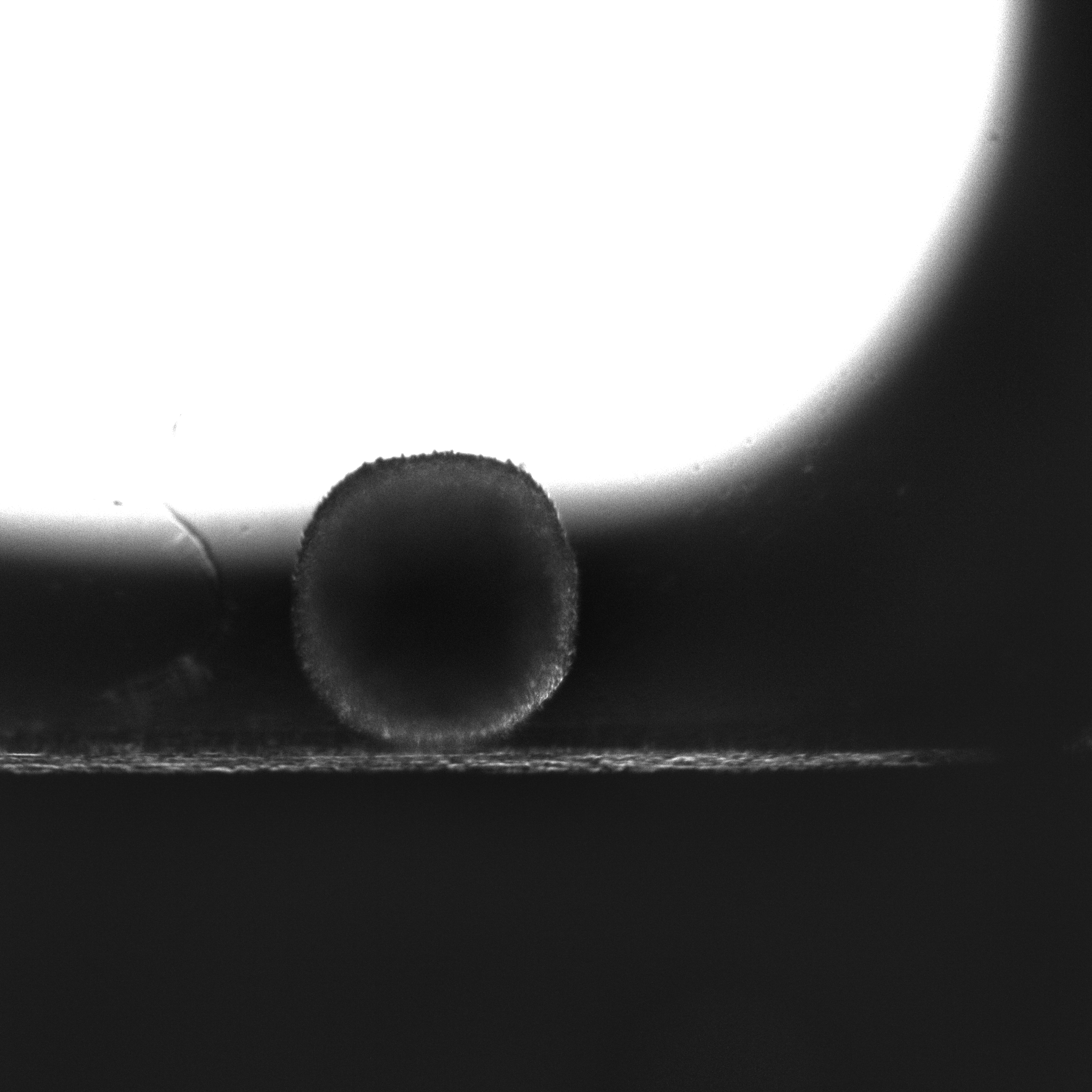

Supplement: Supplementary file 11 — Source Data for Figure 6 [file EMMM-15-e18199-s003.zip › Figure_6/6B/B'_Treatment_PDO_T#5_BF_5.tif]

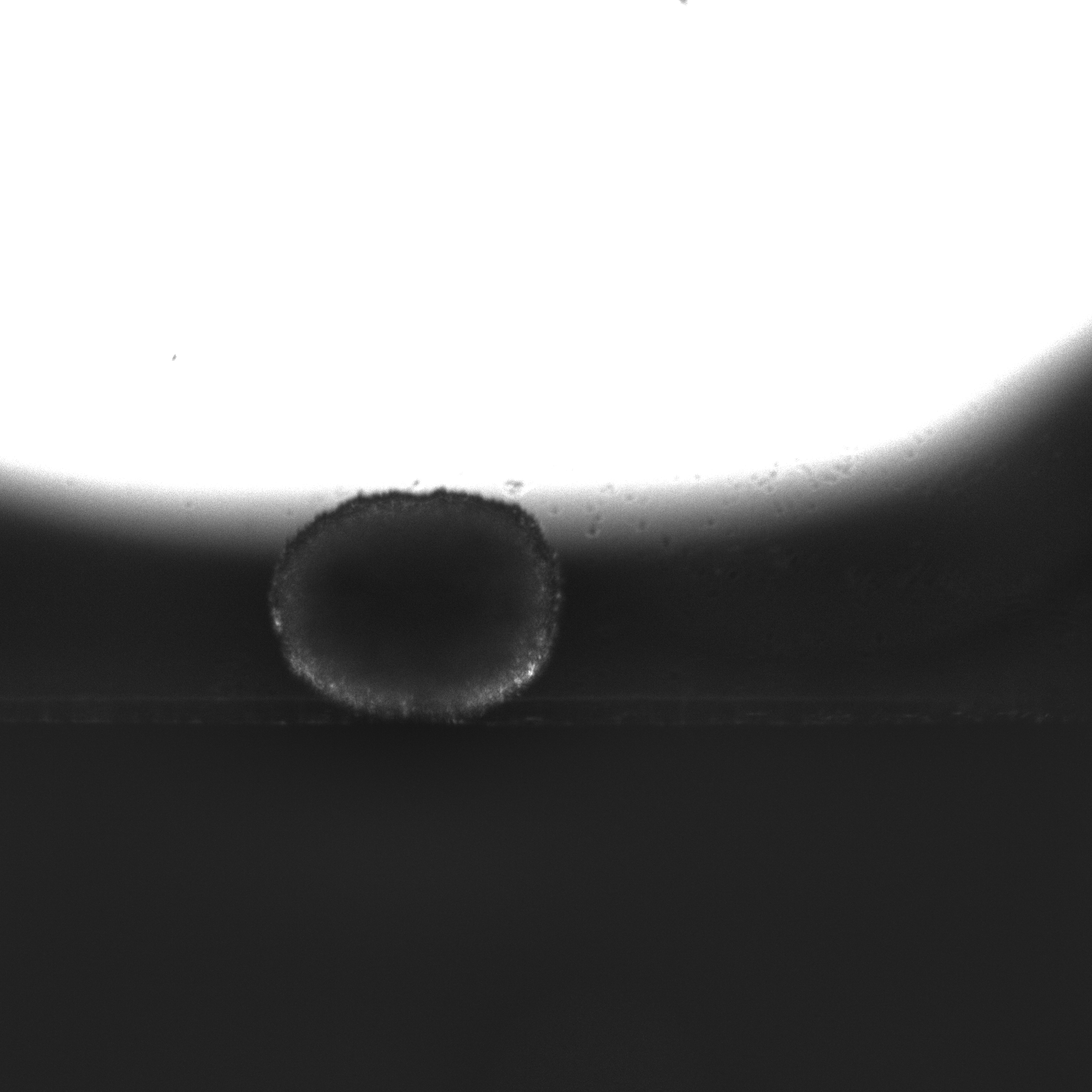

Supplement: Supplementary file 11 — Source Data for Figure 6 [file EMMM-15-e18199-s003.zip › Figure_6/6B/B'_Treatment_PDO_T#5_BF_6.tif]

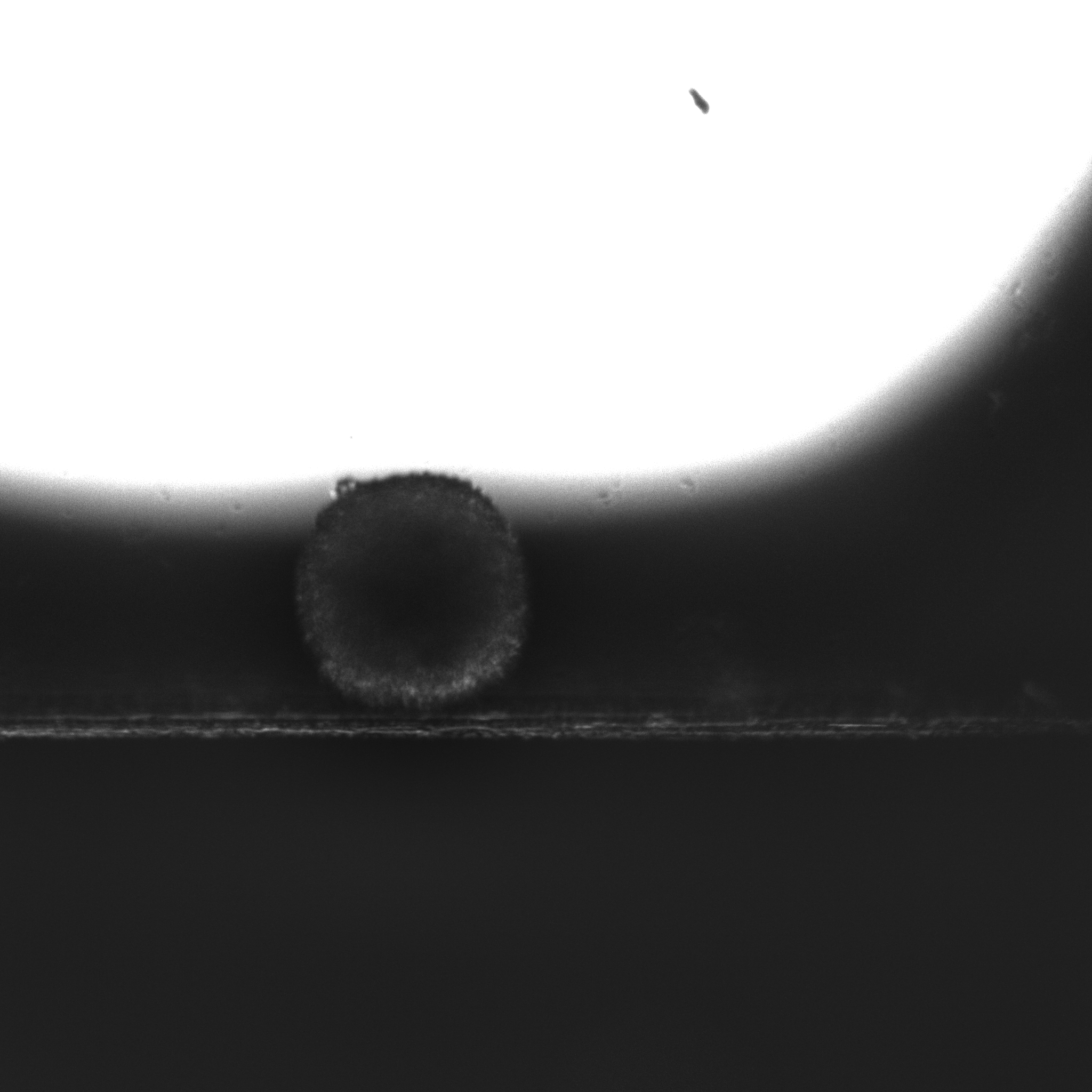

Supplement: Supplementary file 11 — Source Data for Figure 6 [file EMMM-15-e18199-s003.zip › Figure_6/6B/B'_Treatment_PDO_T#5_BF_7.tif]

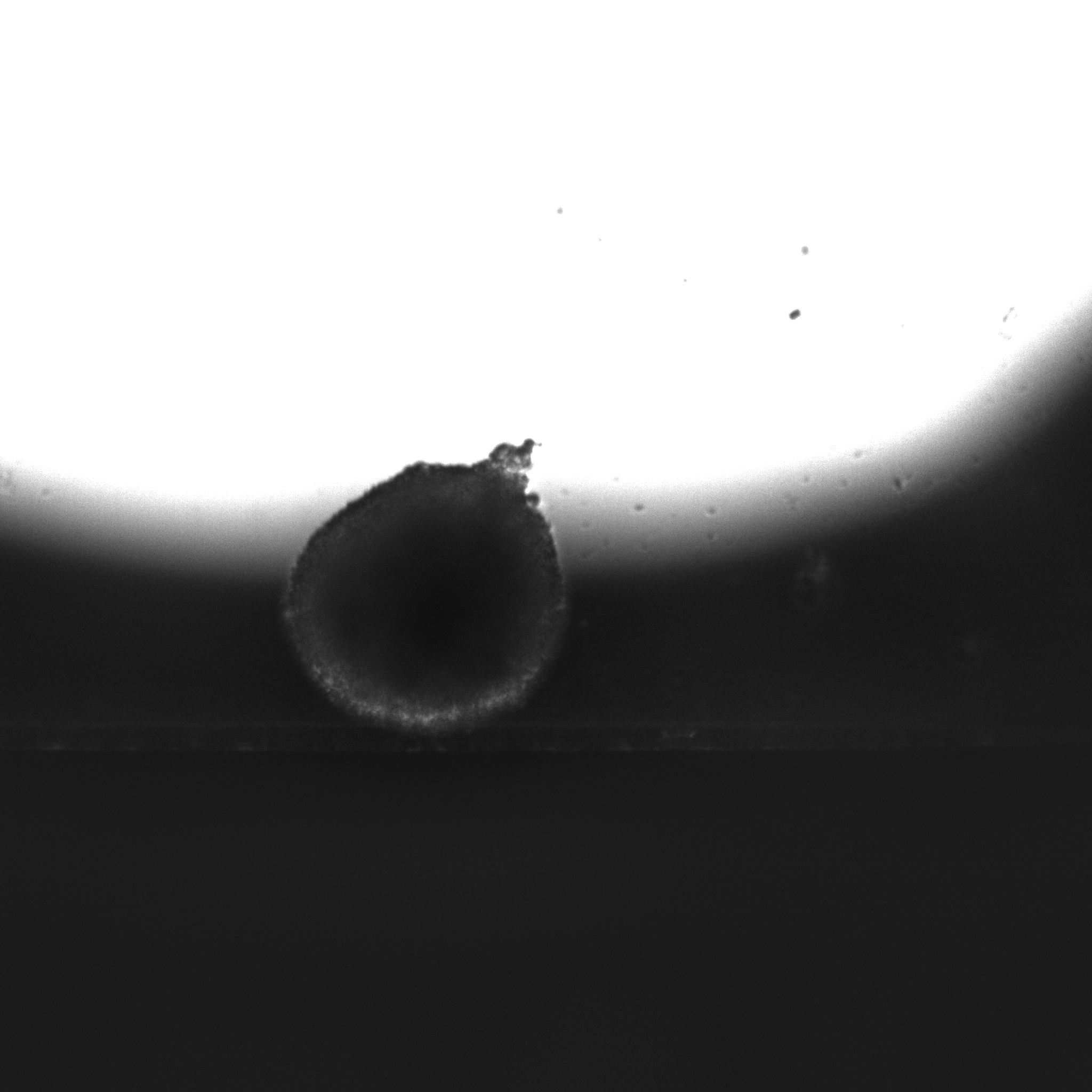

Supplement: Supplementary file 11 — Source Data for Figure 6 [file EMMM-15-e18199-s003.zip › Figure_6/6B/B'_Treatment_PDO_T#5_BF_8.tif]

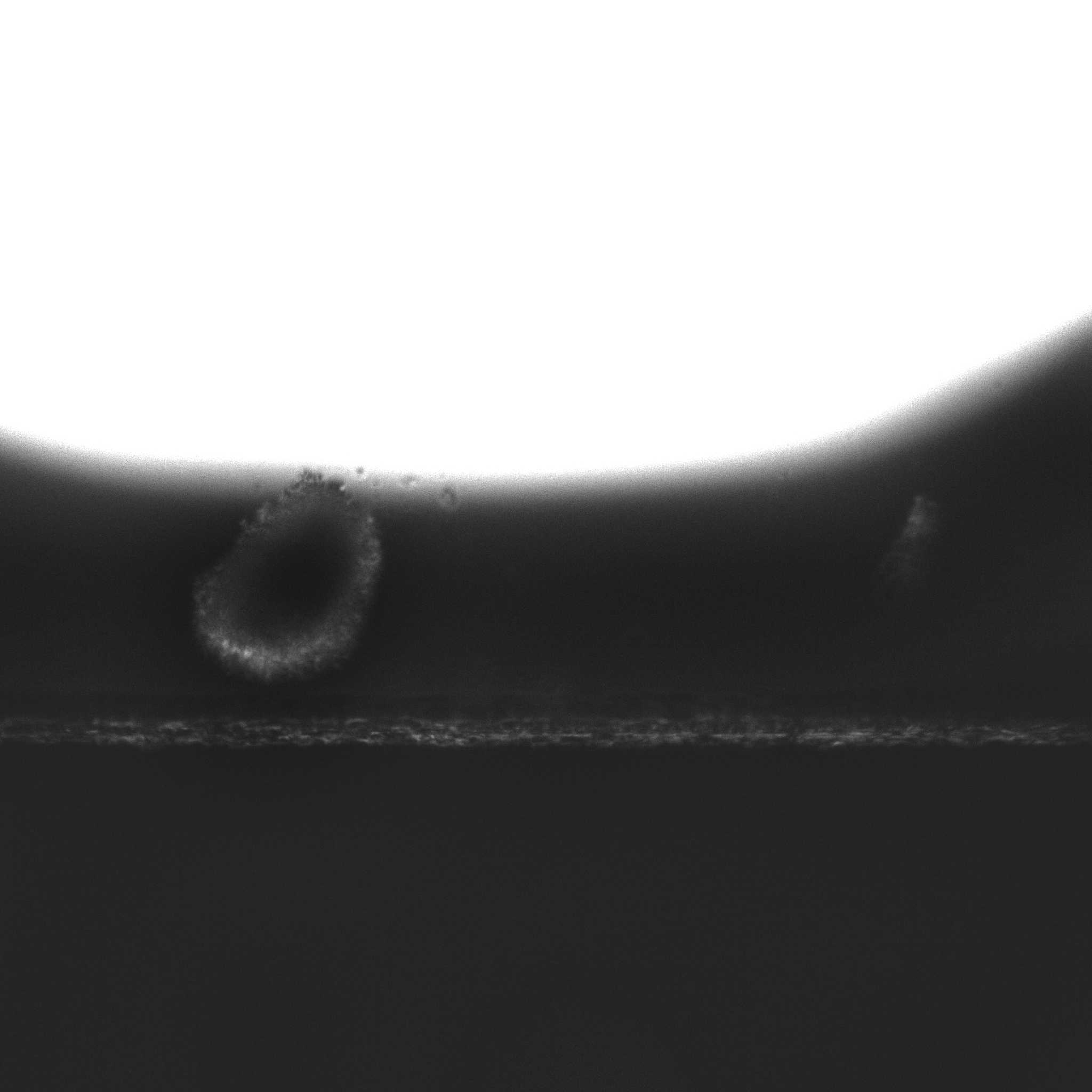

Supplement: Supplementary file 11 — Source Data for Figure 6 [file EMMM-15-e18199-s003.zip › Figure_6/6B/B'_Treatment_PDO_T#5_BF_9.tif]

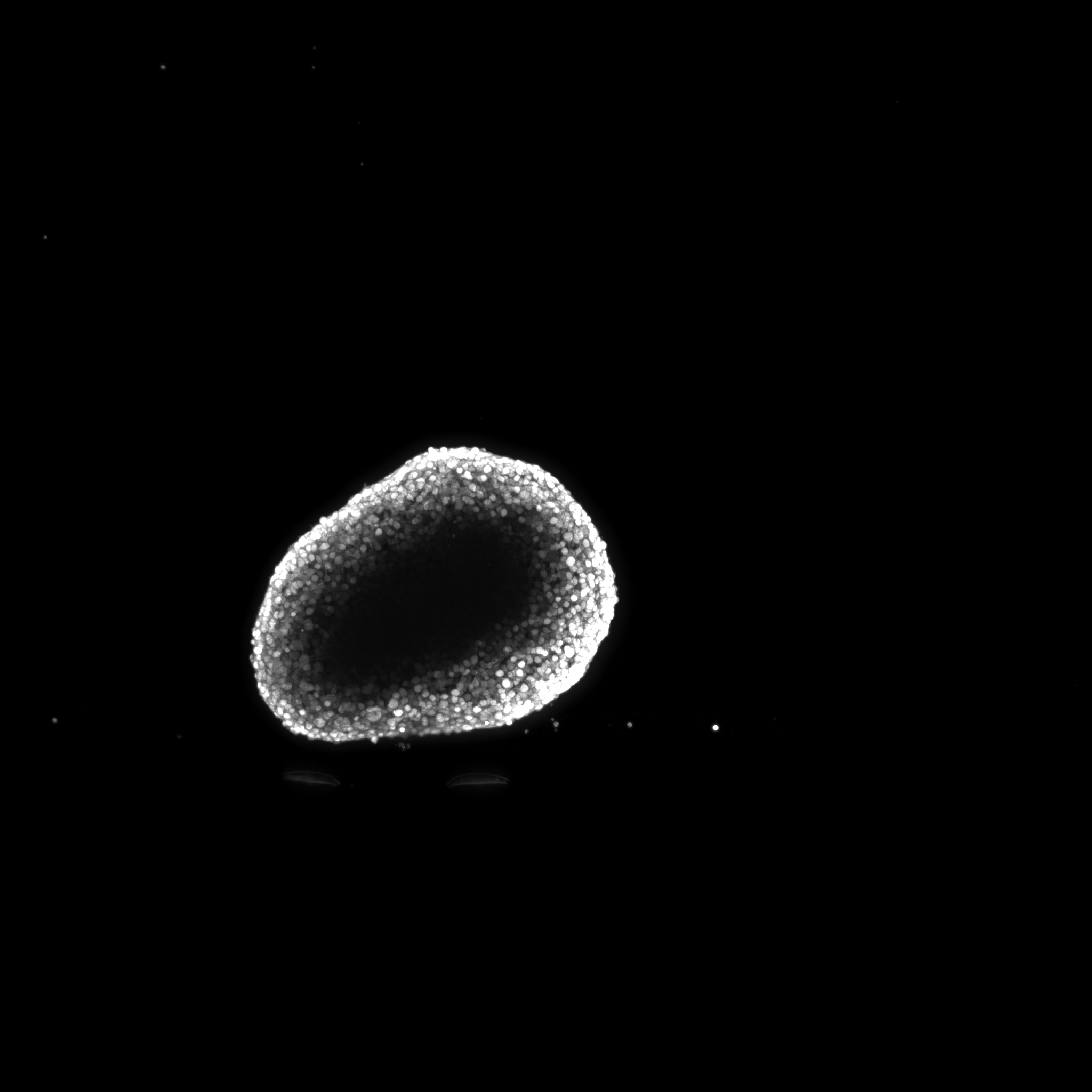

Supplement: Supplementary file 11 — Source Data for Figure 6 [file EMMM-15-e18199-s003.zip › Figure_6/6B/B'_Treatment_PDO_T#5_FLUO_1.tif]

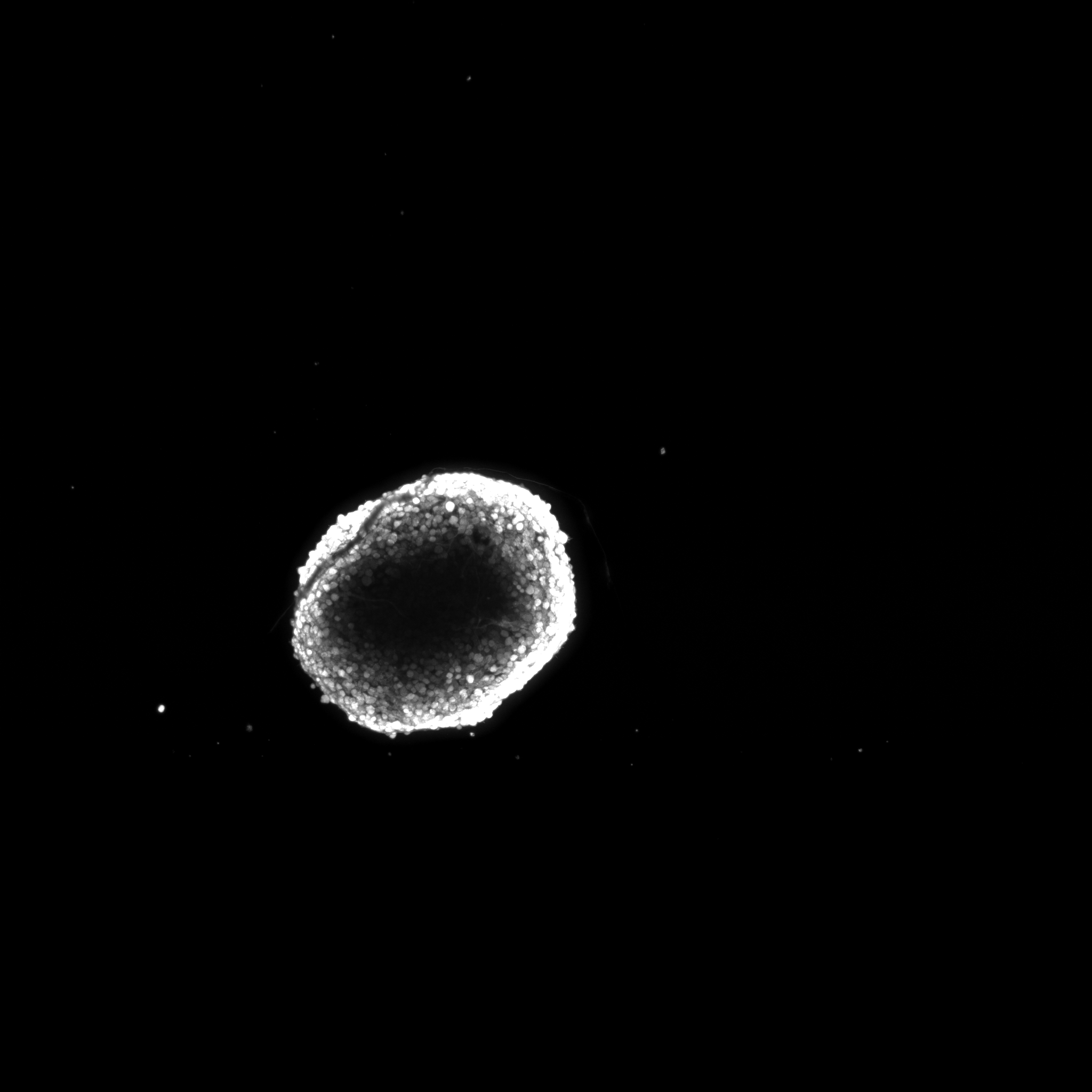

Supplement: Supplementary file 11 — Source Data for Figure 6 [file EMMM-15-e18199-s003.zip › Figure_6/6B/B'_Treatment_PDO_T#5_FLUO_2.tif]

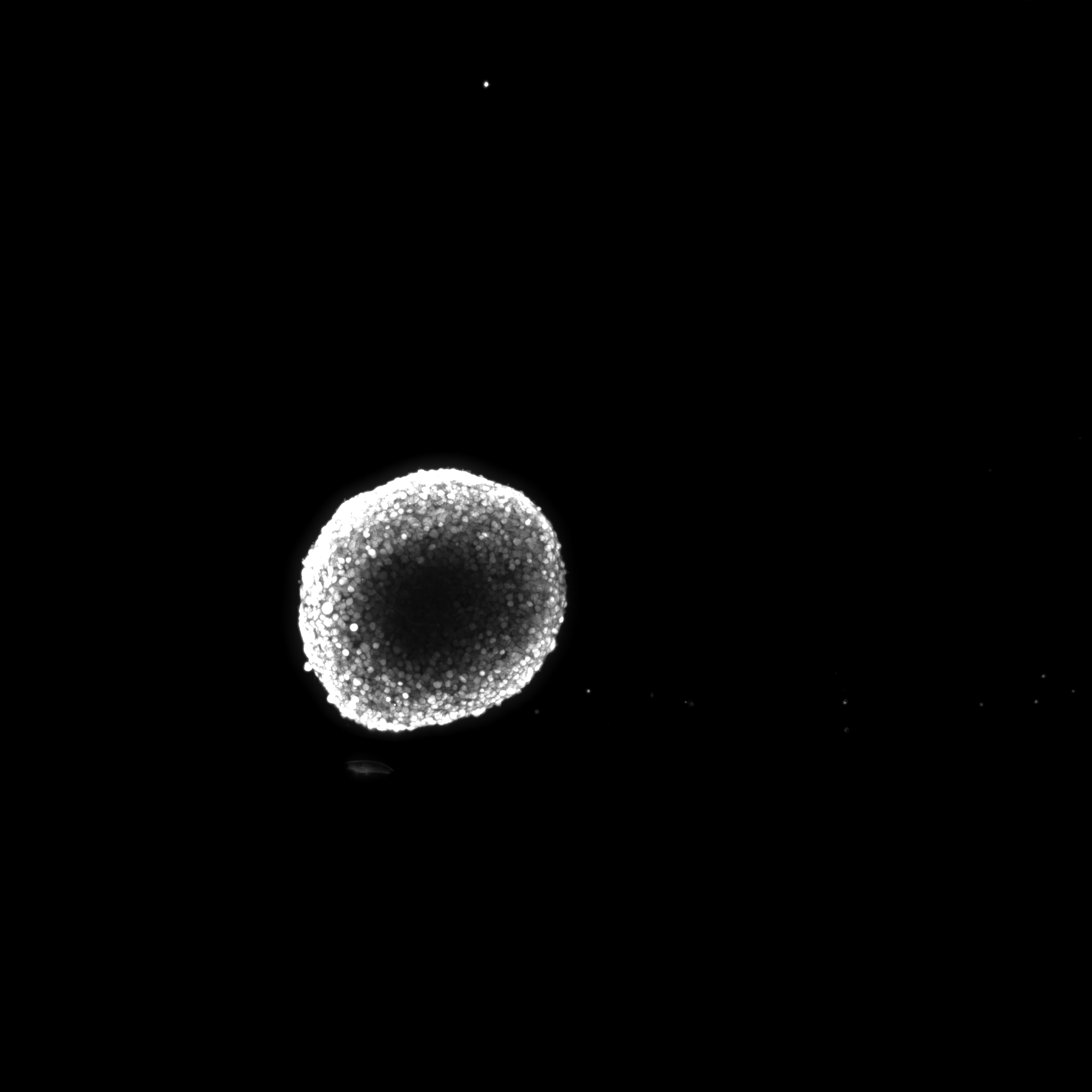

Supplement: Supplementary file 11 — Source Data for Figure 6 [file EMMM-15-e18199-s003.zip › Figure_6/6B/B'_Treatment_PDO_T#5_FLUO_3.tif]

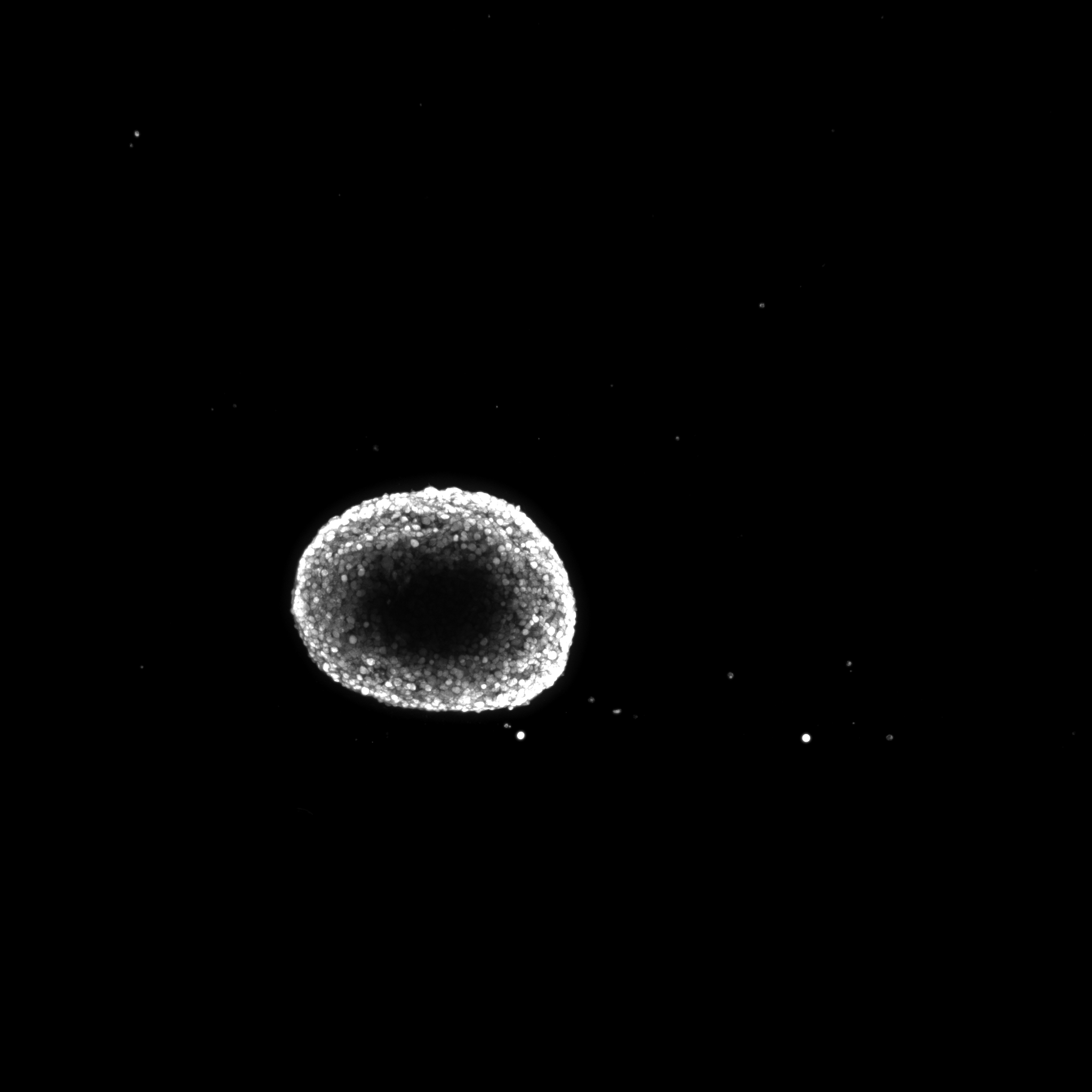

Supplement: Supplementary file 11 — Source Data for Figure 6 [file EMMM-15-e18199-s003.zip › Figure_6/6B/B'_Treatment_PDO_T#5_FLUO_4.tif]

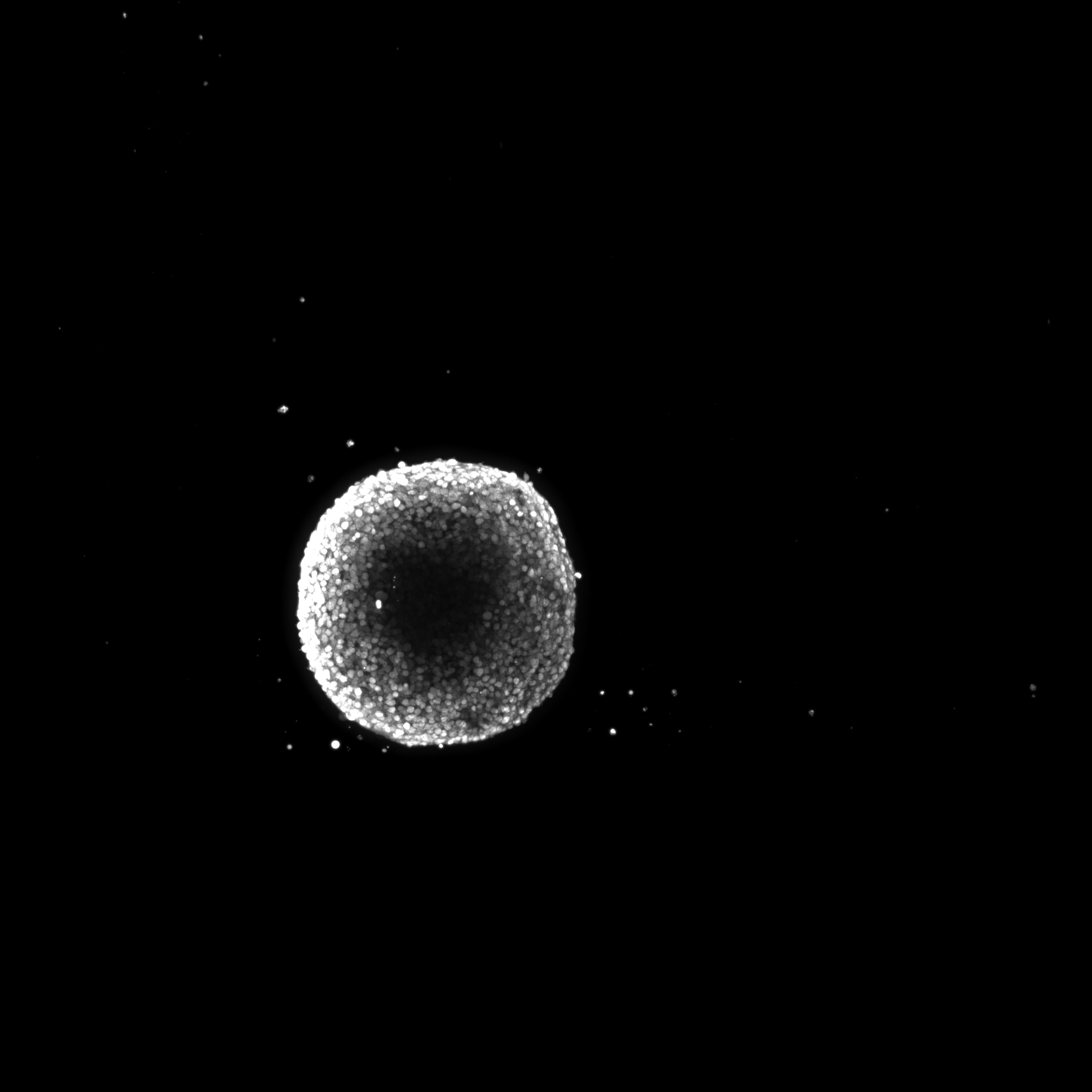

Supplement: Supplementary file 11 — Source Data for Figure 6 [file EMMM-15-e18199-s003.zip › Figure_6/6B/B'_Treatment_PDO_T#5_FLUO_5.tif]

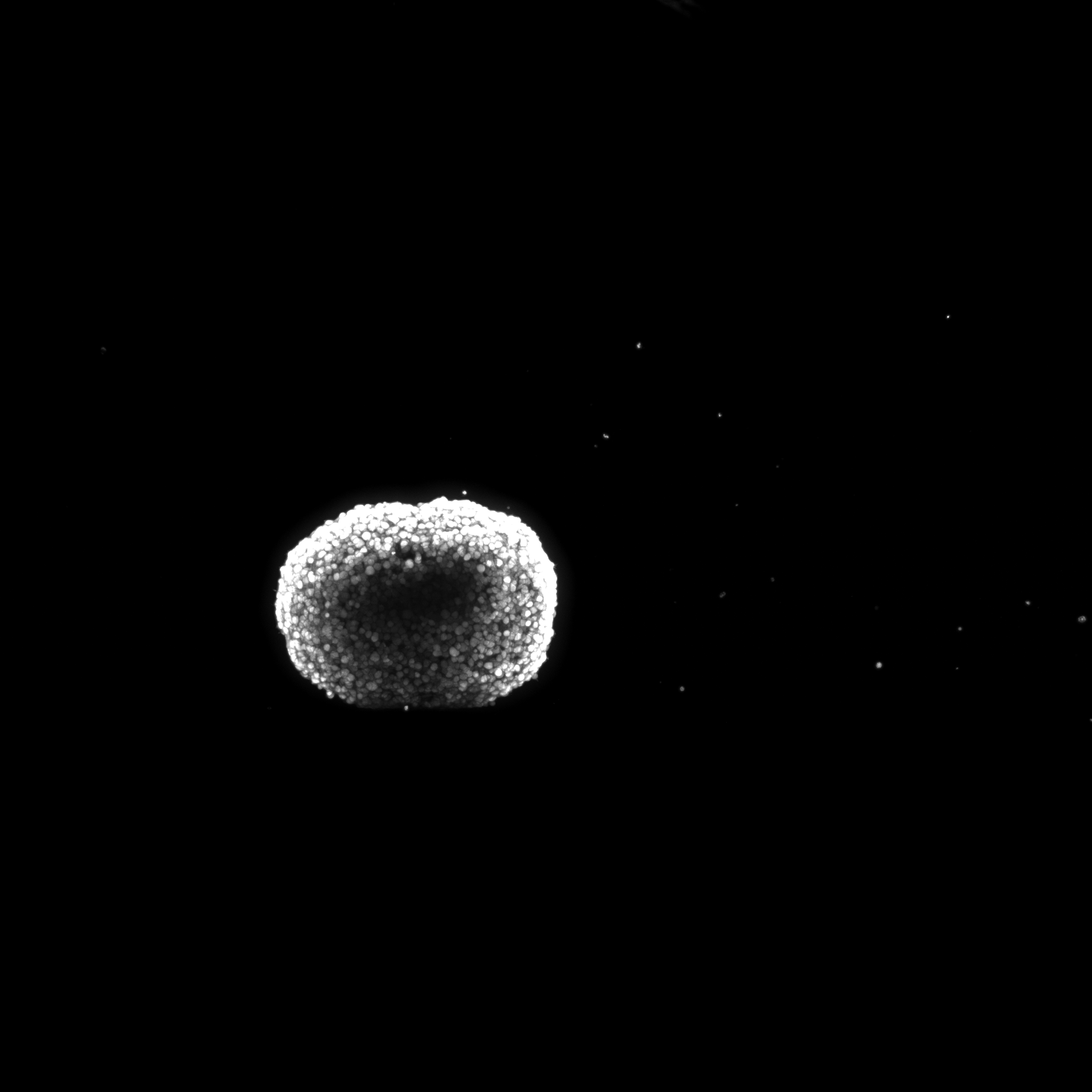

Supplement: Supplementary file 11 — Source Data for Figure 6 [file EMMM-15-e18199-s003.zip › Figure_6/6B/B'_Treatment_PDO_T#5_FLUO_6.tif]

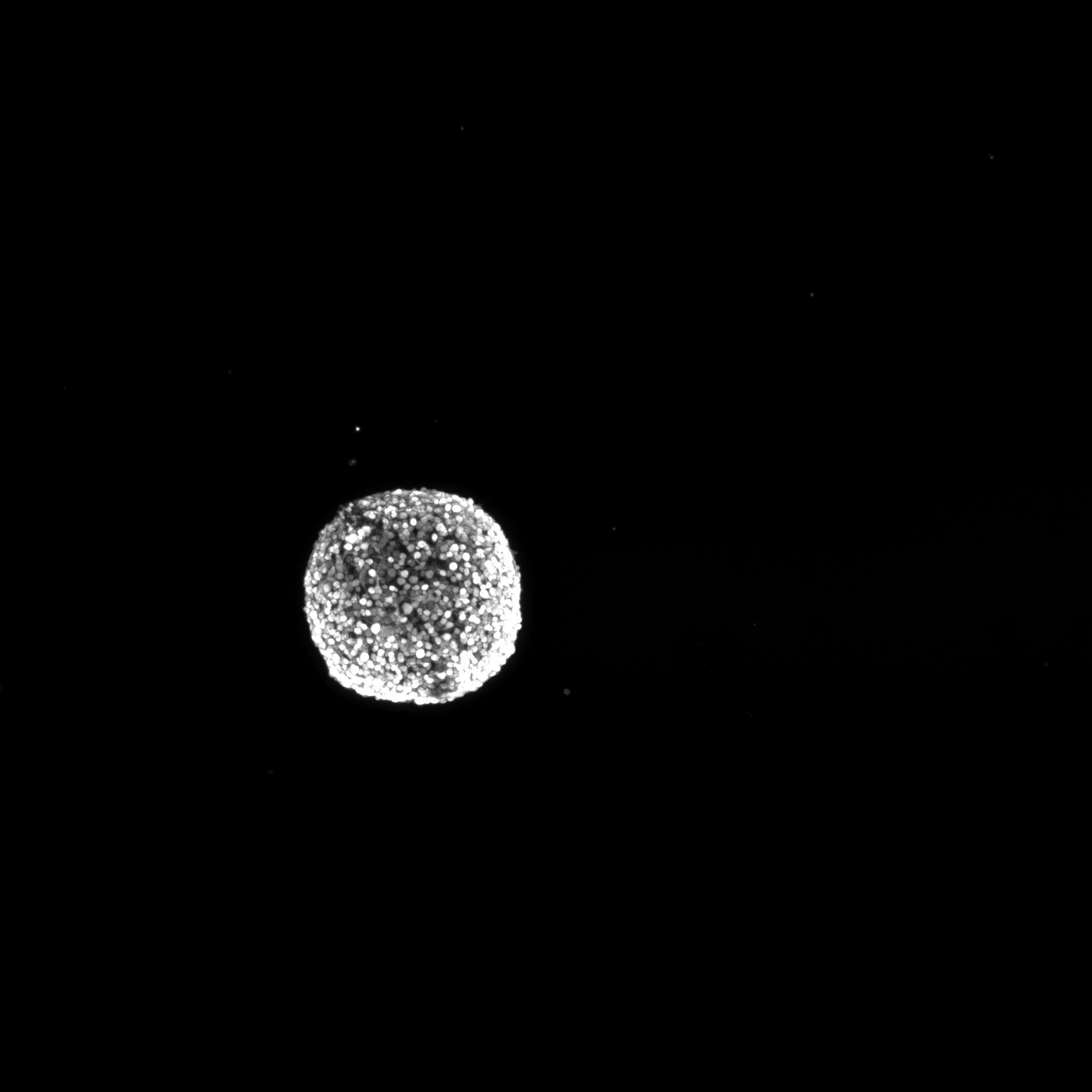

Supplement: Supplementary file 11 — Source Data for Figure 6 [file EMMM-15-e18199-s003.zip › Figure_6/6B/B'_Treatment_PDO_T#5_FLUO_7.tif]

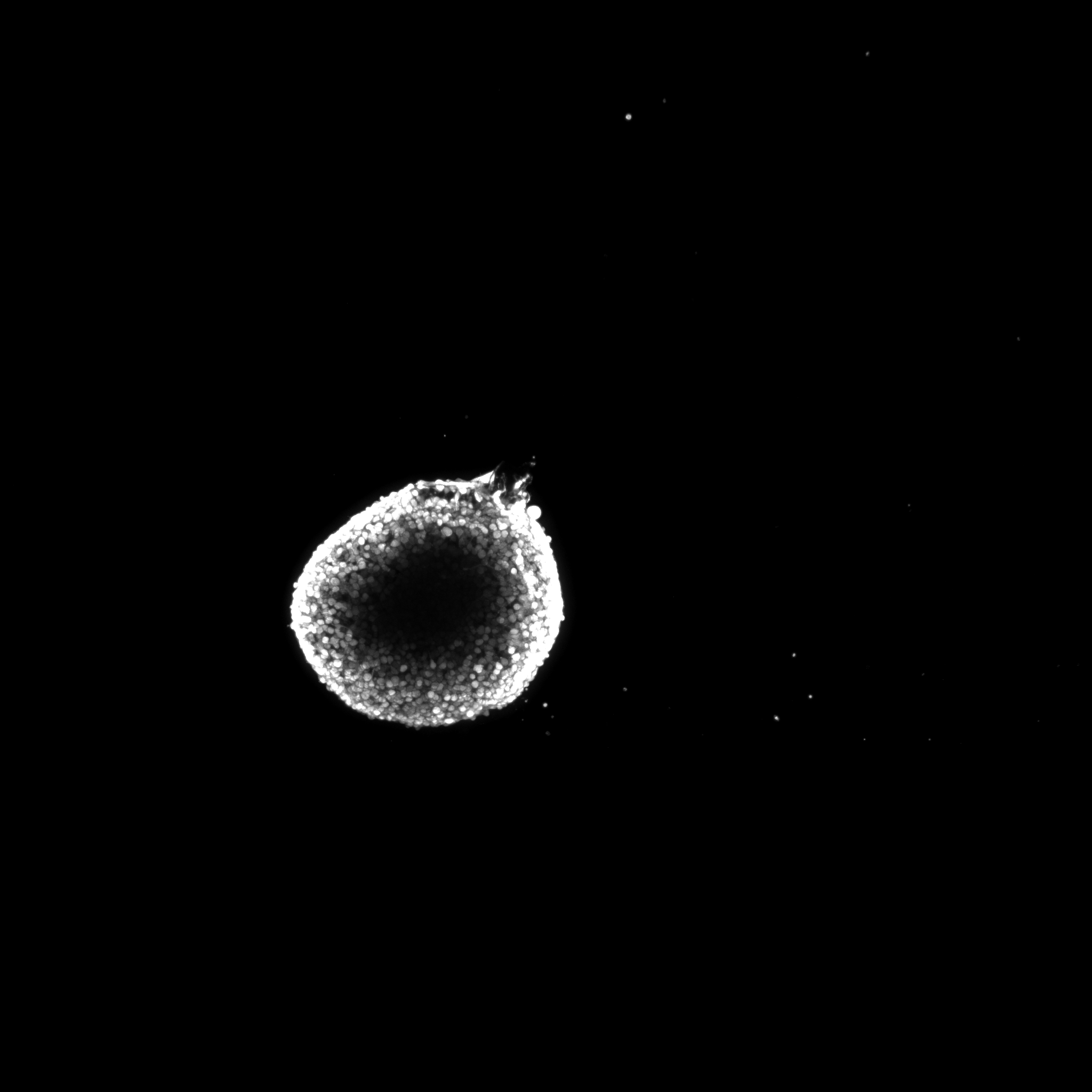

Supplement: Supplementary file 11 — Source Data for Figure 6 [file EMMM-15-e18199-s003.zip › Figure_6/6B/B'_Treatment_PDO_T#5_FLUO_8.tif]

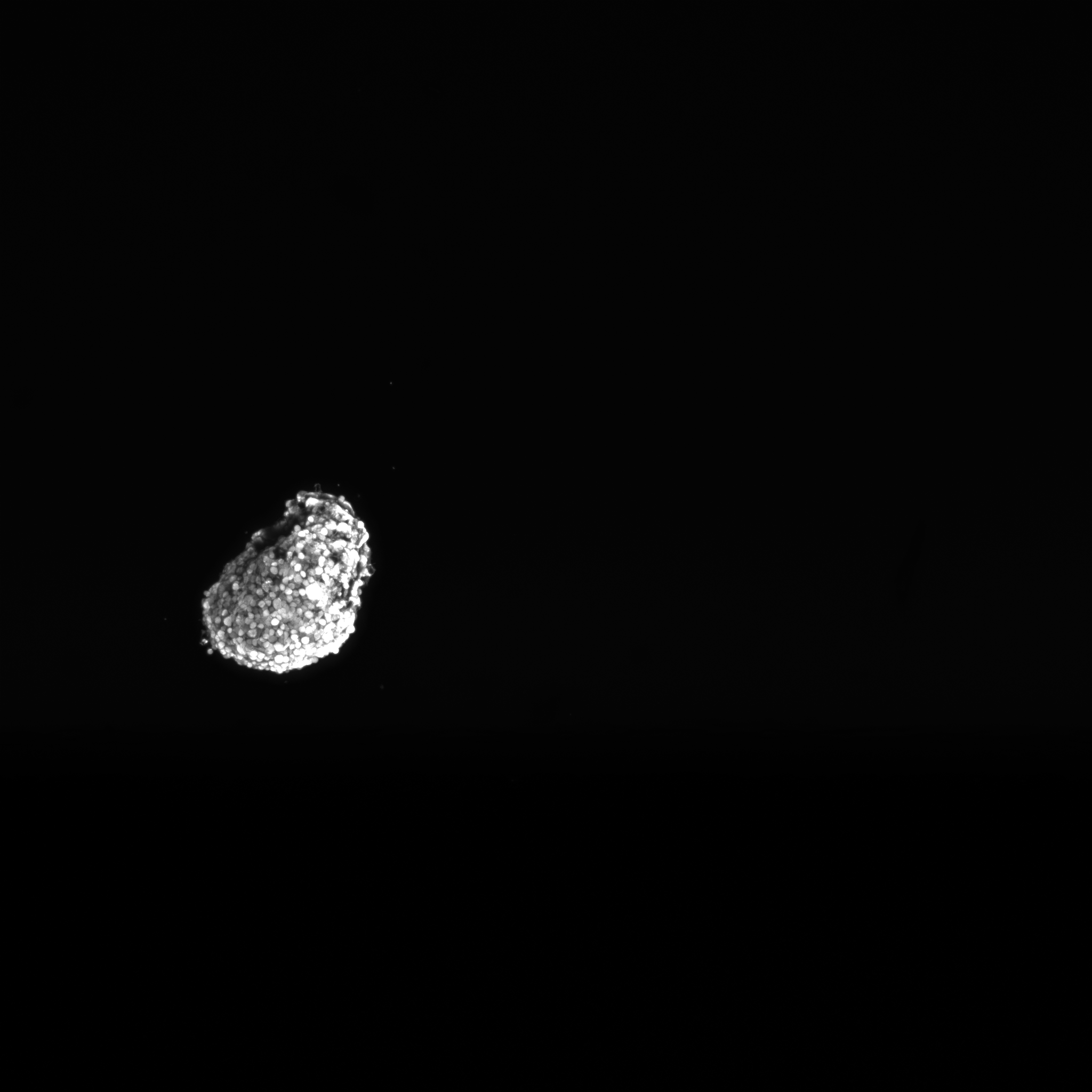

Supplement: Supplementary file 11 — Source Data for Figure 6 [file EMMM-15-e18199-s003.zip › Figure_6/6B/B'_Treatment_PDO_T#5_FLUO_9.tif]

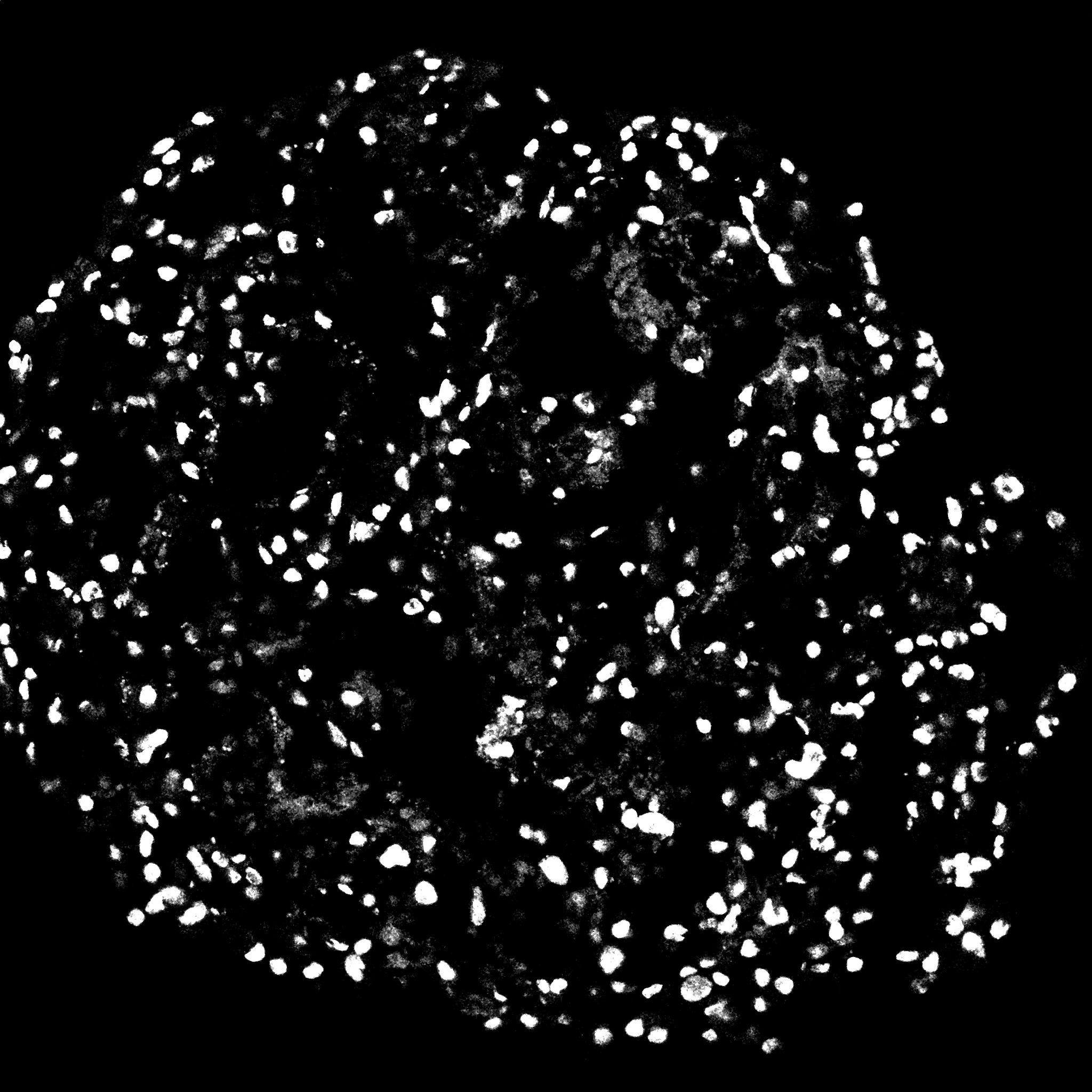

Supplement: Supplementary file 11 — Source Data for Figure 6 [file EMMM-15-e18199-s003.zip › Figure_6/6C/CTRL_PDO_T#5_Ki67_DAPI.tif]

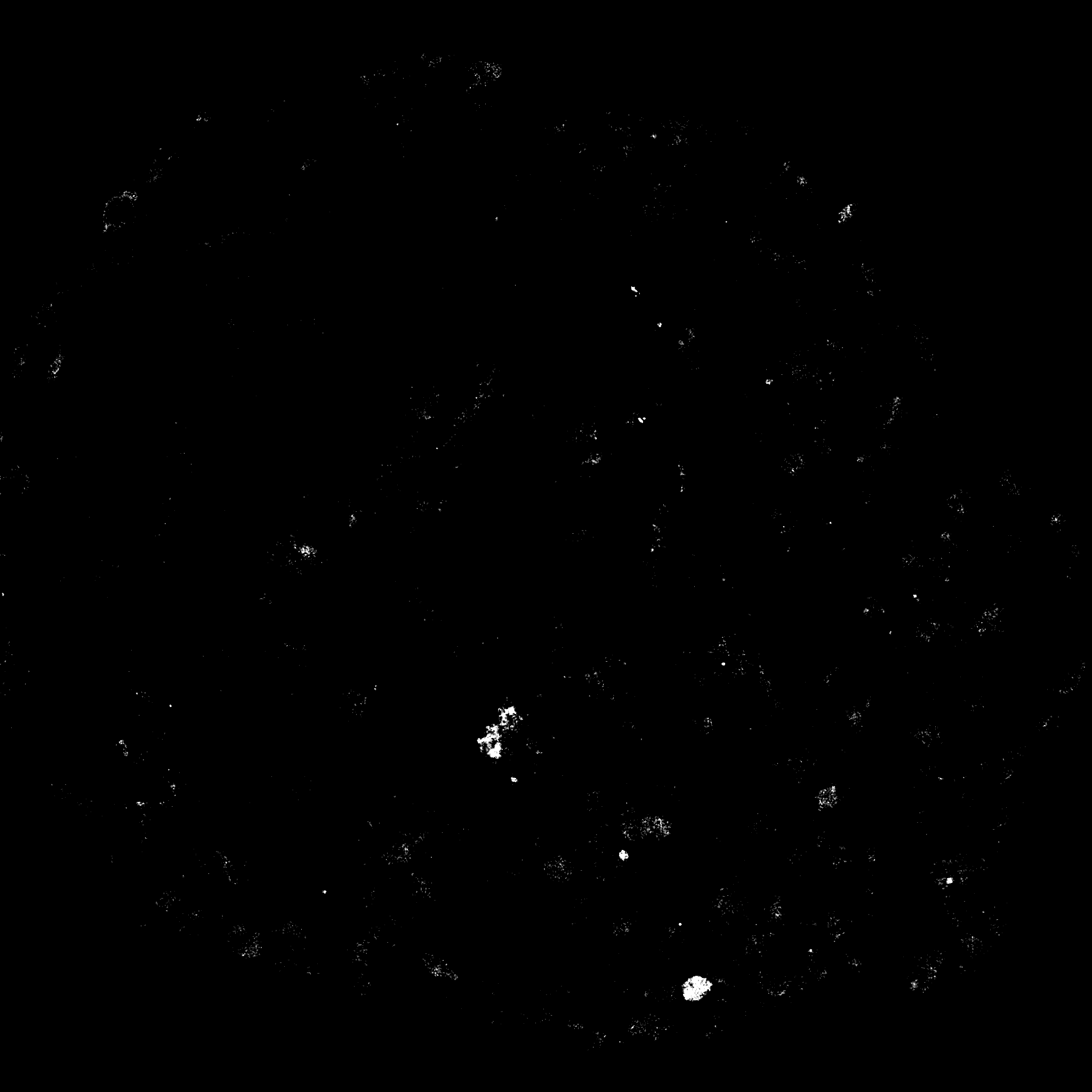

Supplement: Supplementary file 11 — Source Data for Figure 6 [file EMMM-15-e18199-s003.zip › Figure_6/6C/CTRL_PDO_T#5_Ki67_Ki67.tif]

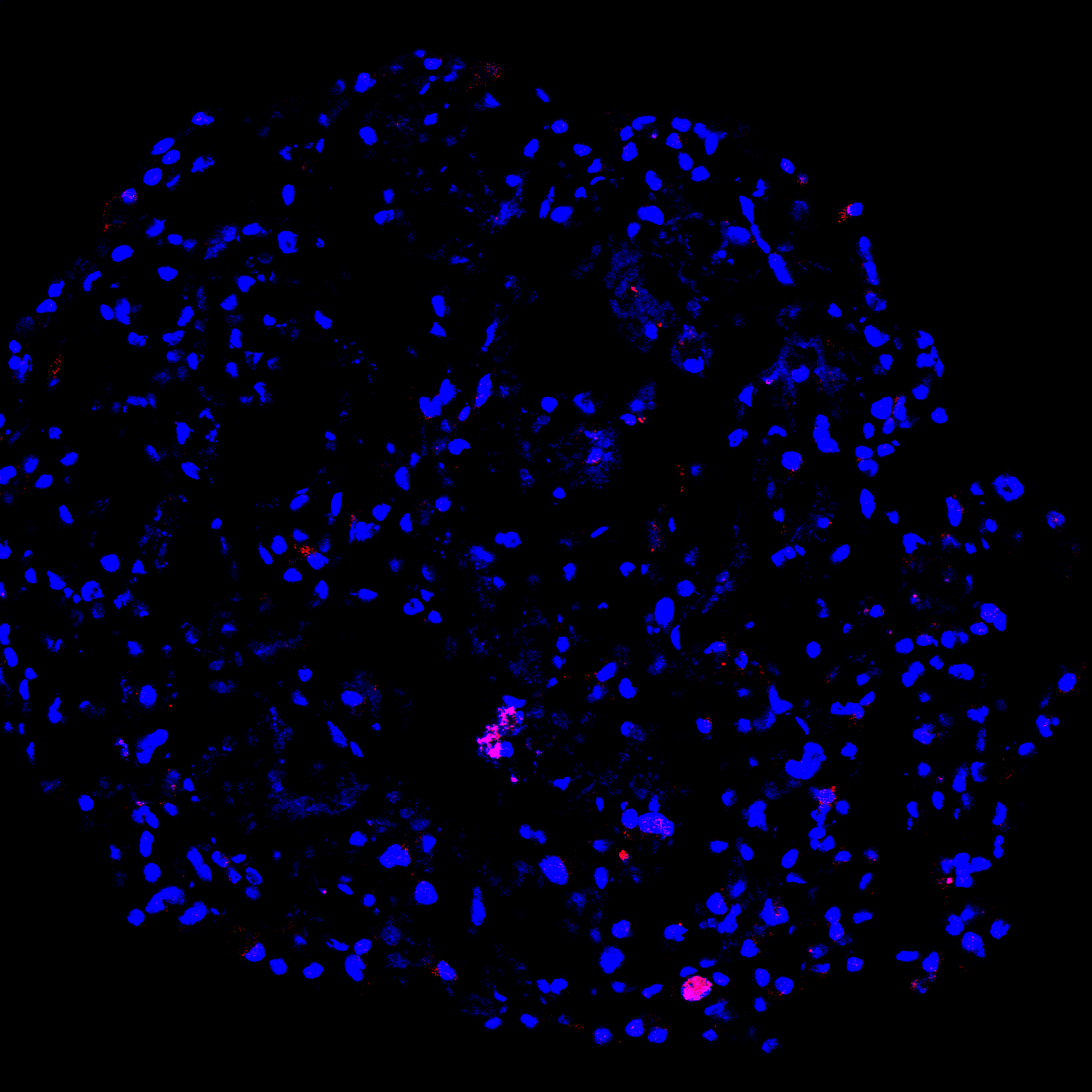

Supplement: Supplementary file 11 — Source Data for Figure 6 [file EMMM-15-e18199-s003.zip › Figure_6/6C/CTRL_PDO_T#5_Ki67_merge.tif]

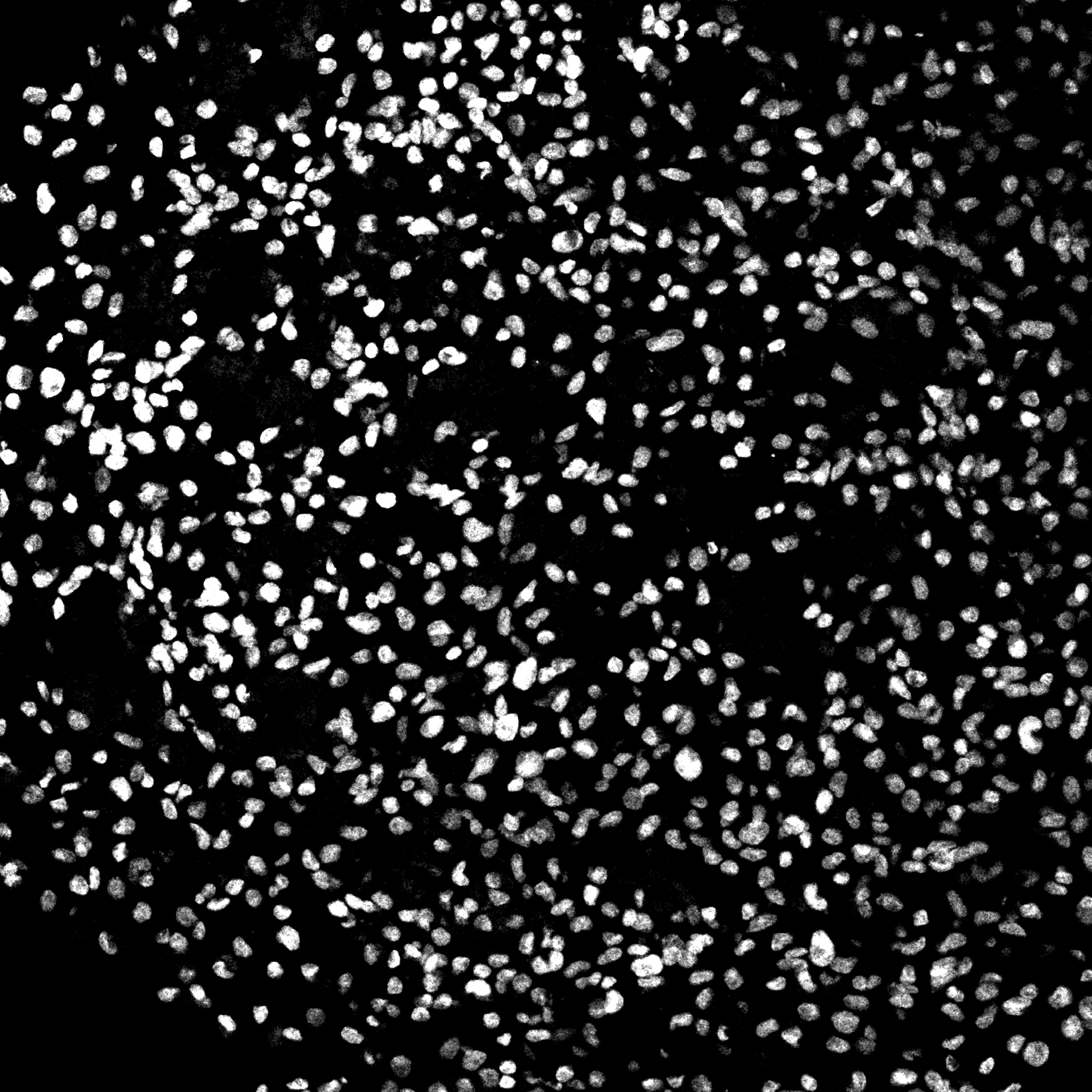

Supplement: Supplementary file 11 — Source Data for Figure 6 [file EMMM-15-e18199-s003.zip › Figure_6/6C/Treatment_PDO_T#5_Ki67_DAPI.tif]

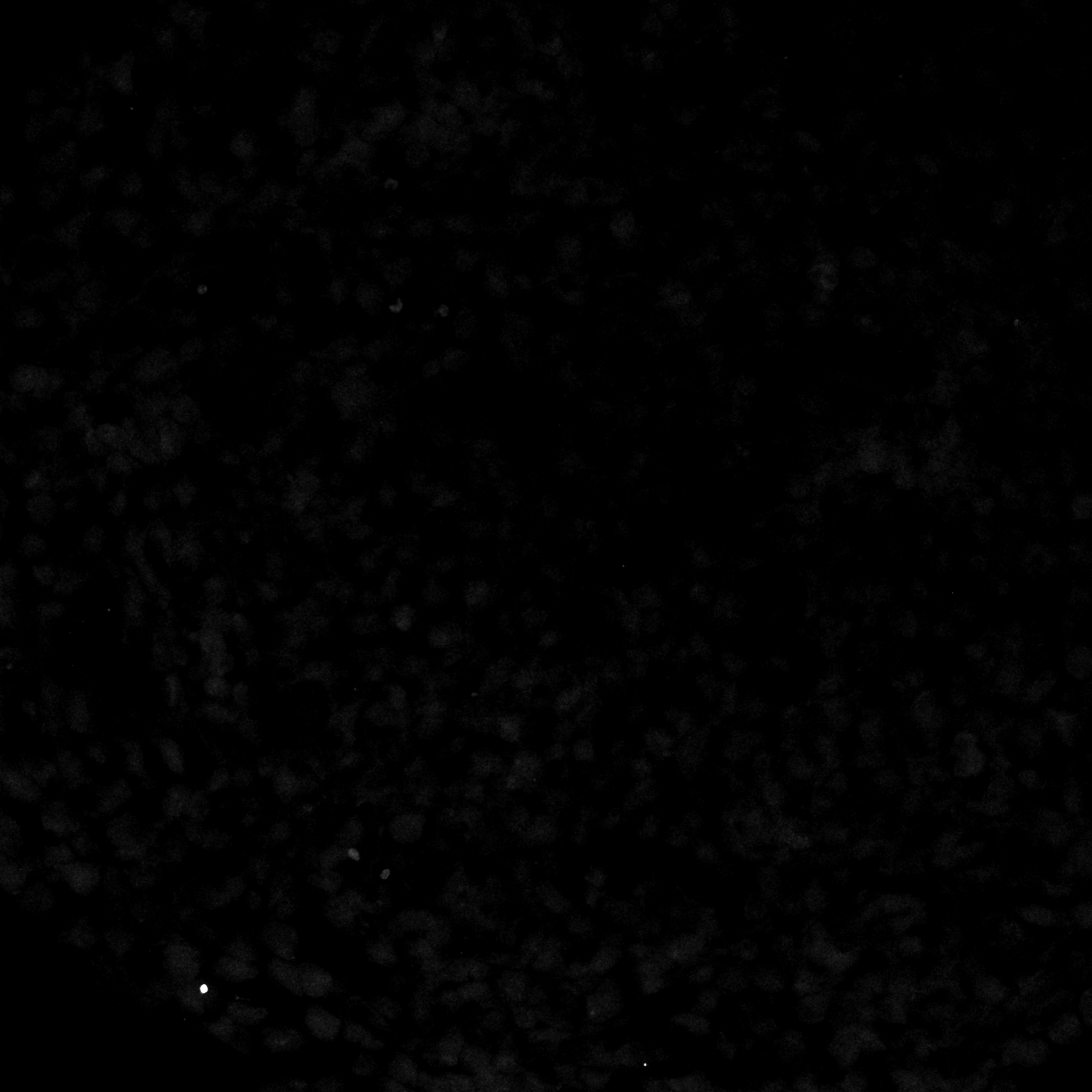

Supplement: Supplementary file 11 — Source Data for Figure 6 [file EMMM-15-e18199-s003.zip › Figure_6/6C/Treatment_PDO_T#5_Ki67_Ki67.tif]

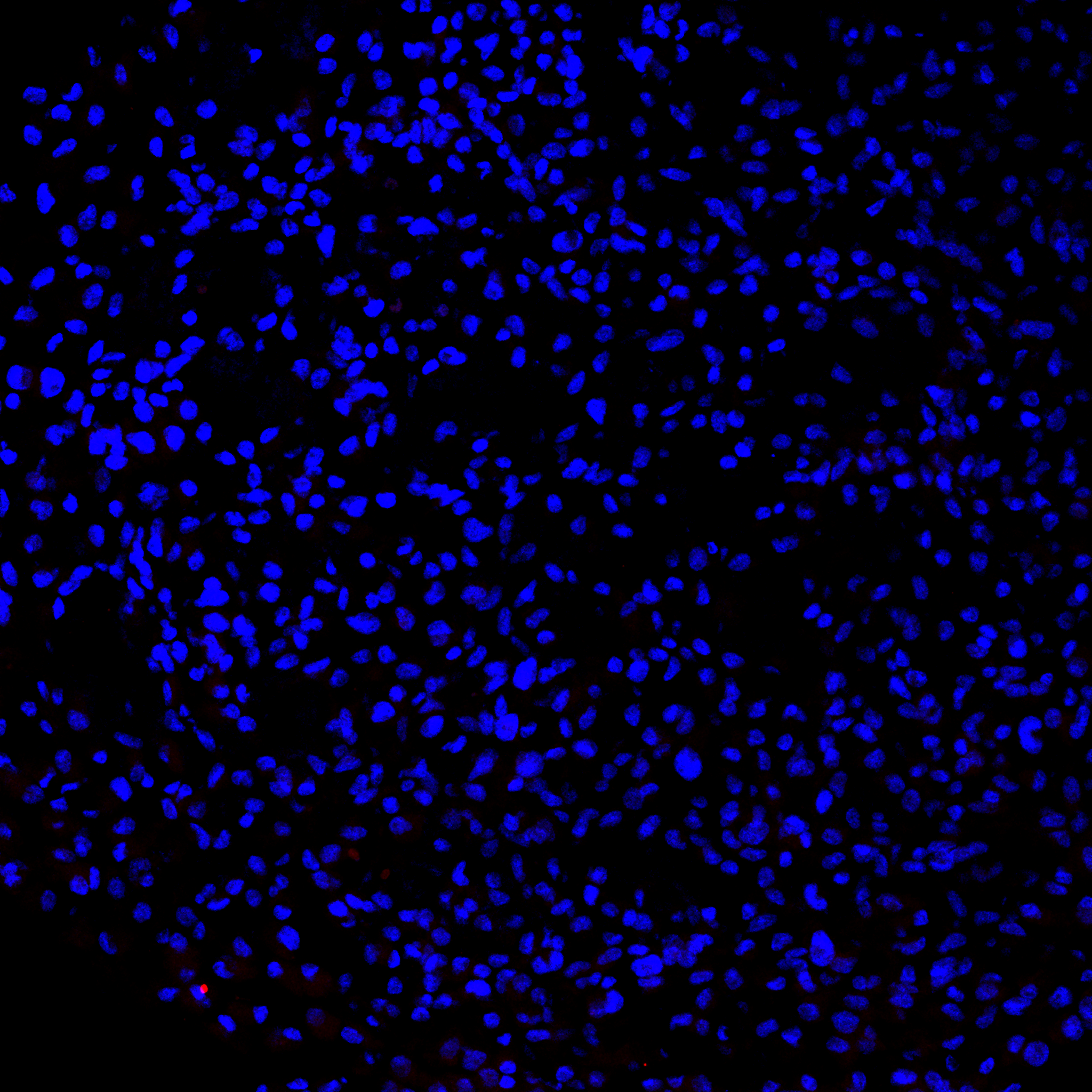

Supplement: Supplementary file 11 — Source Data for Figure 6 [file EMMM-15-e18199-s003.zip › Figure_6/6C/Treatment_PDO_T#5_Ki67_merge.tif]

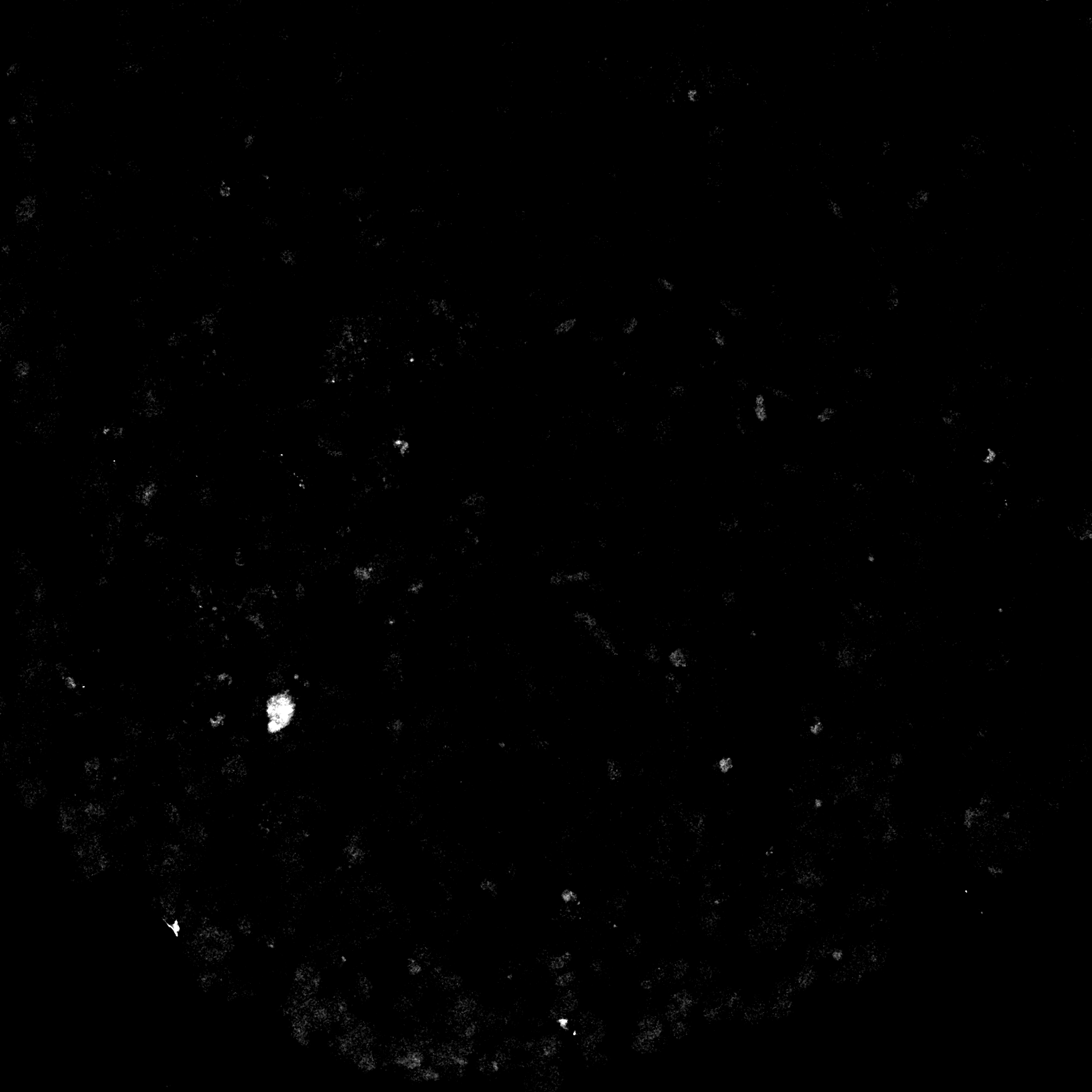

Supplement: Supplementary file 11 — Source Data for Figure 6 [file EMMM-15-e18199-s003.zip › Figure_6/6D/CTRL_PDO_T#5_Caspase3_Caspase3.tif]

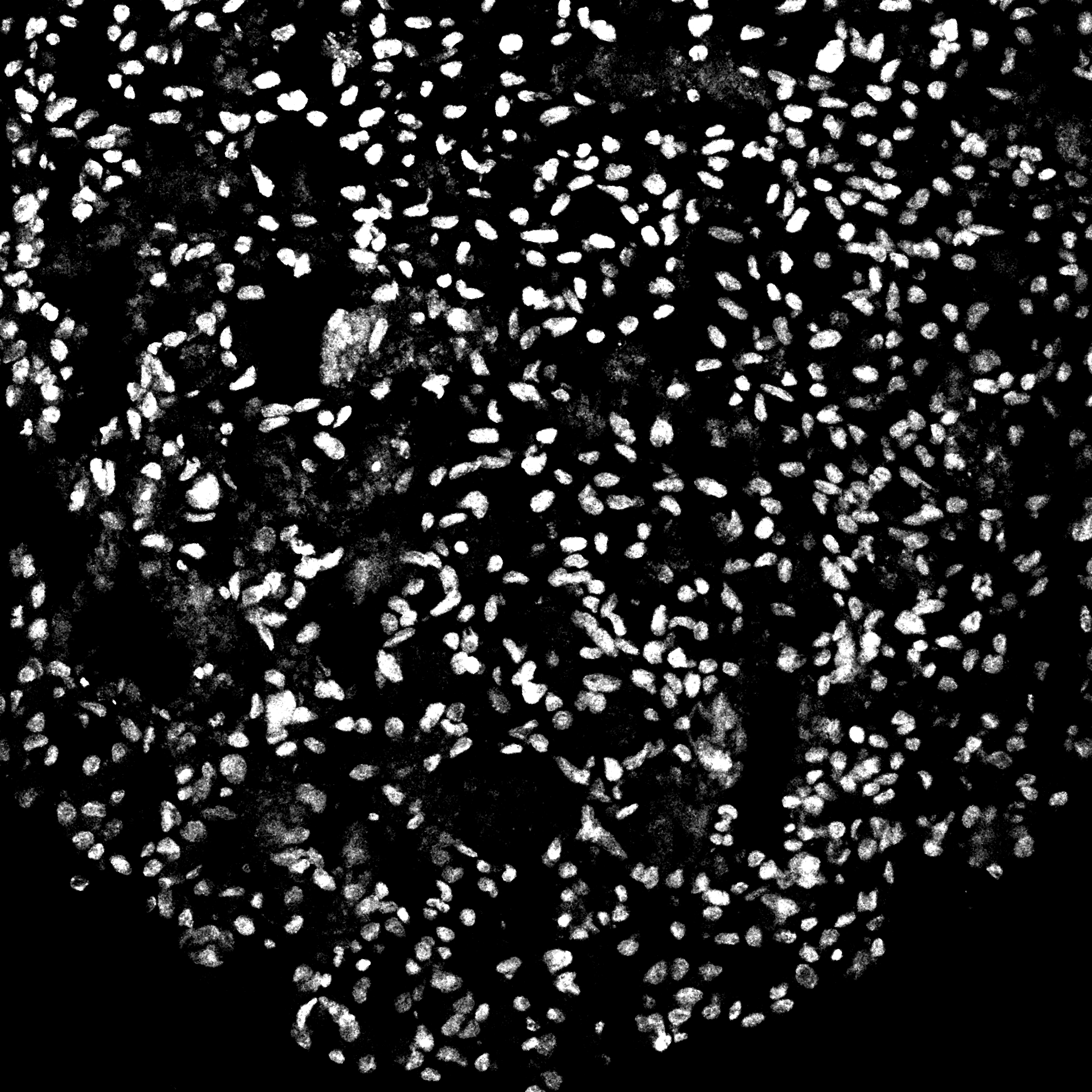

Supplement: Supplementary file 11 — Source Data for Figure 6 [file EMMM-15-e18199-s003.zip › Figure_6/6D/CTRL_PDO_T#5_Caspase3_DAPI.tif]

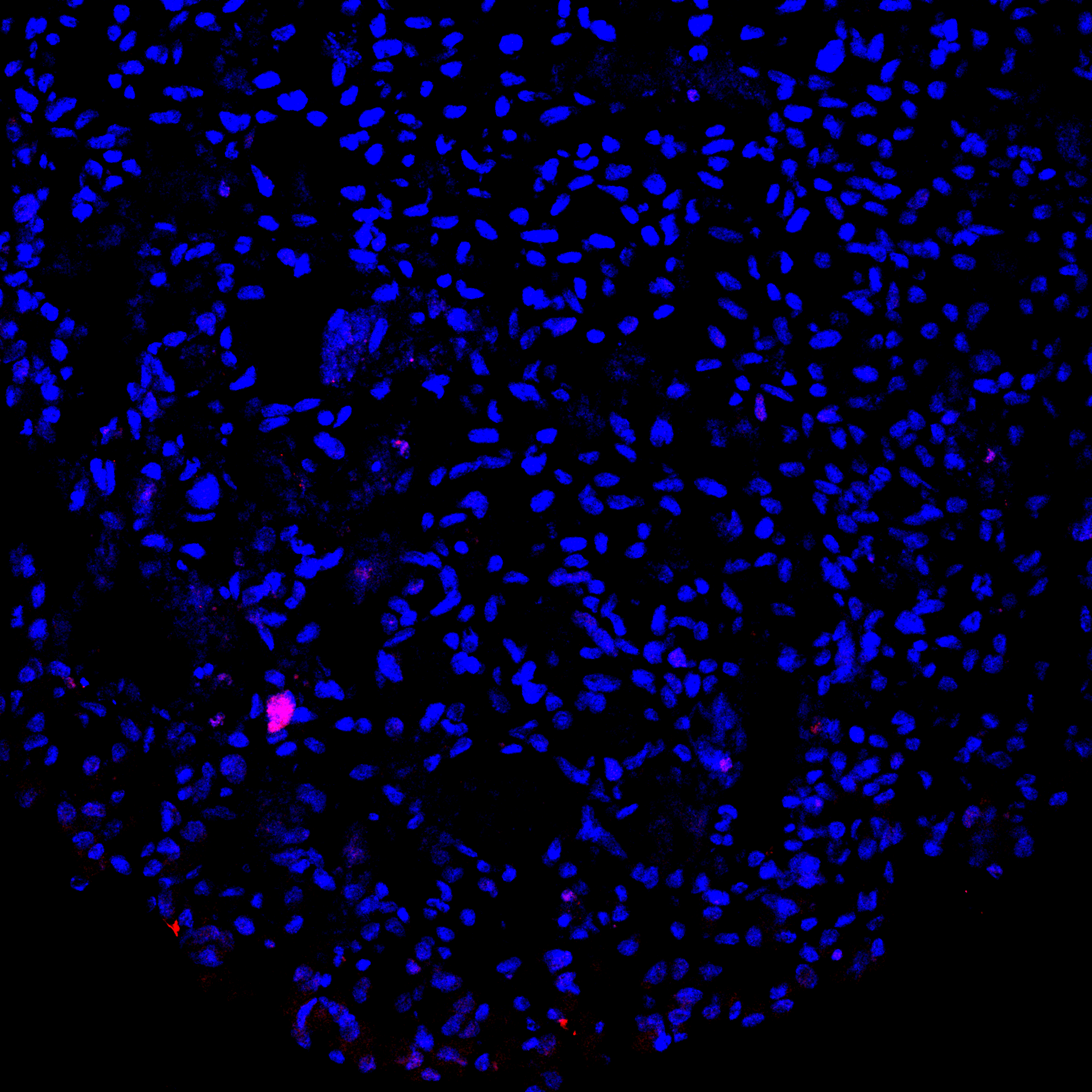

Supplement: Supplementary file 11 — Source Data for Figure 6 [file EMMM-15-e18199-s003.zip › Figure_6/6D/CTRL_PDO_T#5_Caspase3_merge.tif]

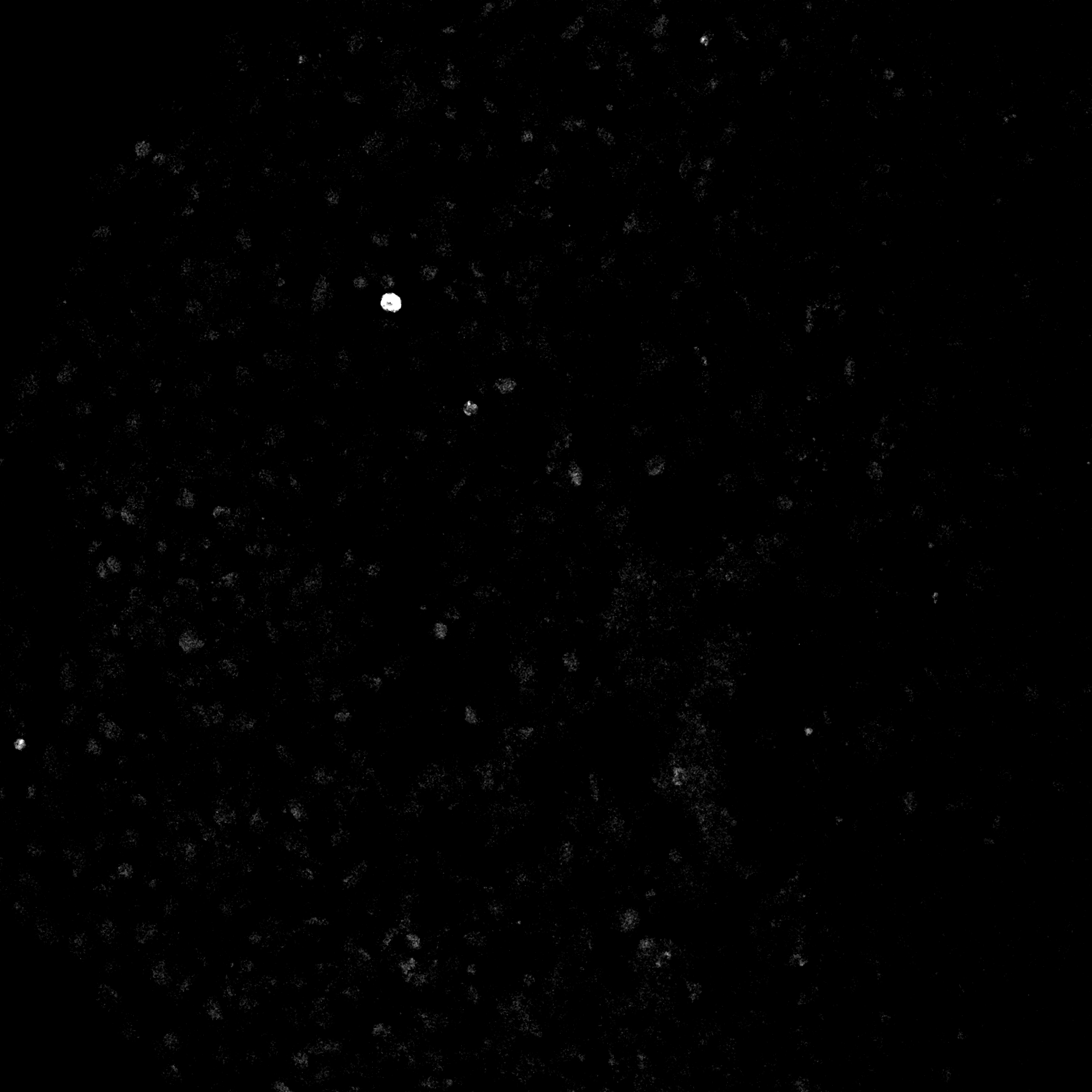

Supplement: Supplementary file 11 — Source Data for Figure 6 [file EMMM-15-e18199-s003.zip › Figure_6/6D/Treatment_PDO_T#5_Caspase3_Caspase3.tif]

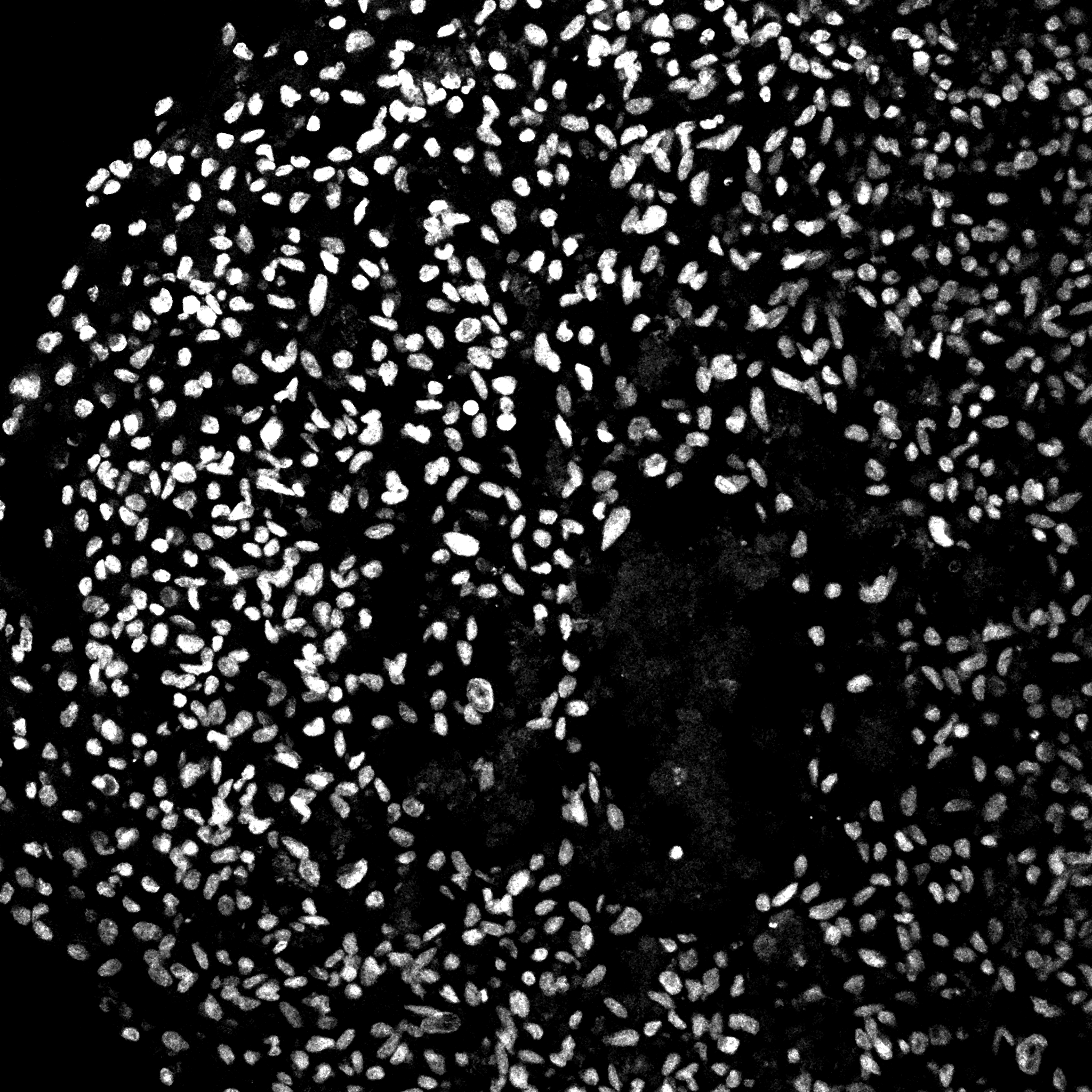

Supplement: Supplementary file 11 — Source Data for Figure 6 [file EMMM-15-e18199-s003.zip › Figure_6/6D/Treatment_PDO_T#5_Caspase3_DAPI.tif]

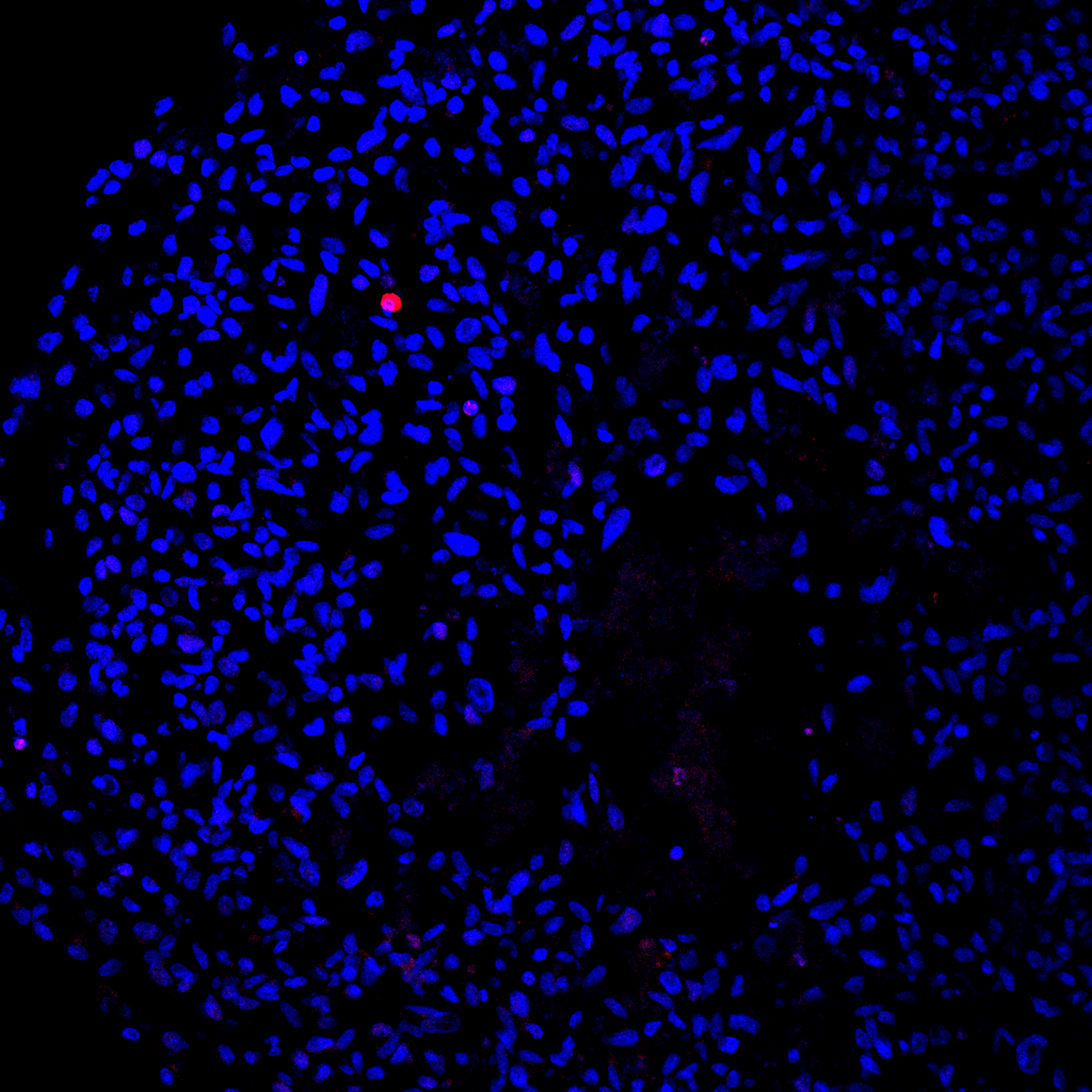

Supplement: Supplementary file 11 — Source Data for Figure 6 [file EMMM-15-e18199-s003.zip › Figure_6/6D/Treatment_PDO_T#5_Caspase3_merge.tif]

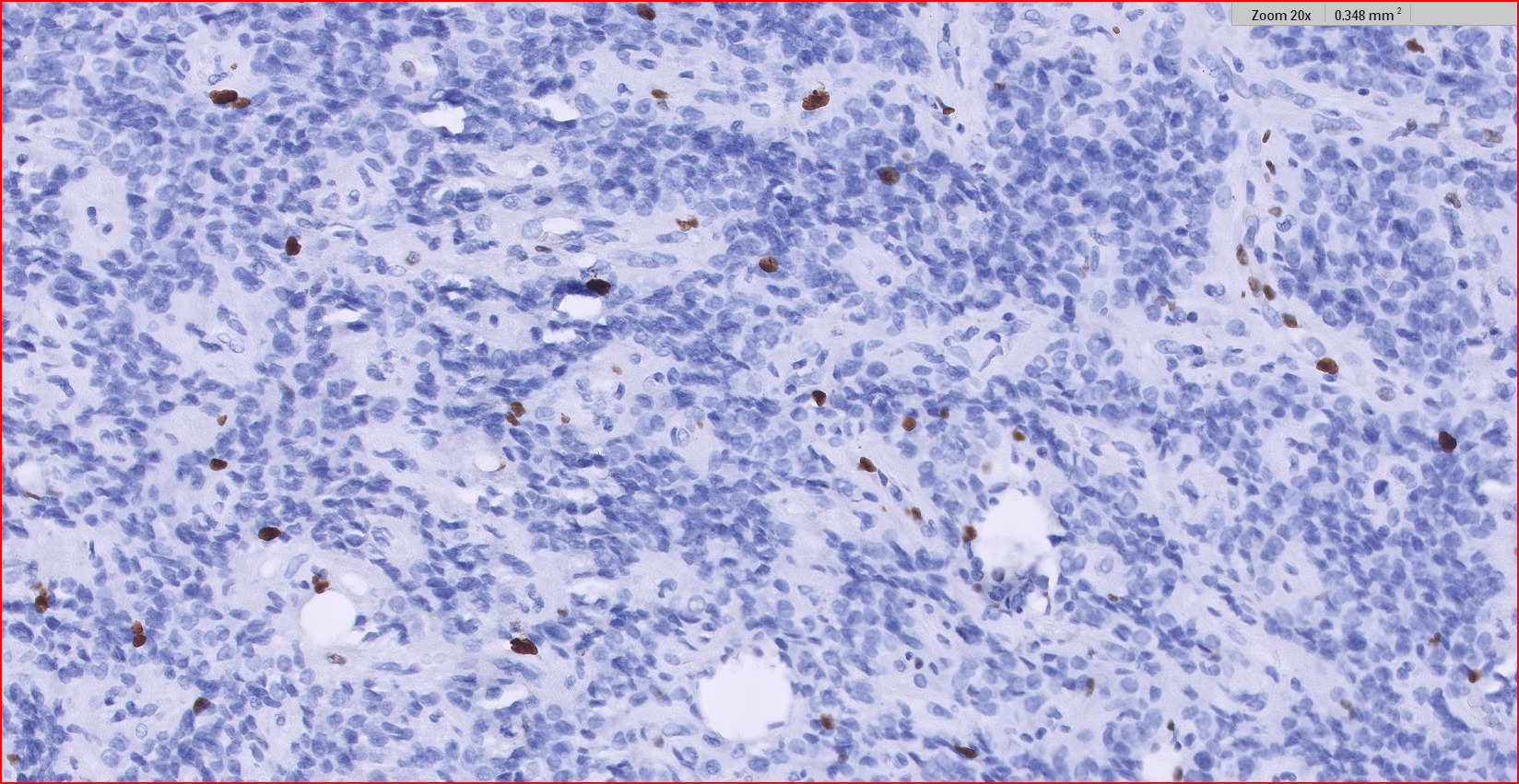

Supplement: Supplementary file 11 — Source Data for Figure 6 [file EMMM-15-e18199-s003.zip › Figure_6/6E/E'''_Tumor_#5_Ki67_post-chemotherapy.jpg]

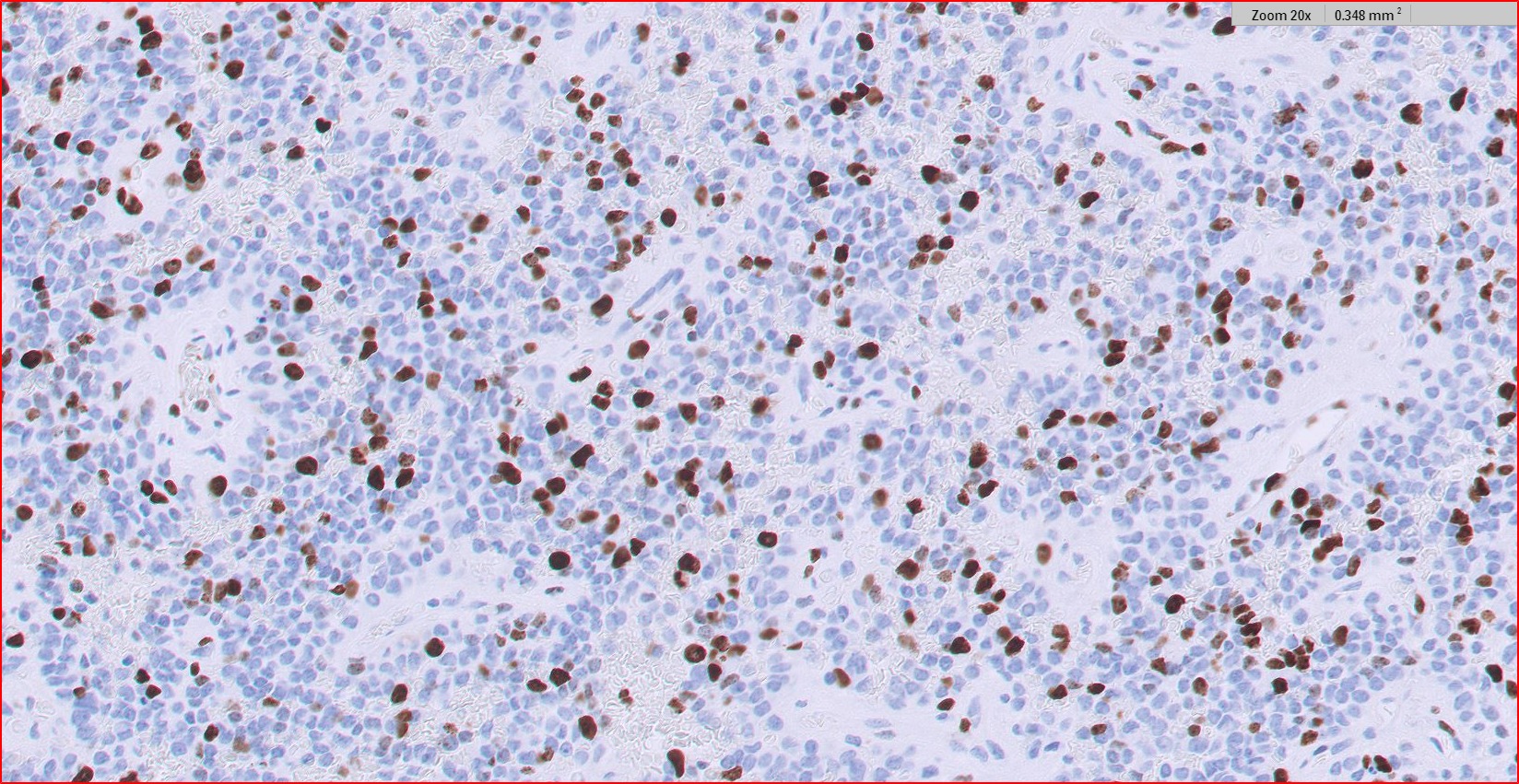

Supplement: Supplementary file 11 — Source Data for Figure 6 [file EMMM-15-e18199-s003.zip › Figure_6/6E/E'''_Tumor_#5_Ki67_post-surgery.jpg]

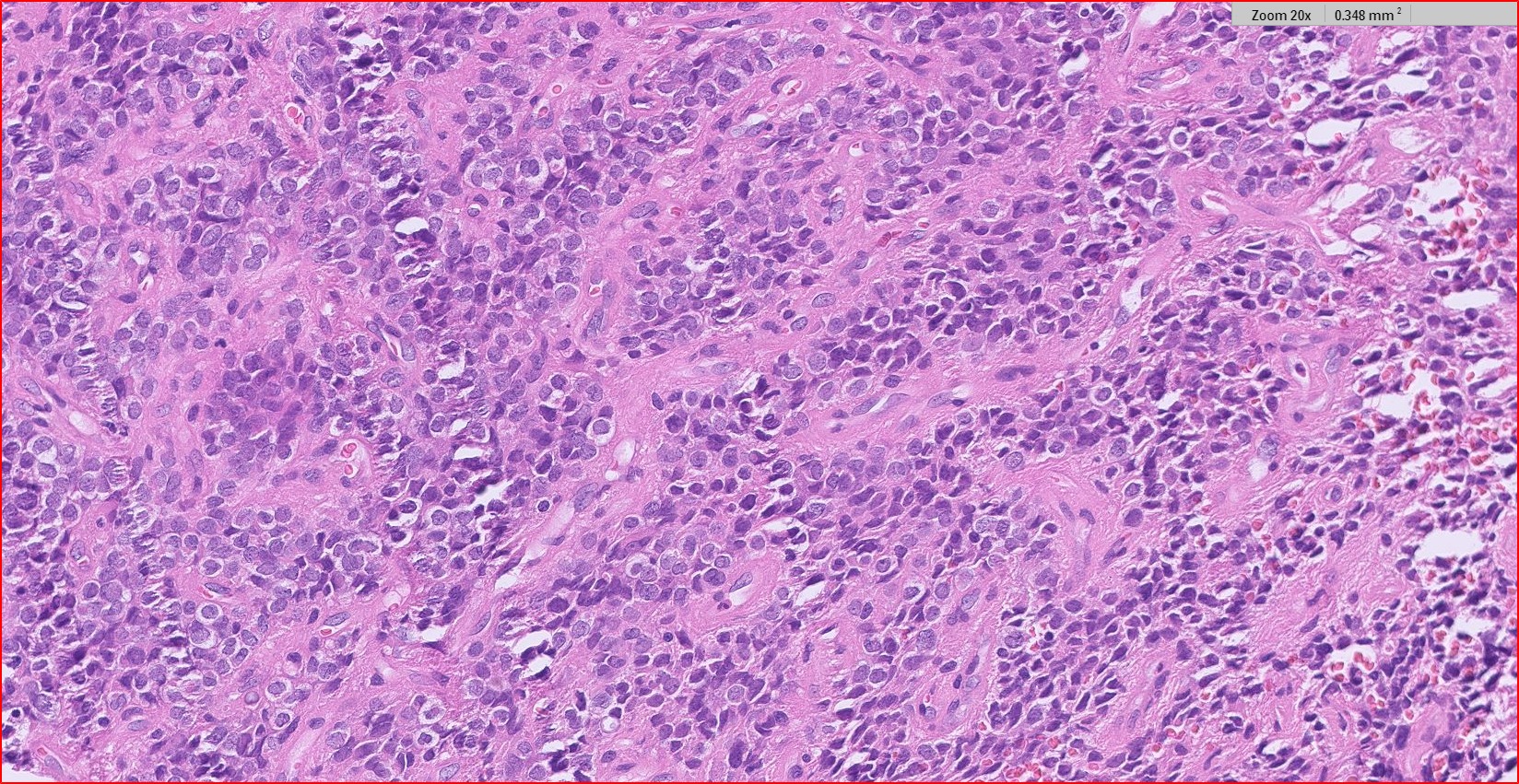

Supplement: Supplementary file 11 — Source Data for Figure 6 [file EMMM-15-e18199-s003.zip › Figure_6/6E/E''_Tumor_#5_H&E_post-chemotherapy.jpg]

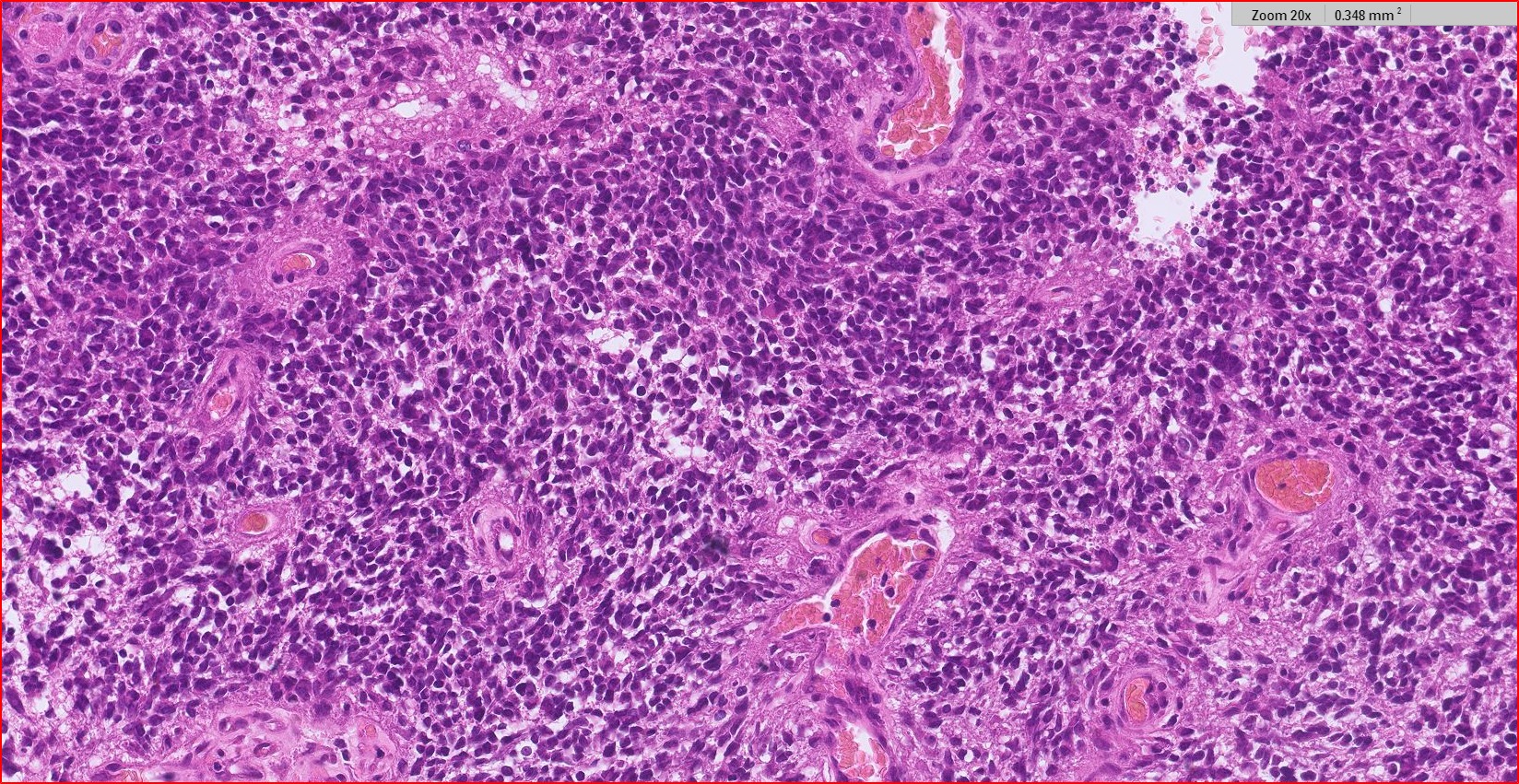

Supplement: Supplementary file 11 — Source Data for Figure 6 [file EMMM-15-e18199-s003.zip › Figure_6/6E/E''_Tumor_#5_H&E_post-surgery.jpg]

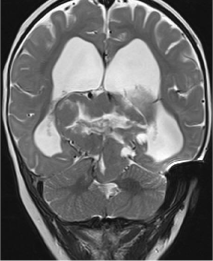

Supplement: Supplementary file 11 — Source Data for Figure 6 [file EMMM-15-e18199-s003.zip › Figure_6/6E/E'_Tumor_#5_MRI_coronal_plane_post-chemotherapy.tif]

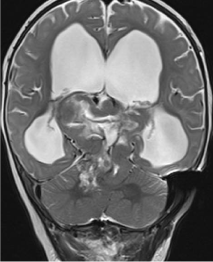

Supplement: Supplementary file 11 — Source Data for Figure 6 [file EMMM-15-e18199-s003.zip › Figure_6/6E/E'_Tumor_#5_MRI_coronal_plane_post-surgery.tif]

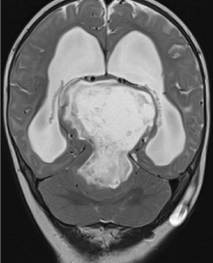

Supplement: Supplementary file 11 — Source Data for Figure 6 [file EMMM-15-e18199-s003.zip › Figure_6/6E/E'_Tumor_#5_MRI_coronal_plane_pre-surgery.tif]

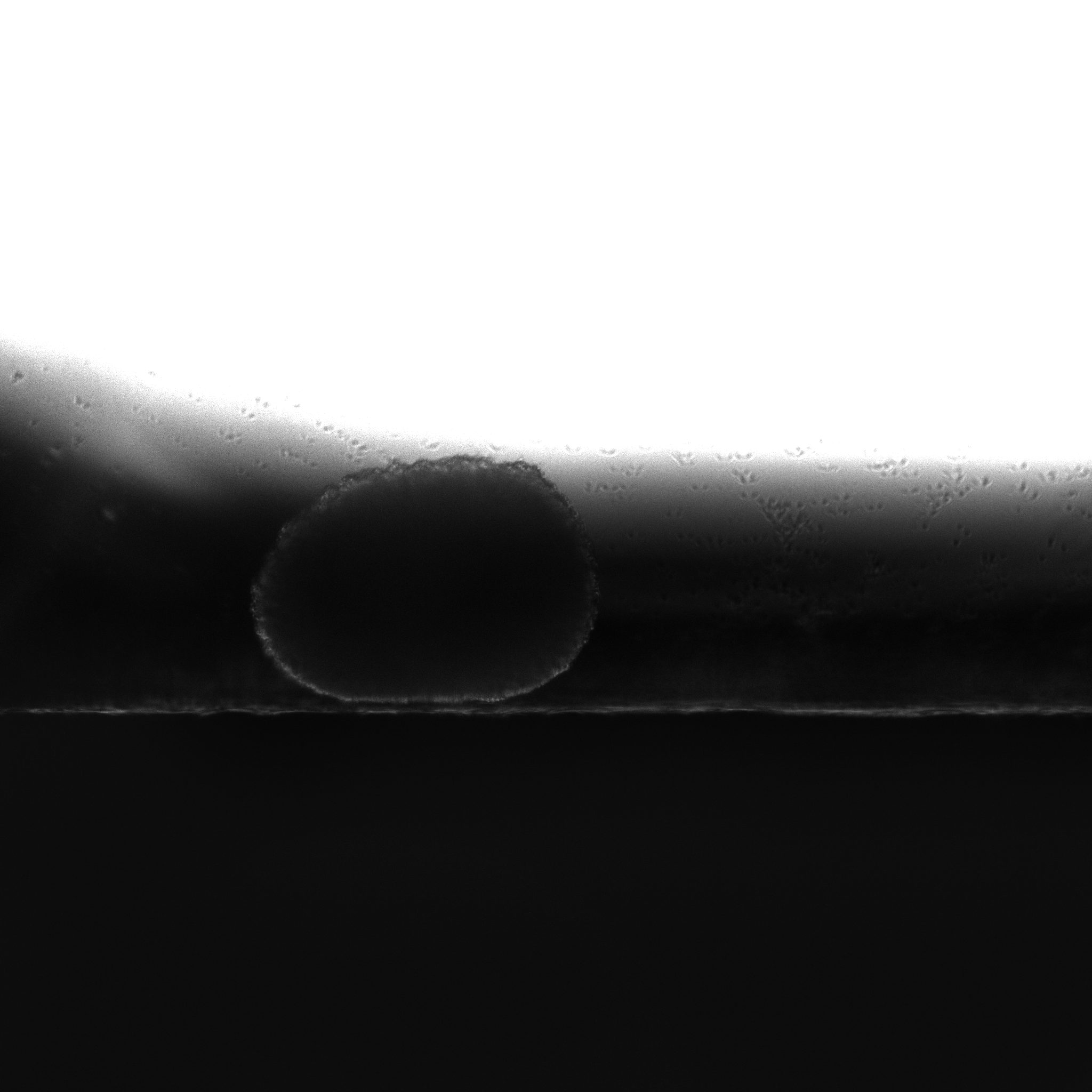

Supplement: Supplementary file 12 — Source Data for Figure 7 [file EMMM-15-e18199-s007.zip › Figure_7/7B/B'_CTRL_Treat._A+B+C_1_month_PDO_T#14_BF.tif]

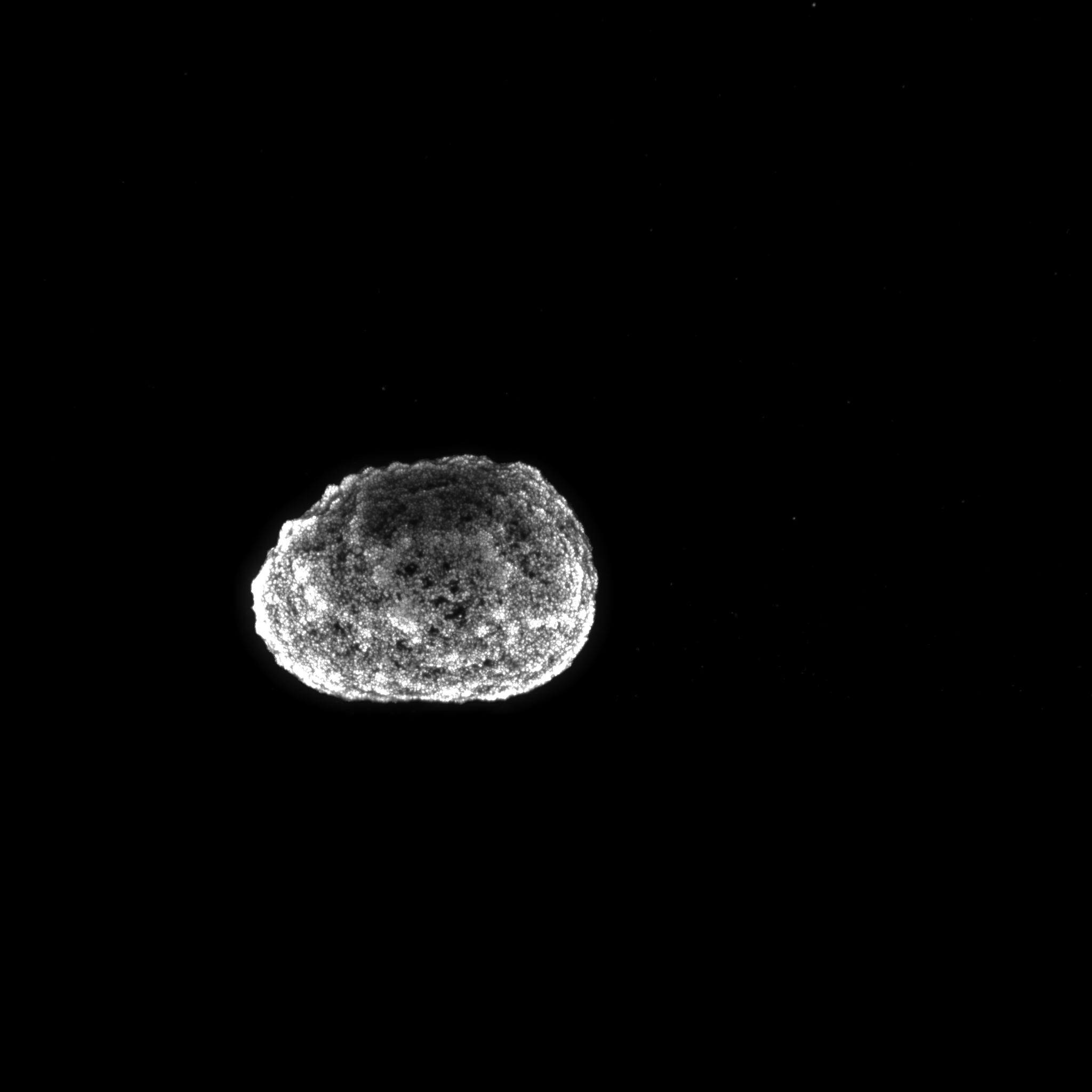

Supplement: Supplementary file 12 — Source Data for Figure 7 [file EMMM-15-e18199-s007.zip › Figure_7/7B/B'_CTRL_Treat._A+B+C_1_month_PDO_T#14_FLUO.tif]

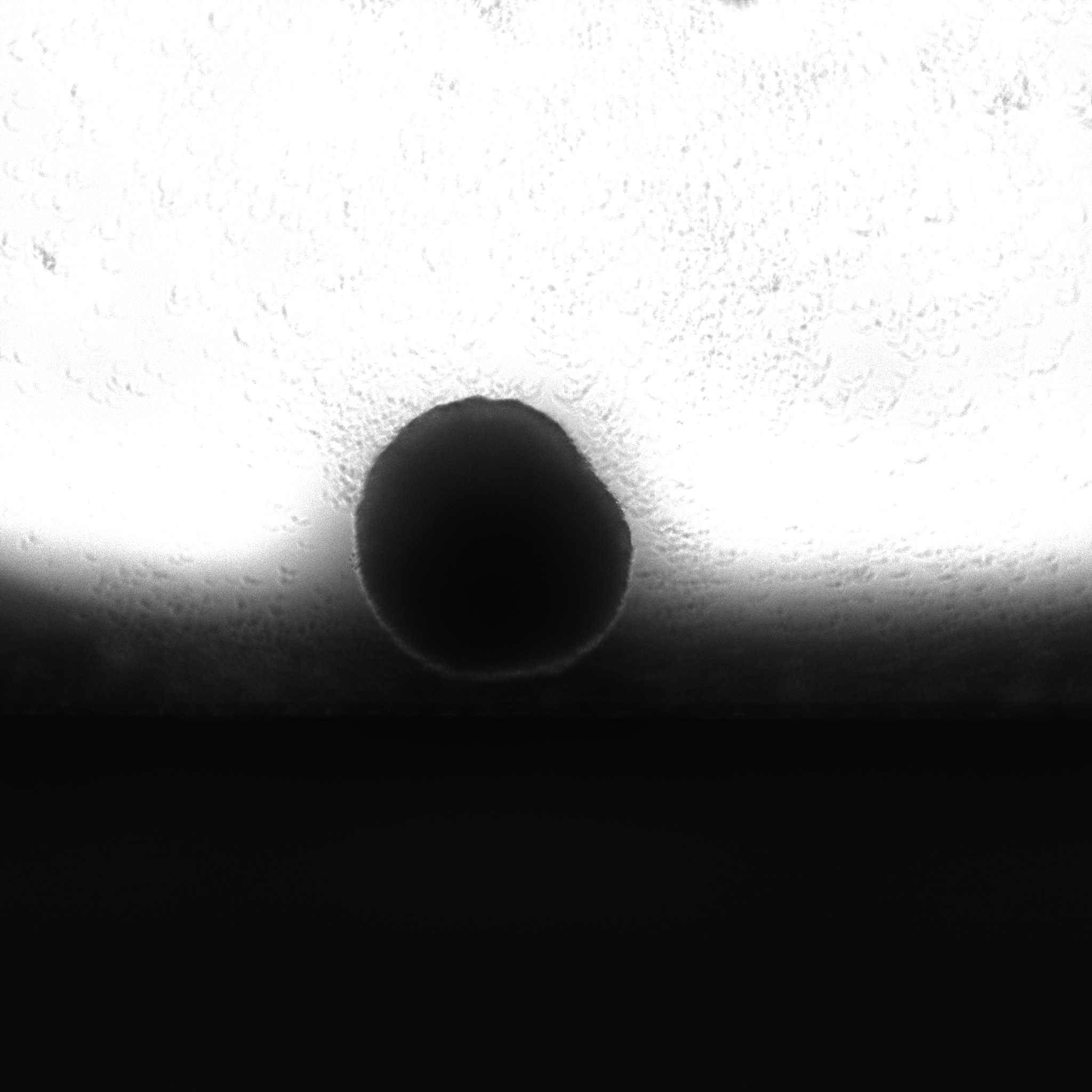

Supplement: Supplementary file 12 — Source Data for Figure 7 [file EMMM-15-e18199-s007.zip › Figure_7/7B/B'_CTRL_Treat._A+B+C_1_week_PDO_T#14_BF.tif]

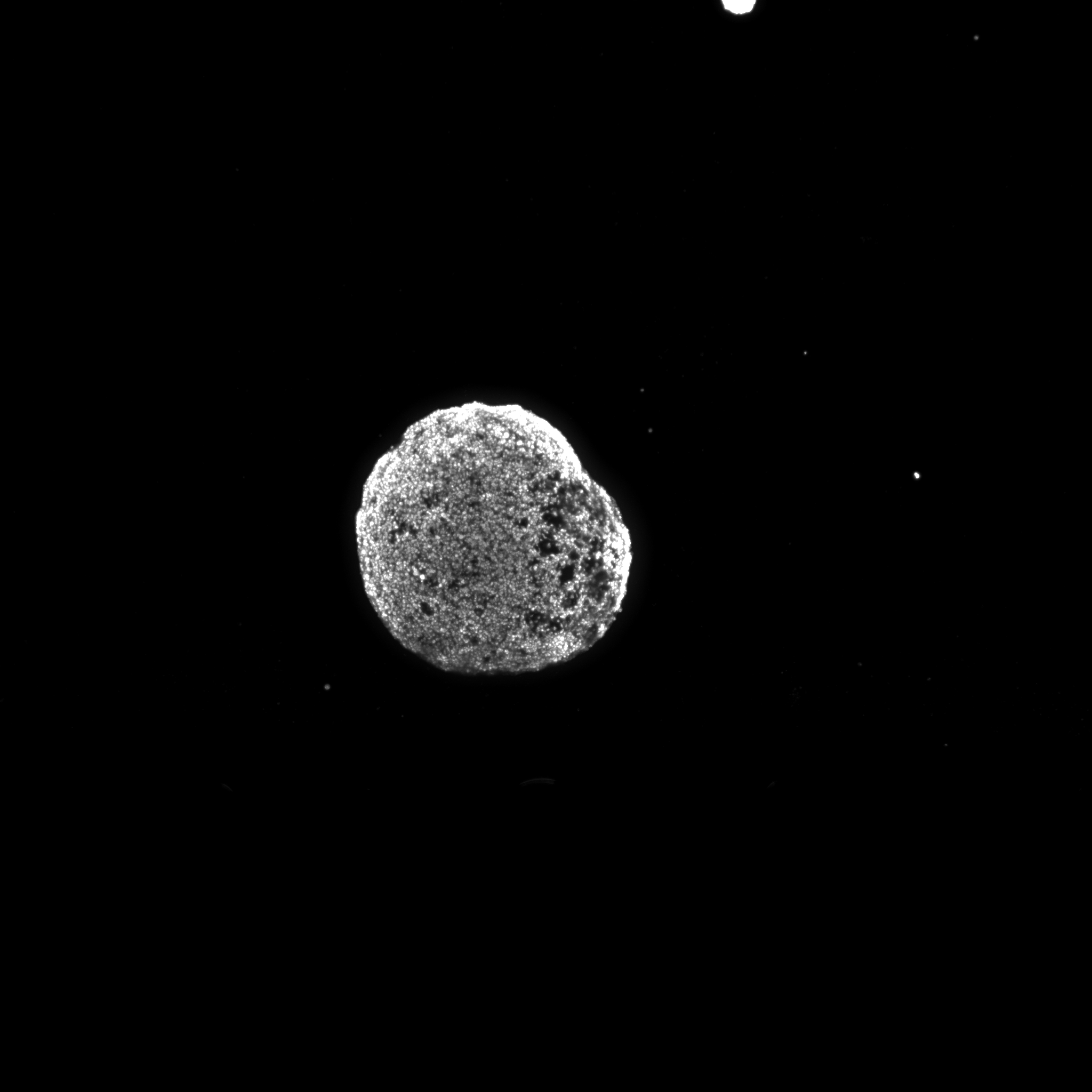

Supplement: Supplementary file 12 — Source Data for Figure 7 [file EMMM-15-e18199-s007.zip › Figure_7/7B/B'_CTRL_Treat._A+B+C_1_week_PDO_T#14_FLUO.tif]

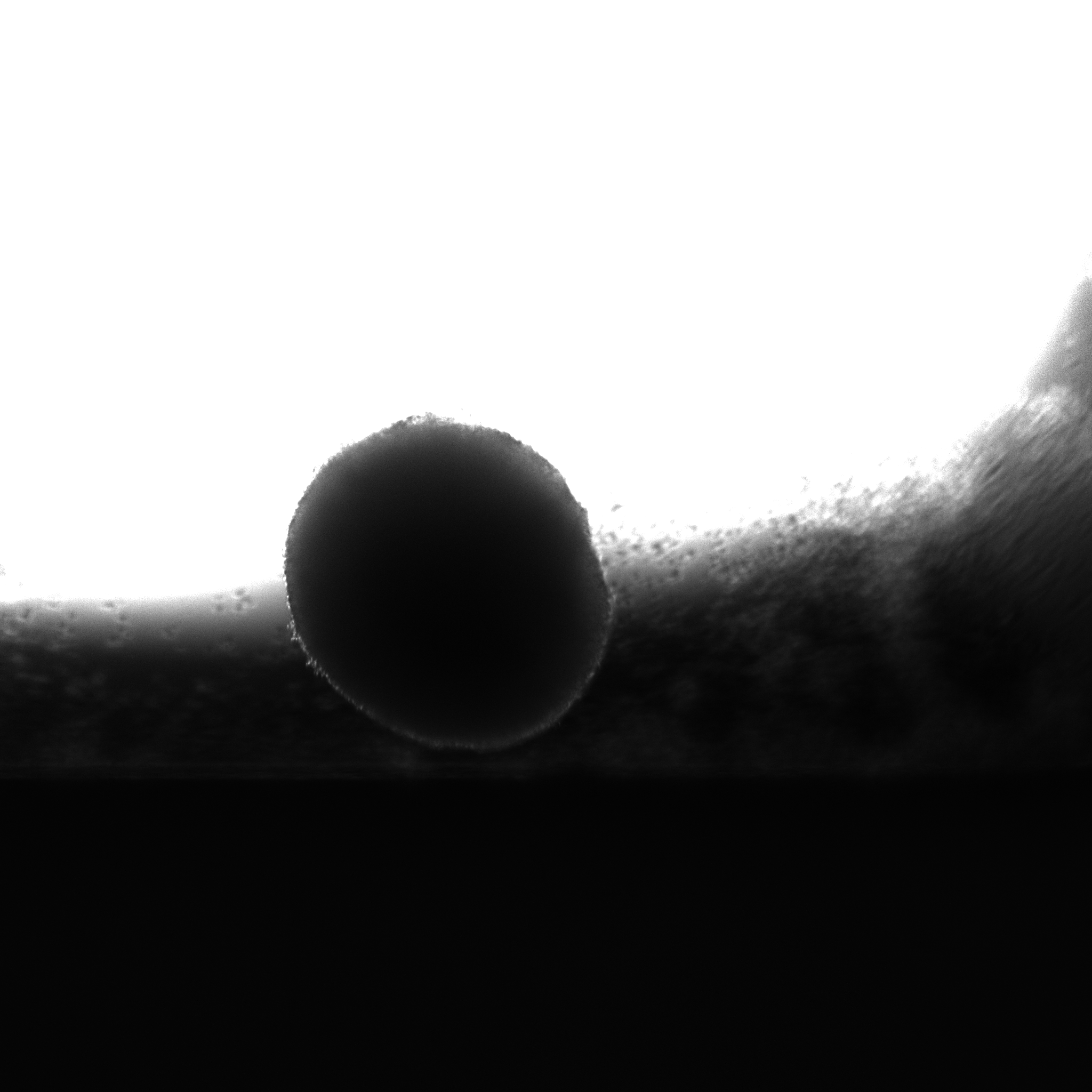

Supplement: Supplementary file 12 — Source Data for Figure 7 [file EMMM-15-e18199-s007.zip › Figure_7/7B/B'_CTRL_Treat._A+B_PDO_T#14_BF.tif]

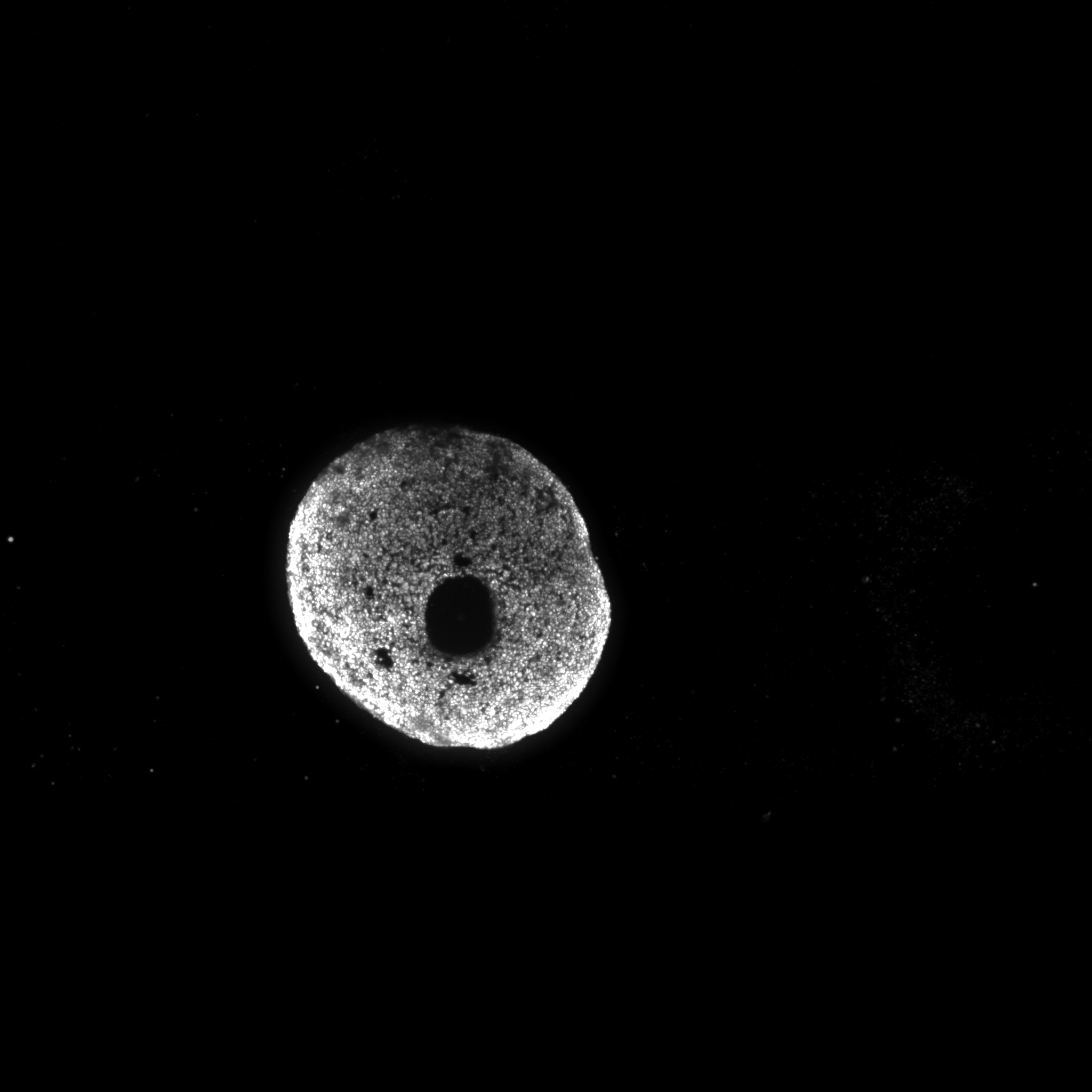

Supplement: Supplementary file 12 — Source Data for Figure 7 [file EMMM-15-e18199-s007.zip › Figure_7/7B/B'_CTRL_Treat._A+B_PDO_T#14_FLUO.tif]

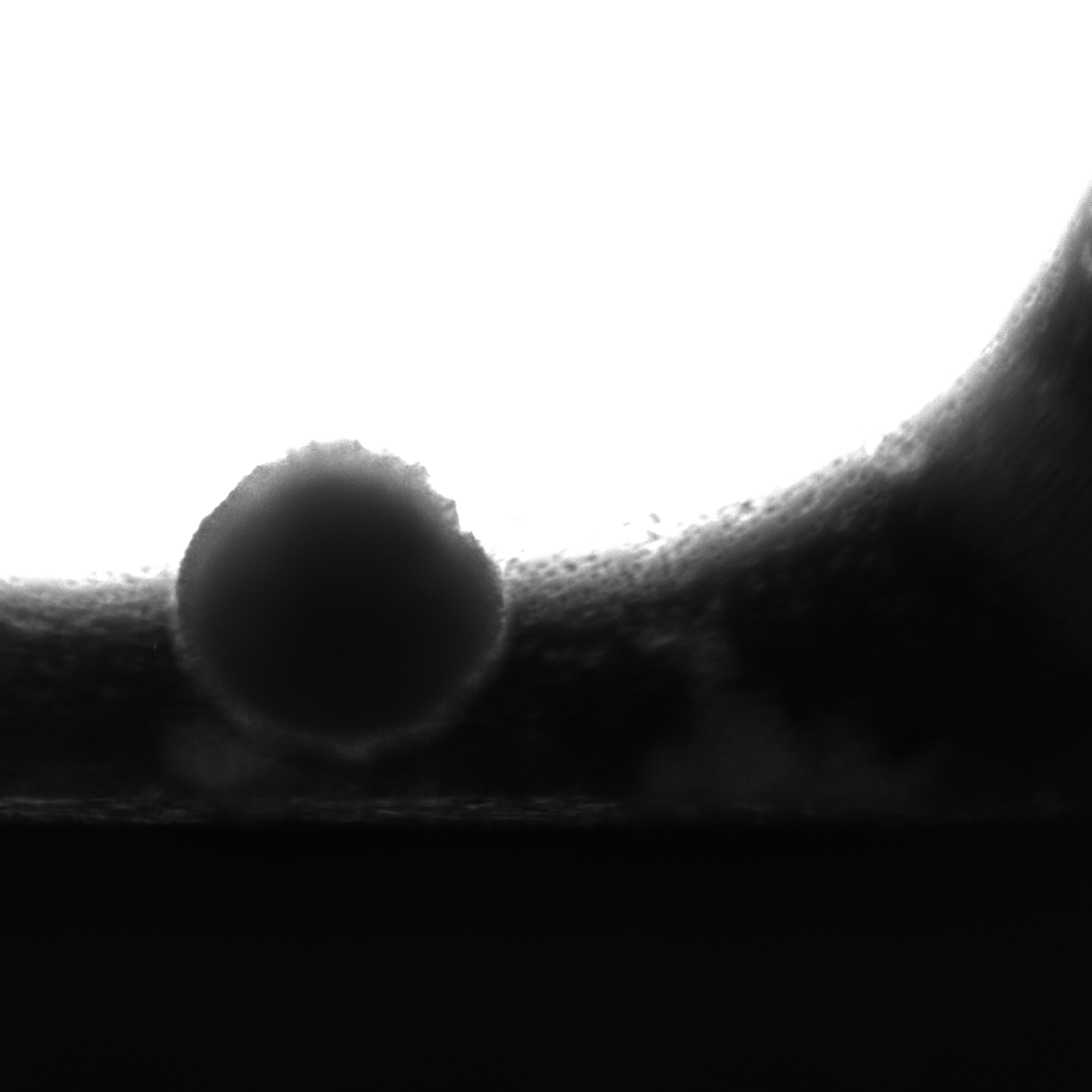

Supplement: Supplementary file 12 — Source Data for Figure 7 [file EMMM-15-e18199-s007.zip › Figure_7/7B/B'_CTRL_Treat._A_PDO_T#14_BF.tif]

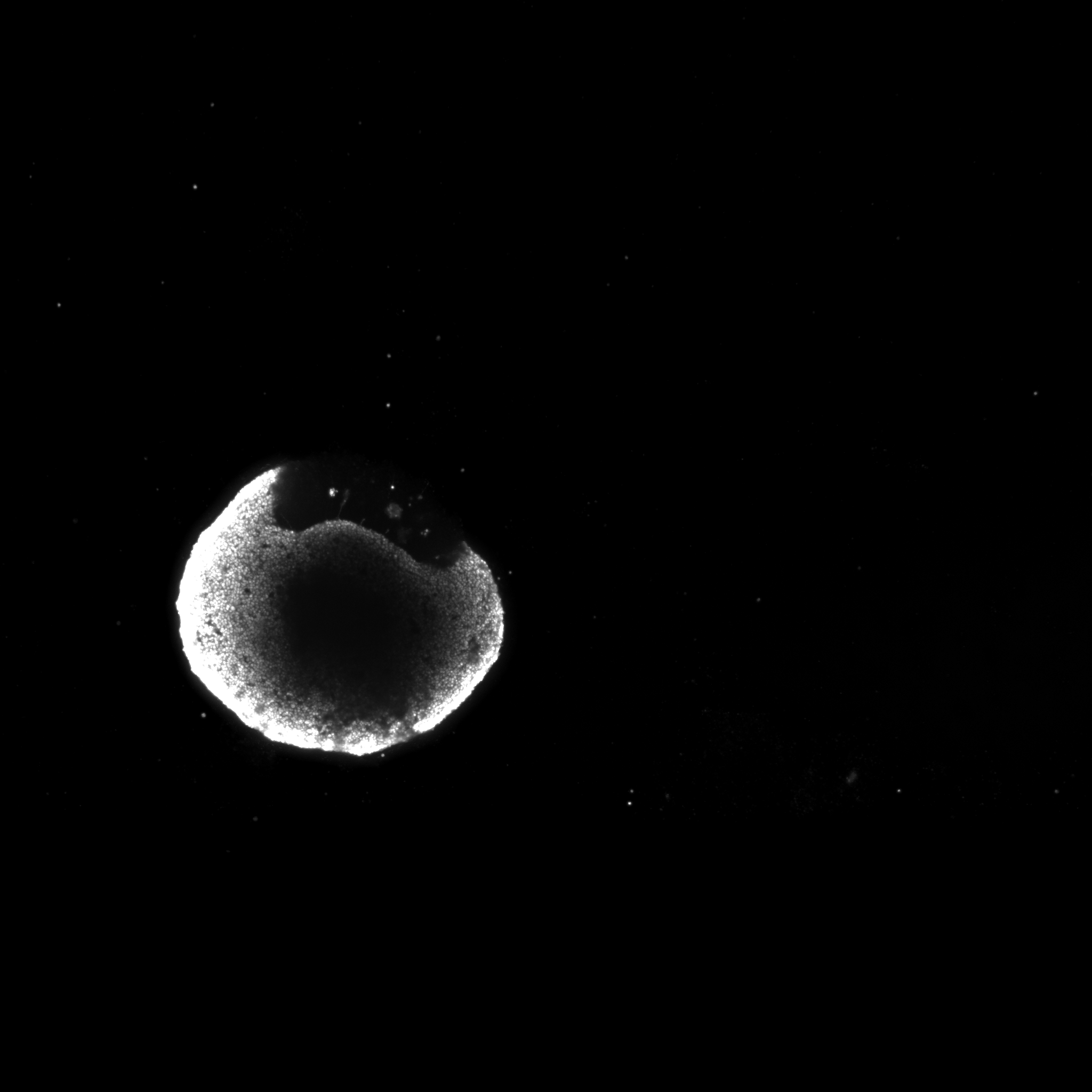

Supplement: Supplementary file 12 — Source Data for Figure 7 [file EMMM-15-e18199-s007.zip › Figure_7/7B/B'_CTRL_Treat._A_PDO_T#14_FLUO.tif]

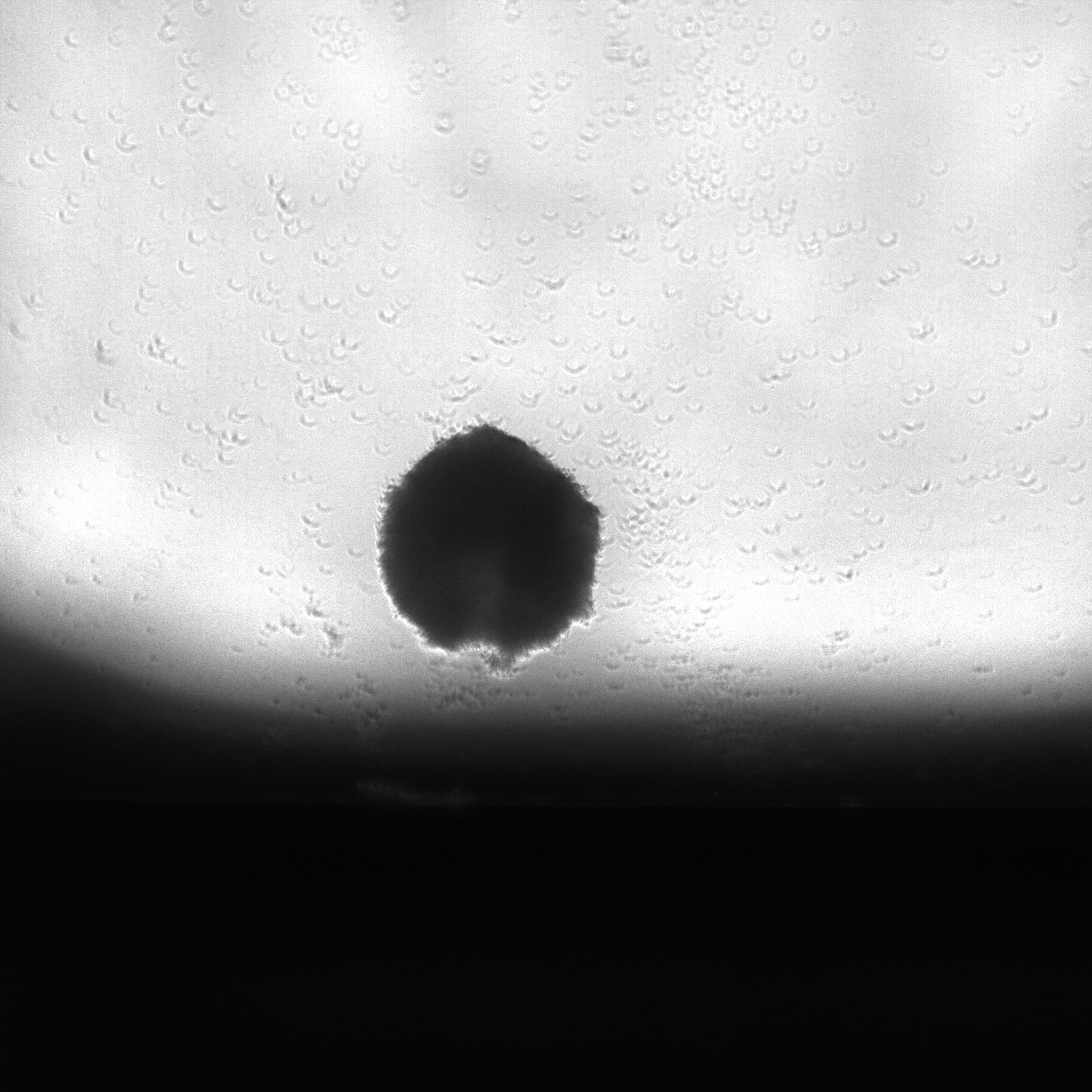

Supplement: Supplementary file 12 — Source Data for Figure 7 [file EMMM-15-e18199-s007.zip › Figure_7/7B/B'_Treat._A+B+C_1_month_PDO_T#14_BF.tif]

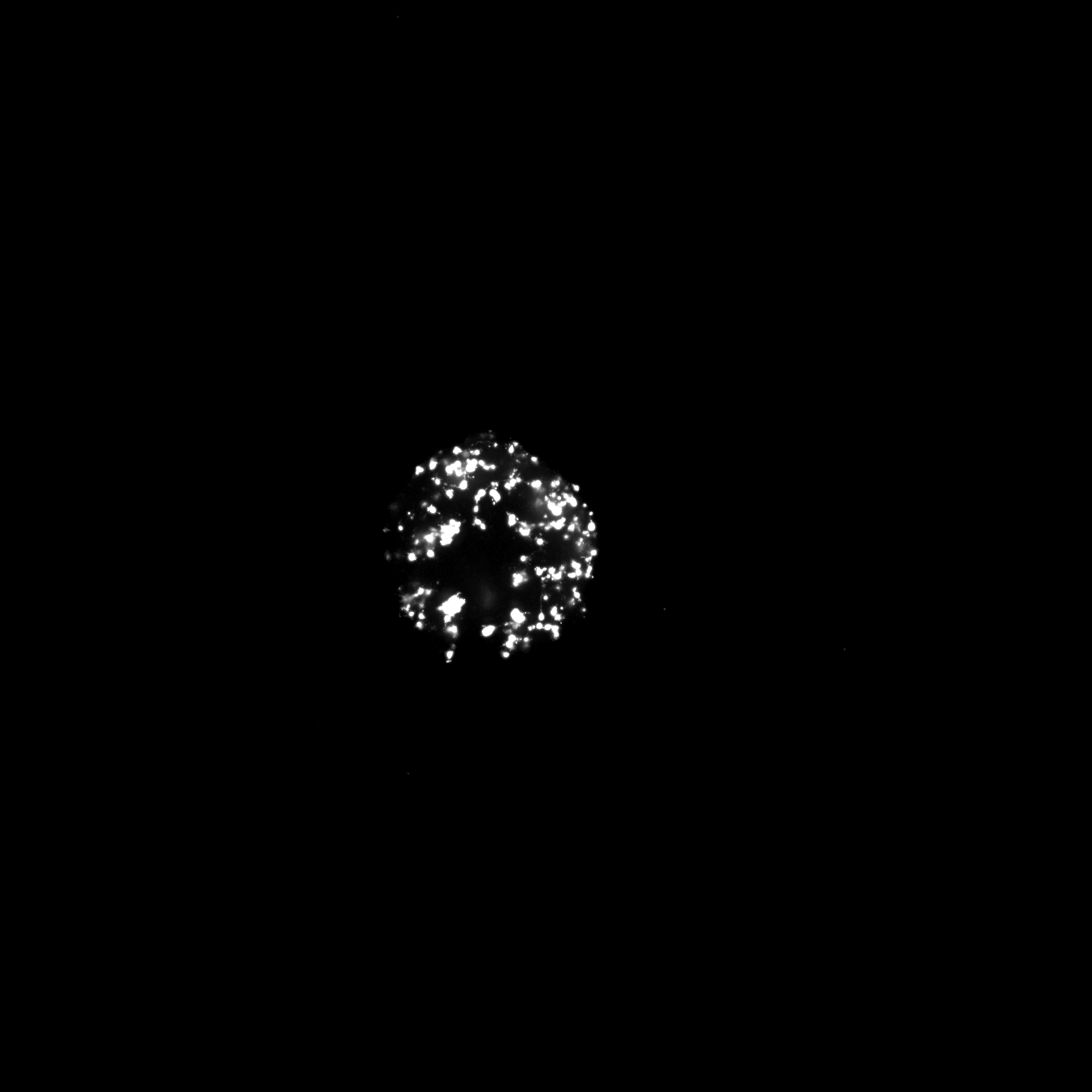

Supplement: Supplementary file 12 — Source Data for Figure 7 [file EMMM-15-e18199-s007.zip › Figure_7/7B/B'_Treat._A+B+C_1_month_PDO_T#14_FLUO.tif]

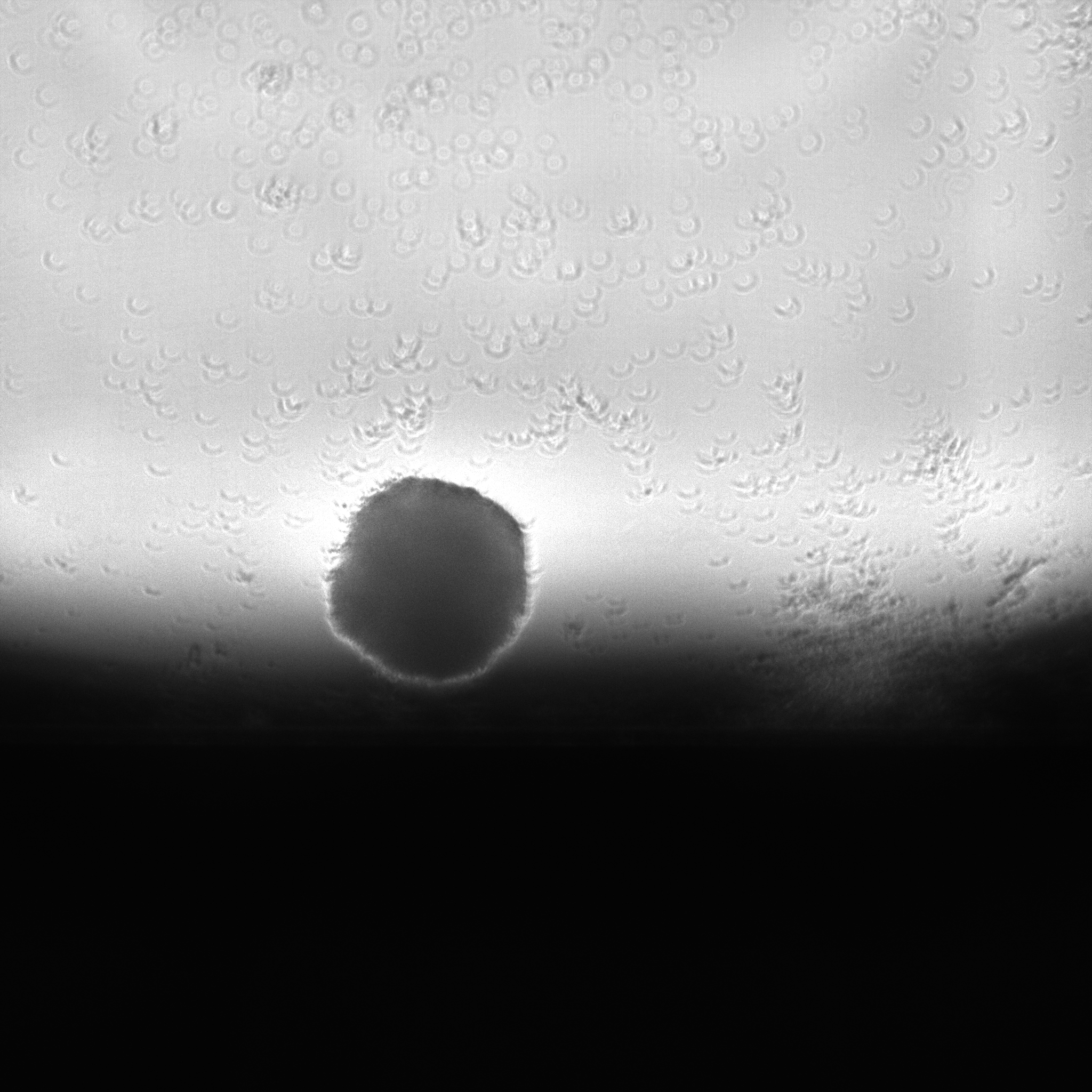

Supplement: Supplementary file 12 — Source Data for Figure 7 [file EMMM-15-e18199-s007.zip › Figure_7/7B/B'_Treat._A+B+C_1_week_PDO_T#14_BF.tif]

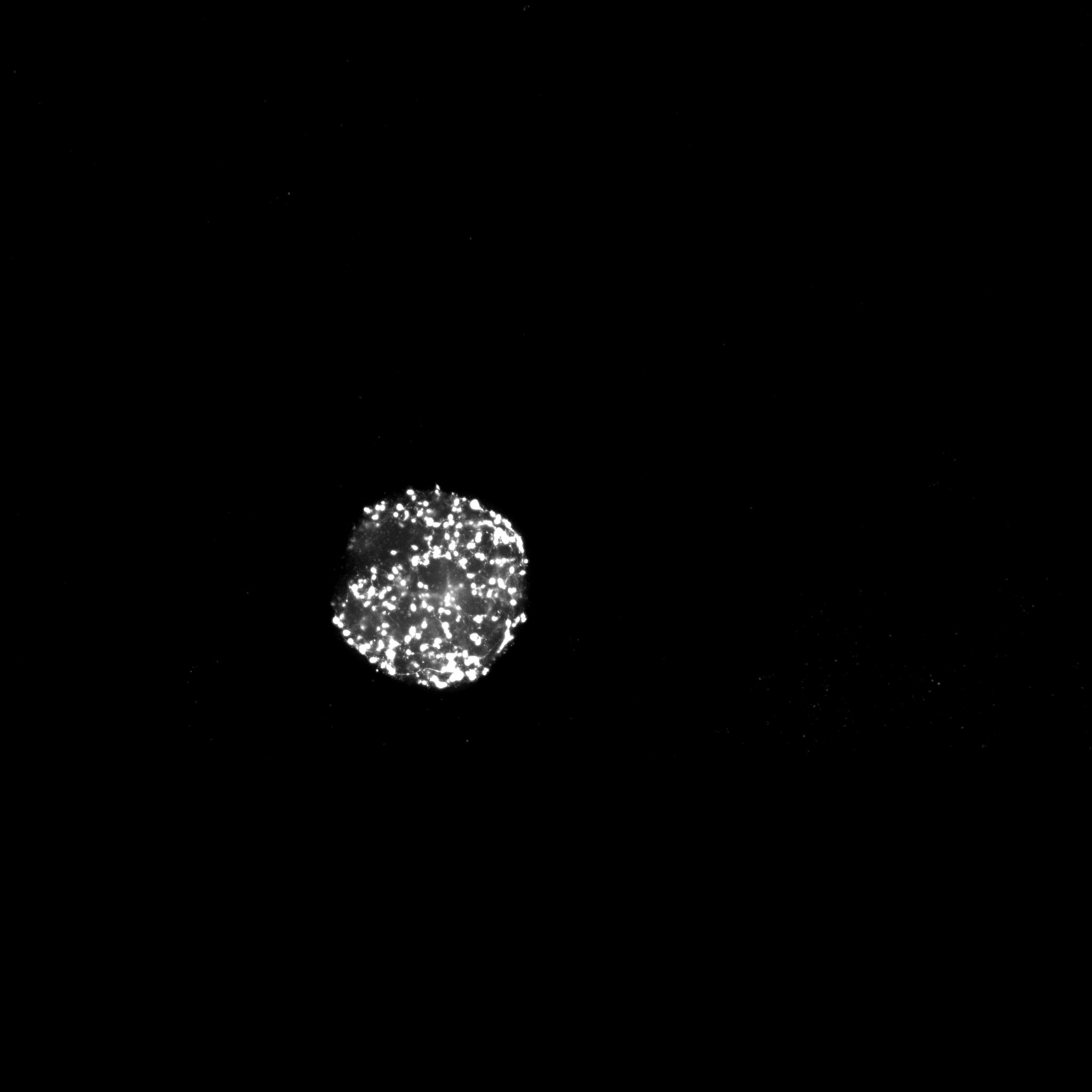

Supplement: Supplementary file 12 — Source Data for Figure 7 [file EMMM-15-e18199-s007.zip › Figure_7/7B/B'_Treat._A+B+C_1_week_PDO_T#14_FLUO.tif]

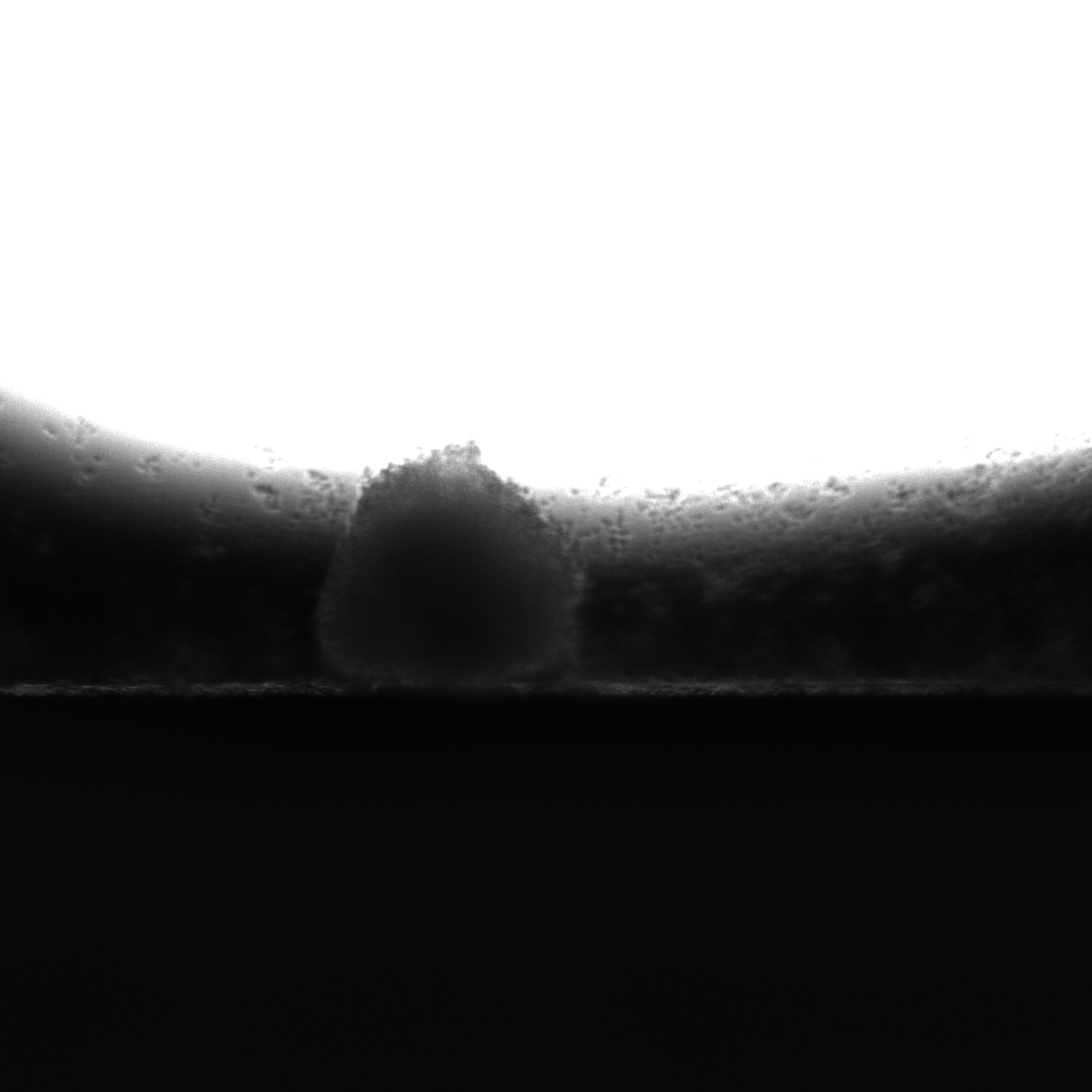

Supplement: Supplementary file 12 — Source Data for Figure 7 [file EMMM-15-e18199-s007.zip › Figure_7/7B/B'_Treat._A+B_PDO_T#14_BF.tif]

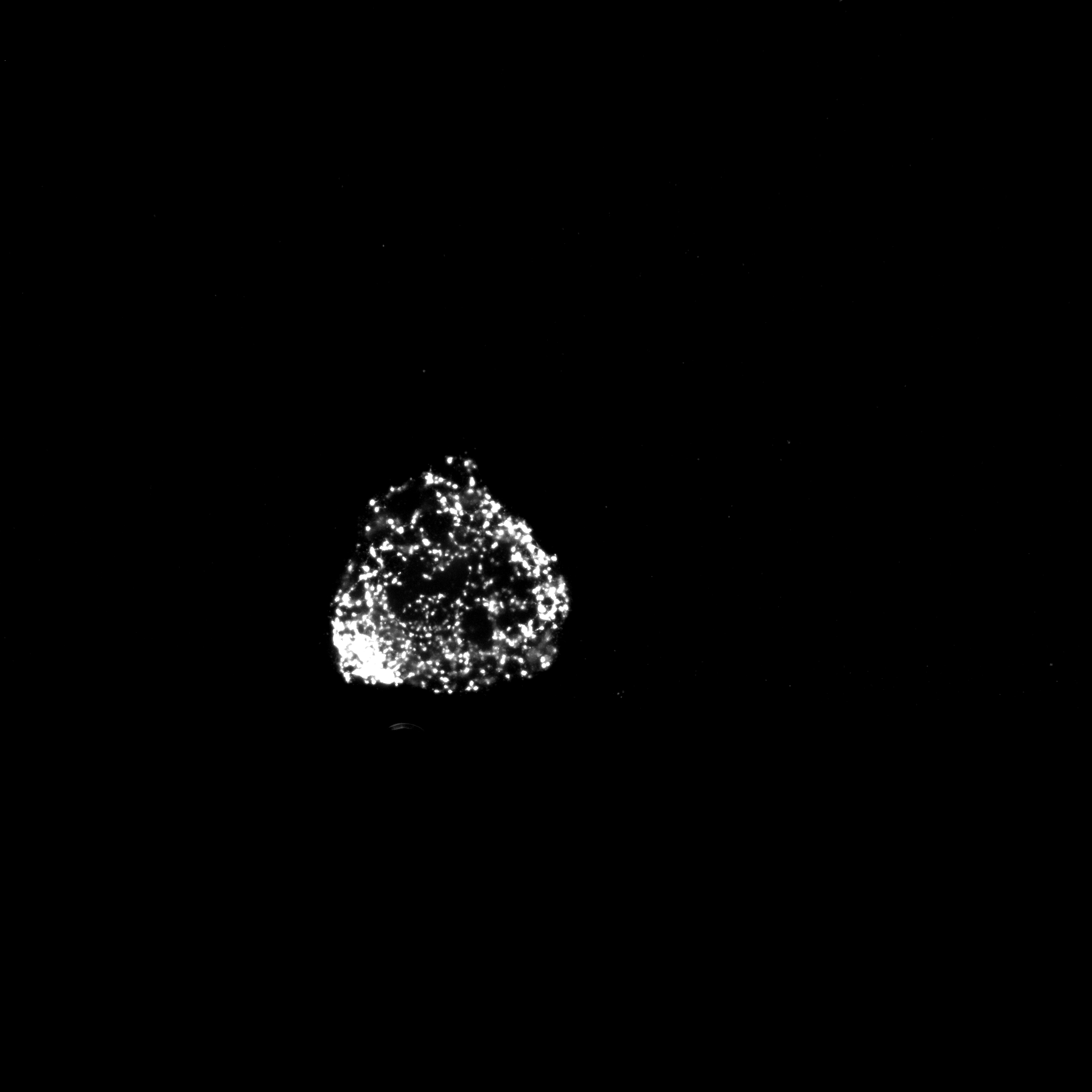

Supplement: Supplementary file 12 — Source Data for Figure 7 [file EMMM-15-e18199-s007.zip › Figure_7/7B/B'_Treat._A+B_PDO_T#14_FLUO.tif]

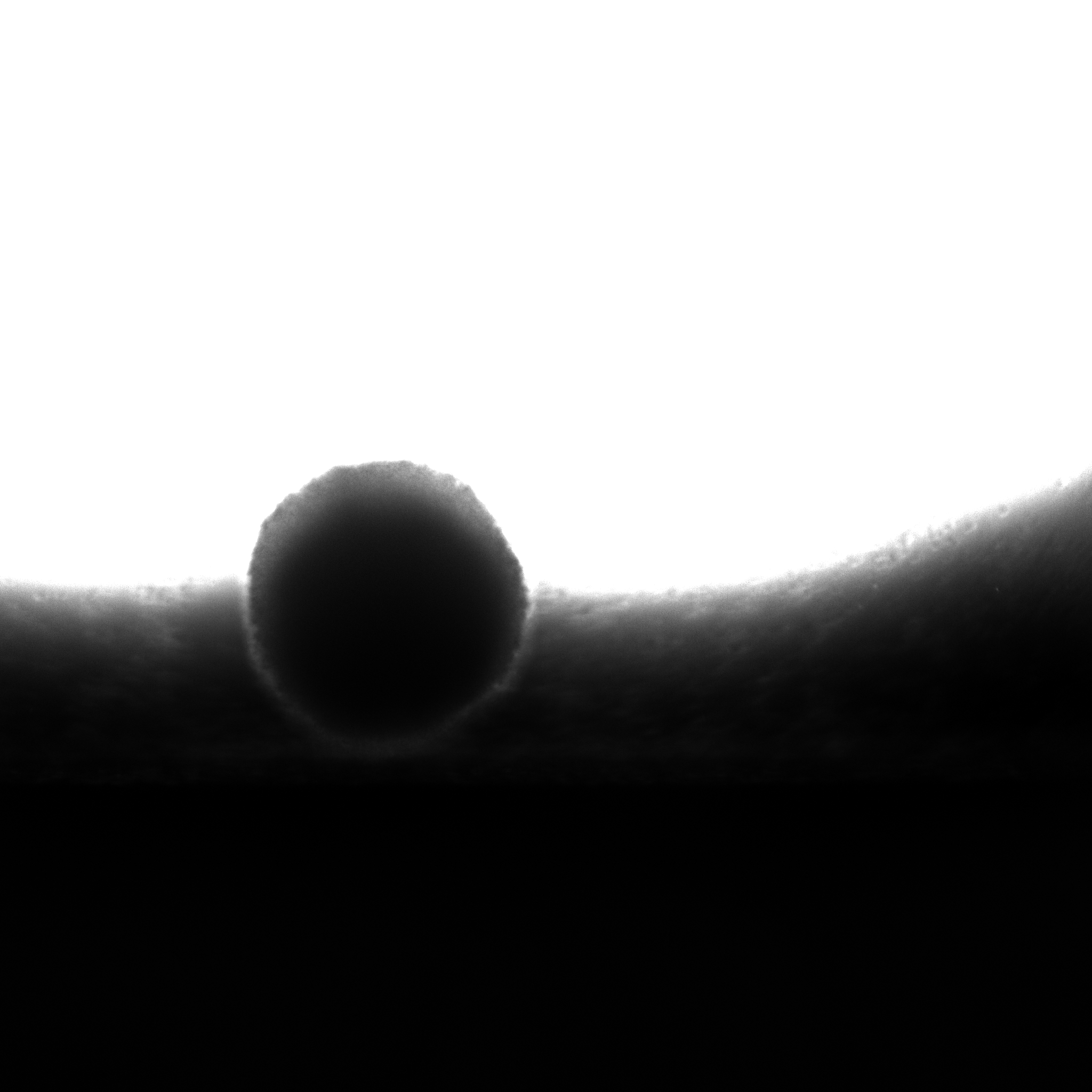

Supplement: Supplementary file 12 — Source Data for Figure 7 [file EMMM-15-e18199-s007.zip › Figure_7/7B/B'_Treat._A_PDO_T#14_BF.tif]

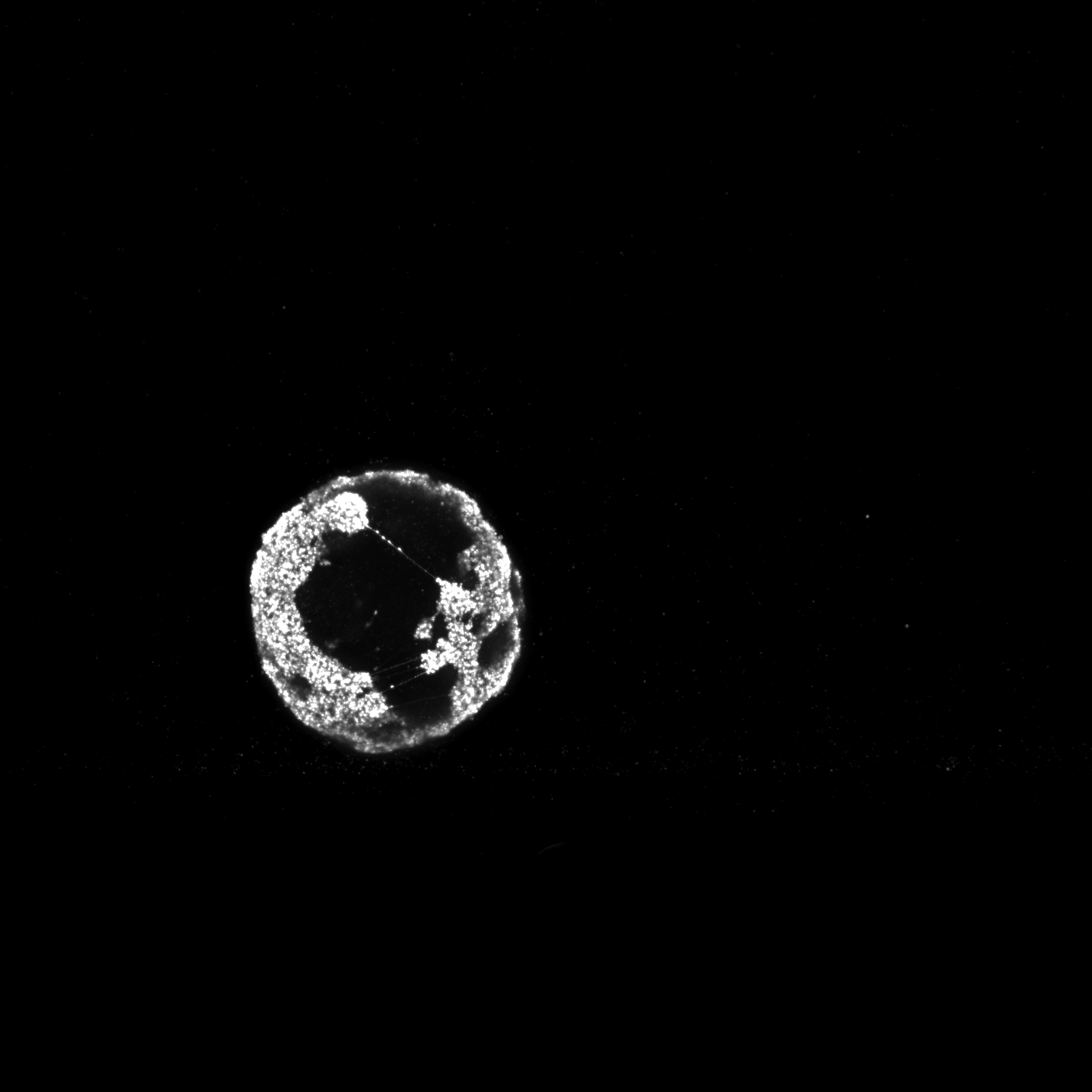

Supplement: Supplementary file 12 — Source Data for Figure 7 [file EMMM-15-e18199-s007.zip › Figure_7/7B/B'_Treat._A_PDO_T#14_FLUO.tif]

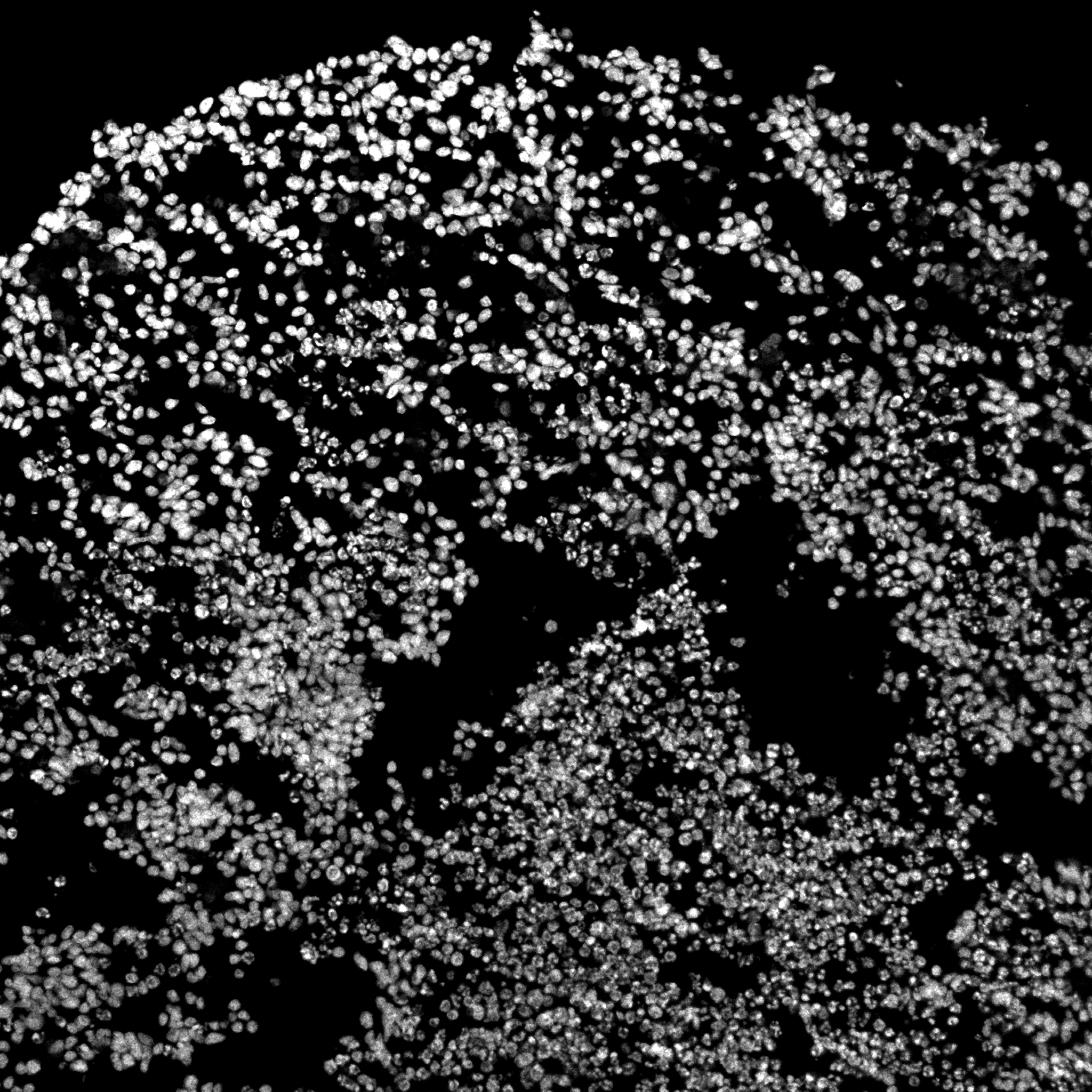

Supplement: Supplementary file 12 — Source Data for Figure 7 [file EMMM-15-e18199-s007.zip › Figure_7/7C/CTRL_Treat._A+B+C_1_month_PDO_T#14_Ki67_DAPI.tif]

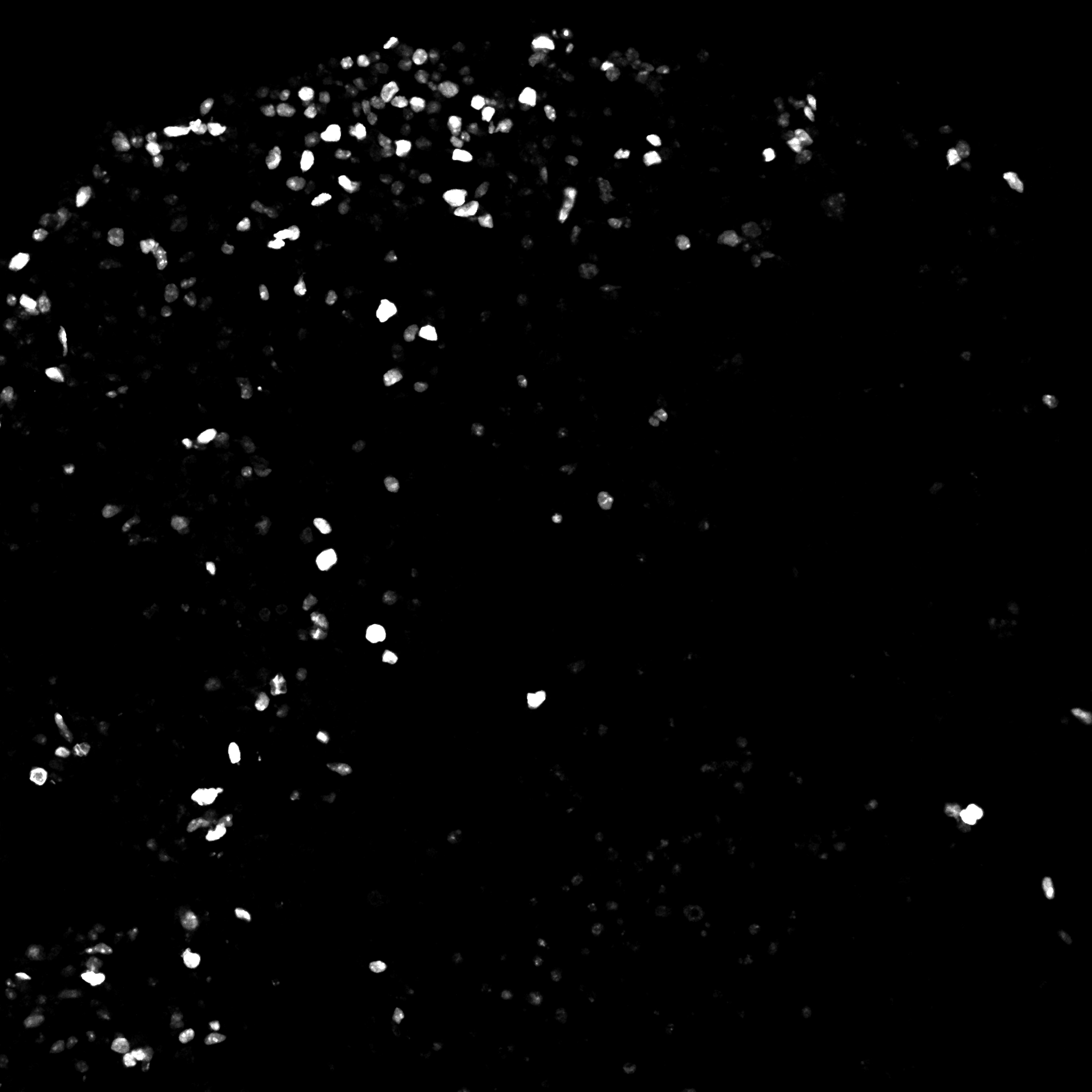

Supplement: Supplementary file 12 — Source Data for Figure 7 [file EMMM-15-e18199-s007.zip › Figure_7/7C/CTRL_Treat._A+B+C_1_month_PDO_T#14_Ki67_Ki67.tif]

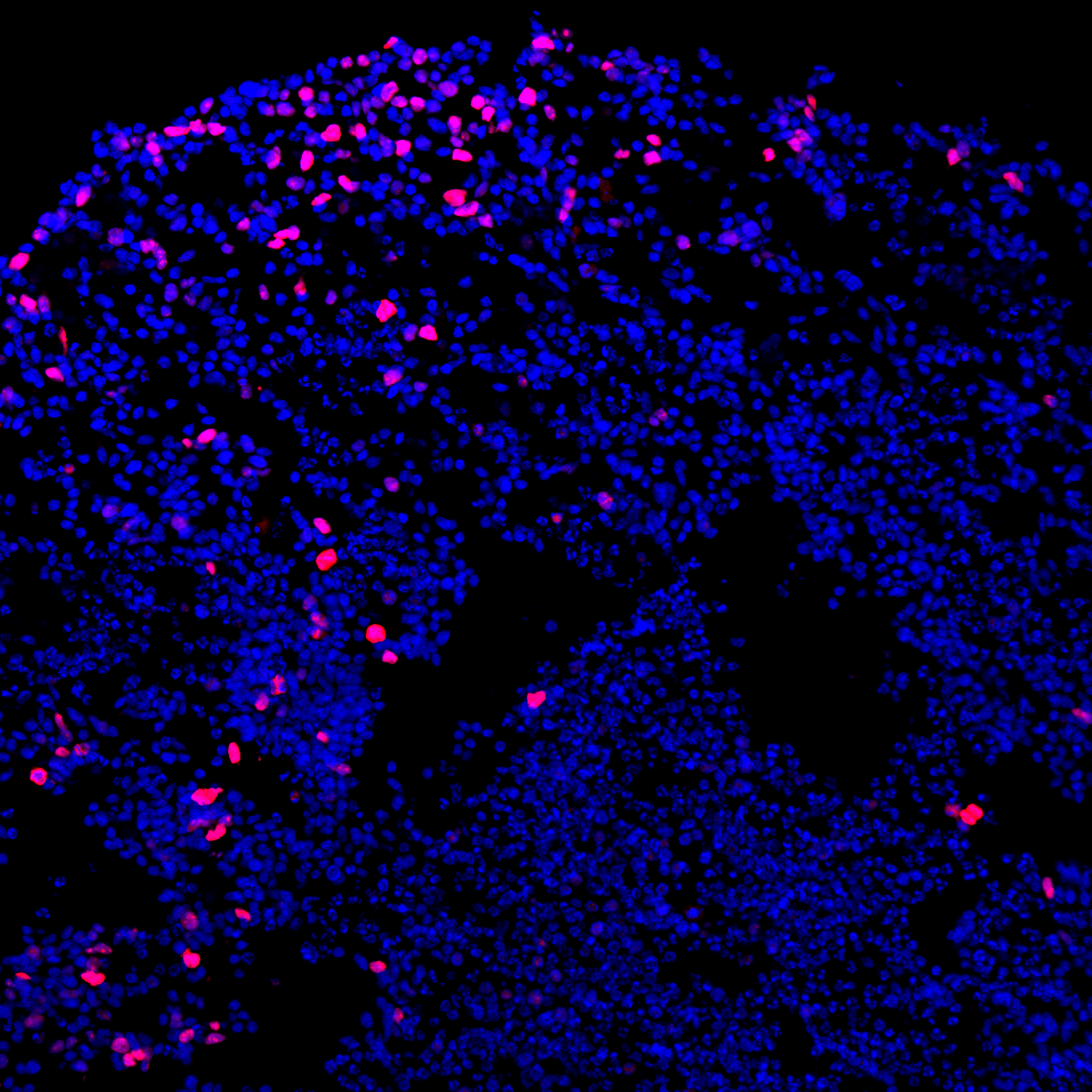

Supplement: Supplementary file 12 — Source Data for Figure 7 [file EMMM-15-e18199-s007.zip › Figure_7/7C/CTRL_Treat._A+B+C_1_month_PDO_T#14_Ki67_merge.tif]

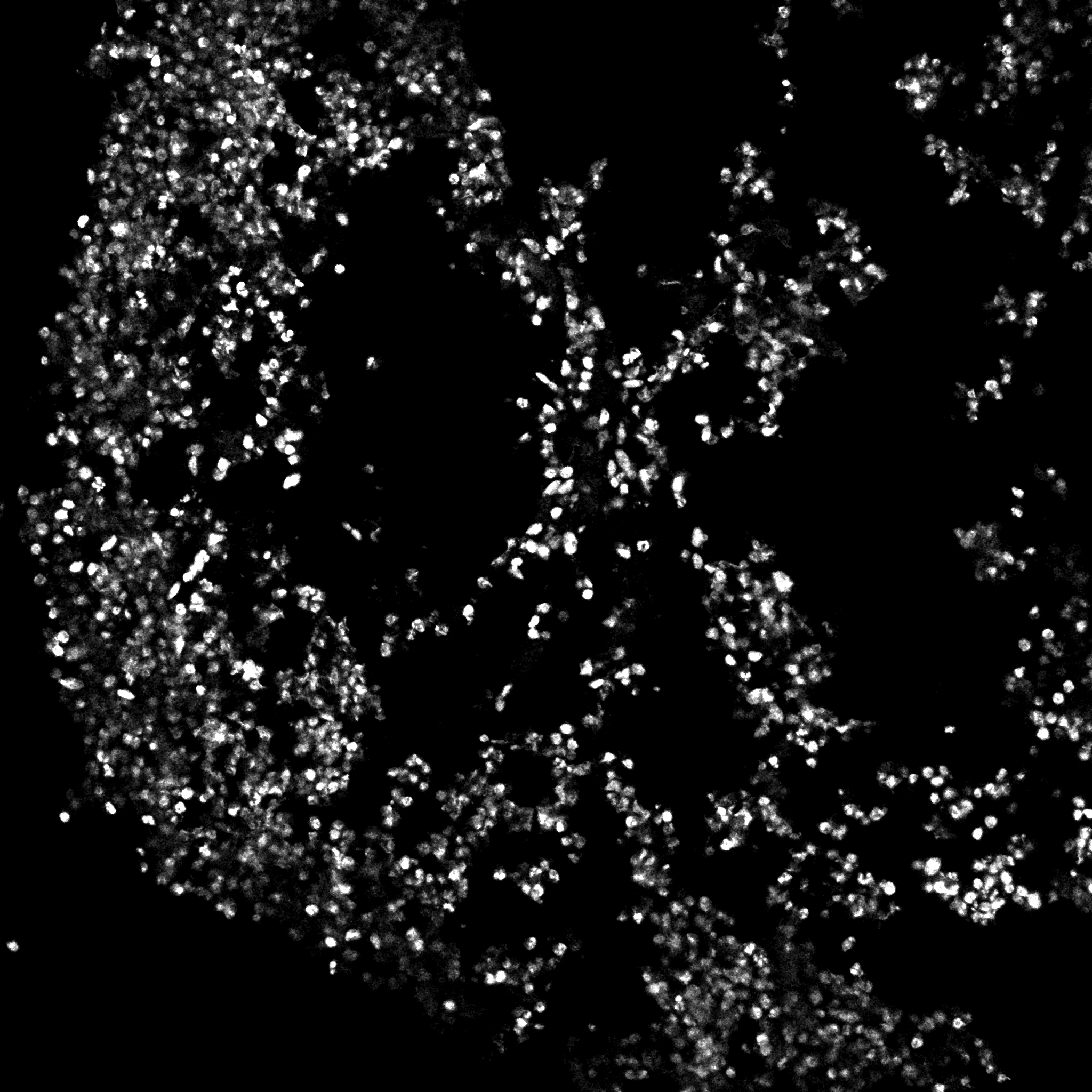

Supplement: Supplementary file 12 — Source Data for Figure 7 [file EMMM-15-e18199-s007.zip › Figure_7/7C/Treat._A+B+C_1_month_PDO_T#14_Ki67_DAPI.tif]

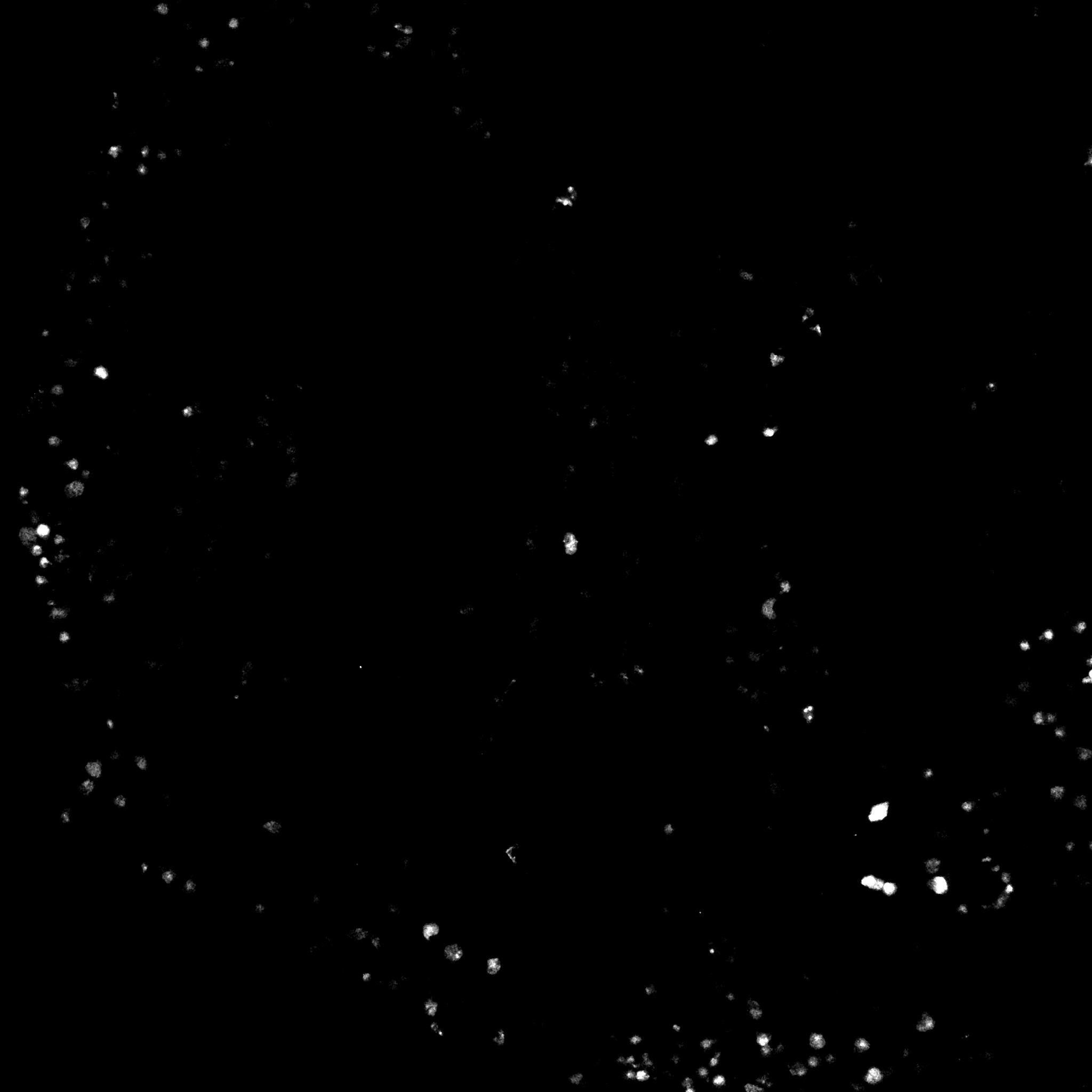

Supplement: Supplementary file 12 — Source Data for Figure 7 [file EMMM-15-e18199-s007.zip › Figure_7/7C/Treat._A+B+C_1_month_PDO_T#14_Ki67_Ki67.tif]

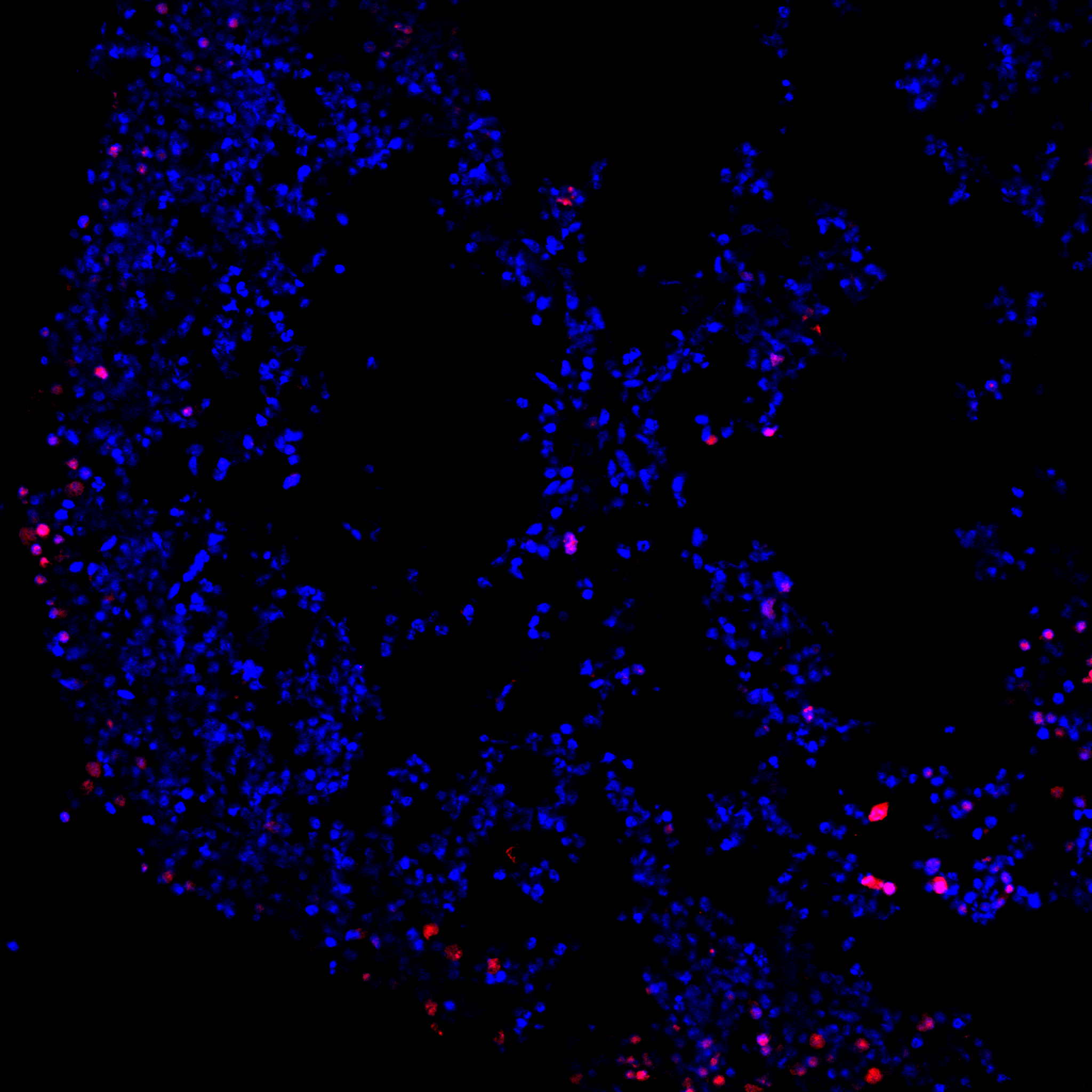

Supplement: Supplementary file 12 — Source Data for Figure 7 [file EMMM-15-e18199-s007.zip › Figure_7/7C/Treat._A+B+C_1_month_PDO_T#14_Ki67_merge.tif]

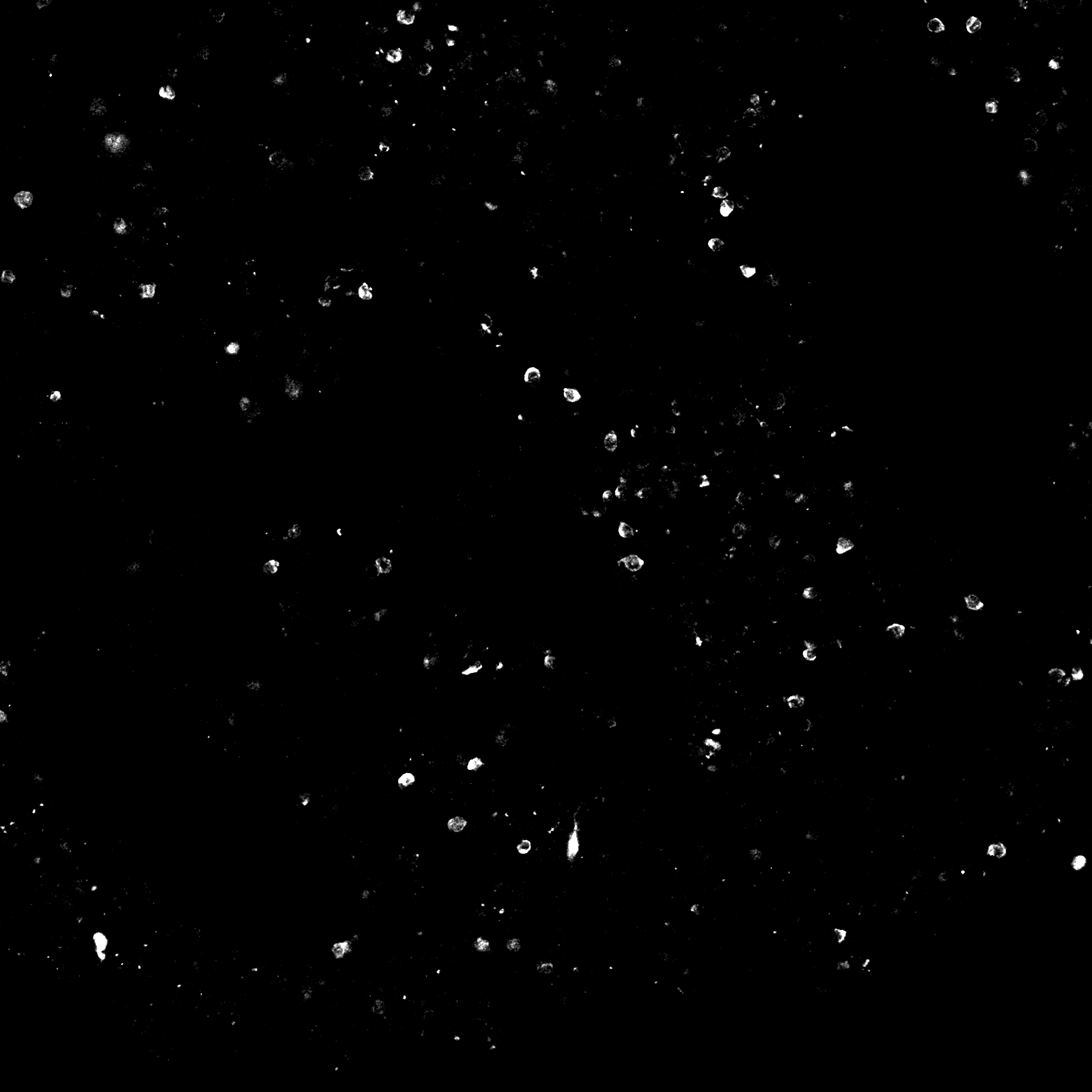

Supplement: Supplementary file 12 — Source Data for Figure 7 [file EMMM-15-e18199-s007.zip › Figure_7/7D/CTRL_Treat._A+B+C_1_month_PDO_T#14_Caspase3_Caspase3.tif]

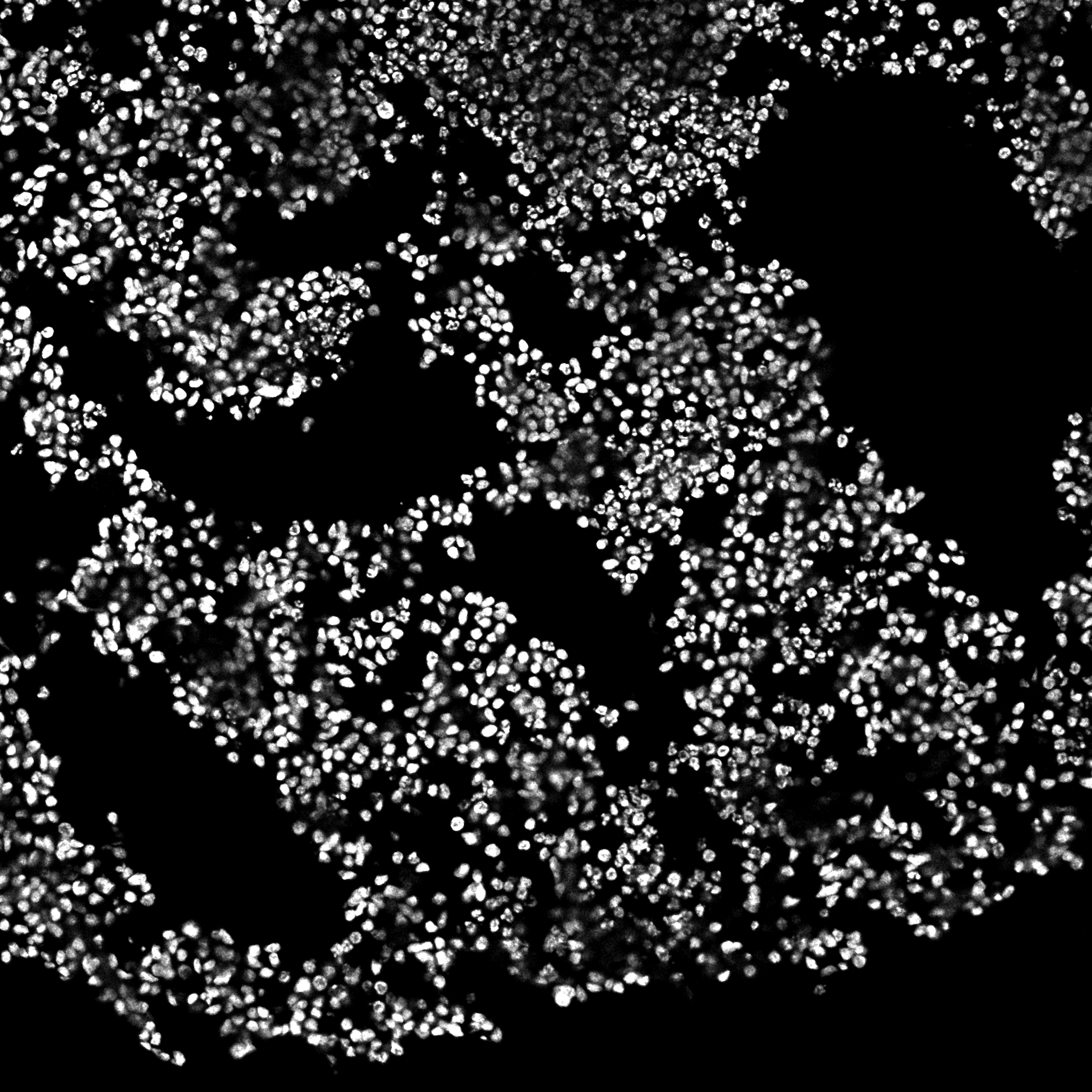

Supplement: Supplementary file 12 — Source Data for Figure 7 [file EMMM-15-e18199-s007.zip › Figure_7/7D/CTRL_Treat._A+B+C_1_month_PDO_T#14_Caspase3_DAPI.tif]

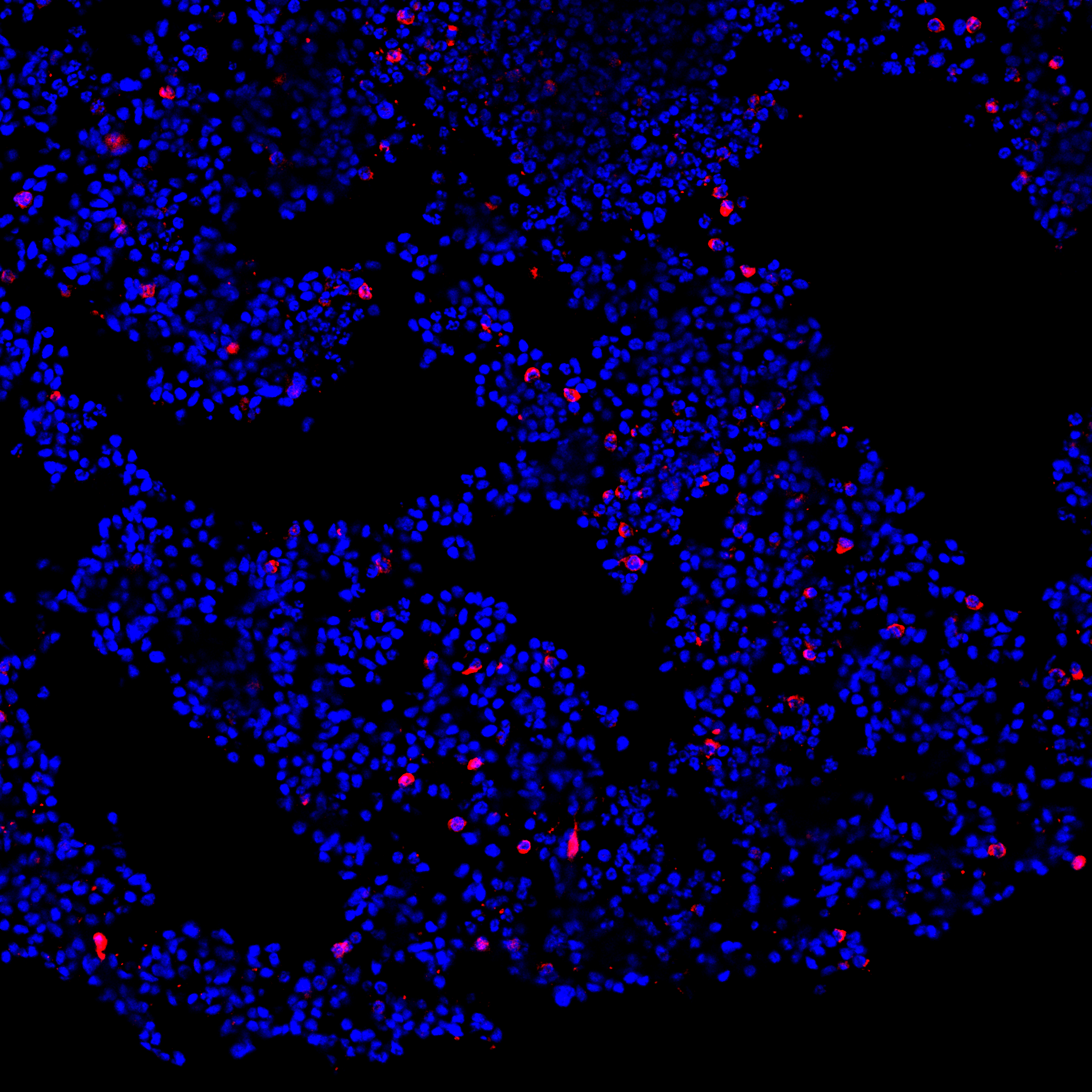

Supplement: Supplementary file 12 — Source Data for Figure 7 [file EMMM-15-e18199-s007.zip › Figure_7/7D/CTRL_Treat._A+B+C_1_month_PDO_T#14_Caspase3_merge.tif]

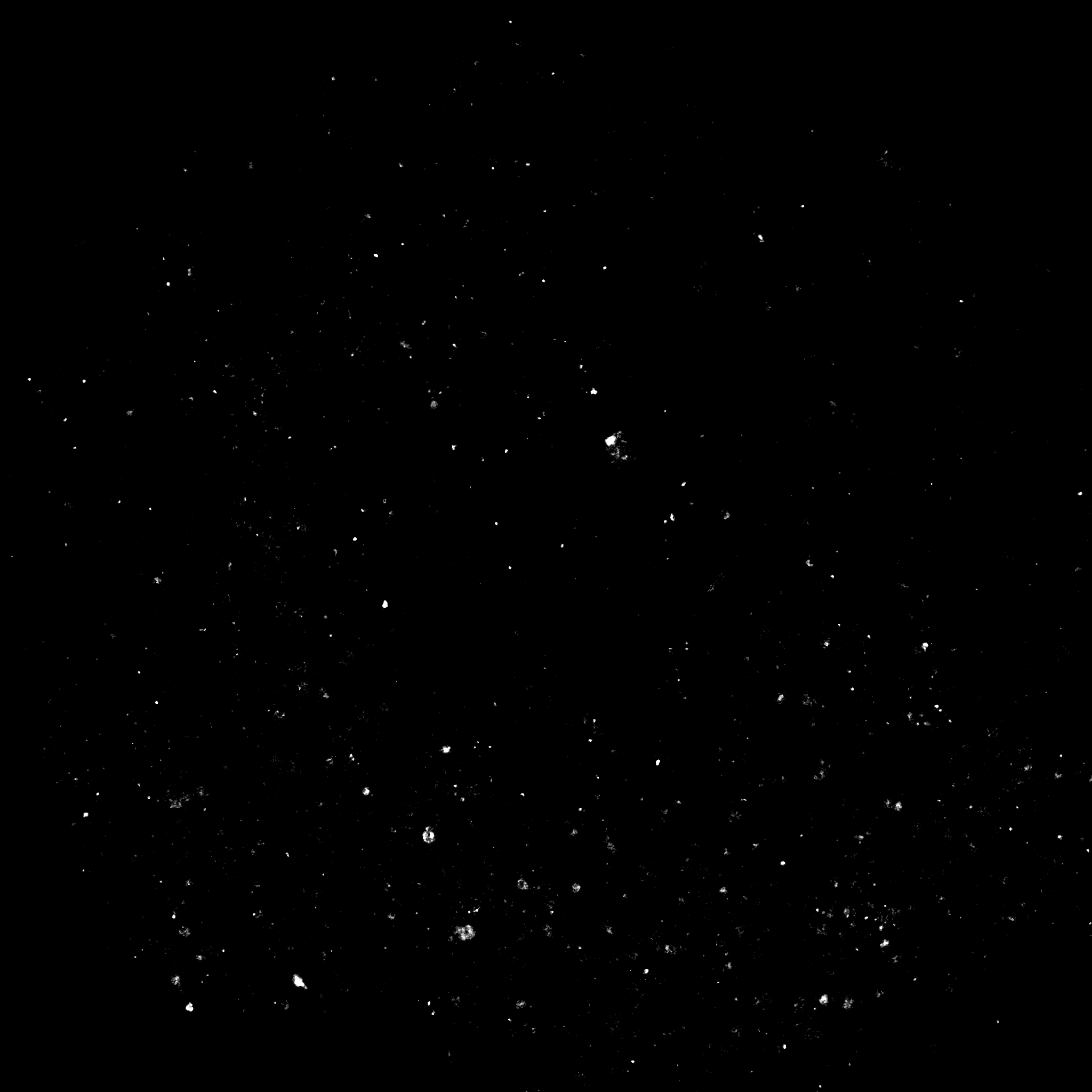

Supplement: Supplementary file 12 — Source Data for Figure 7 [file EMMM-15-e18199-s007.zip › Figure_7/7D/Treat._A+B+C_1_month_PDO_T#14_Caspase3_Caspase3.tif]

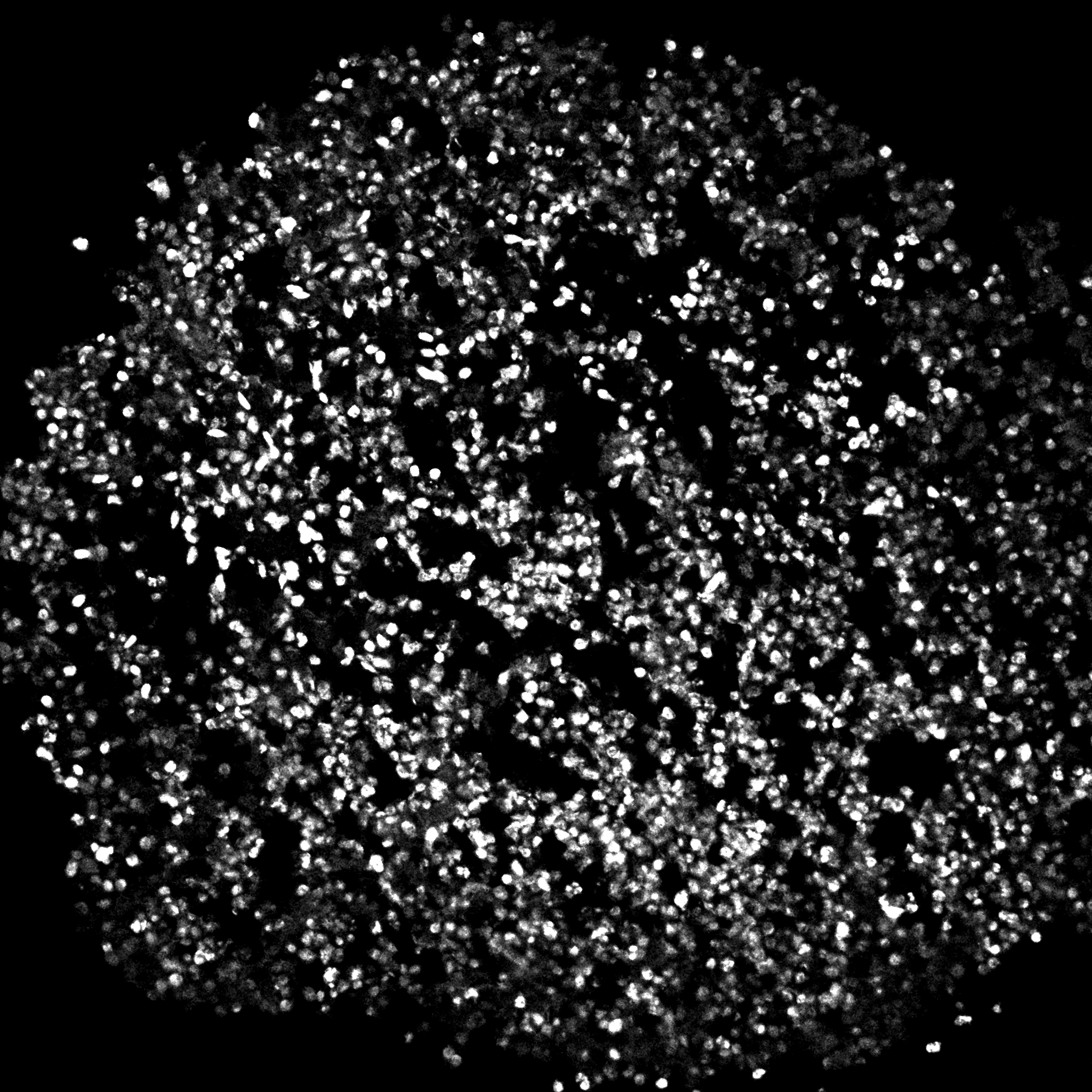

Supplement: Supplementary file 12 — Source Data for Figure 7 [file EMMM-15-e18199-s007.zip › Figure_7/7D/Treat._A+B+C_1_month_PDO_T#14_Caspase3_DAPI.tif]

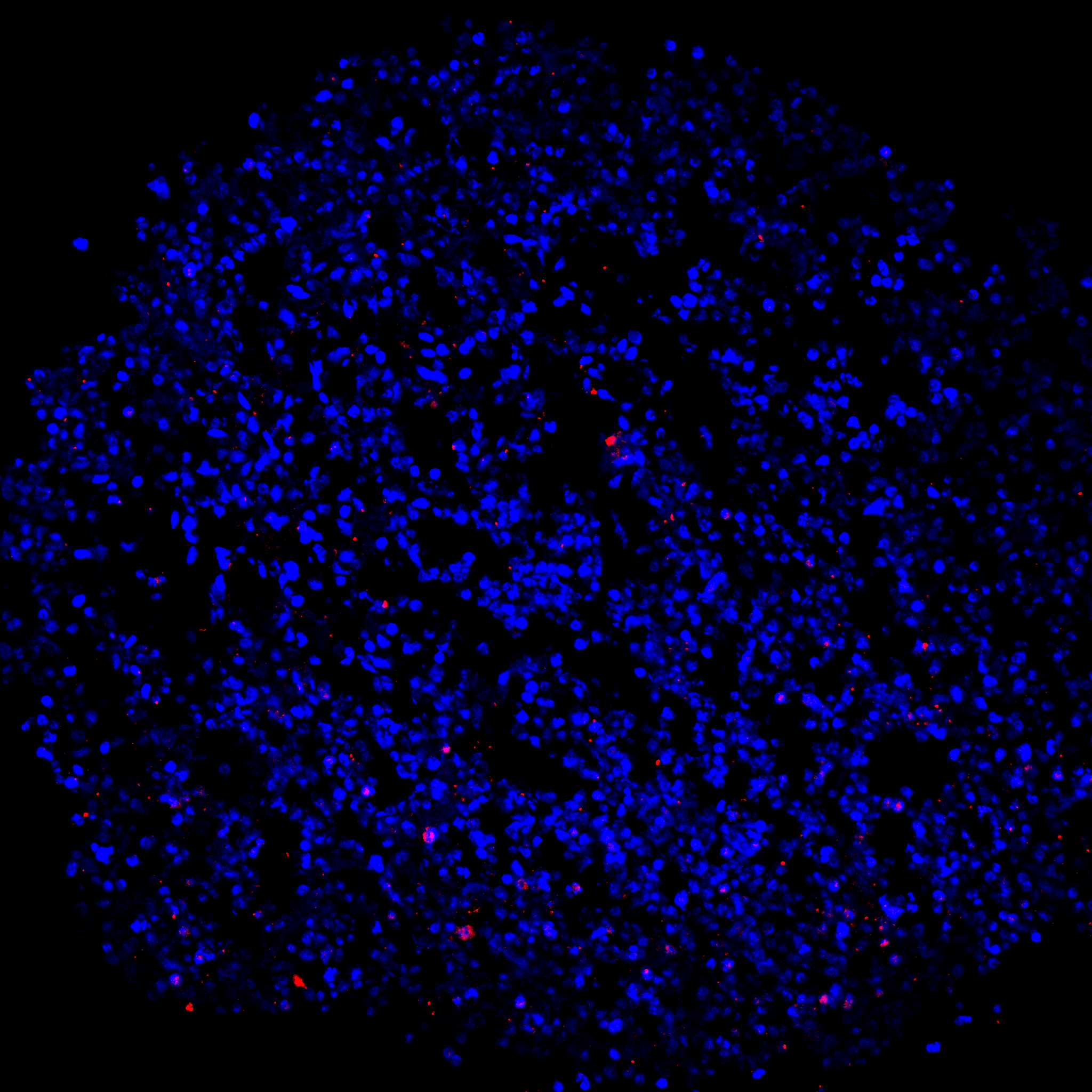

Supplement: Supplementary file 12 — Source Data for Figure 7 [file EMMM-15-e18199-s007.zip › Figure_7/7D/Treat._A+B+C_1_month_PDO_T#14_Caspase3_merge.tif]

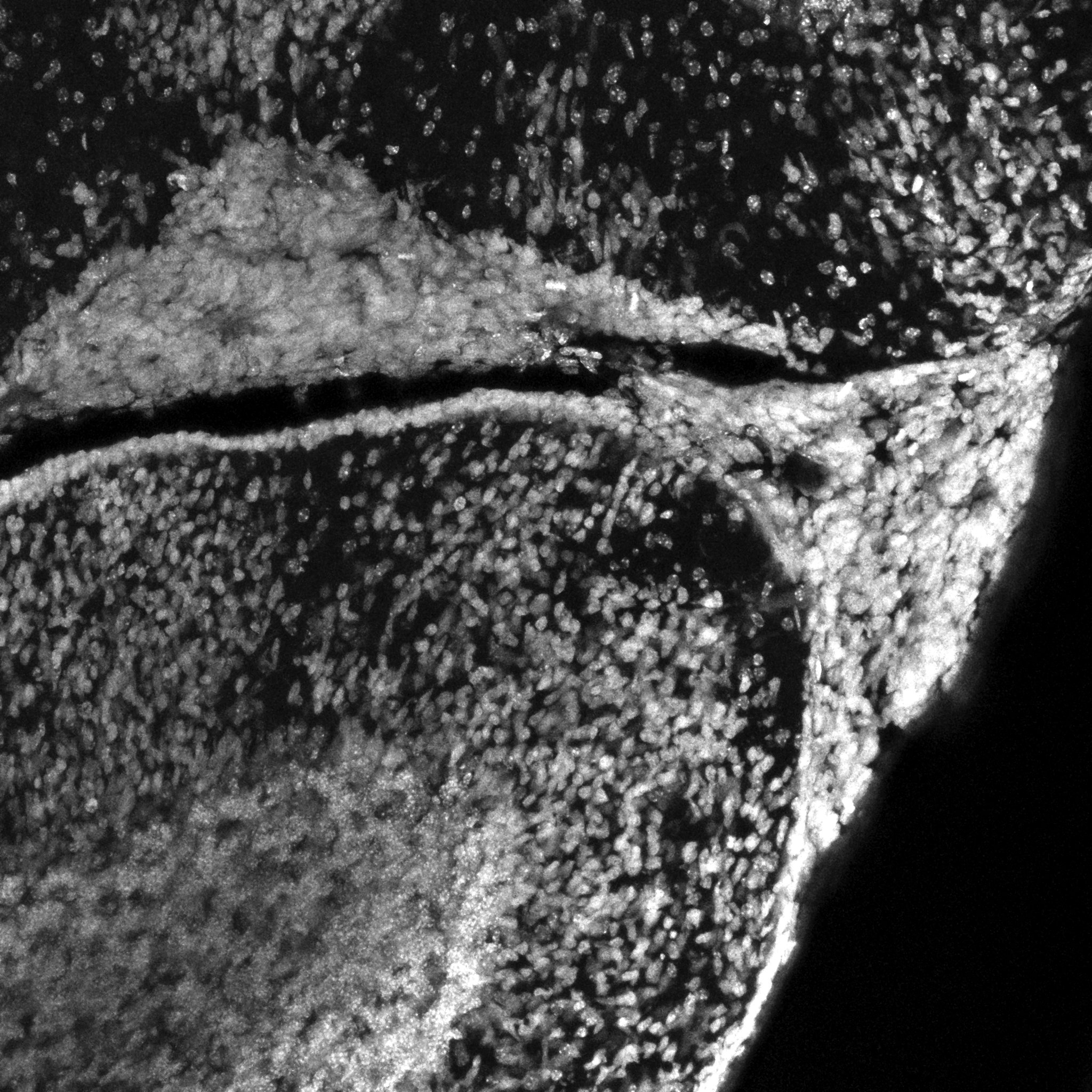

Supplement: Supplementary file 12 — Source Data for Figure 7 [file EMMM-15-e18199-s007.zip › Figure_7/7E/E''_Ki67,_HumAnt_DAPI.tif]

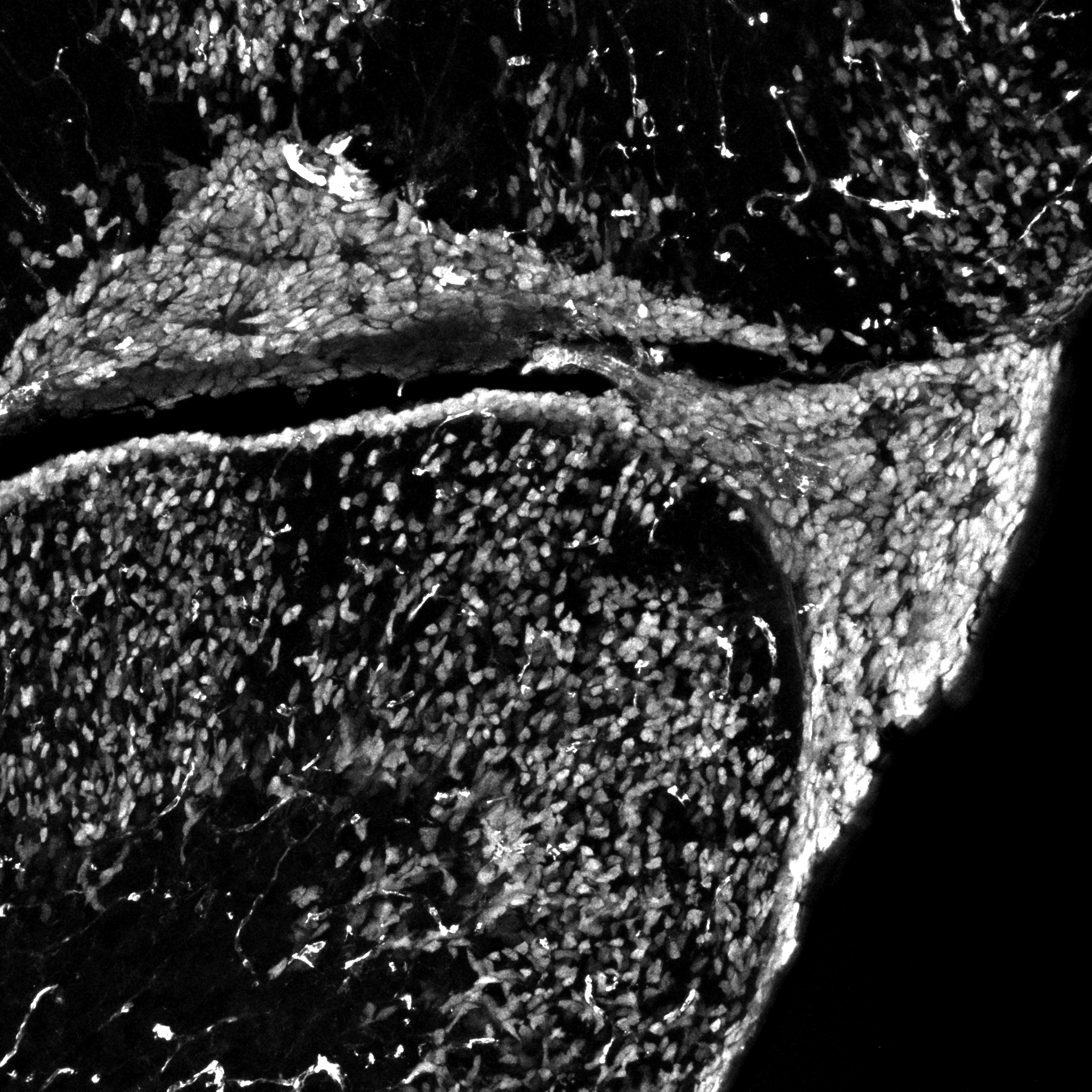

Supplement: Supplementary file 12 — Source Data for Figure 7 [file EMMM-15-e18199-s007.zip › Figure_7/7E/E''_Ki67,_HumAnt_HumAnt.tif]

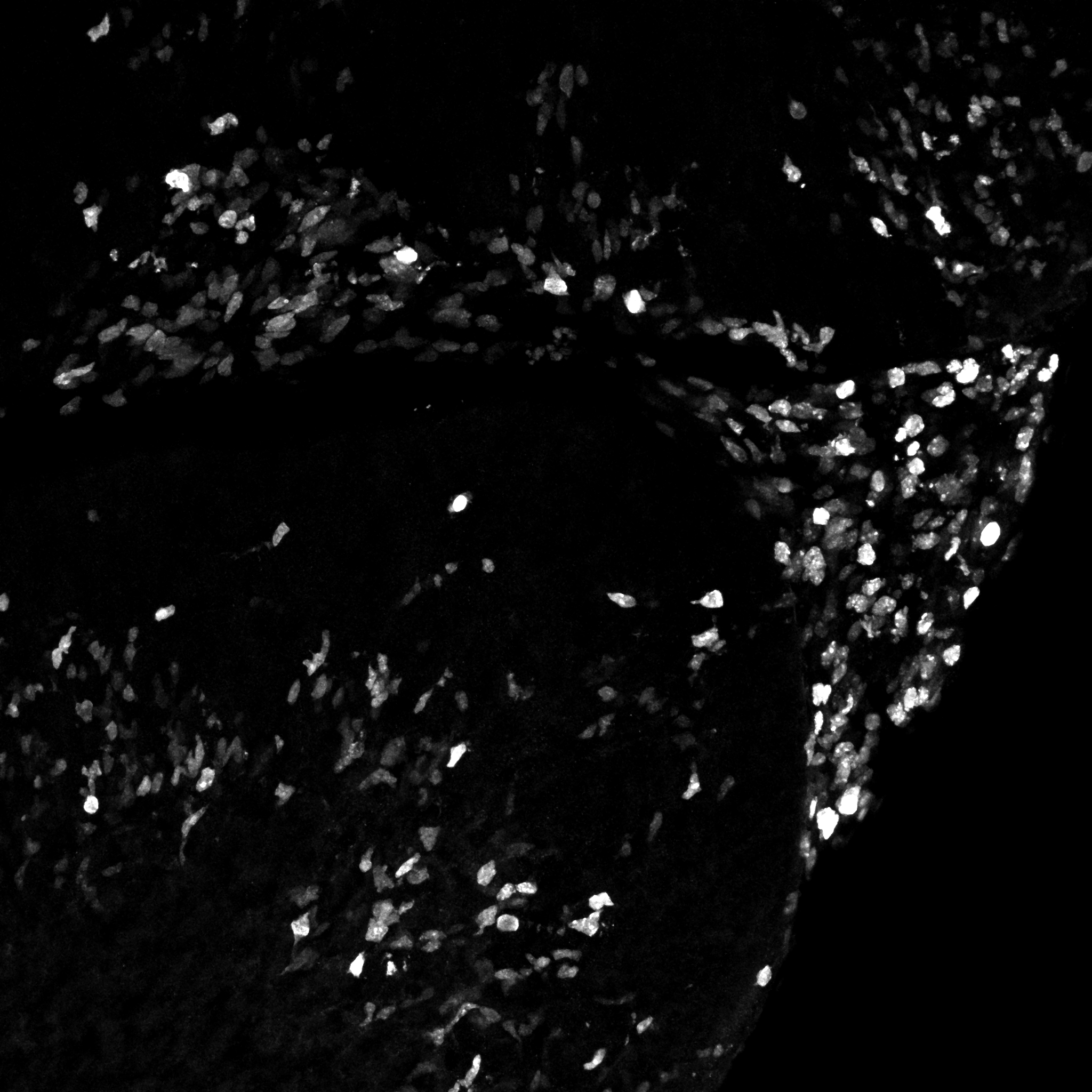

Supplement: Supplementary file 12 — Source Data for Figure 7 [file EMMM-15-e18199-s007.zip › Figure_7/7E/E''_Ki67,_HumAnt_Ki67.tif]

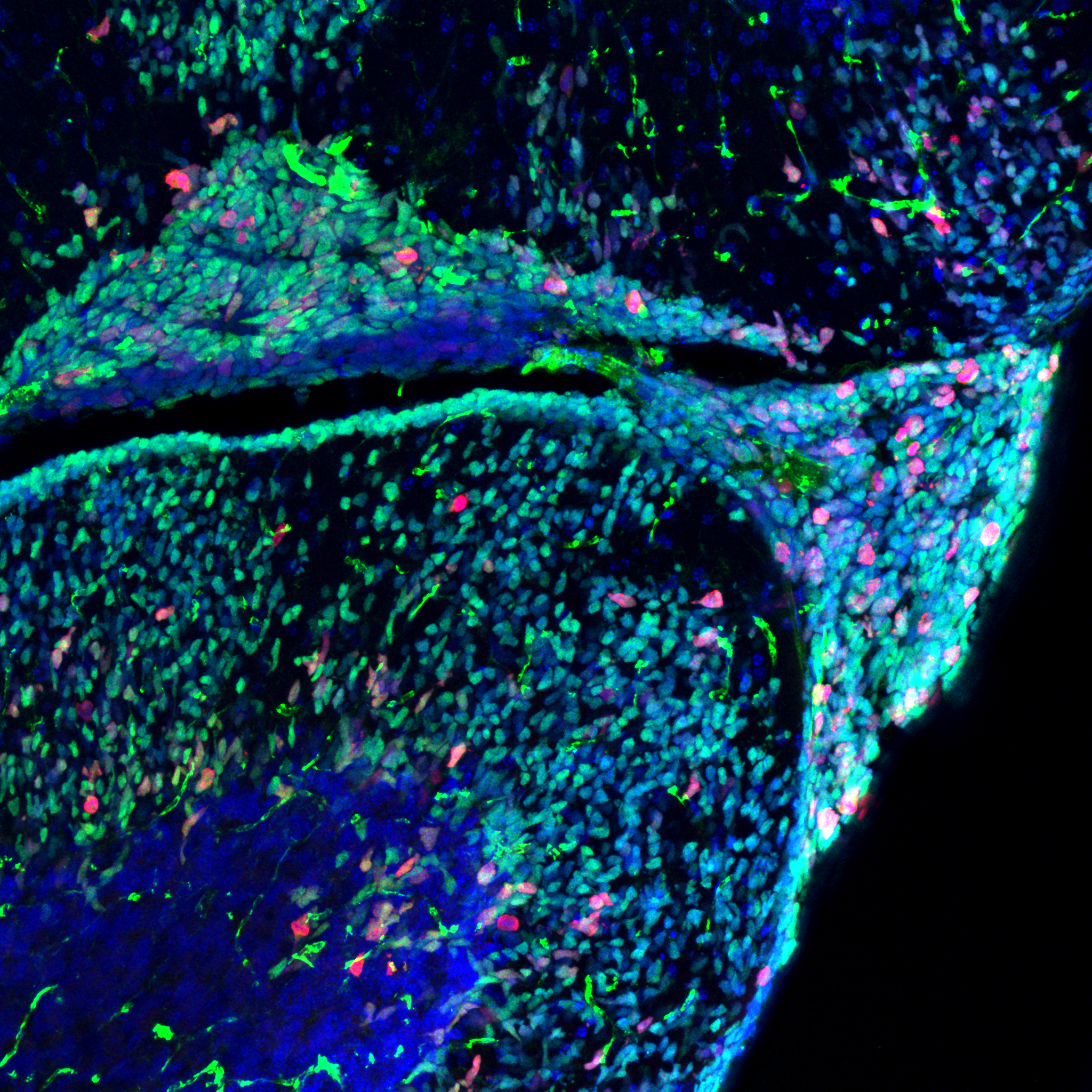

Supplement: Supplementary file 12 — Source Data for Figure 7 [file EMMM-15-e18199-s007.zip › Figure_7/7E/E''_Ki67,_HumAnt_merge.tif]

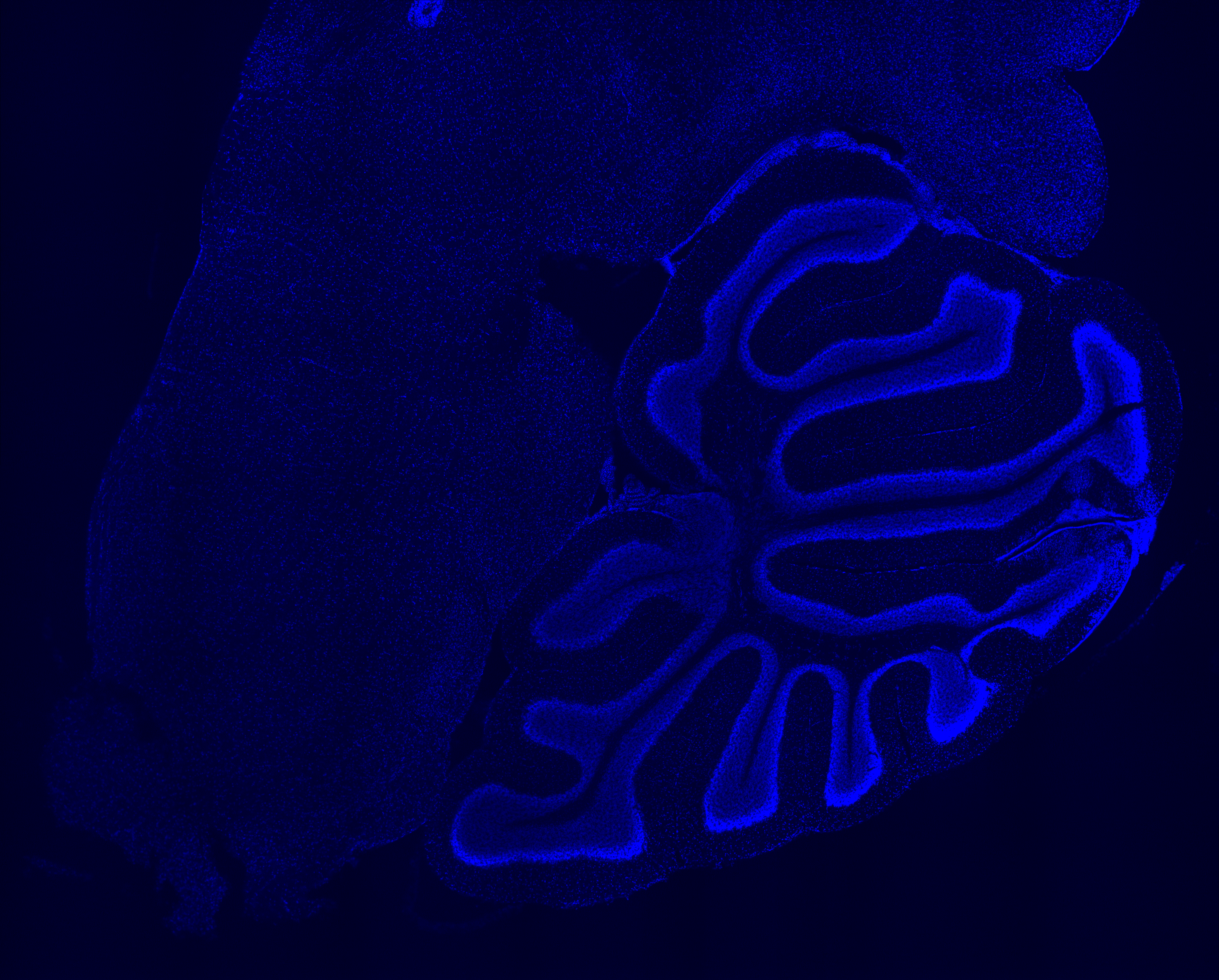

Supplement: Supplementary file 12 — Source Data for Figure 7 [file EMMM-15-e18199-s007.zip › Figure_7/7E/E'_DAPI_mouse_mosaic.tif]

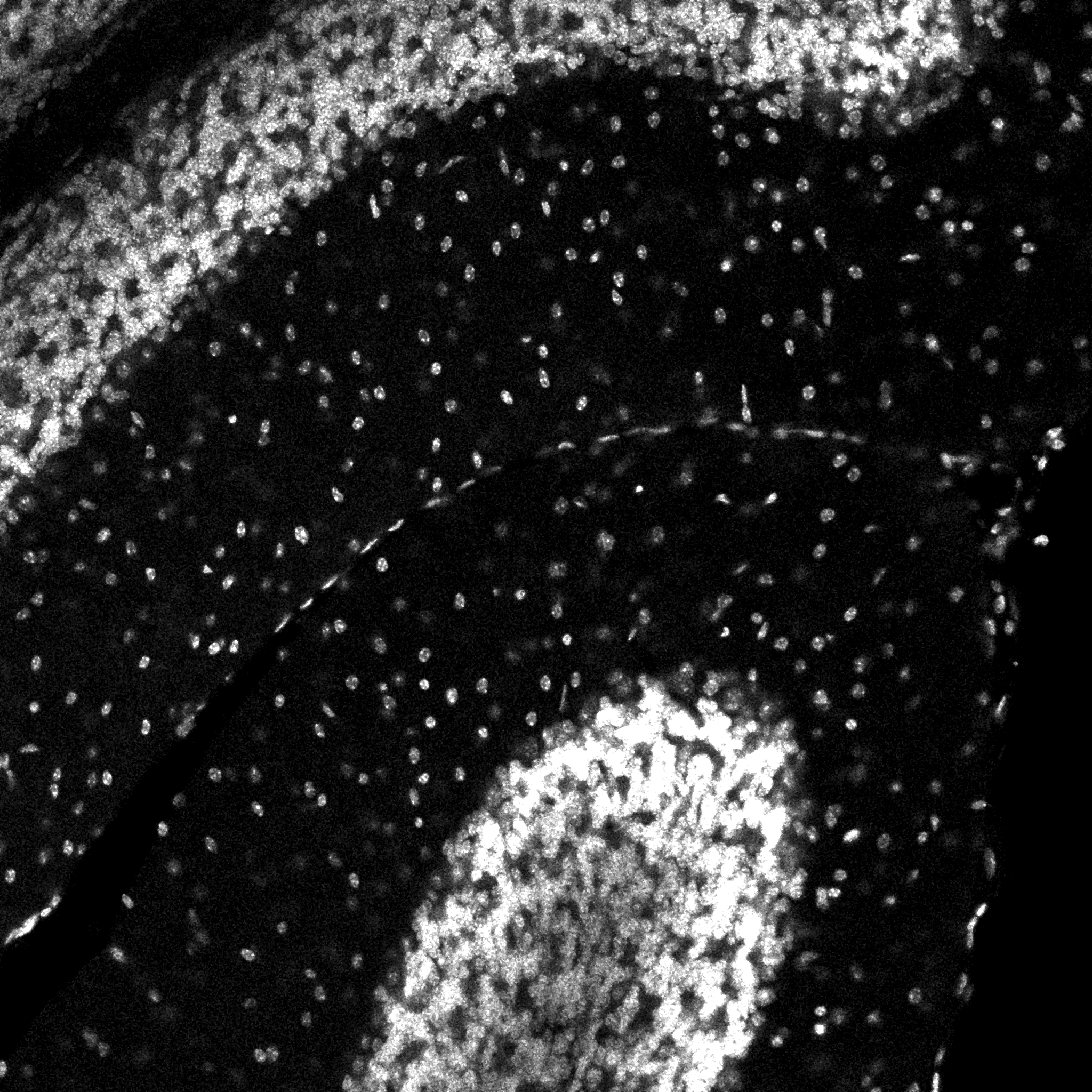

Supplement: Supplementary file 12 — Source Data for Figure 7 [file EMMM-15-e18199-s007.zip › Figure_7/7F/F''_Ki67,_HumAnt_DAPI.tif]

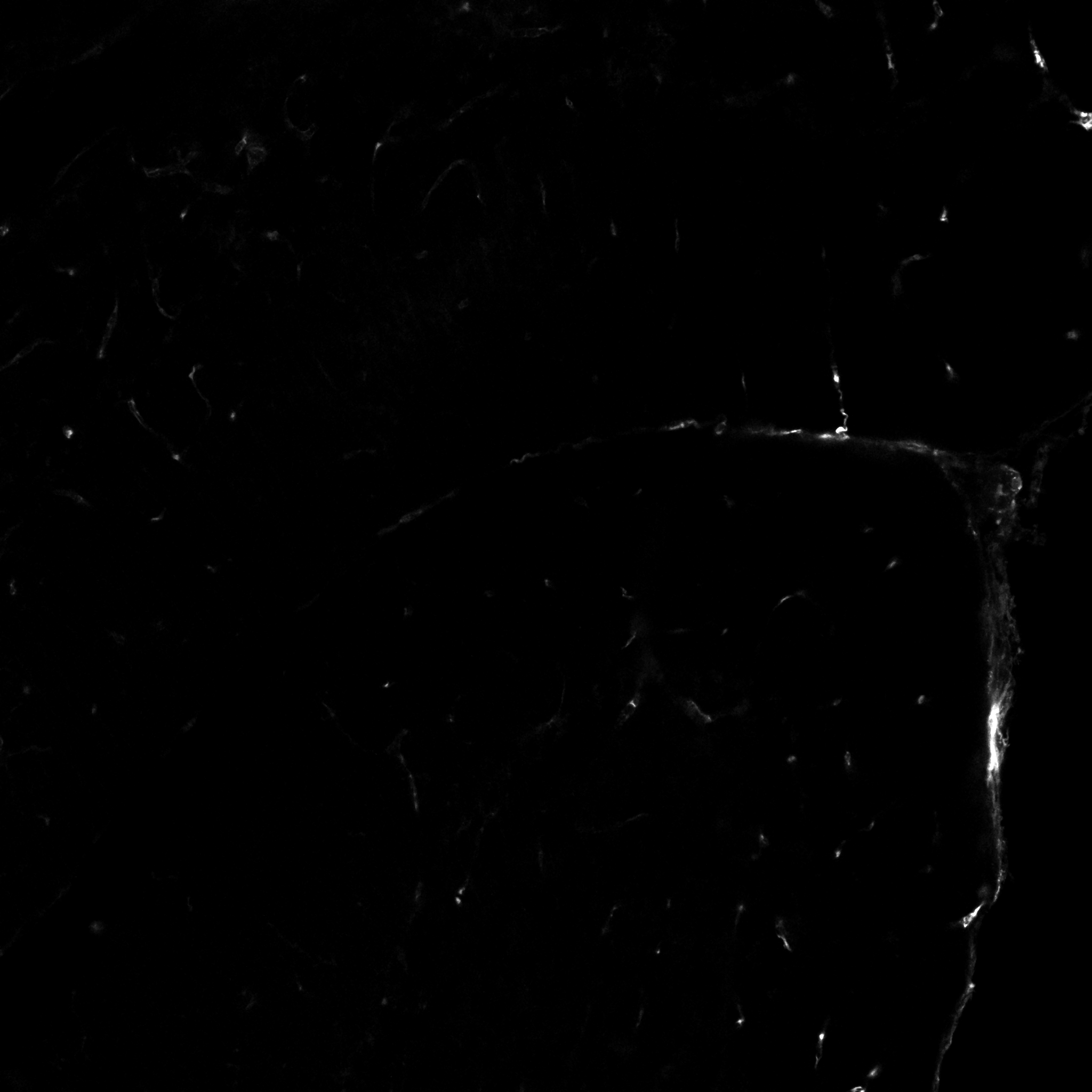

Supplement: Supplementary file 12 — Source Data for Figure 7 [file EMMM-15-e18199-s007.zip › Figure_7/7F/F''_Ki67,_HumAnt_HumAnt.tif]

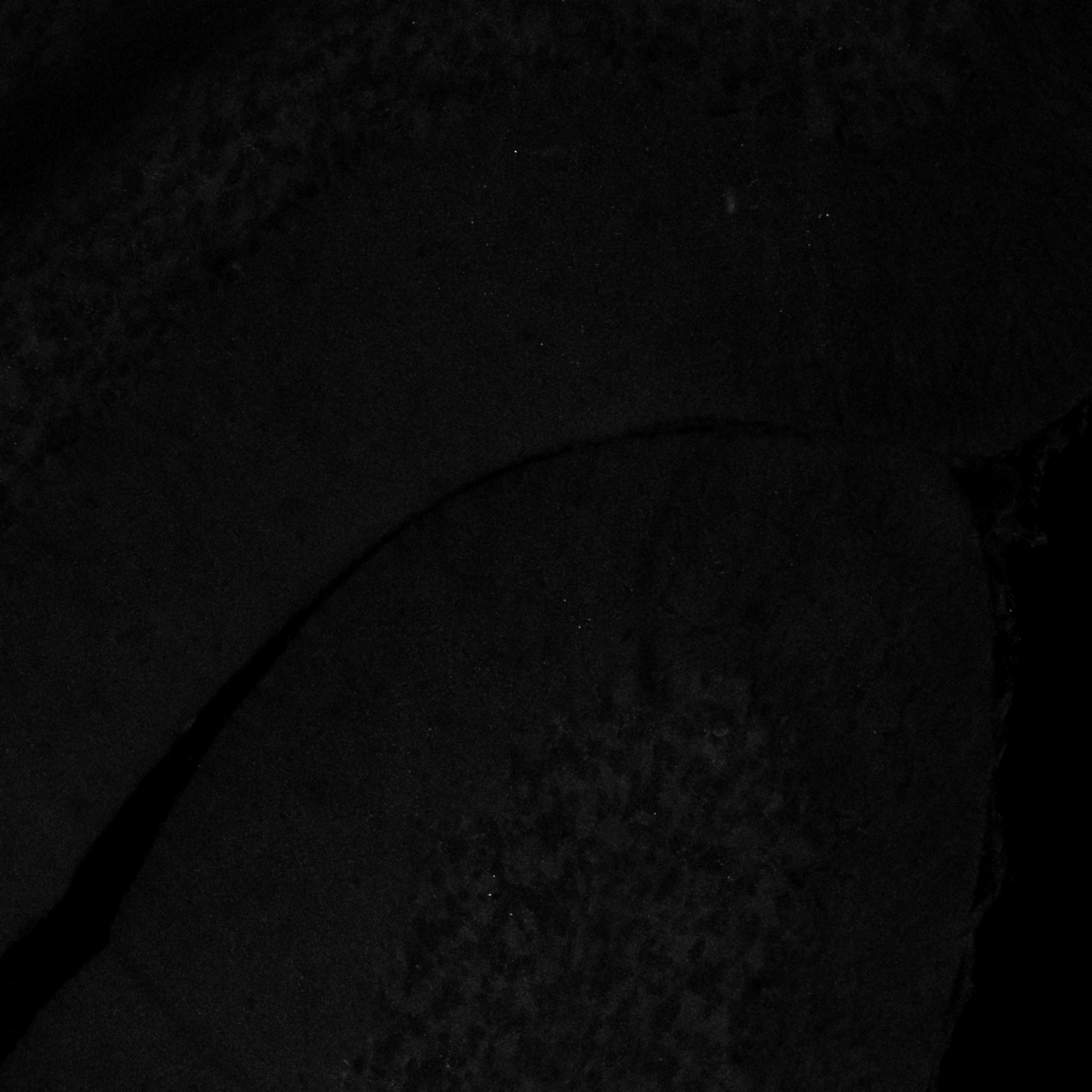

Supplement: Supplementary file 12 — Source Data for Figure 7 [file EMMM-15-e18199-s007.zip › Figure_7/7F/F''_Ki67,_HumAnt_Ki67.tif]

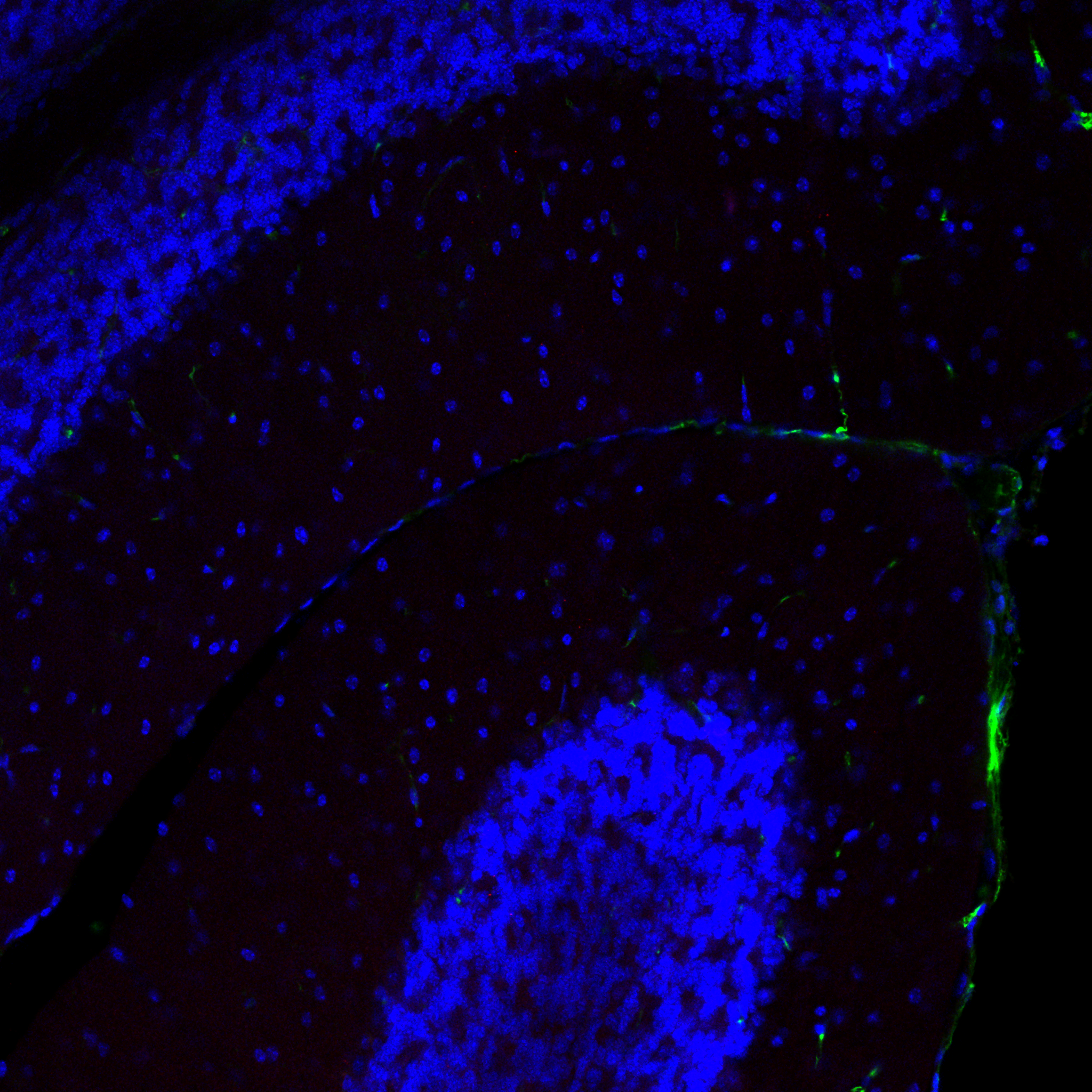

Supplement: Supplementary file 12 — Source Data for Figure 7 [file EMMM-15-e18199-s007.zip › Figure_7/7F/F''_Ki67,_HumAnt_merge.tif]

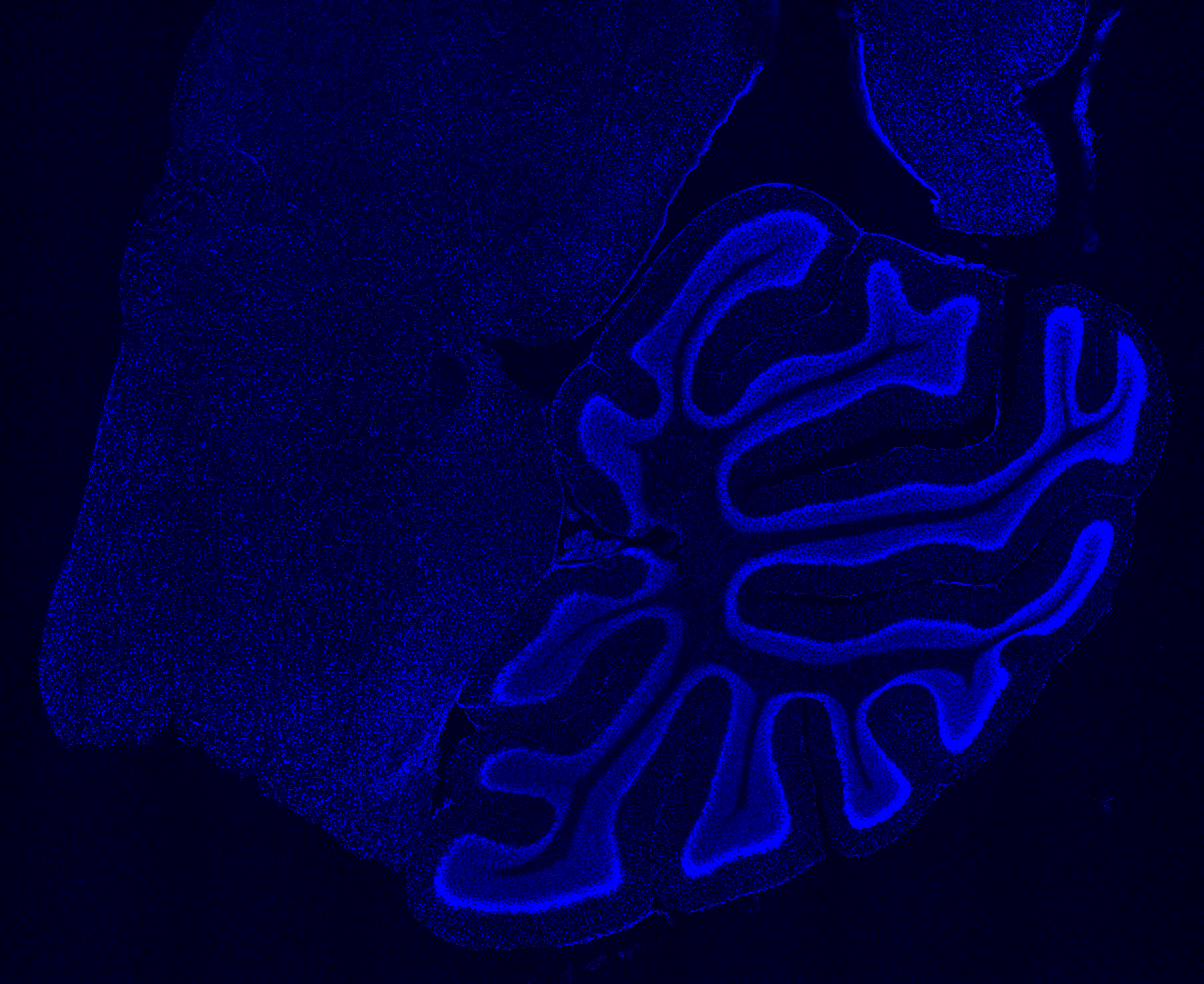

Supplement: Supplementary file 12 — Source Data for Figure 7 [file EMMM-15-e18199-s007.zip › Figure_7/7F/F'_DAPI_mouse_mosaic.tif]

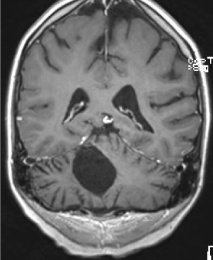

Supplement: Supplementary file 12 — Source Data for Figure 7 [file EMMM-15-e18199-s007.zip › Figure_7/7G/G''''_Tumor_#14_MRI_coronal_plane_off_therapy_post-radiotherapy.tif]

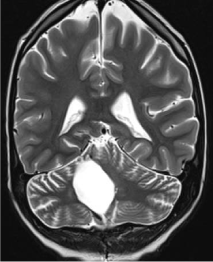

Supplement: Supplementary file 12 — Source Data for Figure 7 [file EMMM-15-e18199-s007.zip › Figure_7/7G/G'''_Tumor_#14_MRI_coronal_plane_post-chemotherapy.tif]

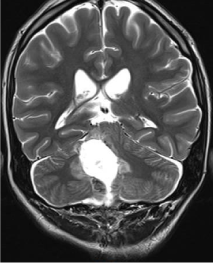

Supplement: Supplementary file 12 — Source Data for Figure 7 [file EMMM-15-e18199-s007.zip › Figure_7/7G/G''_Tumor_#14_MRI_coronal_plane_post-surgery.tif]

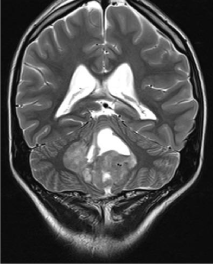

Supplement: Supplementary file 12 — Source Data for Figure 7 [file EMMM-15-e18199-s007.zip › Figure_7/7G/G'_Tumor_#14_MRI_coronal_plane_pre-surgery.tif]
