# Supplementary material for: Patient‐ and xenograft‐derived organoids recapitulate pediatric brain tumor features and patient treatments
Source: EMBO Mol Med. 2023 Nov 30;15(12):e18199. doi: 10.15252/emmm.202318199 (PMC10701620; doi:10.15252/emmm.202318199)
Supplement: Supplementary file 1 — Appendix [file EMMM-15-e18199-s001.pdf]

Appendix

Table of content

Appendix Figure S1

Appendix Figure S2

Appendix Figure S3

Appendix Figure S4

Appendix Figure S5

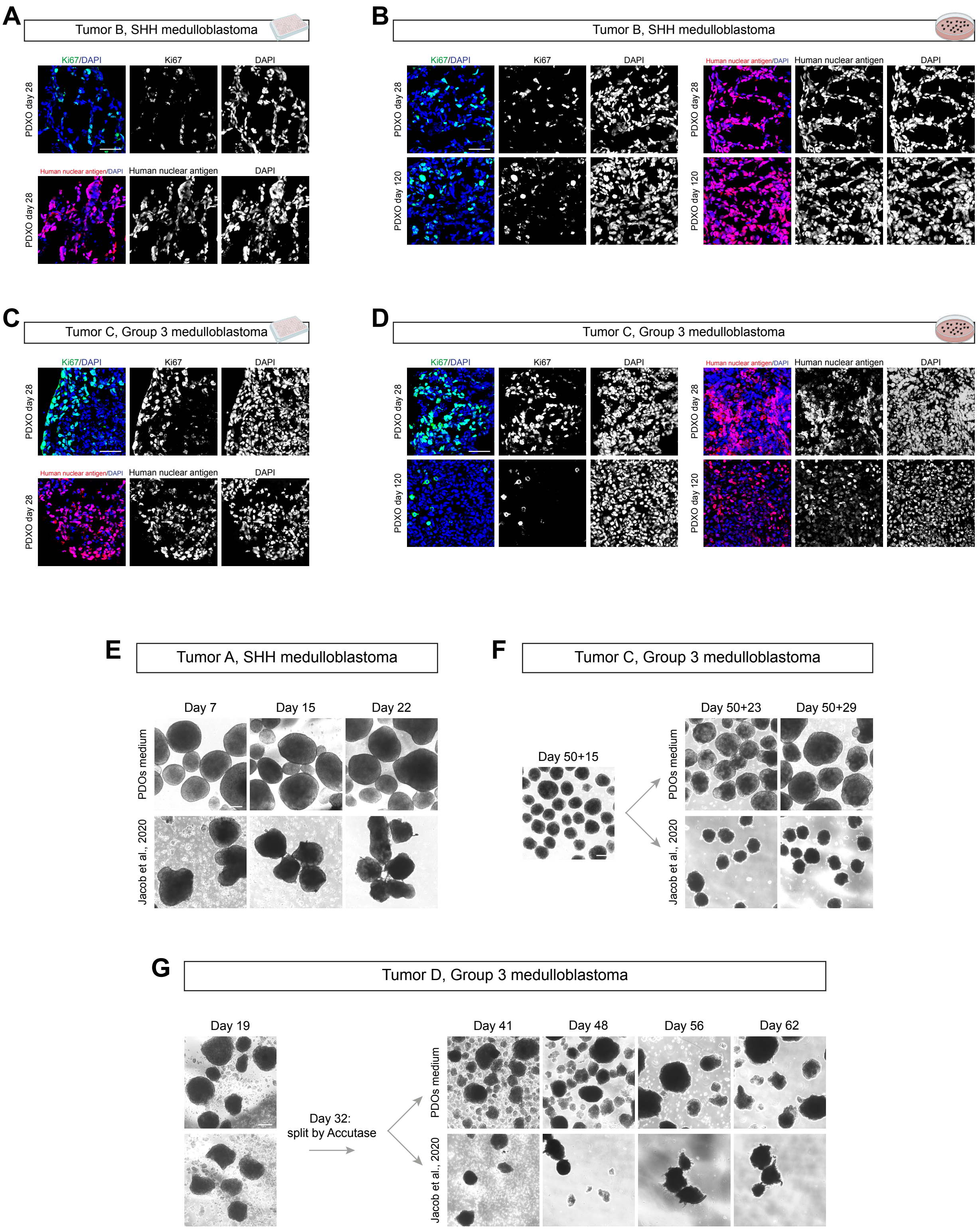

Appendix Figure S1

Appendix Figure S1 - Maintenance of cellular proliferation in PDXOs.

A-B Confocal images of immunofluorescence of Ki67 and human nuclear antigen of PDXOs from SHH MB-derived PDX growth as tumor piece in multiwell (A) or suspension (B).

C-D Confocal images of immunofluorescence of Ki67 and human nuclear antigen of PDXOs from G3 MB-derived PDX growth as tumor piece in multiwell (C) or suspension (D).

E Brightfield images of SHH MB-PDXOs established and grown in PDOs medium or according to (Jacob *et al.*, 2020) at different timepoints.

F-G Brightfield images of G3 MB-PDXOs propagated in PDOs medium or according to (Jacob *et al.*, 2020) at different timepoints.

Scale bar 50 μm (A-D), 200 μm (E-G).

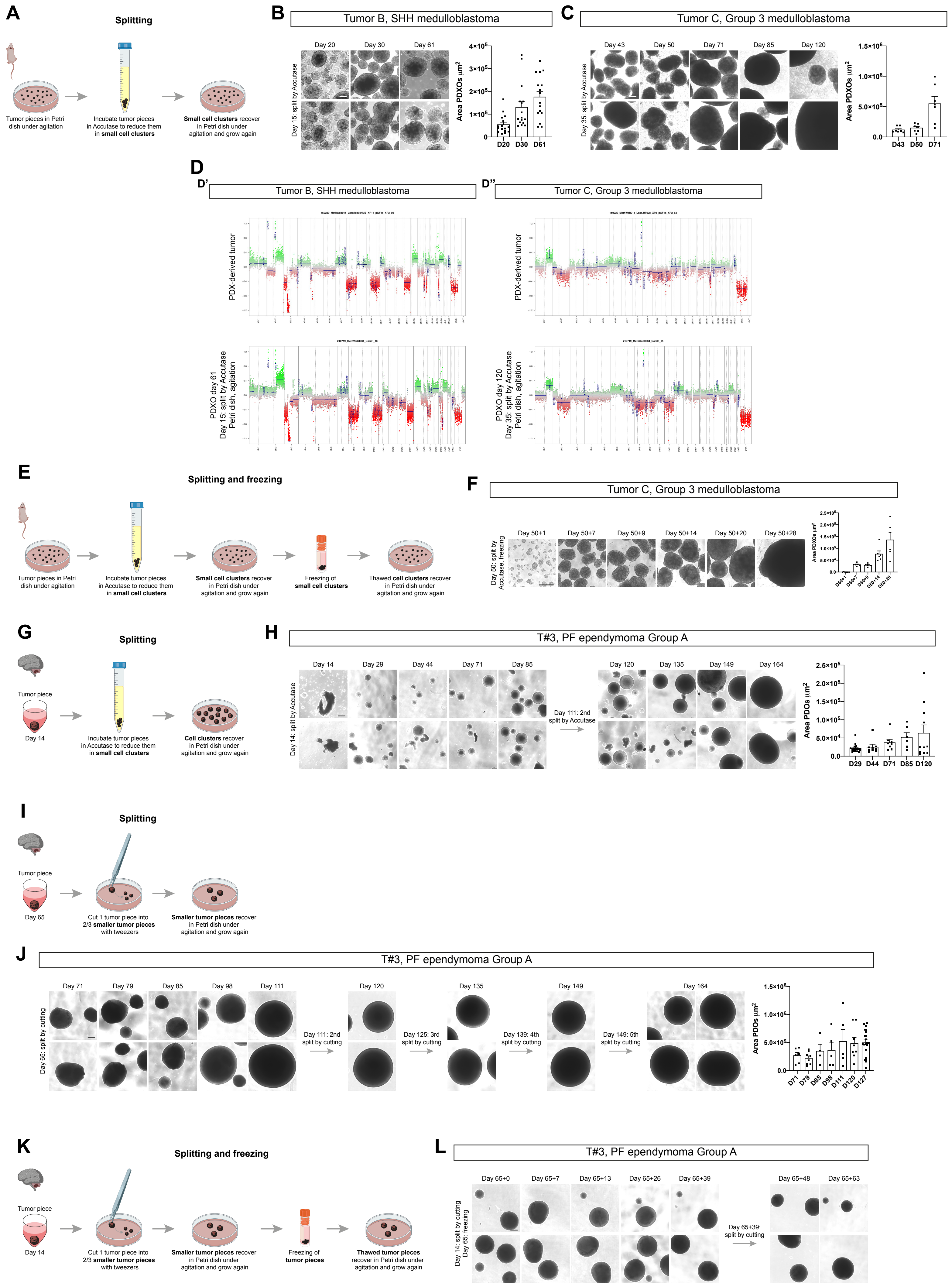

Appendix Figure S2

Appendix Figure S2 - PDXOs and PDOs can be amplified and maintain genomic aberrations.

A Schematic representation of PDXOs amplification through mild enzymatic disaggregation.

B-C Brightfield images of amplified PDXOs from SHH and G3 MB-derived PDXs at different timepoints and PDXOs growth analysis over time.

D Copy number variation profiles comparison between PDX-derived tumor and PDXOs from SHH (D’) and G3 (D’’) MB-derived PDXs after the amplification.

E Schematic representation of PDXOs amplification, cryopreservation and recovery workflow.

F Brightfield images of amplified and cryopreserved PDXOs from G3 MB-derived PDX at different timepoints and PDXOs growth analysis over time.

G Schematic representation of PDOs amplification through mild enzymatic disaggregation.

H Brightfield images of amplified by enzymatic disaggregation EPN-PDOs and PDOs growth analysis over time.

I Schematic representation of PDOs amplification through mechanical cutting.

J Brightfield images of amplified by cutting EPN-PDOs and PDOs growth analysis over time.

K Schematic representation of PDOs amplification, cryopreservation and recovery workflow.

L Brightfield images of amplified and cryopreserved EPN-PDOs at different timepoints.

X axis: chromosomes; Y axis: Log2 copy number ratio. Growth analyses over time data are presented as mean ± s.e.m.; each dot represents a PDO’s (biological replicates) area. n = 16 (B), n = 7 (C), n = 6 (F), n = 5-16 (H), n = 4-21 (J). Scale bar 200 µm in (B-C,F,H,J,L).

DNA methylation (CNV) experiments (D) were performed once per primary tumor/matching PDXOs.

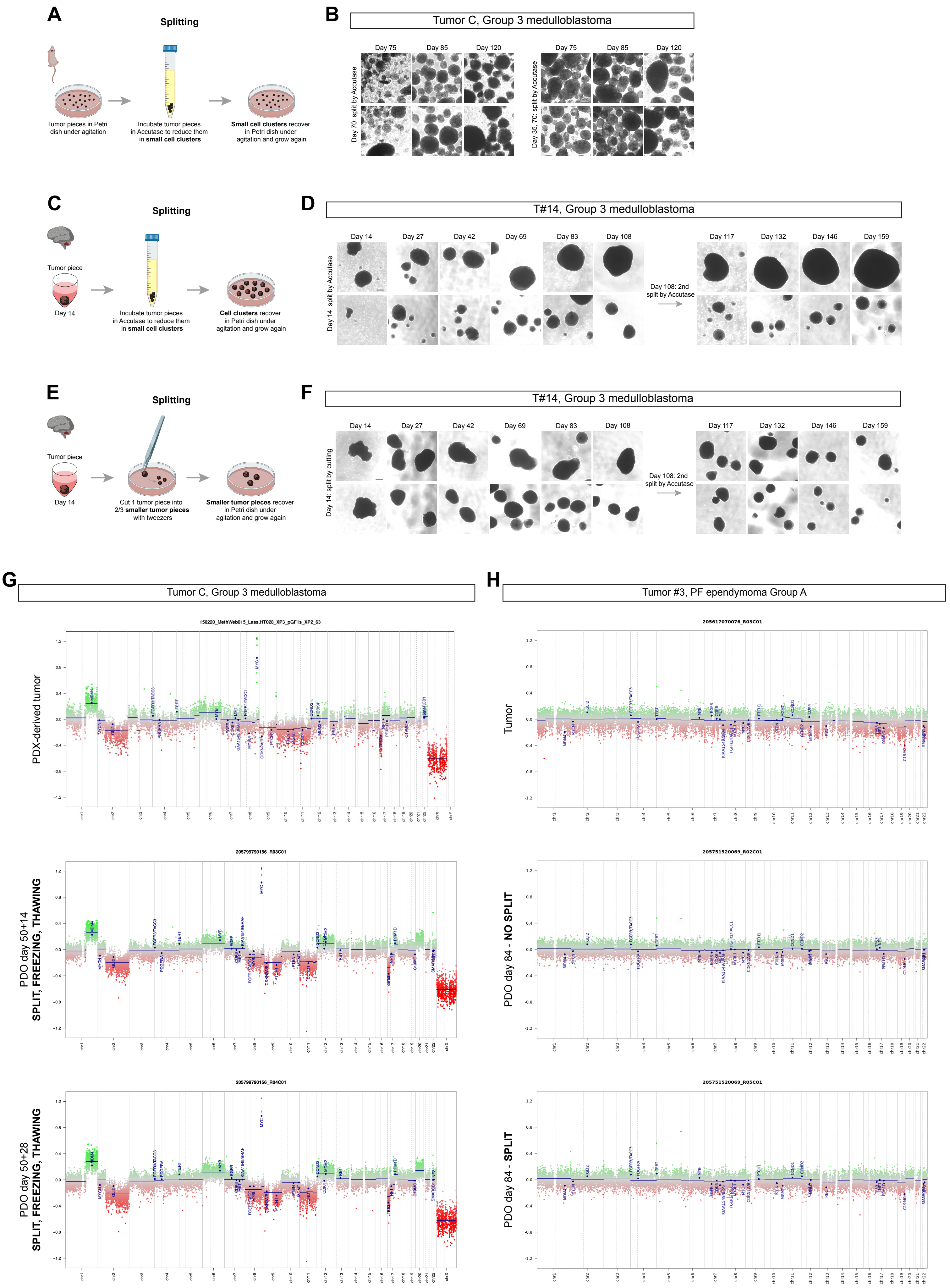

Appendix Figure S3

Appendix Figure S3 - PDXOs and PDOs can be amplified and maintain genomic aberrations.

A Schematic representation of PDXOs amplification through mild enzymatic disaggregation.

B Brightfield images of PDXOs amplified in different moments from G3 MB-derived PDXs at different timepoints.

C Schematic representation of PDOs amplification through mild enzymatic disaggregation.

D Brightfield images of amplified by enzymatic disaggregation MB-PDOs.

E Schematic representation of PDOs amplification through mechanical cutting.

F Brightfield images of amplified by cutting MB-PDOs.

G Copy number variation profiles comparison between PDX-derived tumor and PDXOs from MB-derived PDXs after the amplification.

H Copy number variation profiles comparison between primary parental tumor, EPN-PDOs not amplified and amplified EPN-PDOs.

X axis: chromosomes; Y axis: Log2 copy number ratio. Scale bar 200 μm in (B,D,F).

DNA methylation (CNV) experiments (G,H) were performed once per primary tumor/matching PDOs or PDXOs.

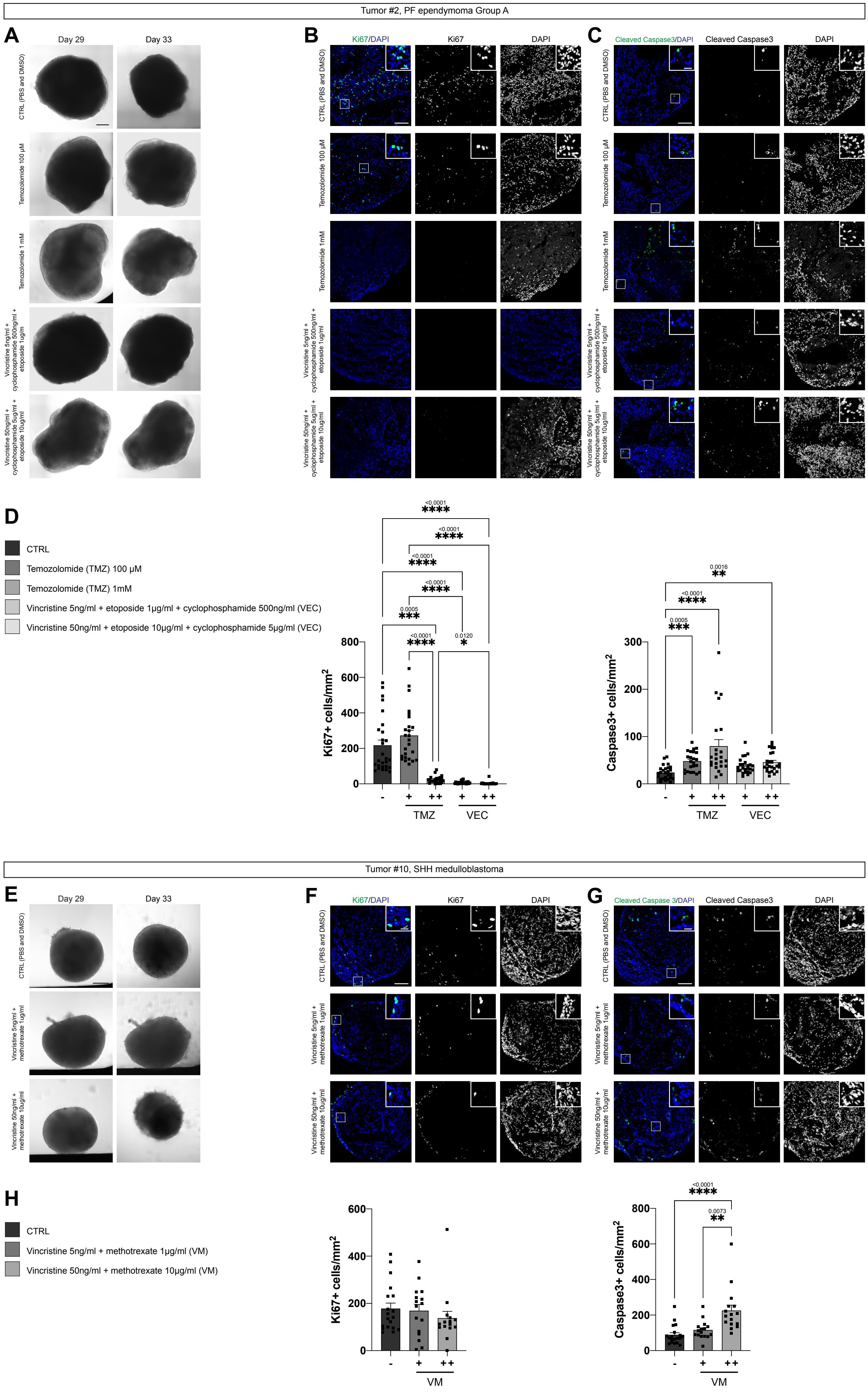

Appendix Figure S4

Appendix Figure S4 - Therapeutic treatments using PDOs in vitro.

A Brightfield images of EPN-PDOs at day 0 and day 5 of drug treatments.

B-C Confocal images of immunofluorescence of Ki67 and cleaved caspase-3 of drug treated EPN-PDOs. The white square marks the region shown at higher magnification in the upper right corner.

D Quantification in EPN-PDOs of Ki67 and cleaved caspase-3 shown as Ki67+ cells/mm^2 and caspase3+ cells/mm^2.

E Brightfield images of MB-PDOs at day 0 and day 5 of drug treatments.

F-G Confocal images of immunofluorescence of Ki67 and cleaved caspase-3 of drug treated MB-PDOs. The white square marks the region shown at higher magnification in the upper right corner.

H Quantification in MB-PDOs of Ki67 and cleaved caspase-3 shown as Ki67+ cells/mm^2 and caspase3+ cells/mm^2.

Data are presented as mean ± s.e.m.; each dot represents a PDO's section. For each treatment condition, n = 4-7 PDOs (biological replicates) where considered; for each PDO, n = 3-4 sections were used. Kruskal-Wallis test with Dunn's post hoc correction; ****: P ≤ 0.0001, ***: P ≤ 0.001, **: P ≤ 0.01, *: P ≤ 0.05. Adjusted P values are reported in figure. Scale bar 200 μm in (**a,e**); 100 μm and 20 μm (higher magnification) in (B-C, F-G). TMZ, temozolomide: VEC, vincristine, etoposide, methotrexate; VM, vincristine, methotrexate.

Drug treatment and quantifications experiments were performed once per primary tumor/matching PDOs.

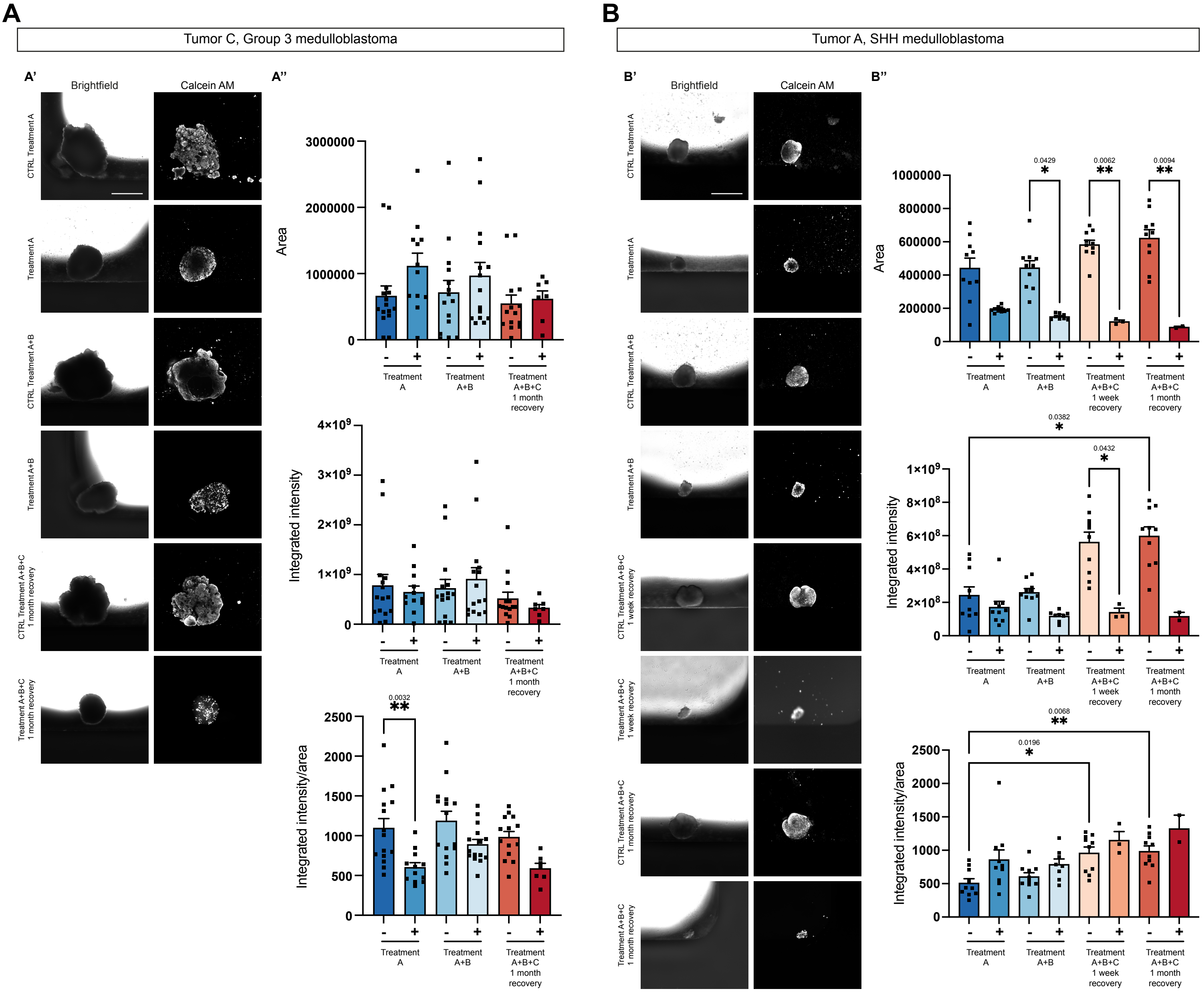

Appendix Figure S5

Appendix Figure S5 - G3 MB and SHH MB-derived PDXOs respond in different ways to high-risk medulloblastoma protocol.

A G3 MB-PDXOs live cells analysis with brightfield and fluorescence images (A') and quantification of area, integrated intensity, and integrated intensity/area after the treatment (A'').

B SHH MB-PDXOs live cells analysis with brightfield and fluorescence images (B') and quantification of area, integrated intensity, and integrated intensity/area after the treatment (B'').

Data are presented as mean ± s.e.m.; each dot represents a PDO's. For each treatment condition, n = 2-15 PDOs (biological replicates) where considered. Ordinary one-way ANOVA or Kruskal-Wallis test with Dunn's post hoc test; **: P ≤ 0.01, *: P ≤ 0.05. Adjusted P values are reported in figure. Scale bar 1 mm in (A',B'). High-risk medulloblastoma protocol treatment experiment was performed once in G3- and SHH MB PDXOs.
