# Supplementary material for: Patient‐ and xenograft‐derived organoids recapitulate pediatric brain tumor features and patient treatments
Source: EMBO Mol Med. 2023 Nov 30;15(12):e18199. doi: 10.15252/emmm.202318199 (PMC10701620; doi:10.15252/emmm.202318199)
Supplement: Supplementary file 13 [file EMMM-15-e18199-s010.pdf]

EMBO Molecular Medicine

Patient and xenograft-derived organoids recapitulate brain tumor features and patient treatments

Luca Tiberi, Chiara Lago, Aniello Federico, Gloria Leva, Norman Mack, Benjamin Schwalm, Claudio Ballabio, Matteo Gianesello, Luana Abballe, Isabella Giovannoni, Sofia Reddel, Sabrina Rossi, Nicolas Leone, Andrea Carai, Angela Mastronuzzi, Alessandra Bisio, Alessia Soldano, Concetta Quintarelli, Franco Locatelli, Marcel Kool, and Evelina Miele
DOI: 10.15252/emmm.202318199

Corresponding authors: Luca Tiberi (luca.tiberi@unitn.it) , Evelina Miele (evelina.miele@opbg.net), Marcel Kool (m.kool@kitz-heidelberg.de)

Review Timeline:

Submission Date:	21st Jun 23
Editorial Decision:	13th Jul 23
Revision Received:	18th Sep 23
Editorial Decision:	17th Oct 23
Revision Received:	19th Oct 23
Accepted:	24th Oct 23

Editor: Lise Roth

Transaction Report:

(Note: With the exception of the correction of typographical or spelling errors that could be a source of ambiguity, letters and reports are not edited. Depending on transfer agreements, referee reports obtained elsewhere may or may not be included in this compilation. Referee reports are anonymous unless the Referee chooses to sign their reports.)

13th Jul 2023

Dear Prof. Tiberi,

Thank you for the submission of your new manuscript to EMBO Molecular Medicine. We have now received feedback from the three reviewers who agreed to evaluate your manuscript. Referees #1 and #2 had reviewed your initial manuscript (and were previously referees #1 and #3, respectively), and referee #3 is a new referee. All referees had access to your point-by-point rebuttal letter. As you will see from the reports below, they are supporting publication of your work pending appropriate revisions.

Addressing the reviewers' concerns in full will be necessary for further considering the manuscript in our journal, and acceptance of the manuscript will entail a second round of review. EMBO Molecular Medicine encourages a single round of revision only and therefore, acceptance or rejection of the manuscript will depend on the completeness of your responses included in the next, final version of the manuscript. For this reason, and to save you from any frustrations in the end, I would strongly advise against returning an incomplete revision.

If you would like to discuss further the points raised by the referees, I am available to do so via email or video. Let me know if you are interested in this option.

When submitting your revised manuscript, please carefully review the instructions that follow below. We perform an initial quality control of all revised manuscripts before re-review; failure to include requested items will delay the evaluation of your revision.

We require:

1) A .docx formatted version of the manuscript text (including legends for main figures, EV figures and tables). Please make sure that the changes are highlighted to be clearly visible.

2) Individual production quality figure files as .eps, .tif, .jpg (one file per figure). For guidance, download the 'Figure Guide PDF' (https://www.embopress.org/page/journal/17574684/authorguide#figureformat).

3) At EMBO Press we ask authors to provide source data for the main figures. Our source data coordinator will contact you to discuss which figure panels we would need source data for and will also provide you with helpful tips on how to upload and organize the files.

4) A .docx formatted letter INCLUDING the reviewers' reports and your detailed point-by-point responses to their comments. As part of the EMBO Press transparent editorial process, the point-by-point response is part of the Review Process File (RPF), which will be published alongside your paper.

5) A complete author checklist, which you can download from our author guidelines (https://www.embopress.org/page/journal/17574684/authorguide#submissionofrevisions). Please insert information in the checklist that is also reflected in the manuscript. The completed author checklist will also be part of the RPF.

6) Please note that all corresponding authors are required to supply an ORCID ID for their name upon submission of a revised manuscript. An ORCID identifier is currently missing for Dr. Madhur Agrawal.

7) It is mandatory to include a 'Data Availability' section after the Materials and Methods. Before submitting your revision, primary datasets produced in this study need to be deposited in an appropriate public database, and the accession numbers and database listed under 'Data Availability'. Please remember to provide a reviewer password if the datasets are not yet public (see https://www.embopress.org/page/journal/17574684/authorguide#dataavailability).

In case you have no data that requires deposition in a public database, please state so in this section ("This study includes no data deposited in external repositories"). Note that the Data Availability Section is restricted to new primary data that are part of this study.
8) For data quantification: please specify the name of the statistical test used to generate error bars and P values, the number (n) of independent experiments (specify technical or biological replicates) underlying each data point and the test used to calculate p-values in each figure legend. The figure legends should contain a basic description of n, P and the test applied. Graphs must include a description of the bars and the error bars (s.d., s.e.m.). Please provide exact p values.

9) Our journal encourages inclusion of *data citations in the reference list* to directly cite datasets that were re-used and obtained from public databases. Data citations in the article text are distinct from normal bibliographical citations and should directly link to the database records from which the data can be accessed. In the main text, data citations are formatted as follows: "Data ref: Smith et al, 2001" or "Data ref: NCBI Sequence Read Archive PRJNA342805, 2017". In the Reference list,

data citations must be labeled with "[DATASET]". A data reference must provide the database name, accession number/identifiers and a resolvable link to the landing page from which the data can be accessed at the end of the reference. Further instructions are available at .

10) We replaced Supplementary Information with Expanded View (EV) Figures and Tables that are collapsible/expandable online. A maximum of 5 EV Figures can be typeset. EV Figures should be cited as 'Figure EV1, Figure EV2" etc... in the text and their respective legends should be included in the main text after the legends of regular figures.
- For the figures that you do NOT wish to display as Expanded View figures, they should be bundled together with their legends in a single PDF file called *Appendix*, which should start with a short Table of Content. Appendix figures should be referred to in the main text as: "Appendix Figure S1, Appendix Figure S2" etc.
- Additional Tables/Datasets should be labeled and referred to as Table EV1, Dataset EV1, etc. Legends have to be provided in a separate tab in case of .xls files. Alternatively, the legend can be supplied as a separate text file (README) and zipped together with the Table/Dataset file.
See detailed instructions here:

11) The paper explained: EMBO Molecular Medicine articles are accompanied by a summary of the articles to emphasize the major findings in the paper and their medical implications for the non-specialist reader. Please provide a draft summary of your article highlighting
- the medical issue you are addressing,
- the results obtained and
- their clinical impact.
This may be edited to ensure that readers understand the significance and context of the research. Please refer to any of our published articles for an example.

12) For more information: There is space at the end of each article to list relevant web links for further consultation by our readers. Could you identify some relevant ones and provide such information as well? Some examples are patient associations, relevant databases, OMIM/proteins/genes links, author's websites, etc...

13) Author contributions: CRediT has replaced the traditional author contributions section because it offers a systematic machine readable author contributions format that allows for more effective research assessment. Please remove the Authors Contributions from the manuscript and use the free text boxes beneath each contributing author's name in our system to add specific details on the author's contribution. More information is available in our guide to authors.

14) Disclosure statement and competing interests: We updated our journal's competing interests policy in January 2022 and request authors to consider both actual and perceived competing interests. Please review the policy https://www.embopress.org/competing-interests and update your competing interests if necessary.

15) Every published paper now includes a 'Synopsis' to further enhance discoverability. Synopses are displayed on the journal webpage and are freely accessible to all readers. They include a short stand first (maximum of 300 characters, including space) as well as 2-5 one-sentences bullet points that summarizes the paper. Please write the bullet points to summarize the key NEW findings. They should be designed to be complementary to the abstract - i.e. not repeat the same text. We encourage inclusion of key acronyms and quantitative information (maximum of 30 words / bullet point). Please use the passive voice. Please attach these in a separate file or send them by email, we will incorporate them accordingly.

Please also suggest a striking image or visual abstract to illustrate your article as a PNG file 550 px wide x 300-600 px high.

16) As part of the EMBO Publications transparent editorial process initiative (see our Editorial at http://embomolmed.embopress.org/content/2/9/329), EMBO Molecular Medicine will publish online a Review Process File (RPF) to accompany accepted manuscripts.
In the event of acceptance, this file will be published in conjunction with your paper and will include the anonymous referee reports, your point-by-point response and all pertinent correspondence relating to the manuscript. Let us know whether you agree with the publication of the RPF and as here, if you want to remove or not any figures from it prior to publication.
Please note that the Authors checklist will be published at the end of the RPF.

EMBO Molecular Medicine has a "scooping protection" policy, whereby similar findings that are published by others during review or revision are not a criterion for rejection. Should you decide to submit a revised version, I do ask that you get in touch after three months if you have not completed it, to update us on the status.

I look forward to receiving your revised manuscript.

With kind regards,

Lise Roth

Lise Roth, PhD
Senior Editor
EMBO Molecular Medicine

***** Reviewer's comments *****

Referee #1 (Comments on Novelty/Model System for Author):

At this stage, some elements of characterization are lacking or are not fully convincing to assess the relevance of this model.

Referee #1 (Remarks for Author):

In this manuscript, Lago et al. describe the establishment of a pipeline for the derivation of brain tumoroids from pediatric patients. The establishment of such models is definitively a major challenge in pediatric neuro-oncology, and their work paves the way towards a biocollection of ependymoma, medulloblastoma, and low-grade glioma models, needed for both basic and pr-clinical research.
However, some concerns must be addressed to strengthen the robustness of their models and their relevance to the concept of patient-derived organoids:

1. Although genomic and epigenetic profilings of tumors/PDO are rather convincing, it is not the case for histological characterization presented in EV4, for one pair of tumor/PDO only. Preservation of histology is a prerequisite to guarantee the robustness of the models. Histological characterization of at least two PDOs for each tumor type must be shown, with both H&E and markers routinely used in the clinic. Authors must also check at least for two samples of each type that the transcriptome profile of each PDO corresponds to its tumor of origin, using PCA or unsupervised hierarchical clustering approach.
2. Since the notion of "days of culture" can be confusing, it should be replaced by the passages (i.e. number of splits) at which experiments were made. A table indicating for each PDO, the number of passages to which cultures have been/are maintained could also be helpful.
3. Authors provide elements indicating that their PDO medium improves the robustness of model derivation compared to already published protocols. The fact that three different tumor types can be derived in the same medium is intriguing and authors should comment on this, especially considering the fact that the authors mentioned that their "culture conditions are not specifically meant to preserve these types of cellular populations".
4. Assessing the sensitivity of PDOs to drugs is a key step toward their use in preclinical settings. If possible, authors should compare the drug response profile of PDOs from two medulloblastoma patients, for example, one good and one poor responder.

Referee #2 (Remarks for Author):

I appreciate the authors' effort of comparison different culture medium and additional tumor PDOs. By presenting the results of culture medium from Abdullah et al. 2022 and Jacob et al. 2020, the authors showed their own culture conditions are better suitable for PF EPN and MB. The authors also further clarify the tumor lines they used for each test, making the paper more readable and consistent. They did TSO500 of 5 PDOs, scRNA analysis of one MB tumor/PDO to support the robustness of their culture system. More importantly, the authors mimicked the standard of care treatment for ST-EPN and G3 MB using the corresponding derived PDOs, which strengthens the impact of usage of PDOs. Quality of figures are improved.
Unfortunately, these efforts couldn't improve the generation of LGG PDOs, which limits the full analysis of LGG PDOs. With full discussion of the possible reasons of low generation rate of LGG PDOs, I think the manuscript is worth to be published and will provide meaningful insights for the field.

Referee #3 (Remarks for Author):

The manuscript presented after adding the revisions should be considered for publication. The topic is of major interest and this effort should help to increase research efforts on pediatric brain tumors. Obviously I am assuming that the authors by reporting them, they will be open to share these models with the scientific community. Otherwise I do not see the potential of the current work.

Rebuttal

Referee #1 (Comments on Novelty/Model System for Author):

At this stage, some elements of characterization are lacking or are not fully convincing to assess the relevance of this model.

Referee #1 (Remarks for Author):

In this manuscript, Lago et al. describe the establishment of a pipeline for the derivation of brain tumoroids from pediatric patients. The establishment of such models is definitively a major challenge in pediatric neuro-oncology, and their work paves the way towards a biocollection of ependymoma, medulloblastoma, and low-grade glioma models, needed for both basic and pr-clinical research.
However, some concerns must be addressed to strengthen the robustness of their models and their relevance to the concept of patient-derived organoids:
1. Although genomic and epigenetic profilings of tumors/PDO are rather convincing, it is not the case for histological characterization presented in EV4, for one pair of tumor/PDO only. Preservation of histology is a prerequisite to guarantee the robustness of the models. Histological characterization of at least two PDOs for each tumor type must be shown, with both H&E and markers routinely used in the clinic.
We agree with reviewer's comment and we added in New Figure EV4 additional panels for 3 additional tumors:
1 PF ependymoma, Group A (relapse),
1 Medulloblastoma G4
1 Pilocytic Astrocytoma
Therefore, now we have two PDOs for each tumor type, with both H&E and markers routinely used in the clinic.

Authors must also check at least for two samples of each type that the transcriptome profile of each PDO corresponds to its tumor of origin, using PCA or unsupervised hierarchical clustering approach.
We agree with the reviewer's comment. However, in the previous revision we tried to extract RNA from the primary tumors in paraffin sections, but unfortunately, we were not able to obtain good quality RNA for further analyses. Therefore, we cannot perform such kind of analyses for primary tumors/matching PDOs.

2. Since the notion of "days of culture" can be confusing, it should be replaced by the passages (i.e. number of splits) at which experiments were made. A table indicating for each PDO, the number of passages to which cultures have been/are maintained could also be helpful.
We agree with the reviewer's comment. We replaced the "days of culture" notion with the passages, whenever possible. Indeed, some PDOs were kept in culture for many days but without actually splitting them through mechanical cutting (e.g., tumor #5, 6 and 7). Other PDOs, e.g. tumor #4, were split just twice. We added this information as a column in Table 1.

	Tumor	Analysis endpoint/Passages
EPENDYMOMAS		
1	PF ependymoma, Group A	Day 61

2	PF ependymoma, Group A (relapse)	Day 63
3	PF ependymoma, Group A	p9 (Day 349)
4	PF ependymoma, Group A	p2 (Day 228)
5	Sopratentorial ependymoma ZFTA-RELA fusion	Day 133
6	PF ependymoma, Group A (relapse)	Day 99
7	PF ependymoma, Group A (relapse)	Day 137
MEDULLOBLASTOMAS		
8	Group 4 medulloblastoma (relapse)	Day 28
9	SHH medulloblastoma	Day 28
10	SHH medulloblastoma	Day 33
11	Group 4 medulloblastoma	Day 28
12	Group 4 medulloblastoma	Day 35
13	Group 4 medulloblastoma	Day 28
14	Group 3 medulloblastoma	p21 (~2 years) and still ongoing
15	Group 3 medulloblastoma	Day 61
LOW-GRADE GLIAL TUMORS		
16	Low grade glioma with FGFR1-TACC1 fusion	Day 28
17	Dysembryoplastic neuroepithelial tumor (relapse)	Day 28
18	Ganglioglioma (relapse)	Day 28
19	Pilocytic astrocytoma (relapse)	Day 28
20	Pilocytic astrocytoma (relapse)	Day 28
21	Pilocytic astrocytoma (relapse)	Day 28
22	Pilocytic astrocytoma	Day 28
23	Polymorphous low-grade neuroepithelial tumor PLNTY	Day 61

3. Authors provide elements indicating that their PDO medium improves the robustness of model derivation compared to already published protocols. The fact that three different tumor types can be derived in the same medium is intriguing and authors should comment on this, especially considering the fact that the authors mentioned that their "culture conditions are not specifically meant to preserve these types of cellular populations".

We thank the reviewer for the comment. We now add a sentence in the discussion, where we point out that the proposed protocol is not optimal for low grade tumors:

“Depending on the type of primary tumor (i.e., EPN, MB or LGG tumors), tumor pieces usually formed more round organoids (PDOs) within one week. Indeed, a difference has been observed in the generation of PDOs depending on the type of original parental tumor. In general, EPN and MB tumors survived to the in vitro culture, could be kept in culture for longer periods of time and could be more easily amplified for many passages. On the contrary, LGG tumors displayed more difficulties in adapting to the in vitro culture and

could be kept in culture for shorter periods of time, apart for some specific cases (e.g., tumor #23, which could be kept in culture up to two months)."

4. Assessing the sensitivity of PDOs to drugs is a key step toward their use in preclinical settings. If possible, authors should compare the drug response profile of PDOs from two medulloblastoma patients, for example, one good and one poor responder.
We are sorry that we did not highlight this experiment in the new manuscript, but we have already tested the drug response to the adapted high-risk medulloblastoma protocol of two medulloblastoma-derived PDXOs as shown in Fig EV10. We observed a good response to the treatment in one sample (tumor A), while a poor response was observed in the other sample (tumor C). We highlighted this aspect also in the discussion: "The same happened for G3 MB PDOs that were subjected to the high-risk medulloblastoma protocol (Gandola et al., 2009; Massimino et al., 2013). Interestingly, when injected in immunodeficient mice, the treated PDOs could not engraft, suggesting a response mirroring the patient's one, currently in follow-up with non-evident disease. Instead, when applied to two different MB-derived PDXOs, the treatment analysis suggested a specific response of PDXOs to this protocol, probably depending on the patient from which they were derived.".

Referee #2 (Remarks for Author):

I appreciate the authors' effort of comparison different culture medium and additional tumor PDOs. By presenting the results of culture medium from Abdullah et al. 2022 and Jacob et al. 2020, the authors showed their own culture conditions are better suitable for PF EPN and MB. The authors also further clarify the tumor lines they used for each test, making the paper more readable and consistent. They did TSO500 of 5 PDOs, scRNA analysis of one MB tumor/PDO to support the robustness of their culture system. More importantly, the authors mimicked the standard of care treatment for ST-EPN and G3 MB using the corresponding derived PDOs, which strengthens the impact of usage of PDOs. Quality of figures are improved.
We thank the reviewer for the positive feedback. We added the table with the clarification of which PDOs line was used for each test as Table 12 (Materials and Methods).

Unfortunately, these efforts couldn't improve the generation of LGG PDOs, which limits the full analysis of LGG PDOs. With full discussion of the possible reasons of low generation rate of LGG PDOs, I think the manuscript is worth to be published and will provide meaningful insights for the field.
We thank the reviewer, we discussed more this aspect in the discussion:
“Depending on the type of primary tumor (i.e., EPN, MB or LGG tumors), tumor pieces usually formed more round organoids (PDOs) within one week. Indeed, a difference has been observed in the generation of PDOs depending on the type of original parental tumor. In general, EPN and MB tumors survived to the in vitro culture, could be kept in culture for longer periods of time and could be more easily amplified for many passages. On the contrary, LGG tumors displayed more difficulties in adapting to the in vitro culture and could be kept in culture for shorter periods of time, apart for some specific cases (e.g., tumor #23, which could be kept in culture up to two months). Indeed, this could be due to the intrinsic nature of LGG tumors that in general present lower aggressiveness, growth, and capacity of infiltrating (K. L. Collins and Pollack 2020). On the other hand, PF EPN, ST EPN with ZFTA-RELA fusion, Group 3 MB with MYC amplification and SHH MB with MYCN amplification and TP53 mutations are known to be highly aggressive and consequently leading to poor prognoses, and their mutational background could influence their maintenance in vitro.”

Referee #3 (Remarks for Author):
The manuscript presented after adding the revisions should be considered for publication. The topic is of major interest and this effort should help to increase research efforts on pediatric brain tumors. Obviously I am assuming that the authors by reporting them, they will be open to share these models with the scientific community. Otherwise I do not see the potential of the current work.
We thank the reviewer for the positive feedback. Indeed, PDOs and PDXOs could be made available to the scientific community.

17th Oct 2023

Dear Luca,

We have now received the feedback from referee #1 on your revised manuscript. As you will see below, this referee is supportive of publication pending minor revisions, and I will therefore be able to accept your manuscript once the following points will be addressed:

1/ Referee's comments:
Please address the remaining concerns from referee #1.

2/ Manuscript text:
- Please accept previous changes and only keep in track changes mode any new modification.
- We note that you currently have together with you, a total of 3 co-corresponding authors. Is that correct? Do you confirm equal contribution of these 3 people, able to take full responsibility for the paper and its content?
- Please note that the email bounced for author Sofia Reddel.
- Please suggest up to 5 keywords.
- Material and methods: This section should be placed after the Discussion.
o Human subjects: please include the full sentence that the experiments conformed to the principles set out in the WMA Declaration of Helsinki and the Department of Health and Human Services Belmont Report.
o Animal models: please provide the housing and husbandry conditions. Please indicate the age and gender of the animals at time of experiments.
o Antibodies: please add a title/legend to the tables.
o Statistics: please include a statement on randomization and exclusion/inclusion criteria.
- Data Availability section: we kindly remind you that datasets must be publicly available before acceptance of the manuscript. Please provide a link for the deposited human clinical and genomic datasets.
- Acknowledgements: Please make sure that the information provided in the manuscript matches the information provided in the submission system (My First AIRC, CARITRO, Accelerator Award - Cancer Research UK/AIRC - INCAR project, AIRC-Special Project 5×1000 no. 9962, AIRC IG 2018 id. 21724, Ricerca Finalizzata, No. GR-2018-12367328, Ministry of Health 5x1000 202205_ONCO_MIELE, Fondazione Heal are currently missing from the submission system).
- Please remove "Supporting Information"
- Please reformat the references to have 10 authors listed before et al. and remove the DOIs.
- The legends for Tables 1-12 should be removed from the manuscript file and be in the tables files only.

3/ Figures and Appendix:
- Please provide exact p values, not a range, in the figures or their legends.
- Tables 6 and 7 should be renamed Dataset EV1 and Dataset EV2 and uploaded as two separate excel files. Tables 1-5 and 8-12 should be uploaded as separate files and the numbering be adjusted.
- Please note that figure legends for figure 3 are not in alphabetical order.
- Please note that scale bar and its definition are missing for figures 6E', E'''

4/ Checklist:
- Please adjust the statistics section according to the Materials and Methods.
- Please fill-in the section: Sample definition - number of times the experiment was replicated.

5/ Please note that all corresponding authors are required to supply an ORCID ID for their name upon submission of a revised manuscript. An ORCID identifier is currently missing for M. Kool.

6/ The paper explained: please let me know if you agree with the following text, or amend as you see fit:
Problem
CNS tumors are the most common pediatric solid tumors and the main cause of childhood cancer-related deaths. In recent years, research efforts have improved our understanding of the underlying molecular landscape, contributing to clinical advances, and improving patient life expectancy. However, developing new models that can fully resemble the heterogeneity of these tumors to test personalized treatment strategies remains a challenge.

Results
Patient-derived organoids (PDOs) and patient-derived xenograft organoids (PDXOs) were established through direct in vitro culture of primary ependymomas, medulloblastomas and low-grade glial tumors, and from medulloblastoma xenografts, respectively.
PDOs and PDXOs were shown to be bona fide replicates of their corresponding primary tumors through genome and mutational status analysis, and by DNA methylation profiling. They also maintained the tumoral heterogeneity and the cellular morphology

and architecture of the primary tumors, as shown by sc-RNA sequencing and immunohistological analysis. PDOs and PDXOs could be largely amplified and biobanked, maintaining the features of their parental tumors, even after many passages in culture. The translational potential of this model was shown by treating PDOs and PDXOs with the same therapeutic regimens as the corresponding patients, as the organoids exhibited similar response to a specific clinical treatment.

Impact
These PDOs and PDXOs, derived from human tumors for which there are currently very limited in vivo and in vitro models, constitute an important proof of concept of their translational application as a reliable tool for wide drug screening, and more generally for personalized medicine.

7/ Synopsis:
I introduced minor modifications in your text to match our style and format. Please let me know if you agree with the following or amend as you see fit:

Patient-Derived Organoids (PDOs) and Patient-Derived Xenograft Organoids (PDXOs) have been established through direct in vitro culture of primary tumors and patient derived xenograft (PDX)-derived tumors with the goal to better model pediatric tumors of the central nervous system.

• PDOs recapitulate cellular heterogeneity, histological features, mutational profiles (number of variants, microsatellite instability, tumor mutational burden) of the corresponding parental tumor even after one year of in vitro culture
• ZFTA-RELA EPN- and G3 MB-PDOs recapitulate patients' response to routinely applied therapeutic regimens
• Several PDOs and PDXOs were derived from human tumors for which there are currently very limited in vivo and in vitro models available
• PDOs and PDXOs are available to the research community, can be subjected to genetic manipulations, to drug screening and translational applications for personalized medicine

8/ As part of the EMBO Publications transparent editorial process initiative (see our Editorial at http://embomolmed.embopress.org/content/2/9/329), EMBO Molecular Medicine will publish online a Review Process File (RPF) to accompany accepted manuscripts.
This file will be published in conjunction with your paper and will include the anonymous referee reports, your point-by-point response and all pertinent correspondence relating to the manuscript. Let us know whether you agree with the publication of the RPF and as here, if you want to remove or not any figures from it prior to publication.
Please note that the Authors checklist will be published at the end of the RPF.

I look forward to receiving your revised manuscript as soon as possible.

With kind regards,

Lise

Lise Roth, PhD
Senior Editor
EMBO Molecular Medicine

***** Reviewer's comments *****

Referee #1 (Remarks for Author):

The revised version of the manuscript takes account of the comments made, but 2 decisive points have not been addressed due to technical limitations (RNAseq) or are not very convincing (histology). The histological analyses presented in EV4 do not clearly show the similarity between the organoids and their original tumor. The absence of comparative RNAseq is also detrimental.

However, given the importance of this work in advancing the modeling of these rare tumors, I propose that two sentences be added to the discussion to acknowledge these limitations, i.e. 1) the fact that the histology very incompletely reproduces that of the original tumor, and that 2) transcriptomic analysis will be necessary to confirm the adequacy and robustness of the models at the molecular level. The same applies to splitting, since some tumors have not been split for more than 100 days, which may

imply that they have not grown sufficiently? This point needs to be discussed quickly.
Once these points have been modified, I think the manuscript is worth to be published.

***** Reviewer's comments *****
Referee #1 (Remarks for Author):

The revised version of the manuscript takes account of the comments made, but 2 decisive points have not been addressed due to technical limitations (RNAseq) or are not very convincing (histology). The histological analyses presented in EV4 do not clearly show the similarity between the organoids and their original tumor. The absence of comparative RNAseq is also detrimental.

However, given the importance of this work in advancing the modeling of these rare tumors, I propose that two sentences be added to the discussion to acknowledge these limitations, i.e. 1) the fact that the histology very incompletely reproduces that of the original tumor, and that 2) transcriptomic analysis will be necessary to confirm the adequacy and robustness of the models at the molecular level. The same applies to splitting, since some tumors have not been split for more than 100 days, which may imply that they have not grown sufficiently? This point needs to be discussed quickly.

Once these points have been modified, I think the manuscript is worth to be published.

We thank the reviewer for the comment, we added the suggested sentences in the Results and Discussion sections.

24th Oct 2023

Dear Luca,

Thank you for submitting your revised files and addressing the last editorial concerns. I am pleased to inform you that your manuscript is accepted for publication and is now being sent to our publisher to be included in the next available issue of EMBO Molecular Medicine!

Regarding data deposition: Thank you for depositing your datasets on GEO. You will be able to change the current zenodo links for GEO links at proofing stage.

Please read below for additional IMPORTANT information regarding your article, its publication and the production process.

Congratulations on your interesting work!

With kind regards,

Lise

Lise Roth, Ph.D
Senior Editor
EMBO Molecular Medicine

Follow us on Twitter @EmboMolMed
Sign up for eTOCs at embopress.org/alertsfeeds

*** *** *** IMPORTANT INFORMATION *** *** ***

SPEED OF PUBLICATION
The journal aims for rapid publication of papers, using using the advance online publication "Early View" to expedite the process: A properly copy-edited and formatted version will be published as "Early View" after the proofs have been corrected. Please help the Editors and publisher avoid delays by providing e-mail address(es), telephone and fax numbers at which author(s) can be contacted.

Should you be planning a Press Release on your article, please get in contact with embomolmed@wiley.com as early as possible, in order to coordinate publication and release dates.

LICENSE AND PAYMENT:

All articles published in EMBO Molecular Medicine are fully open access: immediately and freely available to read, download and share.

EMBO Molecular Medicine charges an article processing charge (APC) to cover the publication costs. You, as the corresponding author for this manuscript, should have already received a quote with the article processing fee separately. Please let us know in case this quote has not been received.

Once your article is at Wiley for editorial production you will receive an email from Wiley's Author Services system, which will ask you to log in and will present you with the publication license form for completion. Within the same system the publication fee can be paid by credit card, an invoice, pro forma invoice or purchase order can be requested.

Payment of the publication charge and the signed Open Access Agreement form must be received before the article can be published online.

PROOFS

You will receive the proofs by e-mail approximately 2 weeks after all relevant files have been sent o our Production Office. Please return them within 48 hours and if there should be any problems, please contact the production office at embopressproduction@wiley.com.

Please inform us if there is likely to be any difficulty in reaching you at the above address at that time. Failure to meet our deadlines may result in a delay of publication.

All further communications concerning your paper proofs should quote reference number EMM-2023-18199-V3 and be directed to the production office at embopressproduction@wiley.com.

EMBO Press Author Checklist

Corresponding Author Name: Luca Tiberi	
Journal Submitted to: Embo Molecular Medicine	
Manuscript Number: EMM-2023-18199	

USEFUL LINKS FOR COMPLETING THIS FORM
The EMBO Journal - Author Guidelines
EMBO Reports - Author Guidelines
Molecular Systems Biology - Author Guidelines
EMBO Molecular Medicine - Author Guidelines

Reporting Checklist for Life Science Articles (updated January
This checklist is adapted from Materials Design Analysis Reporting (MDAR) Checklist for Authors. MDAR establishes a minimum set of requirements in transparent reporting in the life sciences (see Statement of Task: 10.31222/osf.io/9sm4x). Please follow the journal's guidelines in preparing your
Please note that a copy of this checklist will be published alongside your article.

Abridged guidelines for figures
1. Data
The data shown in figures should satisfy the following conditions:
- the data were obtained and processed according to the field's best practice and are presented to reflect the results of the experiments in an accurate and unbiased manner.
- ideally, figure panels should include only measurements that are directly comparable to each other and obtained with the same assay.
- plots include clearly labeled error bars for independent experiments and sample sizes. Unless justified, error bars should not be shown for technical
- if n<5, the individual data points from each experiment should be plotted. Any statistical test employed should be justified.
- Source Data should be included to report the data underlying figures according to the guidelines set out in the authorship guidelines on Data

2. Captions
Each figure caption should contain the following information, for each panel where they are relevant:
- a specification of the experimental system investigated (eg cell line, species name).
- the assay(s) and method(s) used to carry out the reported observations and measurements.
- an explicit mention of the biological and chemical entity(ies) that are being measured.
- an explicit mention of the biological and chemical entity(ies) that are altered/varied/perturbed in a controlled manner.
- the exact sample size (n) for each experimental group/condition, given as a number, not a range;
- a description of the sample collection allowing the reader to understand whether the samples represent technical or biological replicates (including how many animals, litters, cultures, etc.).
- a statement of how many times the experiment shown was independently replicated in the laboratory.
- definitions of statistical methods and measures:
  - common tests, such as t-test (please specify whether paired vs. unpaired), simple χ2 tests, Wilcoxon and Mann-Whitney tests, can be unambiguously identified by name only, but more complex techniques should be described in the methods section;
  - are tests one-sided or two-sided?
  - are there adjustments for multiple comparisons?
  - exact statistical test results, e.g., P values = x but not P values < x;
  - definition of 'center values' as median or average;
  - definition of error bars as s.d. or s.e.m.

Please complete ALL of the questions below.
Select "Not Applicable" only when the requested information is not relevant for your study.

Materials

Newly Created Materials	Information included in the manuscript?	In which section is the information available? (Reagents and Tools Table, Materials and Methods, Figures, Data Availability Section)
New materials and reagents need to be available; do any restrictions apply?	Yes	Materials and Methods, Human subjects

Antibodies	Information included in the manuscript?	In which section is the information available? (Reagents and Tools Table, Materials and Methods, Figures, Data Availability Section)
For **antibodies** provide the following information:<br>- Commercial antibodies: RRID (if possible) or supplier name, catalogue number and or/clone number<br>- Non-commercial: RRID or citation	Yes	Materials and Methods, Histological and immunohistochemical analysis

DNA and RNA sequences	Information included in the manuscript?	In which section is the information available? (Reagents and Tools Table, Materials and Methods, Figures, Data Availability Section)
**Short novel DNA or RNA including primers, probes**: provide the sequences.	Not Applicable	

Cell materials	Information included in the manuscript?	In which section is the information available? (Reagents and Tools Table, Materials and Methods, Figures, Data Availability Section)
**Cell lines:** Provide species information, strain. Provide accession number in repository **OR** supplier name, catalog number, clone number, and/**OR** RRID.	Not Applicable	
**Primary cultures:** Provide species, strain, sex of origin, genetic modification status.	Not Applicable	
Report if the cell lines were recently **authenticated** (e.g., by STR profiling) and tested for mycoplasma contamination.	Not Applicable	

Experimental animals	Information included in the manuscript?	In which section is the information available? (Reagents and Tools Table, Materials and Methods, Figures, Data Availability Section)
**Laboratory animals or Model organisms:** Provide species, strain, sex, age, genetic modification status. Provide accession number in repository **OR** supplier name, catalog number, clone number, **OR** RRID.	Yes	Materials and Methods, Animal models and Animals and orthotopic engraftment of PDOs
**Animal observed in or captured from the field:** Provide species, sex, and age where possible.	Not Applicable	
Please detail **housing and husbandry conditions**.	Yes	Material and Methods, Animal models

Plants and microbes	Information included in the manuscript?	In which section is the information available? (Reagents and Tools Table, Materials and Methods, Figures, Data Availability Section)
**Plants:** provide species and strain, ecotype and cultivar where relevant, unique accession number if available, and source (including location for collected wild specimens).	Not Applicable	
**Microbes:** provide species and strain, unique accession number if available, and source.	Not Applicable	

Human research participants	Information included in the manuscript?	In which section is the information available? (Reagents and Tools Table, Materials and Methods, Figures, Data Availability Section)
If collected and within the bounds of privacy constraints report on age, sex and gender or ethnicity for all study participants.	Yes	Materials and Methods, Human subjects<br>Table 1

Core facilities	Information included in the manuscript?	In which section is the information available? (Reagents and Tools Table, Materials and Methods, Figures, Data Availability Section)
If your work benefited from core facilities, was their service mentioned in the acknowledgments section?	Yes	Acknowledgments

Design

Study protocol	Information included in the manuscript?	In which section is the information available? (Reagents and Tools Table, Materials and Methods, Figures, Data Availability Section)
If study protocol has been **pre-registered, provide DOI in the manuscript**. For clinical trials, provide the trial registration number **OR** cite DOI.	Not Applicable	
Report the **clinical trial registration number** (at ClinicalTrials.gov or equivalent), where applicable.	Not Applicable	

Laboratory protocol	Information included in the manuscript?	In which section is the information available? (Reagents and Tools Table, Materials and Methods, Figures, Data Availability Section)
Provide DOI OR other citation details if **external detailed step-by-step protocols** are available.	Not Applicable	

Experimental study design and statistics	Information included in the manuscript?	In which section is the information available? (Reagents and Tools Table, Materials and Methods, Figures, Data Availability Section)
Include a statement about **sample size** estimate even if no statistical methods were used.	Yes	Material and Methods, Figures
Were any steps taken to minimize the effects of subjective bias when allocating animals/samples to treatment (e.g. **randomization procedure**)? If yes, have they been described?	Yes	Material and Methods
Include a statement about **blinding** even if no blinding was done.	Yes	Material and Methods
Describe **inclusion/exclusion criteria** if samples or animals were excluded from the analysis. Were the criteria pre-established?<br><br>If sample or data points were omitted from analysis, report if this was due to attrition or intentional exclusion and provide justification.	Yes	Material and Methods
For every figure, are **statistical tests** justified as appropriate? Do the data meet the assumptions of the tests (e.g., normal distribution)? Describe any methods used to assess it. Is there an estimate of variation within each group of data? Is the variance similar between the groups that are being statistically compared?	Yes	Material and Methods, Figures

Sample definition and in-laboratory replication	Information included in the manuscript?	In which section is the information available? (Reagents and Tools Table, Materials and Methods, Figures, Data Availability Section)
In the figure legends: state number of times the experiment was **replicated** in laboratory.	Yes	Figure Legends
In the figure legends: define whether data describe **technical or biological replicates**.	Yes	Materials and Methods, Figure Legends

Ethics

Ethics	Information included in the manuscript?	In which section is the information available? (Reagents and Tools Table, Materials and Methods, Figures, Data Availability Section)
Studies involving **human participants**: State details of **authority granting ethics approval** (IRB or equivalent committee(s), provide reference number for approval.	Yes	Materials and Methods
Studies involving **human participants**: Include a statement confirming that **informed consent** was obtained from all subjects and that the experiments conformed to the principles set out in the WMA Declaration of Helsinki and the Department of Health and Human Services Belmont Report.	Yes	Materials and Methods
Studies involving **human participants: For publication of patient photos**, include a statement confirming that consent to publish was obtained.	Yes	Materials and Methods
Studies involving experimental **animals**: State details of **authority granting ethics approval** (IRB or equivalent committee(s), provide reference number for approval. Include a statement of compliance with ethical regulations.	Not Applicable	
Studies involving **specimen and field samples:** State if relevant **permits** obtained, provide details of authority approving study; if none were required, explain why.	Not Applicable	

Dual Use Research of Concern (DURC)	Information included in the manuscript?	In which section is the information available? (Reagents and Tools Table, Materials and Methods, Figures, Data Availability Section)
Could your study fall under dual use research restrictions? Please check biosecurity documents and list of **select agents and toxins** (CDC): https://www.selectagents.gov/sat/list.htm	Not Applicable	
If you used a select agent, is the security level of the lab appropriate and reported in the manuscript?	Not Applicable	
If a study is subject to dual use research of concern regulations, is the name of the **authority granting approval and reference number** for the regulatory approval provided in the manuscript?	Not Applicable	

Reporting

The MDAR framework recommends adoption of discipline-specific guidelines, established and endorsed through community initiatives. Journals have their own policy about requiring specific guidelines and recommendations to complement MDAR.

Adherence to community standards	Information included in the manuscript?	In which section is the information available? (Reagents and Tools Table, Materials and Methods, Figures, Data Availability Section)
State if relevant guidelines or checklists (e.g., **ICMJE, MIBBI, ARRIVE, PRISMA**) have been followed or provided.	Not Applicable	
For **tumor marker prognostic studies,** we recommend that you follow the **REMARK** reporting guidelines (see link list at top right). See author guidelines, under 'Reporting Guidelines'. Please confirm you have followed these guidelines.	Not Applicable	
For **phase II and III randomized controlled trials**, please refer to the **CONSORT** flow diagram (see link list at top right) and submit the CONSORT checklist (see link list at top right) with your submission. See author guidelines, under 'Reporting Guidelines'. Please confirm you have submitted this list.	Not Applicable	

Data Availability

Data availability	Information included in the manuscript?	In which section is the information available? (Reagents and Tools Table, Materials and Methods, Figures, Data Availability Section)
Have **primary datasets** been deposited according to the journal's guidelines (see 'Data Deposition' section) and the respective accession numbers provided in the Data Availability Section?	Yes	Data Availability
Were **human clinical and genomic datasets** deposited in a public access-controlled repository in accordance to ethical obligations to the patients and to the applicable consent agreement?	Yes	Data Availability
Are **computational models** that are central and integral to a study available without restrictions in a machine-readable form? Were the relevant accession numbers or links provided?	Not Applicable	
If publicly available data were reused, provide the respective **data citations in the reference list**.	Not Applicable	
